# Mechanism of 3‑*O*‑Acyl-Directed α‑Mannopyranosylation and Rationalization of the Contrasting Behavior of 3‑*O*‑Acyl Glucopyranosyl Donors

**DOI:** 10.1021/acs.joc.5c01978

**Published:** 2025-10-06

**Authors:** Shuay Abdullayev, David Crich

**Affiliations:** † Department of Pharmaceutical and Biomedical Sciences, 1355 University of Georgia, 250 West Green Street, Athens, Georgia 30602, United States; ‡ Complex Carbohydrate Research Center, University of Georgia, 315 Riverbend Road, Athens, Georgia 30602, United States; § Department of Chemistry, University of Georgia, 302 East Campus Road, Athens, Georgia 30602, United States

## Abstract

An investigation into the α-directing effect of 3-*O*-acyl groups in 4,6-*O*-benzylidene-directed mannopyranosylation is reported. No evidence was found by VT NMR experiments with ^13^C-enriched 3-*O*-benzoyl esters for the formation of a bridged ion. Inclusion of an axial 3-*C*-methyl group in the donors to destabilize the ester ground-state conformation and promote participation enabled the observation of a very minor bridged ion in the NMR experiments. In experiments conducted in the presence of diphenyl sulfoxide, the only species observed were the two mannosyl oxysulfonium ions, yet the reactions were still extremely α-selective. Overall, no support was found for participation by the ester at the 3-position as the main pathway for α-selectivity. An alternative hypothesis is advanced based on acceptor hydrogen bonding to the acyl group, which is oriented in its ground state toward the anomeric position and so is ideally placed to participate in such hydrogen bonding. The much weaker α-directing effect of a 3-*O*-acyl group in the glucopyranosyl series is explained by the steric clash with the equatorial substituent at C2, which interferes with the stereodirecting hydrogen bond, and by the ground-state conformation of the 3-*O*-acyl group that is less well-disposed for the acceptor–donor hydrogen bond.

## Graphical abstract

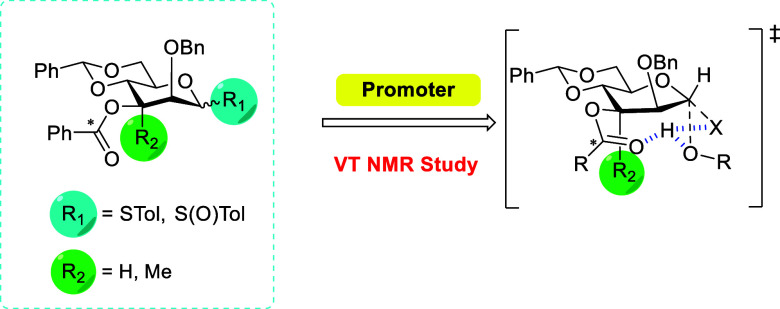

## Introduction

In 1996 and 1997, employing donors such as **1** carrying ether protecting groups on O2 and O3, we described the 4,6-*O*-benzylidene-directed β-mannopyranosylation of primary and secondary alcohols, respectively, so providing a practical, direct solution to one of the most enduring problems in carbohydrate chemistry.^1–3^ In an early demonstration of the power of low and variable temperature NMR spectroscopy in elucidating glycosylation reaction mechanisms,^4–8^ we rationalized our observations in terms of the initial formation of a covalent α-mannosyl triflate **2** with subsequent S_N_2-like displacement by the acceptor alcohol.^9^ In 2000, seeking to further stabilize the intermediate α-glycosyl triflate and thereby enhance β-selectivity, we studied a 4,6-*O*-benzylidene-protected donor **3** carrying an electron-withdrawing benzoate at the 3-position. Contrary to expectation, we found this donor to be very highly α-selective, leading us to suggest in a footnote the possibility of stereodirecting participation by the benzoate ester via a bridged bicyclic ion **4** with a ^1^S_5_ conformation.^10^ Exploiting our discoveries, in 2004, we described the synthesis of a repeating tetrasaccharide unit of an antigenic bacterial oligosaccharide containing both a β- and an α-d-rhamnopyranoside (6-deoxymannopyranoside) unit with excellent stereocontrol in both cases using donors **5** and **6**, differing only in substitution at the 3-position; in this synthesis, the α-substituted benzylidene acetal serves to control the anomeric selectivity before taking part in a regioselective radical fragmentation process to afford the targeted rhamnopyranosides.^11^ The excellent α-directing ability of the 3-*O*-(chloroacetyl) ester in donor **6** provoked our initial doubts as to the validity of the hypothesis of stereodirecting participation in 3-*O*-acyl-4,6-*O*-benzylidene mannopyranosyl donors because a chloromethyl substituent would be expected to stabilize a bridged bicyclic ion to a lesser extent than the phenyl group in **4**. These doubts were reinforced when we introduced the *tert*-butyloxycarbonate group and other esters as probes for remote or distal group participation (DGP) by esters, with the finding of negative results in all but the ideally disposed 3-*O*-*tert*-butyloxycarbonyl allopyranosyl donor **7**, which afforded the cyclic carbonate **8** on activation (Figure 1).^12^

**Figure 1:**
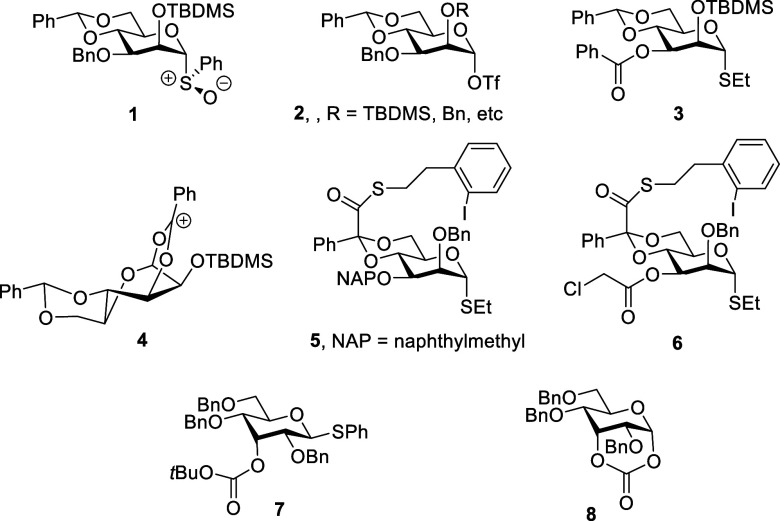
Key structures and intermediates considered in early studies on 4,6-*O*-benzylidene-directed β-mannopyranosylation.

Subsequently, looking to probe for DGP through a six-membered cyclic ion in an ideally disposed case, we synthesized and studied the *tert*-butyl and methyl esters **9** and **10**. Activation of **9** and addition of acceptor **11** afforded lactone **12**, confirming the possibility of bridged ion formation, but activation of **10** provided not the expected α-glycoside arising from participation by the methyl ester but β-mannoside **13**. This lead us to postulate an equilibrium between the fused ion **14** and the α-glycosyl triflate **15**, with loss of a *tert*-butyl cation from the bridged ion in the case of donor **9** but preferential displacement of the triflate by external alcohols in the case of **10** (Scheme 1).^13^

**Scheme 1:**
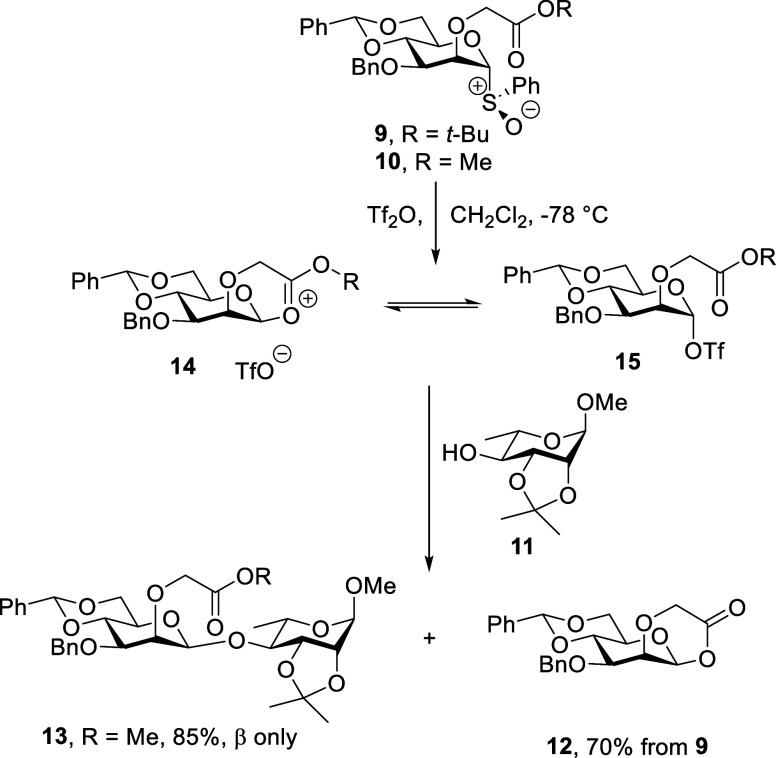
Probe of Ester Participation through a Fused Six-Membered Cyclic Dioxenium Ion

Others have argued strongly in favor of DGP on the basis of (i) (frequently minor) changes in selectivity on the replacement of remote ethers by esters in glycosyl donors; (ii) the formation of 1,3-oxaza-type bridged intermediates on the use of the more nucleophilic amides or imidates as probes; (iii) gas phase computations conducted in the absence of counterions, which necessarily exclude covalent donors from the equation; and (iv) gas phase experiments with IR detection of bridged ions, again with the obligatory exclusion of counterions and so covalent donors from consideration.^14–20^ Most recently, Boltje and co-workers described the use of ^1^H and ^13^C Chemical Exchange Saturation Transfer (CEST) NMR spectroscopy in conjunction with ^13^C enrichment of the carbonyl carbon to characterize the bicyclic bridged ions **16** and **17** but not **18**, with supporting Exchange NMR Spectroscopy (EXSY) measurements of the rate of triflate exchange. They concluded that “even though these (bridged bicyclic ions) are low population reaction intermediates and their exact abundance at equilibrium remains unknown, they can be the main product-forming species if the barrier to reaction intermediate interconversion is smaller than the barrier to product formation according to the Curtin–Hammett principle.” ^21–24^ Nevertheless, referring to the weaker α-directing effect of 3-*O*-acyl groups in the glucopyranosides,^14,15,18^ Boltje and co-workers acknowledged that their results “cannot exclude a role for the SSIP (solvent separated ion pair), which is expected to adopt an ^4^ *H*_3_ half chair conformation and drives the formation of the α-product, as reported by Codée and co-workers”^25^ as we had suggested previously for the mannose series.^18^ Building on their CEST NMR results in the mannopyranosyl series and their inability to detect bridged bicyclic ions analogous to **4** and **16**–**18** in the corresponding glucopyranosyl series, when only glucopyranosyl triflates were observed as intermediates, Boltje, Codée, and co-workers further suggested that the reduced α-directing effect of the 3-*O*-acyl groups in the glucopyranosyl series is due to the destabilization of the gluco-configured bridging ions and the transition states leading to them by the pseudoaxial ethers at the 2-position, as illustrated in **19** (Figure 2).^26^ In yet further work, Boltje and co-workers have similarly employed CEST NMR to rationalize the α-selective glycosylation of 3-*O*-acyl mannuronic acid donors carrying esters in terms of distal group participation.^27^

**Figure 2:**
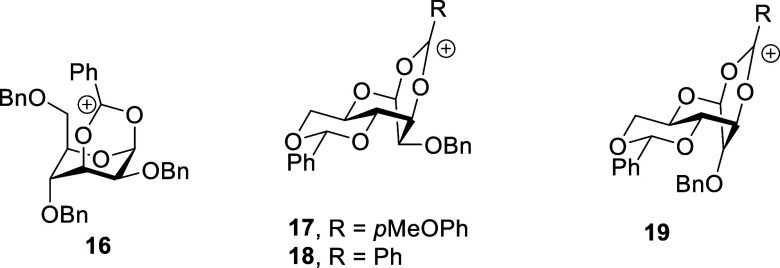
Bridged ions observed (**16** and **17**) or not (**18** and **19**) by CEST NMR by Boltje and Co-workers.^21,22^

For our part, building off long-established conformational preferences of esters of secondary alcohols in which the carbonyl group approximately eclipses the α–C–H bond of the alkoxy moiety^28–30^ and the barrier to rotation about the COO–CHR_2_ bond is calculated to be 4–12 kcal mol^–1^ (Figure 3),^31,32^ we have argued that DGP is disfavored by a series of unfavorable equilibria, including ionization of the covalent donor, conformational reorganization of the glycosyl oxocarbenium ion, and conformational reorganization of the ester group, before ring closure to any bridged bicyclic ion can take place, as illustrated for the frequently postulated participation by a 4-*O*-acyl group in the galactopyranose series in Figure 3.

**Figure 3:**
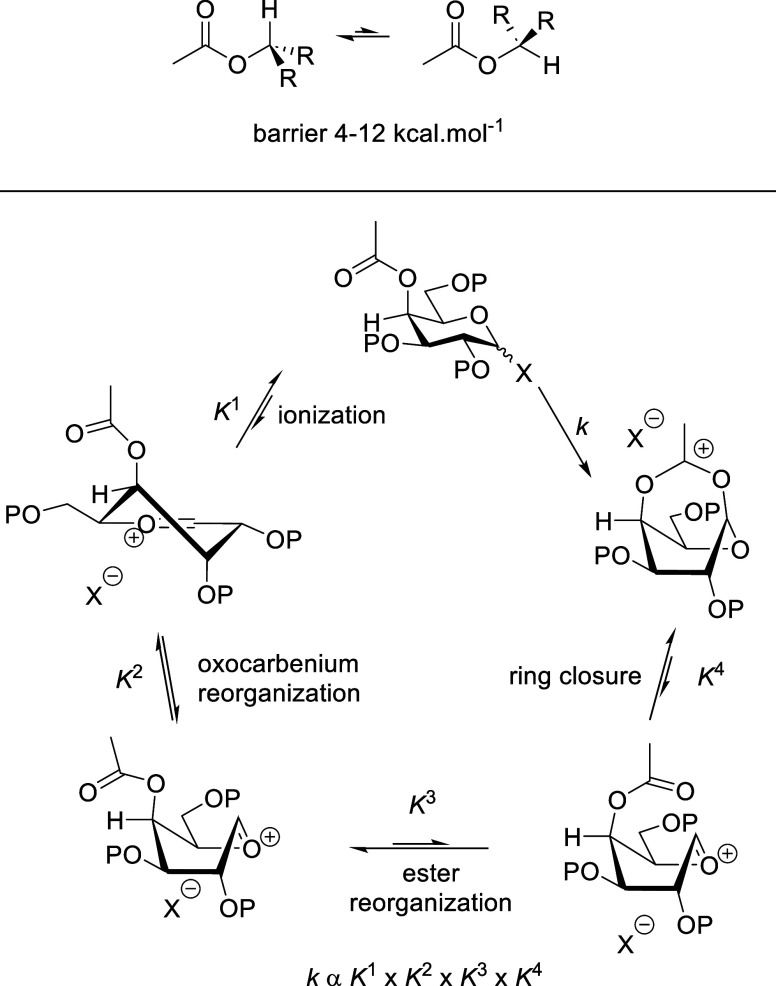
Established conformation of esters of secondary alcohols and the equilibria influencing DGP illustrated for a 4-*O*-acyl galactopyranosyl donor.

Building on the analysis of Figure 3, we suggested that the likelihood of DGP would be enhanced by removing any one or more of the three unfavorable equilibria, i.e., donor ionization, oxocarbenium ion reorganization, and/or ester reorganization, as in the above-mentioned gas phase calculations that start from the oxocarbenium ion and omit the counterion, or the gas phase experimental work using oxocarbenium ions generated in mass spectrometer and lacking the counterion.^14–18^ Following this line of thought, we reasoned that destabilization of the ester ground-state conformation by replacing the α–C–H bond in a secondary ester R_2_CH–O_2_CR′ by an additional α–C–C bond, giving a tertiary ester R_3_C–O_2_CR′, would enhance the likelihood of formation of bridged bicyclic ions. We tested this hypothesis by the synthesis of the 4-*C*-methyl-4-*O*-^13^C-benzoyl and 4-*C*-methyl-4-*O*-*tert*-butoxycarbonyl galactopyranosyl donors **20** and **21** and the desmethyl analogs **22** and **23**. Activation of **20** with triflic anhydride in CD_2_Cl_2_ at −80 °C resulted in clean formation of the bridged ion **24** that was stable to −40 °C, above which it decomposed with predominant formation of 1,6-anhydro derivative **25**. Parallel activation of desmethyl benzoate **22** did not provide any evidence for bridged ion formation but rather afforded mainly galactosyl triflate **26**, which decomposed above −20 °C to give **27** and **28**. Activation of the 4-*C*-methyl-4-*O*-Boc-protected donor **21** with diphenyl sulfoxide and triflic anhydride at −80 °C resulted in isolation of the bicyclic carbonate **29** in 56% yield, whereas the desmethyl analog **23** gave a complex reaction mixture under the same conditions in which we could not identify any corresponding cyclic carbonate and from which we were only able to isolate the Friedel–Crafts product **30** in low yield (Figure 4).^33,34^ In addition to providing the first direct NMR spectroscopic evidence for the formation of a bridged bicyclic ion arising from participation by a distal ester, these results clearly support the hypothesis of the role of ester conformational interconversion in the barrier to DGP.

**Figure 4:**
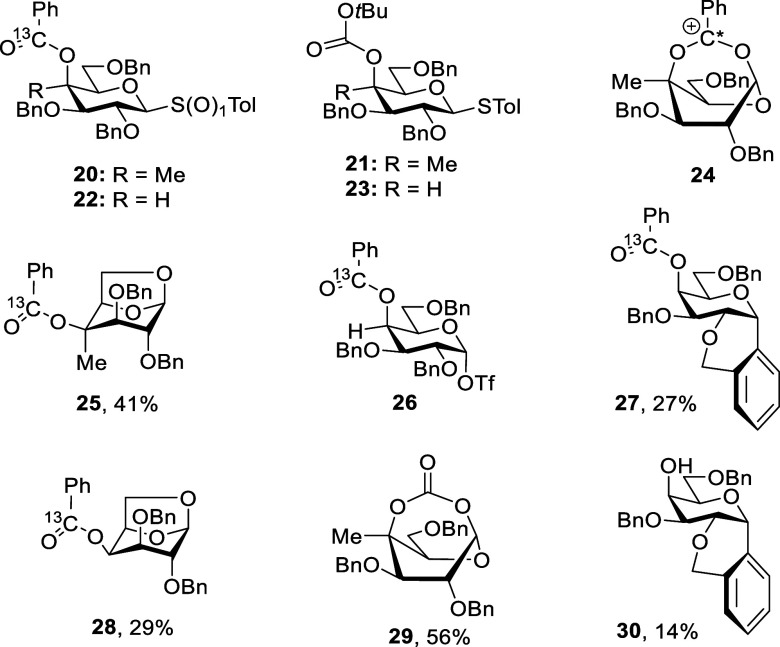
Structures, intermediates, and products establishing the role of ester conformation in the barrier to DGP by galactopyranosyl 4-*O*-acyl groups.

Stimulated by the ongoing controversy surrounding the mechanism of α-mannoside formation in 3-*O*-acyl mannopyranosyl donors and the associated formation of α-rhamnopyranosides from 3-*O*-acyl rhamnopyranosyl donors,^35^ and particularly those carrying the normally strongly β-directing 4,6-*O*-benzylidene acetal, and by the reduced nature of this effect in corresponding glucopyranosyl donors, we now report on the application of the same secondary to tertiary ester switch in probing the mechanism of α-glycoside formation with 4,6-*O*-benzylidene-3-*O*-acyl mannopyranosyl donors. Contrary to the work in the galactopyranose series (Figure 4), we find no evidence of the formation of bridged bicyclic ions at levels greater than 1% in the parent system and 4% in the 3-*C*-methyl system. We also conducted 3-*O*-acyl-directed mannopyranosylation reactions in the presence of excess diphenyl sulfoxide, when the more stable glycosyl oxysulfonium ions are formed in preference to the usual glycosyl triflates,^36–43^ and find that excellent α-selectivity is retained. Overall, we conclude that DGP is unlikely to be the source of the observed α-selectivity and propose an alternative explanation based on acceptor–donor hydrogen bonding at the transition state, in which the 3-*O*-acyl group retains its ground-state conformation.

## Results

### Synthesis of Glycosyl Donors

Anticipating difficulties arising from 1,3-diaxial interactions between the intended C3 axial methyl group and α-configured donors, we targeted β-thiomannosides as donors. To this end, the selectively protected mannopyranosyl β-thioglycoside **31**,^44^ prepared in five steps from acetobromomannose, was silylated on O2 to give **32**, before the PMB group was removed to afford **33**. Dess–Martin oxidation then gave the ketone **34**, which on treatment with methyllithium^45^ gave the 3-*C*-manno-alcohol **35** in 88% yield. Removal of the silyl ether under standard conditions gave diol **36** in 96% yield and was followed by regioselective monobenzylation under phase transfer conditions, which afforded 81% of the tertiary alcohol **37**. Esterification of **37** was achieved with benzoic acid, ^13^C-enriched benzoic acid, and *p*-nitrobenzoic acid with carbonyl diimidazole (CDI) in THF at reflux **38** giving ^13^C-enriched **38**, and **39**, each in excellent yield. This was followed by conversion to the corresponding sulfoxides **40**, ^13^C-enriched **40**, and **41**, each as mixtures of an unassigned less polar major and a more polar minor diastereomer consistent with the precedent for equatorial thioglycosides^46,47^ and in good yield (Scheme 2).

**Scheme 2:**
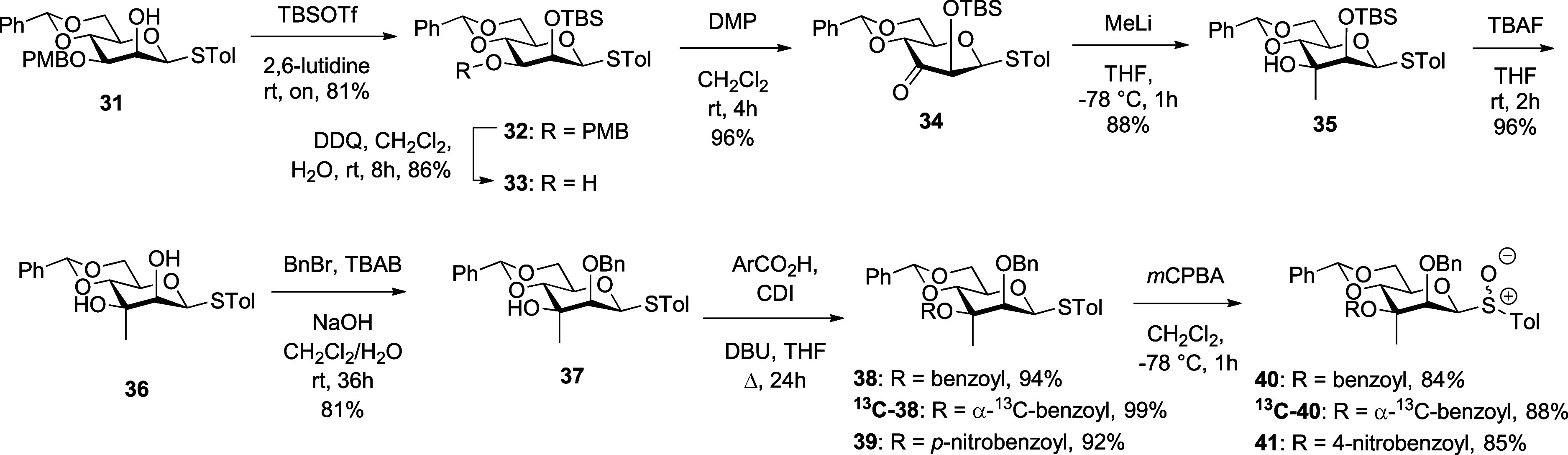
Synthesis of the Equatorial Glycosyl Sulfoxides **40**, ^**13**^ **C-40**, and **41**

The configuration at C3 of the thiomannopyranoside **35** and of all subsequent derivatives at O3 follows from NOE correlations between the methyl group and H’s 1, 2, and 5. Moreover, the ^3^ *J* coupling constants between H’s 1 and 2 (^3^ *J*_*H*1,*H*2_ = 1.2 Hz) and H’s 4 and 5 (^3^ *J*_*H*4,*H*5_ = 9.7 Hz) in all of these compounds are typical for β-mannopyranosides (Figure 5). Evidently, the 1,3-diaxial interactions present in these compounds are not sufficient to cause significant distortions from the standard ^4^ *C*_1_ chair conformation.

**Figure 5:**
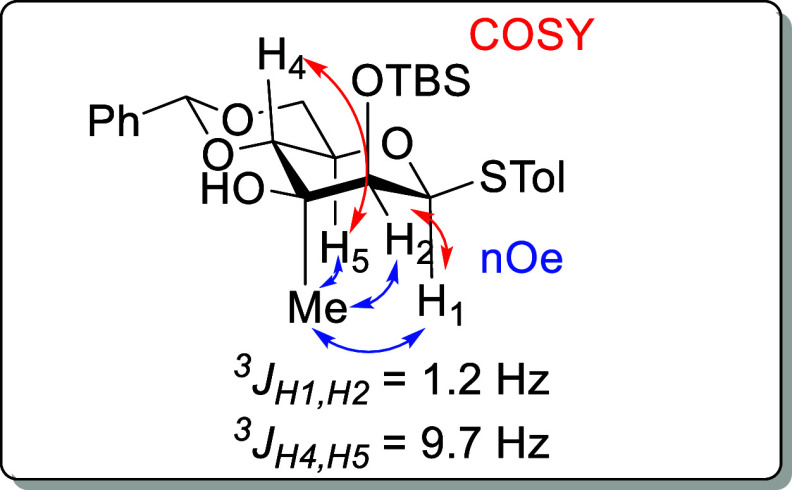
Diagnostic NOE correlations and coupling constants exemplified in **35**.

Ultimately, for comparison purposes, we required the α-configured donors and developed two routes for their preparation by inversion of the configuration of β-thiomannosides (Scheme 3). In the first route, hydrolysis of thioglycoside **35** with NBS in aqueous acetone and subsequent removal of the silyl group with TBAF gave hemiacetal **42** in 72% yield. This hemiacetal was then subject to the Shoda conditions for thioglycoside formation,^48^ stirring with toluenethiol, 2-chlorodimethylimidazolinium chloride, and triethylamine at 0 °C for 3 h when **43** was obtained in 83% yield. Regioselective monobenzylation with benzyl bromide and TBAB in a biphasic mixture of aqueous sodium hydroxide and dichloromethane then afforded **44** in a 78% yield. Esterification with ^13^C-labeled benzoic acid with carbonyl diimidazolide and DBU in THF at reflux then gave the ester ^**13**^ **C**-**45** in 93% yield. In a second approach, the diastereomeric ester ^13^C-**38** was activated with diphenyl sulfoxide and triflic anhydride in dichloromethane at −60 °C before the addition of toluenethiol, resulting in the direct formation of ^**13**^ **C-45** in 74% yield as a single diastereomer. *m*CPBA oxidation of ^**13**^ **C-45** at −78 °C then gave the corresponding sulfoxide ^**13**^ **C-46** in 90% yield as a single diastereomer assigned the (*R*)_S_ configuration by analogy.^46,47^

**Scheme 3:**
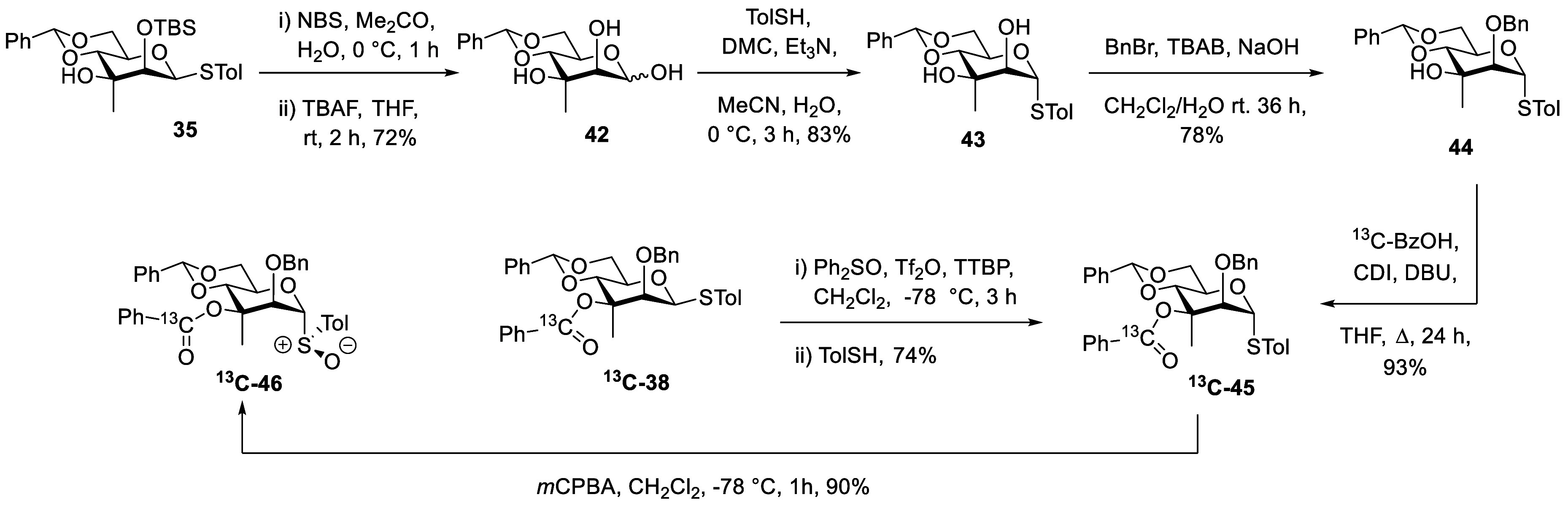
Synthesis of the Axial Glycosyl Sulfoxide ^**13**^ **C-46**

Finally, for comparison purposes, the mannopyranosyl α-sulfoxides **49** and ^**13**^ **C**-**49**, lacking the *C*-methyl group at the 3-position, were prepared by standard means from thioglycoside **47**, via **48** and ^**13**^ **C-48** (Scheme 4). Consistent with expectation, **49** and ^**13**^ **C-49** were obtained as single diastereomers, which were again assigned the (*R*)_S_ configuration by analogy.^46,47^

**Scheme 4:**
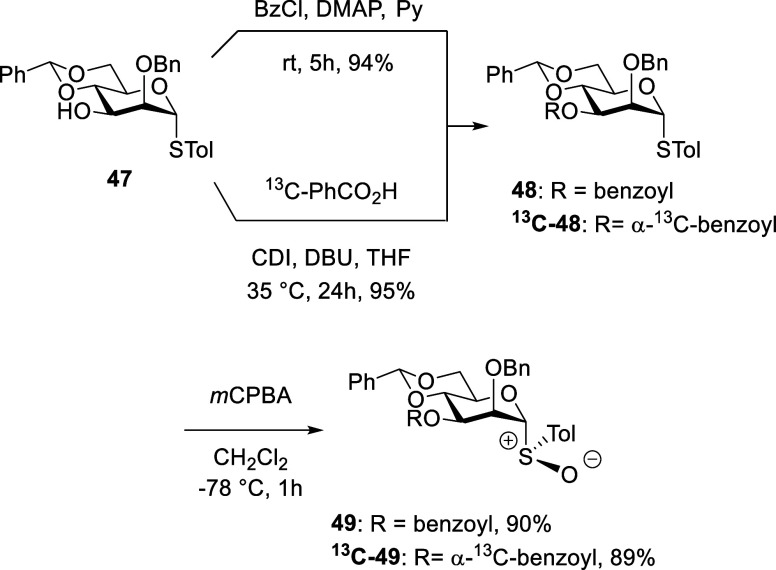
Synthesis of Mannopyranosyl Sulfoxides **49** and ^**13**^ **C-49**

### Ester Conformation

Turning to the conformation of the 3-*O*-acyl groups, esterification at O3 in both the α- and β-series resulted in a downfield shift of the H2 resonance of >1 ppm compared to the corresponding alcohols in the 3-*C*-methyl series of compounds, which was accentuated on oxidation of the thioglycosides to the corresponding sulfoxides (Table 1). Esterification of the desmethyl α-thioglycoside **47** on the other hand resulted in a smaller but still significant downfield H2 shift of ∼0.3 ppm (Table 1, entry 10), which was also accentuated on oxidation to the sulfoxide (Table 1, entry 11).

**Table 1:**
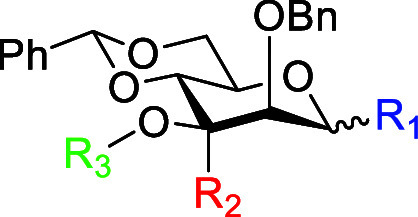
Influence of 3-*C*-Methylation on H2 Chemical Shifts in the 3-*O*-Acyl Donors

entry	cmpd	R_1_	R_2_	R_3_	δ H2
1	**37**	β-STol	Me	H	3.80
2	^**13**^ **C-38**	β-STol	Me	α-^13^C-Bz	5.05
3	**39**	β-STol	Me	*p*-NBz	5.00
4	^**13**^ **C-40**	β-S(O)Tol	Me	α-^13^C-Bz	5.33
5	**41**	β-S(O)Tol	Me	*p*-NBz	5.32
6	**44**	α-STol	Me	H	3.74
7	^**13**^ **C-45**	α-STol	Me	α-^13^C-Bz	4.95
8	^**13**^ **C-46**	α-S(O)Tol	Me	α-^13^C-Bz	5.36
9	**47**	α-STol	H	H	4.10
10	^**13**^ **C-48**	α-STol	H	α-^13^C-Bz	4.39
11	^**13**^ **C-49**	α-S(O)Tol	H	α-^13^C-Bz	4.65

We attribute the large downfield shift of H2 on esterification at O3 in the 3-*C*-methyl series to intramolecular C–H–O hydrogen bonding between H2 and the ester carbonyl group as a result of an increased population of an ester conformation in which the carbonyl group is oriented toward the C2–H bond (Figure 6) owing to destabilization of the normal ground-state conformation (Figure 3) by introduction of the axial group.

**Figure 6:**
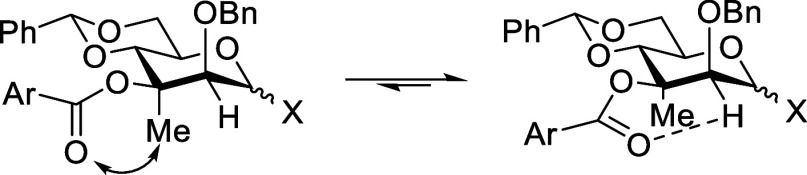
C–H–O hydrogen bonding in tertiary esters **38–41** and **45–46**.

### Effect of 3-*C*-Methylation on Glycosylation Stereoselectivity of 3-*O*-Acyl Mannopyranosyl Donors

To determine the influence of the 3-*C*-methyl group on glycosylation selectivity, we conducted a brief series of coupling reactions under a standard set of conditions, preactivating the methylated donors **40** and **41** and the methyl-free donor **49** with trifluoromethanesulfonic anhydride in the presence of the hindered non-nucleophilic base 2,4,6-tri-*tert*-butylpyrimidine (TTBP) at −60 °C in dichloromethane. The representative glycosyl acceptors, 1,2;3,4-di-*O*-isopropylidene-α-d-galactopyranose **50** and 1,2;5,6-di-*O*-isopropylidene-α-d-glucofuranose **51**, were then added and the reaction mixture stirred for 3 h at the same temperature before quenching by addition of triethylamine and finally warming to room temperature and workup (Table 2).

**Table 2:**
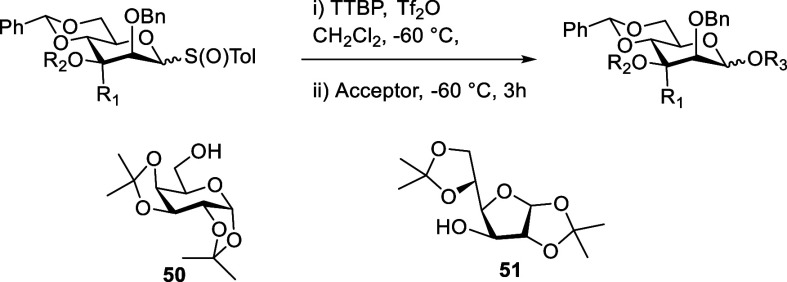

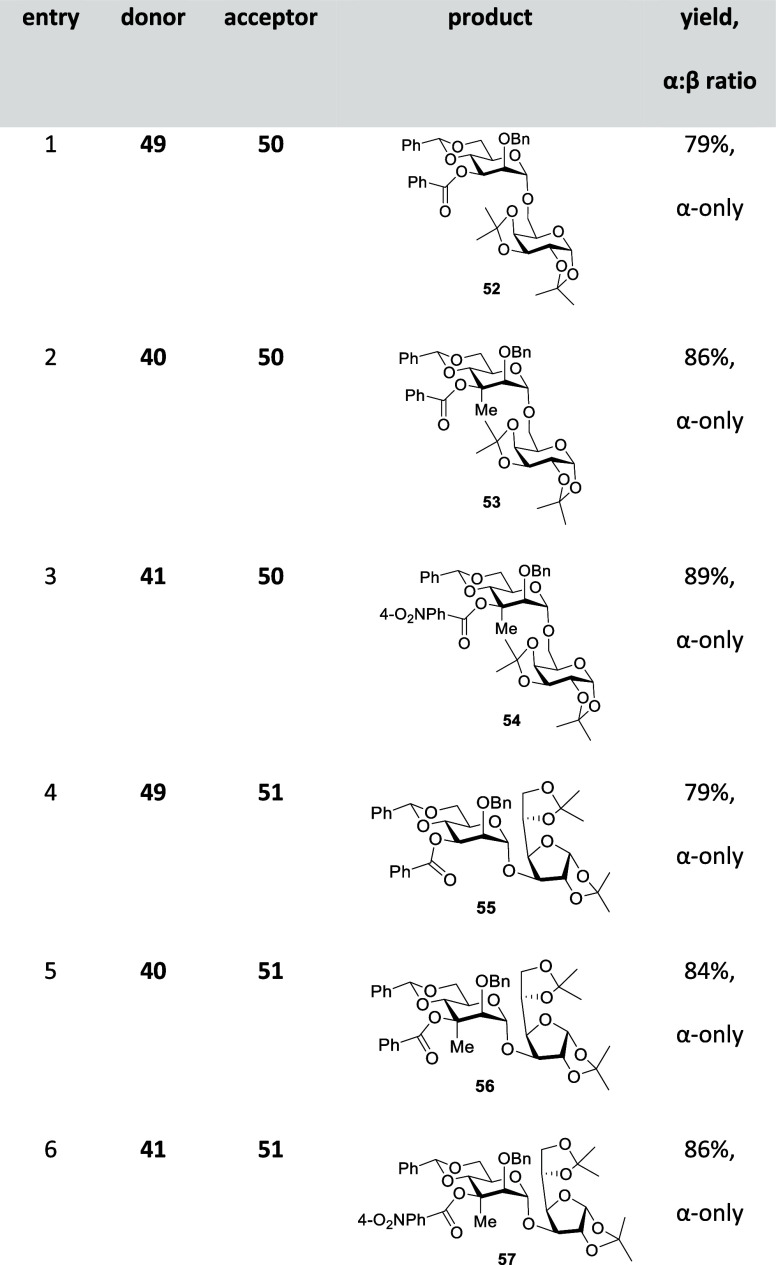
Glycosylation Reactions

All reactions were very highly α-selective, with only the one anomer being detected in the ^1^H NMR spectrum of the crude reaction mixtures and the products being isolated in high yield. The observation of high α-selectivity with the desmethyl donor **49** (Table 2 entries 1 and 4) is fully consistent with our original work, whatever the nature of the ester at the 3-position, benzoate,^10^ chloroacetate,^11^ or acetate,^49^ and that of subsequent workers, with the exception of Alex and Demchenko, who observed α-selectivity with a 3-*O*-benzoyl-protected donor but β-selectivity with the corresponding 3-*O*-picolyl donor.^50,51^ The very high α-selectivity observed with the 3-*C*-methyl-3-*O*-benzoyl-protected donor **40** (Table 2 entries 2 and 5) indicates that the presence of the axial methyl group (i) does not change the outcome of the reaction, (ii) likely does not impact the mechanism of α-direction by the ester group, and (iii) does not give rise to prohibitive 1,3-diaxial interactions at the transition state for α-glycoside formation. The excellent α-selectivity observed with the 3-*C*-methyl-3-*O*-*p*-nitrobenzoyl-protected donor **41** (Table 2 entries 3 and 6) reinforces these conclusions and points to the insensitivity of the directing effect to electron density on the ester, already apparent in our previous studies.^11,49^

Finally, we studied the coupling of thioglycoside **48** and acceptor **50** in dichloromethane at −60 °C activating with triflic anhydride in the presence of 1, 3, and 10 mol equiv of the additive (and copromotor) diphenyl sulfoxide (Table 3).^36–43^ With 1 and 3 mol equiv, crude reaction mixtures were clean and displayed α-glycoside **52** as the only glycoside formed, which was substantiated by its isolation in high yield in both cases (Table 3, entries 1 and 2). With 10 mol equiv, the crude reaction mixture was more complex but still showed no indication of the formation of a β-mannoside, with the α-glycoside **50** ultimately isolated in 37% yield (Table 3, entry 3).

**Table 3:**
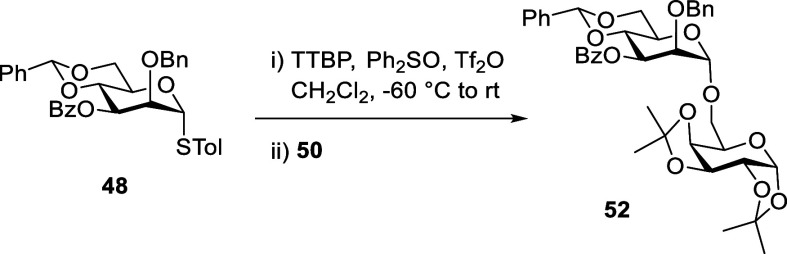
Influence of Diphenyl Sulfoxide on 3-*O*-Acyl-Directed Mannosylation

entry	Ph_2_SO (equiv)	yield (%)	α/β ratio
1	1	64	α-only
2	3	93	α-only
3	10	37	α-only

### Variable Temperature Studies of Activated Donors

In a first experiment, we studied the activation of the 3-*O*-benzoyl-α-mannosyl triflate ^**13**^ **C-49**, by addition of triflic anhydride to a solution of the donor and TTBP in CD_2_Cl_2_ at −78 °C followed by immediate return of the tube to the precooled probe of a 500 MHz NMR spectrometer. The initial ^1^H spectrum recorded at −78 °C, although somewhat broad, shows full and clean conversion to one major species. On warming to −70 °C and thereafter to 10 °C increments, the spectrum gradually sharpens until it is fully resolved around −40 °C (Figure 7), allowing full assignment of all ring protons. The broad singlet for the anomeric proton at δ_H_ 6.13 ppm and the broad multiplet for H5 at δ_H_ 4.14 ppm are consistent with this very major intermediate being covalent α-triflate ^**13**^ **C-58**. This assignment is further supported by a HSQC correlation of H1 with the anomeric ^13^C signal at δ_C_ 105 ppm and a ^1^ *J*_H1,C1_ coupling constant of 185 Hz.^22^ Yet further evidence is provided by the immediate formation at −78 °C of a very major peak at δ_F_ −75.8 ppm in the ^19^F NMR spectrum. Continued warming shows this α-mannosyl triflate to be stable until approximately −10 °C, above which decomposition sets in and is complete by +10 °C (Supporting Information). Critically, the ^13^C NMR spectrum of this reaction mixture is dominated at all temperatures by the ^13^C-enriched benzoate carbonyl group: no indication of the formation of the bridged dioxenium ion **18** was observed at any temperature (Figure 7) consistent with the CEST NMR results of Boltje and co-workers.^21,22^

**Figure 7:**
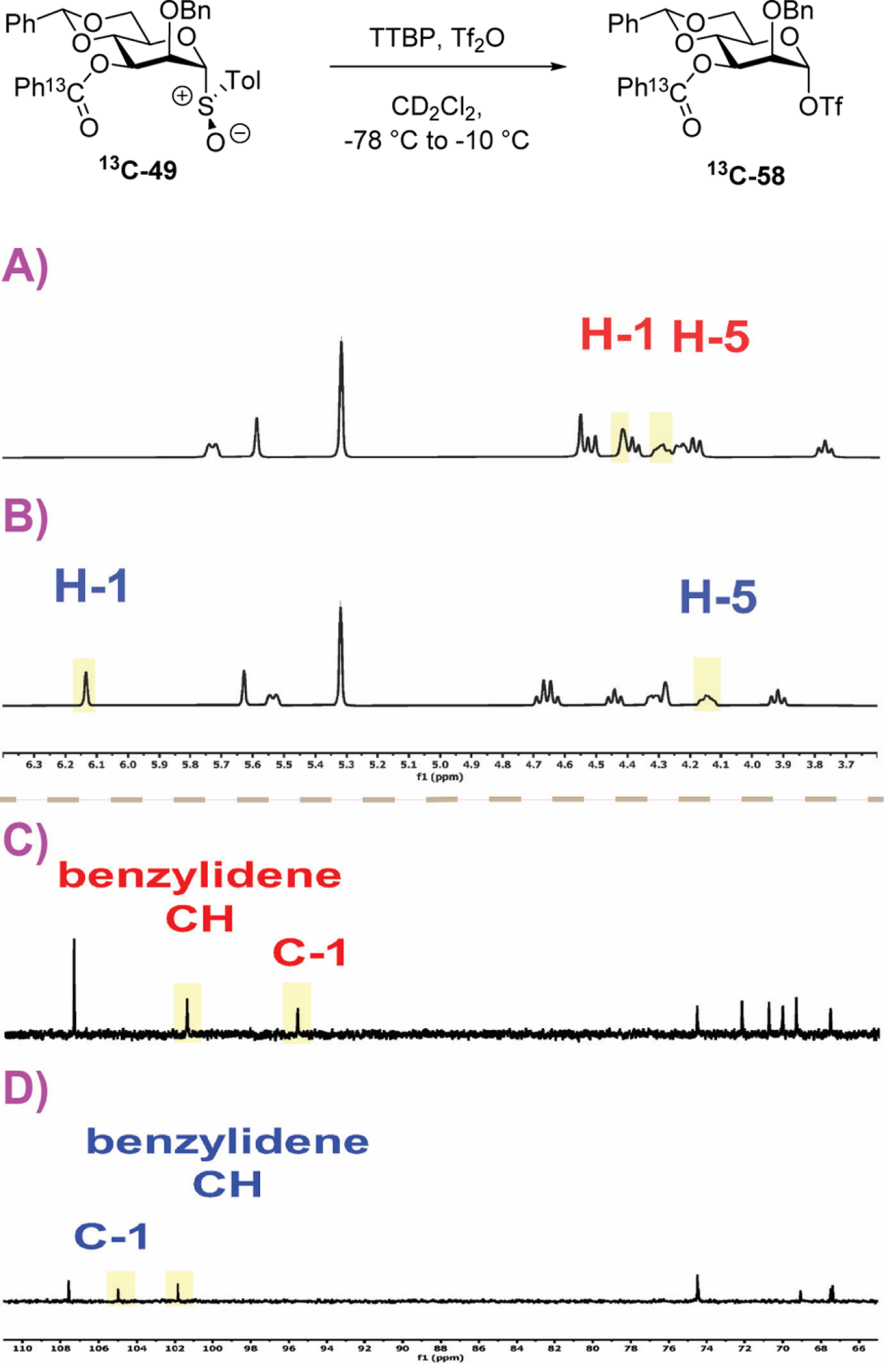
Partial ^1^H and ^13^C NMR spectra of the sulfoxide ^**13**^ **C-49** at −78 °C (A,C) and the reaction mixture at −40 °C following activation at −78 °C (B,D); structure of the α-triflate ^**13**^ **C-58**; see Supporting Information for complete spectra.

When the experiment was repeated with the major, less-polar diastereoisomer of 3-*C*-methyl-β-mannosyl sulfoxide ^**13**^ **C-40**, far more complex spectra were obtained following initial activation at −78 °C (Figure 8). Nevertheless, the observation of broad singlets at δ_H_ 6.24 and δ_F_ −75.9 and a weak peak at δ_C_ 104.4 supported the formation of α-mannosyl triflate ^**13**^ **C-59** among multiple other species. The complexity of the reaction mixture was evident from the ^13^C NMR spectrum, which showed a very poor signal-to-noise ratio, with the exception of the ^13^C-enriched benzoyl carbon. Additionally, this latter signal was split into multiple poorly resolved peaks, reflecting the presence of multiple ester-containing species. A minor peak at δ_C_ 175.5 possibly corresponds to a bridged dioxenium ion ^**13**^ **C-60**, but this signal is very weak in comparison to the total intensity of the carbonyl envelope around δ_C_ 167 and has comparable intensity to the various non-^13^C-enriched aromatic carbon signals in the same region of the spectrum, suggesting that it corresponds to no more than a few percent of the total reaction mixture.^52^ On warming to −70 °C and then higher in 10 °C increments, the ^1^H spectrum gradually sharpens such that from −40 to −30 °C, one major product is visible that is characterized by a broad singlet at δ_H_ 4.91 assigned to H2, in which the aforementioned CH–O hydrogen bonding is still present. In contrast to the general sharpening of the ^1^H NMR spectrum, as the temperature is raised from −78 to −30 °C, the ^1^H and ^19^F resonances ascribed to the α-mannosyl triflate ^**13**^ **C-59** at δ_H_ 6.24 and δ_F_ −75.3 broaden considerably and disappear by −30 °C (see Supporting Information). As the signal for the triflate anion at δ_F_ 78.9 broadens correspondingly over the same −78 to −30 °C temperature range, the implication is that the anomeric triflate is in dynamic exchange with free triflate and that the axial methyl group in ^**13**^ **C-59** considerably destabilizes the anomeric α-triflate ^**13**^ **C-59** with respect to its desmethyl counterpart ^**13**^ **C-58** because of the approximate alignment of the ester carbonyl dipole with the anomeric C–O bond dipole as we have previously suggested.^18^ Comparable results were obtained, under identical VT conditions, with the corresponding 3-*C*-methyl-α-mannosyl sulfoxide ^**13**^ **C-46** (see Supporting Information). In particular, the spectra arising from the activation of both the major and minor isomers of the β-sulfoxide ^**13**^ **C-40** and their α-anomer ^**13**^ **C-46** at −30 °C were grossly similar (see Supporting Information) indicative of the observation of species arising from cleavage of the initial anomeric *C*-sulfoxide bond rather than of any triflated sulfoxides such as we have recently seen in studies of the activation 5-thioglucopyranosyl sulfoxides.^53^ As a further check on this, a sample of the major, less polar isomer of ^**13**^ **C-40** was preactivated at −78 °C in the standard manner, warmed to −70 °C then allowed to rapidly attain −30 °C and spectra recorded before it was immediately recooled to −70 °C. The spectra recorded after recooling to −70 °C were grossly the same as those recorded at that temperature initially, with the exception of the incursion of a ^13^C signal at δ_C_ 181.8, to which we return below, further indicating full activation of the initial sulfoxide with cleavage of the anomeric C–S bond following activation (see Supporting Information).

**Figure 8:**
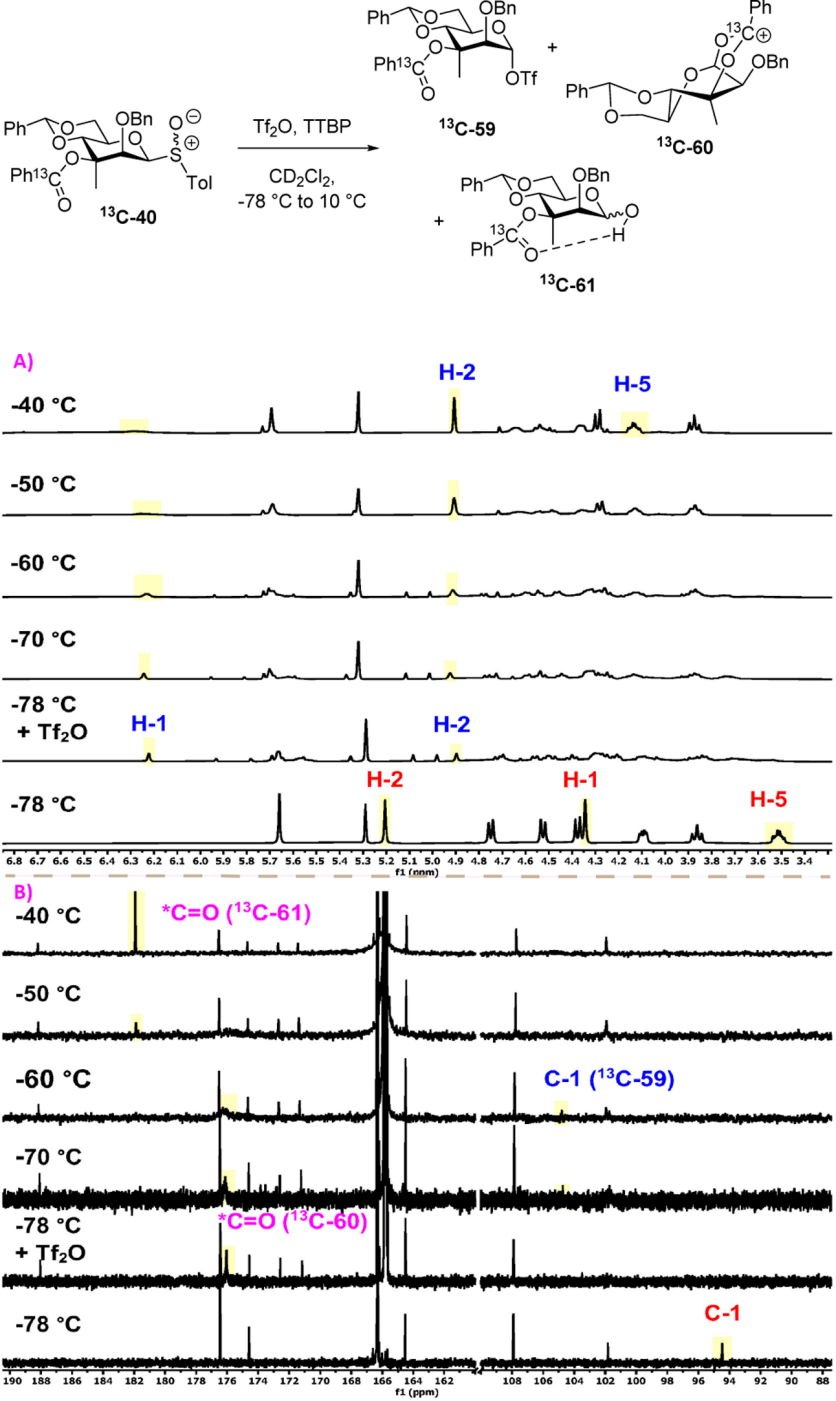
Partial ^1^H (A) and ^13^C (B) NMR spectra of the sulfoxide ^**13**^ **C-40** at −78 °C and the reaction mixture following activation at −78 and in 10 °C increments to −40 °C; structure of the α-triflate ^**13**^ **C-59**, bridged dioxenium ion ^**13**^ **C-60,** and hemiacetal ^**13**^ **C-61**. For full spectra see Supporting Information.

Some experiments, most prominently the activation of the major, less polar isomer of ^**13**^ **C-40** (Figure 8) display a relatively intense ^13^C-enriched signal at δ_C_ 181.8 at temperatures between −40 and −30 °C. Because of its sporadic nature, we believe this signal to be artifactual and tentatively assign it to the hemiacetal ^**13**^ **C-61**, which arises from the introduction and reaction of adventitious water, with the downfield shift of the carbonyl carbon arising from intramolecular hydrogen bonding (Figure 8). Importantly, HMBC spectra consistently failed to detect correlation of the signal at δ_C_ 181.8 with any anomeric proton, thereby ruling out a bridged ion structure for this species and dissuading us from further attempts at assignment.^54^ Finally, when the activation of ^**13**^ **C-40** was repeated in the absence of TTBP but under otherwise identical conditions, apart from the absence of signals due to TTBP and its conjugate acid, no significant differences were seen in the spectra (see Supporting Information), indicating that buffering of the reaction mixture by TTBP does not impact the overall outcome.

Next, we turned our attention to the activation of the desmethyl thioglycoside ^**13**^ **C-48** with the combination of diphenyl sulfoxide and triflic anhydride corresponding to the glycosylations reported in Table 3. In these VT NMR experiments, an excess of diphenyl sulfoxide was employed to ensure the formation of the glycosyl oxysulfonium ions in preference to the less stable glycosyl triflates as demonstrated previously by the Gin and Codée laboratories.^36–43^ Thus, addition of triflic anhydride (1.4 equiv) to a preformed mixture of ^**13**^ **C-48** (1 equiv), diphenyl sulfoxide (3 equiv), and TTBP (3 equiv) at −60 °C resulted in immediate formation of a single mannosyl intermediate ^**13**^ **C-62**. With the help of DEPT, COSY, HSQC, and HMBC spectra, the ^1^H and ^13^C resonances of the mannosyl skeleton were fully assigned and the structure ascribed to the α-diphenyl oxysulfonium species ^**13**^ **C-62** (Figure 9). The assignments of the broad singlet at δ_H_ 6.70 and the singlet at δ_C_ 106.4 to the anomeric proton and carbon resonances, respectively, are supported by a HSQC correlation, while their ^1^ *J*_H1,C1_ coupling constant of 184.4 Hz indicates the α-configuration. The presence of a single peak at δ_F_ −78.9 ppm in the ^19^F NMR spectrum, attributed to the triflate ion, after activation at −60 °C confirms the absence of a covalently bonded triflate under these conditions (see Supporting Information).

**Figure 9:**
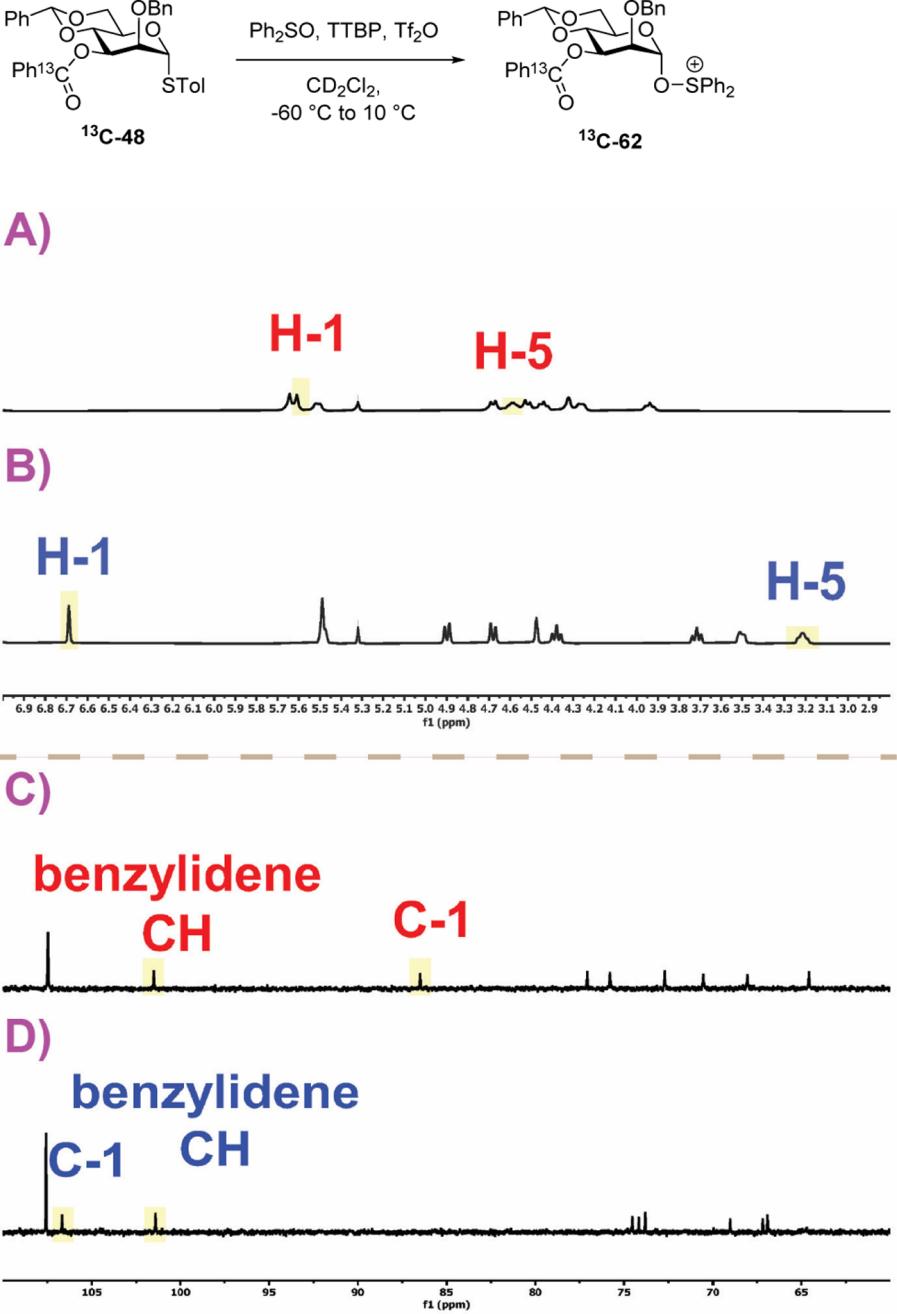
Partial ^1^H and ^13^C NMR spectra of the sulfoxide ^**13**^ **C-48** at −60 °C (A,C) and at −40 °C following activation at −60 °C (B,D); structure of the α-oxysulfonium ^**13**^ **C-62**. For full spectra see Supporting Information.

On gradual warming in 10 °C increments, ^**13**^ **C-62** is stable until −40 °C, with minor decomposition observed starting from −30 °C. Nevertheless, continued warming revealed this α-mannosyl oxysulfonium intermediate to persist even at room temperature (see Supporting Information). Interestingly, the mannose H5 signal at δ_H_ 3.16 of ^**13**^ **C-62** is unusually upfield for a 4,6-*O*-benzylidene-protected α-mannosyl derivative but is consistent with the comparable upfield H5 signal reported following activation under comparable conditions of a cognate bicyclic mannopyranosyl donor, phenyl 4,8-anhydro-2,3-di-*O*-benzyl-6,7-dideoxy-8-phenyl-d-*glycero*-α-d-*manno*-thiooctopyranoside.^55^ We ascribe this unusual H5 chemical shift to shielding by one of the phenyl groups in the oxysulfonium moiety. Neither the ^1^H nor the ^13^C NMR spectra supported the formation of bridged ion ^**13**^ **C-18** under these conditions.

Turning to the 3-*C*-methyl series, β-thiotolyl mannoside ^**13**^ **C-38** was activated at −60 °C under identical conditions to the desmethyl congener ^**13**^ **C-48**, resulting in the formation of a major and a minor species at −60 °C in a 10:1 ratio (Figure 10). Comparable spectra were obtained when the experiment was repeated with the α-anomer ^**13**^ **C-45** (Supporting Information). For the major species, the broad singlet at δ_H_ 6.75 and its correlation with anomeric carbon at δ_C_ 105.9 lead to its attribution as the α-mannosyl oxysulfonium intermediate ^**13**^ **C-63**, with further supported gleaned from the ^1^ *J*_H1,C1_ coupling constant of 184.6 Hz. An upfield H5 signal in this intermediate δ 3.06 is again attributed to shielding by one of the two phenyl groups in the pendant oxysulfonium moiety. The minor species, characterized by a broad singlet for the anomeric proton at δ_H_ 6.08, which is correlated by HSQC to the anomeric carbon signal at δ_C_ 98.1, is assigned as the β-mannosyl oxysulfonium intermediate ^**13**^ **C-64**. Unfortunately, due to the lower intensity of the anomeric carbon resonance of the minor species, the ^1^ *J*_H1,C1_ coupling constant could not be measured. The H5 resonance of this β-configured mannosyl oxysulfonium resonates at δ 3.40, a typical value for 4,6-*O*-benzylidene-protected β-mannopyranosyl derivatives. Warming the reaction mixture in 10 °C increments from −60 to 10 °C resulted in a change in the ratio of the two oxysulfonium species, with the intensity of the β-isomer increasing with temperature (see Supporting Information). No evidence was found on activation of either anomer for the formation of the bridged bicyclic ion ^**13**^ **C-60** under these conditions, but the tentatively assigned hemiacetal ^**13**^ **C-61** (δ_C_ 181.8) was formed to a minor extent, suggesting inadvertent hydrolysis during these reactions.

**Figure 10:**
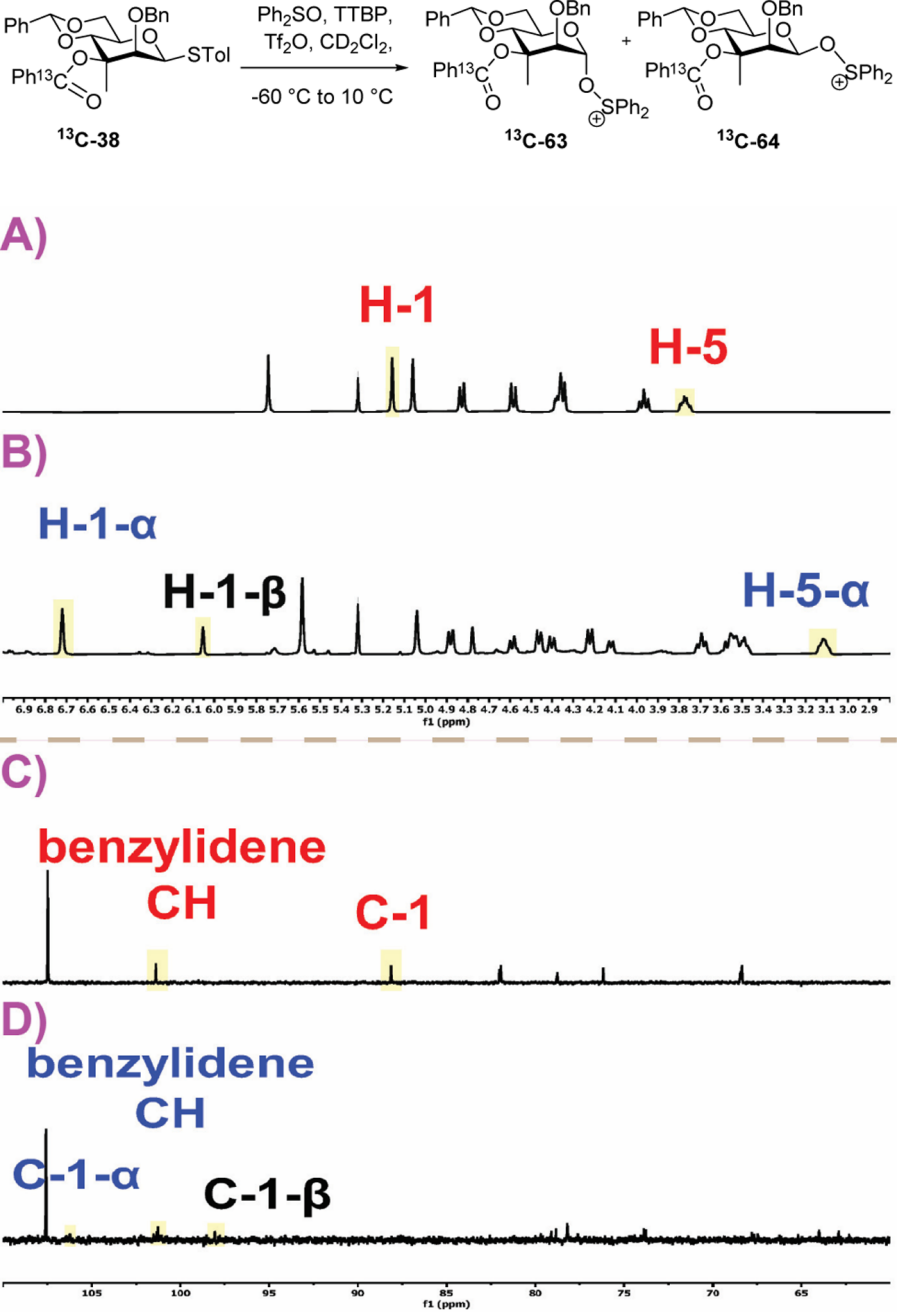
Partial ^1^H and ^13^C NMR spectra of the thioglycosides ^**13**^ **C-38** and ^**13**^ **C-45** at −60 °C (A,C) and the reaction mixture at −40 °C following activation at −60 °C (B,D); structures of the α- and β-oxysulfonium ions ^**13**^ **C-63** and ^**13**^ **C-64**. For full spectra see Supporting Information.

## Discussion

A 3-*C*-methyl-3-*O*-benzoyl-4,6-*O*-benzylidene-protected system of mannopyranosyl donors was designed and prepared with the intention of perturbing the ground-state conformation of the ester and enhancing DGP. In a series of glycosylation reactions conducted by both the glycosyl sulfoxide/triflic anhydride and the thioglycoside/diphenyl sulfoxide/triflic anhydride methods, these donors were shown to be extremely α-selective, consistent with the original observations in the desmethyl series: the presence of the additional methyl group does not impact the reactivity of the donors in a meaningful way.

In VT-NMR experiments with the simple ^13^C-enriched 3-*O*-benzoyl mannopyranosyl donor ^**13**^ **C-49**, we were unable to detect a signal corresponding to the hypothetical bridged dioxenium ion **18** on activation with triflic anhydride. This is consistent with the failure of Boltje and co-workers to detect **18** by the highly sensitive CEST NMR technique, which they initially attributed to this dioxenium ion either having a population below the 1% detection limit of their CEST technique or to its “exceedingly fast consumption”, which prevents its detection by the CEST technique.^22^ Subsequently, however, Boltje and Codée focus on the destabilizing conformational restraint imposed by the benzylidene ring as the reason underlying their failure to detect the bridged dioxenium ion **18**.^26^ For our part, we place an upper-limit of <1% on the population of **18** compared to the α-mannosyl triflate **58** in the same experiment as the intensity of the putative dioxenium C, 99%-enriched in ^13^C, is substantially less than the intensity of the nonenriched carbons in the major triflate **58** in the same experiment. Parallel experiments with both anomers of the 3-*C*-methyl donors ^**13**^ **C-40** and ^**13**^ **C-46** were complex, as evidenced by the broad 500 MHz ^1^H NMR spectra below −40 °C and the splitting of the ^13^C-enriched carbonyl carbon envelope in the 126 MHz ^13^C NMR spectra into multiple resonances. We attribute this broadening to the destabilization of the α-triflate by its 1,3-diaxial interaction with the methyl group and the presence of multiple slowly equilibrating conformations on the ^1^H NMR time scale. In spite of this complexity, a minor signal at δ_C_ 175.5 was observed in the ^13^C NMR spectra at the lower temperatures employed, consistent with the presence of bridged ion ^**13**^ **C-60** arising from DGP and the CEST NMR work of Boltje and co-workers with detection of the bridged ions **16** and **17**.^21,22^ The intensity of this signal, however, is only ∼4% of the intensity of the ^13^C-enriched carbonyl carbon envelope, indicating it to be far less stable than mannosyl triflate ^**13**^ **C-59**. In the temperature window between −40 and −30 °C when the ^1^H NMR spectra are sharper, this signal persists but with a reduced intensity of <1% of that of the enriched carbonyl carbon envelope. Thus, while the inclusion of the methyl group does enhance the formation of a bridged ion **60** over and above the standard desmethyl series, consistent with the design hypothesis and with our earlier observations on DGP from the 4-position of galactopyranosyl donor (Figure 4),^33^ the population of this ion remains minimal and much less than seen in the earlier galactopyranosyl series.

In the VT-NMR experiments conducted with the thioglycoside ^**13**^ **C-48** and the 3-*C*-analogs ^**13**^ **C-38** and ^**13**^ **C-45** through triflic anhydride preactivation in the presence of Ph_2_SO and TTBP, sharp ^1^H NMR spectra were observed even at −60 °C, but no evidence was found for the presence of a bridged ion ^**13**^ **C-60** even in the presence of the axial methyl group. Rather, clean formation of the α-mannosyl oxysulfonium ions **^13^C-62** and **^13^C-63** were seen at low temperature and in the case of the 3-*C*-methyl donors a less populated but substantial amount of the corresponding β-anomer ^**13**^ **C-64**. Importantly, consistent with the work of Gin,^38^ the inclusion of excess diphenyl sulfoxide in the reaction mixture completely replaces any mannosyl triflates by the corresponding mannosyl oxysulfonium ions. The maximum population of the bridged ion ^**13**^ **C-60** formed in the course of activation by the diphenyl sulfoxide/triflic anhydride combination is estimated at <0.1% of that of the mannosyl oxysulfonium ions; i.e., the intensity of the ^13^C-enriched signal at δ_C_ 175.5 is not even visible in comparison to the nonenriched ^13^C NMR signals in the same region of the spectrum. Such low concentrations of the bridged ion ^**13**^ **C-60** relative to the mannosyl oxysulfonium ions ^**13**^ **C-63** and ^**13**^ **C-64** in the absence of any data on the relative rates of subsequent conversion of such bridged ions and of the covalent triflates or oxysulfonium ions to the final products argue against a Curtin–Hammett kinetic scheme for the formation of the α-mannopyranosides involving DGP.

Overall, the preponderance of the evidence including (i) the inability to observe bridged ions by all but the highly sensitive CEST NMR technique for standard 3-*O*-acyl-4,6-*O*-benzylidene-protected mannopyranosyl donors; (ii) the very low population of such bridged ions in the case of the participation enhancing 3-*C*-methyl groups presented here on activation of the glycosyl sulfoxides with triflic anhydride; (iii) the complete absence of such ions on activation of the corresponding thioglycosides with excess diphenyl sulfoxide and triflic anhydride; (iv) the extreme α-selectivity in glycosylation reactions conducted under parallel conditions including thioglycoside activation by diphenyl sulfoxide and triflic anhydride; (v) the failure to trap such bridged species using carbonate probes;^12^ and (vi) the failure of neighboring group participation-directed α-mannosylation through six-membered cyclic ions even in ideally predisposed systems (Scheme 1)^13^ argues against DGP as the underlying cause of highly selective α-mannosylation in such systems. It follows that an alternative mechanism exists for α-mannoside formation in the 3-*O*-acyl-4,6-*O*-benzylidene protected mannopyranosyl donors and that this alternative mechanism likely extends to the simple 3-*O*-acyl mannopyranosyl donors that lack the rigidifying benzylidene acetal. Alternatively stated, Ockham’s razor^56^ suggests that one mechanism should suffice for both the 4,6-*O*-benzylidene series and the simpler and more conformationally mobile monocyclic series and consequently that as DGP is not operative in the 4,6-*O*-benzylidene protected series there is no need to invoke it in the absence of the rigidifying acetal when mannosylation is already moderately α-selective even in the absence of a 3-*O*-acyl group.

Turning to the much reduced α-directing effect of the 3-*O*-acyl group in glucopyranosyl donors,^26^ it follows that if the α-directing effect of the 3-*O*-acyl group in the mannopyranosyl series is not due to DGP, the absence of such a directing effect in the glucopyranosyl series cannot be used to explain the differences in stereoselectivity between the 3-*O*-acyl gluco- and mannopyranosyl donors. Certainly, we agree with Boltje, Codée and co-workers that DGP is less favorable in the glucopyranosyl series than in the above studied mannopyranosides as it would place the 2-*O*-substituent in an unfavorable bowsprit position in the bridging ion **19** and the preceding transition state, but this cannot explain the difference between the two series if DGP is also insignificant in α-mannopyranoside formation.

As previously,^18^ we posit that the highly α-directing effect of the 3-*O*-acyl group in the mannopyranosides is better explained by acceptor–donor hydrogen bonding. The concept of acceptor–donor hydrogen bonding at the transition state of glycosylation reactions, long advocated by Whitfield,^57–62^ has gained impetus following the introduction of Demchenko’s notion of hydrogen bond mediated aglycone delivery (HAD)^63–66^ and Jacobsen’s bisthiourea catalysts for dual donor and acceptor activation,^67–70^ and is now commonplace.^71–76^ In 4,6-*O*-benzylidene-directed β-mannosylation, density functional theory calculations supporting ^13^C primary kinetic isotope effect studies of the mechanism indicate acceptor hydrogen bonding to O3 of the donor across the β-face of the activated donor (Figure 11a).^77^ Such an acceptor–donor hydrogen bond is obviously significantly weakened when the standard ether-type protecting group in the β-selective systems is replaced by an ester in the α-selective systems under discussion. Overall, the effect of replacing an ether-type protecting group at the 3-position of a mannosyl donor by an acyl group is 2-fold: it weakens any acceptor–donor hydrogen bonding on the β-face and so increases the activation barrier to β-glycoside formation while providing an ideally poised hydrogen bond acceptor in the form of the acyl group oriented toward the anomeric position on the α-face. Acceptor hydrogen bonding to this acyl group locates the nucleophilic oxygen ideally for α-facial attack on the minor β-configuration of the activated donor (Figure 11b). The observation of β-mannosyl triflates however is rare owing to their relative instability^6,27,78^ but that of β-mannosyl oxysulfonium ions, which benefit from greater stability but retain similarly high selectivity is demonstrated in the above VT NMR experiments. In their very detailed study of the formation and reactivity of 4,6-*O*-benzylidene-2,3-*O*-methyl protected mannopyranosyl donors, Asensio and co-workers compute a ratio 16523/1 in favor of the α- over the β-configured covalent triflate. Asensio and co-workers also find that equilibration between the two triflates is slow compared to subsequent displacement by the acceptor alcohols, causing them to argue against a Curtin–Hammett kinetic scheme for the formation of 2,3-di-*O*-methyl α-mannopyranosides.^78^ In contrast, in their 2024 study of the 3-*O*-acyl directing effect most pertinent to the current problem, Boltje and Codée and co-workers compute a 2.4 kcal mol^–1^ difference in free energy between the more stable α- and the less stable β-triflates of 3-*O*-benzoyl-4,6-*O*-ethylidene-2-*O*-methyl mannopyranose, indicative of an equilibrium ratio of <100:1.^26^ This reduction in free energy difference between the α- and β-mannosyl triflates on introduction of a 3-*O*-acyl group is consistent with our earlier suggestion that the 3-*O*-acyl group destabilizes the α-triflate because of the approximate alignment of the dipoles of the ester carbonyl group and the anomeric C–O bond.^18^ The lower mannosyl triflate anomeric ratio computed in the presence of the 3-*O*-acyl group compared to the 3-*O*-methyl group will result in a lower activation energy for their interconversion, whether by a dissociative mechanism as argued by Boltje and co-workers^22^ or an associative one as demonstrated in the 2,3-di-*O*-methyl series by Asensio and co-workers,^78^ and increase the likelihood of a Curtin–Hammett kinetic scheme involving the β-triflate as required for the mechanism proposed in Figure 11b.

**Figure 11:**
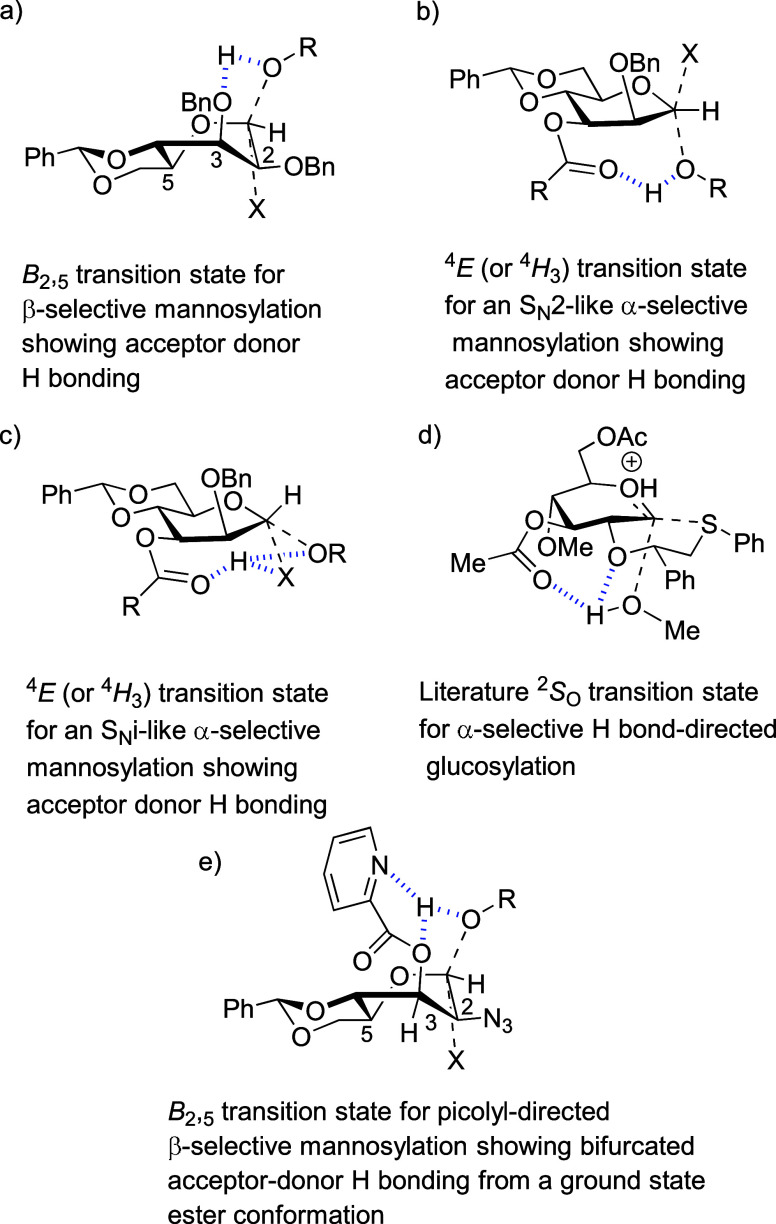
Acceptor–donor hydrogen bonding in the (a) 3-*O*-ether; (b) 3-*O*-acyl-4,6-*O*-benzylidene mannopyranosyl donor operating via an S_N_2-mechanism; and (c) 3-*O*-acyl-4,6-*O*-benzylidene mannopyranosyl donor operating via an S_N_i-mechanism. (d) Hydrogen-bonded transition state for 3-*O*-acyl-directed α-glucosylation. Adapted from Fang, T.; Gu, Y.; Huang, W.; Boons, G.-J. Mechanism of Glycosylation of Anomeric Sulfonium Ions. *J. Am. Chem. Soc.* **2016**, 138, 3002–3011. Copyright 2016 American Chemical Society. (e) Reinforcement of acceptor–donor hydrogen bonding in benzylidene-directed β-mannosylation by use of a 3-*O*-picolyl ester. X = TfO^–^, Ph_2_S^+^-O, etc.

Alternatively, consistent with recent proposals on the frontside (S_N_i) displacement of triflates by weak nucleophiles^79–81^ and the established operation of such mechanisms in retaining glycosyl transferases,^82–86^ it is possible that the incoming acceptor is involved in a bifurcated hydrogen bond with the ester carbonyl group and the departing triflate, or other leaving group, from the α-face of the molecule (Figure 11c). This scenario has the obvious advantage of not requiring population of the unstable β-triflate or other minor β-donor, so avoiding the need for any Curtin–Hammett kinetic scheme.

Support for the posited α-directing acceptor–donor hydrogen bonding in the 3-*O*-acyl system is drawn from the work of Boons and co-workers on the origin of the α-directing effect of a 3-*O*-acyl group in their chiral glycosyl sulfonium ion-mediated glycosylation method.^87,88^ In this computational study, a transition state for a loose S_N_2-like displacement by methanol was located with an approximate ^2^ *S*_O_ conformation of the pyranose ring in which the acceptor is held in place by hydrogen bonding to the carbonyl group of the 3-*O*-acyl group (Figure 11d).^89^ Moreover, it was stated that all attempts to locate a transition state for α-glycosylation lacking the hydrogen bond failed, pointing to the importance of this feature. The ^2^ *S*_O_-like transition-state conformation derived by Boons and co-workers (Figure 11d), which is imposed by the fused bicyclic nature of the system, reduces the O2–C2–C3–O3 torsion angle from the 60° in the initial ^4^ *C*_1_ glucopyranose conformation, and so creates space for the bifurcated acceptor–donor hydrogen bonding interaction indicated. Such a ^2^ *S*_O_ transition-state conformation is not feasible for donors restricted by the presence of a 4,6-*O*-benzylidene acetal, and any *B*_2,5_ or ^4^ *E* or related ^4^ *H*_3_ transition states such as depicted in Figure 11a,b for the mannopyranosyl series would open up the O2–C2–C3–O3 torsion angle in the corresponding glucopyranosides and so sterically hinder any donor–acceptor hydrogen bonding to the 3-*O*-acyl group on the α-face. This model therefore not only adequately explains the excellent α-selectivity observed with the 3-*O*-acyl-4,6-*O*-benzylidene-mannopyranosyl donors but also the absence of such a 3-*O*-acyl-directing effect in the corresponding glucopyranosyl donors discussed by Boltje, Codée, and co-workers.^26^

We now return to the work of Demchenko and co-workers on hydrogen bond-mediated aglycone delivery and specifically to their observation that an ethyl 2-azido-3-*O*-benzoyl-4,6-*O*-benzylidene-2-deoxy-α-d-thiomannopyranosyl donor was fully α-selective on activation with *N*-iodosuccinimide and triflic acid in dichloroethane at −30 °C whereas the corresponding 3-*O*-picolyl donor afforded the β-glycoside with 9:1 selectivity.^50^ The α-selectivity observed with the 3-*O*-benzoyl donor is fully consistent with our original observations and the work we report here and is best explained by the models of Figure 11b,c. Demchenko and co-workers explained the β-selectivity of the 3-*O*-picolyl donor as arising from acceptor hydrogen bonding to the picolyl nitrogen but with the ester group in the high energy syn-conformation.^90^ In the light of the above discussion, the β-selectivity observed with the picolyl ester is better explained (Figure 11e) by a *B*_2,5_ transition state, akin to that proposed (Figure 11a) for 4,6-*O*-benzylidene-directed mannosylation in general,^77,91^ in which the ester retains the ground-state conformation and the acceptor participates in a bifurcated hydrogen bond with O3 and the picolyl nitrogen. The picolyl ester therefore reinforces the natural β-directing effect of the benzylidene acetal by strengthening an already present hydrogen bond. Aside from the details of the transition state (Figure 11e), the main lesson to be taken from Demchenko’s observations on the changes in selectivity between the 3-*O*-benzoyl and the 3-*O*-picolyl esters is that the α-directing effect of the benzoate ester is overridden by a simple bifurcated hydrogen bond to the stronger hydrogen bond accepting picolyl nitrogen.

Finally, we return to the importance of ester conformation in DGP. As discussed above (Figure 3), it is well-established that the predominant conformation of carboxylate esters of secondary alcohols is one in which the carbonyl oxygen approximately eclipses the α–C–H bond of the alkoxy moiety,^28–30^ with the barrier to rotation about the C­(=O)–O–C­(H)-R_2_ bond calculated to be 4–12 kcal mol^–1^ (Figure 3).^31,32^ Indeed, Boltje and Codée in their own calculations find the standard ground-state ester conformation for esters at the 3-position of manno- and glucopyranosyl donors and calculate the barrier to rotation about the C3-OBz bond to be 8.3 and 9.6 kcal mol^–1^ in the gluco- and manno-series, respectively.^26^ However, as discussed by Anderson and co-workers,^30^ closer inspection of the crystallographic record reveals that the conformation is influenced by substitution at the two flanking carbons. For cyclic equatorial secondary alcohol-derived esters, Anderson and co-workers find a staggered conformation with an H–C–O–C­(=O) torsion angle of 30–40° to be most common and using molecular mechanics calculations estimate a barrier of only 1 kcal mol^–1^ for the equilibration of two such staggered conformers via the eclipsed conformation. Esters derived from cyclic equatorial secondary alcohols with one equatorial flanking substituent mostly adopted a staggered conformation with the carbonyl oxygen canted away from the flanking substituent by a torsion angle of 28°. Esters of equatorial secondary alcohols with an equatorial flanking substituent on either side on the other hand occupied a more constrained area of chemical space with H–C–O–C­(=O) torsion angles <20° of which more than half had a torsion angle <10°. Molecular mechanics calculations with the isomers of 2,6-dimethylcyclohexyl acetate were conducted and revealed the all-equatorial isomer, in which the ester group is flanked by two equatorial methyl groups, to preferentially adopt the eclipsed conformation (H–C–O–C­(=O) = 0°. On the other hand, in the isomer with one axial and one equatorial methyl group, the minimum energy conformation is a staggered one in which the carbonyl group is rotated toward the axial methyl group with a H–C–O–C­(=O) torsion angle of ∼40°.^30^ Extrapolating from these findings, it is reasonable to expect a 3-*O*-acyl group in a glucopyranosyl system, with flanking equatorial substituents at both the 2- and 4-positions, to take up a close to eclipsed conformation (Figure 12a), while in mannopyranosides with an axial substituent at the 2-position and an equatorial one at the 4-position, the preferred conformation will be staggered with the acyl group turned toward C2 (Figure 12b). Conversely in the galactopyranosyl series, with an axial substituent at the 4-position and an equatorial substituent at the 2-position, a staggered conformation will be adopted with the acyl group angled toward C4 (Figure 12c). The precise values of the H–C–O–C­(=O) torsion angles in each case can be expected to vary according to the size of the ester R group, the O2 and O4 protecting groups, P, and even the anomeric configuration, α- or β-. For the 3-*C*-methyl sugars studied here, the eclipsed conformation will be significantly destabilized, leading in the mannose case to a greater preference for the staggered conformation in which the acyl carbonyl group is oriented toward C2. The imposition of this conformation by the axial methyl group forces the carbonyl group into proximity of H2, resulting in the CH–O hydrogen bonding discussed above (Figure 6). The configuration-dependent conformations of acyl groups at the 3-position of glucose, mannose, and galactose derivatives are exemplified by the methyl peracetyl gluco-, manno-, and galactopyranosides **65**, **66**, and **67**.^92^ In the structure of the glucopyranoside **65**, the C2–C3–O3-carbonyl C torsion angle is 118.7°, meaning the carbonyl group almost perfectly eclipses the C3–H3 bond, whereas in the manno- and galactopyranosides **66** and **67**, the C2–C3–O3-carbonyl C torsion angle is 78.6° and 158.2°, respectively. Thus, in mannopyranoside **66**, the carbonyl group is rotated approximately 40° toward C2, whereas in galactopyranoside **67**, it is rotated by the same amount toward C4 (Figure 12).

**Figure 12:**
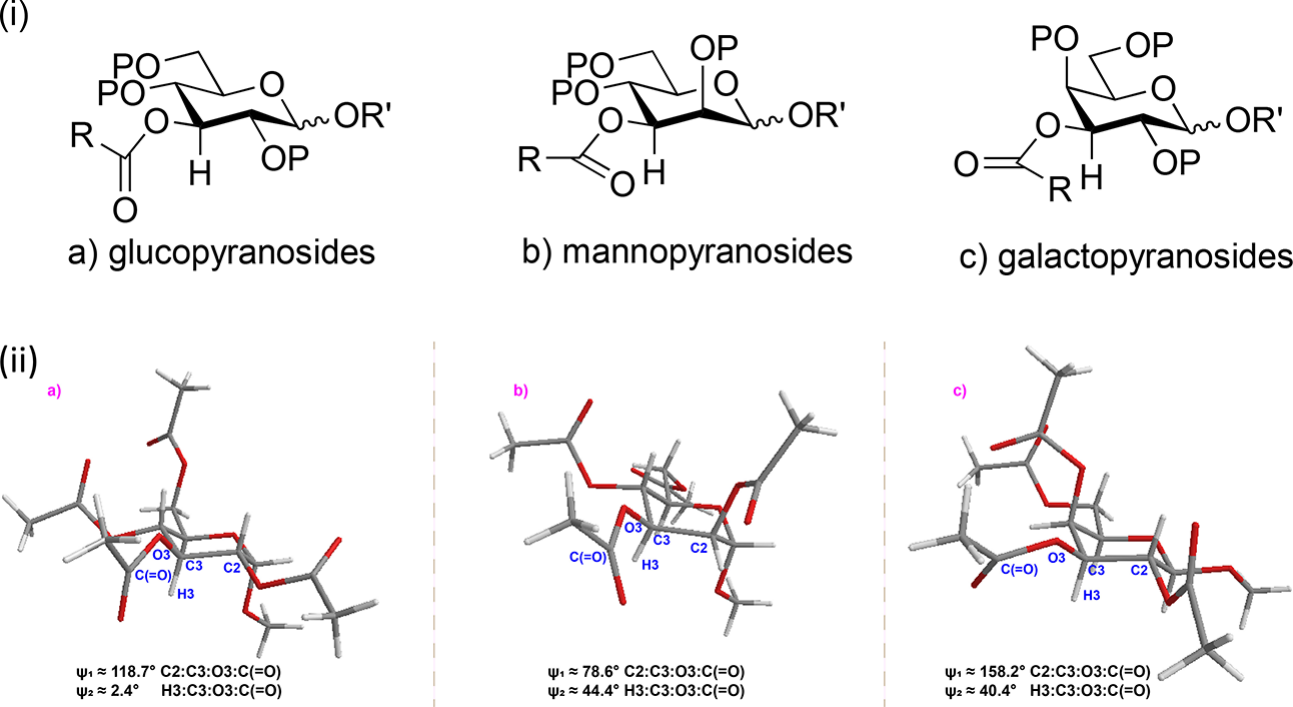
(i) Anticipated conformations of 3-*O*-acyl groups in (a) glucopyranosides, (b) mannopyranosides, and (c) galactopyranosides; (ii) X-ray crystal structures of (a) methyl α-d-glucopyranoside **65** (CCDC: WAYBIK), (b) methyl α-d-mannopyranoside **66** (CCDC: WAYBUW), and (c) methyl β-d-galactopyranoside **67** (CCDC: WAYBOQ).^92^

The canting of the carbonyl group toward C2 in the 3-*O*-acyl mannopyranosides by approximately 40° suggests that it is more favorably placed to accept a hydrogen bond from the incoming acceptor alcohol on the α-face of the molecule than is the same carbonyl group in the corresponding gluco- and galactopyranosyl donors. In other words, the high α-selectivity in the 3-*O*-acyl mannopyranosyl donors is further facilitated by the conformation of the ester, which in its ground state is ideally disposed to support donor–acceptor hydrogen bonding.

## Conclusions

On the basis of an extensive chemical and VT-NMR spectroscopic investigation, we conclude that the strong α-directing effect of a 3-*O*-acyl group in mannopyranosylation, that overcomes even the well-known β-directing influence of a 4,6-*O*-benzylidene acetal is unlikely to arise from DGP. Rather, we suggest that it arises from hydrogen bonding of the acceptor to the 3-*O*-acyl group at the transition state for glycosidic bond formation. This acceptor–donor hydrogen bonding is favored in the mannopyranose series by the staggered ground-state conformation of the 3-*O*-acyl group, which orients the carbonyl group toward C2 and the anomeric position and ideally positions it to take part in the postulated hydrogen bond. The much weaker α-directing effect of 3-*O*-acyl glucopyranosyl donors is due to the clash of the equatorial O2 substituent with any acceptor–donor hydrogen bond on the α-face and to the requirement for a nonground-state conformation of the glucosyl 3-*O*-acyl group.

## Supplementary Material



## Data Availability

The data underlying this study are available in the published article and its Supporting Information.

## Notes

Full experimental details, copies of the ^1^H, ^13^C, and ^19^F NMR spectra of all newly synthesized compounds, and stacked plots for the VT NMR data studies (PDF)
