# Supplementary material for: Mechanism of 3‑O‑Acyl-Directed α‑Mannopyranosylation and Rationalization of the Contrasting Behavior of 3‑O‑Acyl Glucopyranosyl Donors
Source: J Org Chem. 2025 Oct 6;90(41):14769–85. doi: 10.1021/acs.joc.5c01978 (PMC12538595; doi:10.1021/acs.joc.5c01978)

Supporting Information

for

Mechanism of 3-*O*-Acyl-Directed α-Mannopyranosylation and Rationalization of the Contrasting Behavior of 3-*O*-Acyl Glucopyranosyl Donors

Shuay Abdullayev,[a,b] and David Crich[a,b,c,*]

a) Department of Pharmaceutical and Biomedical Sciences, University of Georgia, 250 West Green Street, Athens, GA 30602, USA

b) Complex Carbohydrate Research Center, University of Georgia, 315 Riverbend Road, Athens, GA 30602, USA

c) Department of Chemistry, University of Georgia, 302 East Campus Road, Athens, GA 30602, USA

Email: David.Crich@uga.edu

Table of Contents

General Information S2

Experimental Procedures for the Preparation of Compounds S3

References S25

^{1}H, ^{13}C{^{1}H}, DEPT, COSY, HSQC, HMBC NMR Spectra of Compounds S26

Variable Temperature (VT) NMR Study S211

General Information

Material and Methods

All reactions in organic media were performed in standard oven dried glassware under an argon atmosphere. All reagents were used as supplied without prior purification unless otherwise stated. Solvents used for column chromatography were analytical grade and purchased from commercial suppliers. For all heated reactions an appropriately-sized, thermostatically-controlled heating block was used as heat source. Reactions were monitored by analytical thin-layer chromatography (TLC) using 250 µm glass backed silica (XHL) and compounds were visualized with a 254 nm UV lamp or ceric ammonium molybdate solution (100 mL H_2SO_4, 900 mL H_2O, 25 g $(NH_4)_6Mo_7O_{24}H_2O$, 10 g $Ce(SO_4)_2$), and subsequent spots developed by gentle warming with a heat-gun. Purifications were performed by flash column chromatography using a COMBIFLASH® NEXTGEN system with the indicated eluent. Specific rotations $[\alpha]_D$ were measured at 589 nm and 22±1 °C in $CHCl_3$ or CH_3OH as stated with a path length of 10 cm with a Rudolph Research Analytical AUTOPOL® III automatic polarimeter. Nuclear Magnetic Resonance (NMR) spectroscopy was used to record ^1H NMR and ^{13}C NMR spectra at 500 and 600 MHz and at 126 or 151 MHz, respectively, on JEOL, Varian and Bruker spectrometers. Proton and carbon chemical shifts (δ) are reported in ppm relative to the chemical shift of residual CD_2Cl_2 (in ^1H 5.32 ppm, in ^{13}C 53.84 ppm), $CDCl_3$ (in ^1H 7.26 ppm, in ^{13}C 77.16 ppm) and CD_3OD (in ^1H, 3.31 ppm and in ^{13}C, 49.0 ppm). Selective 1D Nuclear Overhauser Effect SpectroscopY (NOESY) and 2D homonuclear COrrelation SpectroscopY ^1H-^1H (COSY), ^1H-^{13}C Heteronuclear Single Quantum Coherence (HSQC), and Heteronuclear Multiple bond Correlation (HMBC) experiments were used to confirm NMR peak assignments. Coupling constants (*J*) are reported in Hertz (Hz), and the following abbreviations are used for peak multiplicities: singlet (s), broad singlet (br s), doublet (d), doublet of doublets (dd), doublet of doublets of doublets (ddd), triplet (t), doublet of triplets (dt), triplet of doublets (td), triplet of triplets (tt), multiplet (m). High-resolution electrospray ionization (ESI) mass spectrometry data were recorded using a Thermo Scientific Orbitrap mass analyzer.

Experimental Procedures for the Preparation of Compounds

General Procedure for Esterification (GP1):

Aromatic acid (522 mg, 4.27 mmol, 10 equiv) and 1,1'-carbonyl diimidazole (CDI, 693 mg, 4.27 mmol, 10 equiv) in THF (10 mL, 0.5 M) was stirred for 1 h at room temperature (or reflux temperature) under an argon atmosphere. To this reaction mixture was added a solution of alcohol **37**, **44**, or **47** (205 mg, 0.428 mmol, 1 equiv) in THF (8 mL, 0.055 M) followed by 1,8-diazabicyclo[5.4.0]undec-7-ene (DBU, 0.96 mL, 6.42 mmol, 15 equiv). After stirring for 12 h at 65 °C further DBU (0.96 mL, 6.42 mmol, 15 equiv) was added to the reaction mixture and stirring continued for another 12 h. The reaction mixture was diluted in EtOAc, washed with saturated aqueous $NaHCO_3$ and brine. The organic layer was dried over anhydrous Na_2SO_4 and concentrated *in vacuo*. The residue was purified by flash column chromatography by gradient elution of hexane: EtOAc (0% EtOAc in hexane to 20% EtOAc/hexane).

General Procedure for Sulfoxide Formation from Sulfide (GP2):

To a solution of sulfide **38**, **^{13}C-38**, **39**, **^{13}C-45**, **48**, or **^{13}C-48** (330 mg, 0.566 mmol, 1 equiv) in DCM (11.3 mL, 0.05 M) was added 3-chloroperbenzoic acid (*m*CPBA, 152 mg, 0.678 mmol, 1.2 equiv) in DCM (6.8 mL, 0.1 M) at -78 °C under an argon atmosphere. The reaction mixture was stirred for 1 h, then quenched by addition of saturated aqueous $NaHCO_3$. The organic layer was extracted with DCM and washed with saturated aqueous $NaHCO_3$, brine, dried over anhydrous Na_2SO_4 and concentrated *in vacuo*. The residue was purified by flash column chromatography by gradient elution of hexane: EtOAc (5% EtOAc in hexane to 100% EtOAc/hexane).

General Procedure for Glycosylation (Sulfoxide as the Donor) (GP3):

A mixture of sulfoxide donor **40**, **41**, or **49** (36 mg, 0.062 mmol, 1.65 equiv), 2,4,6-tri-*tert*-butylpyrimidine (TTBP, 32 mg, 0.126 mmol, 3.3 equiv) and 4Å MS (38 mg, 0.2 g per mmol) in anhydrous DCM (2.5 mL, 0.025 M) was stirred for 30 min. at -60 °C, under an argon atmosphere. After the addition of freshly distilled trifluoromethanesulfonic anhydride (Tf_2O, 11 µL, 0.065 mmol, 1.7 equiv) the resulting mixture was stirred for 10 minutes. Acceptor **50** or **51** (10 mg, 0.038 mmol, 1 equiv) in DCM (0.2 mL, 0.19 M) was added, and the reaction mixture was stirred for 3 h, before quenching by addition of Et_3N. The reaction mixture was allowed to reach room temperature, then filtered over a pad of Celite. The filtrate was washed with saturated aqueous $NaHCO_3$ and brine. The organic layer was dried over anhydrous Na_2SO_4 and concentrated *in vacuo*. The residue was purified by flash column chromatography by gradient elution of hexane: EtOAc (1% EtOAc in hexane to 30% EtOAc/hexane).

General Procedure for Glycosylation (Sulfide as the Donor) (GP4):

A mixture of sulfide donor **48** (50 mg, 0.088 mmol, 1 equiv), TTBP (67 mg, 0.264 mmol, 3 equiv), diphenyl sulfoxide (56 mg, 0.264 mmol, 3 equiv) and 4Å MS (88 mg, 1 g per mmol) in anhydrous DCM (4 mL, 0.022 M) was stirred for 30 min. at -60 °C, under an argon atmosphere. Then, to this mixture was added freshly distilled Tf_2O (21 µL, 0.065 mmol, 1.4 equiv) and the resulting mixture was stirred for 10 minutes. Acceptor **50** (35 mg, 0.132 mmol, 1.5 equiv) in DCM (0.75 mL, 0.18 M) was added, and the reaction mixture was stirred for 6 h. The reaction mixture was allowed to reach room temperature, then filtered over a pad of Celite. The filtrate was washed with aqueous solution of saturated aqueous $NaHCO_3$ and brine. The organic layer was dried over anhydrous Na_2SO_4 and concentrated *in vacuo*. The residue was purified by flash

column chromatography by gradient elution of hexane: EtOAc (1% EtOAc in hexane to 30% EtOAc/hexane).

General Procedure for Variable Temperature (VT) NMR Study (GP5):

Table S1. Reagents ratio for Variable Temperature NMR Study.

Entry	Donor (1.0 equiv)	TTBP (equiv)	Tf_2O (equiv)	Ph_2SO (equiv)	CD_2Cl_2	Starting T (°C)
1	Sulfoxide (21 mg, 0.035 mmol)	1.0	2.0	-	0.7 mL (0.05 M)	-78
2	Sulfoxide (42 mg, 0.07 mmol)	-	2.0	-	0.7 mL (0.1 M)	-78
3	Sulfide (31 mg, 0.053 mmol)	3.0	1.4	3.0	0.7 mL (0.075 M)	-60

A solution of sulfoxide **40**, **^{13}C-40**, **^{13}C-46**, or **^{13}C-49**, or sulfide **^{13}C-38**, **^{13}C-45**, or **^{13}C-48**, in CD_2Cl_2 containing TTBP (and Ph_2SO) was placed into an NMR tube and cooled to -78 °C in the NMR probe. After initial ^{1}H and ^{13}C spectra were recorded, the sample quickly removed from the probe and freshly distilled and precooled Tf_2O was added. After shaking, the sample was quickly returned to the NMR probe and once temperature stabilized, ^{1}H, ^{13}C and ^{19}F NMR were recorded. The temperature were increased 10 °C increments with new spectra recorded at each interval.

***p*-Methylphenyl 4,6-*O*-benzylidene-3-*O*-*p*-methoxybenzyl-2-*O*-*tert*-butyldimethylsilyl-thio-β-D-mannopyranoside 32**:

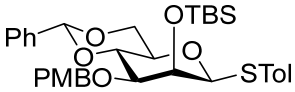

To a solution of compound **31**[1] (8.7 g, 17.6 mmol, 1 equiv) in dry 2,6-lutidine (70 mL) was added *tert*-butyldimethylsilyl trifluoromethanesulfonate (TBSOTf, 6.0 mL, 26.1 mmol, 1.5 equiv) and the reaction mixture was allowed to stir vigorously at room temperature for 4 h. The reaction mixture was quenched with MeOH and concentrated *in vacuo*. The residue was purified by flash column chromatography by gradient elution of hexane: EtOAc (1% EtOAc in hexane to 15% EtOAc/hexane) to afford compound **32** (8.68 g, 14.3 mmol, 81%) as a white foam.

$[\alpha]_D^{22}$ -11.7 (*c* 1.0, $CHCl_3$).

^{1}H NMR (600 MHz, $CDCl_3$) δ 7.56 – 7.54 (m, 2H, H_o-Ph), 7.43 – 7.37 (m, 5H, H_p-, H_m-Ph; H_o-STol), 7.32 – 7.30 (m, 2H, H_o-PMB), 7.13 (d, *J* = 8.1 Hz, 2H, H_m-STol), 6.89 – 6.87 (m, 2H, H_m-PMB), 5.67 (s, 1H, benzylidene-CH), 4.80 – 4.78 (m, 2H, OCH_2Ar; H-1), 4.71 (d, *J* = 11.8 Hz, 1H, OCH_2Ar), 4.35 (dd, *J* = 10.5, 4.8 Hz, 1H, H-6a), 4.33 (dd, *J* = 2.9, 1.1 Hz, 1H, H-2), 4.16 (t, *J* = 9.4 Hz, 1H, H-4), 3.96 (t, *J* = 10.3 Hz, 1H, H-6b), 3.83 (s, 3H, CH_3-PMB), 3.58 (dd, *J* = 9.7, 2.9 Hz, 1H, H-3), 3.42 (td, *J* = 9.7, 4.8 Hz, 1H, H-5), 2.36 (s, 3H, CH_3-STol), 1.04 (s, 9H, Si-$C(CH_3)_3$), 0.26 (s, 3H, Si-CH_3), 0.21 (s, 3H, Si-CH_3).

^{13}C{^{1}H} NMR (151 MHz, $CDCl_3$) δ 159.3 (C_p-PMB), 137.8 (C_p-STol), 137.3 (C_i-Ph), 132.0 (C_i-STol), 131.1 (C_o-STol), 130.3 (C_i-PMB), 129.9 (C_o-PMB), 129.8 (C_m-STol), 129.0 (C_p-Ph), 128.3 (C_m-Ph), 126.2 (C_o-Ph), 113.7 (C_m-PMB), 101.6 (benzylidene-CH), 90.5 (C-1), 79.0 (C-4), 78.1 (C-3), 74.3 (C-2), 72.8 (OCH_2Ar), 71.9 (C-5), 68.9 (C-6), 55.3 (OCH_3-PMB), 26.4 (Si-$C(CH_3)_3$), 21.2 (CH_3-STol), 18.9 (Si-$C(CH_3)_3$), -3.5 (Si-CH_3), -4.4 (Si-CH_3).

ESI-HRMS: *m/z* calcd for $C_{34}H_{44}O_6SSi$ $[M+Na]^+$ 631.2520, found 631.2513.

***p*-Methylphenyl 4,6-*O*-benzylidene-2-*O*-*tert*-butyldimethylsilyl-thio-β-D-mannopyranoside 33:**

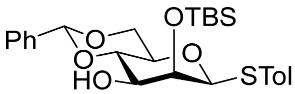

To a solution of compound **32** (8.21 g, 13.5 mmol, 1 equiv) in DCM/water mixture (v/v:20/1; 80/4 mL) was added, 3-dichloro-5,6-dicyano-1,4-benzoquinone (DDQ, 3.67 g, 16.2 mmol, 1.2 equiv) at room temperature. The reaction mixture was stirred for 2 h and quenched with saturated aqueous $NaHCO_3$ (20 mL). The organic layer was extracted, diluted in DCM (200 mL), washed with 2M NaOH (100 mL), brine solution (100 mL), dried over anhydrous Na_2SO_4 and concentrated *in vacuo*. The residue was purified by flash column chromatography by gradient elution of hexane: EtOAc (1% EtOAc in hexane to 15% EtOAc/hexane) to afford compound **33** (5.9 g, 12.08 mmol, 90%) as a white foam.

$[\alpha]_D^{22}$ – 72.5 (*c* 1.0, $CHCl_3$).

^{1}H NMR (600 MHz, $CDCl_3$) δ 7.50 – 7.48 (m, 2H, H_o-Ph), 7.39 – 7.33 (m, 5H, H_p-, H_m-Ph; H_o-STol), 7.12 (d, *J* = 8.0 Hz, 2H, H_m-STol), 5.57 (s, 1H, benzylidene-CH), 4.81 (d, *J* = 1.4 Hz, 1H, H-1), 4.34 – 4.31 (m, 2H, H-2, H-6a), 3.91 – 3.87 (m, 2H, H-4; H-6b), 3.78 (ddd, *J* = 9.8, 4.8, 3.1 Hz, 1H, H-3), 3.42 (td, *J* = 9.7, 4.9 Hz, 1H, H-5), 2.34 (s, 3H, CH_3-STol), 2.30 (d, *J* = 5.0 Hz, 1H, OH), 1.02 (s, 9H, Si-$C(CH_3)_3$), 0.28 (s, 3H, Si-CH_3), 0.22 (s, 3H, Si-CH_3).

^{13}C{^{1}H} NMR (151 MHz, $CDCl_3$) δ 137.6 (C_p-STol), 137.3 (C_i-Ph), 131.7 (C_i-STol), 131.4 (C_o-STol), 129.9 (C_m-STol), 129.4 (C_p-Ph), 128.5 (C_m-Ph), 126.4 (C_o-Ph), 102.3 (benzylidene-CH), 90.3 (C-1), 78.6 (C-4), 74.7 (C-2), 72.8 (C-3), 71.5 (C-5), 68.8 (C-6), 25.9 (Si-C(CH_3)$_3$), 21.2 (CH_3-STol), 18.9 (Si-C(CH_3)$_3$), -3.6 (Si-CH_3), -4.1 (Si-CH_3).

ESI-HRMS: *m/z* calcd for $C_{26}H_{36}O_5SSi$ $[M+Na]^+$ 511.1945, found 511.1924.

***p*-Methylphenyl 4,6-*O*-benzylidene-2-*O*-*tert*-butyldimethylsilyl-thio-β-D-*arabino*-hexopyranosid-3-uloside 34:**

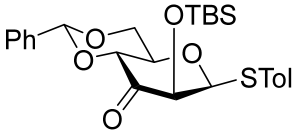

To a solution of compound **33** (4.1 g, 8.39 mmol, 1 equiv) in dry DCM (56 mL) was added Dess-Martin periodinane (DMP, 5.34 g, 12.59 mmol, 1.5 equiv) at room temperature under an argon atmosphere. The reaction mixture stirred for 4 h and quenched by adding saturated aqueous $Na_2S_2O_3$ (20 mL), and stirred for an additional 15 min. The organic layer was extracted, diluted in DCM (150 mL), washed with saturated aqueous $NaHCO_3$ (100 mL), dried over anhydrous Na_2SO_4 and concentrated *in vacuo*. The residue was purified by flash column chromatography by gradient elution of hexane: EtOAc (1% EtOAc in hexane to 15% EtOAc/hexane) to afford compound **34** (3.92 g, 8.05 mmol, 96%) as a white foam.

$[\alpha]_D^{22}$ +15.2 (*c* 1.0, $CHCl_3$).

^{1}H NMR (600 MHz, $CDCl_3$) δ 7.52 – 7.50 (m, 2H, H_o-Ph), 7.42 – 7.40 (m, 2H, H_o-STol), 7.38 – 7.34 (m, 3H, H_p-, H_m-Ph), 7.15 – 7.14 (m, 2H, H_m-STol), 5.62 (s, 1H, benzylidene-CH), 4.95 (d, *J* = 1.5 Hz, 1H, H-1), 4.88 (d, *J* = 9.9 Hz, 1H, H-4), 4.42 (dd, *J* = 10.6, 4.9 Hz, 1H, H-6a), 4.40 (d, *J* = 1.5 Hz, 1H, H-2), 4.04 (t, *J* = 10.2 Hz,

1H, H-6b), 3.61 (td, *J* = 9.9, 4.9 Hz, 1H, H-5), 2.35 (s, 3H, CH_3-STol), 0.97 (s, 9H, Si-C(CH_3)$_3$), 0.25 (s, 3H, Si-CH_3), 0.14 (s, 3H, Si-CH_3).

^{13}C{^{1}H} NMR (151 MHz, $CDCl_3$) δ 198.6 (C=O), 138.4 (C_p-STol), 136.6 (C_i-Ph), 132.3 (C_o-STol), 130.4 (C_i-STol), 130.1 (C_m-STol), 129.5 (C_p-Ph), 128.5 (C_m-Ph), 126.6 (C_o-Ph), 102.2 (benzylidene-CH), 92.8 (C-1), 80.9 (C-4), 80.0 (C-2), 73.1 (C-5), 69.3 (C-6), 25.9 (Si-C(*C*H_3)$_3$), 21.3 (CH_3-STol), 18.5 (Si-*C*(CH_3)$_3$), -4.7 (Si-CH_3), -4.9 (Si-CH_3).

ESI-HRMS: *m/z* calcd for $C_{26}H_{34}O_5SSi$ $[M+Na]^+$ 509.1788, found 509.1776.

***p*-Methylphenyl 4,6-*O*-benzylidene-2-*O*-*tert*-butyldimethylsilyl-3-*C*-methyl-thio-β-D-mannopyranoside 35:**

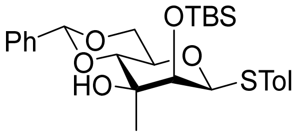

To a solution of compound **34** (1.55 g, 3.19 mmol, 1 equiv) in dry THF (20 mL) was added a 1.6 M solution of methyllithium in THF (2.4 mL, 3.84 mmol, 1.2 equiv) at -78 °C under an argon atmosphere. The reaction mixture was stirred for 1 h at the same temperature, then quenched with saturated aqueous NH_4Cl, (10 mL) diluted in EtOAc (80 mL). The organic layer was extracted, washed with brine (50 mL), dried over anhydrous Na_2SO_4 and concentrated *in vacuo*. The residue was purified by flash column chromatography by gradient elution of hexane: EtOAc (1% EtOAc in hexane to 20% EtOAc/hexane) to afford compound **35** (1.41 g, 2.8 mmol, 88%) as white foam.

$[\alpha]_D^{22}$ -67.2 (*c* 1.0, $CHCl_3$).

^{1}H NMR (600 MHz, $CDCl_3$) δ 7.50 **–** 7.49 (m, 2H, H_o-Ph), 7.41 – 7.35 (m, 5H, H_p-, H_m-Ph; H_o-STol), 7.15 – 7.13 (m, 2H, H_m-STol), 5.58 (s, 1H, benzylidene-CH), 4.99 (d, *J* = 1.2 Hz, 1H, H-1), 4.33 (dd, *J* = 10.5, 4.9 Hz, 1H, H-6a), 3.97 (d, *J* = 1.2 Hz, 1H, H-2), 3.88 – 3.82 (m, 2H, H-4, H-6b), 3.55 (td, *J* = 9.7, 4.9 Hz, 1H, H-5), 2.35 (CH_3-STol), 2.29 (s, 1H, OH), 1.38 (s, 3H, HO-C-CH_3), 1.04 (s, 9H, Si-C(CH_3)$_3$), 0.34 (s, 3H, Si-CH_3), 0.24 (s, 3H, Si-CH_3).

^{13}C{^{1}H} NMR (151 MHz, $CDCl_3$) δ 137.7 (C_p-STol), 137.6 (C_i-Ph), 131.9 (C_i-STol), 131.3 (C_o-STol), 129.9 (C_m-STol), 129.2 (C_p-Ph), 128.3 (C_m-Ph), 126.4 (C_o-Ph), 102.3 (benzylidene-*C*H), 88.8 (C-1), 81.6 (C-4), 79.9 (C-2), 72.6 (C-3), 70.0 (C-5), 69.3 (C-6), 26.5 (Si-C(*C*H_3)$_3$), 21.2 (CH_3-STol), 18.9 (HO-C-*C*H_3) 18.8 (Si-*C*(CH_3)$_3$), -3.5 (Si-CH_3), -3.7 (Si-CH_3).

ESI-HRMS: *m/z* calcd for $C_{27}H_{38}O_5SSi$ $[M+Na]^+$ 525.2101, found 525.2082.

***p*-Methylphenyl 4,6-*O*-benzylidene-3-*C*-methyl-thio-β-D-mannopyranoside 36:**

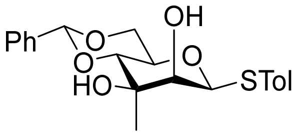

A solution of compound **35** (1.55 g, 3.08 mmol, 1 equiv) in THF (20 mL) was treated with a 1 M solution of tetrabutylammonium fluoride (TBAF, 6.1 mL, 6.1 mmol, 2 equiv) in THF at room temperature. The reaction

mixture was stirred for 2 h then concentrated *in vacuo*. The residue was purified by flash column chromatography by gradient elution of hexane: EtOAc (10% EtOAc in hexane to 60% EtOAc/hexane) to afford compound **36** (1.15 g, 2.96 mmol, 96%) as white foam.

$[\alpha]_D^{22}$ -60.3 (*c* 1.0, CH_3OH).

^{1}H NMR (600 MHz, $CDCl_3$) δ 7.49 – 7.46 (m, 2H, H_o-Ph), 7.44 – 7.42 (m, 2H, H_o-STol), 7.37 – 7.34 (m, 3H, H_p-, H_m-Ph), 7.15 – 7.14 (m, 2H, H_m-STol), 5.57 (s, 1H, benzylidene-CH), 4.99 (d, *J* = 1.2 Hz, 1H, H-1), 4.31 (dd, *J* = 10.4, 5.0 Hz, 1H, H-6a), 3.98 (d, *J* = 9.7 Hz, 1H, H-4), 3.89 – 3.86 (m, 2H, H-2, H-6b), 3.51 (td, *J* = 9.7, 5.0 Hz, 1H, H-5), 2.36 (s, 3H, CH_3-STol), 1.36 (s, 3H, HO-C-CH_3).

^{13}C{^{1}H} NMR (151 MHz, $CDCl_3$) δ 138.1 (C_p-STol), 137.4 (C_i-Ph), 132.0 (C_o-STol), 130.5 (C_i-STol), 129.9 (C_m-STol), 129.3 (C_p-Ph), 128.3 (C_m-Ph), 126.4 (C_o-Ph), 102.2 (benzylidene-CH), 87.4 (C-1), 80.8 (C-4), 77.3 (C-2), 72.4 (C-3), 69.8 (C-5), 68.9 (C-6), 21.2 (CH_3-STol), 18.5 (HO-C-CH$_3$).

ESI-HRMS: *m/z* calcd for $C_{21}H_{24}O_5S$ $[M+Na]^+$ 411.1237, found 411.1222.

***p*-Methylphenyl 2-*O*-benzyl-4,6-*O*-benzylidene-3-*C*-methyl-thio-β-D-mannopyranoside 37:**

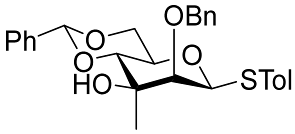

To a mixture of compound **36** (1.15g 2.96 mmol, 1 equiv), tetrabutylammonium bromide (TBAB, 0.05 g, 0.15 mmol, 0.05 equiv) and benzyl bromide (0.9 mL, 7.57 mmol, 2.6 equiv) in DCM (20 mL, 0.15 M), was added a 10% aqueous solution of NaOH (20 mL, v/v:1/1 with DCM) at room temperature. The reaction mixture was stirred for 36 h, diluted in DCM and washed with brine. The organic layer was dried over anhydrous Na_2SO_4 and concentrated *in vacuo*. The residue was purified by flash column chromatography by gradient elution of hexane: EtOAc (5% EtOAc in hexane to 25% EtOAc/hexane) to afford compound **37** (1.15 g, 2.29 mmol, 81%) as a white foam.

$[\alpha]_D^{22}$ -73.9 (*c* 1.0, $CHCl_3$).

^{1}H NMR (600 MHz, $CDCl_3$) δ 7.51 – 7.49 (m, 2H, H_o-OBn), 7.48 – 7.46 (m, 2H, H_o-Ph), 7.45 – 7.44 (m, 2H, H_o-STol), 7.41 – 7.39 (m, 2H, H_m-OBn), 7.37 – 7.33 (m, 4H, H_p-Ph; H_m-Ph; H_p-OBn), 7.16 (d, *J* = 7.9 Hz, 2H, H_m-STol), 5.55 (s, 1H, benzylidene-CH), 5.08 (d, *J* = 10.9 Hz, 1H, OCH_2Ph), 5.06 (d, *J* = 1.3 Hz, 1H, H-1), 4.85 (d, *J* = 10.9 Hz, 1H, OCH_2Ph), 4.31 (dd, *J* = 10.5, 5.0 Hz, 1H, H-6a), 3.90 (d, *J* = 9.6 Hz, 1H, H-4), 3.86 (t, *J* = 10.2 Hz, 1H, H-6b), 3.80 (d, *J* = 1.3 Hz, 1H, H-2), 3.52 (td, *J* = 9.7, 5.0 Hz, 1H, H-5), 2.58 (s, 1H, OH), 2.37 (s, 3H, CH_3-STol), 1.41 (s, 3H, HO-C-CH_3).

^{13}C{^{1}H} NMR (151 MHz, $CDCl_3$) δ 138.1 (C_p-STol), 137.6 (C_i-Ph), 137.5 (C_i-OBn), 131.8 (C_o-STol), 131.1 (C_i-STol), 130.0 (C_m-STol), 129.2 (C_p-Ph), 128.7 (C_m-Ph), 128.5 (C_m-OBn), 128.3 (C_o-OBn), 128.3 (C_p-OBn), 126.4 (C_o-Ph), 102.1 (benzylidene-CH), 88.5 (d, $^1J_{CH}$ = 153.7 Hz; C-1), 86.5 (C-2), 81.6 (C-4), 77.7 (OCH_2Ph), 73.0 (C-3), 70.0 (C-5), 69.0 (C-6), 21.3 (CH_3-STol), 18.6 (HO-C-CH$_3$).

ESI-HRMS: *m/z* calcd for $C_{28}H_{30}O_5S$ $[M+Na]^+$ 501.1706, found 501.1688.

p-Methylphenyl 3-O-benzoyl-2-O-benzyl-4,6-O-benzylidene-3-C-methyl-thio-β-D-mannopyranoside 38:

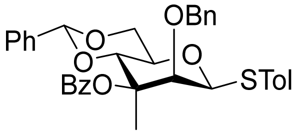

Prepared from compound **37** (205 mg, 0.428 mmol, 1 equiv) with the following quantities of reagents, temperature and time of a reaction according to General Procedure GP1.

i) Benzoic acid (522 mg, 4.274 mmol, 10 equiv) and CDI (693 mg, 4.274 mmol, 10 equiv) in THF (10 mL) under reflux condition.
ii) DBU (0.96 mL, 6.42 mmol, 15 equiv) was added at the beginning of the reaction, and the same amount was added after 12 h.

Compound **38** (234 mg, 0.402 mmol, 94%) was obtained as a white foam.

$[\alpha]_D^{22}$ -105.4 (*c* 1.0, $CHCl_3$).

^{1}H NMR (600 MHz, $CDCl_3$) δ 8.03 – 8.01 (m, 2H, H_o-OBz), 7.58 – 7.55 (m, 3H, H_p-OBz; H_o-Ph), 7.50 (d, *J* = 7.9 Hz, 2H, H_o-STol), 7.44 – 7.38 (m, 5H, H_m-OBz; H_m-, H_p-Ph), 7.28 – 7.26 (m, 2H, H_o-OBn), 7.21 (d, *J* = 7.9 Hz, 2H, H_m-STol), 7.18 – 7.14 (m, 3H, H_o-Ph; H_p-H_m-OBn), 5.76 (s, 1H, benzylidene-CH), 5.10 (d, *J* = 1.1 Hz, 1H, H-1), 5.05 (d, *J* = 1.1 Hz, 1H, H-2), 5.01 (d, *J* = 10.9 Hz, 1H, OCH_2Ph), 4.69 (d, *J* = 10.9 Hz, 1H, OCH_2Ph), 4.45 (d, *J* = 9.7 Hz, 1H, H-4), 4.41 (dd, *J* = 10.5, 4.9 Hz, 1H, H-6a), 4.01 (t, *J* = 10.2 Hz, 1H, H-6b), 3.75 (td, *J* = 9.7, 4.9 Hz, 1H, H-5), 2.40 (s, 3H, CH_3-STol), 1.90 (s, 3H, HO-C-CH_3).

^{13}C{^{1}H} NMR (151 MHz, $CDCl_3$) δ 166.5 (C=O), 138.1 (C_p-STol), 137.9 (C_i-Ph), 137.6 (C_i-OBn), 133.1 (C_p-OBz), 132.1 (C_o-STol), 131.2 (C_i-OBz), 131.0 (C_i-STol), 130.0 (C_m-STol), 129.8 (C_o-OBz), 129.1 (C_p-Ph), 128.4 (C_o-OBz), 128.4 (C_m-Ph), 128.1 (C_m-OBn), 128.0 (C_o-OBn), 127.5 (C_p-OBn), 126.2 (C_o-Ph), 101.8 (benzylidene-CH), 89.0 (C-1), 83.2 (C-2), 82.5 (C-3), 79.4 (C-4), 76.6 (OCH_2Ph), 69.2 (C-5), 69.0 (C-6), 21.3 (CH_3-STol), 16.3 (BzO-C-CH_3).

ESI-HRMS: *m/z* calcd for $C_{35}H_{34}O_6S$ $[M+Na]^+$ 605.1968, found 605.1951.

p-Methylphenyl 3-O-(benzoyl-α-^{13}C)-2-O-benzyl-4,6-O-benzylidene-3-C-methyl-thio-β-D-mannopyranoside ^{13}C-38:

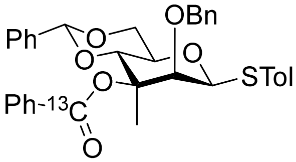

Prepared from compound **37** (250 mg, 0.522 mmol, 1 equiv) with the following quantities of reagents, temperature and time of reaction according to General Procedure GP1.

i) Benzoic acid-α-^{13}C (650 mg, 5.224 mmol, 10 equiv) and CDI (864 mg, 5.224 mmol, 10 equiv) in THF (12.5 mL) under reflux condition.
ii) DBU (1.2 mL, 8.02 mmol, 15 equiv) was added at the beginning of the reaction, and the same amount was added after 12 h.

Compound **^{13}C-38** (303 mg, 0.519 mmol, 99%) was obtained as white foam.

$[\alpha]_D^{22}$ -111.4 (*c* 0.5, $CHCl_3$).

^{1}H NMR (600 MHz, $CDCl_3$) δ 8.01 – 8.00 (m, 2H, H_o-OBz), 7.56 – 7.54 (m, 3H, H_p-OBz; H_o-Ph), 7.49 (d, *J* = 7.9 Hz, 2H, H_o-STol), 7.43 – 7.37 (m, 5H, H_m-OBz; H_o-OBn; H_p-Ph), 7.26 – 7.24 (m, 2H, H_o-OBn), 7.19 (d, *J* = 7.9 Hz, 2H, H_m-STol), 7.16 – 7.14 (m, 3H, H_m-Ph; H_p-H_m-OBn), 5.75 (s, 1H, benzylidene-CH), 5.10 (s, 1H, H-1), 5.05 (s, 1H, H-2), 5.01 (d, *J* = 10.9 Hz, 1H, OCH_2Ph), 4.68 (d, *J* = 10.9 Hz, 1H, OCH_2Ph), 4.44 (d, *J* = 10.0 Hz, 1H, H-4), 4.40 (dd, *J* = 10.4, 4.7 Hz, 1H, H-6a), 4.00 (t, *J* = 10.2 Hz, 1H, H-6b), 3.74 (td, *J* = 9.7, 4.7 Hz, 1H, H-5), 2.39 (s, 3H, CH_3-STol), 1.89 (s, 3H, ArO-C-CH_3).

^{13}C{^{1}H} NMR (151 MHz, $CDCl_3$) δ 166.5 (^{13}C=O), 138.1 (C_p-STol), 137.9 (C_i-Ph), 137.6 (C_i-OBn), 133.1 (C_p-OBz), 132.1 (C_o-STol), 131.2 (C_i-STol), 131.2 (d, *J* = 74.4 Hz, C_i-OBz), 130.0 (C_m-STol), 129.8 (d, *J* = 2.4 Hz, C_o-OBz), 129.1 (C_p-Ph), 128.4 (C_m-OBz), 128.4 (C_m-Ph), 128.1 (C_m-OBn), 128.0 (C_o-OBn), 127.5 (C_p-OBn), 126.2 (C_o-Ph), 101.8 (benzylidene-CH), 89.0 (C-1), 83.2 (C-2), 82.5 (d, *J* = 2.6 Hz, C-3), 79.4 (d, J = 3.7 Hz, C-4), 76.6 (O*C*H_2Ph), 69.2 (C-5), 69.0 (C-6), 21.3 (CH_3-STol), 16.3 (BzO-C-*C*H_3).

ESI-HRMS: *m/z* calcd for $C_{34}{}^{13}CH_{34}O_6S$ $[M+Na]^+$ 606.2002, found 606.1985.

***p*-Methylphenyl 2-*O*-benzyl-4,6-*O*-benzylidene-3-*O*-*p*-nitrobenzoyl-3-*C*-methyl-thio-β-D-mannopyranoside 39:**

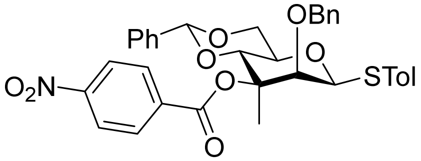

Prepared from compound **37** (150 mg, 0.313 mmol, 1 equiv) with the following quantities of reagents, temperature and time of reaction according to General Procedure GP1.

i) *p*-Nitrobenzoic acid (534 mg, 3.134 mmol, 10 equiv) and CDI (519 mg, 3.134 mmol, 10 equiv) in THF (10 mL) under reflux condition.
ii) DBU (0.72 mL, 4.82 mmol, 15 equiv) was added at the beginning of the reaction and the same amount was added after 12 h.

Compound **39** (181 mg, 0.288 mmol, 92%) was obtained as white solid.

$[\alpha]_D^{22}$ -126.0 (*c* 1.0, $CHCl_3$).

^{1}H NMR (600 MHz, $CDCl_3$) δ 8.19 – 8.17 (m, 2H, H_m-PNB), 8.02 – 8.00 (m, 2H, H_o-PNB), 7.53 – 7.50 (m, 2H, H_o-Ph), 7.49 – 7.48 (m, 2H, H_o-STol), 7.41 – 7.36 (m, 3H, H_o-OBn; H_p-Ph), 7.35 – 7.33 (m, 2H, H_o-STol), 7.20 – 7.19 (m, 2H, H_m-STol; H_m-Ph), 7.08 – 7.02 (m, 3H, H_p-OBn; H_m-OBn), 5.75 (s, 1H, benzylidene-CH), 5.13 (d, *J* = 11.5 Hz, 1H, OCH_2Ph), 5.09 (d, *J* = 1.1 Hz, 1H, H-1), 5.00 (d, *J* = 1.1 Hz, 1H, H-2), 4.56 (d, *J* = 11.5 Hz, 1H, OCH_2Ph), 4.43 (d, *J* = 9.6 Hz, 1H, H-4), 4.40 (dd, *J* = 10.6, 4.9 Hz, 1H, H-6a), 4.00 (t, *J* = 10.2 Hz, 1H, H-6b), 3.73 (td, *J* = 9.8, 4.9 Hz, 1H, H-5), 2.39 (s, 3H, CH_3-STol), 1.87 (s, 3H, ArO-C-CH_3).

^{13}C{^{1}H} NMR (151 MHz, $CDCl_3$) δ 164.3 (C=O), 150.4 (C_p-PNB), 138.3 (C_p-STol), 137.9 (C_i-Ph), 137.4 (C_i-OBn), 136.2 (C_i-PNB), 132.2 (C_o-STol), 130.8 (C_i-STol; C_o-PNB), 130.1 (C_m-STol), 129.3 (C_p-Ph), 128.4 (C_m-Ph), 128.1 (C_m-OBn), 127.5 (C_p-OBn), 127.4 (C_o-OBn), 126.2 (C_m-Ph), 123.3 (C_m-PNB), 101.9 (benzylidene-CH), 88.9 (C-

1), 83.8 (C-2), 83.6 (C-3), 79.3 (C-4), 77.0 (OCH_2Ph), 69.2 (C-5), 68.9 (C-6), 21.3 (CH_3-STol), 16.1 (ArO-C-CH_3).

ESI-HRMS: *m/z* calcd for $C_{35}H_{33}O_8NS$ $[M+Na]^+$ 650.1819, found 650.1803.

***p*-Methylphenyl 3-*O*-benzoyl-2-*O*-benzyl-4,6-*O*-benzylidene-3-*C*-methyl-thio-β-D-mannopyranoside *S*-oxide 40:**

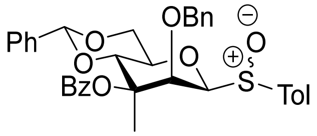

Compound **38** (330 mg, 0.566 mmol) in DCM was treated with *m*CPBA (152 mg, 0.678 mmol) according to General Procedure GP2 to afford compound **40** (276 mg, 0.461 mmol, 81%) in a ratio of two diastereoisomers (1.46/1: less-polar/polar).

Less-polar diastereoisomer (white solid)**:**

$[\alpha]_D^{22}$ -23.6 (*c* 1.0, $CHCl_3$).

^{1}H NMR (600 MHz, $CDCl_3$) δ 8.02 (d, *J* = 7.6 Hz, 2H, H_o-OBz), 7.66 (d, *J* = 8.0 Hz, 2H, H_m-S(O)Tol), 7.54 (t, *J* = 7.5 Hz, 1H, H_p-OBz), 7.50 – 7.48 (m, 2H, H_o-Ph), 7.41 (t, *J* = 7.7 Hz, 2H, H_m-OBz), 7.38 – 7.33 (m, 5H, H_o-S(O)Tol; H_p-, H_m-Ph), 7.27 – 7.25 (m, 2H, H_o-OBn), 7.19 – 7.14 (m, 3H, H_p- H_m-OBn), 5.70 (s, 1H, benzylidene-CH), 5.33 (s, 1H, H-2), 5.06 (d, *J* = 10.8 Hz, 1H, OCH_2Ph), 4.72 (d, *J* = 10.8 Hz, 1H, OCH_2Ph), 4.46 (d, *J* = 9.7 Hz, 1H, H-4), 4.30 (s, 1H, H-1), 4.16 (dd, *J* = 10.4, 4.8 Hz, 1H, H-6a), 3.86 (t, *J* = 10.1 Hz, 1H, H-6b), 3.54 (td, *J* = 9.7, 4.9 Hz, 1H, H-5), 2.45 (s, 3H, CH_3-S(O)Tol), 1.83 (s, 3H, BzO-C-CH_3).

$^{13}C\{^1H\}$ NMR (151 MHz, $CDCl_3$) δ 166.2 (C=O), 142.2 (C_i-S(O)Tol), 138.8 (C_p-S(O)Tol), 138.0 (C_i-Ph), 137.4 (C_i-OBn), 133.2 (C_p-OBz), 130.9 (C_i-OBz), 129.9 (C_o-OBz), 129.9 (C_o-S(O)Tol), 129.2 (C_p-Ph), 128.4 (C_m-OBz), 128.4 (C_m-Ph), 128.3 (C_m-OBn), 128.2 (C_o-OBn), 127.7 (C_p-OBn), 126.2 (C_o-Ph), 125.3 (C_m-S(O)Tol), 102.0 (benzylidene-CH), 95.8 (C-1), 82.1 (C-3), 79.7 (C-4), 77.7 (C-2), 76.3 (OCH_2Ph), 69.7 (C-5), 68.5 (C-6), 21.6 (CH_3-STol), 16.0 (BzO-C-CH_3).

ESI-HRMS: *m/z* calcd for $C_{35}H_{34}O_7S$ $[M+Na]^+$ 621.1917, found 621.1909.

Polar diastereoisomer (colorless amorphous solid)**:**

$[\alpha]_D^{22}$ – 62.8 (*c* 1.0, $CHCl_3$).

^{1}H NMR (600 MHz, C_6D_6) δ 8.13 – 8.11 (m, 2H, H_o-OBz), 7.64 – 7.63 (m, 2H, H_m-S(O)Tol), 7.55 – 7.54 (m, 2H, H_o-Ph), 7.34 – 7.33 (m, 2H, H_o-OBn), 7.14 (t, *J* = 7.5 Hz, 2H, H_m-Ph), 7.08 – 7.01 (m, 4H, H_p-Ph; H_p-OBz; H_m-OBn), 6.98 – 6.96 (m, 3H, H_m-OBz; H_p-OBn), 6.88 (d, *J* = 7.9 Hz, 2H, H_o-S(O)Tol), 5.23 (s, 1H, benzylidene-CH), 4.92 (d, *J* = 10.7 Hz, 1H, OCH_2Ph), 4.84 (s, 1H, H-2), 4.80 (d, *J* = 10.7 Hz, 1H, OCH_2Ph), 4.48 (d, *J* = 9.7 Hz, 1H, H-4), 4.44 (s, 1H, H-1), 4.20 (dd, *J* = 10.3, 4.8 Hz, 1H, H-6a), 3.64 (t, *J* = 10.1 Hz, 1H, H-6b), 3.46 (td, *J* = 9.7, 4.8 Hz, 1H, H-5), 1.94 (s, 3H, CH_3-S(O)Tol), 1.56 (s, 3H, BzO-C-CH_3).

$^{13}C\{^1H\}$ NMR (151 MHz, C_6D_6) δ 166.5 (C=O), 142.4 (C_i-S(O)Tol), 140.0 (C_p-S(O)Tol), 138.4 (C_i-Ph), 138.3 (C_i-OBn), 133.2 (C_p-OBz), 131.5 (C_i-OBz), 130.3 (C_o-S(O)Tol), 130.0 (C_o-OBz), 129.0 (C_p-Ph), 128.6 (C_m-OBz), 128.5 (C_m-Ph), 128.3 (C_m-OBn), 128.3 (C_o-OBn), 128.0 (C_p-OBn), 126.6 (C_o-Ph), 126.3 (C_m-S(O)Tol), 101.9

(benzylidene-CH), 97.3 (C-1), 83.0 (C-3), 79.8 (C-4), 79.6 (C-2), 75.3 (OCH_2Ph), 70.6 (C-5), 68.8 (C-6), 21.2 (CH_3-S(O)Tol), 16.1 (BzO-C-CH_3).

ESI-HRMS: *m/z* calcd for $C_{35}H_{34}O_7S$ $[M+Na]^+$ 621.1917, found 621.1904.

***p*-Methylphenyl 3-*O*-(benzoyl-α-^{13}C)-2-*O*-benzyl-4,6-*O*-benzylidene-3-*C*-methyl-thio-β-D-mannopyranoside *S*-oxide ^{13}C-40:**

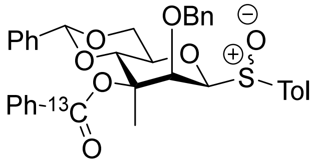

Compound **^{13}C-38** (303 mg, 0.519 mmol) in DCM was treated with *m*CPBA (140 mg, 0.623 mmol) according to General Procedure GP2 to afford compound **^{13}C-40** (256 mg, 0.427 mmol, 82%) in a ratio of two diastereoisomers (1.55/1:less polar/polar).

Less-polar diastereoisomer (white solid)**:**

$[\alpha]_D^{22}$ -31.7 (*c* 1.0, $CHCl_3$).

^{1}H NMR (600 MHz, $CDCl_3$) δ 8.01 (dd, *J* = 8.0, 4.1 Hz, 2H, H_o-OBz), 7.66 (d, *J* = 7.9 Hz, 2H, H_m-S(O)Tol), 7.55 (t, *J* = 7.4 Hz, 1H, H_p-OBz), 7.50 – 7.48 (m, 2H, H_o-Ph), 7.41 (t, *J* = 7.7 Hz, 2H, H_m-OBz), 7.38 – 7.35 (m, 5H, H_o-S(O)Tol; H_p-, H_m-Ph), 7.27 – 7.26 (m, 2H, H_o-OBn), 7.19 – 7.14 (m, 3H, H_p- H_m-OBn), 5.70 (s, 1H, benzylidene-CH), 5.33 (s, 1H, H-2), 5.06 (d, *J* = 10.8 Hz, 1H, OCH_2Ph), 4.71 (d, *J* = 10.8 Hz, 1H, OCH_2Ph), 4.46 (d, *J* = 9.7 Hz, 1H, H-4), 4.29 (s, 1H, H-1), 4.16 (dd, *J* = 10.4, 4.9 Hz, 1H, H-6a), 3.86 (t, *J* = 10.1 Hz, 1H, H-6b), 3.54 (td, *J* = 9.7, 4.9 Hz, 1H, H-5), 2.45 (s, 3H, CH_3-S(O)Tol), 1.82 (s, 3H, ArO-C-C*H*$_3$).

^{13}C{^{1}H} NMR (151 MHz, $CDCl_3$) δ 166.2 (^{13}C=O), 142.2 (C_i-S(O)Tol), 138.9 (C_p-S(O)Tol), 138.0 (C_i-Ph), 137.4 (C_i-OBn), 133.2 (C_p-OBz), 131.0 (d, *J* = 74.6 Hz, C_i-OBz), 129.9 (d, *J* = 2.6 Hz, C_o-OBz), 129.9 (C_o-S(O)Tol), 129.2 (C_p-Ph), 128.5 (d, *J* = 4.5 Hz, C_m-OBz), 128.4 (C_m-Ph), 128.3 (C_m-OBn), 128.2 (C_o-OBn), 127.7 (C_p-OBn), 126.2 (C_o-Ph), 125.3 (C_m-S(O)Tol), 102.0 (benzylidene-CH), 95.9 (C-1), 82.1 (d, *J* = 2.7 Hz, C-3), 79.7 (d, *J* = 3.6 Hz, C-4), 77.7 (C-2), 76.3 (OCH_2Ph), 69.8 (C-5), 68.5 (C-6), 21.6 (CH_3-STol), 16.1 (α-^{13}C-BzO-C-C*H*$_3$).

ESI-HRMS: *m/z* calcd for $C_{34}{}^{13}CH_{34}O_7S$ $[M+Na]^+$ 622.1951, found 622.1940.

Polar diastereoisomer (colorless amorphous solid)**:**

$[\alpha]_D^{22}$ – 67.7 (*c* 1.0, $CHCl_3$).

^{1}H NMR (600 MHz, C_6D_6) δ 8.12 – 8.10 (m, 2H, H_o-OBz), 7.65 (d, *J* = 7.9 Hz, 2H, H_m-S(O)Tol), 7.55 – 7.54 (m, 2H, H_o-Ph), 7.34 – 7.33 (m, 2H, H_o-OBn), 7.16 – 7.13 (m, 2H, H_m-Ph), 7.09 – 7.03 (m, 4H, H_p-Ph; H_p-OBz; H_m-OBn), 6.99 – 6.96 (m, 3H, H_m-OBz; H_p-OBn), 6.89 (d, *J* = 7.9 Hz, 2H, H_o-S(O)Tol), 5.24 (s, 1H, benzylidene-CH), 4.90 (d, *J* = 10.7 Hz, 1H, OCH_2Ph), 4.83 (s, 1H, H-2), 4.80 (d, *J* = 10.7 Hz, 1H, OCH_2Ph), 4.51 (s, 1H, H-1), 4.49 (d, *J* = 9.7 Hz, 1H, H-4), 4.20 (dd, *J* = 10.3, 4.8 Hz, 1H, H-6a), 3.66 (t, *J* = 10.1 Hz, 1H, H-6b), 3.52 (td, *J* = 9.7, 4.8 Hz, 1H, H-5), 1.94 (s, 3H, CH_3-S(O)Tol), 1.58 (s, 3H, α-^{13}C-BzO-C-C*H*$_3$).

^{13}C{^{1}H} NMR (151 MHz, C_6D_6) δ 166.5 (^{13}C=O), 142.5 (C_i-S(O)Tol), 139.9 (C_p-S(O)Tol), 138.3 (C_i-Ph), 138.3 (C_i-OBn), 133.2 (C_p-OBz), 131.5 (d, *J* = 74.4 Hz, C_i-OBz), 130.3 (C_o-S(O)Tol), 130.0 (d, *J* = 2.5 Hz, C_o-OBz), 129.0 (C_p-Ph), 128.6 (d, *J* = 4.5 Hz, C_m-OBz), 128.5 (C_m-Ph), 128.3 (C_m-OBn), 128.3 (C_o-OBn), 128.0 (C_p-OBn),

126.6 (C_o-Ph), 126.4 (C_m-S(O)Tol), 101.9 (benzylidene-CH), 97.3 (C-1), 83.0 (d, *J* = 2.6 Hz, C-3), 79.8 (d, *J* = 3.7 Hz, C-4), 79.6 (C-2), 75.3 (OCH_2Ph), 70.6 (C-5), 68.8 (C-6), 21.2 (CH_3- S(O)Tol), 16.1 (α-^{13}C-BzO-C-*C*H_3).

ESI-HRMS: *m/z* calcd for $C_{34}{}^{13}CH_{34}O_7S$ $[M+Na]^+$ 622.1951, found 622.1935.

***p*-Methylphenyl 2-*O*-benzyl-4,6-*O*-benzylidene-3-*O*-*p*-nitrobenzoyl-3-*C*-methyl-thio-β-D-mannopyranoside *S*-oxide 41:**

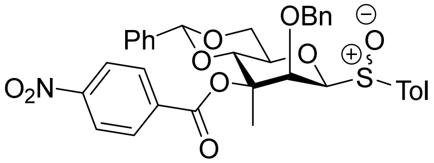

Compound **39** (140 mg, 0.223 mmol) in DCM was treated with *m*CPBA (60 mg, 0.268 mmol) according to General Procedure GP2 to afford compound **41** (122 mg, 0.189 mmol, 85%) in a ratio of two diastereoisomers (1.5/1:less polar/polar).

Less-polar diastereoisomer (white solid)**:**

$[\alpha]_D^{22}$ -47.1 (*c* 1.0, $CHCl_3$).

^{1}H NMR (600 MHz, $CDCl_3$) δ 8.21 – 8.19 (m, 2H, H_m-PNB), 8.07 – 8.05 (m, 2H, H_o-PNB), 7.68 – 7.67 (m, 2H, H_m-S(O)Tol), 7.51 – 7.45 (m, 2H, H_o-Ph), 7.39 – 7.35 (m, 5H, H_o-S(O)Tol; H_p-, H_m-Ph), 7.24 – 7.22 (m, 2H, H_o-OBn), 7.13 – 7.11 (m, 2H, H_m-OBn), 7.09 – 7.07 (m, 1H, H_p-OBn), 5.72 (s, 1H, benzylidene-CH), 5.32 (d, *J* = 1.2 Hz, 1H, H-2), 5.18 (d, *J* = 11.4 Hz, 1H, OCH_2Ph), 4.66 (d, *J* = 11.4 Hz, 1H, OC*H*$_2$Ph), 4.47 (d, *J* = 9.7 Hz, 1H, H-4), 4.30 (d, *J* = 1.2 Hz, 1H, H-1), 4.19 (dd, *J* = 10.5, 4.9 Hz, 1H, H-6a), 3.89 (t, *J* = 10.1 Hz, 1H, H-6b), 3.55 (td, *J* = 9.7, 4.9 Hz, 1H, H-5), 2.46 (s, 3H, CH_3-S(O)Tol), 1.83 (s, 3H, ArO-C-C*H*$_3$).

^{13}C{^{1}H} NMR (151 MHz, $CDCl_3$) δ 164.1 (C=O), 150.5 (C_p-PNB), 142.4 (C_i-S(O)Tol), 138.6 (C_p-S(O)Tol), 137.9 (C_i-OBn), 137.2 (C_i-Ph), 136.1 (C_i-PNB), 130.8 (C_o-PNB), 129.9 (C_o-S(O)Tol), 129.4 (C_p-Ph), 128.4 (C_m-Ph), 128.2 (C_m-OBn), 127.6 (C_o-OBn), 127.6 (C_p-OBn), 126.2 (C_o-Ph), 125.3 (C_m-S(O)Tol), 123.4 (C_m-PNB), 102.1 (benzylidene-CH), 95.8 (C-1), 83.2 (C-3), 79.5 (C-4), 78.2 (C-2), 76.6 (OCH_2Ph), 69.7 (C-5), 68.4 (C-6), 21.6 (CH_3-S(O)Tol), 15.8 (ArO-C-*C*H_3).

ESI-HRMS: *m/z* calcd for $C_{35}H_{33}O_9NS$ $[M+Na]^+$ 666.1768, found 666.1754.

Polar diastereoisomer (colorless amorphous solid)**:**

$[\alpha]_D^{22}$ –67.7 (*c* 1.0, $CHCl_3$).

^{1}H NMR (600 MHz, C_6D_6) δ 7.73 (d, *J* = 8.8 Hz, 2H, H_m-PNB), 7.64 (d, *J* = 7.7 Hz, 2H, H_m-S(O)Tol), 7.58 (d, *J* = 8.8 Hz, 2H, H_o-PNB), 7.54 (d, *J* = 7.2 Hz, 2H, H_o-Ph), 7.27 – 7.25 (m, 2H, H_o-OBn), 7.19 – 7.16 (m, 2H, H_m-Ph), 7.10 – 7.07 (m, 1H, H_p-Ph), 6.96 (t, *J* = 7.5 Hz, 2H, H_m-OBn), 6.92 – 6.87 (m, 3H, H_m-S(O)Tol; H_p-OBn), 5.28 (s, 1H, benzylidene-CH), 4.98 (d, *J* = 10.8 Hz, 1H, OCH_2Ph), 4.78 (s, 1H, H-2), 44.58 (d, *J* = 10.8 Hz, 1H, OCH_2Ph), 4.44 (d, *J* = 9.6 Hz, 1H, H-4), 4.42 (s, 1H, H-1), 4.20 (dd, *J* = 10.4, 4.7 Hz, 1H, H-6a), 3.65 (t, *J* = 10.1 Hz, 1H, H-6b), 3.43 (td, *J* = 9.6, 4.7 Hz, 1H, H-5), 1.96 (s, 3H, CH_3-S(O)Tol), 1.53 (s, 3H, ArO-C-C*H*$_3$).

^{13}C{^{1}H} NMR (151 MHz, C_6D_6) δ 164.6 (C=O), 150.7 (C_p-PNB), 142.6 (C_i-S(O)Tol), 139.8 (C_p-S(O)Tol), 138.1 (C_i-OBn), 138.0 (C_i-Ph), 135.8 (C_i-PNB), 130.6 (C_o-PNB), 130.3 (C_o-S(O)Tol), 129.3 (C_p-Ph), 128.6 (C_m-Ph), 128.4 (C_m-OBn), 128.3 (C_o-OBn), 128.0 (C_p-OBn), 126.6 (C_o-Ph), 126.3 (C_m-S(O)Tol), 123.4 (C_m-PNB), 102.2

(benzylidene-CH), 96.5 (C-1), 84.0 (C-3), 79.8 (C-4), 79.6 (C-2), 75.6 (OCH_2Ph), 70.5 (C-5), 68.7 (C-6), 21.2 (CH_3-S(O)Tol), 15.9 (ArO-C-CH_3).

ESI-HRMS: *m/z* calcd for $C_{35}H_{33}O_9NS$ $[M+Na]^+$ 666.1768, found 666.1760.

4,6-*O*-Benzylidene-3-*C*-methyl-D-mannopyranose 42:

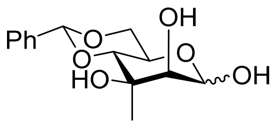

To a solution of compound **35** (0.43 g, 0.85 mmol, 1 equiv) in an acetone/water mixture (v/v:10/; 10/1 mL) was added NBS (0.46 g, 2.58 mmol, 3 equiv) at 0 °C. The reaction mixture stirred for 1 h at the same temperature and quenched by adding saturated aqueous $Na_2S_2O_3$, and stirred for an additional 15 min. The organic layer was extracted with EtOAc, washed with saturated aqueous $NaHCO_3$ (20 mL), dried over anhydrous Na_2SO_4 and concentrated *in vacuo*. To a solution of crude residue in dry THF (10 mL) was added a 1 M solution of TBAF (1.7 mL, 1.7 mmol, 2 equiv) in THF at room temperature. The reaction mixture was stirred for 2 h then concentrated *in vacuo*. The residue was purified by flash column chromatography by gradient elution of DCM: MeOH (1% MeOH in DCM to 10% MeOH/DCM) to afford compound **42** (0.17 g, 0.62 mmol, 72%; α:β mixture of anomers ~1.3:1) as a white solid.

^{1}H NMR (600 MHz, CD_3OD) δ 7.50 – 7.48 (m, 4H, H_o-Ph-α; H_o-Ph-β), 7.36 – 7.33 (m, 6H, H_p-, H_m-Ph-α; H_p-, H_m-Ph-β), 5.59 (s, 1H, benzylidene-CH-α), 5.58 (s, 1H, benzylidene-CH-β), 5.14 (s, 1H, H-1-α), 5.00 (s, 1H, H-1-β), 4.24 (dd, *J* = 10.2, 4.9 Hz, 1H, H-6a-β), 4.18 (dd, *J* = 10.1, 4.9 Hz, 1H, H-1-α), 3.95 (td, *J* = 9.9, 4.9 Hz, 1H, H-5-α), 3.87 – 3.85 (m, 2H, H-4-β; H-4-α), 3.79 – 3.71 (m, 2H, H-6b-α; H-6b-β), 3.52 – 3.48 (m, 3H, H-5-β; H-2-α; H-2-β), 1.46 (s, 3H, HO-C-CH_3-α), 1.33 (s, 3H, HO-C-CH_3-β).

^{13}C{^{1}H} NMR (151 MHz, CD_3OD) δ 139.5 (C_i-Ph-α), 139.4 (C_i-Ph-β), 129.9 (C_p-Ph-β), 129.8 (C_p-Ph-α), 129.0 (C_m-Ph-α; C_m-Ph-β), 127.5 (C_o-Ph-α), 127.5 (C_o-Ph-β), 103.3 (benzylidene-CH-β), 103.3 (benzylidene-CH-α), 97.1 ($^1J_{CH}$ = 167.9 Hz, C-1-α), 94.9 ($^1J_{CH}$ = 159.8 Hz, C-1-β), 82.8 (C-4-α), 82.5 (C-4-β), 78.0 (C-2-β), 77.2 (C-2-α), 72.9 (C-3-β), 71.9 (C-3-α), 70.4 (C-6-α), 70.1 (C-6-β), 67.1 (C-5-β), 63.5 (C-5-α) 19.6 (HO-C-*C*H_3-α), 18.6 (HO-C-*C*H_3-β).

ESI-HRMS: *m/z* calcd for $C_{14}H_{18}O_6$ $[M+Na]^+$ 305.0996, found 305.0994.

***p*-Methylphenyl 4,6-*O*-benzylidene-3-*C*-methyl-thio-α-D-mannopyranoside 43:**

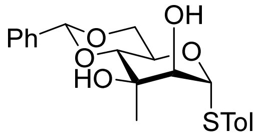

To a mixture of **42** (0.15 g, 0.53 mmol, 1 equiv), *p*-methylbenzenethiol (0.33 g, 2.66 mmol, 5 equiv) and Et_3N (0.74 mL, 5.31 mmol, 10 equiv) in acetonitrile/water (v/v:1/1; 10 mL) was added 3-chloro-1,3-dimethylimidazolinium chloride (DMC, 0.27 g, 1.60 mmol, 3 equiv) at 0 °C. The reaction mixture was stirred for 3 h at the same temperature, then diluted in EtOAc, washed with a saturated solution of $NaHCO_3$, brine, dried over anhydrous Na_2SO_4 and concentrated *in vacuo*. The residue was purified by flash column chromatography by gradient elution of hexane: EtOAc (10% EtOAc in hexane to 60% EtOAc/hexane) to afford compound **43** (0.17 g, 0.44 mmol, 83%) as a white foam.

$[\alpha]_D^{22}$ +152.8 (*c* 0.5, $CHCl_3$).

^{1}H NMR (600 MHz, $CDCl_3$) δ 7.50 (dd, *J* = 7.3, 2.4 Hz, 2H, H_o-Ph), 7.41 – 7.34 (m, 5H, H_o-STol; H_p-, H_m-Ph), 7.13 (d, *J* = 8.0 Hz, 2H, H_m-STol), 5.58 (s, 1H, benzylidene-CH), 5.52 (s, 1H, H-1), 4.28 – 4.24 (m, 2H, H-5,H-6a), 3.99 – 3.97 (m, 2H, H-4, H-2), 3.82 – 3.78 (m, 1H, H-6b), 3.27 (s, 1H, CH-O*H*), 2.66 (s, 1H, *H*O-C-CH_3) 2.34 (s, 3H, CH_3-STol), 1.62 (s, 3H, HO-C-C*H*$_3$).

^{13}C{^{1}H} NMR (151 MHz, $CDCl_3$) δ 138.1 (C_p-STol), 137.5 (C_i-Ph), 132.3 (C_o-STol), 130.9 (C_i-STol), 130.0 (C_m-STol), 129.4 (C_p-Ph), 128.5 (C_m-Ph), 126.5 (C_o-Ph), 102.4 (benzylidene-CH), 88.6 ($^1J_{CH}$ = 163.6 Hz, C-1), 81.4 (C-4), 77.3 (C-2), 71.6 (C-3), 69.0 (C-6), 63.0 (C-5), 21.3 (CH_3-STol), 20.3 (HO-C-*C*H_3).

ESI-HRMS: *m/z* calcd for $C_{21}H_{24}O_5S$ $[M+Na]^+$ 411.1237, found 411.1217.

***p*-Methylphenyl 2-*O*-benzyl-4,6-*O*-benzylidene-3-*C*-methyl-thio-α-D-mannopyranoside 44:**

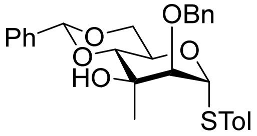

To a mixture of compound **43** (0.16 g, 0.41 mmol, 1 equiv), TBAB (7 mg, 0.02 mmol, 0.05 equiv) and benzyl bromide (0.11 mL, 0.92 mmol, 2.2 equiv) in DCM (2.7 mL, 0.15 M), was added a 10% solution of NaOH (2.7 mL, v/v:1/1 with DCM) at room temperature. The reaction mixture was stirred 36 h, diluted in DCM and washed with brine, the organic layer was dried over anhydrous Na_2SO_4 and concentrated *in vacuo*. The residue was purified by flash column chromatography by gradient elution of hexane: EtOAc (5% EtOAc in hexane to 25% EtOAc/hexane) to afford compound **44** (0.15 g, 0.32 mmol, 78%) as a white foam.

$[\alpha]_D^{22}$ +99.2 (*c* 0.5, $CHCl_3$).

^{1}H NMR (600 MHz, $CDCl_3$) δ 7.50 (dd, *J* = 7.3, 2.0 Hz, 2H, H_o-Ph), 7.38 – 7.31 (m, 10H, H_p-, H_m-Ph; H_o-, H_m-, H_p-OBn; H_o-STol), 7.14 (d, *J* = 7.8 Hz, 2H, H_m-STol), 5.57 (s, 1H, benzylidene-CH), 5.50 (d, *J* = 1.3 Hz, 1H, H-1), 4.72 (d, *J* = 11.3 Hz, 1H, OCH_2Ph), 4.60 (d, *J* = 11.4 Hz, 1H, OCH_2Ph), 4.28 – 4.24 (m, 2H, H-6a; H-5), 3.86 (d, *J* = 9.1 Hz, 1H, H-4), 3.80 – 3.76 (m, H-6b), 3.74 (d, *J* = 1.3 Hz, 1H, H-2), 3.09 (br. s, 1H, OH), 2.35 (s, 3H, CH_3-STol), 1.60 (s, 3H, HO-C-C*H*$_3$).

^{13}C{^{1}H} NMR (151 MHz, $CDCl_3$) δ 138.2 (C_p-STol), 137.6 (C_i-Ph), 136.9 (C_i-OBn), 132.4 (C_o-STol), 131.1 (C_i-STol), 130.1 (C_m-STol), 129.1 (C_p-Ph), 128.8 (C_m-Ph), 128.4 (C_p-OBn), 128.3 (C_m-OBn), 128.3 (C_o-OBn), 126.5 (C_o-Ph), 102.1 (benzylidene-CH), 86.6 ($^1J_{CH}$ = 165.0 Hz; C-1), 85.4 (C-2), 82.4 (C-4), 73.5 (OCH_2Ph), 70.8 (C-3), 68.9 (C-6), 63.4 (C-5), 21.2 (CH_3-STol) 18.9 (HO-C-*C*H_3).

ESI-HRMS: *m/z* calcd for $C_{28}H_{30}O_5S$ $[M+Na]^+$ 501.1706, found 501.1701.

_p_-Methylphenyl 3-_O_-(benzoyl-α-^{13}C)-2-_O_-benzyl-4,6-_O_-benzylidene-3-_C_-methyl-thio-α-D-mannopyranoside ^{13}C-45:

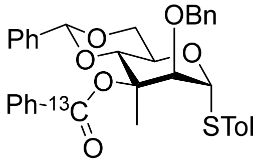

Prepared from compound **44** (40 mg, 0.083 mmol, 1 equiv) with the following quantities of reagents, temperature and time of reaction according to General Procedure GP1.

i) Benzoic acid-α-^{13}C (102 mg, 0.828 mmol, 10 equiv) and CDI (134 mg, 0.826 mmol, 10 equiv) in THF (1.5 mL) at room temperature.

ii) DBU (25 µL, 0.166 mmol, 2 equiv) was added at the beginning of the reaction followed by an equal amount after 24 h.

Compound **^{13}C-45** (45 mg, 0.077 mmol, 93%) was obtained as a white foam.

$[\alpha]_D^{22}$ +29.3 (*c* 1.0, $CHCl_3$).

^{1}H NMR (600 MHz, $CDCl_3$) δ 8.03 – 8.01 (m, 2H, H_o-OBz), 7.57 – 7.53 (m, 3H, H_p-OBz, H_o-Ph), 7.43 – 7.38 (m, 5H, H_m-OBz; H_o-OBn; H_p-Ph), 7.35 – 7.33 (m, 2H, H_o-STol), 7.18 – 7.10 (m, 7H, H_m-Ph; H_p-, H_m-OBn; H_m-STol), 5.73 (s, 1H, benzylidene-CH), 5.50 (d, *J* = 1.2 Hz, 1H, H-1), 4.95 (d, *J* = 1.2 Hz, 1H, H-2), 4.55 (d, *J* = 10.9 Hz, 1H, OCH_2Ph), 4.47 (d, *J* = 10.9 Hz, 1H, OCH_2Ph), 4.43 (td, *J* = 9.9, 5.0 Hz, 1H, H-5), 4.34 (d, *J* = 9.9 Hz, 1H, H-4), 4.31 (dd, *J* = 10.4, 5.0 Hz, 1H, H-6a), 3.88 (t, *J* = 10.2 Hz, 1H, H-6b), 2.36 (s, 3H, CH_3-STol), 2.06 (s, 3H, HO-C-CH_3).

^{13}C{^{1}H} NMR (151 MHz, $CDCl_3$) δ 161.1 (^{13}C=O),138.2 (C_p-STol), 137.7 (C_i-Ph), 137.4 (C_i-OBn), 132.9 (C_p-OBz), 132.5 (C_o-STol), 131.3 (d, *J* = 74.6 Hz, C_i-OBz), 131.2 (C_i-STol), 130.0 (C_m-STol), 129.9 (d, *J* = 2.3 Hz, C_o-OBz), 129.2 (C_p-Ph), 128.4 (C_m-Ph), 128.4 (C_m-OBz), 128.3 (C_m-OBn), 128.3 (C_o-OBn), 127.9 (C_p-OBn), 126.3 (C_o-Ph), 101.8 (benzylidene-CH), 86.6 (C-1), 82.5 (C-2), 81.1 (d, *J* = 2.7 Hz, C-3), 80.2 (d, *J* = 3.7 Hz, C-4), 73.3 (OCH_2Ph), 69.0 (C-6), 62.4 (C-5), 21.3 (CH_3-STol), 17.2 (BzO-C-CH_3).

ESI-HRMS: *m/z* calcd for $C_{34}{}^{13}CH_{34}O_6S$ $[M+Na]^+$ 606.2002, found 606.2000.

_p_-Methylphenyl 3-_O_-(benzoyl-α-^{13}C)-2-_O_-benzyl-4,6-_O_-benzylidene-3-_C_-methyl-thio-α-D-mannopyranoside _S_-oxide ^{13}C-46:

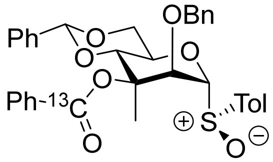

Compound **^{13}C-45** (40 mg, 0.068 mmol) in DCM was treated with *m*CPBA (18 mg, 0.080 mmol) according to General Procedure GP2 to afford compound **^{13}C-46** (37 mg, 0.062 mmol, 90%) as a white foam.

$[\alpha]_D^{22}$ -47.0 (*c* 0.1, $CHCl_3$).

^{1}H NMR (600 MHz, CDCl$_3$) δ 8.03 – 8.01 (m, 2H, H_o-OBz), 7.58 – 7.52 (m, 5H, H_m-S(O)Tol; H_p-OBz; H_o-Ph), 7.42 – 7.36 (m, 7H, H_m-OBz; H_o-OBn; H_p-Ph; H_o-S(O)Tol), 7.16 – 7.14 (m, 1H, H_p-OBn), 7.12 – 7.09 (m, 4H, H_o-Ph; H_m-OBn), 5.70 (s, 1H, benzylidene-CH), 5.36 (d, *J* = 1.2 Hz, 1H, H-2), 4.54 – 4.50 (m, 2H, OCH_2Ph), 4.44 (s, 1H, H-1), 4.36 (d, *J* = 9.9 Hz, 1H, H-4), 4.19 (dd, *J* = 10.5, 5.0 Hz, 1H, H-6a), 4.09 (td, *J* = 9.9, 5.0 Hz, 1H, H-5), 3.71 (t, *J* = 10.1 Hz, 1H, H-6b), 2.45 (s, 3H, CH_3-STol), 2.18 (s, 3H, HO-C-C*H*$_3$).

^{13}C{^{1}H} NMR (151 MHz, CDCl$_3$) δ 165.8 (^{13}C=O), 142.7 (C_p-S(O)Tol), 139.1 (C_i-S(O)Tol), 137.3 (C_i-Ph), 137.1 (C_i-OBn), 133.0 (C_p-OBz), 131.2 (d, *J* = 74.6 Hz, C_i-OBz), 130.2 (C_o-S(O)Tol), 129.9 (d, *J* = 2.3 Hz, C_o-OBz), 129.3 (C_p-Ph), 128.5 (C_m-Ph), 128.4 (C_m-OBn), 128.4 (d, *J* = 4.5 Hz, C_m-OBz), 128.3 (C_o-OBn), 127.9 (C_p-OBn), 126.2 (C_o-Ph), 125.0 (C_m-S(O)Tol), 101.9 (benzylidene-CH), 96.1 (C-1), 80.2 (d, *J* = 2.7 Hz, C-3), 79.0 (d, *J* = 3.5 Hz, C-4), 77.0 (C-2), 73.4 (OCH_2Ph), 68.6 (C-6), 66.9 (C-5), 21.7 (CH_3-S(O)Tol), 16.8 (BzO-C-C*H*$_3$).

ESI-HRMS: *m/z* calcd for $C_{34}{}^{13}CH_{34}O_7S$ $[M+Na]^+$ 622.1951, found 622.1956.

***p*-Methylphenyl 3-*O*-benzoyl-2-*O*-benzyl-4,6-*O*-benzylidene-thio-α-D-mannopyranoside 48:**

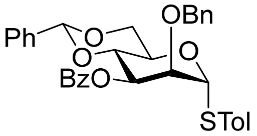

Compound **48** was prepared according to previously reported literature protocol.[2]

$[\alpha]_D^{22}$ +59.4 (*c* 1.0, CHCl$_3$).

The spectral data are consistent with those reported in the literature.[3]

^{1}H NMR (600 MHz, CDCl$_3$) δ 8.11– 8.10 (m, 2H, H_o-OBz), 7.62 – 7.59 (m, 1H, H_p-OBz), 7.51 – 7.46 (m, 4H, H_o-Ph; H_m-OBz), 7.40 – 7.39 (m, 2H, H_o-STol), 7.37 – 7.33 (m, 3H, H_p-, H_m-Ph), 7.29 – 7.25 (m, 2H, H_o-OBn), 7.24 – 7.15 (m, 5H, H_p-, H_m-OBn; H_m-STol), 5.67 (s, 1H, benzylidene-CH), 5.60 (dd, *J* = 10.3, 3.4 Hz, 1H, H-3), 5.55 (d, *J* = 1.4 Hz, 1H, H-1), 4.68 (d, *J* = 11.9 Hz, 1H, OCH_2Ph), 4.57 (d, *J* = 11.9 Hz, 1H, OCH_2Ph), 4.53 (td, *J* = 9.7, 4.7 Hz, 1H, H-5), 4.46 (t, *J* = 9.9 Hz, 1H, H-4), 4.39 (dd, *J* = 3.4, 1.4 Hz, 1H, H-2), 4.30 (dd, *J* = 10.2, 4.7 Hz, 1H, H-6a), 3.95 (t, *J* = 10.2 Hz, 1H, H-6b), 2.38 (s, 3H, CH_3-STol).

^{13}C{^{1}H} NMR (151 MHz, CDCl$_3$) δ 165.9 (C=O), 138.2 (C_p-STol), 137.4 (C_i-Ph), 137.3 (C_i-OBn), 133.3 (C_p-OBz), 132.6 (C_o-STol), 130.1 (C_m-STol), 130.0 (C_o-OBz), 130.0 (C_i-STol), 129.9 (C_i-OBz), 129.1 (C_p-Ph), 128.5 (C_m-OBz), 128.5 (C_m-Ph), 128.3 (C_m-OBn), 128.1 (C_p-, C_o-OBn), 126.3 (C_o-Ph), 101.9 (benzylidene-CH), 87.0 (C-1), 77.8 (C-2), 76.5 (C-4), 73.2 (OCH_2Ph), 71.2 (C-3), 68.7 (C-6), 65.4 (C-5), 21.3 (CH_3-STol).

ESI-HRMS: *m/z* calcd for $C_{34}H_{32}O_6S$ $[M+Na]^+$ 591.1812, found 591.1801.

***p*-Methylphenyl 3-*O*-(benzoyl-α-^{13}C)-2-*O*-benzyl-4,6-*O*-benzylidene-thio-α-D-mannopyranoside ^{13}C-48:**

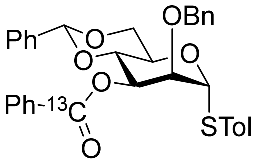

Prepared from compound **47** (220 mg, 0.473 mmol, 1 equiv) with the following quantities of reagents, temperature and time of reaction according to General Procedure GP1.

i) Benzoic acid-α-^{13}C (300 mg, 2.436 mmol, 5 equiv) and CDI (395 mg, 2.436 mmol, 5 equiv) in THF (4 mL) at room temperature.

ii) DBU (142 µL, 0.949 mmol, 2 equiv) was added at the beginning of the reaction followed by an equal amount after 12 h.

Compound **^{13}C-48** (249 mg, 0.437 mmol, 92%) was obtained as a white foam.

$[\alpha]_D^{22}$ +59.6 (*c* 1.0, $CHCl_3$).

^{1}H NMR (600 MHz, $CDCl_3$) δ 8.10 (ddd, *J* = 8.4, 4.1, 1.4 Hz, 2H, H_o-OBz), 7.62 – 7.59 (m, 1H, H_p-OBz), 7.51 – 7.47 (m, 4H, H_o-Ph; H_m-OBz), 7.40 – 7.39 (m, 2H, H_o-STol), 7.38 – 7.33 (m, 3H, H_p-, H_m-Ph), 7.79 – 7.26 (m, 2H, H_o-OBn), 7.24 – 7.17 (m, 5H, H_p-, H_m-OBn; H_m-STol), 5.67 (s, 1H, benzylidene-CH), 5.59 (dt, *J* = 10.3, 3.2 Hz, 1H, H-3), 5.55 (d, *J* = 1.4 Hz, 1H, H-1), 4.69 (d, *J* = 11.9 Hz, 1H, OCH_2Ph), 4.56 (d, *J* = 11.9 Hz, 1H, O*C*H_2Ph), 4.53 (td, *J* = 9.7, 4.7 Hz, 1H, H-5), 4.46 (t, *J* = 9.9 Hz, 1H, H-4), 4.39 (dd, *J* = 3.5, 1.4 Hz, 1H, H-2), 4.30 (dd, *J* = 10.2, 4.7 Hz, 1H, H-6a), 3.96 (t, *J* = 10.2 Hz, 1H, H-6b), 2.38 (s, 3H, CH_3-STol).

^{13}C{^{1}H} NMR (151 MHz, $CDCl_3$) δ 165.9 (^{13}C=O), 138.2 (C_p-STol), 137.3 (C_i-Ph), 137.2 (C_i-OBn), 133.3 (C_p-OBz), 132.6 (C_o-STol), 130.1 (C_m-STol), 130.0 (d, *J* = 2.6 Hz, C_o-OBz), 129.9 (d, J = 75.7, C_i-OBz), 129.9 (C_i-STol), 129.1 (C_p-Ph), 128.5 (C_m-Ph), 128.5 (d, *J* = 4.5 Hz, C_m-OBz), 128.3 (C_m-OBn), 128.1 (C_p-, C_o-OBn), 126.3 (C_o-Ph), 101.9 (benzylidene-*C*H), 87.0 (C-1), 77.7 (C-2), 76.4 (d, *J* = 2.3 Hz, C-4), 73.2 (O*C*H_2Ph), 71.2 (d, *J* = 2.2 Hz, C-3), 68.6 (C-6), 65.4 (C-5), 21.3 (*C*H_3-STol).

ESI-HRMS: *m/z* calcd for $C_{33}{}^{13}CH_{32}O_6S$ $[M+Na]^+$ 592.1845, found 592.1818.

***p*-Methylphenyl 3-*O*-benzoyl-2-*O*-benzyl-4,6-*O*-benzylidene-thio-α-D-mannopyranoside *S*-oxide 49:**

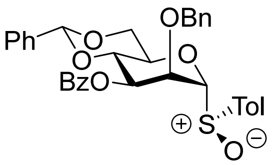

Compound **48** (330 mg, 0.580 mmol) in DCM was treated with *m*CPBA (156 mg, 0.696 mmol) according to General Procedure GP2 to afford compound **49** (306 mg, 0.523 mmol, 90%) as a white foam.

$[\alpha]_D^{22}$ +59.4 (*c* 1.0, $CHCl_3$).

^{1}H NMR (600 MHz, $CDCl_3$) δ 8.06 (d, *J* = 7.8 Hz, 2H, H_o-OBz), 7.59 – 7.56 (m, 3H, H_m-S(O)Tol; H_p-OBz), 7.48 – 7.43 (m, 4H, H_m-OBz; H_o-Ph), 7.38 (d, *J* = 7.9 Hz, 2H, H_o-S(O)Tol), 7.33 – 7.30 (m, 3H, H_p-, H_m-Ph), 7.21 – 7.15 (m, 1H, H_p-OBn), 7.13 – 7.08 (m, 4H, H_o-, H_m-OBn), 5.88 (dd, *J* = 10.5, 3.8 Hz, 1H, H-3), 5.62 (s, 1H, benzylidene-CH), 4.66 (d, *J* = 3.8 Hz, 1H, H-2), 4.55 – 4.49 (m, 2H, H-1; OCH_2Ph), 4.43 (t, *J* = 9.8 Hz, 1H, H-4), 4.38 (d, *J* = 11.8 Hz, 1H, O*C*H_2Ph), 4.32 – 4.25 (m, 2H, H-5; H-6a), 3.79 (t, *J* = 9.9 Hz, 1H, H-6b), 2.46 (s, 3H, CH_3-S(O)Tol).

^{13}C{^{1}H} NMR (151 MHz, $CDCl_3$) δ 165.6 (C=O), 142.5 (C_p-S(O)Tol), 137.9 (C_i-S(O)Tol), 137.1 (C_i-Ph), 136.8 (C_i-OBn), 133.2 (C_p-OBz), 130.4 (C_o-S(O)Tol), 130.0 (C_o-OBz), 129.9 (C_i-OBz), 129.2 (C_p-Ph), 128.4 (C_m-Ph), 128.4 (C_m-OBz), 128.3 (C_m-OBn), 128.2 (C_o-OBn), 128.1 (C_p-OBn), 126.3 (C_o-Ph), 124.7 (C_m-S(O)Tol), 101.9 (benzylidene-CH), 97.3 (C-1), 75.7 (C-4), 73.3 (OCH_2Ph), 72.7 (C-2), 71.1 (C-3), 70.0 (C-5), 68.4 (C-6), 21.7 (CH_3-S(O)Tol).

ESI-HRMS: *m/z* calcd for $C_{34}H_{32}O_7S$ $[M+Na]^+$ 607.1761, found 607.1738.

***p*-Methylphenyl 3-*O*-(benzoyl-α-^{13}C)-2-*O*-benzyl-4,6-*O*-benzylidene-thio-α-D-mannopyranoside *S*-oxide ^{13}C-49:**

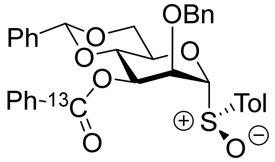

Compound **^{13}C-48** (135 mg, 0.237 mmol) in DCM was treated with *m*CPBA (64 mg, 0.285 mmol) according to General Procedure GP2 to afford compound **^{13}C-49** (123 mg, 0.210 mmol, 89%) as a white foam.

$[\alpha]_D^{22}$ +27.0 (*c* 0.5, $CHCl_3$).

^{1}H NMR (600 MHz, $CDCl_3$) δ 8.06 – 8.05 (m, 2H, H_o-OBz), 7.58 – 7.56 (m, 3H, H_m-S(O)Tol; H_p-OBz), 7.45 – 7.43 (m, 4H, H_m-OBz; H_o-Ph), 7.38 (d, *J* = 7.9 Hz, 2H, H_o-S(O)Tol), 7.35 – 7.30 (m, 3H, H_p-, H_m-Ph), 7.19 – 7.16 (m, 1H, H_p-OBn), 7.13 – 7.08 (m, 4H, H_o-, H_m-OBn), 5.87 (dt, *J* = 10.5, 3.4 Hz, 1H, H-3), 5.62 (s, 1H, benzylidene-CH), 4.65 (dd, *J* = 3.8, 1.2 Hz, 1H, H-2), 4.53 – 4.50 (m, 2H, H-1; OCH_2Ph), 4.43 (t, *J* = 9.9 Hz, 1H, H-4), 4.37 (d, *J* = 11.8 Hz, 1H, OCH_2Ph), 4.32 – 4.24 (m, 2H, H-5; H-6a), 3.78 (t, *J* = 9.9 Hz, 1H, H-6b), 2.46 (s, 3H, CH_3-S(O)Tol).

^{13}C{^{1}H} NMR (151 MHz, $CDCl_3$) δ 165.6 (^{13}C=O), 142.5 (C_p-S(O)Tol), 138.0 (C_i-S(O)Tol), 137.1 (C_i-Ph), 136.9 (C_i-OBn), 133.2 (C_p-OBz), 130.4 (C_o-S(O)Tol), 130.0 (d, *J* = 2.6 Hz, C_o-OBz), 129.9 (d, *J* = 75.6 Hz C_i-OBz), 129.2 (C_p-Ph), 128.5 (C_m-Ph), 128.4 (d, *J* = 4.3 Hz, C_m-OBz), 128.4 (C_m-OBn), 128.2 (C_o-OBn), 128.1 (C_p-OBn), 126.3 (C_o-Ph), 124.8 (Co-S(O)Tol), 101.9 (benzylidene-CH), 97.4 (C-1), 75.7 (d, *J* = 2.2 Hz, C-4), 73.4 (OCH_2Ph), 72.7 (C-2), 71.1 (d, *J* = 2.2 Hz, C-3), 70.0 (C-5), 68.4 (C-6), 21.7 (CH_3-S(O)Tol).

ESI-HRMS: *m/z* calcd for $C_{33}{}^{13}CH_{32}O_7S$ $[M+Na]^+$ 608.1794, found 608.1783.

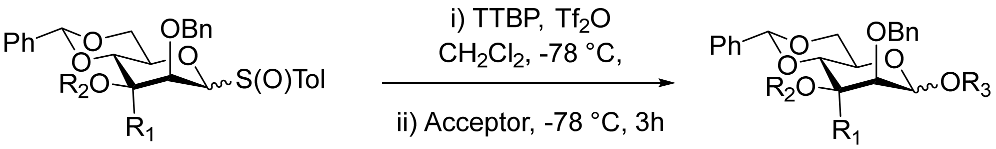

Table S2. Glycosylation with sulfoxide donors.

Entry	Donor		Acceptor	Product	Yield %[a] (α:β)
	R_1	R_2			
1	H	Ph	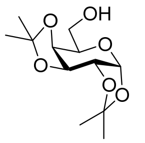 50	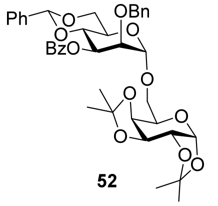 52	79 (α only)
2	Me	Ph		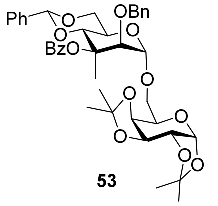 53	86 (α only)
3	Me	*p*NO_2Ph		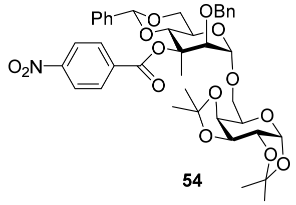 54	89 (α only)
4	H	Ph	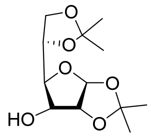 51	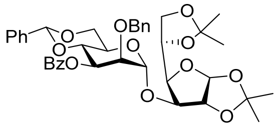 55	79 (α only)
5	Me	Ph		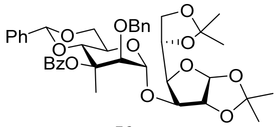 56	84 (α only)
6	Me	*p*NO_2Ph		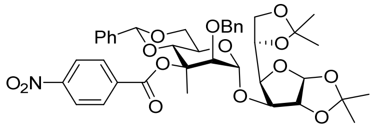 57	86 (α only)

General Procedure GP3: Acceptor (1 equiv; 0.025 M in DCM), Donor (1.63 equiv), TTBP (1.7 equiv). a-isolated yield

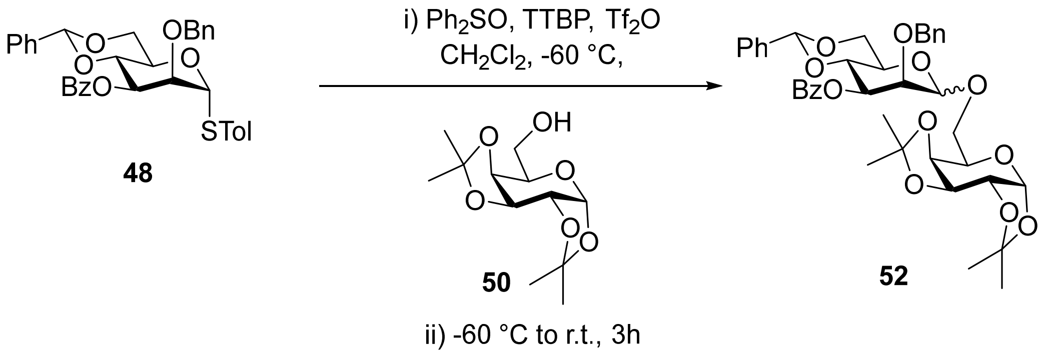

Table S3. Glycosylation with sulfide donor: influence of the quantity of Ph_2SO.

Entry	Ph_2SO (equiv)	Product	Yield %[a] (α:β)
1	1	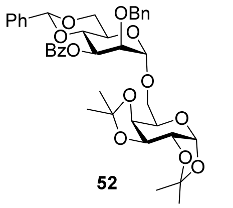	64 (α only)
2	3		93 (α only)
3	10		37 (α only)

General Procedure GP4: Donor (1 equiv; 0.022 M in DCM), Acceptor (1.5 equiv), TTBP (3.0 equiv). a-isolated yield

6-*O*-(3-*O*-Benzoyl-2-*O*-benzyl-4,6-*O*-benzylidene-α-D-mannopyranosyl)-1,2:3,4-di-*O*-isopropylidene-α-D-galactopyranose 52:

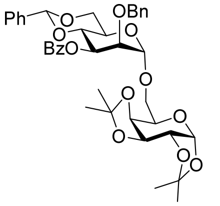

a) Prepared from sulfoxide donor **49** (36 mg, 0.062 mmol) and acceptor **50** (10 mg, 0.038 mmol), with the following quantities of reagents according to General Procedure GP3.
   TTBP (32 mg, 0.126 mmol), 4Å MS (38 mg), and Tf_2O (11 µL, 0.065 mmol).
   Compound **52** (21 mg, 0.030 mmol, 79%) was obtained as a white solid.

b) Prepared from sulfide donor **48** (50 mg, 0.088 mmol) and acceptor **50** (35 mg, 0.132 mmol), with the following quantities of reagents according to General Procedure GP4 and obtained with the yields shown in Table 4.
   TTBP (67 mg, 0.264 mmol), 4Å MS (88 mg), and Tf_2O (21 µL, 0.065 mmol).

Table S4. Quantity of phenyl sulfoxide and obtained yield of compound **52.**

Ph_2SO	Compound **52**
19 mg, 0.088 mmol, 1 equiv	40 mg, 0.057 mmol, 64%
56 mg, 0.264 mmol, 3 equiv	58 mg, 0.082 mmol, 93%
185 mg, 0.879 mmol, 10 equiv	23 mg, 0.033 mmol, 37%

$[\alpha]_D^{22}$ -30.8 (*c* 1.0, $CHCl_3$).

^{1}H NMR (600 MHz, $CDCl_3$) δ 8.07– 8.05 (m, 2H, H_o-OBz), 7.58 – 7.56 (m, 1H, H_p-OBz), 7.47 – 7.43 (m, 4H, H_o-Ph; H_m-OBz), 7.33 – 7.26 (m, 5H, H_p-, H_m-Ph; H_o-OBn), 7.21 – 7.19 (m, 3H, H_p-, H_m-OBn), 5.62 (s, 1H, benzylidene-CH), 5.56 (dd, *J* = 10.4, 3.5 Hz, 1H, H-3’), 5.53 (d, *J* = 5.0 Hz, 1H, H-1), 5.00 (d, *J* = 1.6 Hz, 1H, H-1’), 4.68 – 4.61 (m, 3H, OCH_2Ph; H-3), 4.36 – 4.28 (m, 4H, H-6a’; H-4’; H-2; H-4), 4.12 (dd, *J* = 3.6, 1.6 Hz, 1H, H-2’), 4.05 – 4.01 (m, 2H, H-5’; H-5), 3.90 (t, *J* = 10.3 Hz, 1H, H-6b’), 3.84 (dd, *J* = 10.1, 6.1 Hz, 1H, H-6a), 3.73 (dd, *J* = 10.1, 7.6 Hz, 1H, H-6b), 1.57 (s, 3H, $C(CH_3)_2$), 1.46 (s, 3H, $C(CH_3)_2$), 1.36 (s, 3H, $C(CH_3)_2$), 1.34 (s, 3H, $C(CH_3)_2$).

^{13}C{^{1}H} NMR (151 MHz, $CDCl_3$) δ 166.0 (C=O), 137.7 (C_i-Ph), 137.5 (C_i-OBn), 133.2 (C_p-OBz), 130.1 (C_i-OBz), 130.0 (C_o-OBz), 129.0 (C_p-Ph), 128.5 (C_m-Ph), 128.5 (C_m-OBz), 128.3 (C_m-OBn), 128.0 (C_p-OBn), 127.9 (C_o-OBn), 126.3 (C_o-Ph), 109.5 (*C*$(CH_3)_2$), 108.9 (*C*$(CH_3)_2$), 101.8 (benzylidene-CH), 99.2 (C-1’), 96.5 (C-1), 76.5 (C-2’), 76.5 (C-4’), 73.7 (O*C*H_2Ph), 71.3 (C-3’), 70.9 (C-4), 70.8 (C-2), 70.8 (C-3), 69.0 (C-6’), 66.4 (C-6), 65.9 (C-5), 64.4 (C-5’), 26.3 (C(*C*$H_3)_2$), 26.2 (C(*C*$H_3)_2$), 25.1 (C(*C*$H_3)_2$), 24.7 (C(*C*$H_3)_2$).

ESI-HRMS: *m/z* calcd for $C_{39}H_{44}O_{12}$ $[M+Na]^+$ 727.2725, found 727.2695.

6-*O*-(3-*O*-Benzoyl-2-*O*-benzyl-4,6-*O*-benzylidene-3-*C*-methyl-α-D-mannopyranosyl)-1,2:3,4-di-*O*-isopropylidene-α-D-galactopyranose 53:

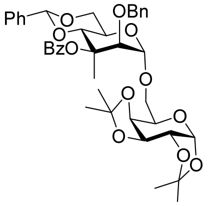

Prepared from sulfoxide donor **40** (38 mg, 0.063 mmol) and acceptor **50** (10 mg, 0.038 mmol), with the following quantities of reagents according to General Procedure GP3.

TTBP (32 mg, 0.126 mmol), 4Å MS (38 mg), and Tf_2O (11 µL, 0.065 mmol)

Compound **53** (23 mg, 0.033 mmol, 86%) was obtained as a white solid.

$[\alpha]_D^{22}$ -57.0 (*c* 0.4, $CHCl_3$).

^{1}H NMR (600 MHz, $CDCl_3$) δ 7.99 (dd, *J* = 8.3, 1.4 Hz, 2H, H_o-OBz), 7.55 – 7.51 (m, 3H, H_p-OBz; H_o-Ph), 7.41 – 7.31 (m, 5H, H_m-OBz; H_p-, H_m-Ph;), 7.16 – 7.13 (m, 1H, H_p-OBn), 7.13 – 7.11 (m, 4H, H_o- H_m-OBn), 5.70 (s, 1H, benzylidene-CH), 5.55 (d, *J* = 5.0 Hz, 1H, H-1), 4.98 (d, *J* = 1.4 Hz, 1H, H-1’), 4.66 – 4.64 (m, 2H, H-2’; H-3), 4.57 (d, *J* = 10.9 Hz, 1H, OCH_2Ph), 4.49 (d, *J* = 10.9 Hz, 1H, OCH_2Ph), 4.34 (dd, *J* = 5.0, 2.5 Hz, 1H, H-2), 4.31 (dd, *J* = 10.2, 5.0 Hz, 1H, H-6a’), 4.27 (dd, *J* = 7.9, 1.9 Hz, 1H, H-4), 4.24 (d, *J* = 9.8 Hz, 1H, H-4’), 4.06 – 4.01 (m, 2H, H-5’; H-5), 3.88 – 3.80 (m, 2H, H-6b’; H-6a), 3.65 (dd, *J* = 10.3, 5.7 Hz, 1H, H-6b), 1.92 (s, 3H, BzO-C-CH_3), 1.54 (s, 3H, $C(CH_3)_2$), 1.46 (s, 3H, $C(CH_3)_2$), 1.36 (s, 3H, $C(CH_3)_2$), 1.34 (s, 3H, $C(CH_3)_2$).

^{13}C{^{1}H} NMR (151 MHz, $CDCl_3$) δ 166.2 (C=O), 137.9 (C_i-Ph), 137.8 (C_i-OBn), 132.8 (C_p-OBz), 131.6 (C_i-OBz), 129.8 (C_o-OBz), 129.1 (C_p-Ph), 128.4 (C_m-Ph), 128.3 (C_m-OBz), 128.3 (C_m-OBn), 128.0 (C_p-OBn), 127.7 (C_o-OBn), 126.3 (C_o-Ph), 109.6 ($\mathit{C}(CH_3)_2$), 108.8 ($\mathit{C}(CH_3)_2$), 101.7 (benzylidene-CH), 98.2 (C-1’), 96.5 (C-1), 81.6 (C-3’), 80.3 (C-2’), 79.9 (C-4’), 73.6 (OCH_2Ph), 71.3 (C-4), 70.9 (C-3), 70.8 (C-2), 69.3 (C-6’), 66.7 (C-6), 66.4 (C-5), 61.8 (C-5’), 26.3 ($C(\mathit{C}H_3)_2$), 26.2 ($C(\mathit{C}H_3)_2$), 25.1 ($C(\mathit{C}H_3)_2$), 24.7 ($C(\mathit{C}H_3)_2$), 16.6 (BzO-C-$\mathit{C}H_3$).

ESI-HRMS: *m/z* calcd for $C_{40}H_{46}O_{12}$ $[M+Na]^+$ 741.2881, found 741.2858.

6-*O*-(2-*O*-Benzyl-4,6-*O*-benzylidene-3-*O*-*p*-nitrobenzoyl-3-*C*-methyl-α-D-mannopyranosyl)-1,2:3,4-di-*O*-isopropylidene-α-D-galactopyranose 54:

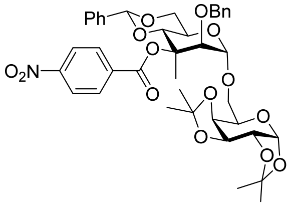

Prepared from sulfoxide donor **41** (40 mg, 0.062 mmol) and acceptor **50** (10 mg, 0.038 mmol), with the following quantities of reagents according to General Procedure GP3.

TTBP (32 mg, 0.126 mmol), 4Å MS (38 mg), and Tf_2O (11 µL, 0.065 mmol)

Compound **54** (26 mg, 0.034 mmol, 89%) was obtained as a white solid.

$[\alpha]_D^{22}$ -53.8 (*c* 1.0, $CHCl_3$).

^{1}H NMR (600 MHz, $CDCl_3$) δ 8.22 – 8.20 (m, 2H, H_m-PNB), 8.10 – 8.07 (m, 2H, H_o-PNB), 7.53 (dd, *J* = 7.9, 1.8 Hz, 2H, H_o-Ph), 7.41 – 7.37 (m, 3H, H_p-, H_m-Ph;), 7.16 – 7.13 (m, 1H, H_p-OBn), 7.11 – 7.09 (m, 4H, H_o-H_m-OBn), 5.70 (s, 1H, benzylidene-CH), 5.56 (d, *J* = 5.0 Hz, 1H, H-1), 5.03 (d, *J* = 1.3 Hz, 1H, H-1'), 4.66 (dd, *J* = 7.9, 2.5 Hz, 1H, H-3), 4.63 – 4.62 (m, 2H, H-2'; OCH_2Ph), 4.41 (d, *J* = 11.0 Hz, 1H, 1H, OCH_2Ph), 4.36 – 4.31 (m, 2H, H-2, H-6a'), 4.27 (dd, *J* = 7.9, 1.9 Hz, 1H, H-4), 4.25 (d, *J* = 9.8 Hz, 1H, H-4'), 4.08 – 4.03 (m, 2H, H-5'; H-5), 3.89 – 3.84 (m, 2H, H-6b'; H-6a), 3.68 (dd, *J* = 10.4, 5.3 Hz, 1H, H-6b), 1.93 (s, 3H, ArO-C-CH_3), 1.54 (s, 3H, $C(CH_3)_2$), 1.47 (s, 3H, $C(CH_3)_2$), 1.36 (s, 3H, $C(CH_3)_2$), 1.34 (s, 3H, $C(CH_3)_2$).

^{13}C{^{1}H} NMR (151 MHz, $CDCl_3$) δ 164.1 (C=O), 150.4 (C_p-PNB), 137.7 (C_i-Ph), 137.5 (C_i-OBn), 137.0 (C_i-PNB), 130.8 (C_o-PNB), 129.2 (C_p-Ph), 128.4 (C_m-Ph), 128.3 (C_m-OBn), 127.9 (C_p-OBn), 127.8 (C_o-OBn), 126.3 (C_o-Ph), 123.4 (C_m-PNB), 109.7 (*C*$(CH_3)_2$), 108.8 (*C*$(CH_3)_2$), 101.9 (benzylidene-CH), 97.7 (C-1'), 96.5 (C-1), 82.9 (C-3'), 80.3 (C-2'), 79.7 (C-4'), 73.5 (OCH_2Ph), 71.4 (C-4), 70.9 (C-2), 70.7 (C-3), 69.2 (C-6'), 66.7 (C-6), 66.4 (C-5), 61.7 (C-5'), 26.2 (C(*C*$H_3)_2$), 26.1 (C(*C*$H_3)_2$), 25.1 (C(*C*$H_3)_2$), 24.7 (C(*C*$H_3)_2$), 16.5 (ArO-C-*C*H_3).

ESI-HRMS: *m/z* calcd for $C_{40}H_{45}O_{14}N$ $[M+Na]^+$ 786.2732, found 786.2709.

3-*O*-(3-*O*-Benzoyl-2-*O*-benzyl-4,6-*O*-benzylidene-α-D-mannopyranosyl)-1,2:5,6-di-*O*-isopropylidene-α-D-glucofuranose 55:

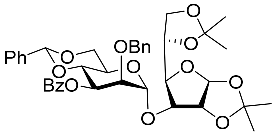

Prepared from sulfoxide donor **49** (36 mg, 0.062 mmol) and acceptor **51** (10 mg, 0.038 mmol), with the following quantities of reagents according to General Procedure GP3.

TTBP (32 mg, 0.126 mmol), 4Å MS (38 mg), and Tf_2O (11 µL, 0.065 mmol)

Compound **55** (21 mg, 0.030 mmol, 79%) was obtained as a white solid.

$[\alpha]_D^{22}$ -38.4 (*c* 1.0, $CHCl_3$).

^{1}H NMR (600 MHz, $CDCl_3$) δ 8.05 (dd, *J* = 8.2, 1.4 Hz, 2H, H_o-OBz), 7.59 – 7.56 (m, 1H, H_p-OBz), 7.46 – 7.44 (m, 4H, H_o-Ph; H_m-OBz), 7.34 – 7.30 (m, 3H, H_p-, H_m-Ph), 7.27 – 7.25 (m, 2H, H_o-OBn), 7.21 – 7.16 (m, 3H, H_p-, H_m-OBn), 5.93 (d, *J* = 3.5 Hz, 1H, H-1), 5.65 (s, 1H, benzylidene-CH), 5.53 (dd, *J* = 10.5, 3.5 Hz, 1H, H-3'), 5.32 (d, *J* = 1.5 Hz, 1H, H-1'), 4.70 (d, *J* = 11.9 Hz, 1H, OCH_2Ph), 4.62 (d, *J* = 3.5 Hz, 1H, H-2), 4.56 (d, *J* = 11.9 Hz, 1H, OCH_2Ph), 4.39 – 4.35 (m, 2H, H-6a'; H-4'), 4.34 (d, *J* = 2.8 Hz, 1H, H-3), 4.20 – 4.15 (m, 1H, H-4; H-6a), 4.12 (dd, *J* = 3.5, 1.5 Hz, 1H, H-2'), 4.10 – 4.04 (m, 2H, H-5; H-6b), 4.01 (td, *J* = 9.8, 4.6 Hz, 1H, H-5'), 3.94 (t, *J* = 10.3 Hz, 1H, H-6b'), 1.52 (s, 3H, $C(CH_3)_2$), 1.42 (s, 3H, $C(CH_3)_2$), 1.34 (s, 3H, $C(CH_3)_2$), 1.33 (s, 3H, $C(CH_3)_2$).

^{13}C{^{1}H} NMR (151 MHz, $CDCl_3$) δ 165.9 (C=O), 137.5 (C_i-Ph), 137.3 (C_i-OBn), 133.3 (C_p-OBz), 130.0 (C_o-, C_i-OBz), 129.1 (C_p-Ph), 128.5 (C_m-Ph), 128.5 (C_m-OBz), 128.4 (C_m-OBn), 128.1 (C_p-OBn), 127.9 (C_o-OBn), 126.1 (C_o-Ph), 112.3 (*C*$(CH_3)_2$), 109.6 (*C*$(CH_3)_2$), 105.5 (C-1), 101.7 (benzylidene-CH), 99.7 (C-1'), 84.4 (C-2), 81.7 (C-5), 80.6 (C-3), 76.4 (C-4'), 76.1 (C-2'), 73.5 (OCH_2Ph), 72.8 (C-4), 70.8 (C-3'), 68.9 (C-6'), 68.0 (C-6), 65.1 (C-5'), 27.1 (C(*C*$H_3)_2$), 27.0 (C(*C*$H_3)_2$), 26.4 (C(*C*$H_3)_2$), 25.7 (C(*C*$H_3)_2$).

ESI-HRMS: *m/z* calcd for $C_{39}H_{44}O_{12}$ $[M+Na]^+$ 727.2725, found 727.2700.

3-*O*-(3-*O*-Benzoyl-2-*O*-benzyl-4,6-*O*-benzylidene-3-*C*-methyl-α-D-mannopyranosyl)-1,2:5,6-di-*O*-isopropylidene-α-D-glucofuranose 56:

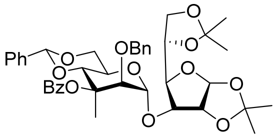

Prepared from sulfoxide donor **40** (38 mg, 0.063 mmol) and acceptor **51** (10 mg, 0.038 mmol), with the following quantities of reagents according to General Procedure GP3.

TTBP (32 mg, 0.126 mmol), 4Å MS (38 mg), and Tf_2O (11 µL, 0.065 mmol)

Compound **56** (23 mg, 0.032 mmol, 84%) was obtained as a white solid.

$[\alpha]_D^{22}$ -44.9 (*c* 1.0, $CHCl_3$).

^{1}H NMR (600 MHz, $CDCl_3$) δ 7.99 (dd, *J* = 7.9, 1.5 Hz, 2H, H_o-OBz), 7.56 – 7.52 (m, 3H, H_p-OBz; H_o-Ph), 7.41 – 7.36 (m, 5H; H_m-OBz; H_p-, H_m-Ph), 7.16 – 7.13 (m, 1H, H_p-OBn), 7.11 – 7.07 (m, 4H, H_o-, H_m-OBn), 5.91 (d, *J* = 3.6 Hz, 1H, H-1), 5.73 (s, 1H, benzylidene-CH), 5.32 (s, 1H, H-1'), 4.68 (s, 1H, H-2'), 4.61 – 4.58 (m, 2H, OCH_2Ph; H-2), 4.45 (d, *J* = 10.7 Hz, 1H, OCH_2Ph), 4.38 (dd, *J* = 10.4, 4.8 Hz, 1H, H-6a'), 4.31 – 4.27 (m, 3H, H-3; H-4; H-4'), 4.21 (dd, *J* = 8.8, 6.1 Hz, 1H, H-6a), 4.10 – 4.08 (m, 2H, H-5; H-6b), 4.00 (td, *J* = 9.9, 4.8 Hz, 1H, H-5'), 3.89 (t, *J* = 10.3 Hz, 1H, H-6b'), 1.89 (s, 3H, BzO-C-CH_3), 1.52 (s, 3H, $C(CH_3)_2$), 1.43 (s, 3H, $C(CH_3)_2$), 1.35 (s, 6H, 2 x $C(CH_3)_2$).

$^{13}C\{^1H\}$ NMR (151 MHz, $CDCl_3$) δ 166.1 (C=O), 137.6 (C_i-Ph), 137.5 (C_i-OBn), 132.9 (C_p-OBz), 131.4, C_i-OBz), 129.8 (C_o-OBz), 129.2 (C_p-Ph), 128.4 (C_m-Ph), 128.4 (C_m-OBz), 128.3 (C_m-OBn), 127.9 (C_o-OBn), 127.8 (C_p-OBn), 126.2 (C_o-Ph), 112.4 ($\mathit{C}(CH_3)_2$), 109.6 ($\mathit{C}(CH_3)_2$), 105.5 (C-1), 101.7 (benzylidene-CH), 99.4 (C-1'), 84.3 (C-2), 81.8 (C-5, C-3'), 80.9 (C-3), 80.0 (C-2'), 79.8 (C-4'), 73.5 (OCH_2Ph), 72.9 (C-4), 69.2 (C-6'), 68.0 (C-6), 62.5 (C-5'), 27.1 ($C(\mathit{C}H_3)_2$), 27.1 ($C(\mathit{C}H_3)_2$), 26.5 ($C(\mathit{C}H_3)_2$), 25.5 ($C(\mathit{C}H_3)_2$), 16.5 (BzO-C-$\mathit{C}H_3$).

ESI-HRMS: *m/z* calcd for $C_{40}H_{46}O_{12}$ $[M+Na]^+$ 741.2881, found 741.2858.

3-*O*-(2-*O*-Benzyl-4,6-*O*-benzylidene-3-*O*-*p*-nitrobenzoyl-3-*C*-methyl-α-D-mannopyranosyl)-1,2:5,6-di-*O*-isopropylidene-α-D-glucofuranose 57:

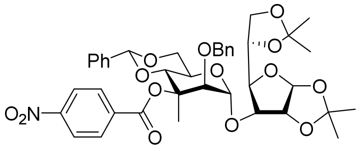

Prepared from sulfoxide donor **41** (40 mg, 0.062 mmol) and acceptor **51** (10 mg, 0.038 mmol), with the following quantities of reagents according to General Procedure GP3.

TTBP (32 mg, 0.126 mmol), 4Å MS (38 mg), and Tf_2O (11 µL, 0.065 mmol)

Compound **57** (25 mg, 0.033 mmol, 86%) was obtained as a white solid.

$[\alpha]_D^{22}$ -72.8 (*c* 1.0, $CHCl_3$).

^{1}H NMR (600 MHz, $CDCl_3$) δ 8.22 – 8.20 (m, 2H, H_m-PNB), 8.09 – 8.06 (m, 2H, H_o-PNB), 7.53 (dd, *J* = 7.8, 1.9 Hz, 2H, H_o-Ph), 7.43 – 7.37 (m, 3H, H_p-, H_m-Ph), 7.16 – 7.14 (m, 1H, H_p-OBn), 7.11 – 7.08 (m, 2H, H_o-OBn), 7.06 – 7.05 (m, 2H, H_m-OBn), 5.92 (d, *J* = 3.5 Hz, 1H, H-1), 5.73 (s, 1H, benzylidene-CH), 5.35 (d, *J* = 1.3 Hz, 1H, H-1'), 4.67 – 4.62 (m, 2H, OCH_2Ph; H-2'), 4.59 (d, *J* = 3.5 Hz, 1H, H-2), 4.40 (dd, *J* = 10.4, 4.9 Hz, 1H, H-6a'), 4.37 (d, *J* = 10.8 Hz, 1H, OCH_2Ph), 4.33 (d, *J* = 2.8 Hz, 1H, H-3), 4.31 – 4.27 (m, 2H, H-4; H-4'), 4.23 (dd, *J* = 8.8, 6.1 Hz, 1H, H-6a), 4.11 – 4.09 (m, 2H, H-5; H-6b), 44.00 (td, *J* = 9.9, 4.8 Hz, 1H, H-5'), 3.90 (t, *J* = 10.3 Hz, 1H, H-6b'), 1.90 (s, 3H, ArO-C-CH_3), 1.53 (s, 3H, $C(CH_3)_2$), 1.43 (s, 3H, $C(CH_3)_2$), 1.35 (s, 6H, 2 x $C(CH_3)_2$).

^{13}C{^{1}H} NMR (151 MHz, $CDCl_3$) δ 164.0 (C=O), 150.5 (C_p-PNB), 137.4 (C_i-Ph), 137.3 (C_i-OBn), 136.8 (C_i-PNB), 130.8 (C_o-PNB), 129.4 (C_p-Ph), 128.5 (C_m-Ph), 128.3 (C_m-OBn), 127.9 (C_p-OBn), 127.7 (C_o-OBn), 126.2 (C_o-Ph), 123.5 (C_m-PNB), 112.5 ($\mathit{C}(CH_3)_2$), 109.7 ($\mathit{C}(CH_3)_2$), 105.5 (C-1), 101.9 (benzylidene-CH), 99.2 (C-1'), 84.3 (C-2), 82.2 (C-3'), 81.8 (C-5), 81.0 (C-3), 79.9 (C-2'), 79.6 (C-4'), 73.4 (OCH_2Ph), 72.9 (C-4), 69.2 (C-6'), 68.1 (C-6), 62.5 (C-5'), 27.1 ($C(\mathit{C}H_3)_2$), 27.1 ($C(\mathit{C}H_3)_2$), 26.5 ($C(\mathit{C}H_3)_2$), 25.6 ($C(\mathit{C}H_3)_2$), 16.5 (ArO-C-$\mathit{C}H_3$).

ESI-HRMS: *m/z* calcd for $C_{40}H_{45}O_{14}N$ $[M+Na]^+$ 786.2732, found 786.2717.

References

(1) Yu, H. N.; Furukawa, J.-i.; Ikeda, T.; Wong, C.-H. *Org. Lett.* **2004**, *6*, 723-726.
(2) Crich, D.; Sharma, I. *J. Org. Chem.* **2010**, *75*, 8383-8391.
(3) Cendret, V.; François-Heude, M.; Méndez-Ardoy, A.; Moreau, V.; Fernández, J. M. G.; Djedaïni-Pilard, F. *Chem. Commun.* **2012**, *48*, 3733-3735.

^{1}H, ^{13}C{^{1}H}, DEPT, COSY, HSQC, HMBC NMR Spectra of Compounds

Figure S1. ^{1}H NMR (600 MHz, $CDCl_3$) spectrum of *p*-methylphenyl 4,6-*O*-benzylidene-3-*O*-*p*-methoxybenzyl-2-*O*-*tert*-butyldimethylsilyl-thio-β-D-mannopyranoside **32**:

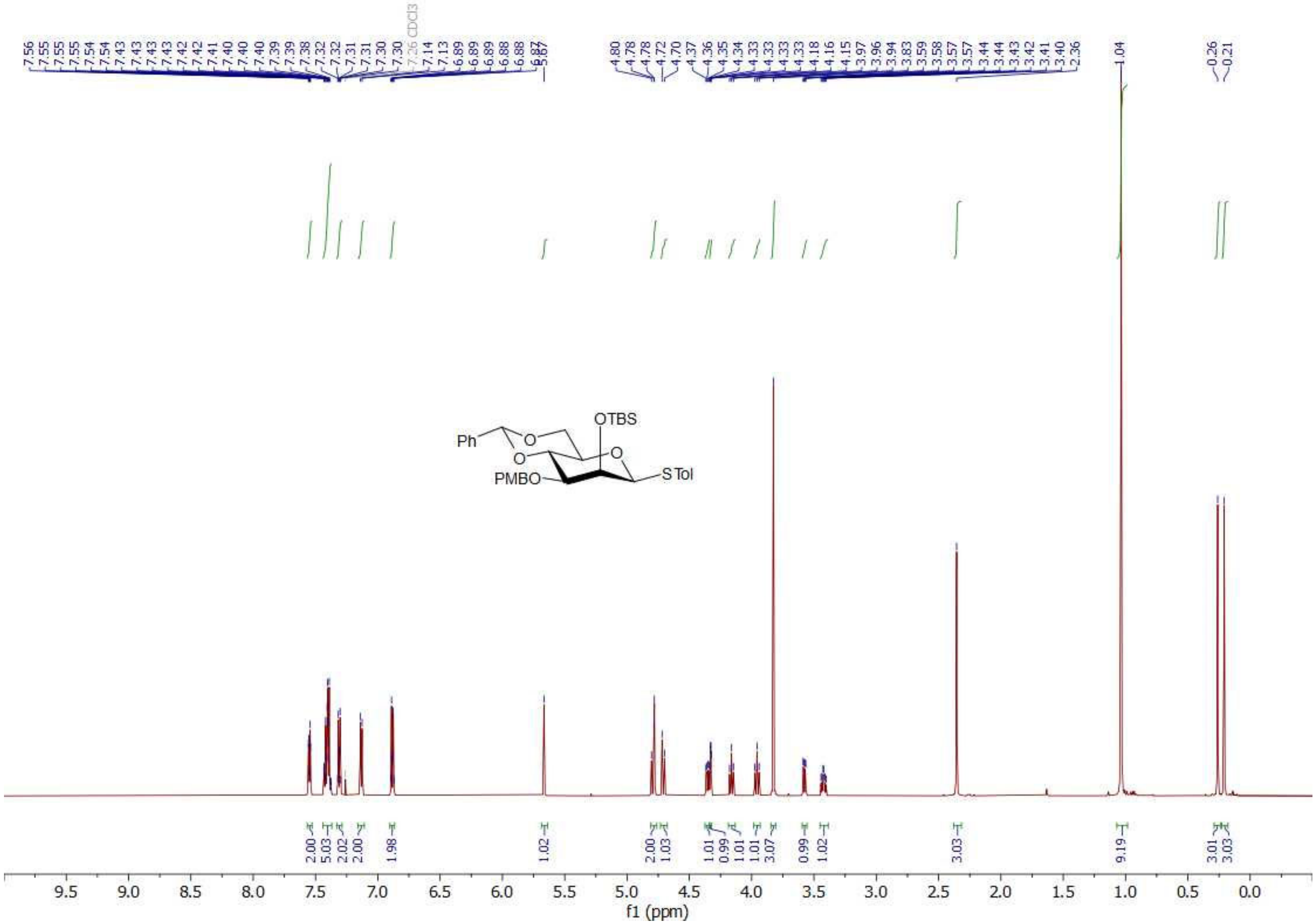

Figure S2. COSY NMR (600 MHz, $CDCl_3$) spectrum of *p*-methylphenyl 4,6-*O*-benzylidene-3-*O*-*p*-methoxybenzyl-2-*O*-*tert*-butyldimethylsilyl-thio-β-D-mannopyranoside **32**:

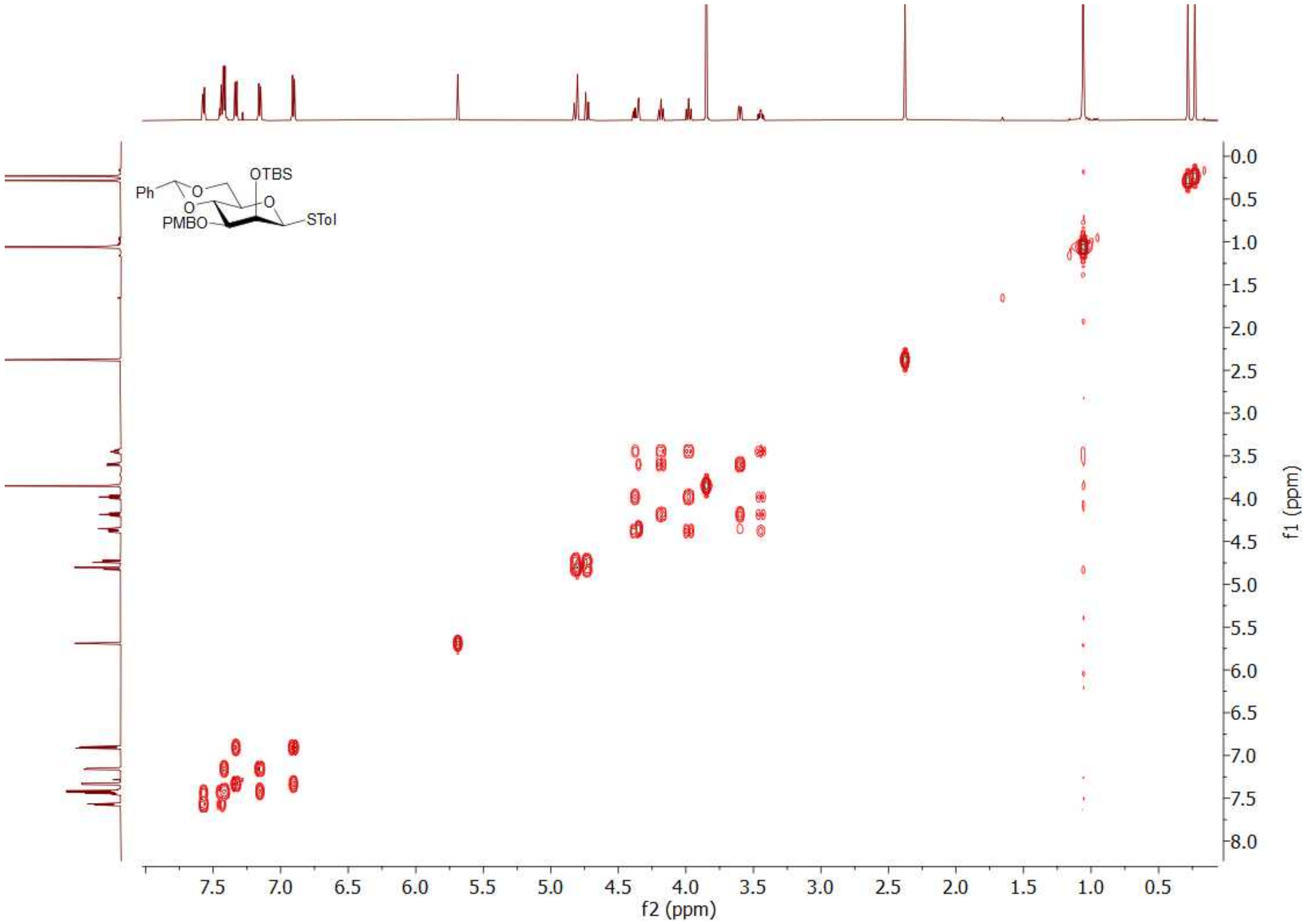

Figure S3. $^{13}C\{^{1}H\}$ NMR (151 MHz, CDCl3) spectrum of *p*-methylphenyl 4,6-*O*-benzylidene-3-*O*-*p*-methoxybenzyl-2-*O*-*tert*-butyldimethylsilyl-thio-β-D-mannopyranoside **32**:

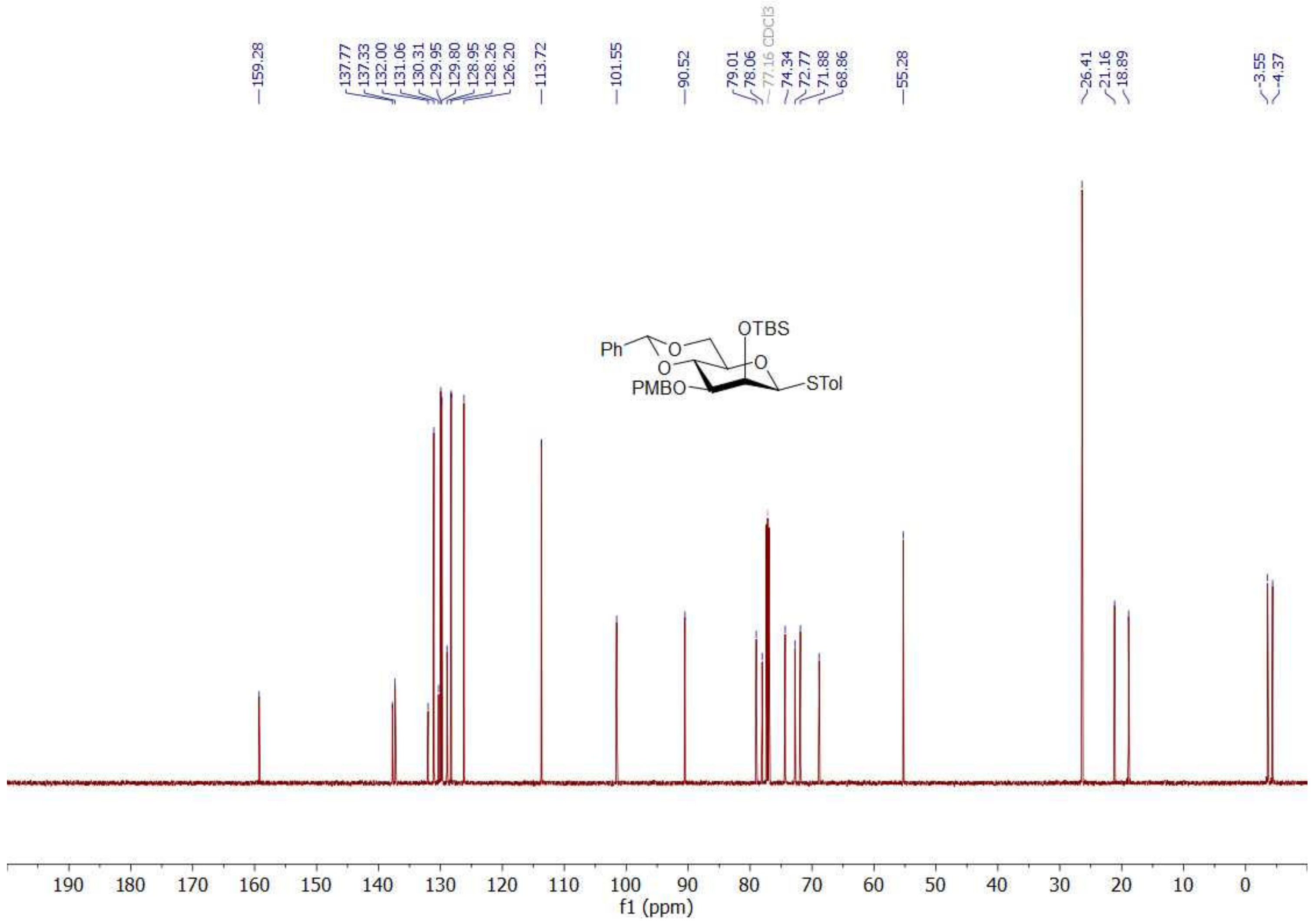

Figure S4. $^{13}C\{^1H\}$ DEPT NMR (151 MHz, $CDCl_3$) spectrum of *p*-methylphenyl 4,6-*O*-benzylidene-3-*O*-*p*-methoxybenzyl-2-*O*-*tert*-butyldimethylsilyl-thio-β-D-mannopyranoside **32**:

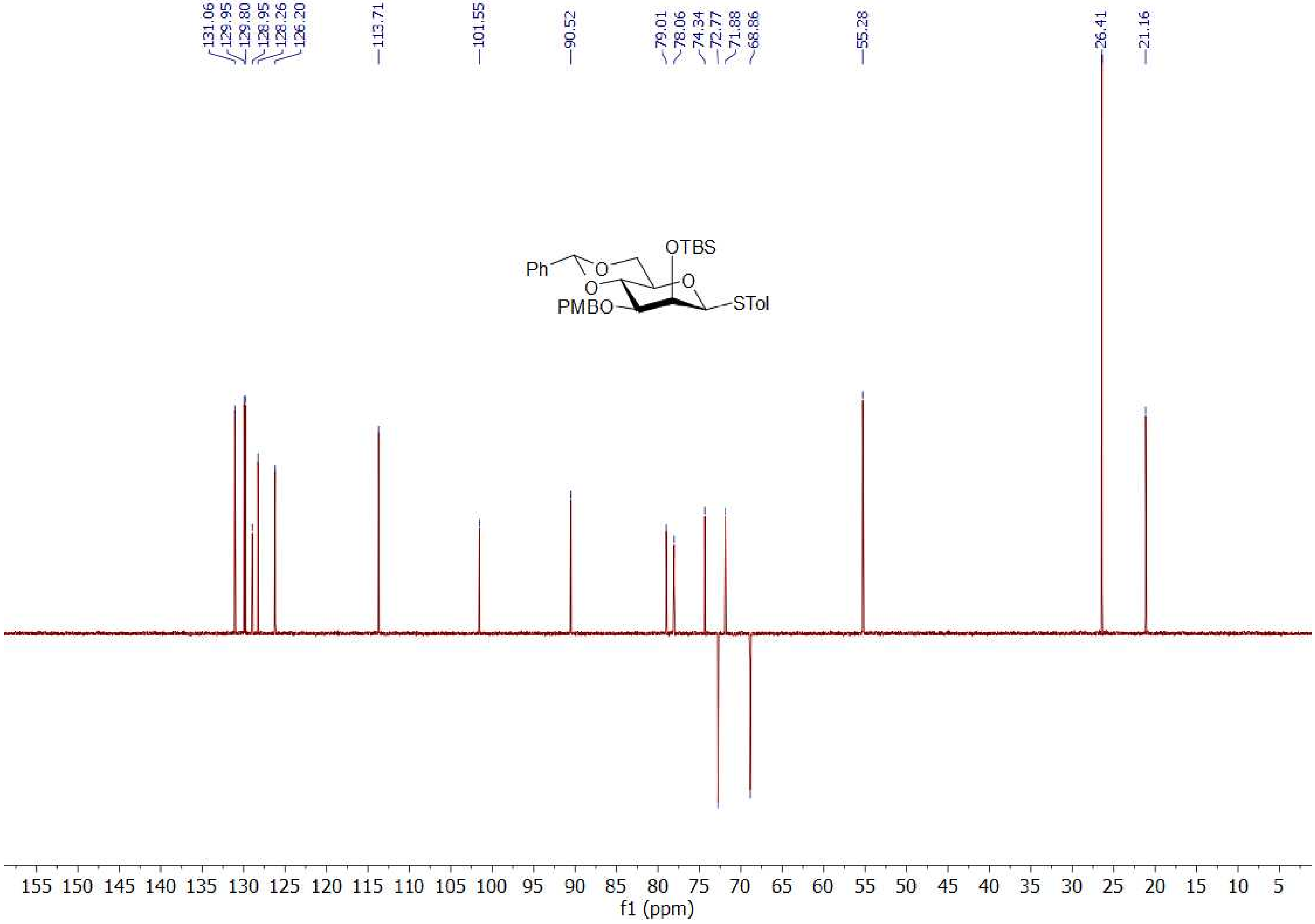

Figure S5. HSQC NMR (600 MHz, $CDCl_3$) spectrum of *p*-methylphenyl 4,6-*O*-benzylidene-3-*O*-*p*-methoxybenzyl-2-*O*-*tert*-butyldimethylsilyl-thio-β-D-mannopyranoside **32**:

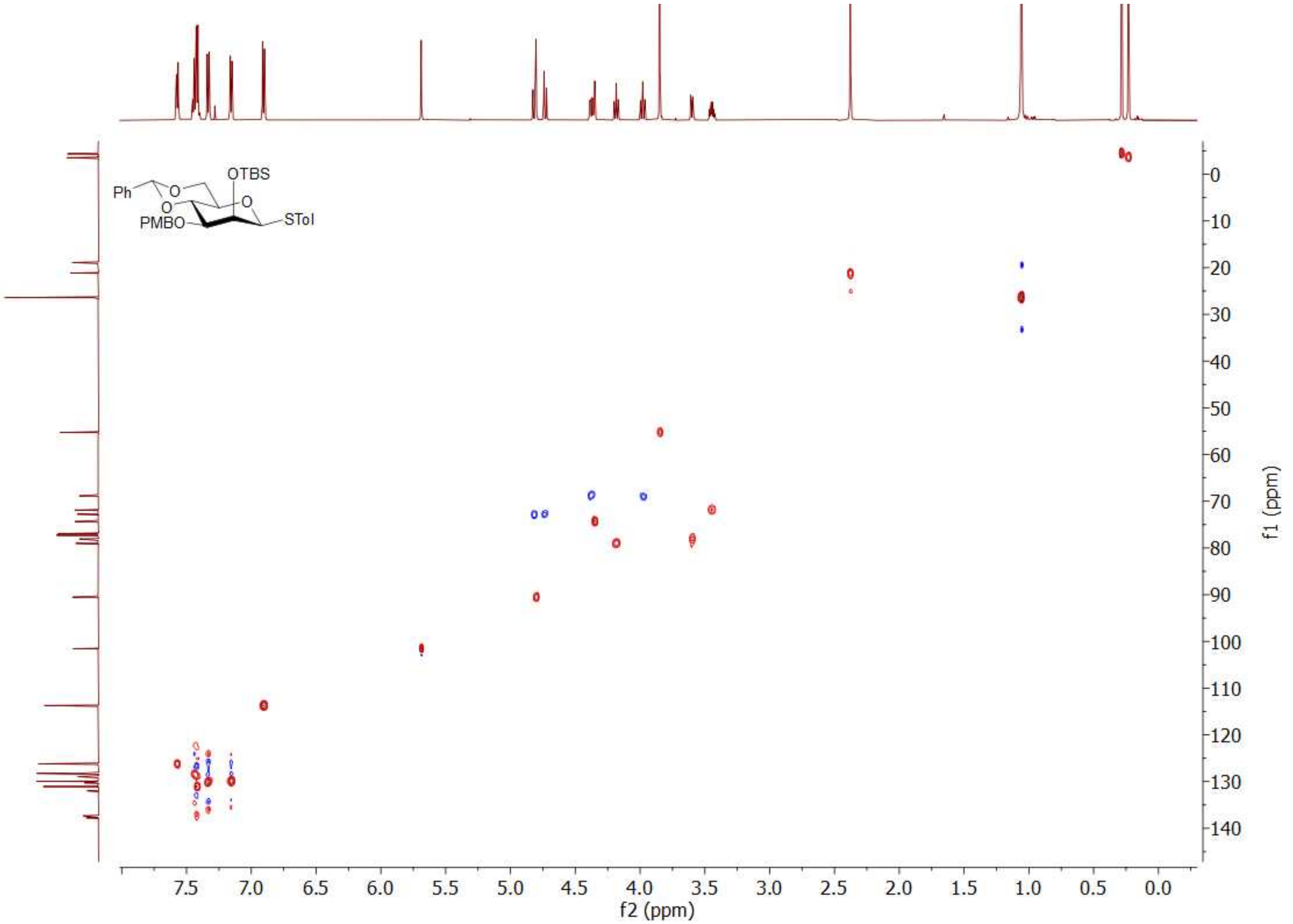

Figure S6. HMBC NMR (600 MHz, $CDCl_3$) spectrum of *p*-methylphenyl 4,6-*O*-benzylidene-3-*O*-*p*-methoxybenzyl-2-*O*-*tert*-butyldimethylsilyl-thio-β-D-mannopyranoside **32**:

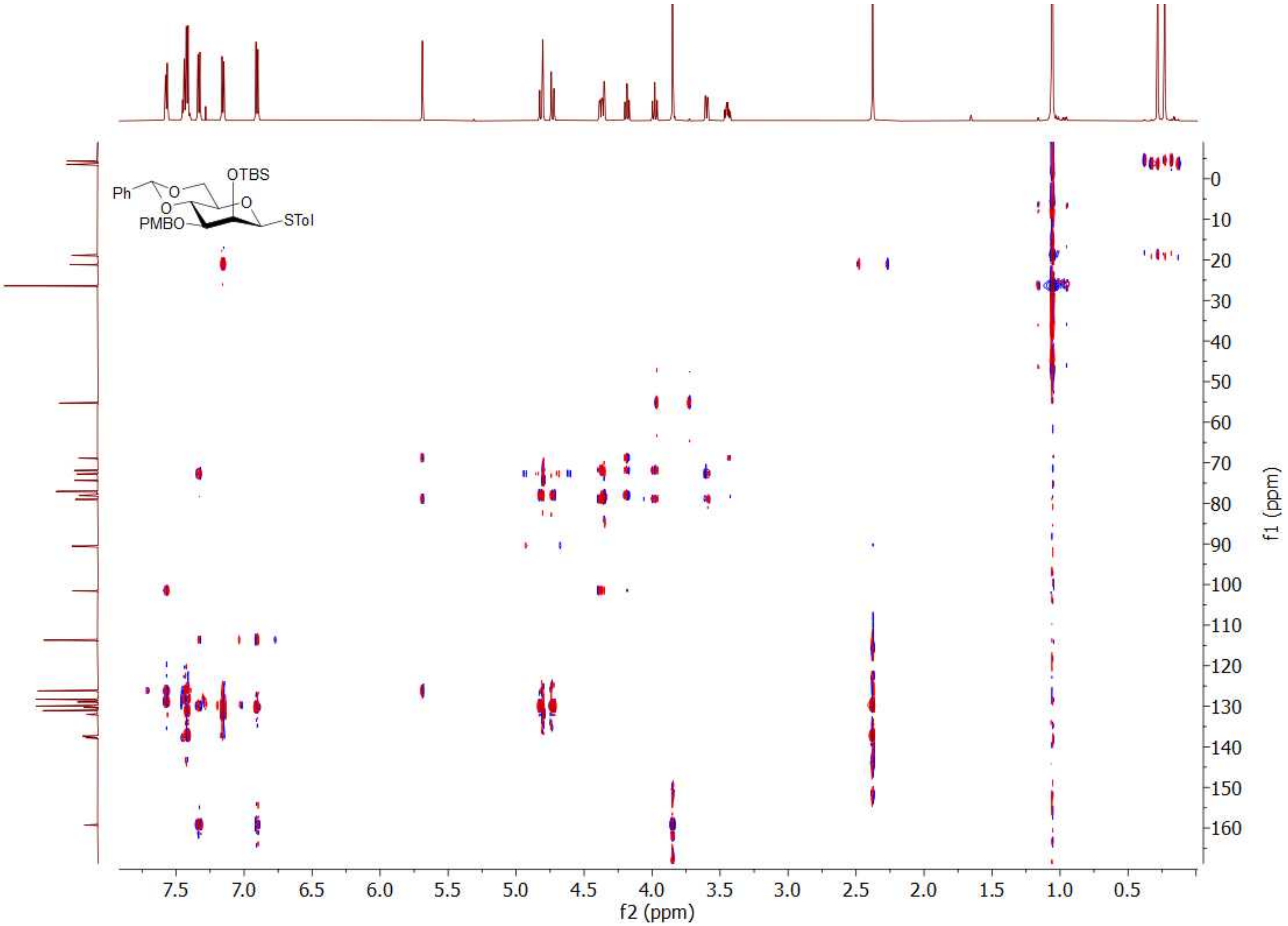

Figure S7. ^{1}H NMR (600 MHz, $CDCl_3$) spectrum of *p*-methylphenyl 4,6-*O*-benzylidene-2-*O*-*tert*-butyldimethylsilyl-thio-β-D-mannopyranoside **33**:

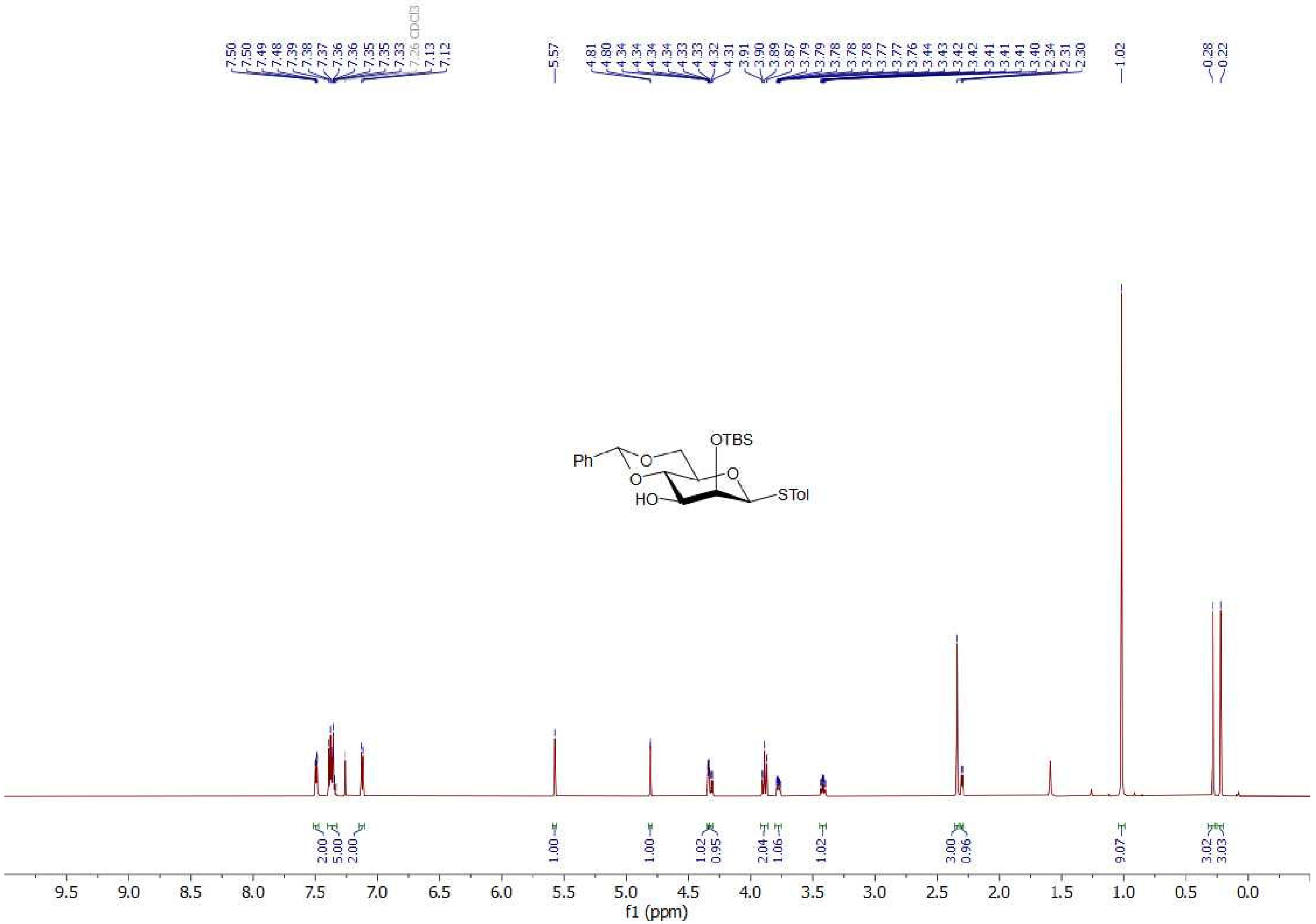

Figure S8. COSY NMR (600 MHz, $CDCl_3$) spectrum of *p*-methylphenyl 4,6-*O*-benzylidene-2-*O*-*tert*-butyldimethylsilyl-thio-β-D-mannopyranoside **33**:

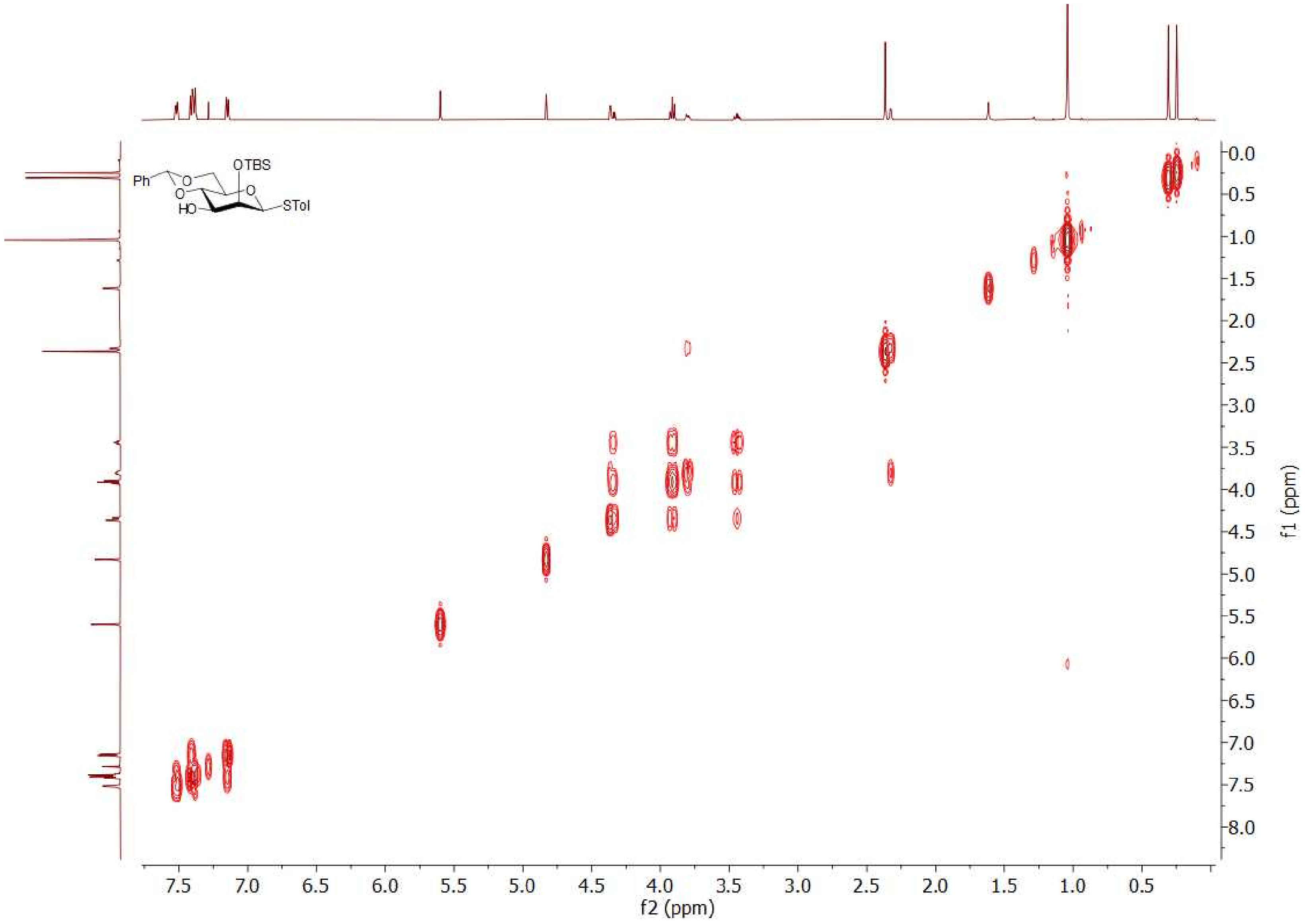

Figure S9. $^{13}C\{^{1}H\}$ NMR (151 MHz, $CDCl_3$) spectrum of *p*-methylphenyl 4,6-*O*-benzylidene-2-*O*-*tert*-butyldimethylsilyl-thio-β-D-mannopyranoside **33**:

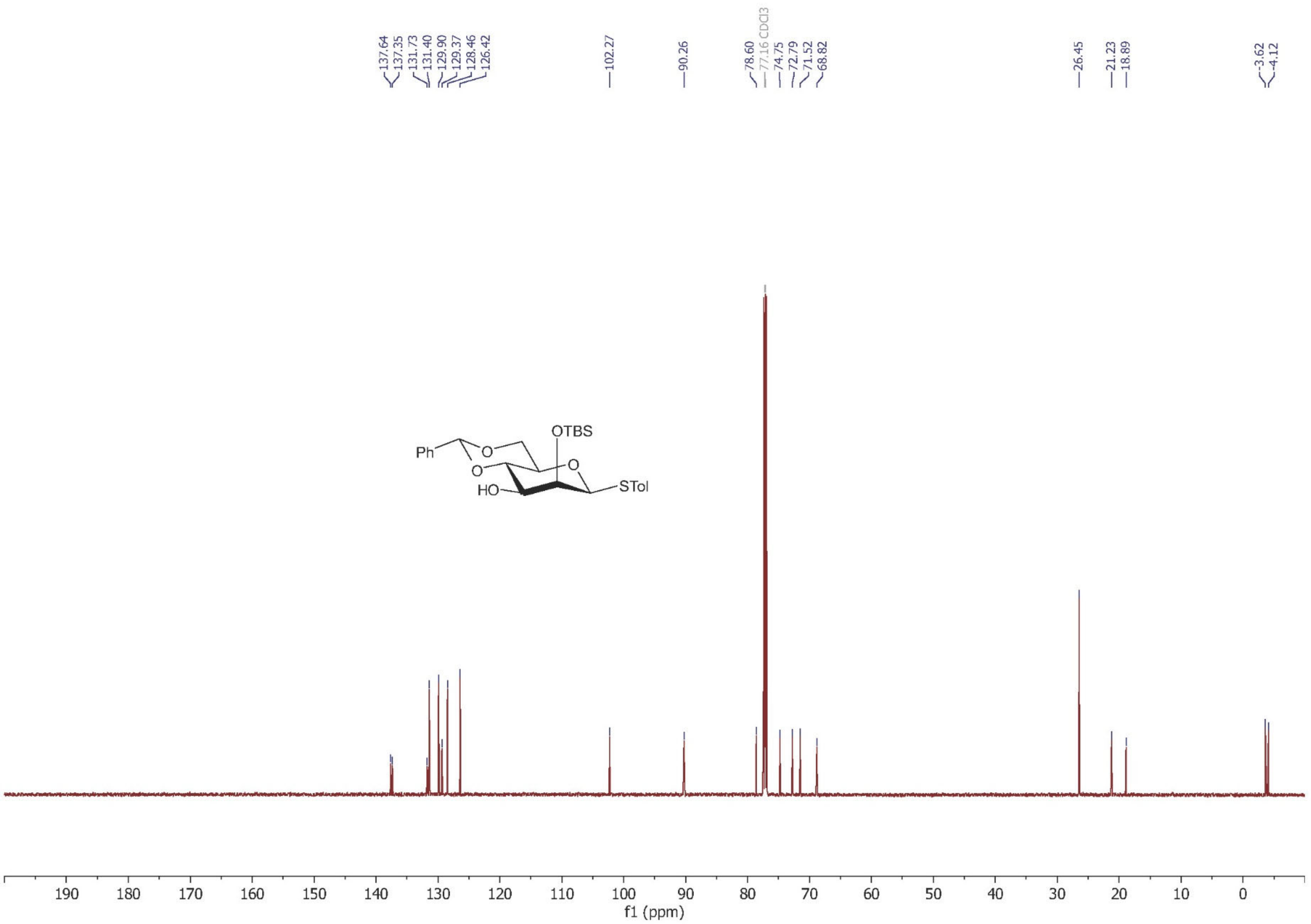

Figure S10. $^{13}C\{^1H\}$ DEPT NMR (151 MHz, $CDCl_3$) spectrum of *p*-methylphenyl 4,6-*O*-benzylidene-2-*O*-*tert*-butyldimethylsilyl-thio-β-D-mannopyranoside **33**:

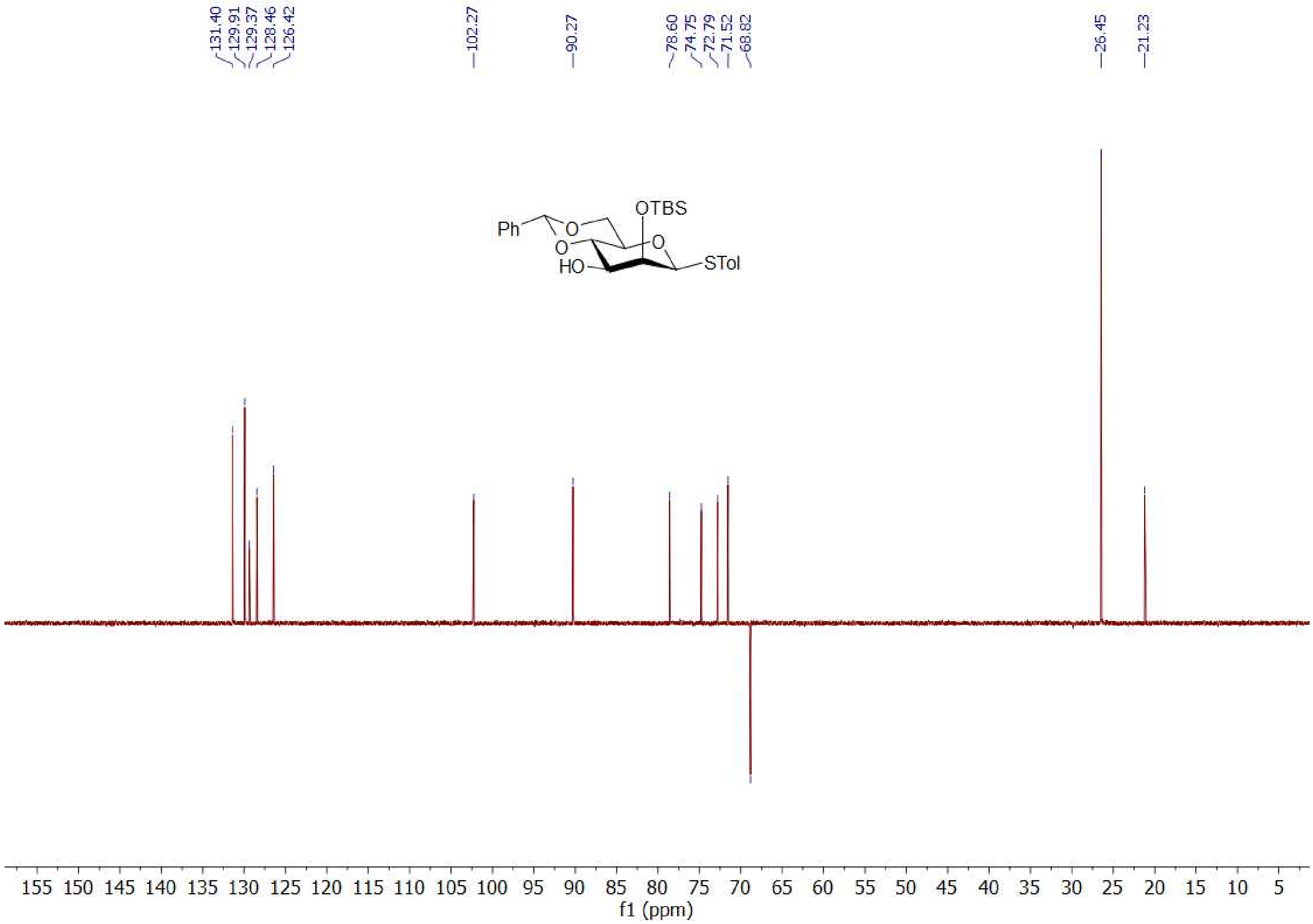

Figure S11. HSQC NMR (600 MHz, $CDCl_3$) spectrum of *p*-methylphenyl 4,6-*O*-benzylidene-2-*O*-*tert*-butyldimethylsilyl-thio-β-D-mannopyranoside **33**:

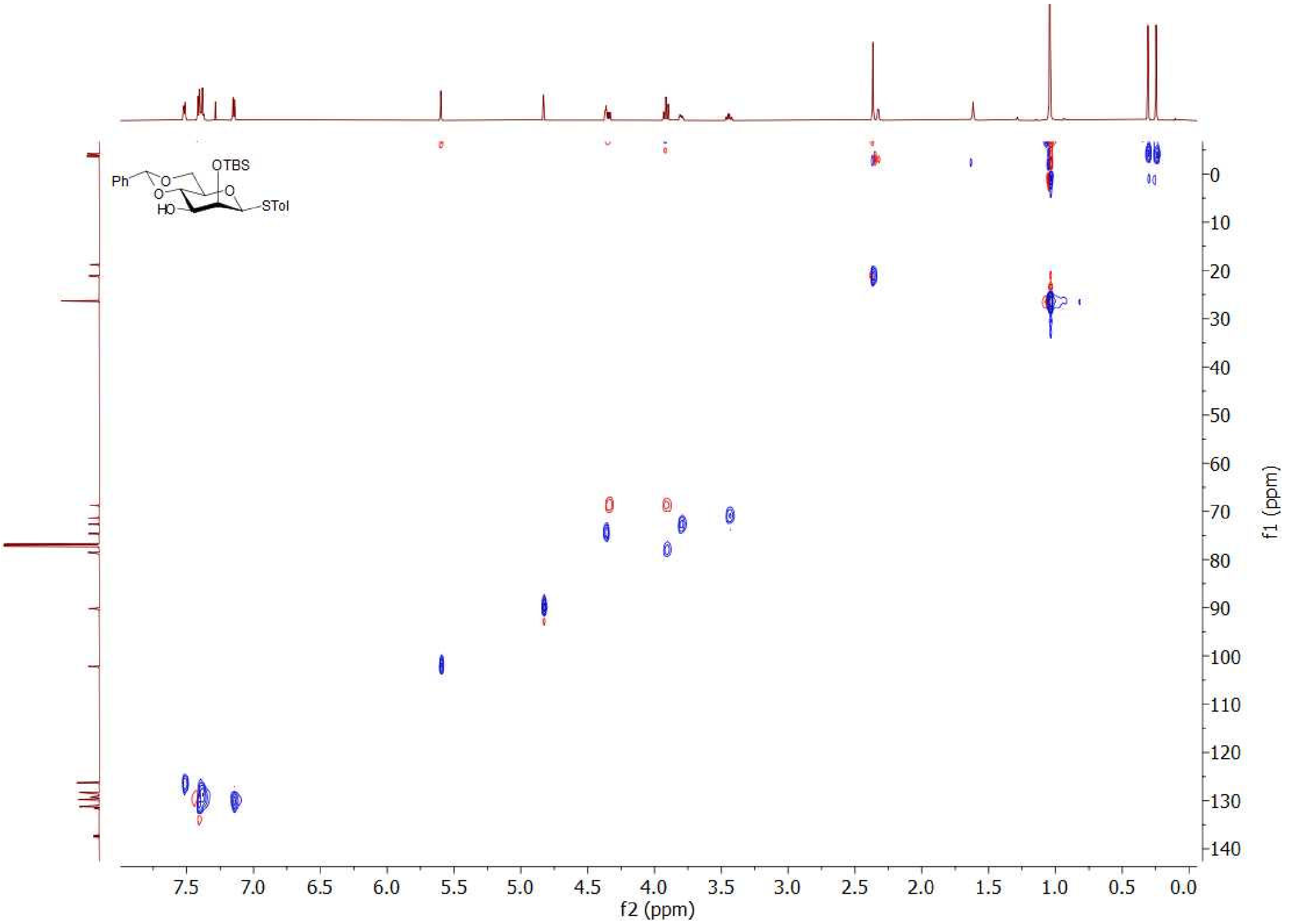

Figure S12. HMBC NMR (600 MHz, $CDCl_3$) spectrum of *p*-methylphenyl 4,6-*O*-benzylidene-2-*O*-*tert*-butyldimethylsilyl-thio-β-D-mannopyranoside **33**:

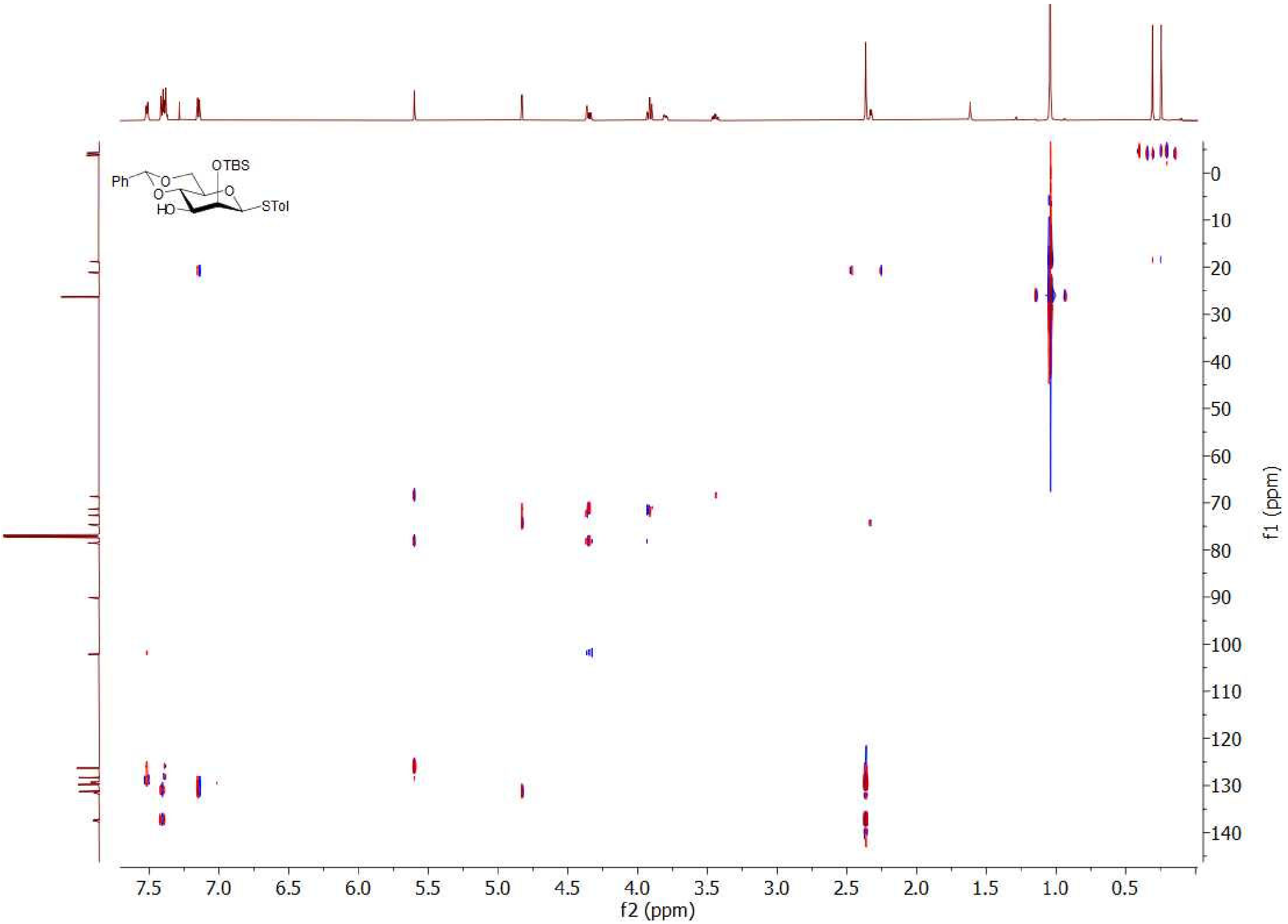

Figure S13. ^{1}H NMR (600 MHz, $CDCl_3$) spectrum of *p*-methylphenyl 4,6-*O*-benzylidene-2-*O*-*tert*-butyldimethylsilyl-thio-β-D-*arabino*-hexopyranosid-3-uloside **34**:

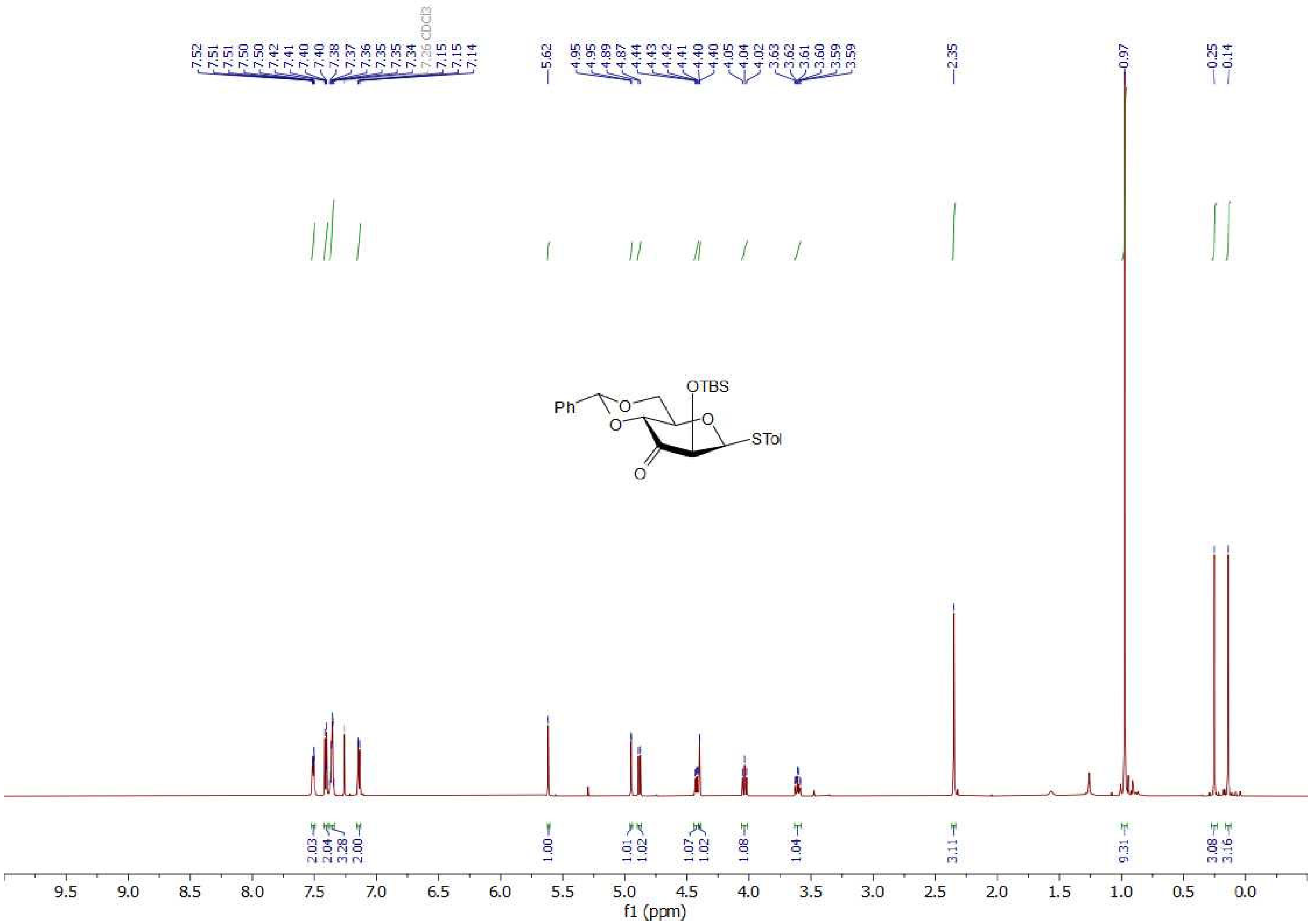

Figure S14. COSY NMR (600 MHz, $CDCl_3$) spectrum of *p*-methylphenyl 4,6-*O*-benzylidene-2-*O*-*tert*-butyldimethylsilyl-thio-β-D-*arabino*-hexopyranosid-3-uloside **34**:

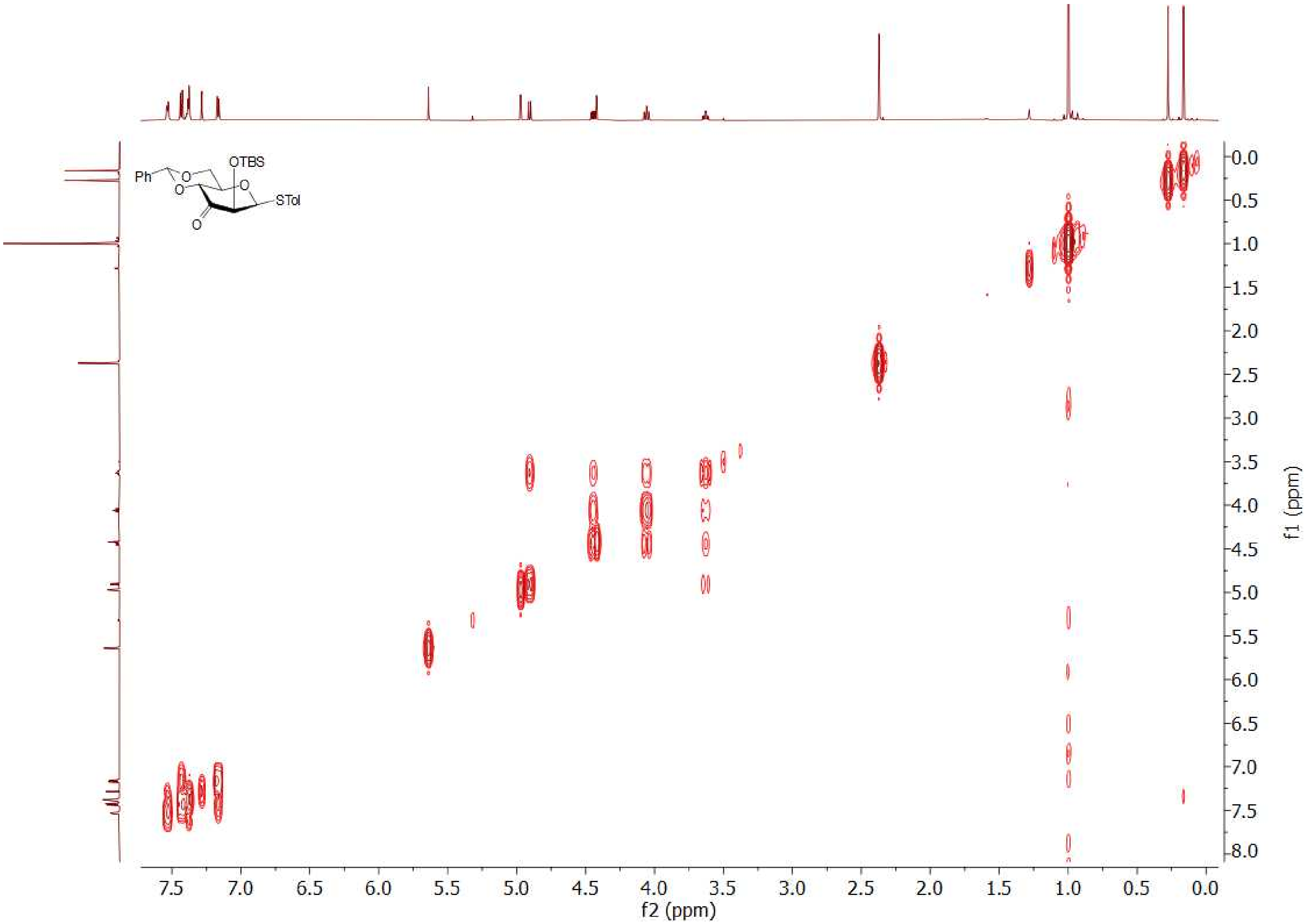

Figure S15. $^{13}C\{^{1}H\}$ NMR (151 MHz, $CDCl_3$) spectrum of *p*-methylphenyl 4,6-*O*-benzylidene-2-*O*-*tert*-butyldimethylsilyl-thio-β-D-*arabino*-hexopyranosid-3-uloside **34**:

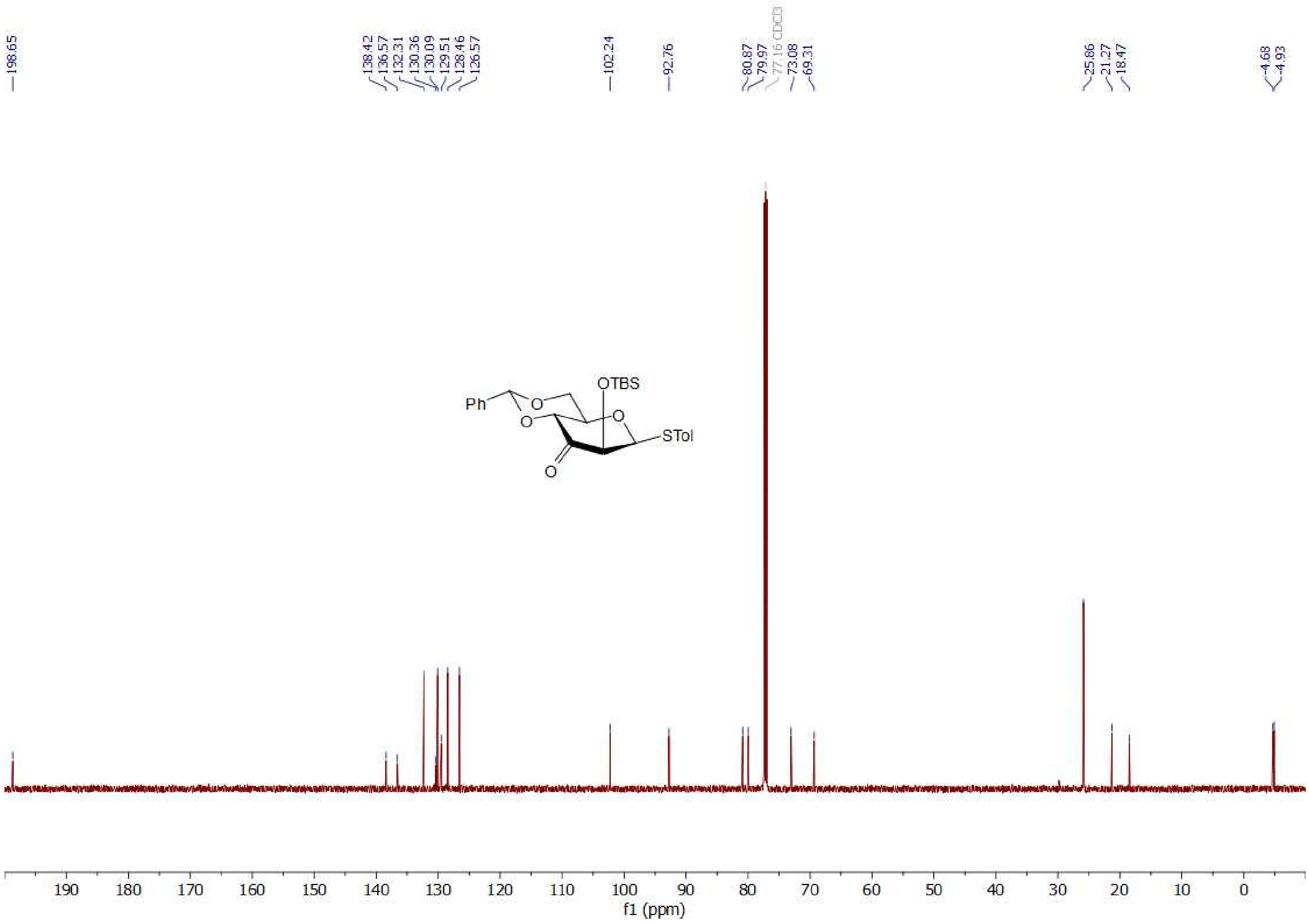

Figure S16. $^{13}C\{^1H\}$ DEPT NMR (151 MHz, $CDCl_3$) spectrum of *p*-methylphenyl 4,6-*O*-benzylidene-2-*O*-*tert*-butyldimethylsilyl-thio-β-D-*arabino*-hexopyranosid-3-uloside **34**:

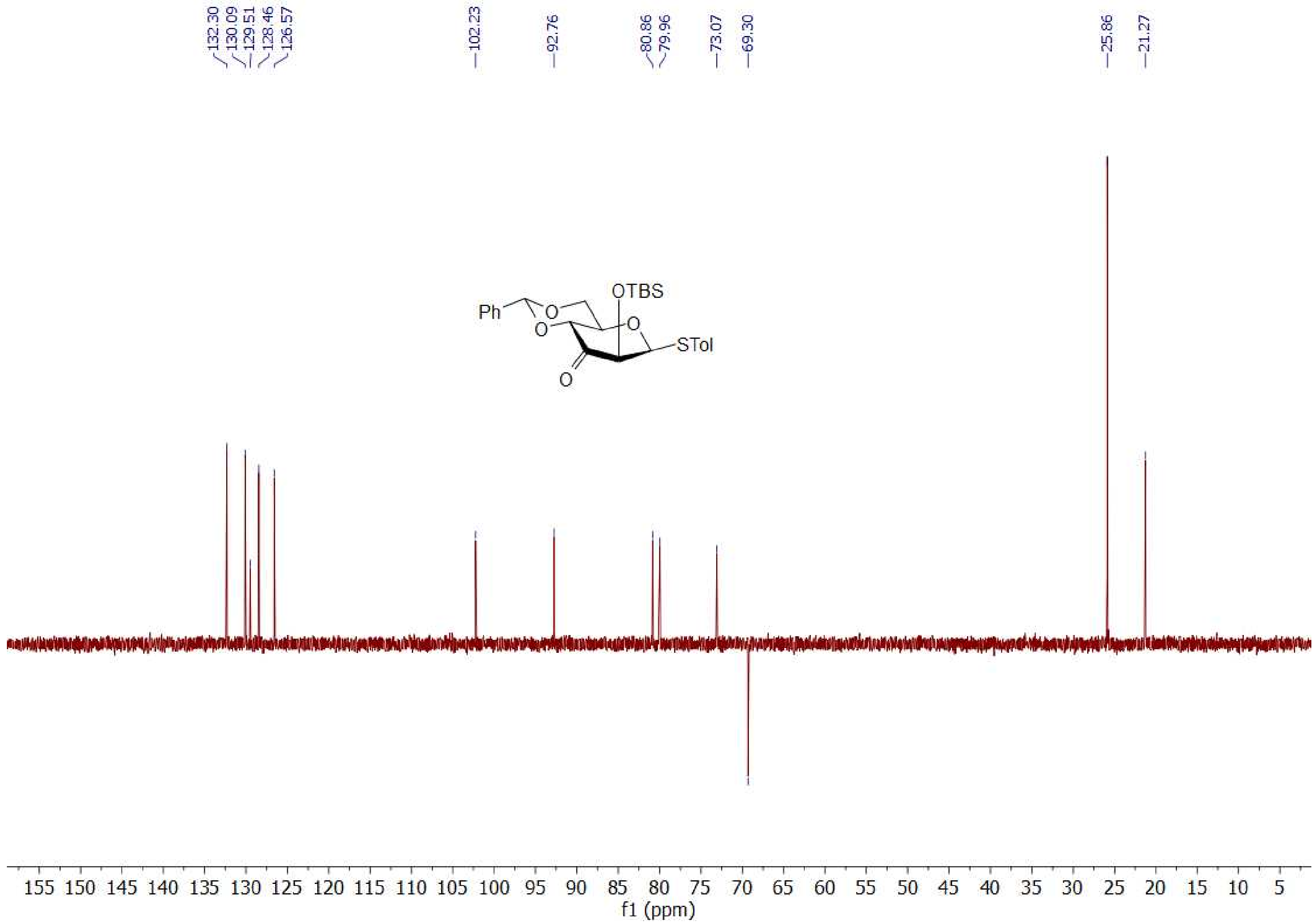

Figure S17. HSQC NMR (600 MHz, $CDCl_3$) spectrum of *p*-methylphenyl 4,6-*O*-benzylidene-2-*O*-*tert*-butyldimethylsilyl-thio-β-D-*arabino*-hexopyranosid-3-uloside **34**:

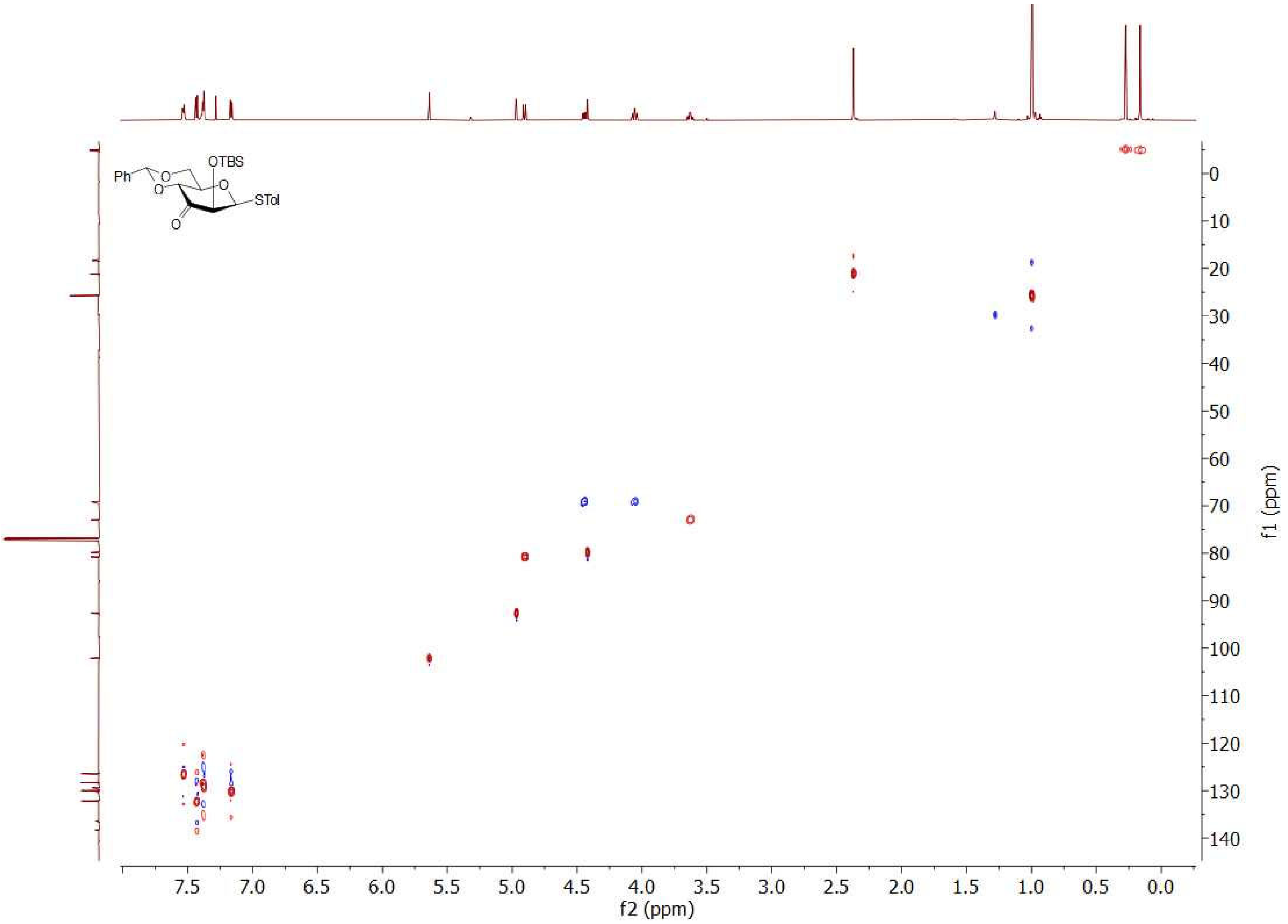

Figure S18. HMBC NMR (600 MHz, $CDCl_3$) spectrum of *p*-methylphenyl 4,6-*O*-benzylidene-2-*O*-*tert*-butyldimethylsilyl-thio-β-D-*arabino*-hexopyranosid-3-uloside **34**:

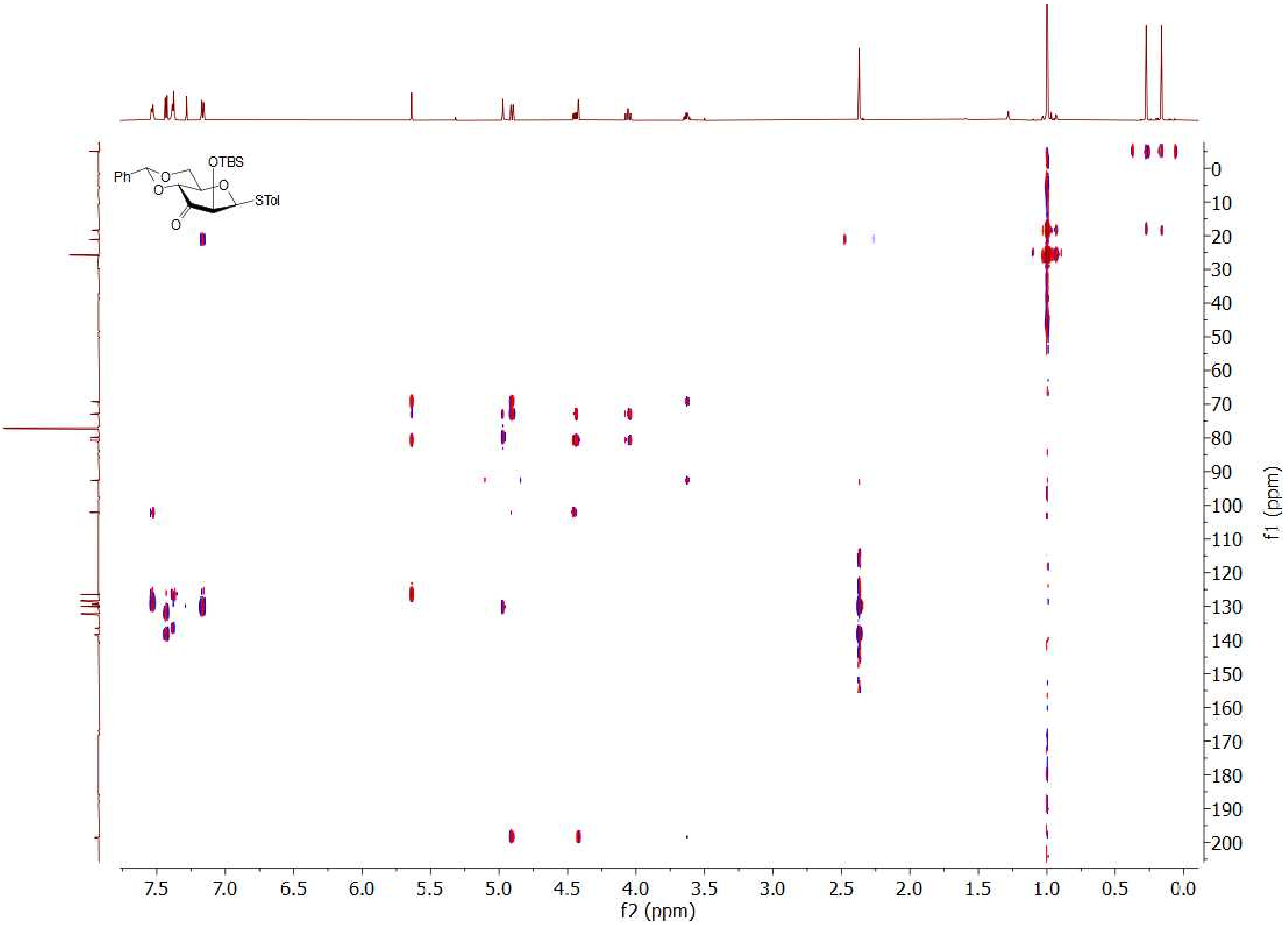

Figure S19. ^{1}H NMR (600 MHz, $CDCl_3$) spectrum of *p*-methylphenyl 4,6-*O*-benzylidene-2-*O*-*tert*-butyldimethylsilyl-3-*C*-methyl-thio-β-D-mannopyranoside **35**:

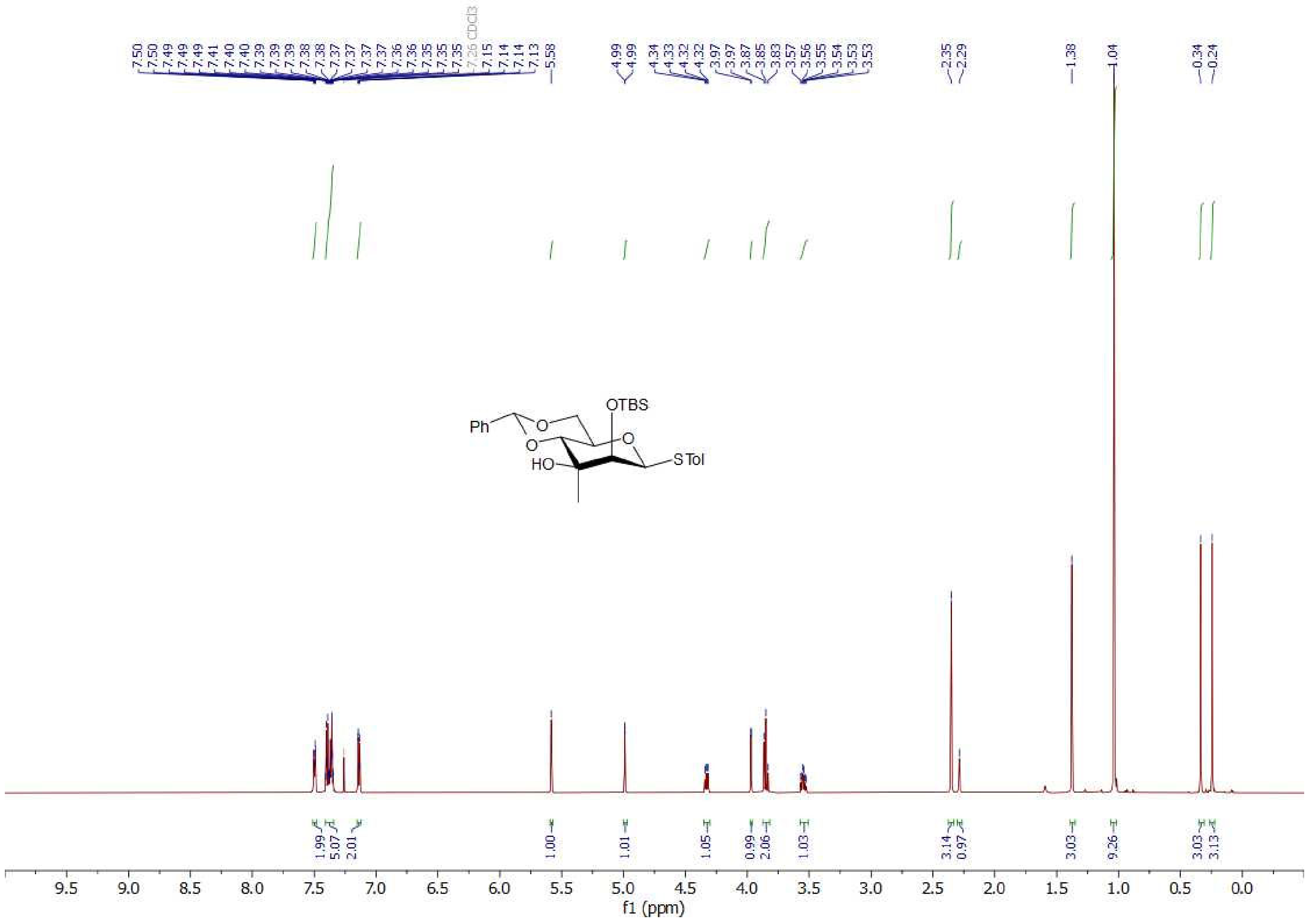

Figure S20. COSY NMR (600 MHz, $CDCl_3$) spectrum of *p*-methylphenyl 4,6-*O*-benzylidene-2-*O*-*tert*-butyldimethylsilyl-3-*C*-methyl-thio-β-D-mannopyranoside **35**:

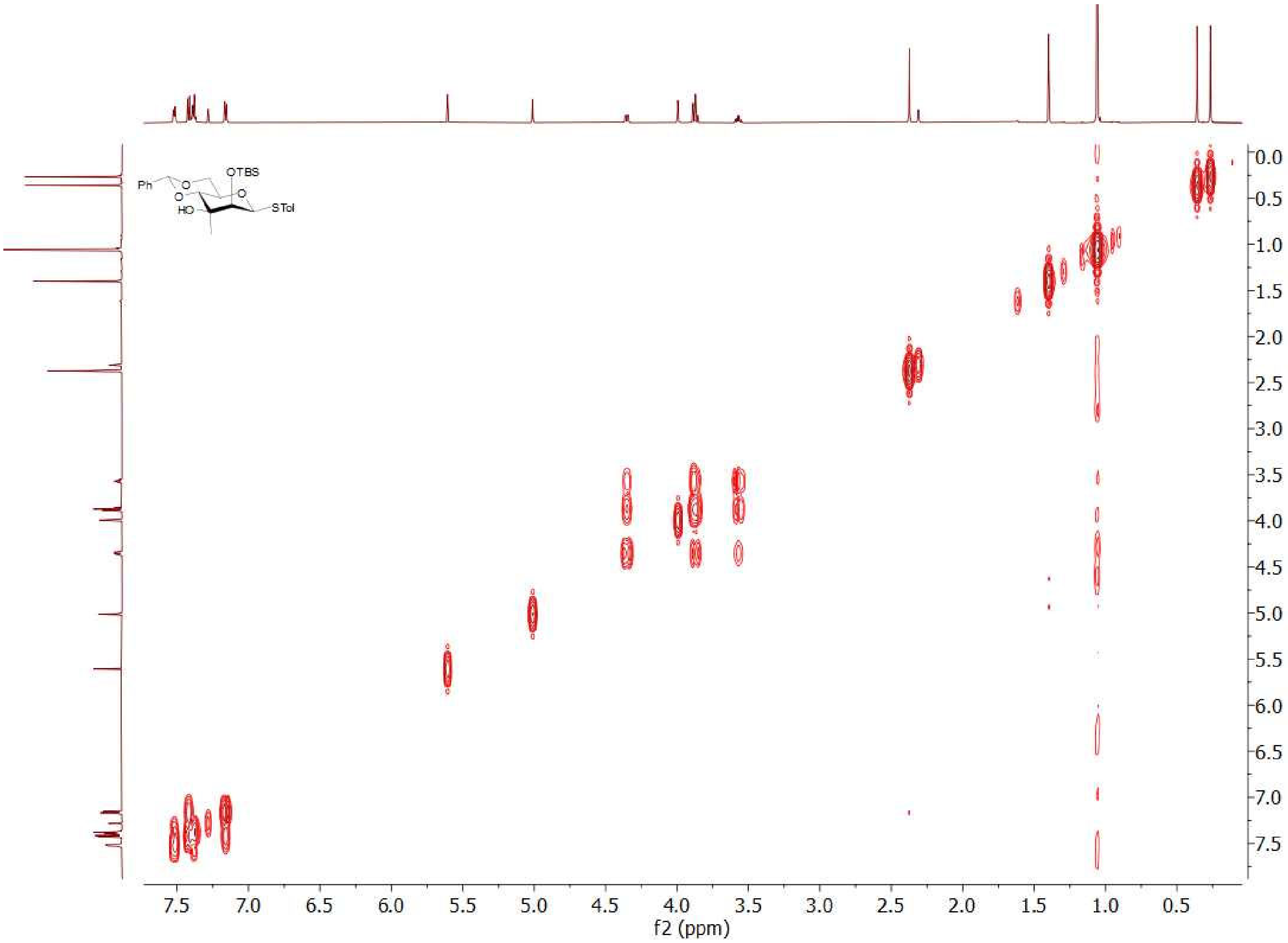

Figure S21. $^{13}C\{^{1}H\}$ NMR (151 MHz, $CDCl_3$) spectrum of *p*-methylphenyl 4,6-*O*-benzylidene-2-*O*-*tert*-butyldimethylsilyl-3-*C*-methyl-thio-β-D-mannopyranoside **35**:

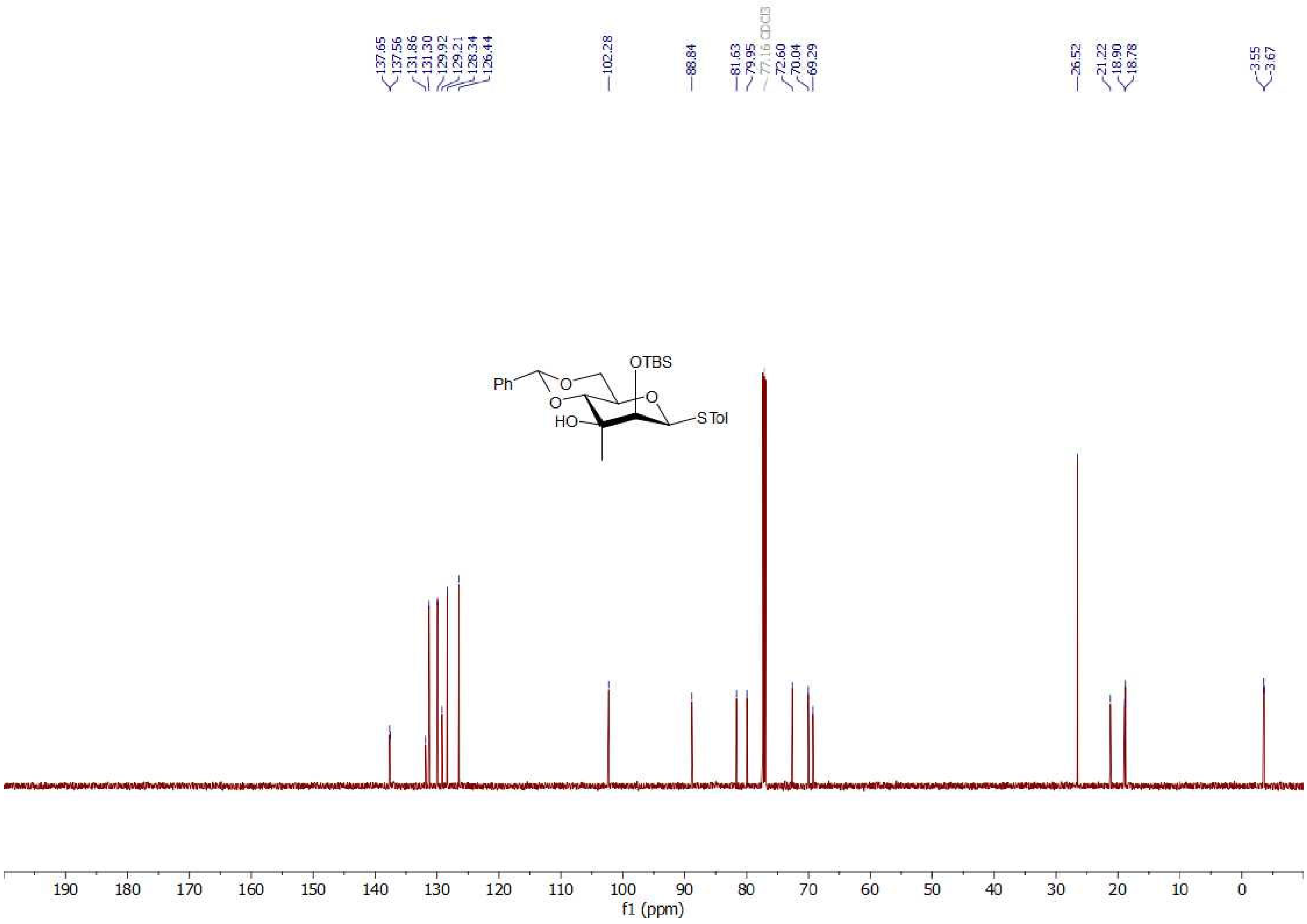

Figure S22. $^{13}C\{^{1}H\}$ DEPT NMR (151 MHz, $CDCl_3$) spectrum of *p*-methylphenyl 4,6-*O*-benzylidene-2-*O*-*tert*-butyldimethylsilyl-3-*C*-methyl-thio-β-D-mannopyranoside **35**:

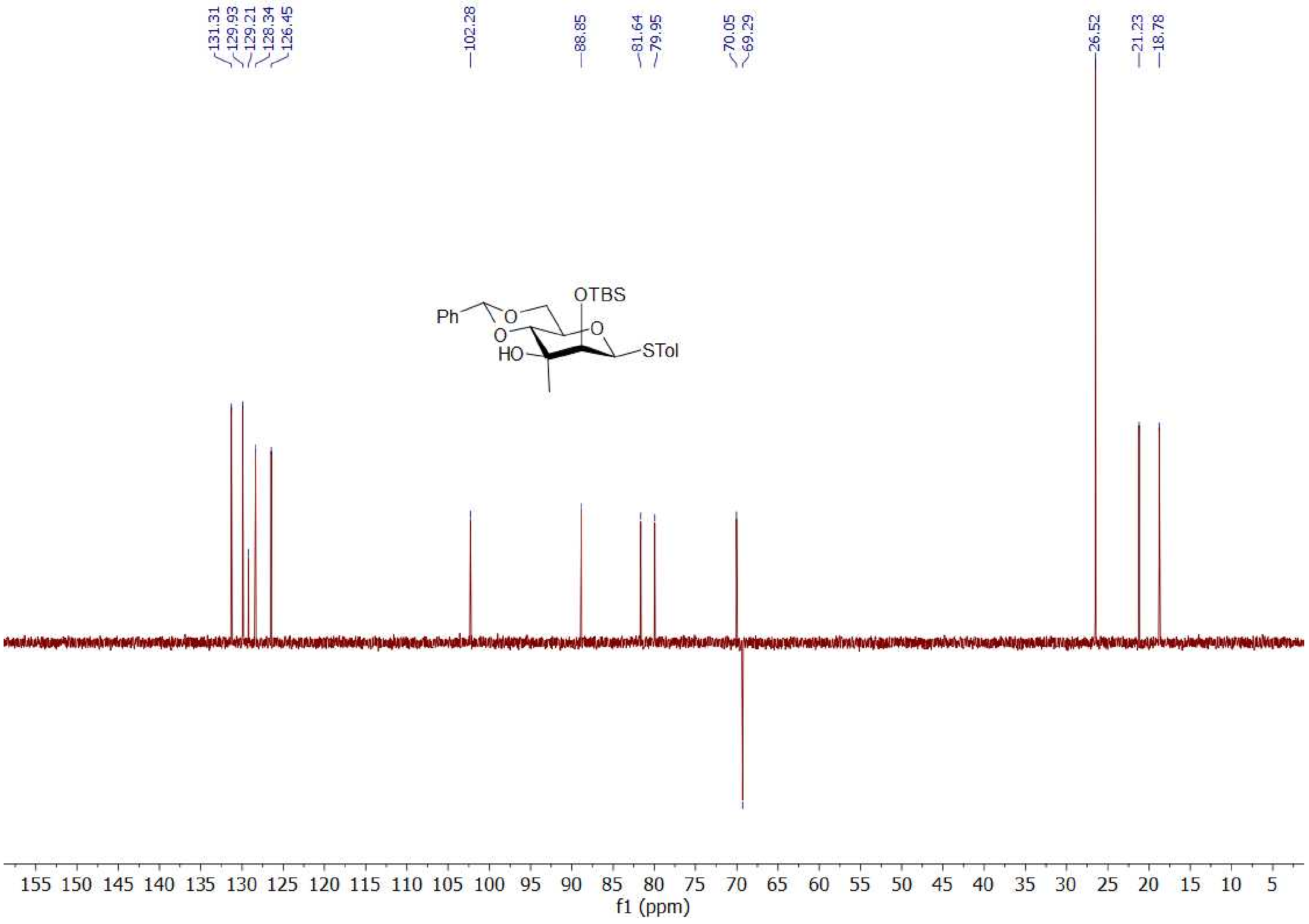

Figure S23. HSQC NMR (600 MHz, $CDCl_3$) spectrum of *p*-methylphenyl 4,6-*O*-benzylidene-2-*O*-*tert*-butyldimethylsilyl-3-*C*-methyl-thio-β-D-mannopyranoside **35**:

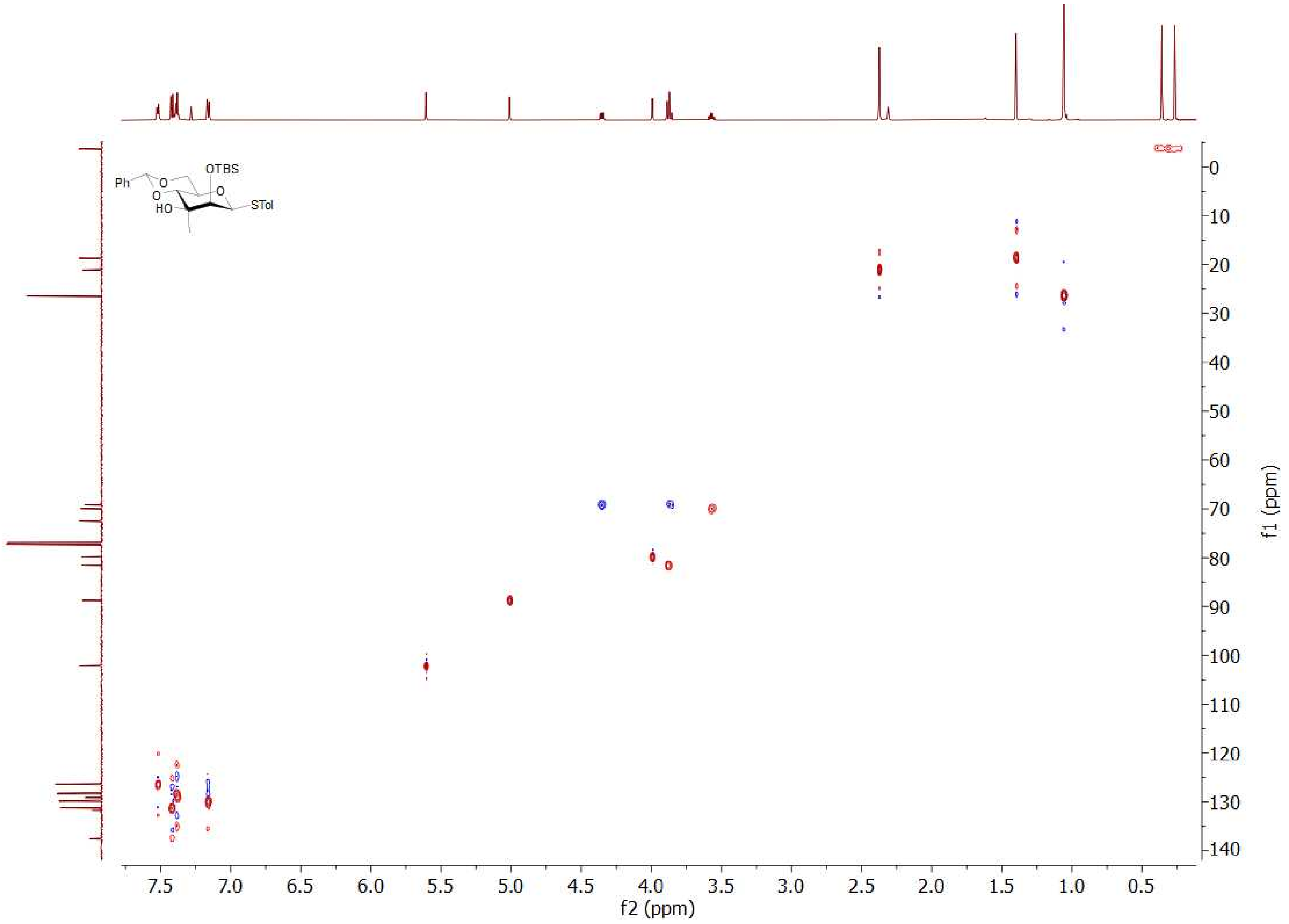

Figure S24. HMBC NMR (600 MHz, $CDCl_3$) spectrum of *p*-methylphenyl 4,6-*O*-benzylidene-2-*O*-*tert*-butyldimethylsilyl-3-*C*-methyl-thio-β-D-mannopyranoside **35**:

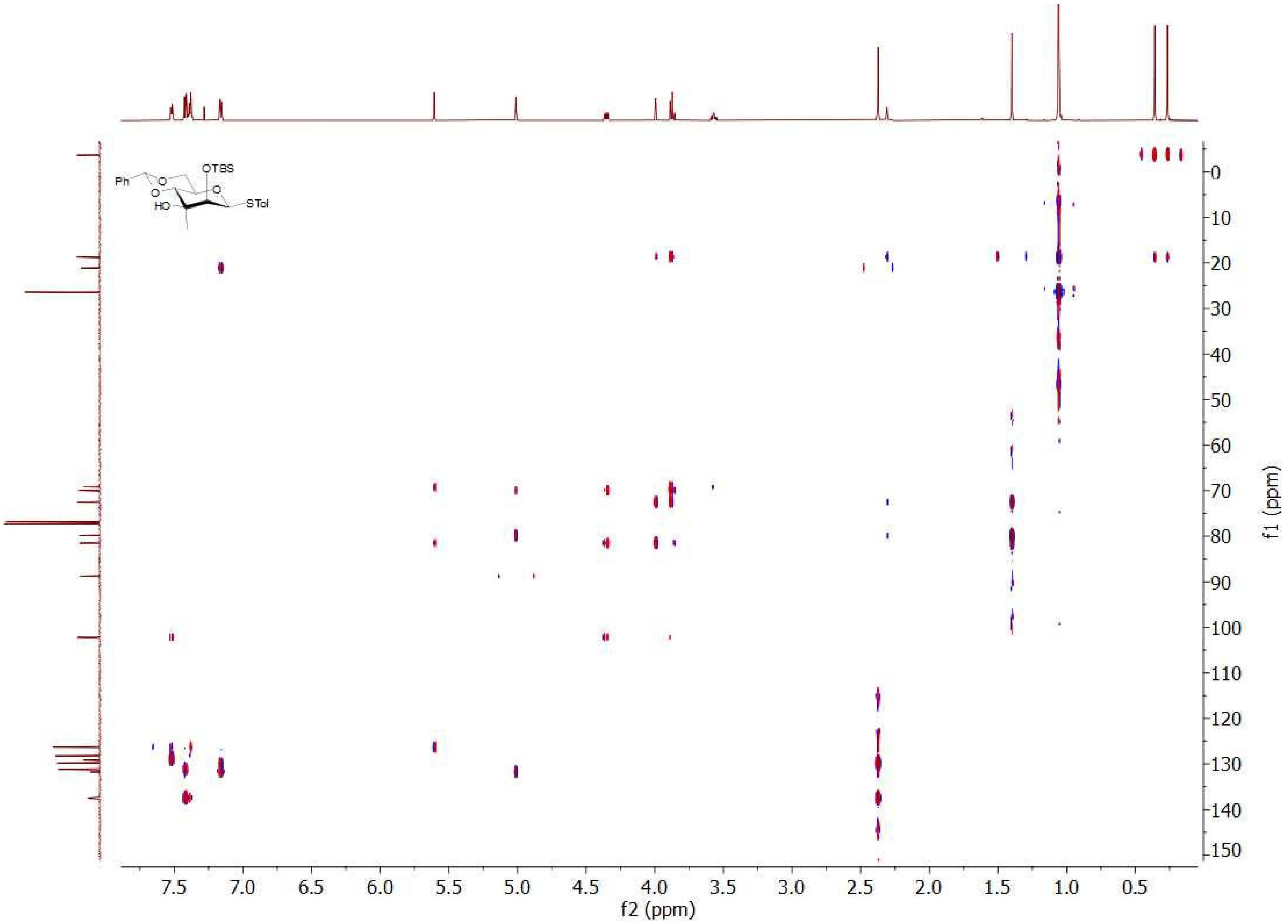

Figure S25. ^{1}H (NOE) NMR (600 MHz, $CDCl_3$) spectrum of *p*-methylphenyl 4,6-*O*-benzylidene-2-*O*-*tert*-butyldimethylsilyl-3-*C*-methyl-thio-β-D-mannopyranoside **35**:

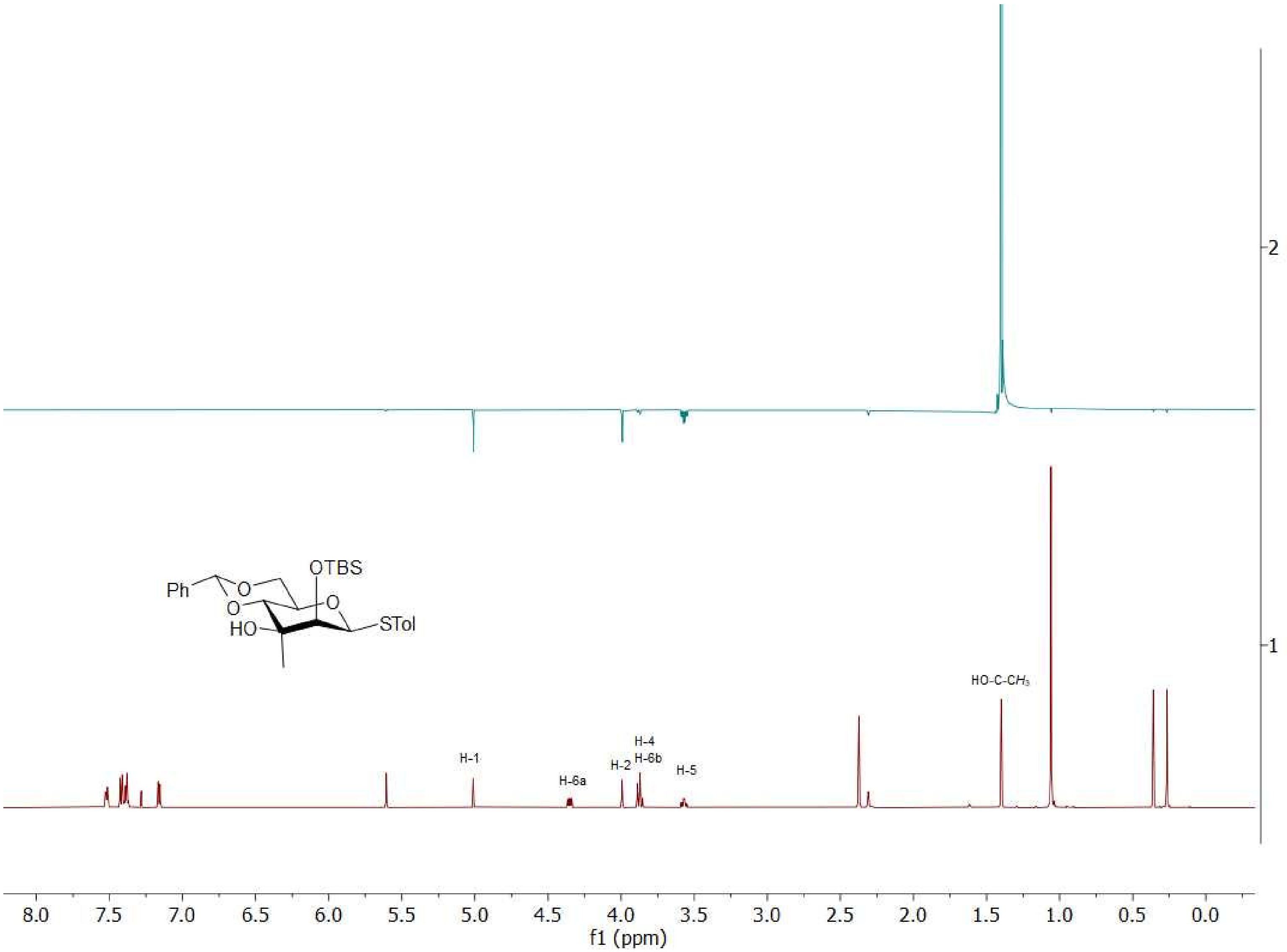

Figure S26. ^{1}H NMR (600 MHz, $CDCl_3$) spectrum of *p*-methylphenyl 4,6-*O*-benzylidene-3-*C*-methyl-thio-β-D-mannopyranoside **36**:

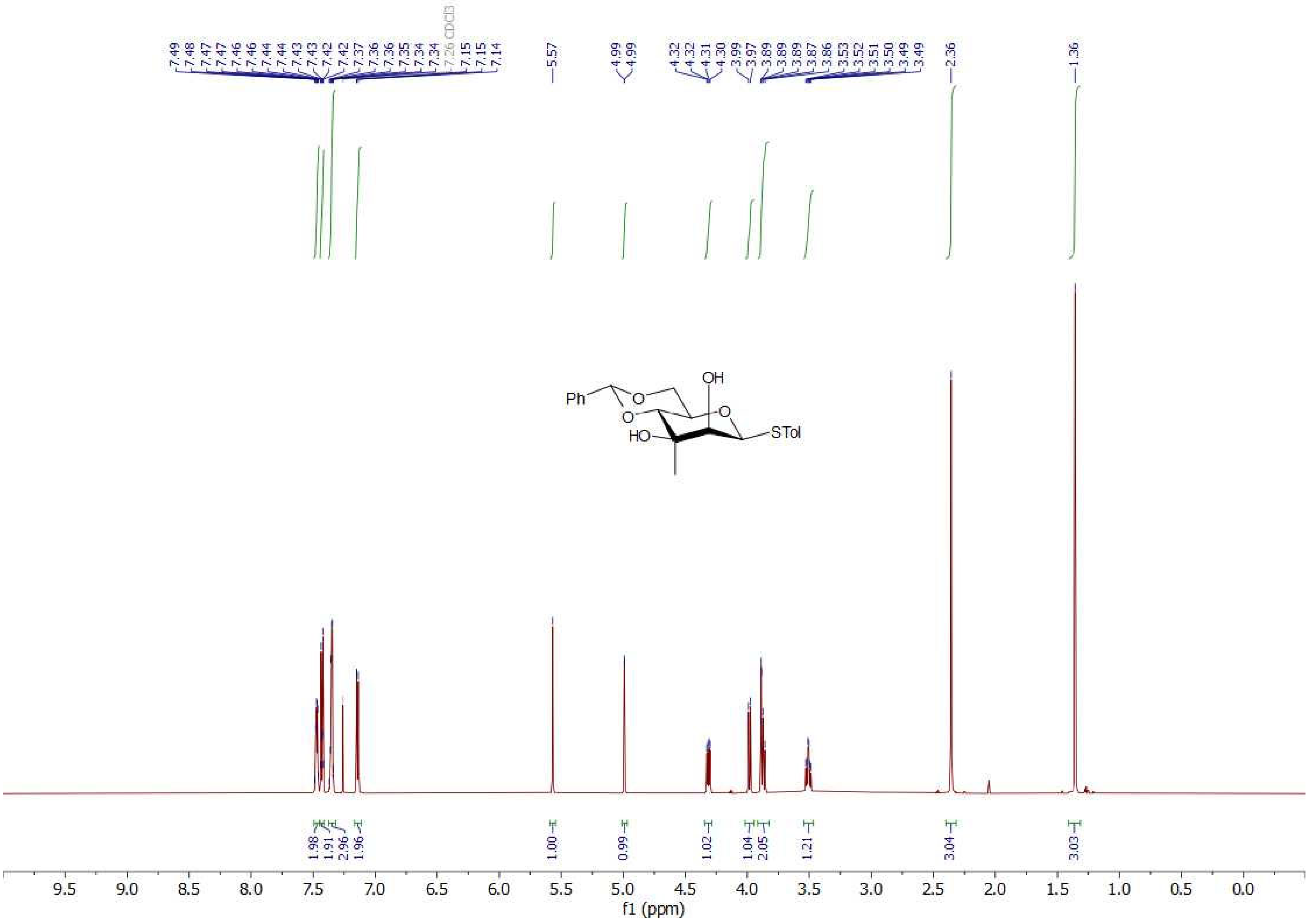

Figure S27. COSY NMR (600 MHz, $CDCl_3$) spectrum of *p*-methylphenyl 4,6-*O*-benzylidene-3-*C*-methyl-thio-β-D-mannopyranoside **36**:

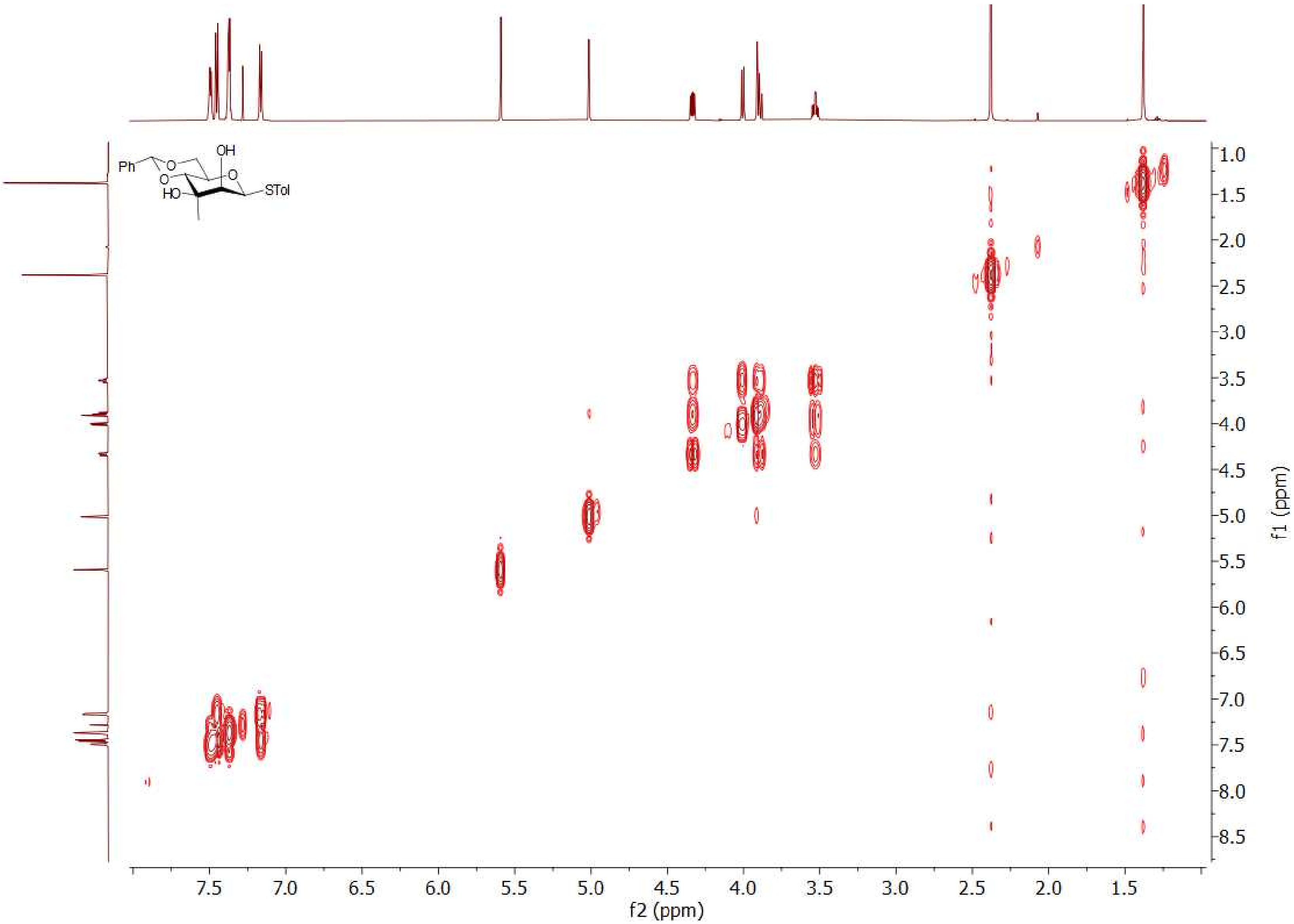

Figure S28. $^{13}C\{^{1}H\}$ NMR (151 MHz, $CDCl_3$) spectrum of *p*-methylphenyl 4,6-*O*-benzylidene-3-*C*-methyl-thio-β-D-mannopyranoside **36**:

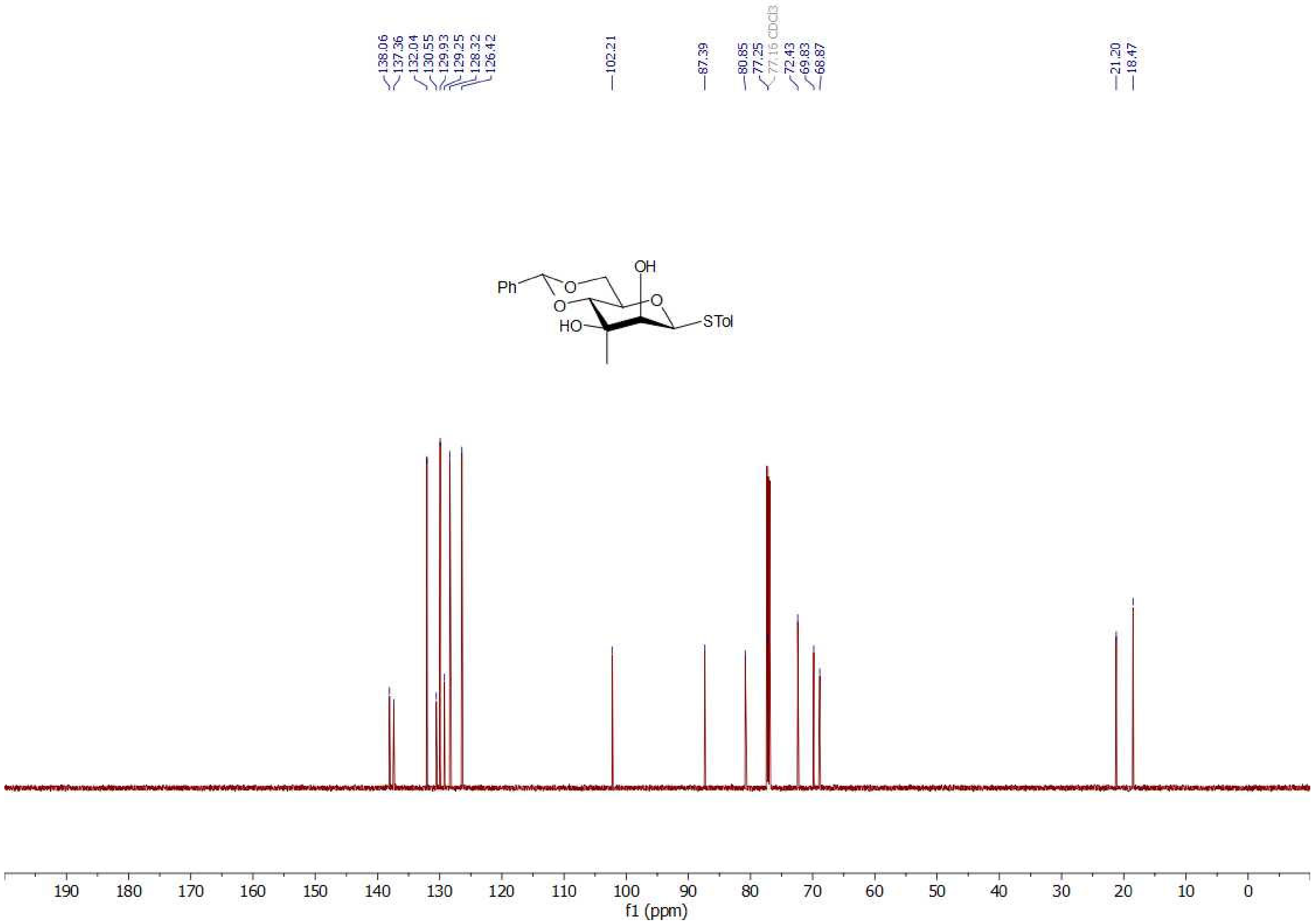

Figure S29. $^{13}C\{^1H\}$ DEPT NMR (151 MHz, $CDCl_3$) spectrum of *p*-methylphenyl 4,6-*O*-benzylidene-3-*C*-methyl-thio-β-D-mannopyranoside **36**:

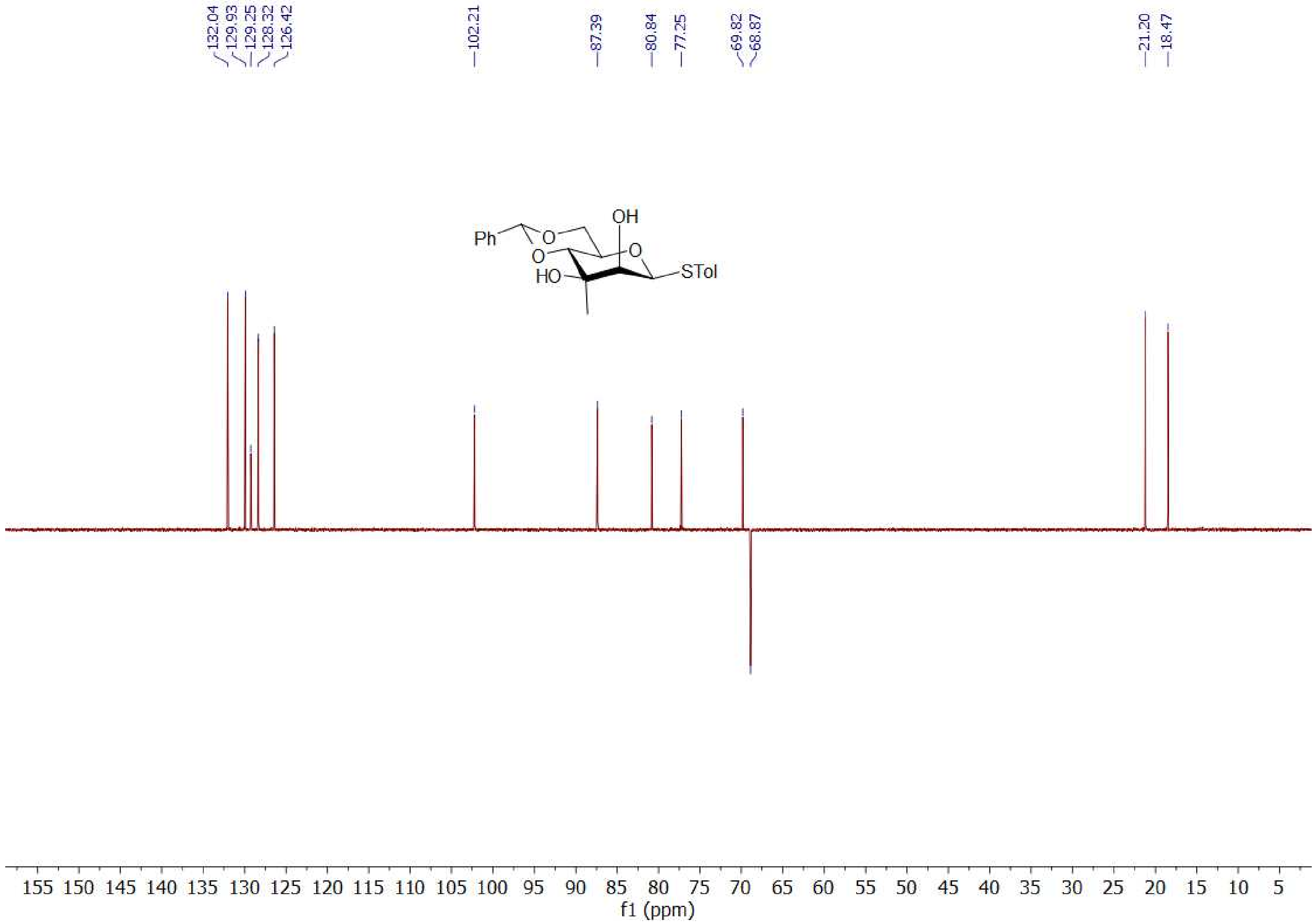

Figure S30. HSQC NMR (600 MHz, $CDCl_3$) spectrum of *p*-methylphenyl 4,6-*O*-benzylidene-3-*C*-methyl-thio-β-D-mannopyranoside **36**:

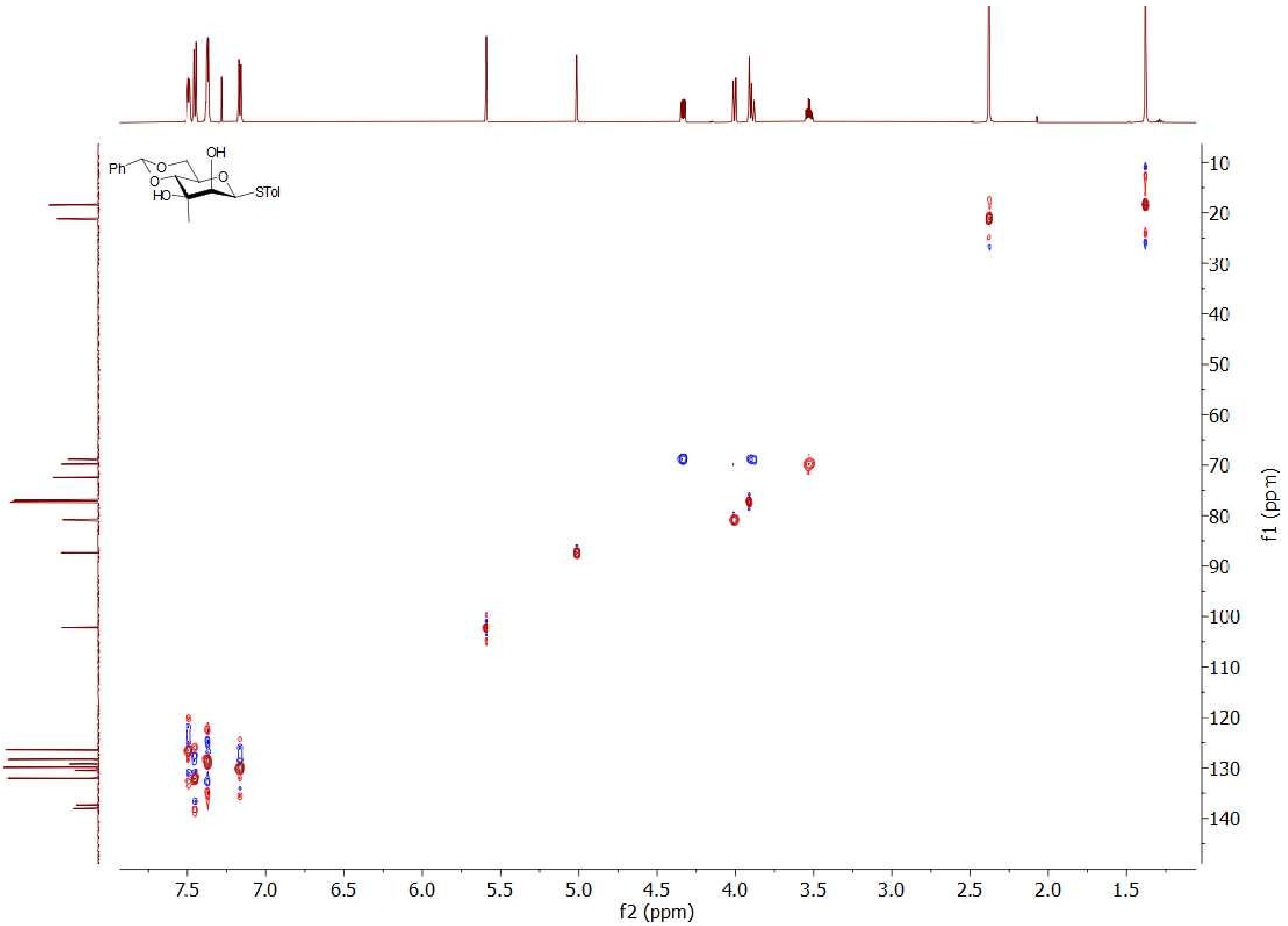

Figure S31. HMBC NMR (600 MHz, $CDCl_3$) spectrum of *p*-methylphenyl 4,6-*O*-benzylidene-3-*C*-methyl-thio-β-D-mannopyranoside **36**:

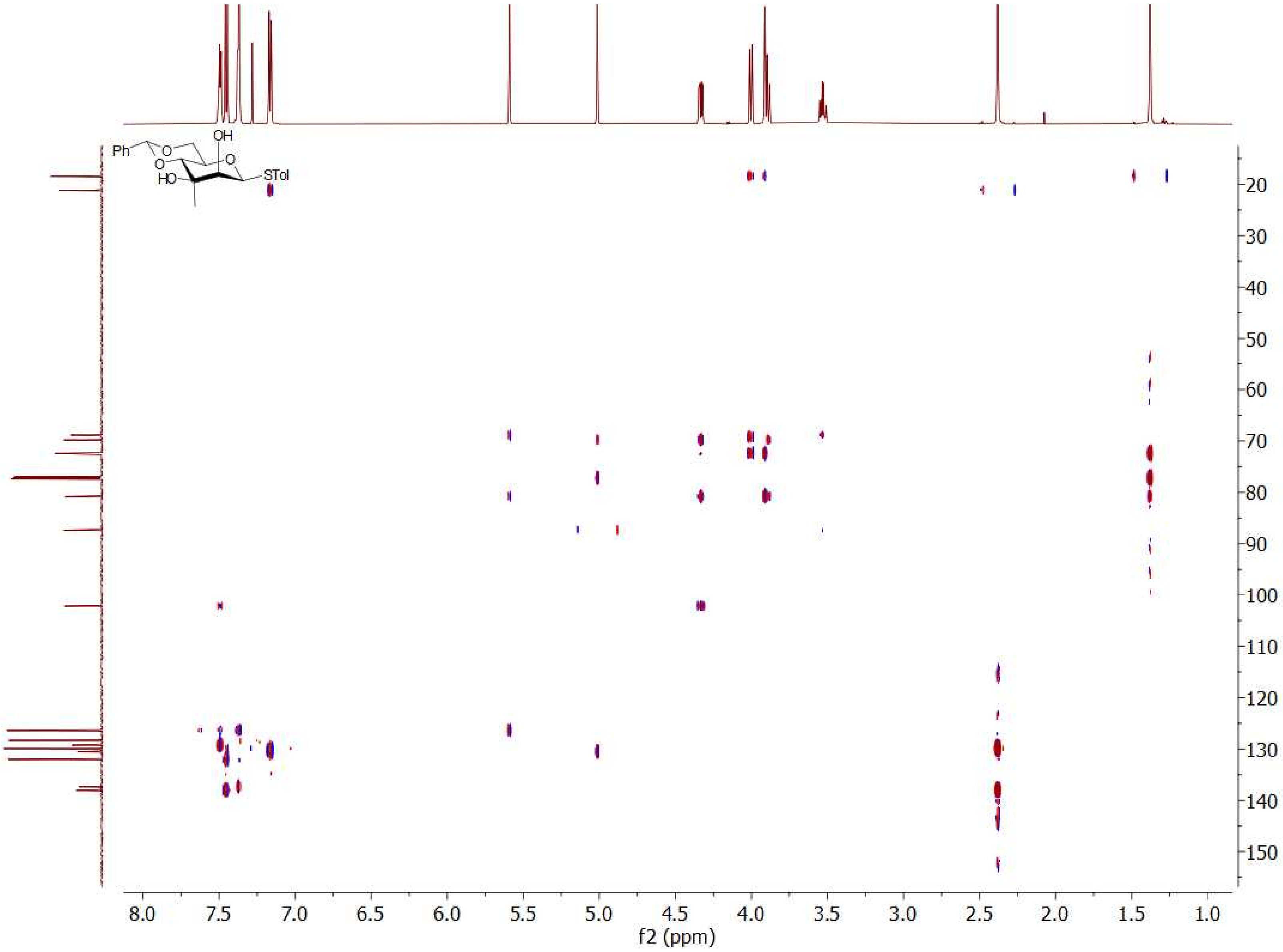

Figure S32. ^{1}H NMR (600 MHz, $CDCl_3$) spectrum of *p*-methylphenyl 2-*O*-benzyl-4,6-*O*-benzylidene-3-*C*-methyl-thio-β-D-mannopyranoside **37**:

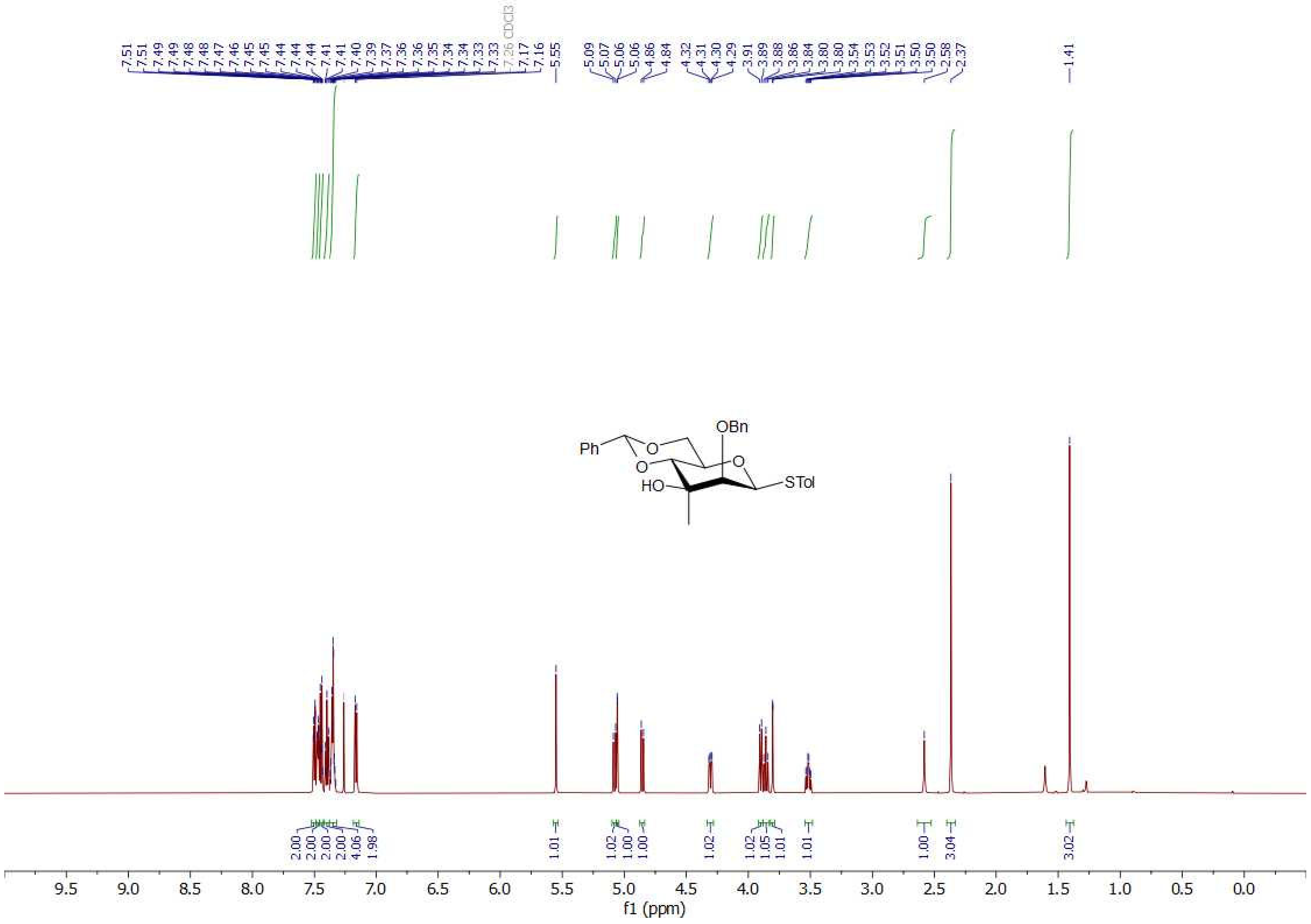

Figure S33. **C**OSY NMR (600 MHz, $CDCl_3$) spectrum of *p*-methylphenyl 2-*O*-benzyl-4,6-*O*-benzylidene-3-*C*-methyl-thio-β-D-mannopyranoside **37**:

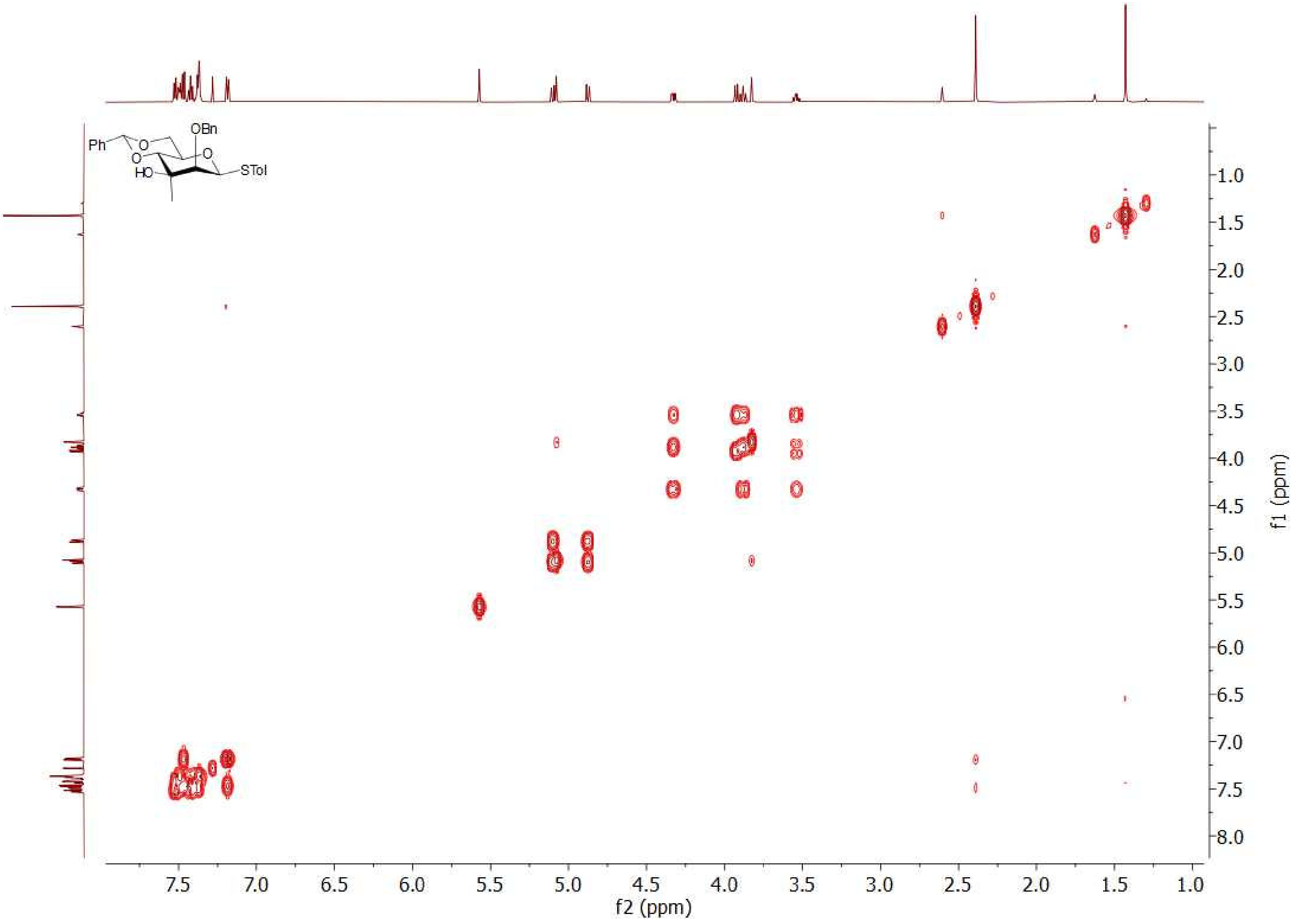

Figure S34. $^{13}C\{^{1}H\}$ NMR (151 MHz, $CDCl_3$) spectrum of *p*-methylphenyl 2-*O*-benzyl-4,6-*O*-benzylidene-3-*C*-methyl-thio-β-D-mannopyranoside **37**:

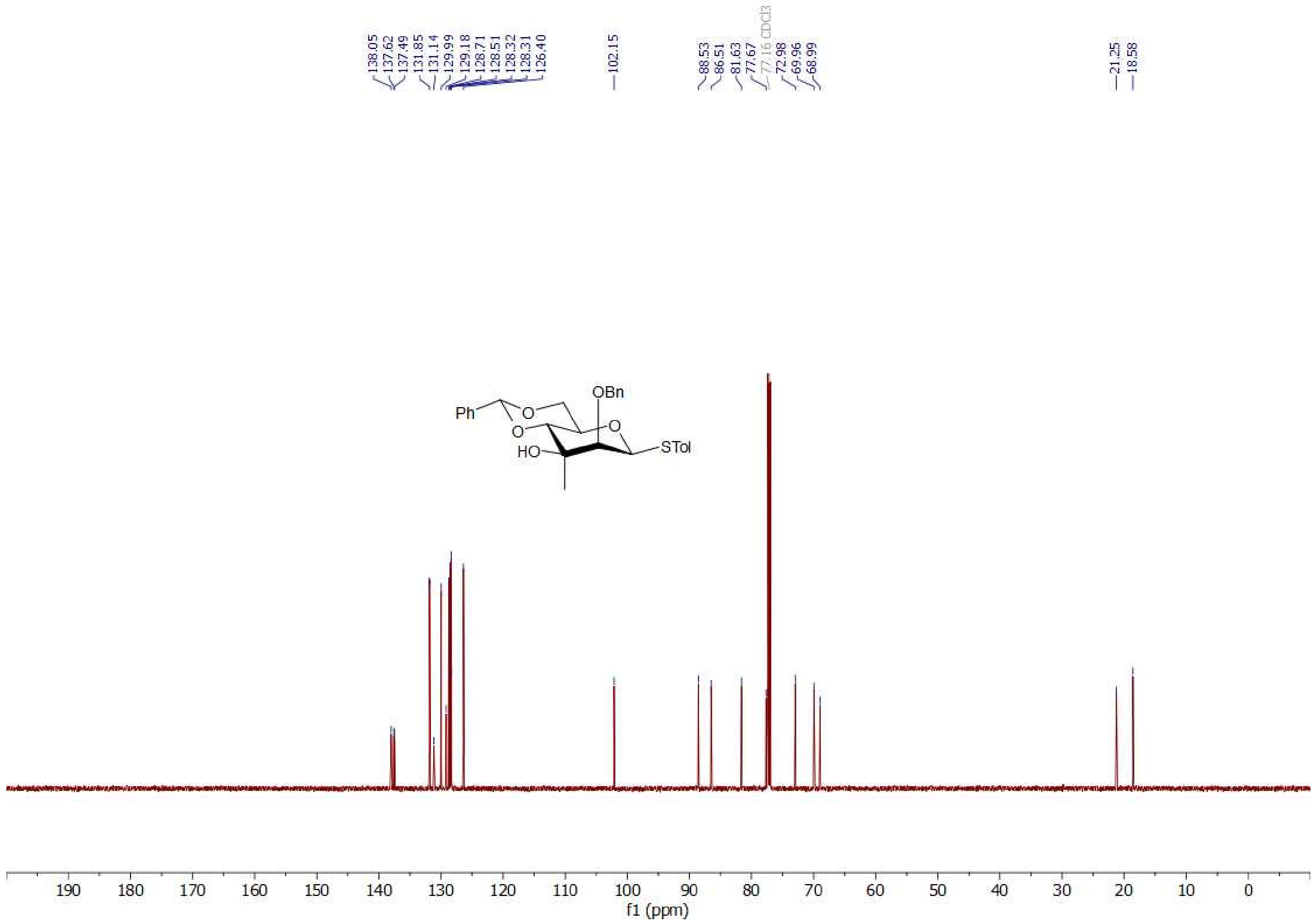

Figure S35. $^{13}C\{^1H\}$ DEPT NMR (151 MHz, $CDCl_3$) spectrum of *p*-methylphenyl 2-*O*-benzyl-4,6-*O*-benzylidene-3-*C*-methyl-thio-β-D-mannopyranoside **37**:

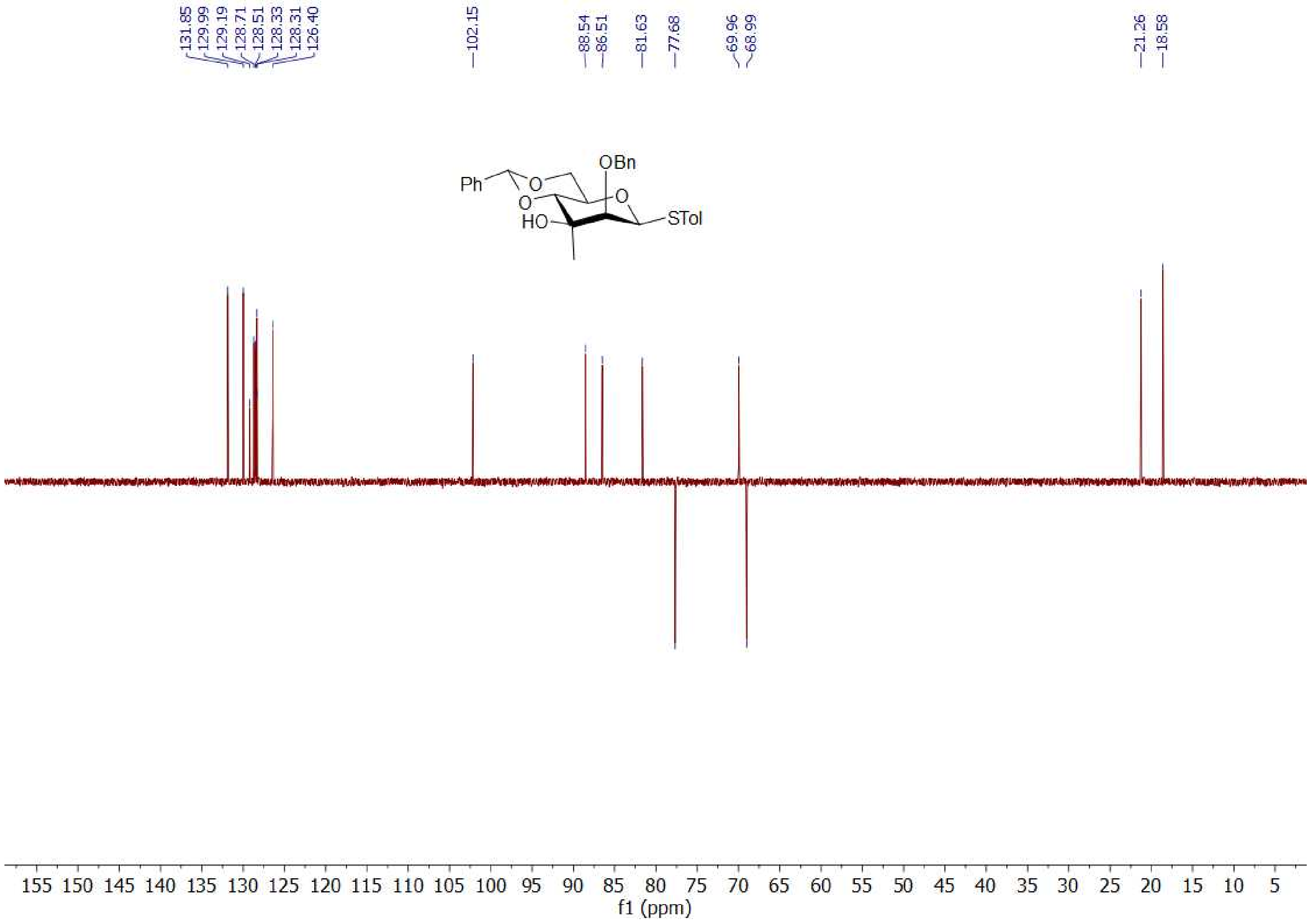

Figure S36. ^{13}C (C-H coupled) NMR (151 MHz, $CDCl_3$) spectrum of *p*-methylphenyl 2-*O*-benzyl-4,6-*O*-benzylidene-3-*C*-methyl-thio-β-D-mannopyranoside **37**:

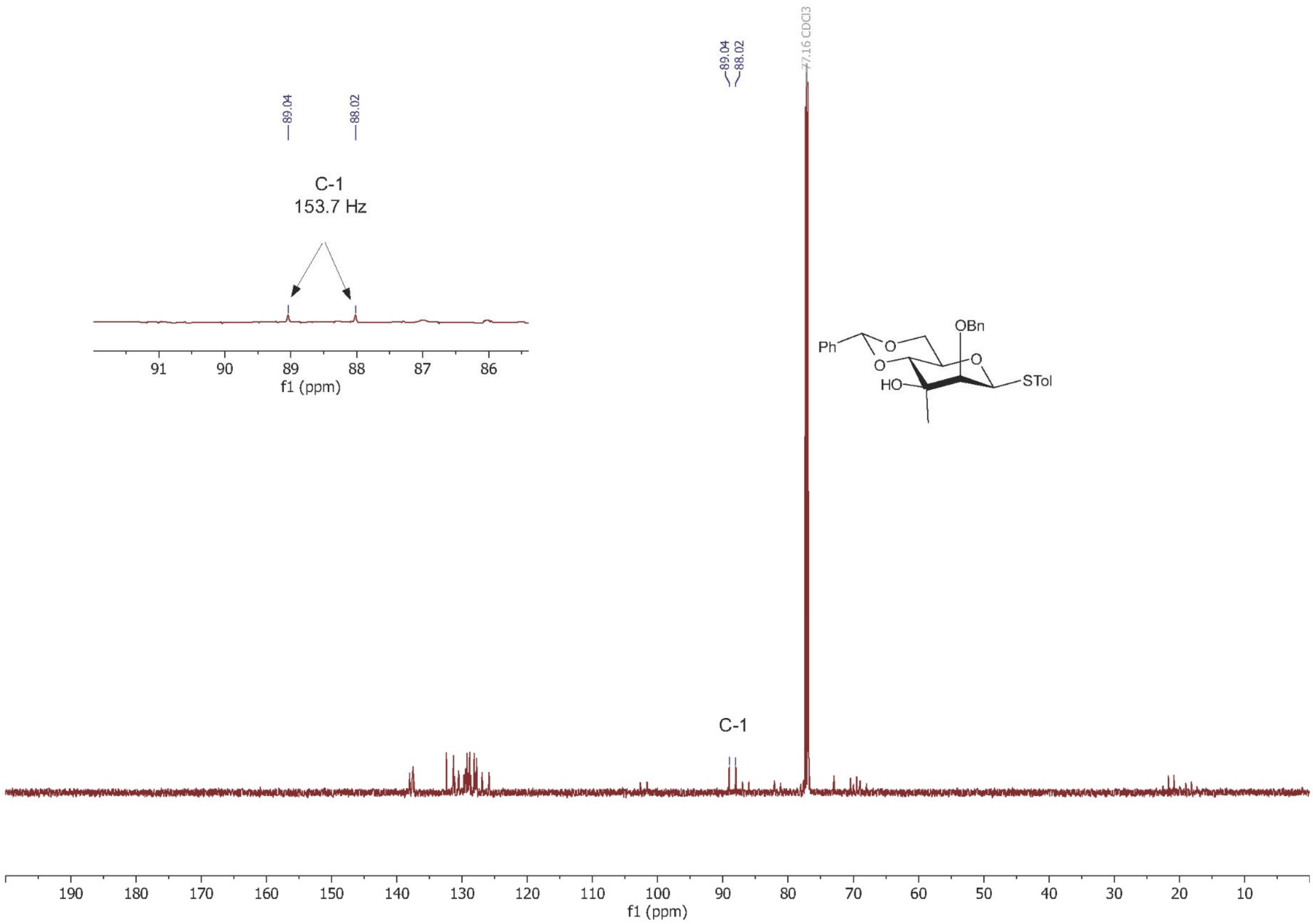

Figure S37. HSQC NMR (600 MHz, $CDCl_3$) spectrum of *p*-methylphenyl 2-*O*-benzyl-4,6-*O*-benzylidene-3-*C*-methyl-thio-β-D-mannopyranoside **37**:

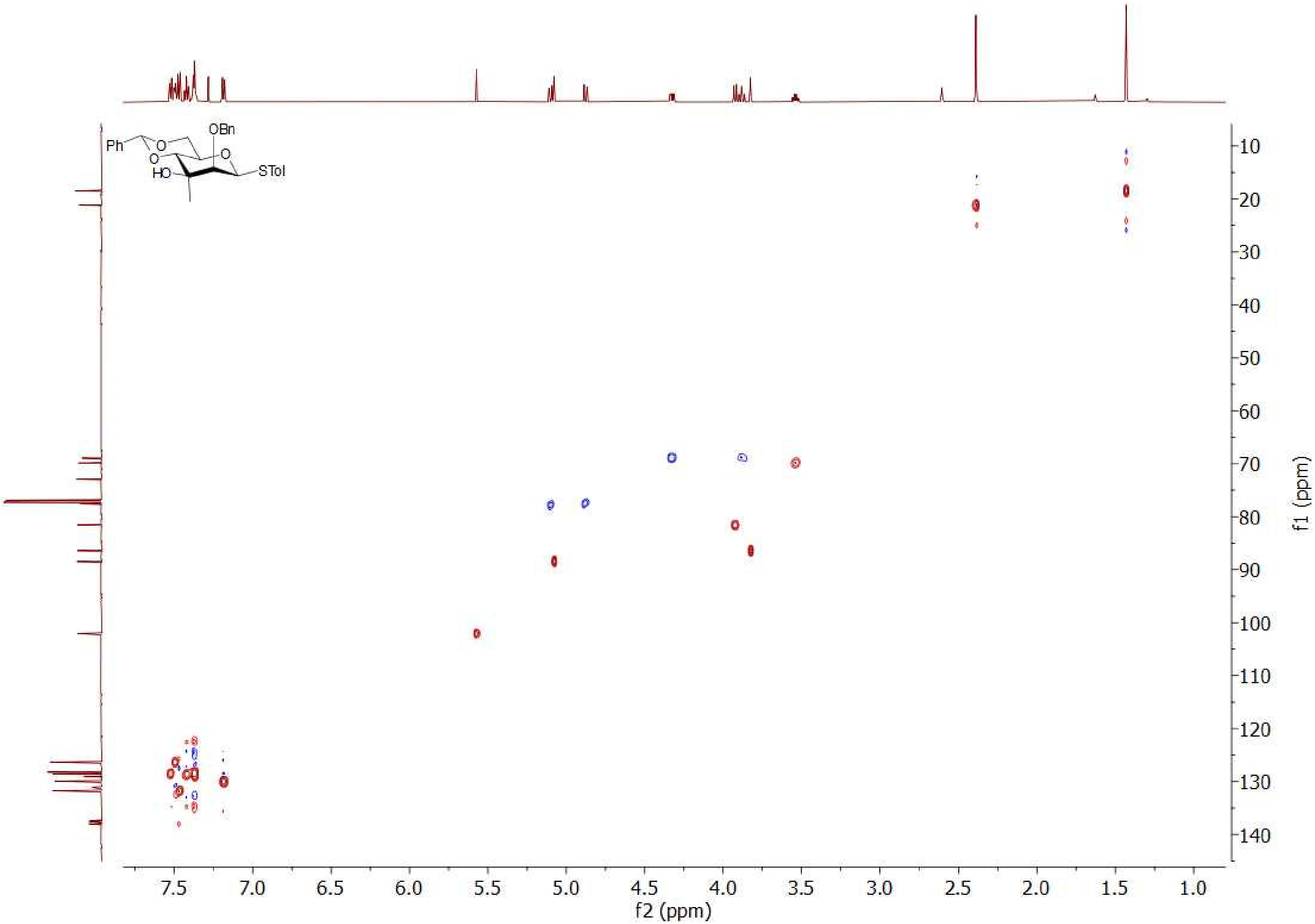

Figure S38. HMBC NMR (600 MHz, $CDCl_3$) spectrum of *p*-methylphenyl 2-*O*-benzyl-4,6-*O*-benzylidene-3-*C*-methyl-thio-β-D-mannopyranoside **37**:

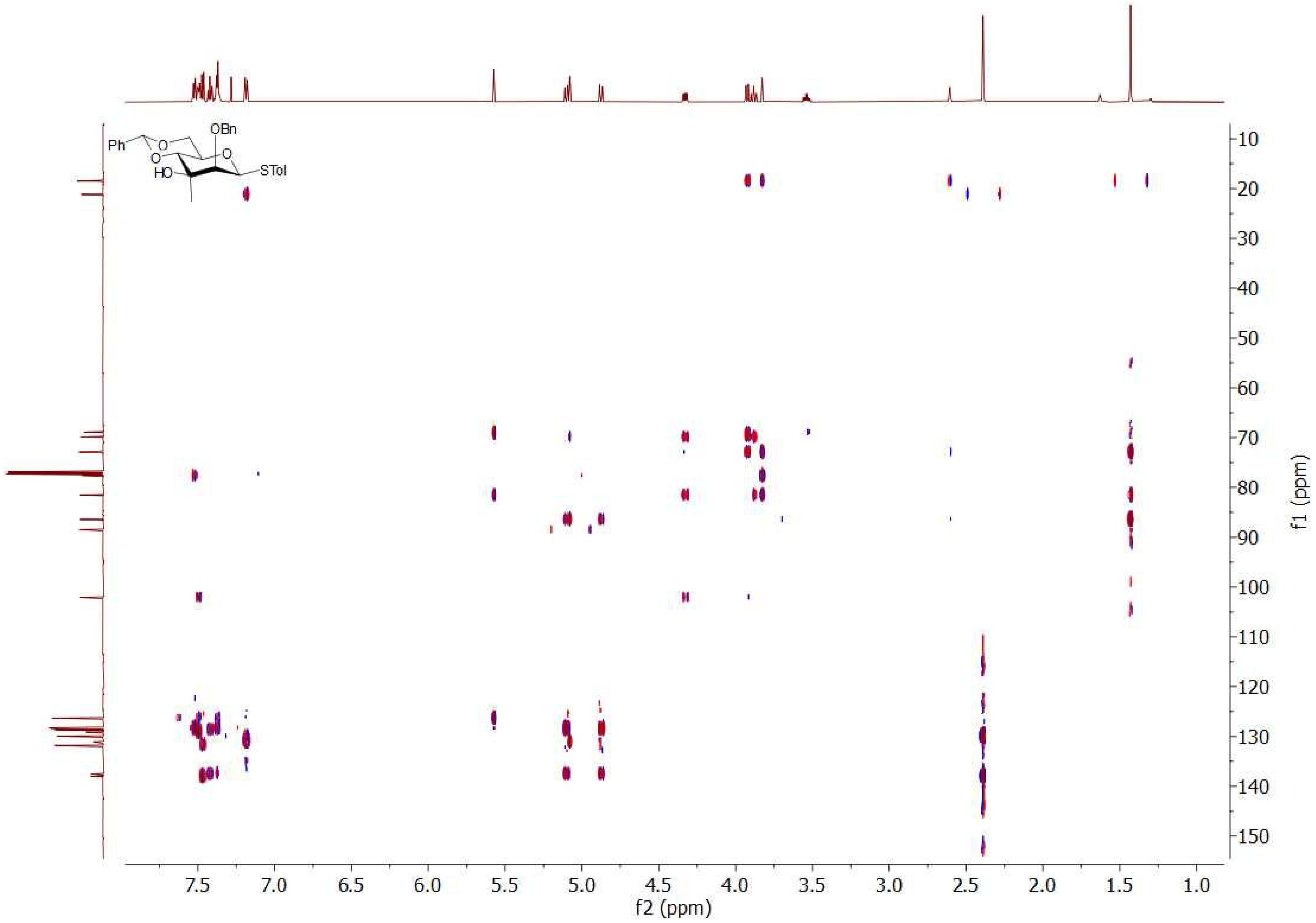

Figure S39. ^{1}H NMR (600 MHz, $CDCl_3$) spectrum of *p*-methylphenyl 3-*O*-benzoyl-2-*O*-benzyl-4,6-*O*-benzylidene-3-*C*-methyl-thio-β-D-mannopyranoside **38**:

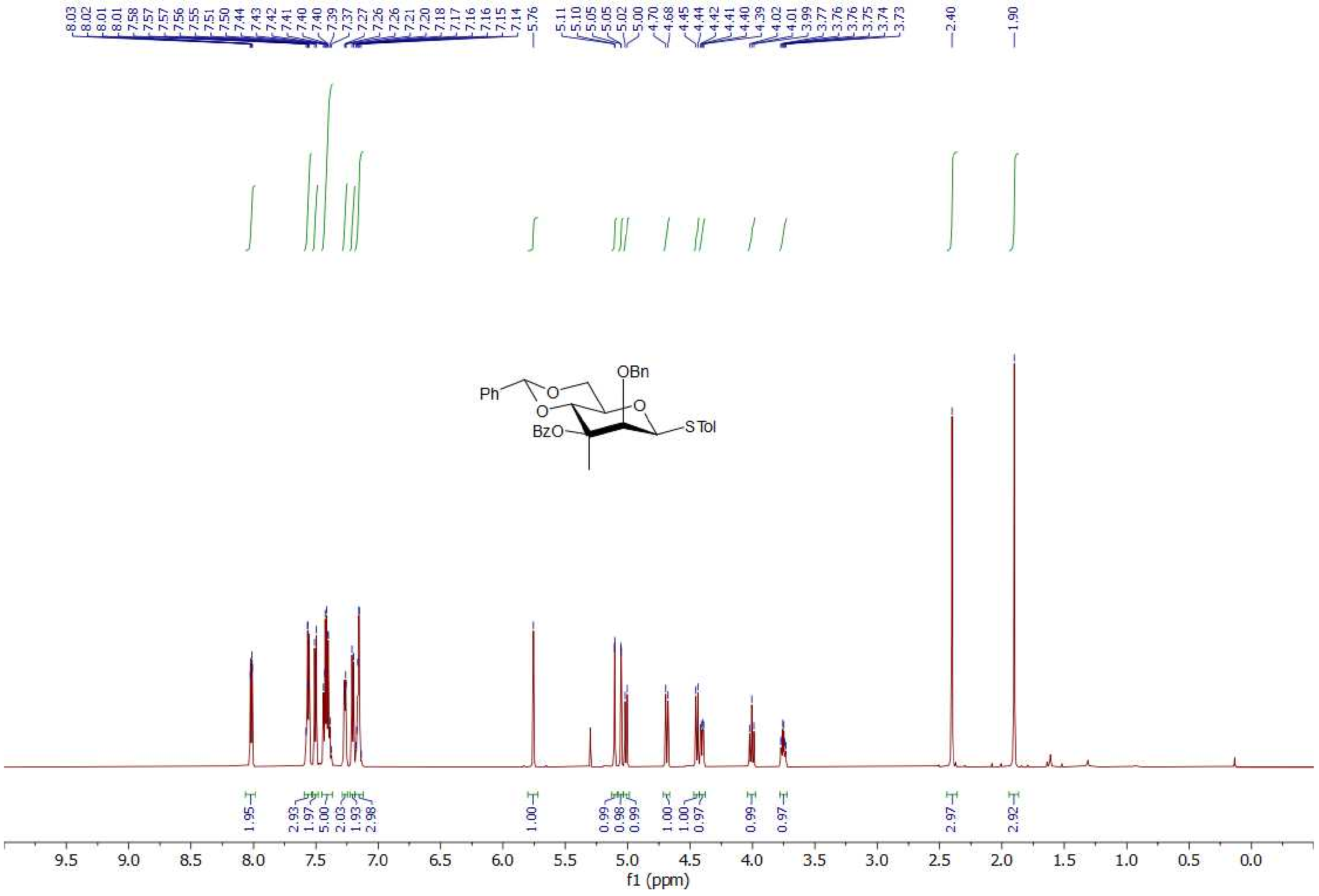

Figure S40. COSY NMR (600 MHz, $CDCl_3$) spectrum of *p*-methylphenyl 3-*O*-benzoyl-2-*O*-benzyl-4,6-*O*-benzylidene-3-*C*-methyl-thio-β-D-mannopyranoside **38**:

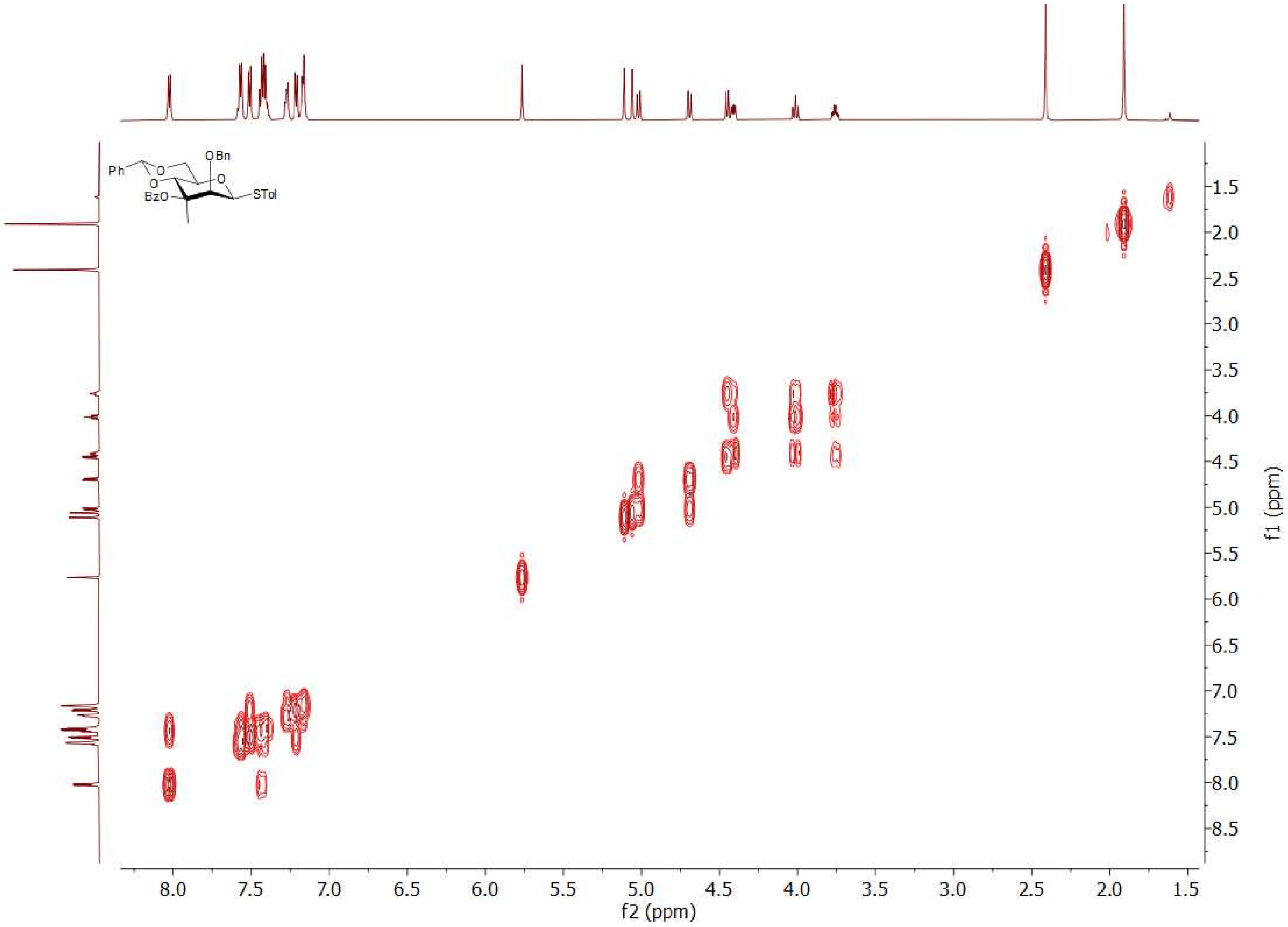

Figure S41. $^{13}C\{^1H\}$ NMR (151 MHz, $CDCl_3$) spectrum of *p*-methylphenyl 3-*O*-benzoyl-2-*O*-benzyl-4,6-*O*-benzylidene-3-*C*-methyl-thio-β-D-mannopyranoside **38**:

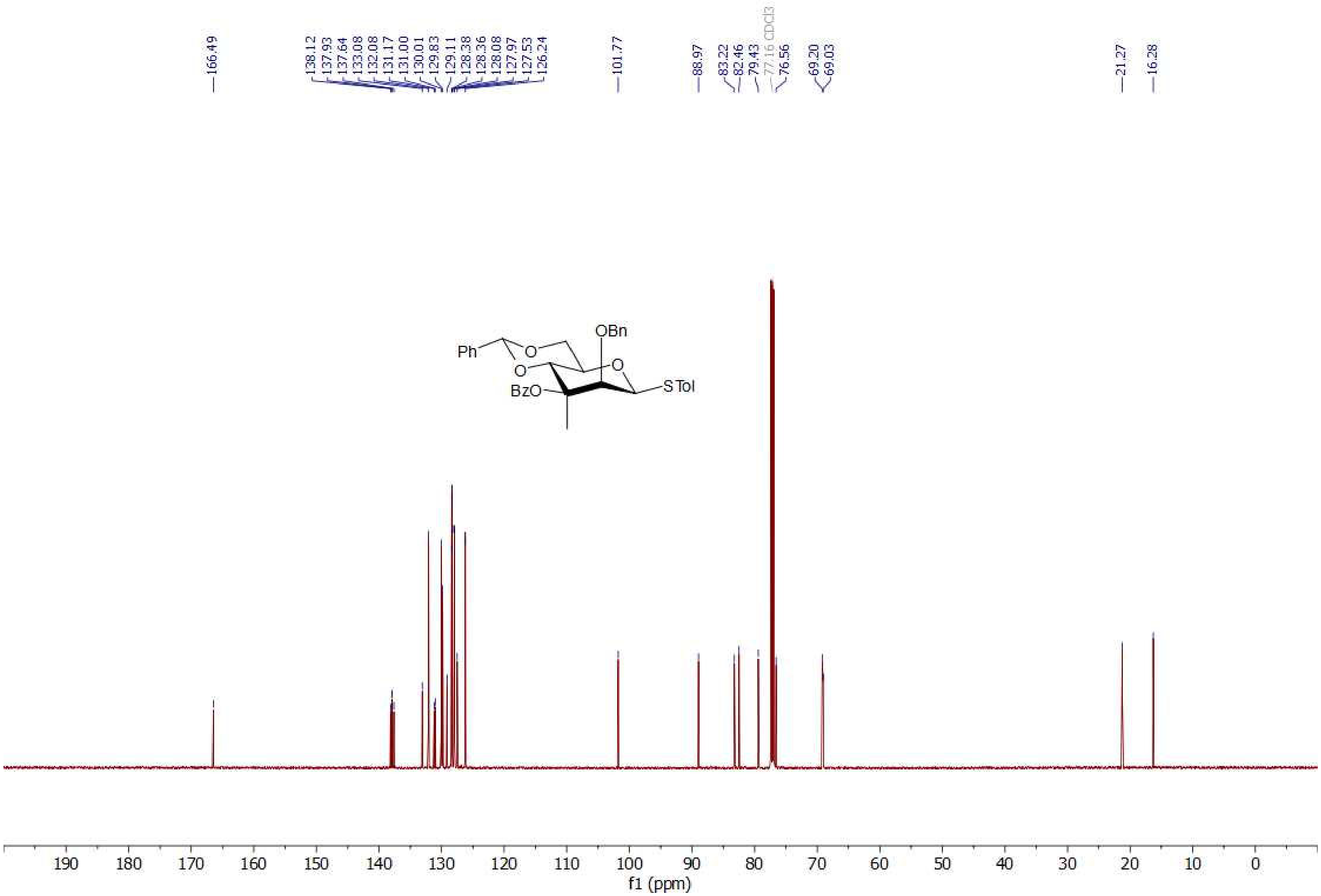

Figure S42. $^{13}C\{^{1}H\}$ DEPT NMR (151 MHz, $CDCl_3$) spectrum of *p*-methylphenyl 3-*O*-benzoyl-2-*O*-benzyl-4,6-*O*-benzylidene-3-*C*-methyl-thio-β-D-mannopyranoside **38**:

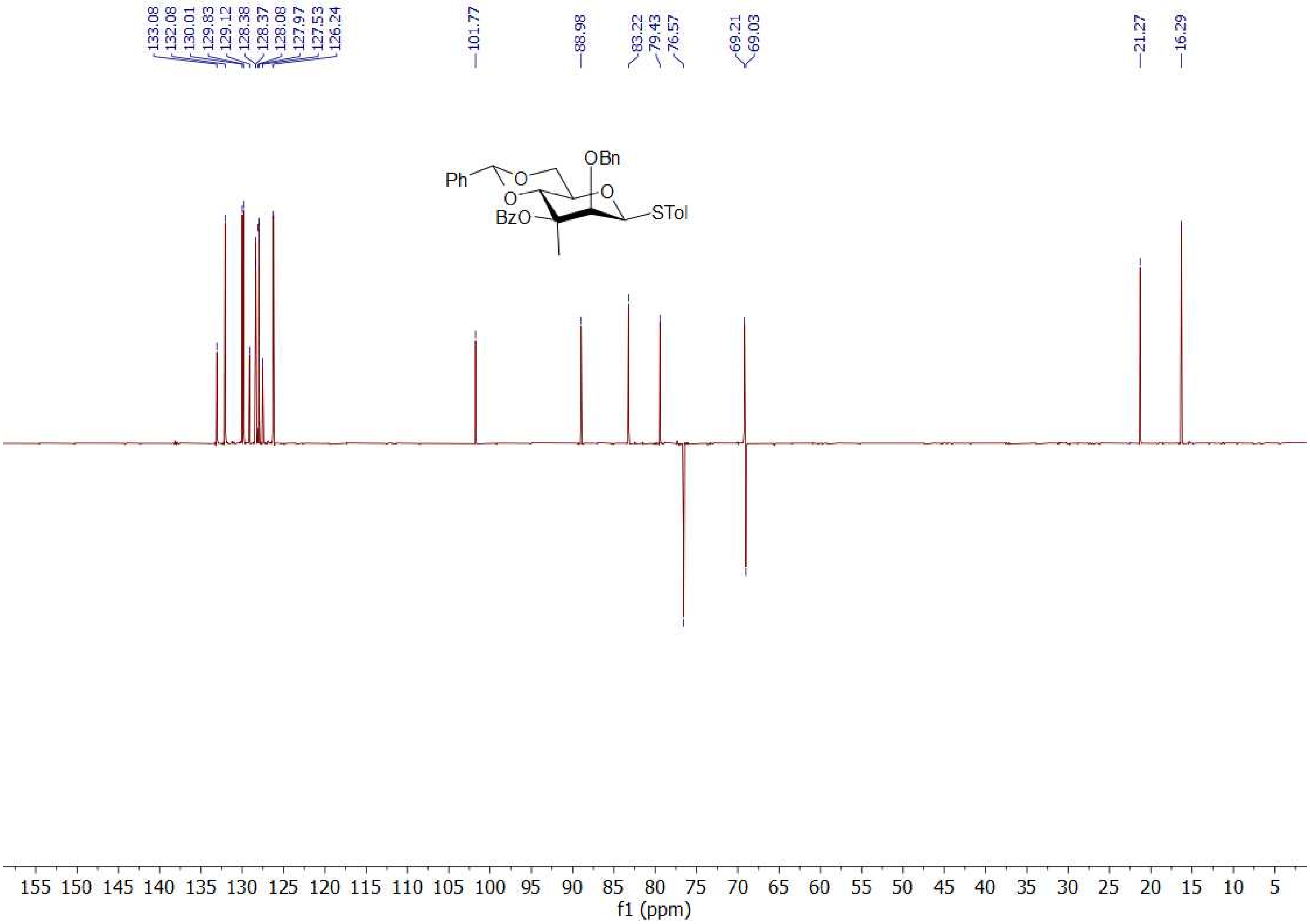

Figure S43. HSQC NMR (600 MHz, $CDCl_3$) spectrum of *p*-methylphenyl 3-*O*-benzoyl-2-*O*-benzyl-4,6-*O*-benzylidene-3-*C*-methyl-thio-β-D-mannopyranoside **38**:

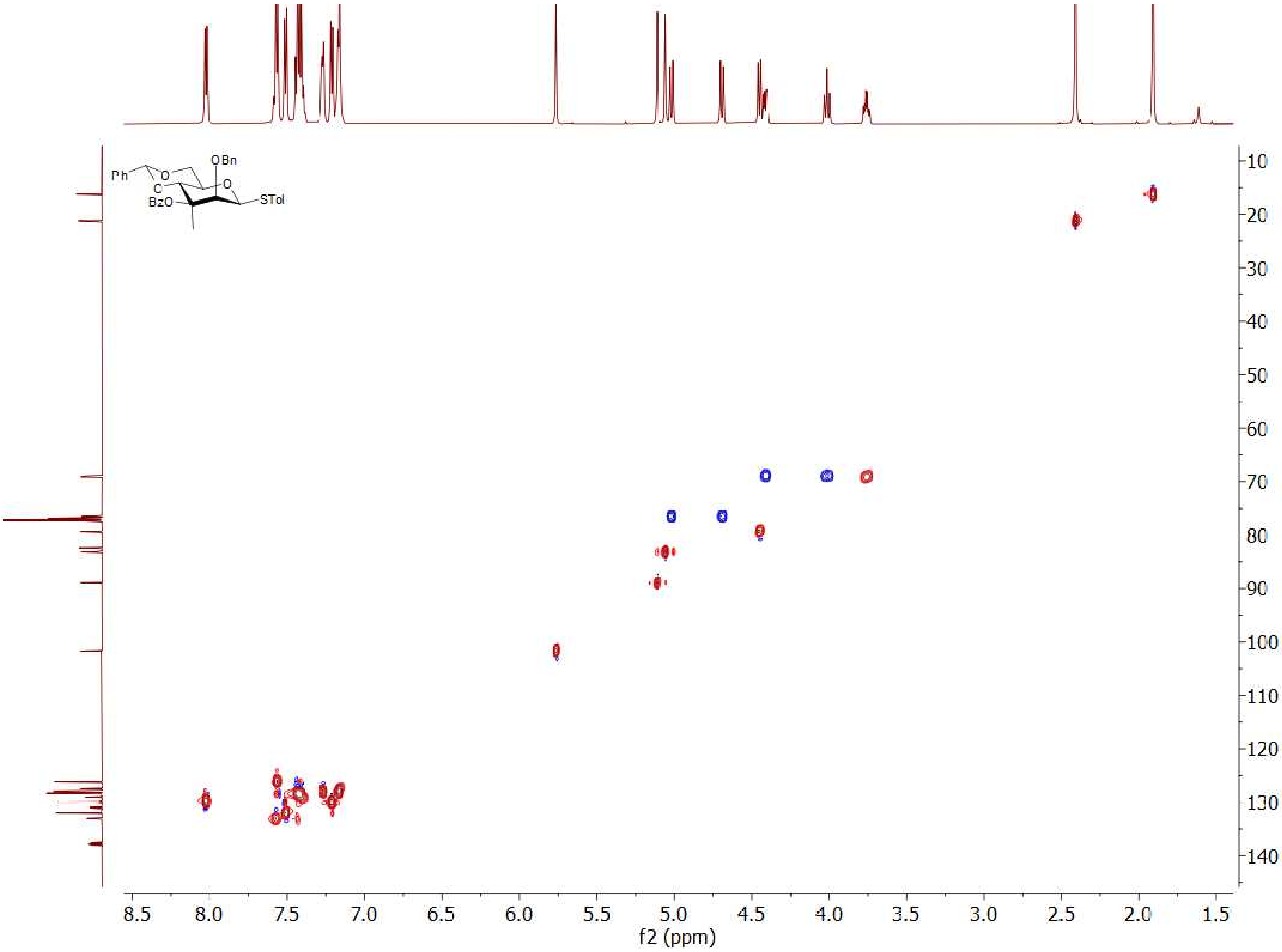

Figure S44. HMBC NMR (600 MHz, $CDCl_3$) spectrum of *p*-methylphenyl 3-*O*-benzoyl-2-*O*-benzyl-4,6-*O*-benzylidene-3-*C*-methyl-thio-β-D-mannopyranoside **38**:

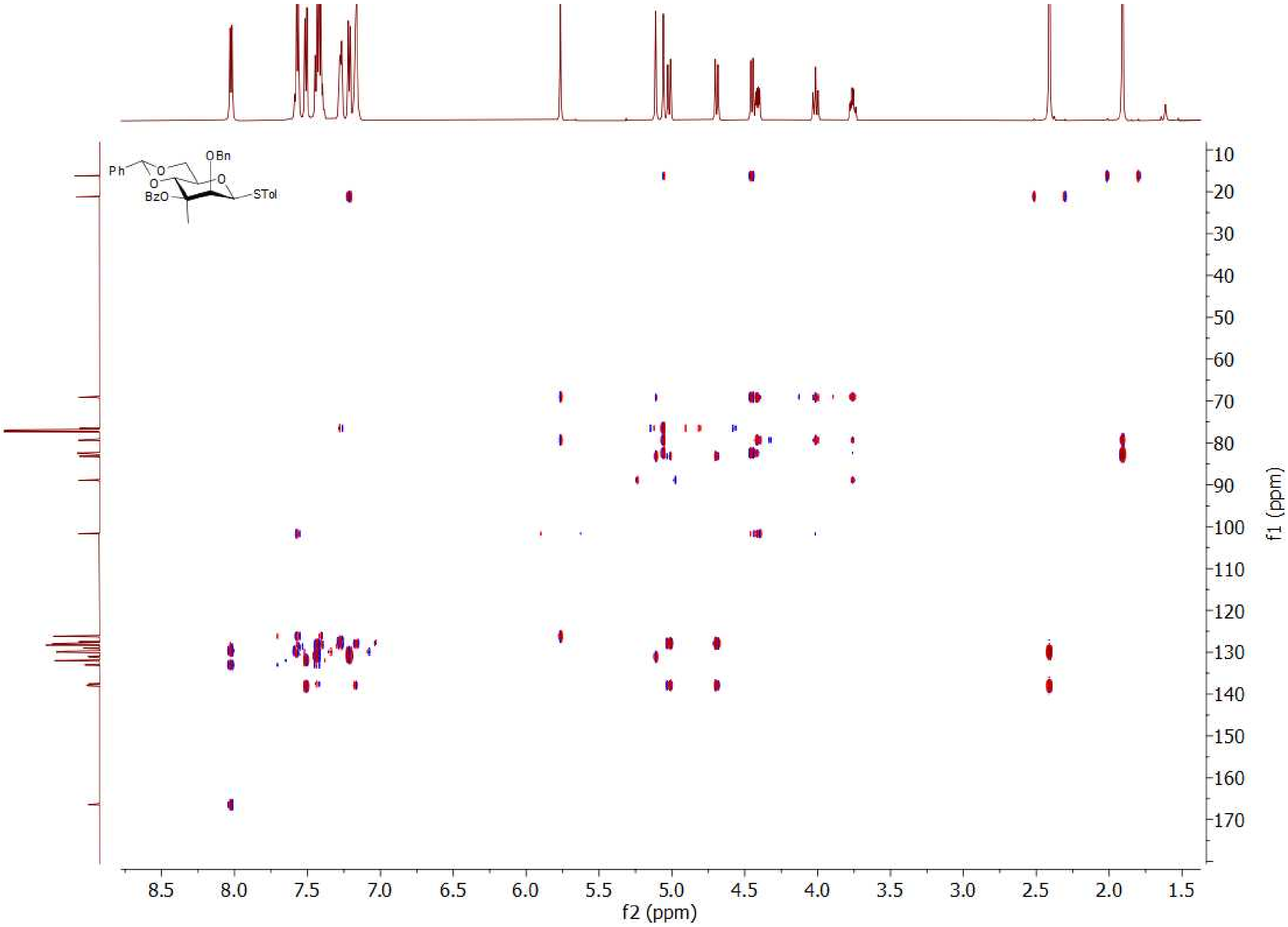

Figure S45. ^{1}H NMR (600 MHz, $CDCl_3$) spectrum of *p*-methylphenyl 3-*O*-(benzoyl-α-^{13}C)-2-*O*-benzyl-4,6-*O*-benzylidene-3-*C*-methyl-thio-β-D-mannopyranoside **^{13}C-38**:

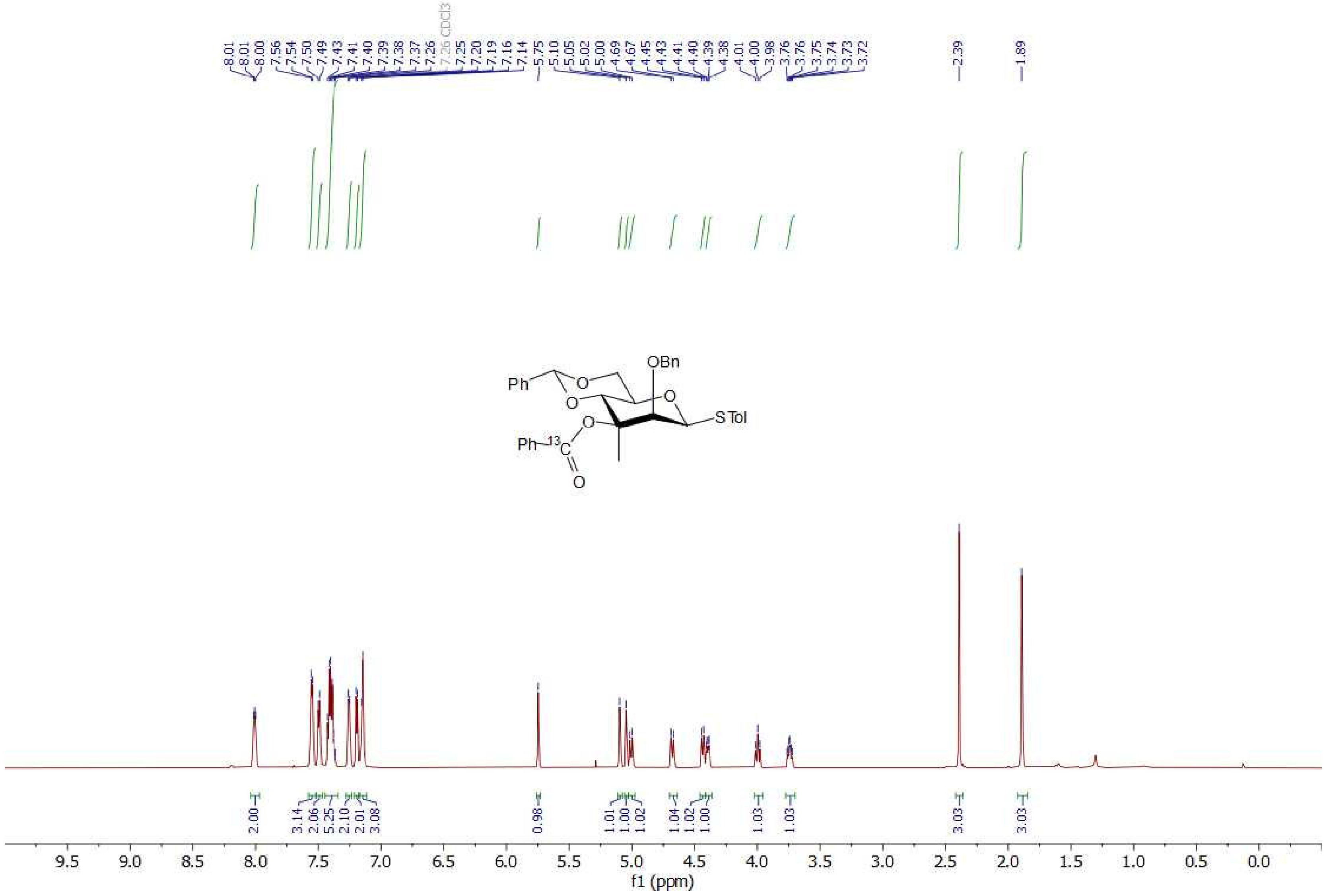

Figure S46. COSY NMR (600 MHz, $CDCl_3$) spectrum of *p*-methylphenyl 3-*O*-(benzoyl-α-^{13}C)-2-*O*-benzyl-4,6-*O*-benzylidene-3-*C*-methyl-thio-β-D-mannopyranoside **^{13}C-38**:

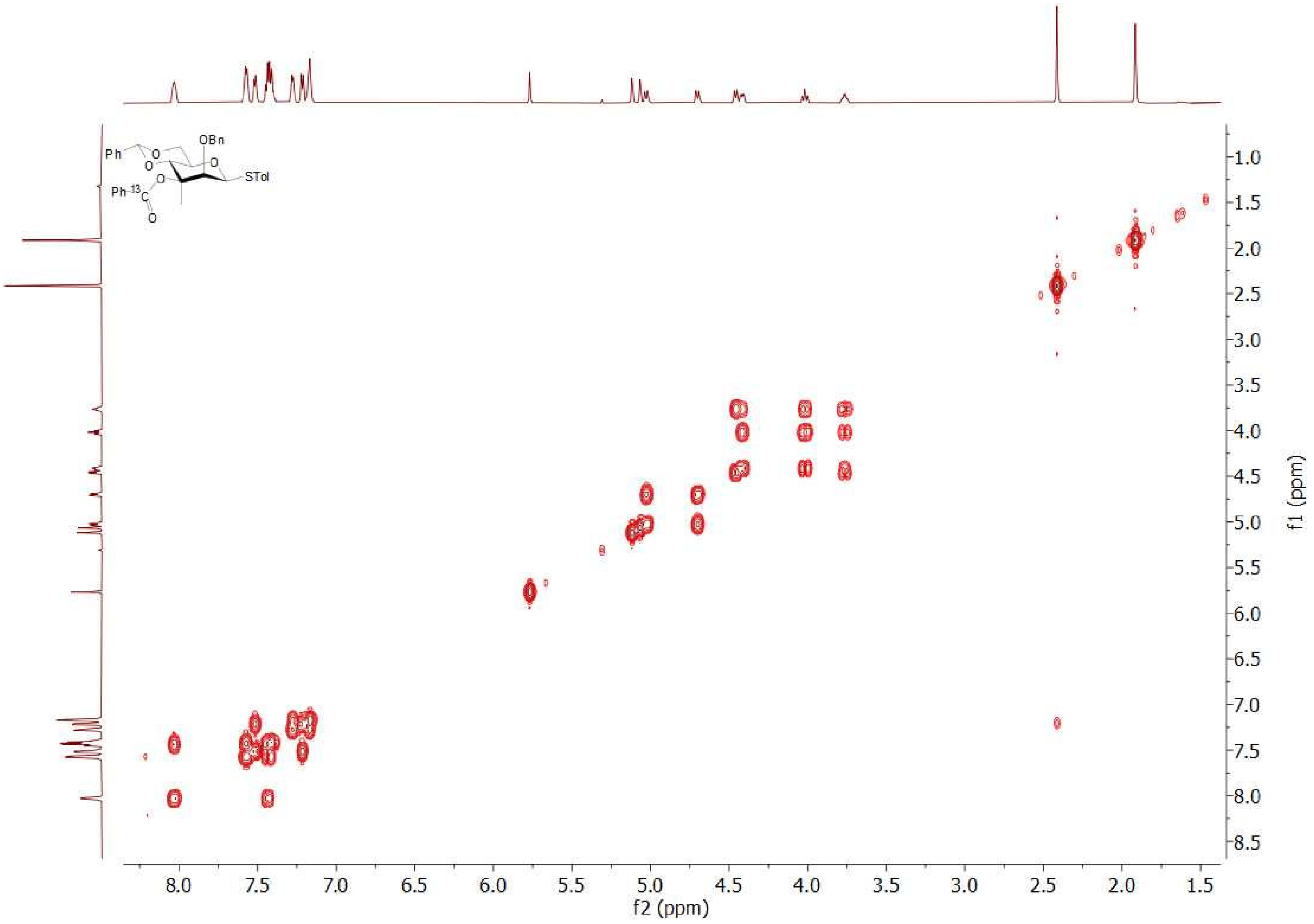

Figure S47. $^{13}C\{^{1}H\}$ NMR (151 MHz, $CDCl_3$) spectrum of *p*-methylphenyl 3-*O*-(benzoyl-α-^{13}C)-2-*O*-benzyl-4,6-*O*-benzylidene-3-*C*-methyl-thio-β-D-mannopyranoside **^{13}C-38**:

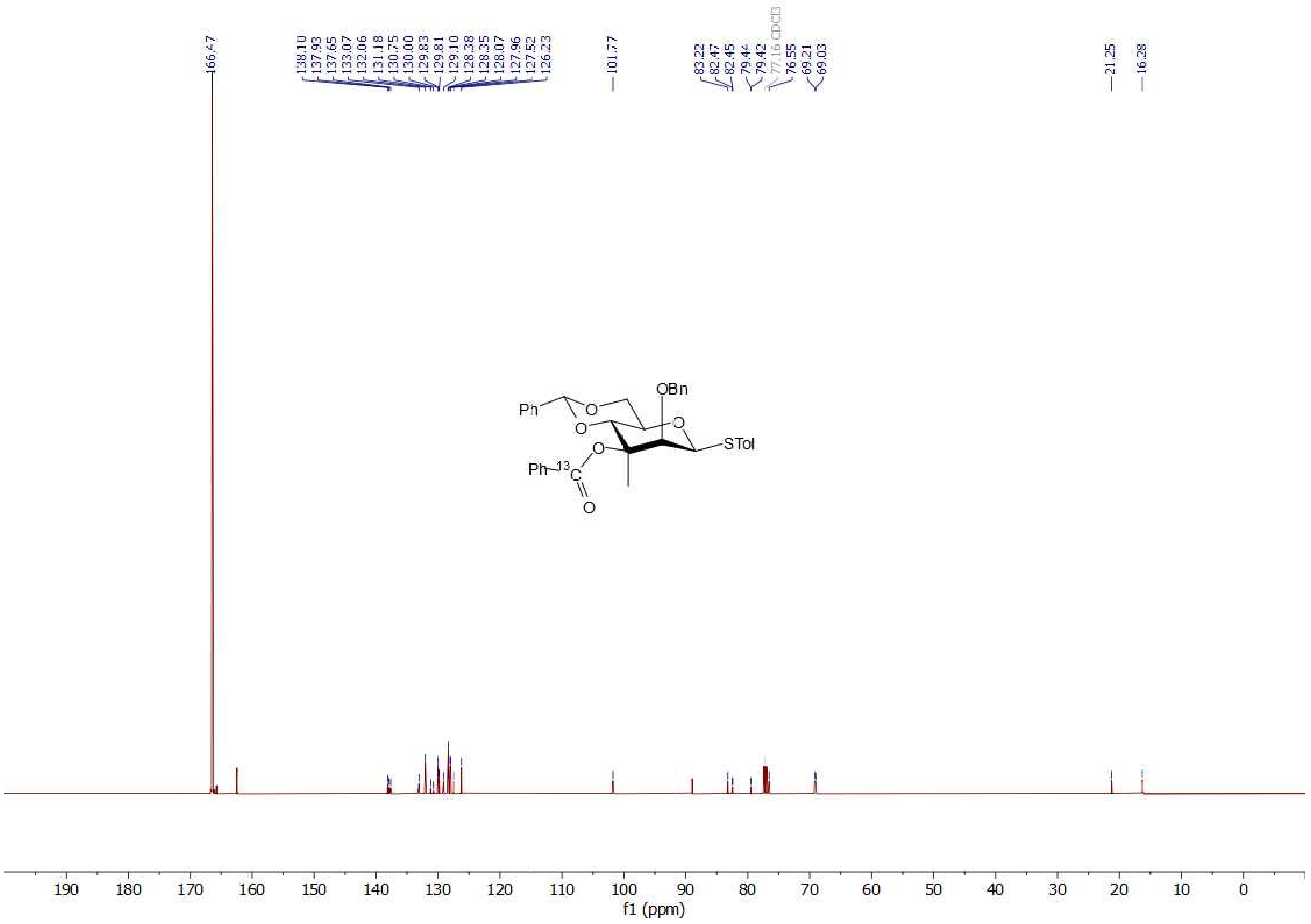

Figure S48. $^{13}C\{^1H\}$ DEPT NMR (151 MHz, $CDCl_3$) spectrum of *p*-methylphenyl 3-*O*-(benzoyl-α-^{13}C)-2-*O*-benzyl-4,6-*O*-benzylidene-3-*C*-methyl-thio-β-D-mannopyranoside **^{13}C-38**:

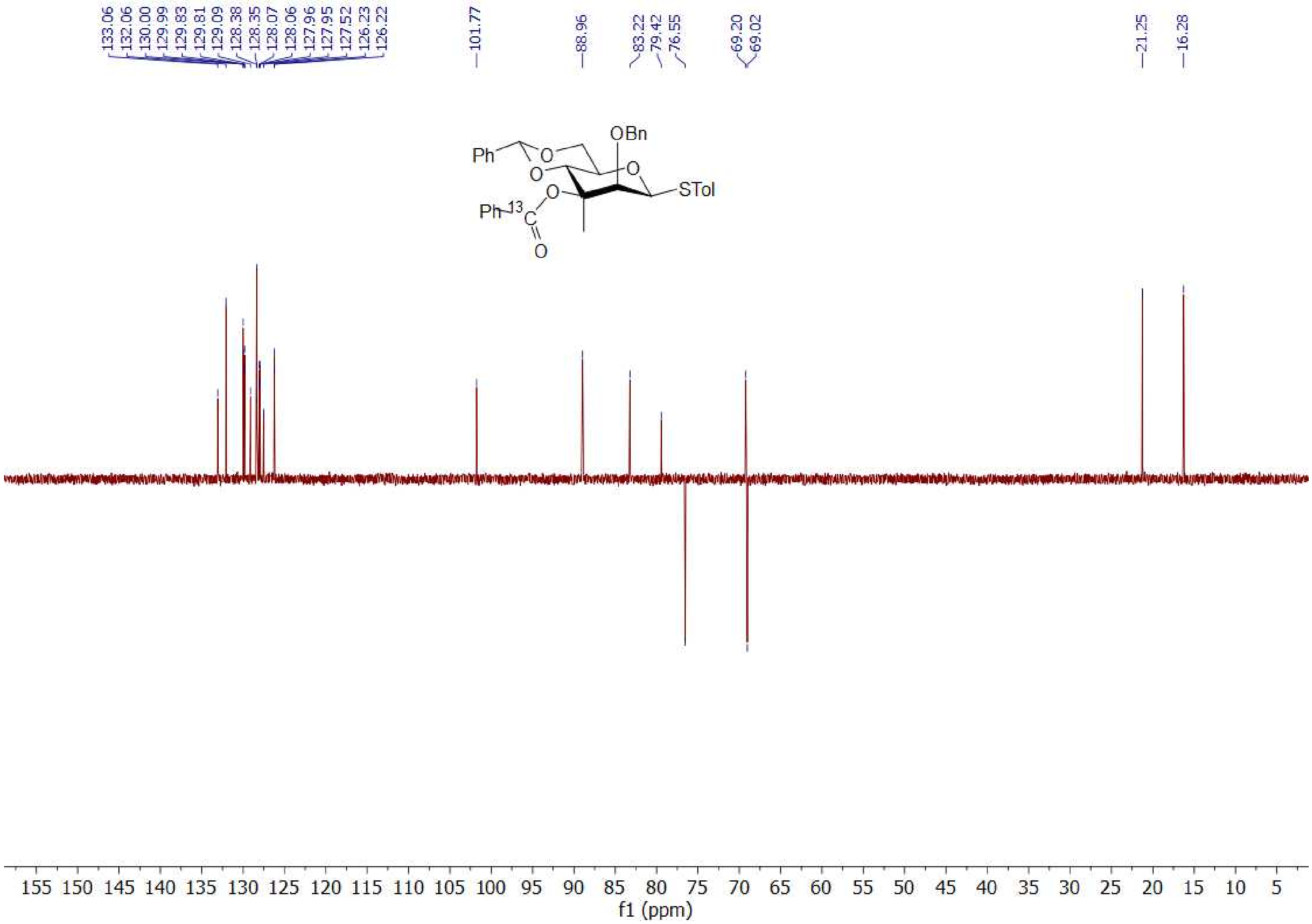

Figure S49. HSQC NMR (600 MHz, $CDCl_3$) spectrum of *p*-methylphenyl 3-*O*-(benzoyl-α-^{13}C)-2-*O*-benzyl-4,6-*O*-benzylidene-3-*C*-methyl-thio-β-D-mannopyranoside **^{13}C-38**:

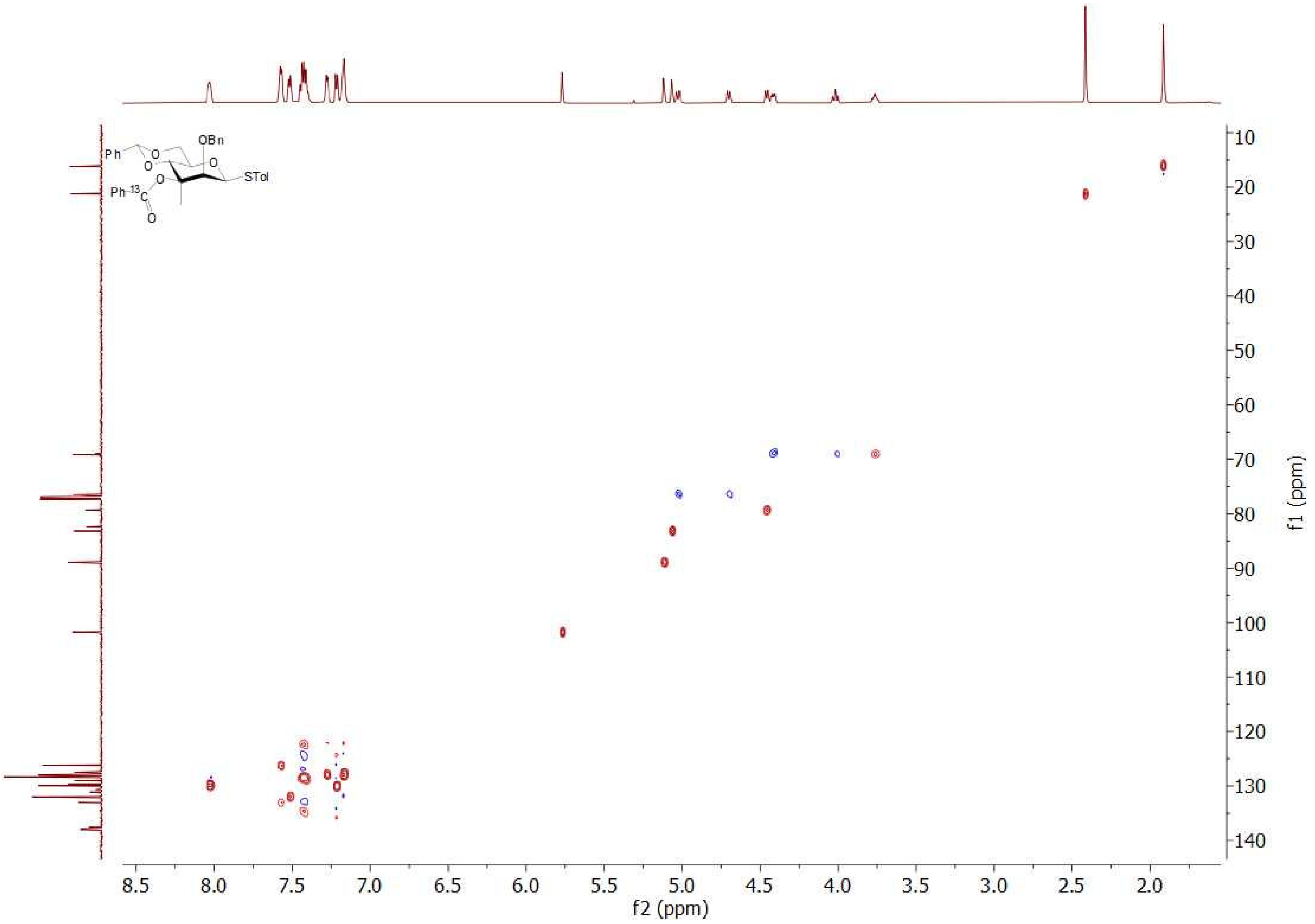

Figure S50. HMBC NMR (600 MHz, $CDCl_3$) spectrum of *p*-methylphenyl 3-*O*-(benzoyl-α-^{13}C)-2-*O*-benzyl-4,6-*O*-benzylidene-3-*C*-methyl-thio-β-D-mannopyranoside **^{13}C-38**:

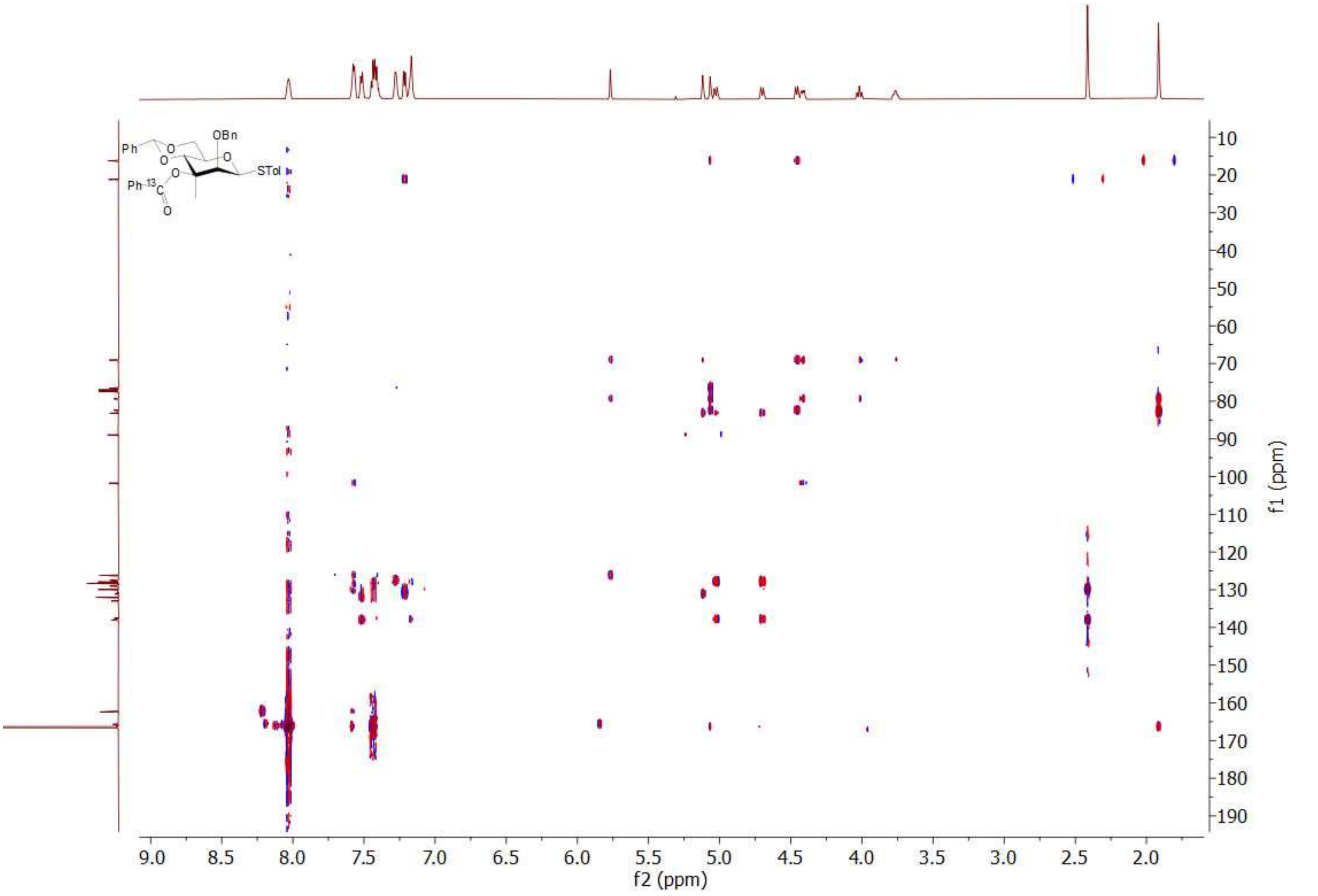

Figure S51. ^{1}H NMR (600 MHz, $CDCl_3$) spectrum of *p*-methylphenyl 2-*O*-benzyl-4,6-*O*-benzylidene-3-*O*-*p*-nitrobenzoyl-3-*C*-methyl-thio-β-D-mannopyranoside **39**:

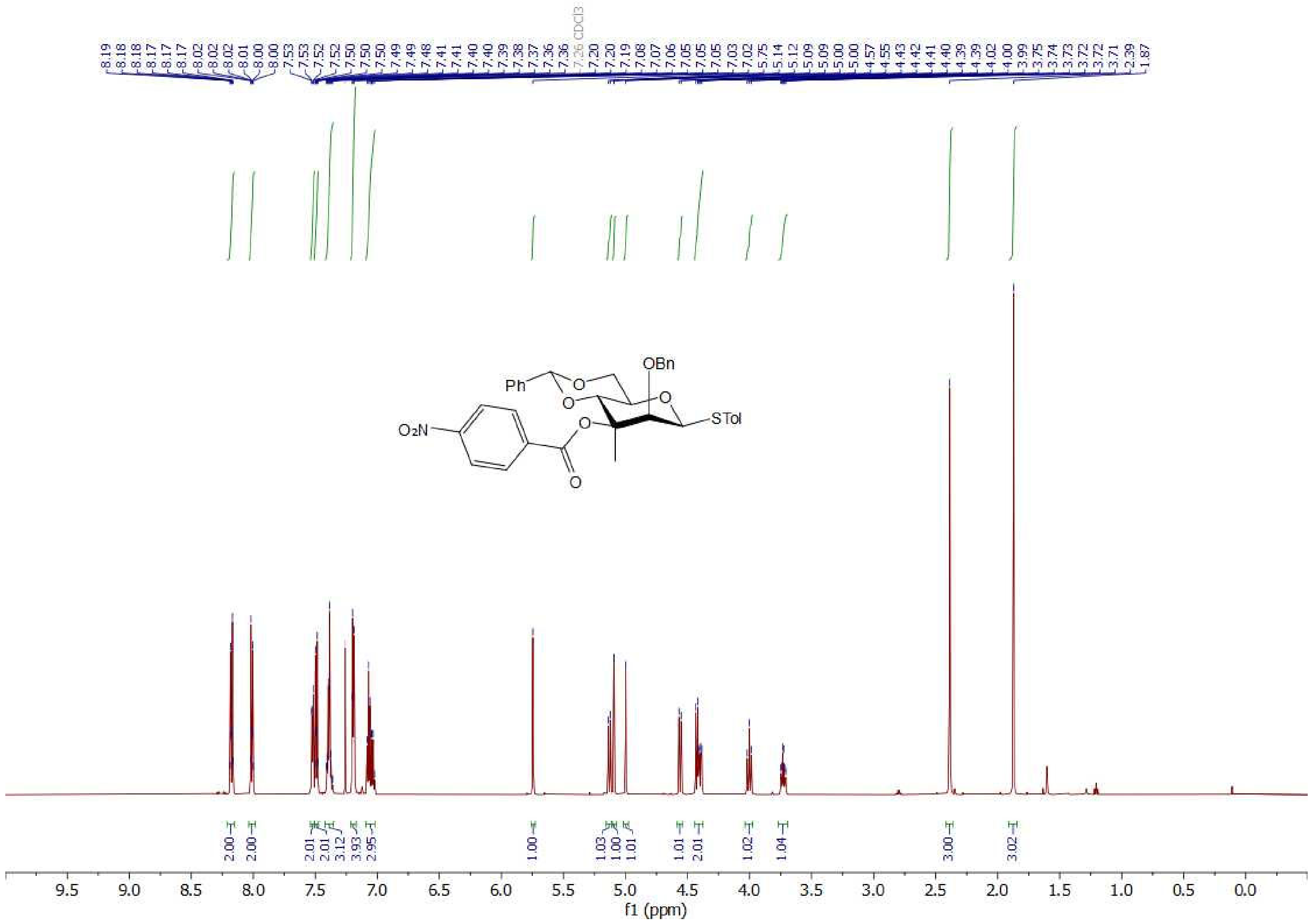

Figure S52. COSY NMR (600 MHz, $CDCl_3$) spectrum of *p*-methylphenyl 2-*O*-benzyl-4,6-*O*-benzylidene-3-*O*-*p*-nitrobenzoyl-3-*C*-methyl-thio-β-D-mannopyranoside **39**:

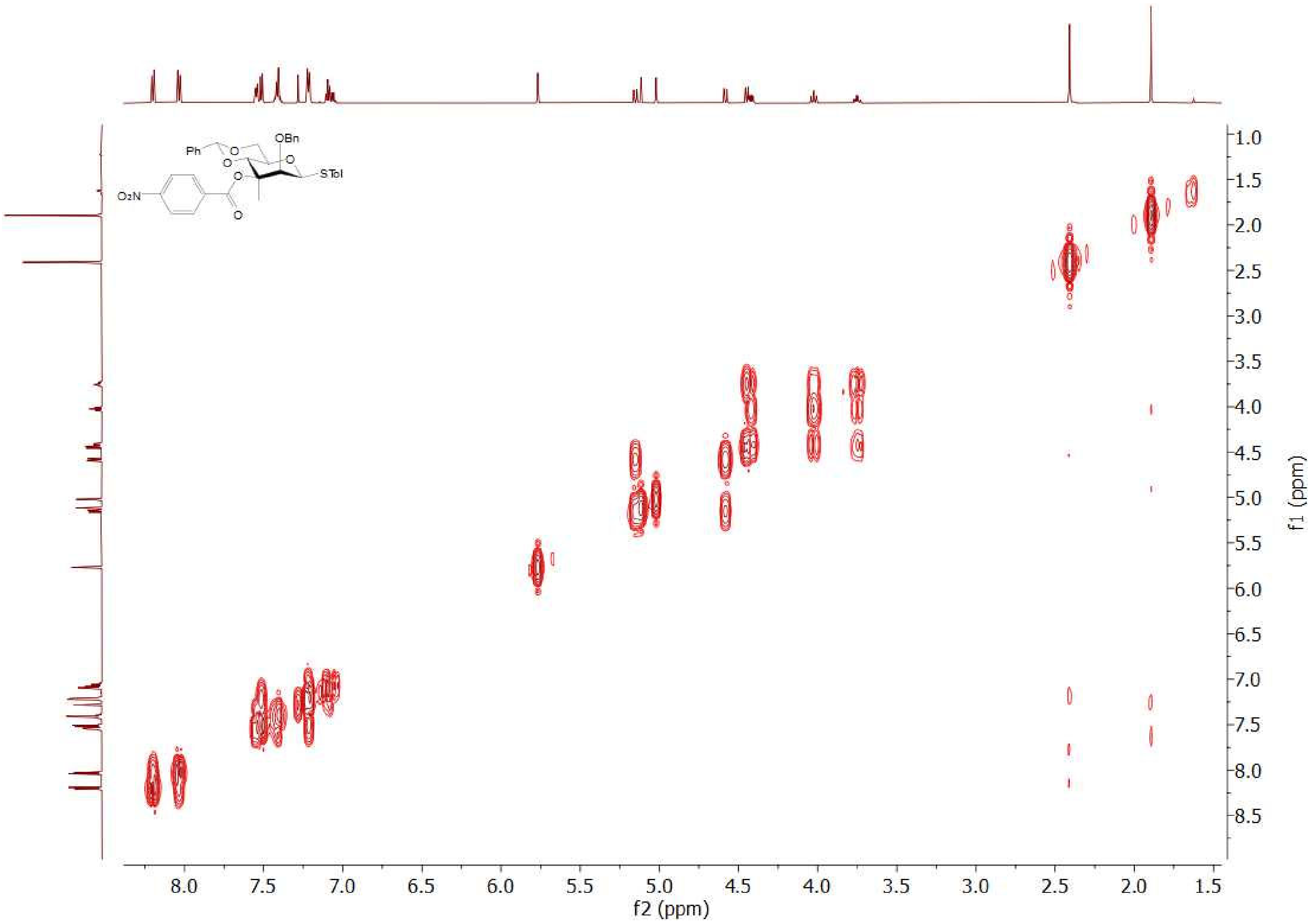

Figure S53. $^{13}C\{^1H\}$ NMR (151 MHz, $CDCl_3$) spectrum of *p*-methylphenyl 2-*O*-benzyl-4,6-*O*-benzylidene-3-*O*-*p*-nitrobenzoyl-3-*C*-methyl-thio-β-D-mannopyranoside **39**:

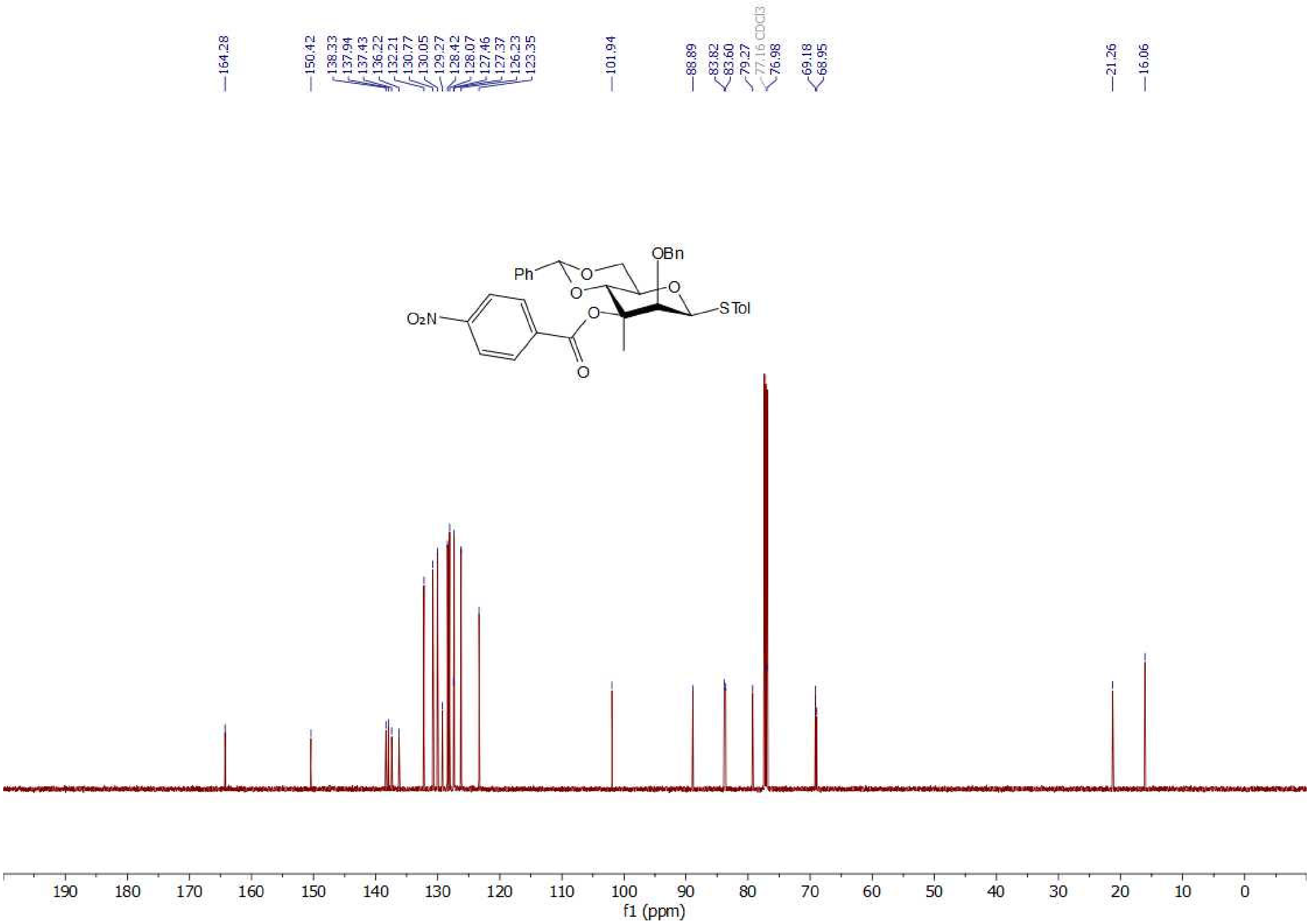

Figure S54. $^{13}C\{^1H\}$ DEPT NMR (151 MHz, $CDCl_3$) spectrum of *p*-methylphenyl 2-*O*-benzyl-4,6-*O*-benzylidene-3-*O*-*p*-nitrobenzoyl-3-*C*-methyl-thio-β-D-mannopyranoside **39**:

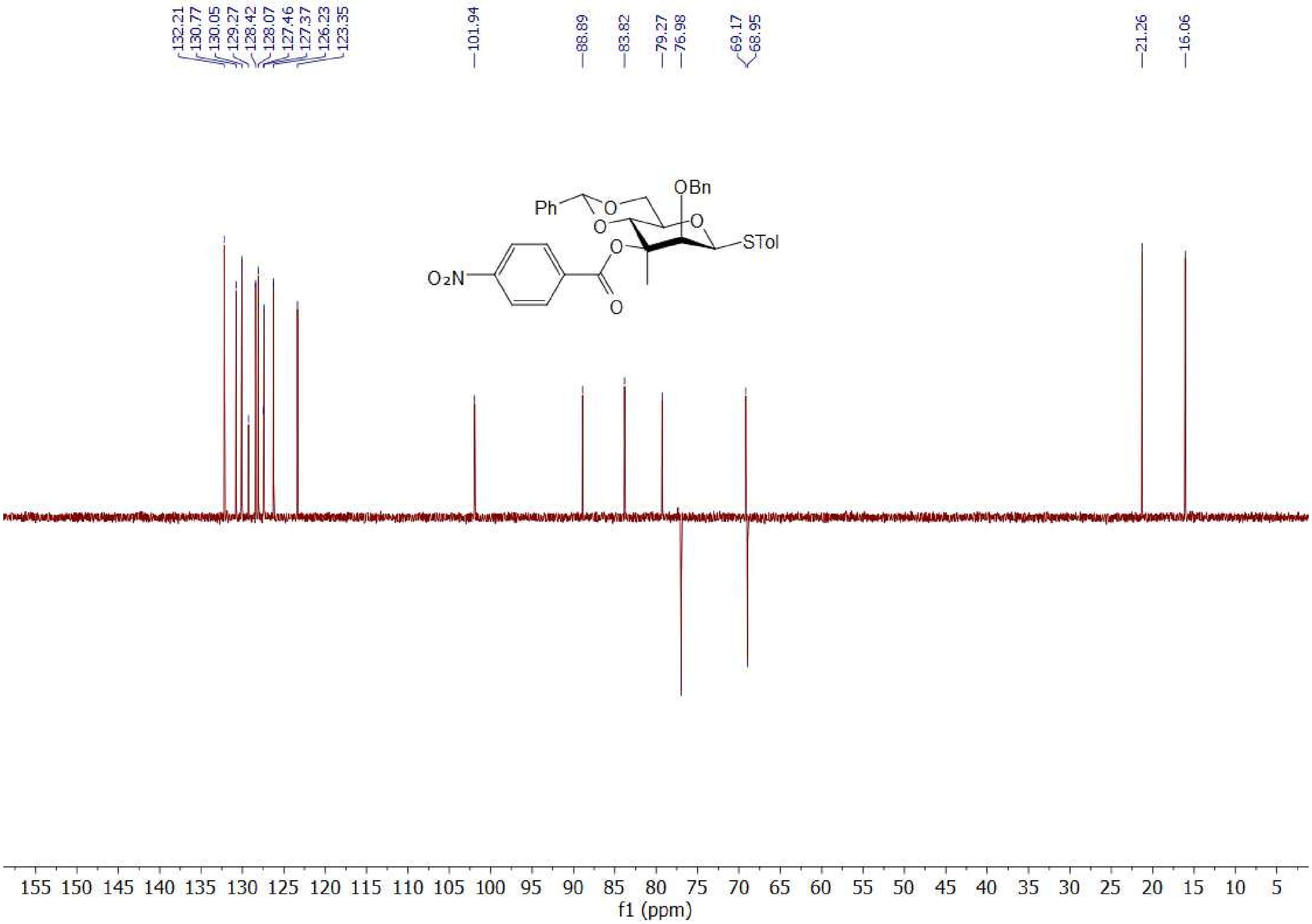

Figure S55. HSQC NMR (600 MHz, $CDCl_3$) spectrum of *p*-methylphenyl 2-*O*-benzyl-4,6-*O*-benzylidene-3-*O*-*p*-nitrobenzoyl-3-*C*-methyl-thio-β-D-mannopyranoside **39**:

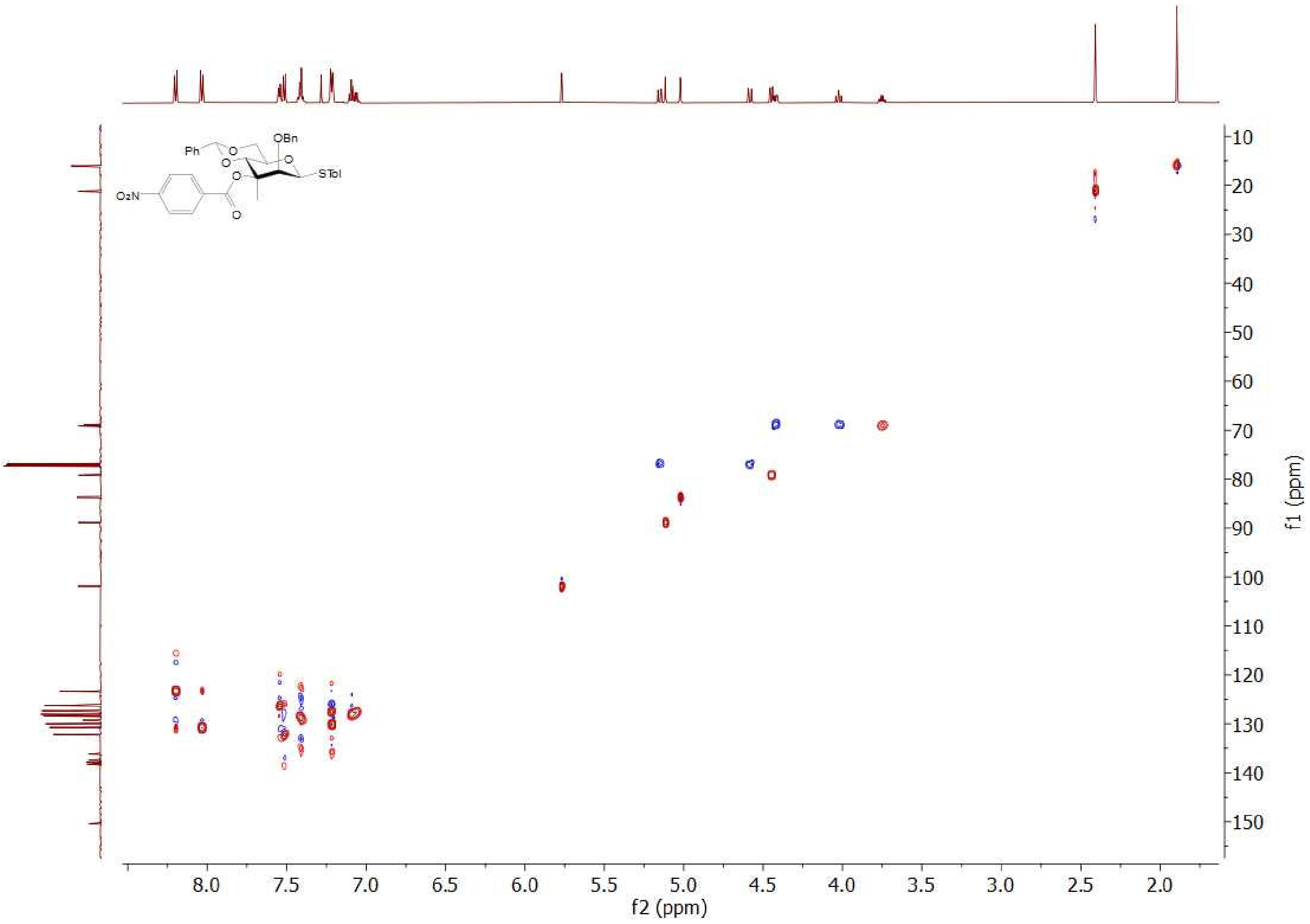

Figure S56. HMBC NMR (600 MHz, $CDCl_3$) spectrum of *p*-methylphenyl 2-*O*-benzyl-4,6-*O*-benzylidene-3-*O*-*p*-nitrobenzoyl-3-*C*-methyl-thio-β-D-mannopyranoside **39**:

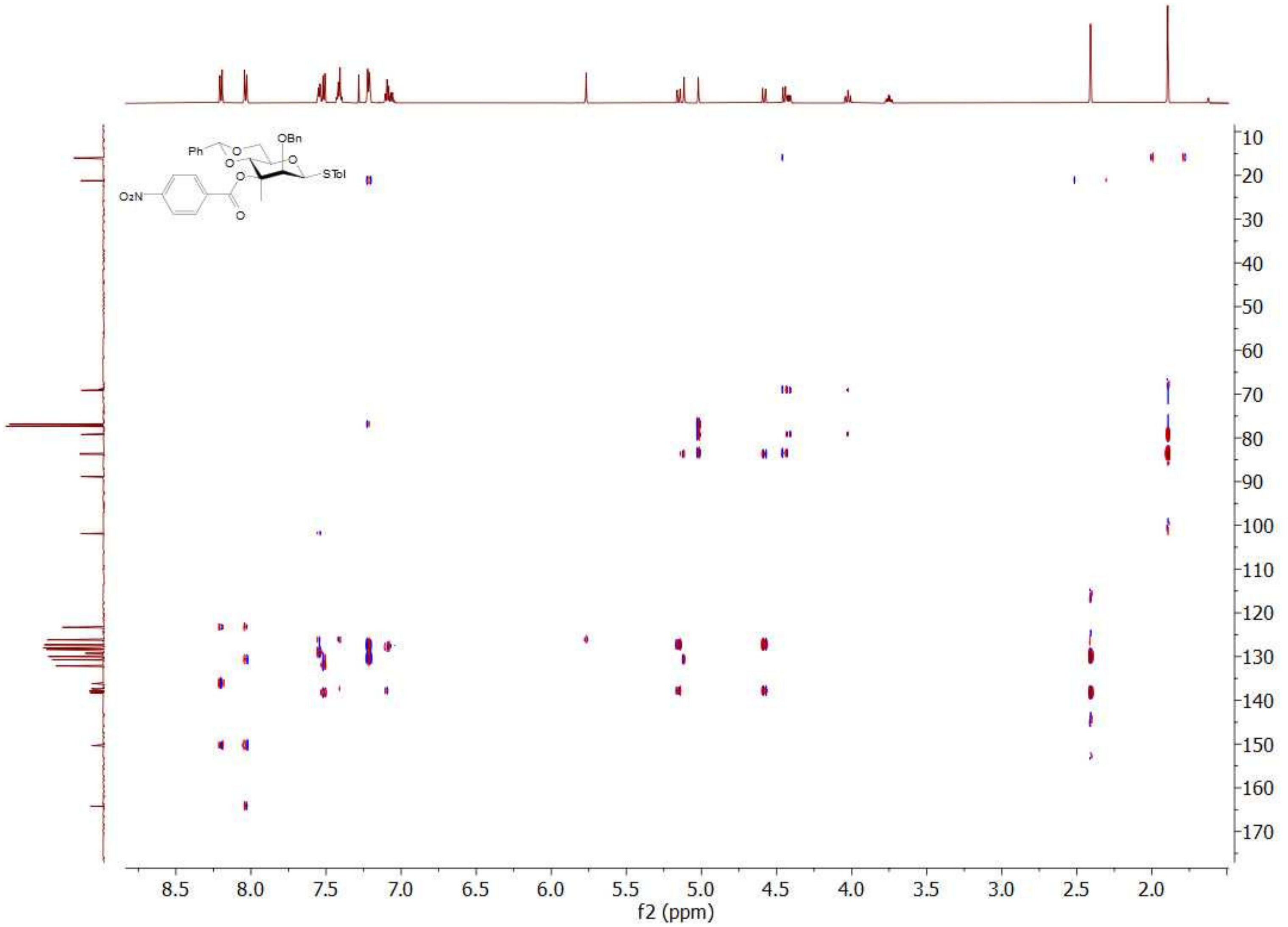

Figure S57. ^{1}H NMR (600 MHz, $CDCl_3$) spectrum of *p*-methylphenyl 3-*O*-benzoyl-2-*O*-benzyl-4,6-*O*-benzylidene-3-*C*-methyl-thio-β-D-mannopyranoside *S*-oxide **40** (Less-polar diastereoisomer):

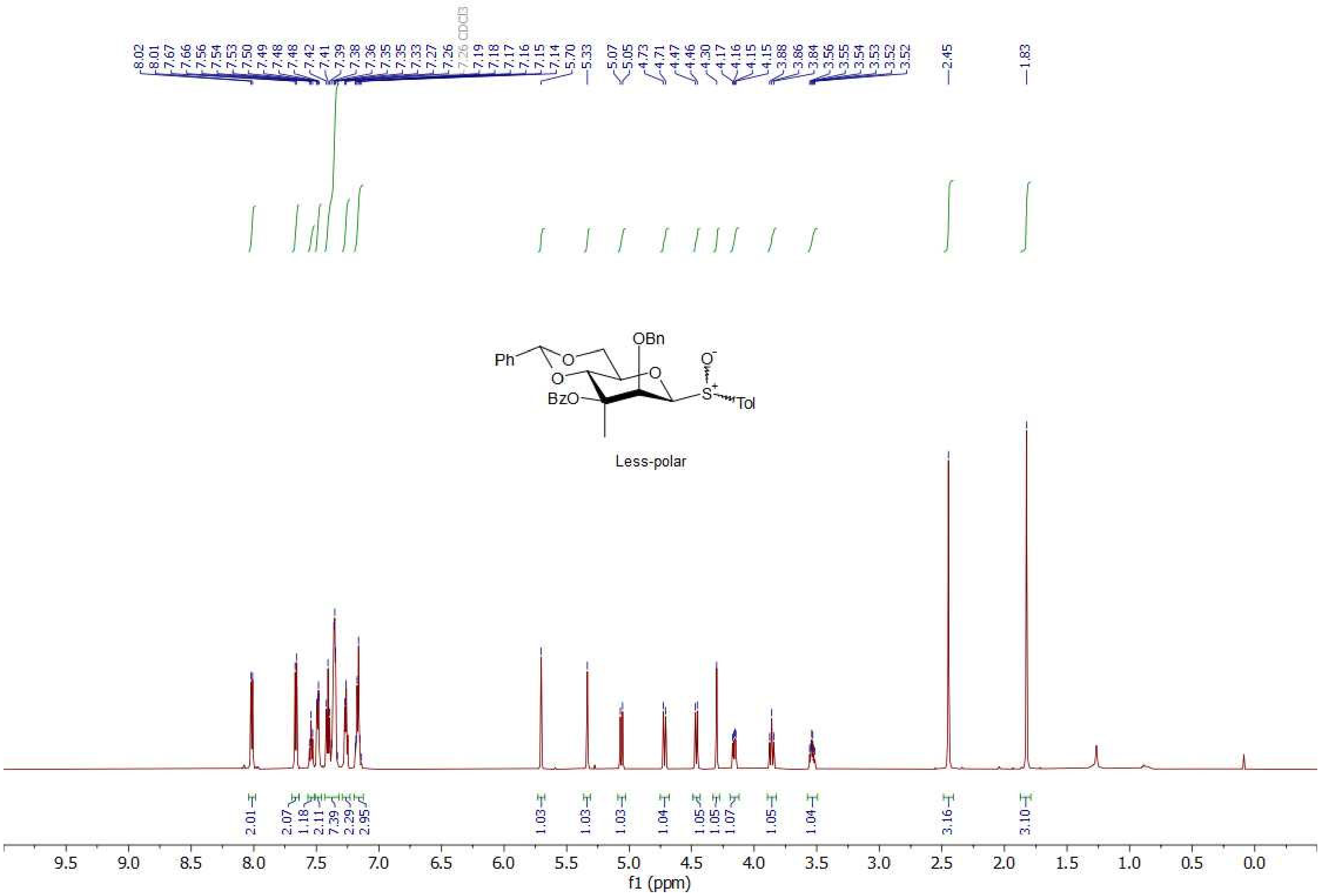

Figure S58. COSY NMR (600 MHz, $CDCl_3$) spectrum of *p*-methylphenyl 3-*O*-benzoyl-2-*O*-benzyl-4,6-*O*-benzylidene-3-*C*-methyl-thio-β-D-mannopyranoside *S*-oxide **40** (Less-polar diastereoisomer):

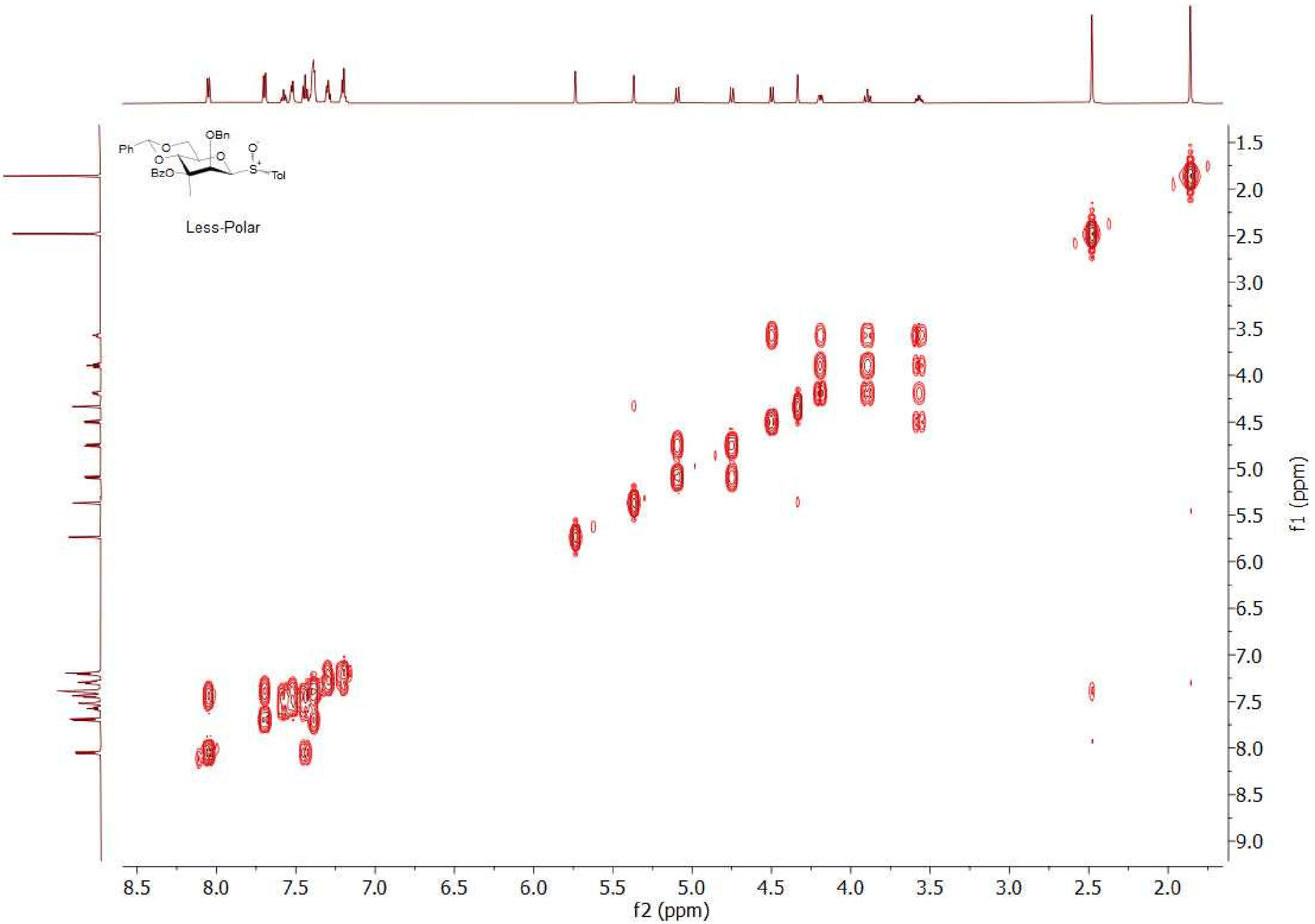

Figure S59. $^{13}C\{^{1}H\}$ NMR (151 MHz, $CDCl_3$) spectrum of *p*-methylphenyl 3-*O*-benzoyl-2-*O*-benzyl-4,6-*O*-benzylidene-3-*C*-methyl-thio-β-D-mannopyranoside *S*-oxide **40** (Less-polar diastereoisomer):

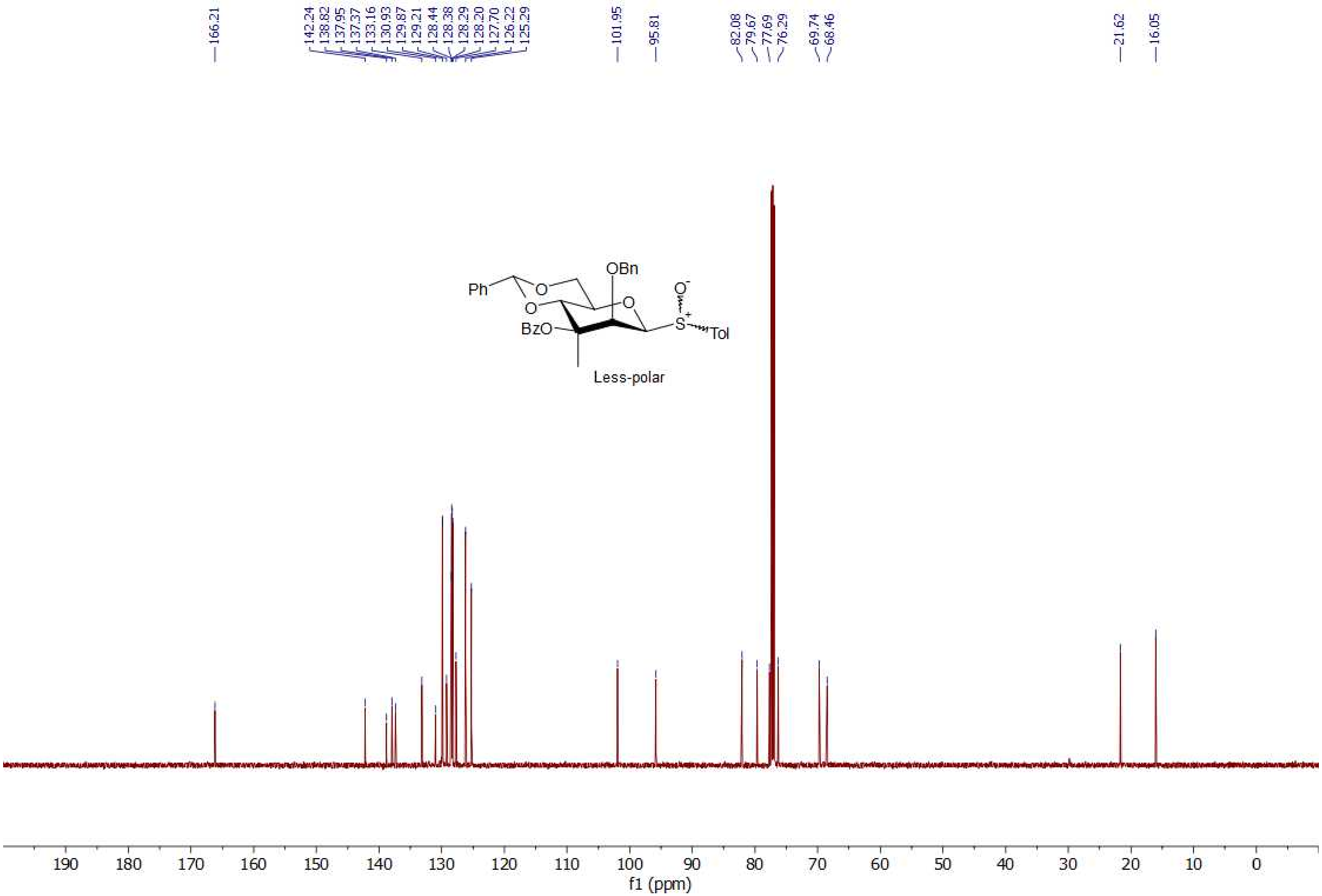

Figure S60. $^{13}C\{^{1}H\}$ DEPT NMR (151 MHz, $CDCl_3$) spectrum of *p*-methylphenyl 3-*O*-benzoyl-2-*O*-benzyl-4,6-*O*-benzylidene-3-*C*-methyl-thio-β-D-mannopyranoside *S*-oxide **40** (Less-polar diastereoisomer):

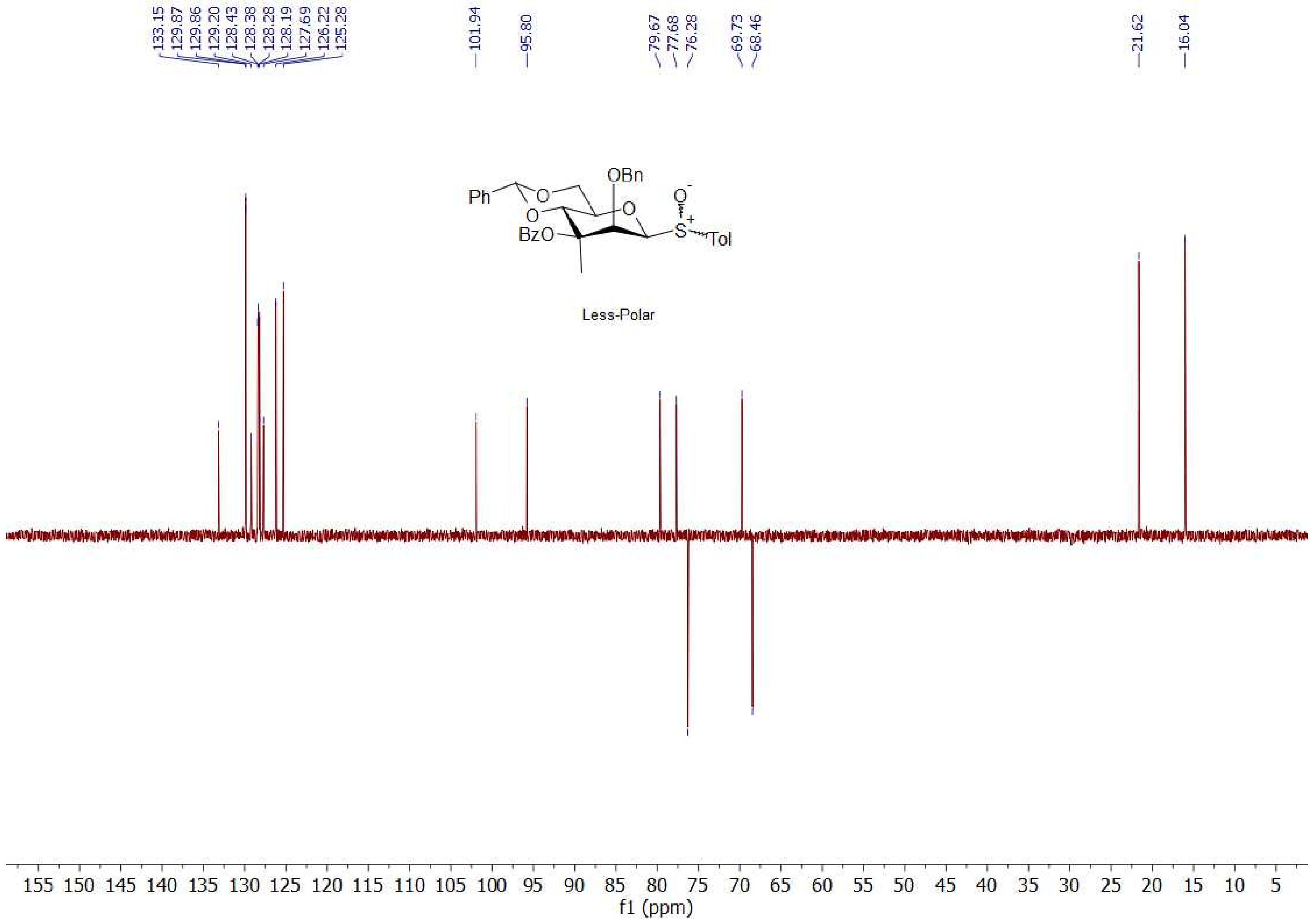

Figure S61. HSQC NMR (600 MHz, $CDCl_3$) spectrum of *p*-methylphenyl 3-*O*-benzoyl-2-*O*-benzyl-4,6-*O*-benzylidene-3-*C*-methyl-thio-β-D-mannopyranoside *S*-oxide **40** (Less-polar diastereoisomer):

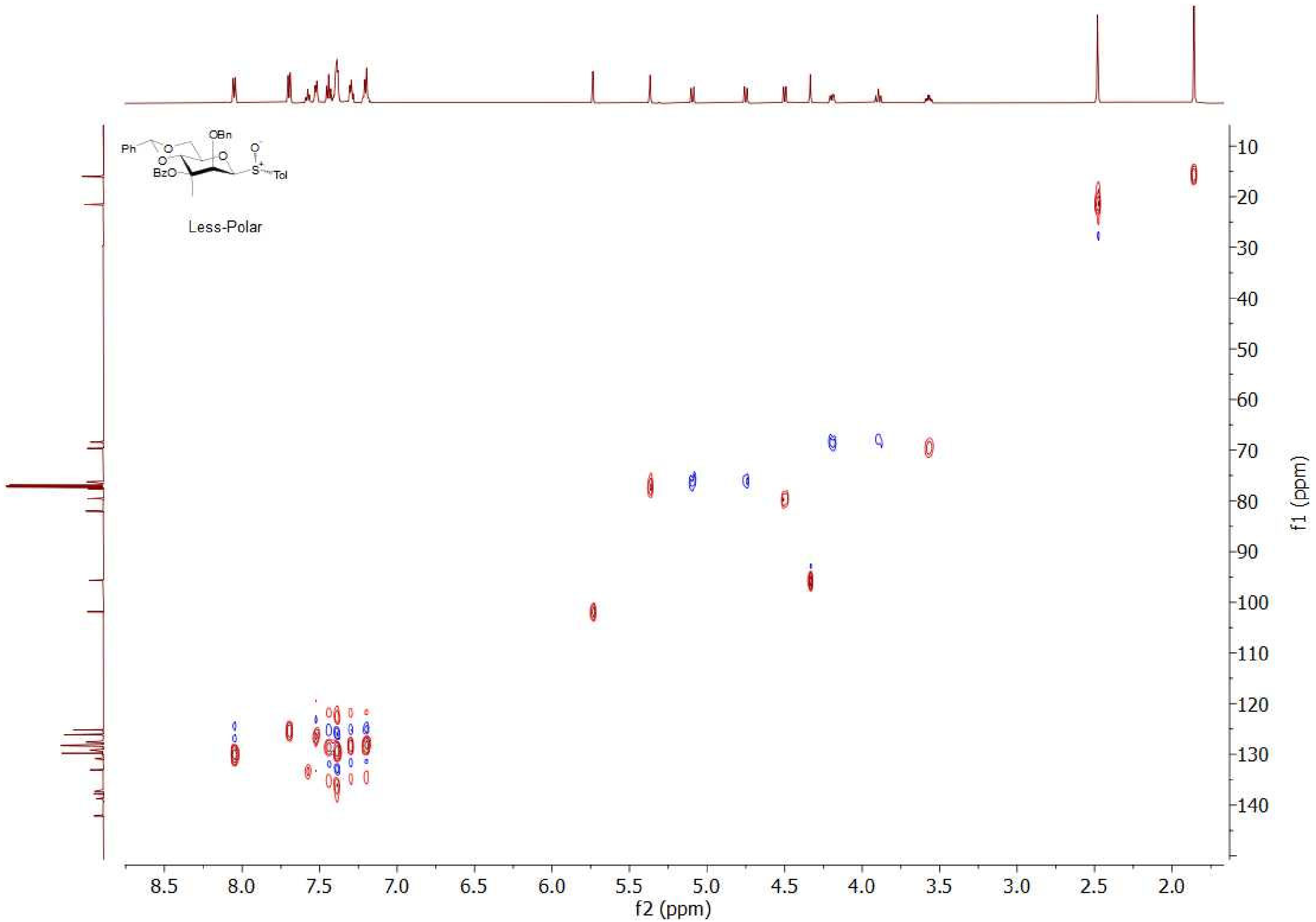

Figure S62. HMBC NMR (600 MHz, $CDCl_3$) spectrum of *p*-methylphenyl 3-*O*-benzoyl-2-*O*-benzyl-4,6-*O*-benzylidene-3-*C*-methyl-thio-β-D-mannopyranoside *S*-oxide **40** (Less-polar diastereoisomer):

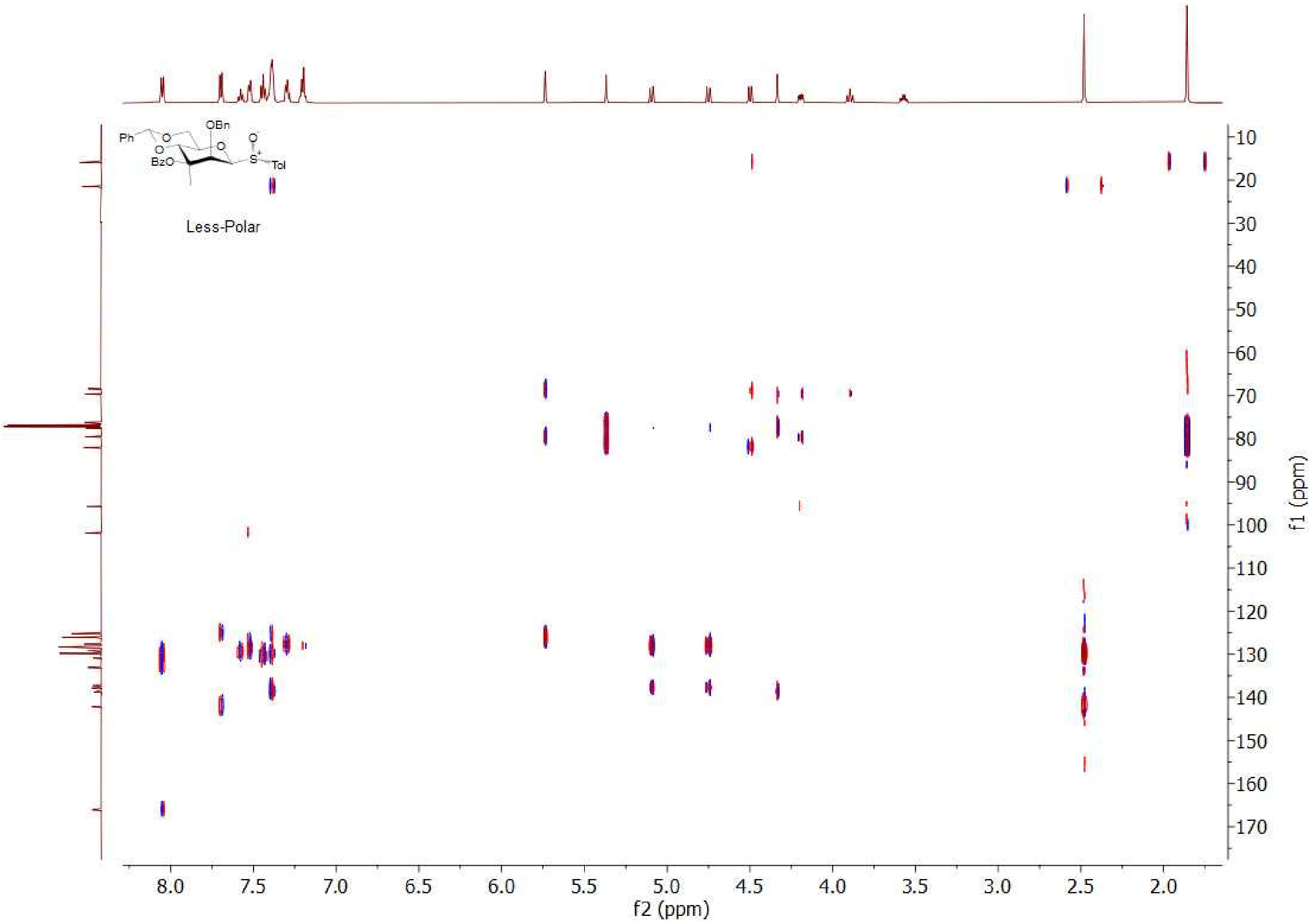

Figure S63. ^{1}H NMR (600 MHz, C_6D_6) spectrum of *p*-methylphenyl 3-*O*-benzoyl-2-*O*-benzyl-4,6-*O*-benzylidene-3-*C*-methyl-thio-β-D-mannopyranoside *S*-oxide **40** (Polar diastereoisomer):

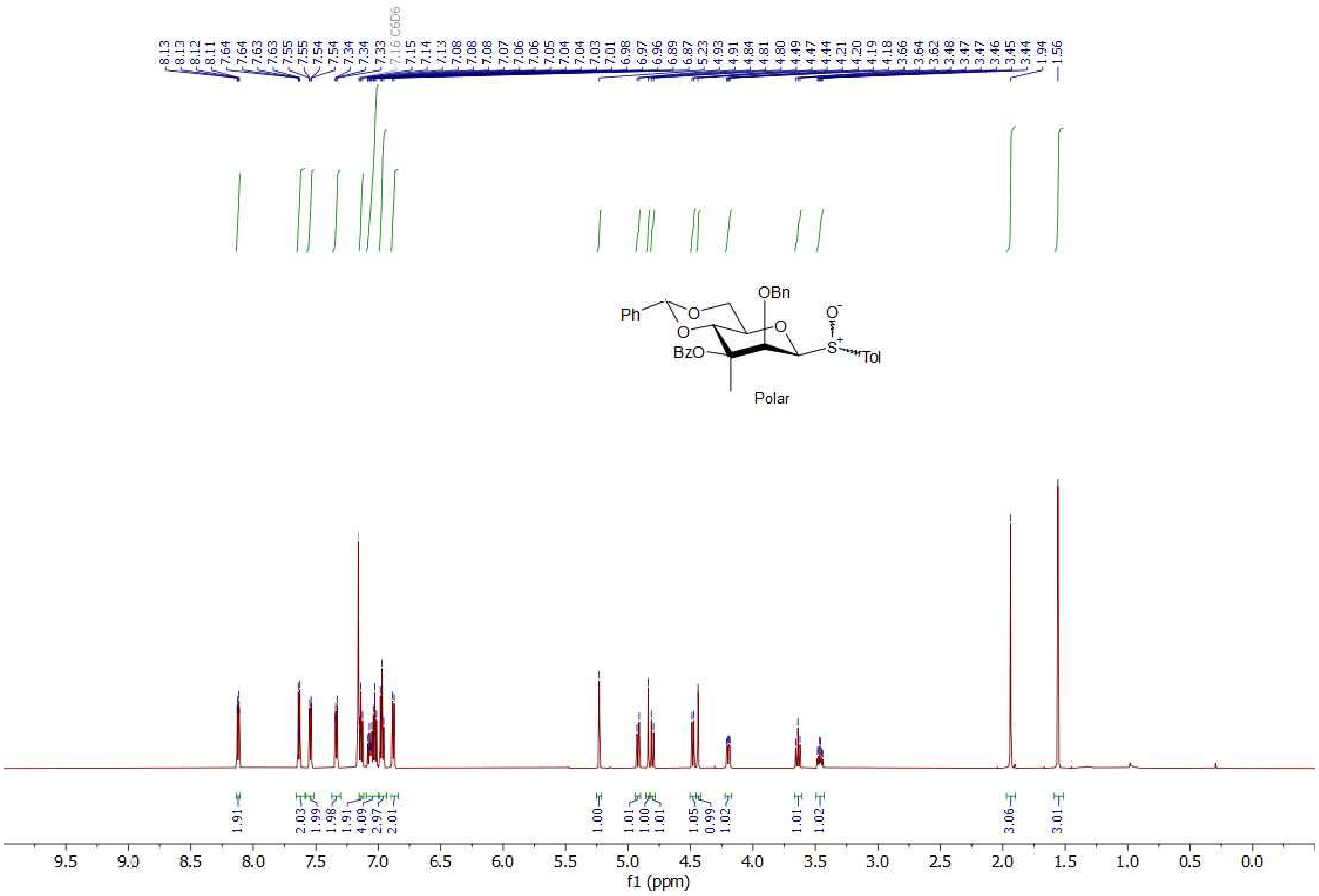

Figure S64. COSY NMR (600 MHz, C_6D_6) spectrum of *p*-methylphenyl 3-*O*-benzoyl-2-*O*-benzyl-4,6-*O*-benzylidene-3-*C*-methyl-thio-β-D-mannopyranoside *S*-oxide **40** (Polar diastereoisomer):

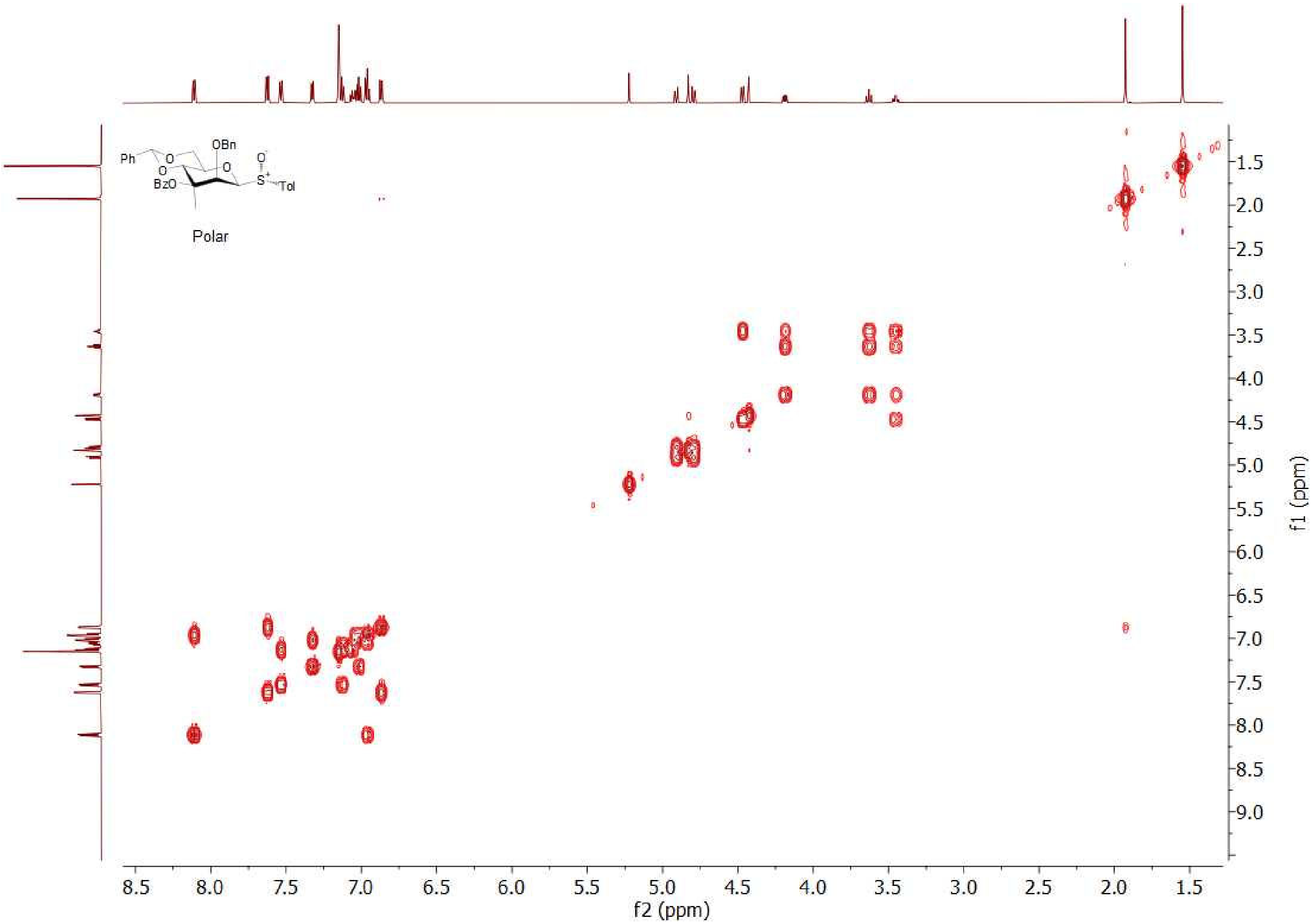

Figure S65. $^{13}C\{^1H\}$ NMR (151 MHz, C_6D_6) spectrum of *p*-methylphenyl 3-*O*-benzoyl-2-*O*-benzyl-4,6-*O*-benzylidene-3-*C*-methyl-thio-β-D-mannopyranoside *S*-oxide **40** (Polar diastereoisomer):

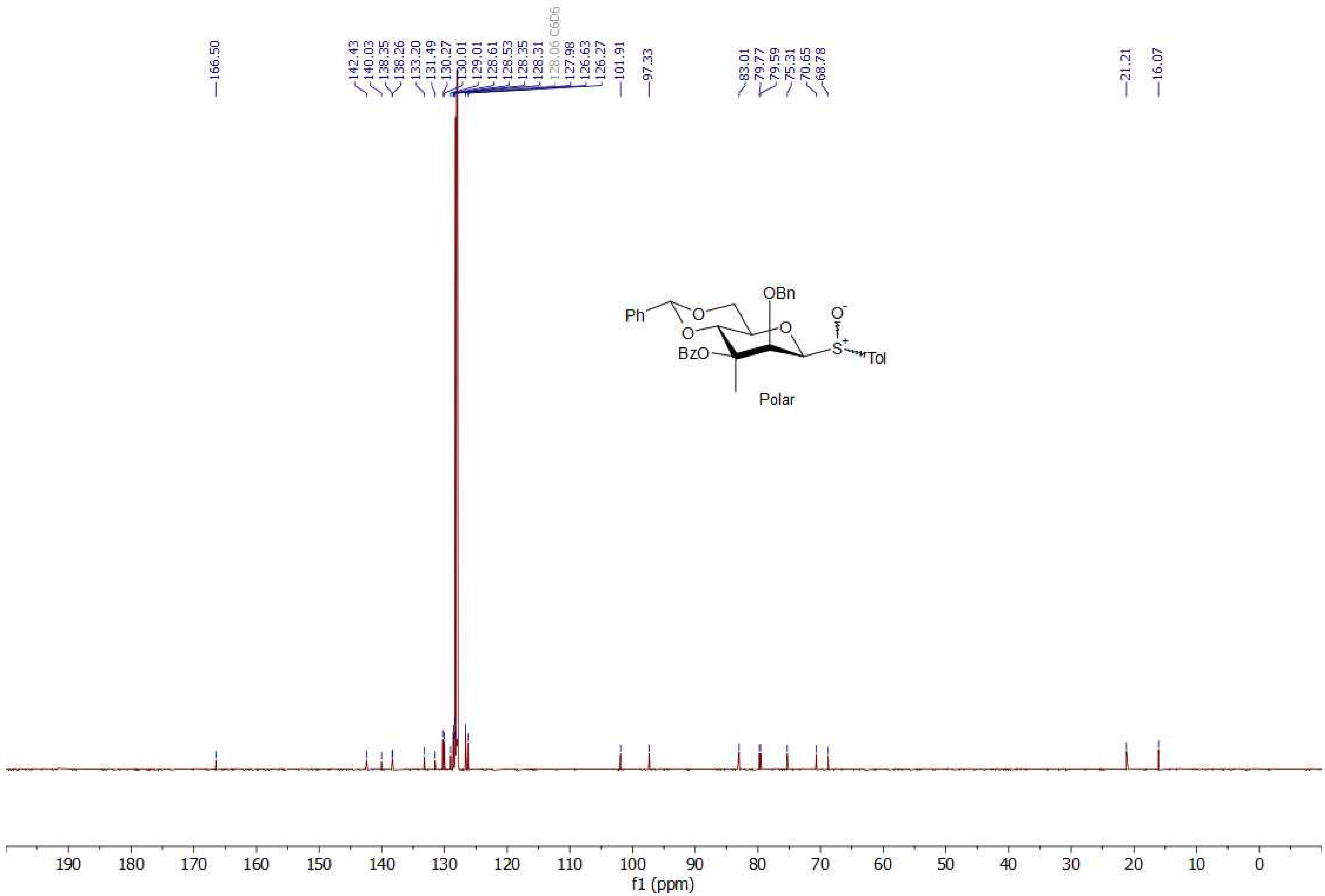

Figure S66. $^{13}C\{^{1}H\}$ DEPT NMR (151 MHz, C_6D_6) spectrum of *p*-methylphenyl 3-*O*-benzoyl-2-*O*-benzyl-4,6-*O*-benzylidene-3-*C*-methyl-thio-β-D-mannopyranoside *S*-oxide **40** (Polar diastereoisomer):

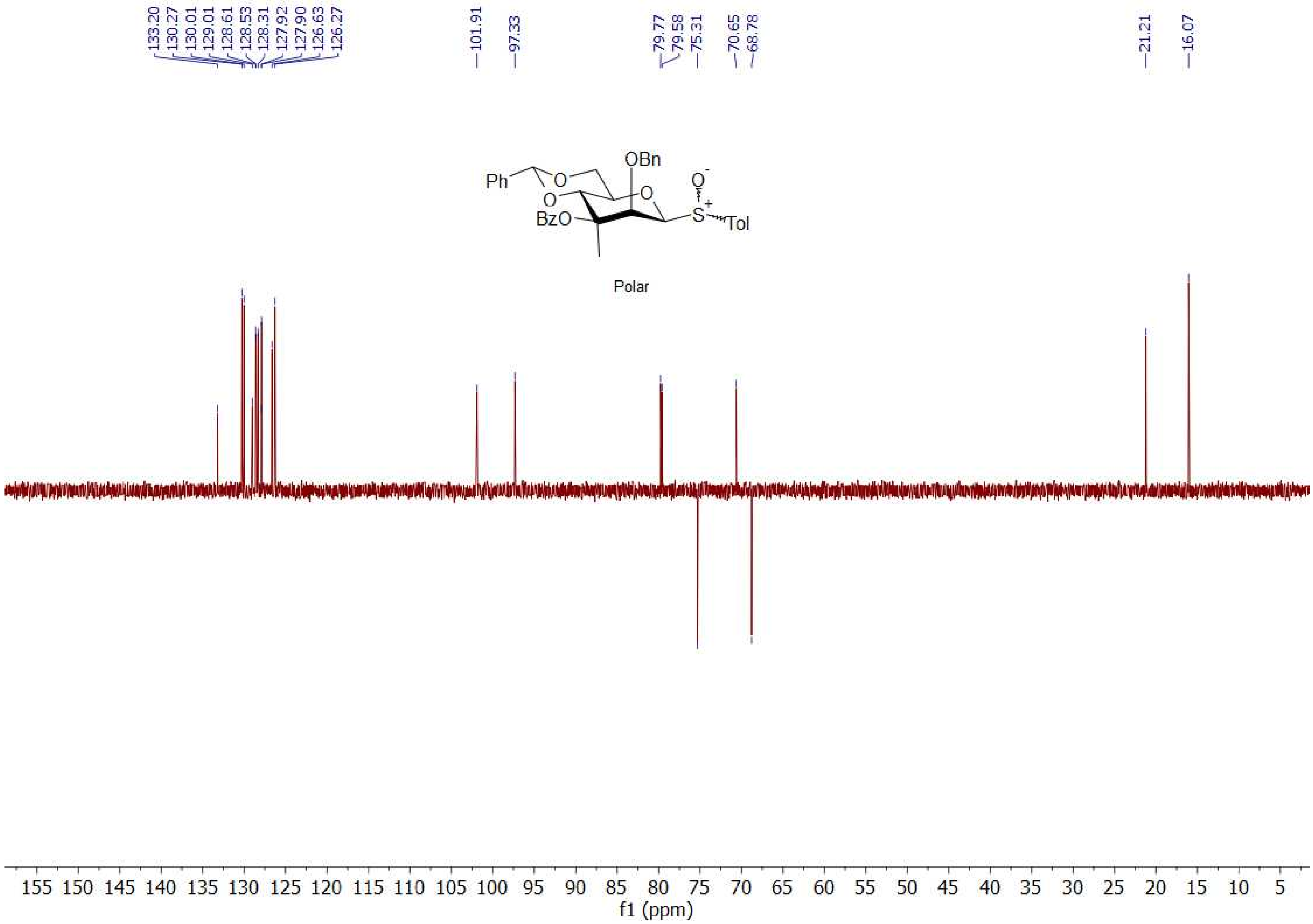

Figure S67. HSQC NMR (600 MHz, C_6D_6) spectrum of *p*-methylphenyl 3-*O*-benzoyl-2-*O*-benzyl-4,6-*O*-benzylidene-3-*C*-methyl-thio-β-D-mannopyranoside *S*-oxide **40** (Polar diastereoisomer):

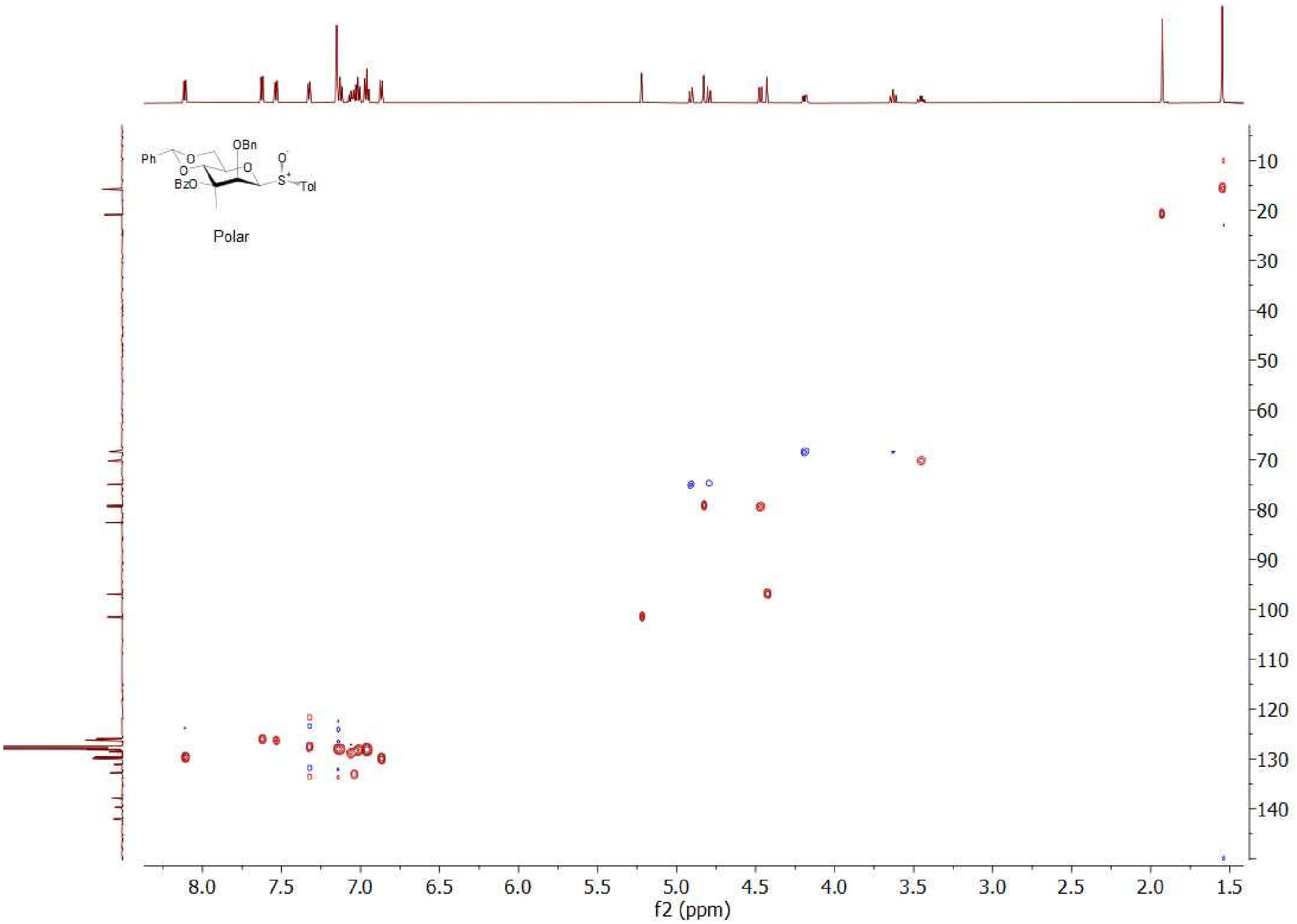

Figure S68. HMBC NMR (600 MHz, C_6D_6) spectrum of *p*-methylphenyl 3-*O*-benzoyl-2-*O*-benzyl-4,6-*O*-benzylidene-3-*C*-methyl-thio-β-D-mannopyranoside *S*-oxide **40** (Polar diastereoisomer):

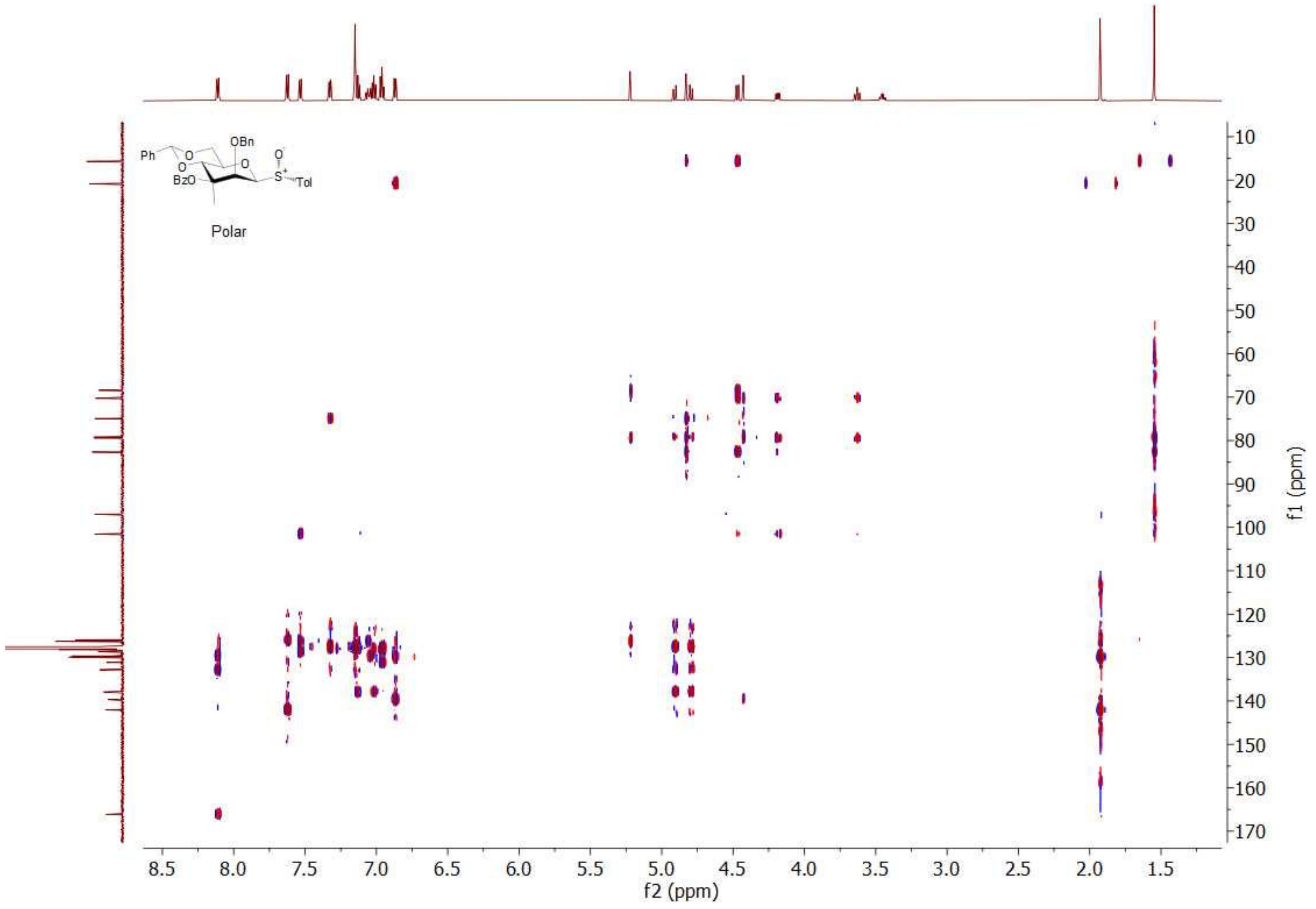

Figure S69. ^{1}H NMR (600 MHz, $CDCl_3$) spectrum of *p*-methylphenyl 3-*O*-(benzoyl-α-^{13}C)-2-*O*-benzyl-4,6-*O*-benzylidene-3-*C*-methyl-thio-β-D-mannopyranoside *S*-oxide **^{13}C-40** (Less-polar diastereoisomer):

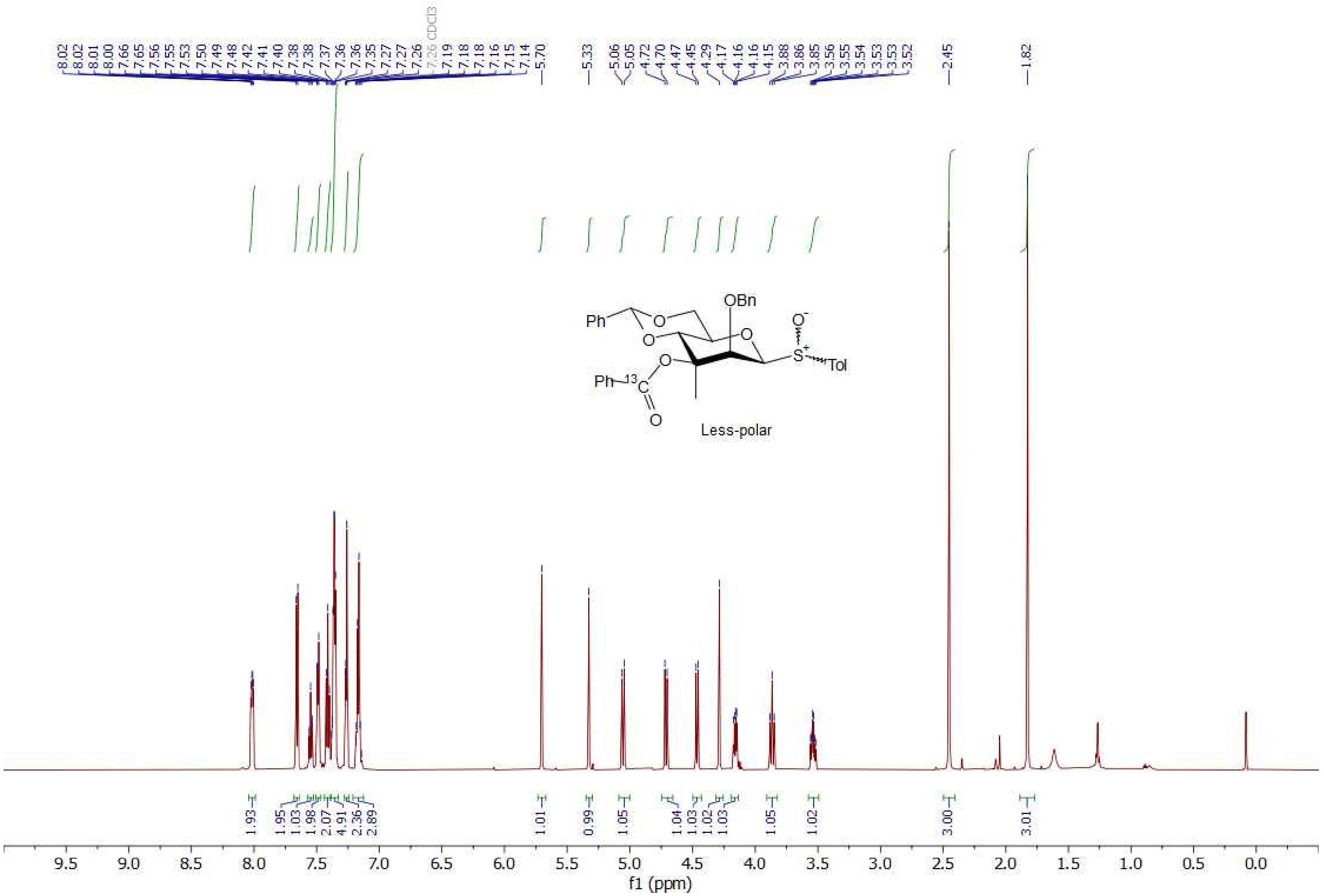

Figure S70. COSY NMR (600 MHz, $CDCl_3$) spectrum of *p*-methylphenyl 3-*O*-(benzoyl-α-^{13}C)-2-*O*-benzyl-4,6-*O*-benzylidene-3-*C*-methyl-thio-β-D-mannopyranoside *S*-oxide **^{13}C-40** (Less-polar diastereoisomer):

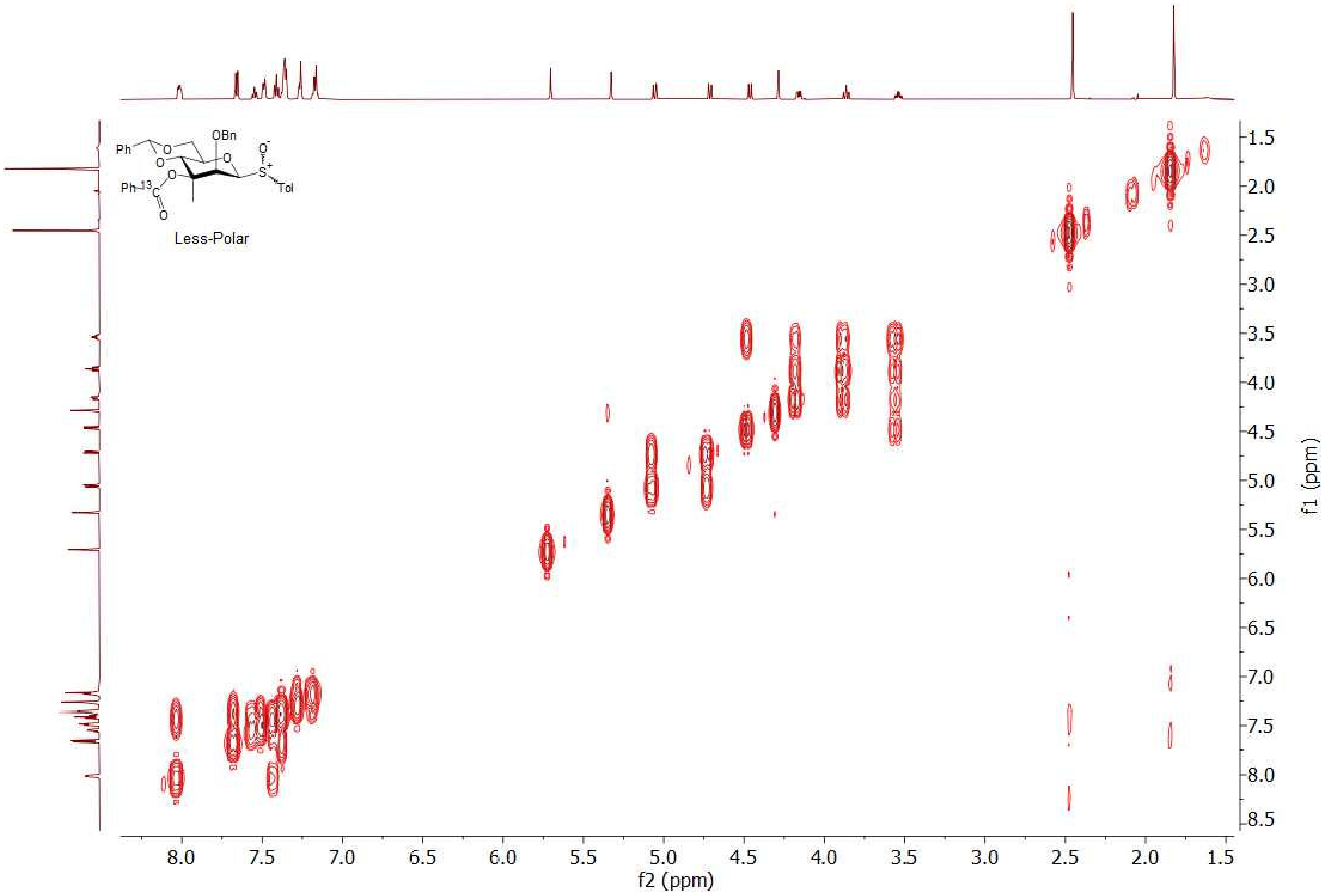

Figure S71. $^{13}C\{^{1}H\}$ NMR (151 MHz, $CDCl_3$) spectrum of *p*-methylphenyl 3-*O*-(benzoyl-α-^{13}C)-2-*O*-benzyl-4,6-*O*-benzylidene-3-*C*-methyl-thio-β-D-mannopyranoside *S*-oxide **^{13}C-40** (Less-polar diastereoisomer):

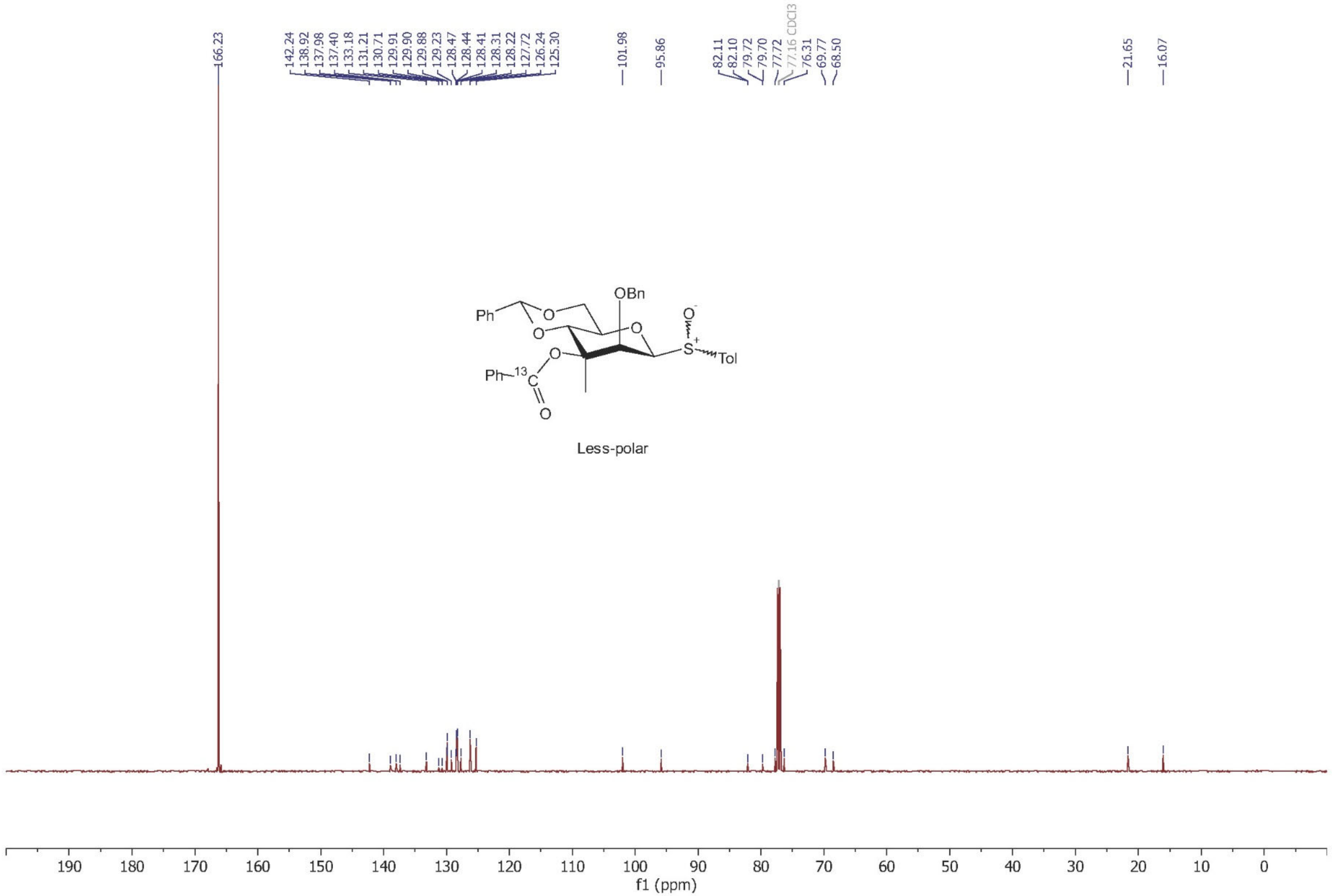

Figure S72. $^{13}C\{^{1}H\}$ DEPT NMR (151 MHz, $CDCl_3$) spectrum of *p*-methylphenyl 3-*O*-(benzoyl-α-^{13}C)-2-*O*-benzyl-4,6-*O*-benzylidene-3-*C*-methyl-thio-β-D-mannopyranoside *S*-oxide **^{13}C-40** (Less-polar diastereoisomer):

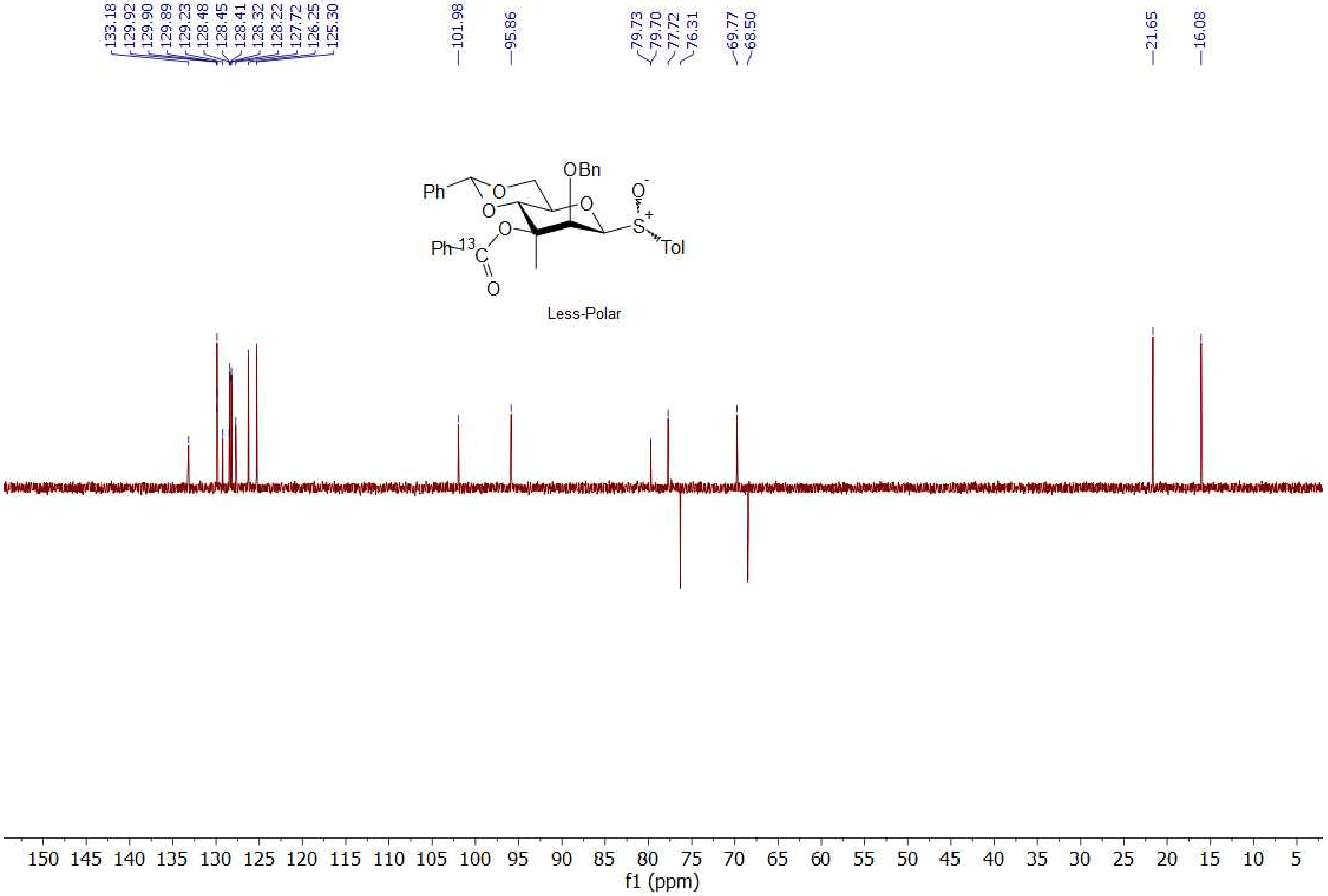

Figure S73. HSQC NMR (600 MHz, $CDCl_3$) spectrum of *p*-methylphenyl 3-*O*-(benzoyl-α-^{13}C)-2-*O*-benzyl-4,6-*O*-benzylidene-3-*C*-methyl-thio-β-D-mannopyranoside *S*-oxide **^{13}C-40** (Less-polar diastereoisomer):

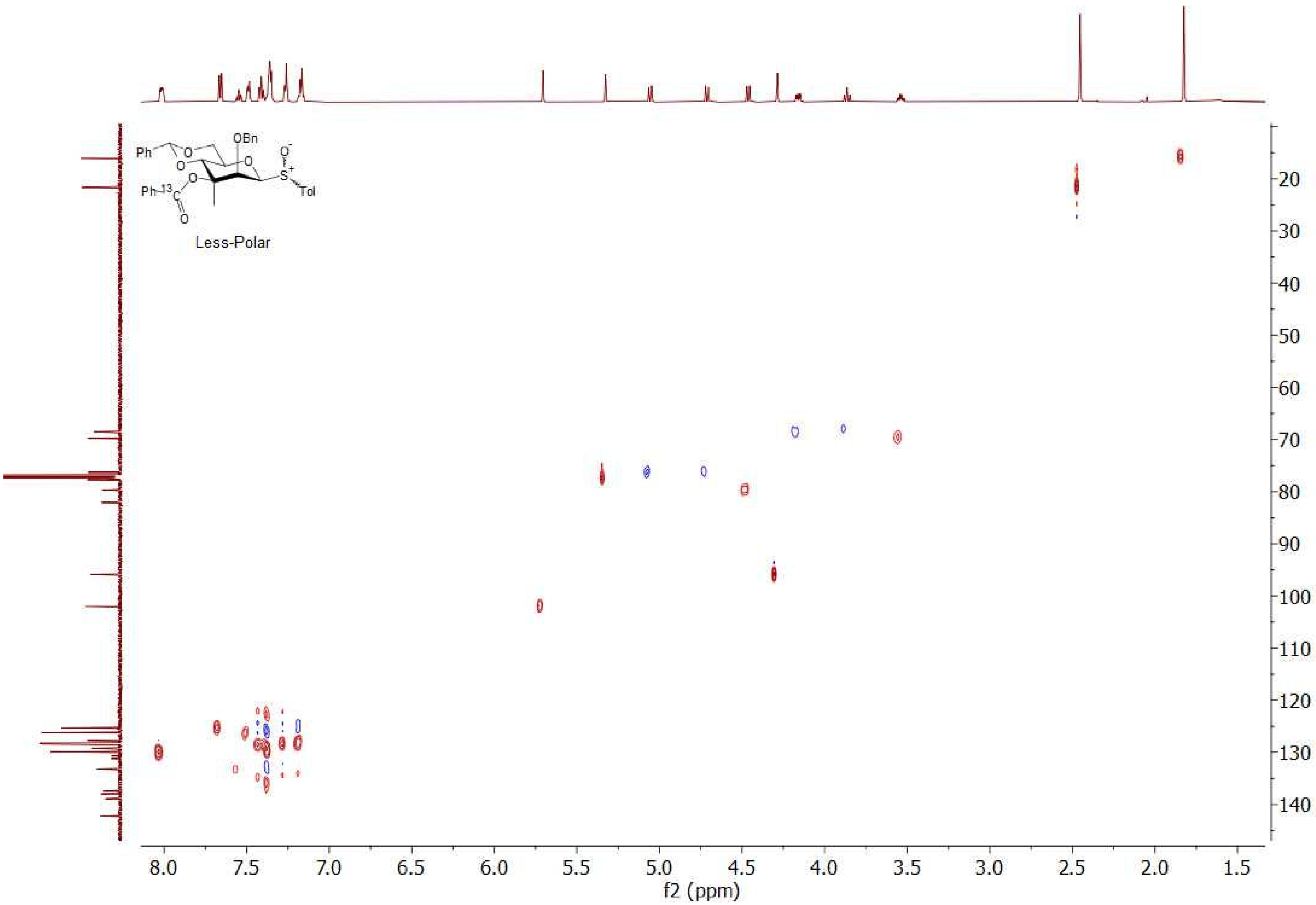

Figure S74. HMBC NMR (600 MHz, $CDCl_3$) spectrum of *p*-methylphenyl 3-*O*-(benzoyl-α-^{13}C)-2-*O*-benzyl-4,6-*O*-benzylidene-3-*C*-methyl-thio-β-D-mannopyranoside *S*-oxide **^{13}C-40** (Less-polar diastereoisomer):

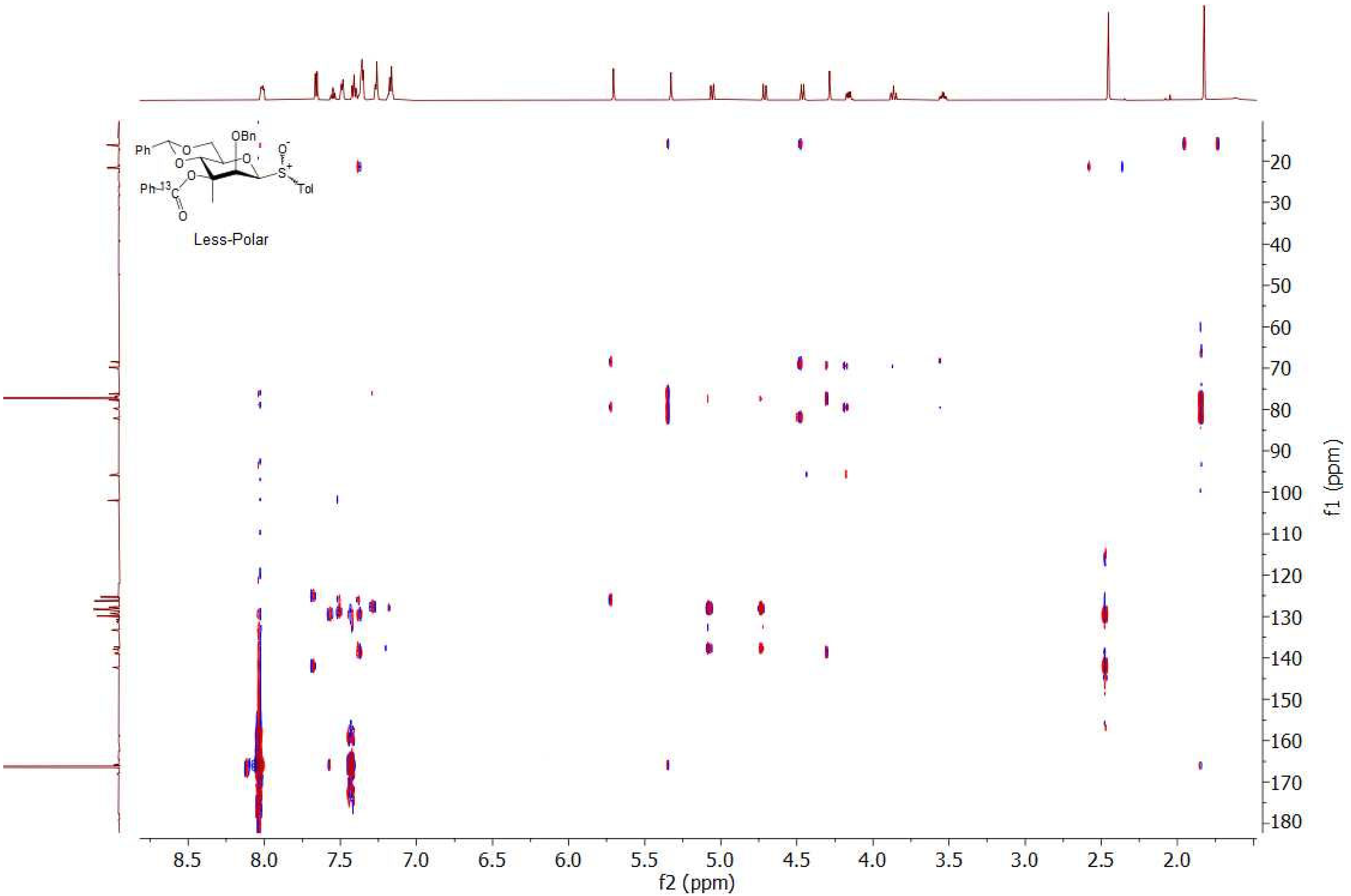

Figure S75. ^{1}H NMR (600 MHz, C_6D_6) spectrum of *p*-methylphenyl 3-*O*-(benzoyl-α-^{13}C)-2-*O*-benzyl-4,6-*O*-benzylidene-3-*C*-methyl-thio-β-D-mannopyranoside *S*-oxide **^{13}C-40** (Polar diastereoisomer):

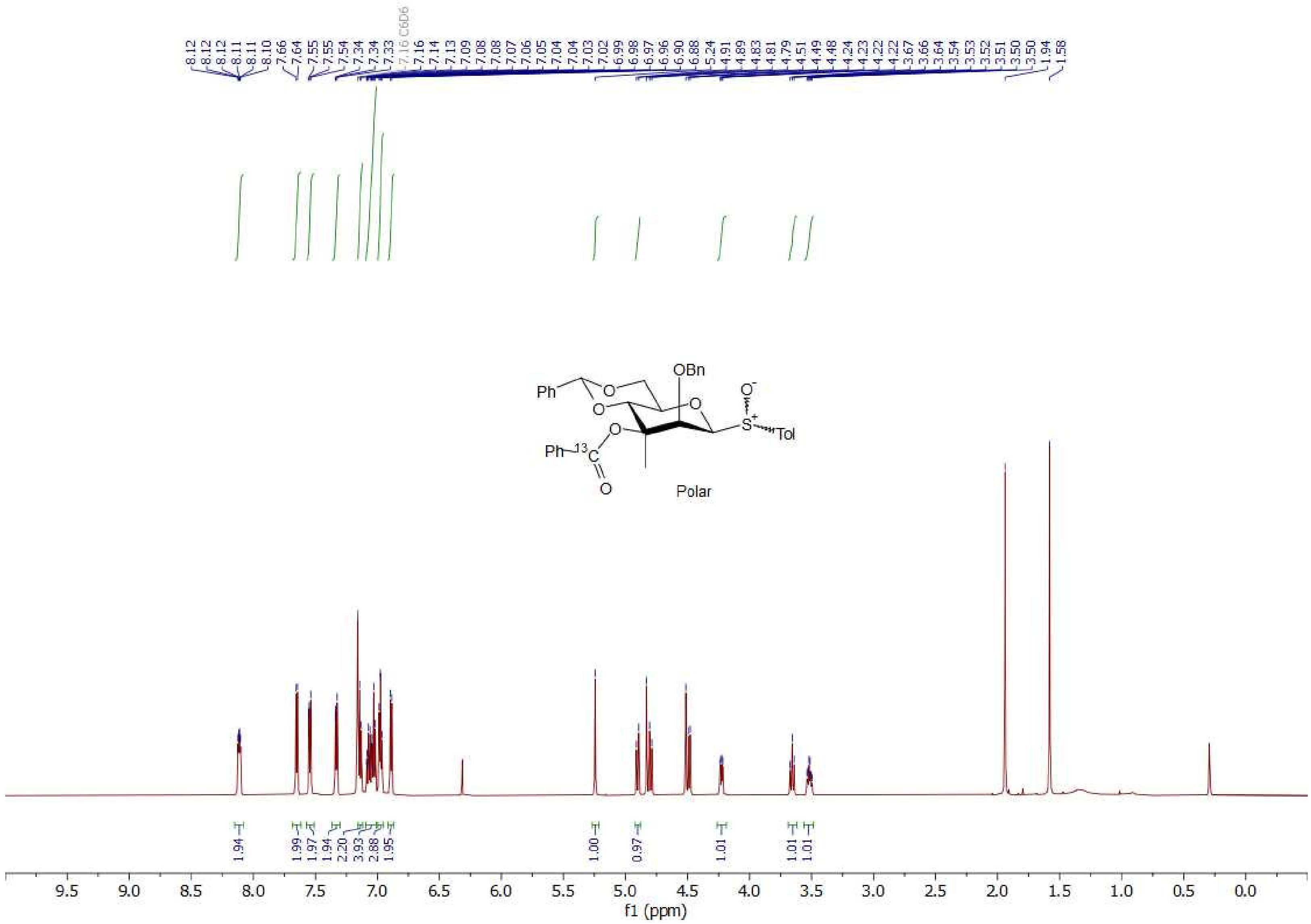

Figure S76. COSY NMR (600 MHz, C_6D_6) spectrum of *p*-methylphenyl 3-*O*-(benzoyl-α-^{13}C)-2-*O*-benzyl-4,6-*O*-benzylidene-3-*C*-methyl-thio-β-D-mannopyranoside *S*-oxide **^{13}C-40** (Polar diastereoisomer):

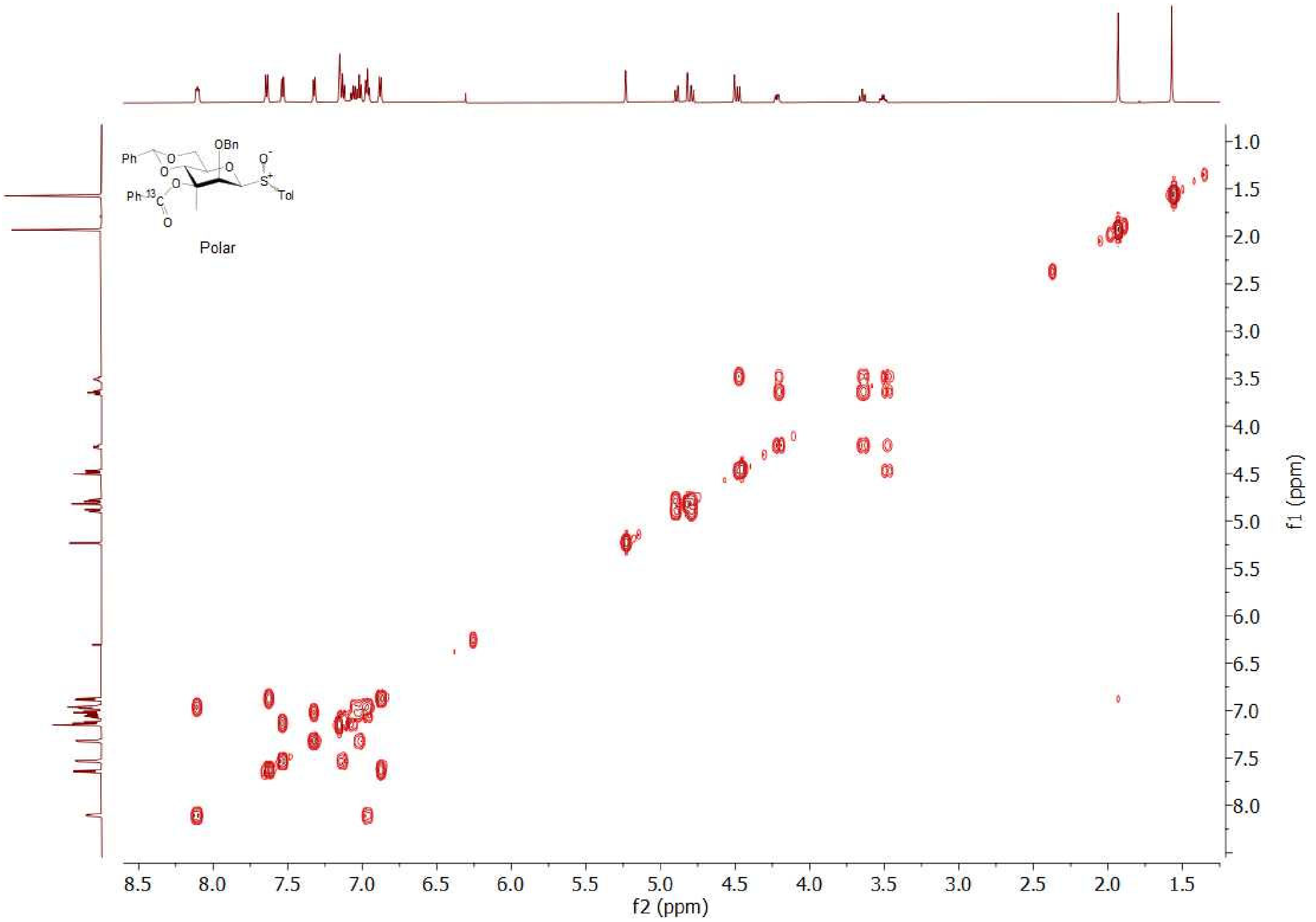

Figure S77. $^{13}C\{^{1}H\}$ NMR (151 MHz, C_6D_6) spectrum of *p*-methylphenyl 3-*O*-(benzoyl-α-^{13}C)-2-*O*-benzyl-4,6-*O*-benzylidene-3-*C*-methyl-thio-β-D-mannopyranoside *S*-oxide **^{13}C-40** (Polar diastereoisomer):

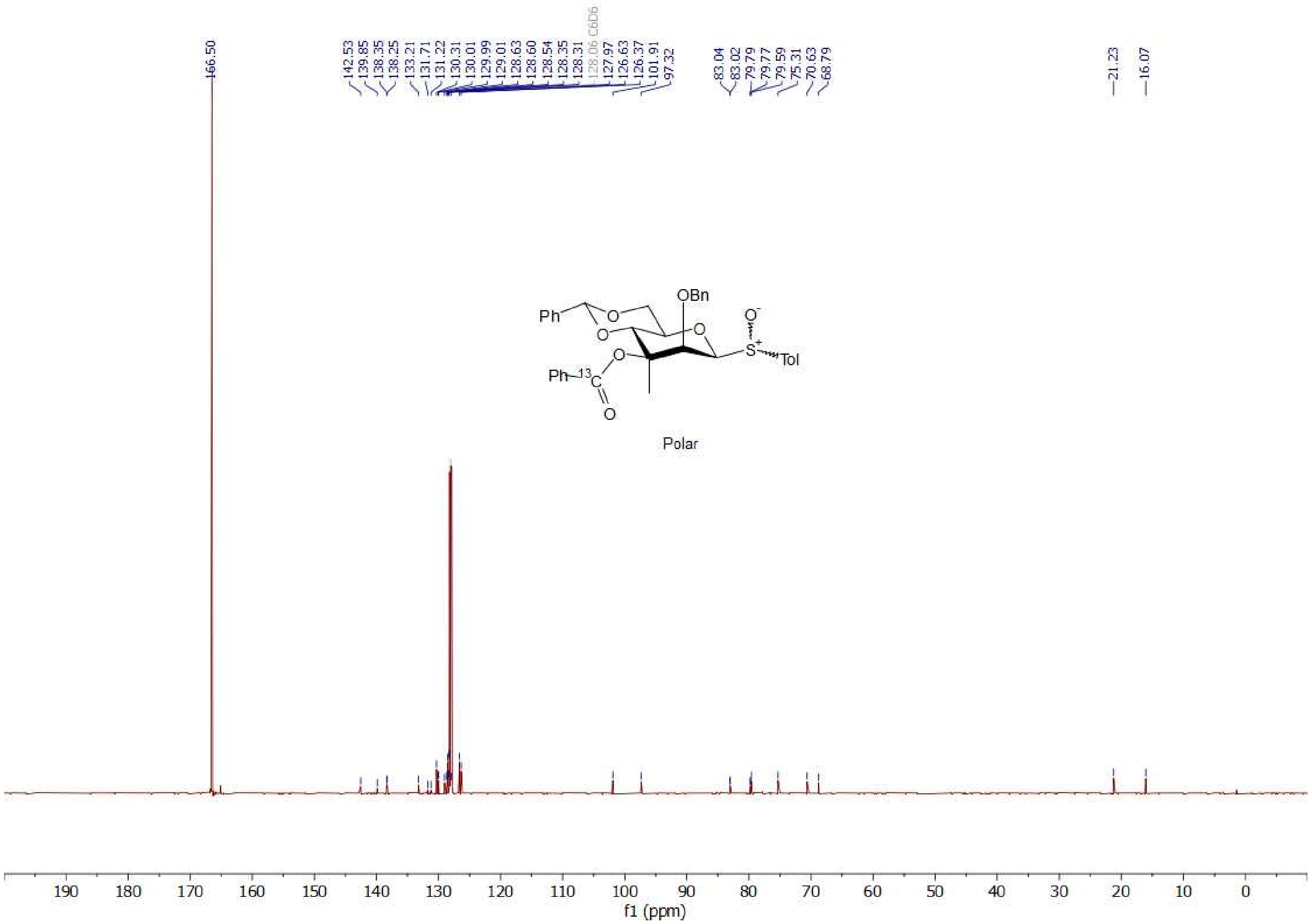

Figure S78. $^{13}C\{^{1}H\}$ DEPT NMR (151 MHz, C_6D_6) spectrum of *p*-methylphenyl 3-*O*-(benzoyl-α-^{13}C)-2-*O*-benzyl-4,6-*O*-benzylidene-3-*C*-methyl-thio-β-D-mannopyranoside *S*-oxide **^{13}C-40** (Polar diastereoisomer):

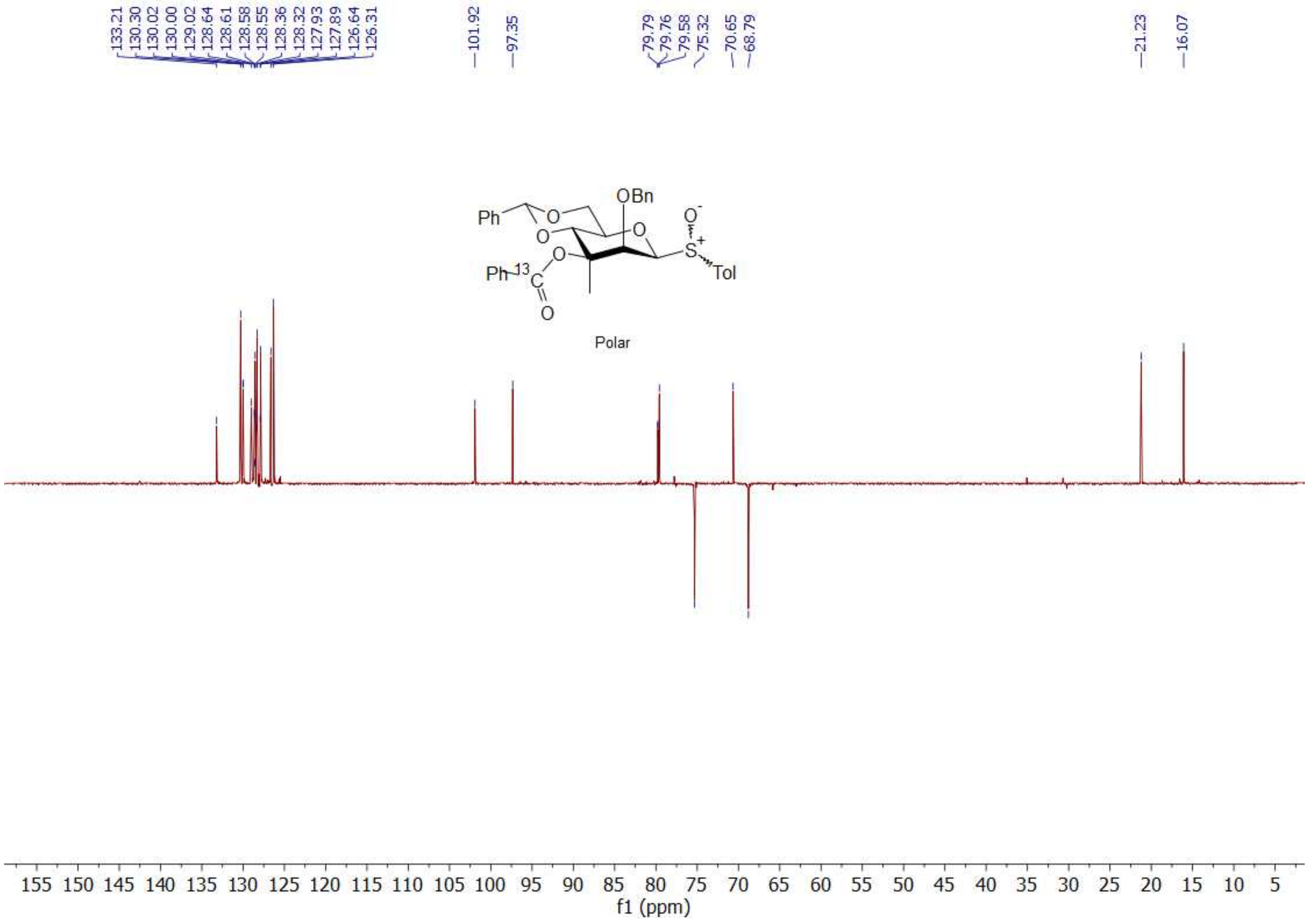

Figure S79. HSQC NMR (600 MHz, C_6D_6) spectrum of *p*-methylphenyl 3-*O*-(benzoyl-α-^{13}C)-2-*O*-benzyl-4,6-*O*-benzylidene-3-*C*-methyl-thio-β-D-mannopyranoside *S*-oxide **^{13}C-40** (Polar diastereoisomer):

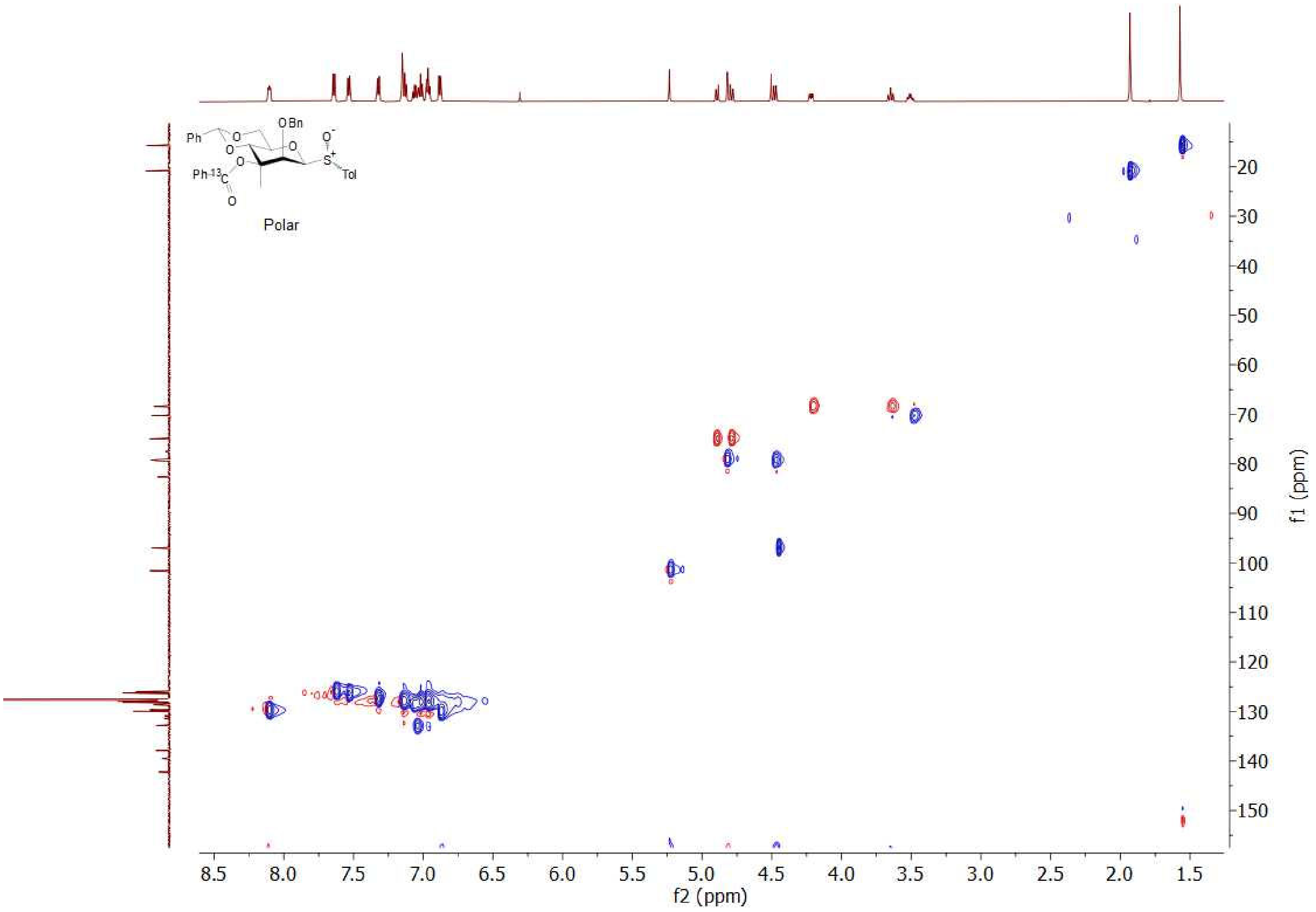

Figure S80. HMBC NMR (600 MHz, C_6D_6) spectrum of *p*-methylphenyl 3-*O*-(benzoyl-α-^{13}C)-2-*O*-benzyl-4,6-*O*-benzylidene-3-*C*-methyl-thio-β-D-mannopyranoside *S*-oxide **^{13}C-40** (Polar diastereoisomer):

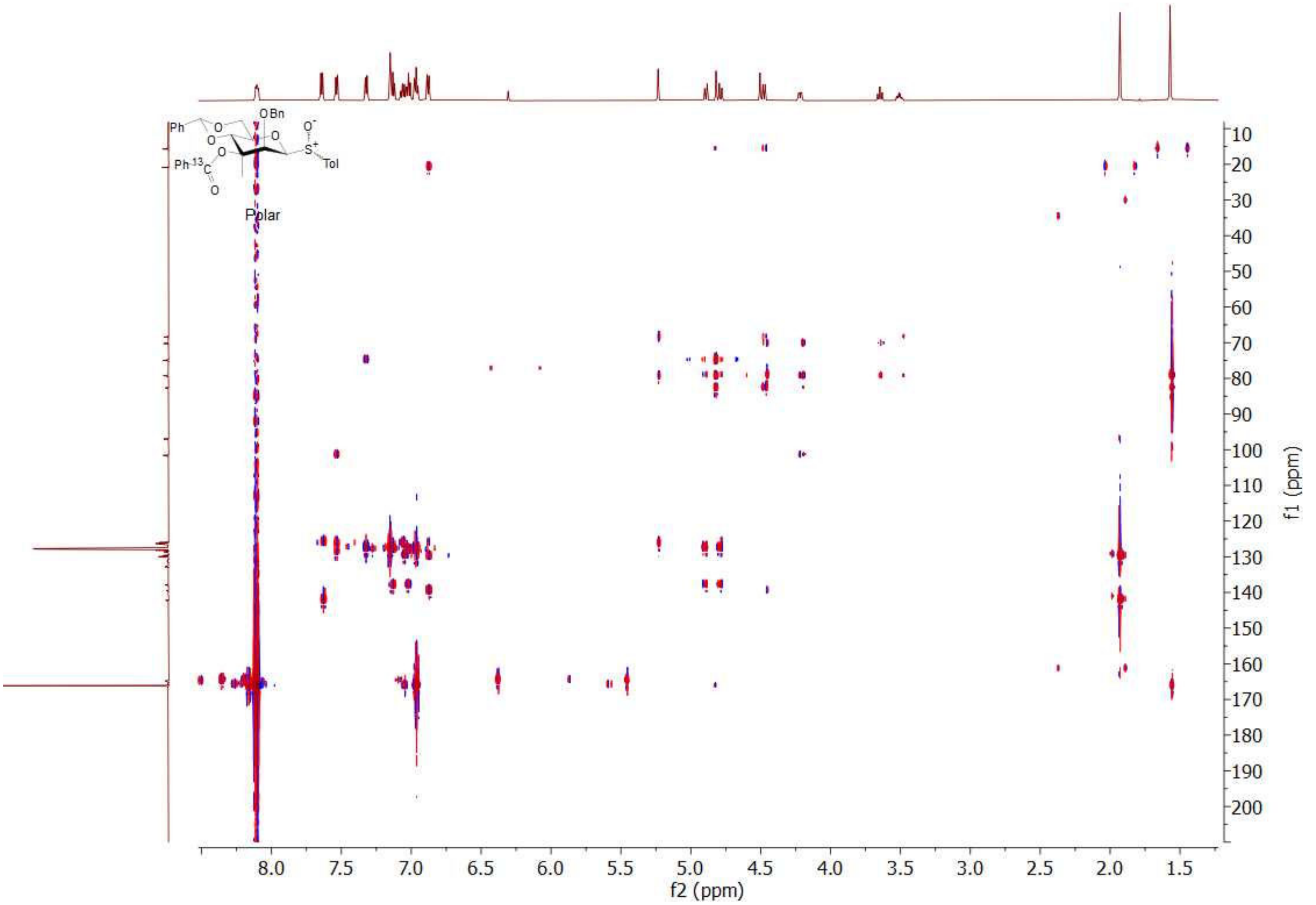

Figure S81. ^{1}H NMR (600 MHz, $CDCl_3$) spectrum of *p*-methylphenyl 2-*O*-benzyl-4,6-*O*-benzylidene-3-*O*-*p*-nitrobenzoyl-3-*C*-methyl-thio-β-D-mannopyranoside *S*-oxide **41** (Less-polar diastereoisomer):

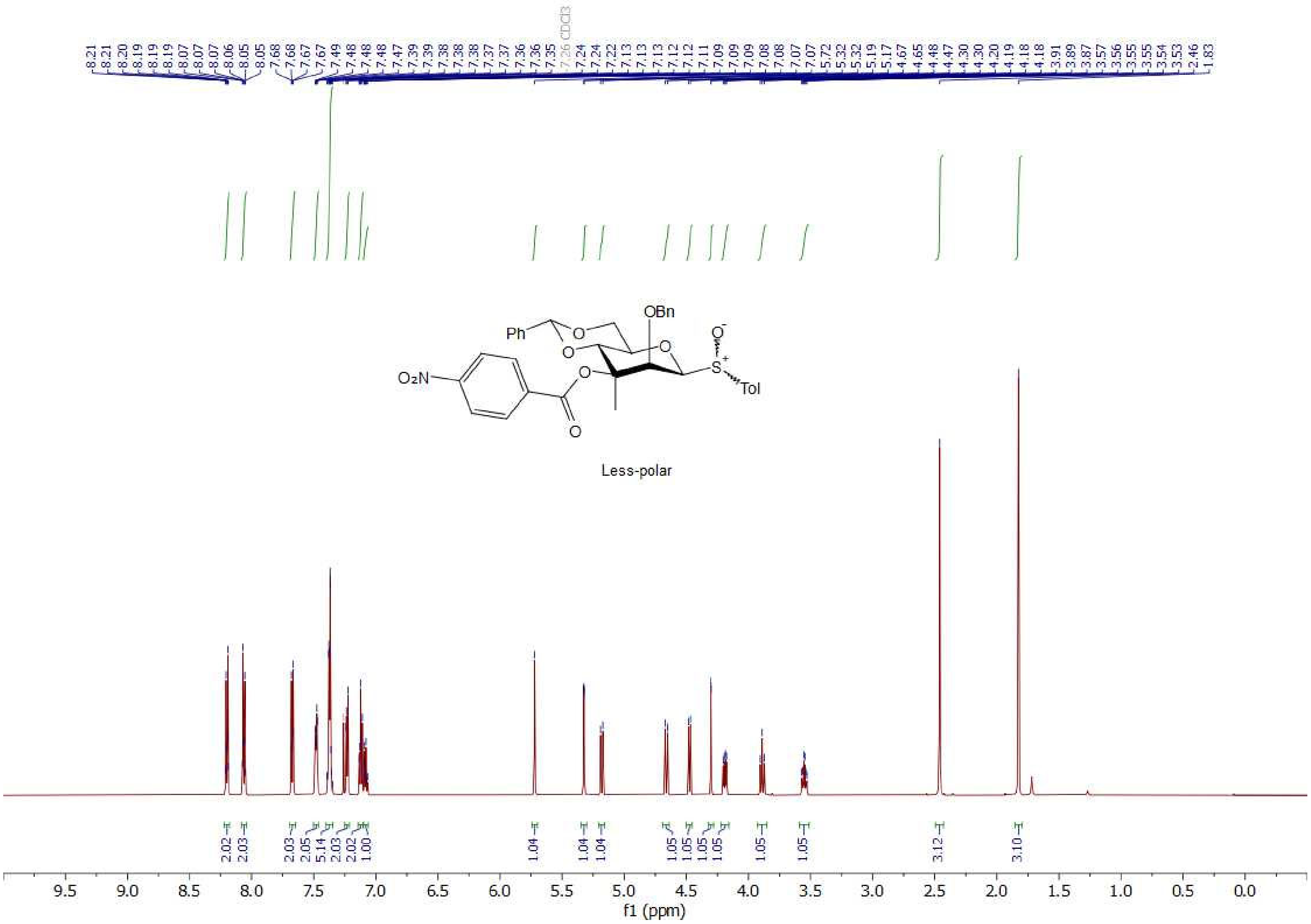

Figure S82. COSY NMR (600 MHz, $CDCl_3$) spectrum of *p*-methylphenyl 2-*O*-benzyl-4,6-*O*-benzylidene-3-*O*-*p*-nitrobenzoyl-3-*C*-methyl-thio-β-D-mannopyranoside *S*-oxide **41** (Less-polar diastereoisomer):

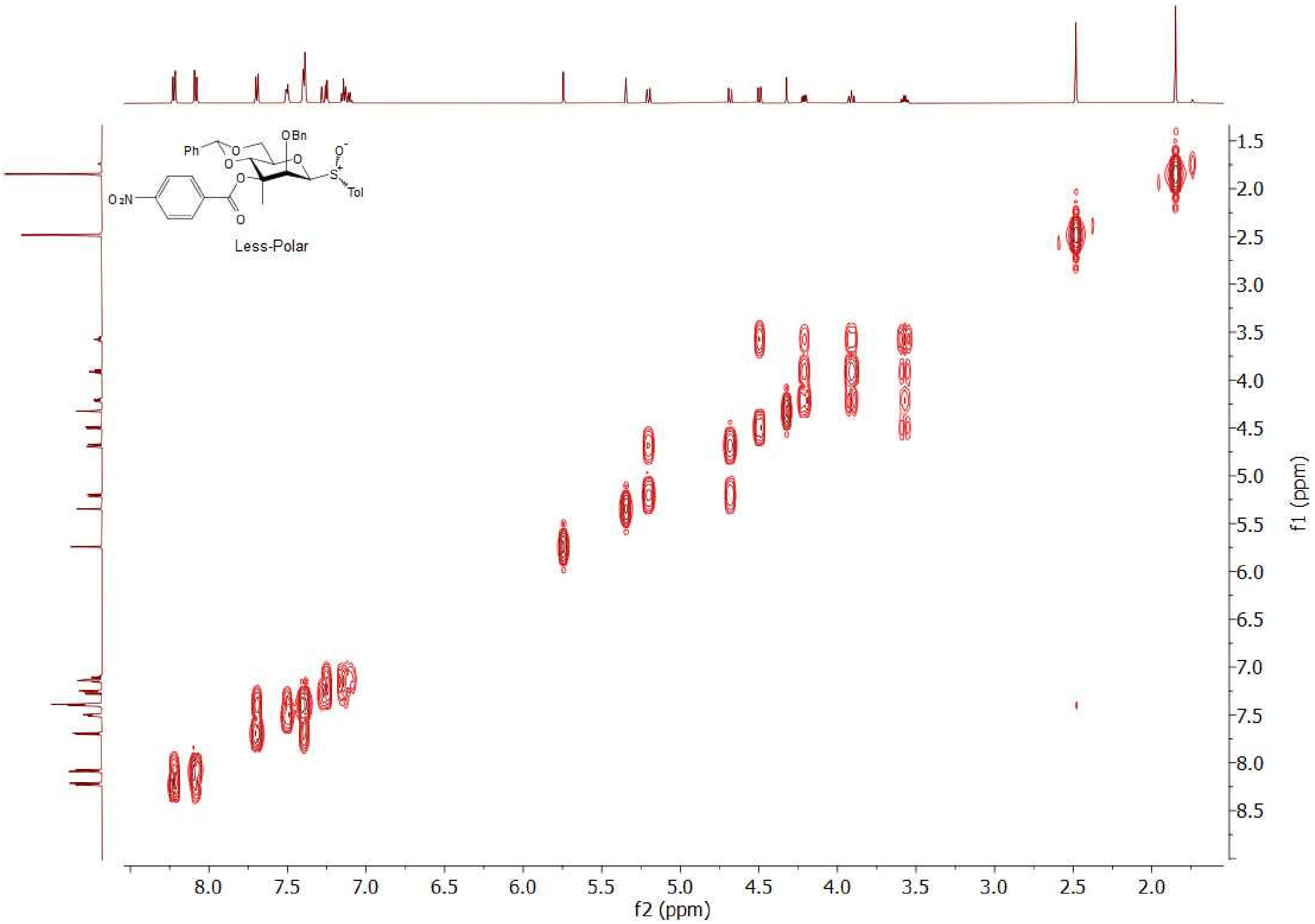

Figure S83. $^{13}C\{^1H\}$ NMR (151 MHz, $CDCl_3$) spectrum of *p*-methylphenyl 2-*O*-benzyl-4,6-*O*-benzylidene-3-*O*-*p*-nitrobenzoyl-3-*C*-methyl-thio-β-D-mannopyranoside *S*-oxide **41** (Less-polar diastereoisomer):

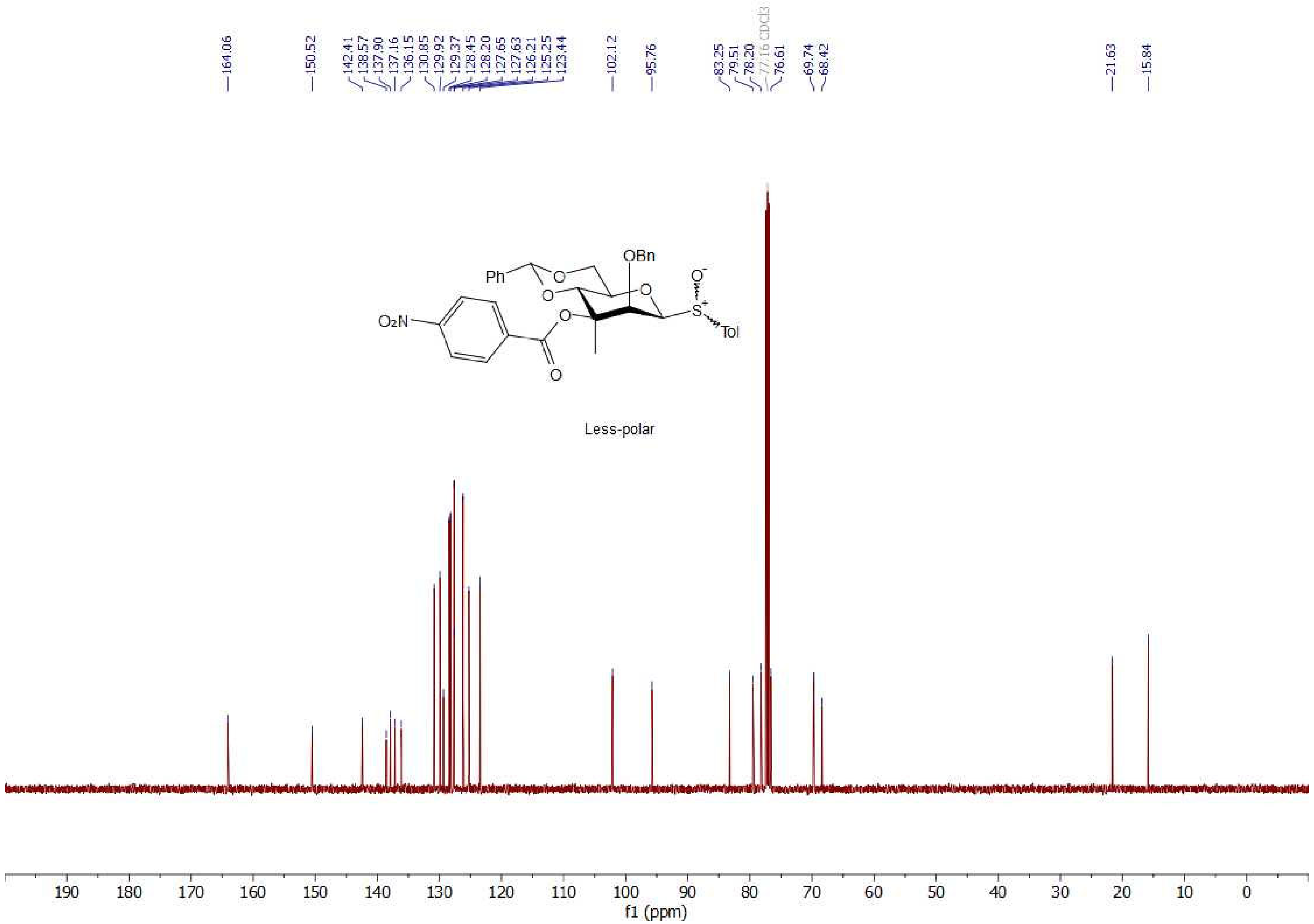

Figure S84. $^{13}C\{^{1}H\}$ NMR (151 MHz, $CDCl_3$) spectrum of *p*-methylphenyl 2-*O*-benzyl-4,6-*O*-benzylidene-3-*O*-*p*-nitrobenzoyl-3-*C*-methyl-thio-β-D-mannopyranoside *S*-oxide **41** (Less-polar diastereoisomer):

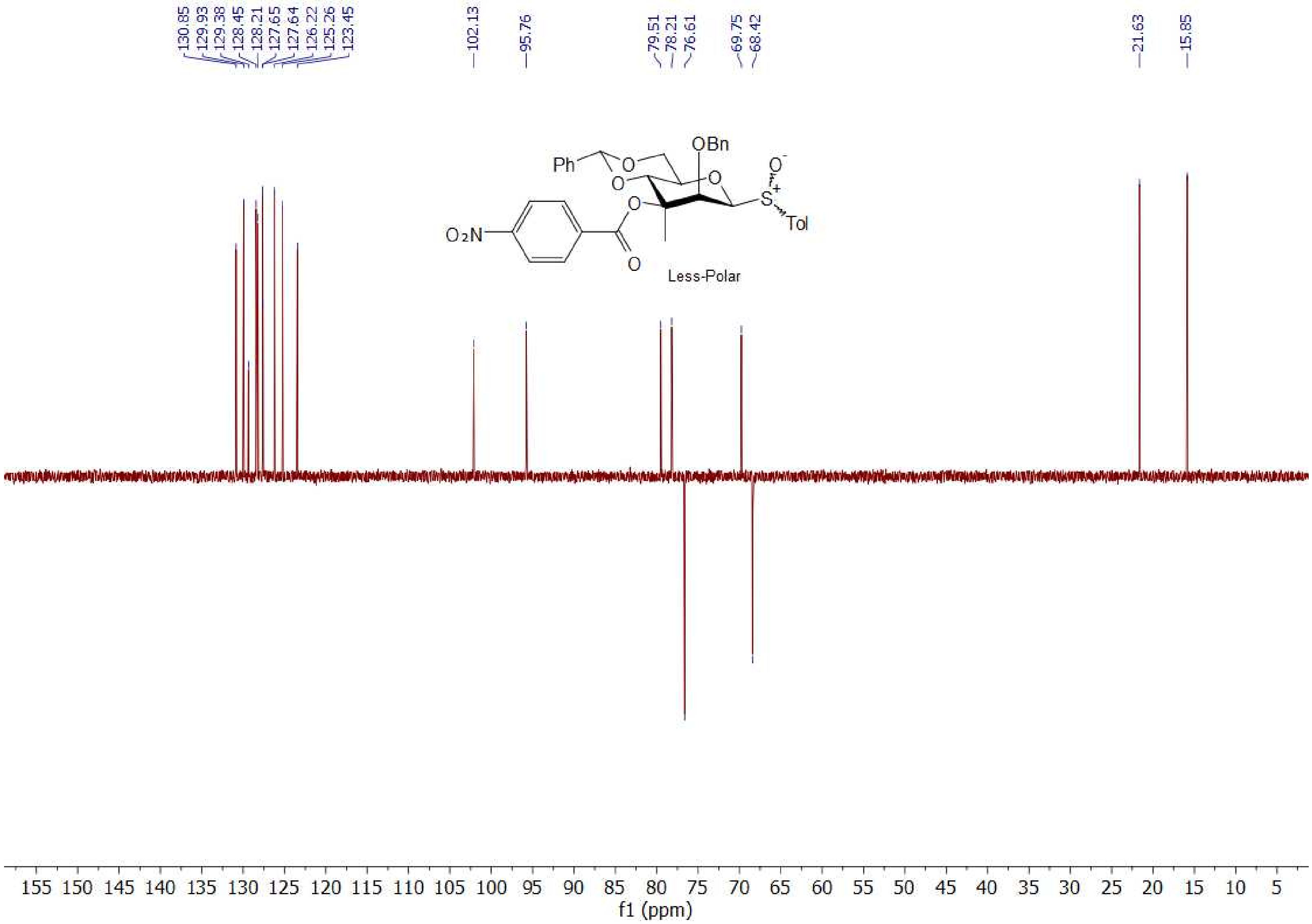

Figure S85. HSQC NMR (600 MHz, $CDCl_3$) spectrum of *p*-methylphenyl 2-*O*-benzyl-4,6-*O*-benzylidene-3-*O*-*p*-nitrobenzoyl-3-*C*-methyl-thio-β-D-mannopyranoside *S*-oxide **41** (Less-polar diastereoisomer):

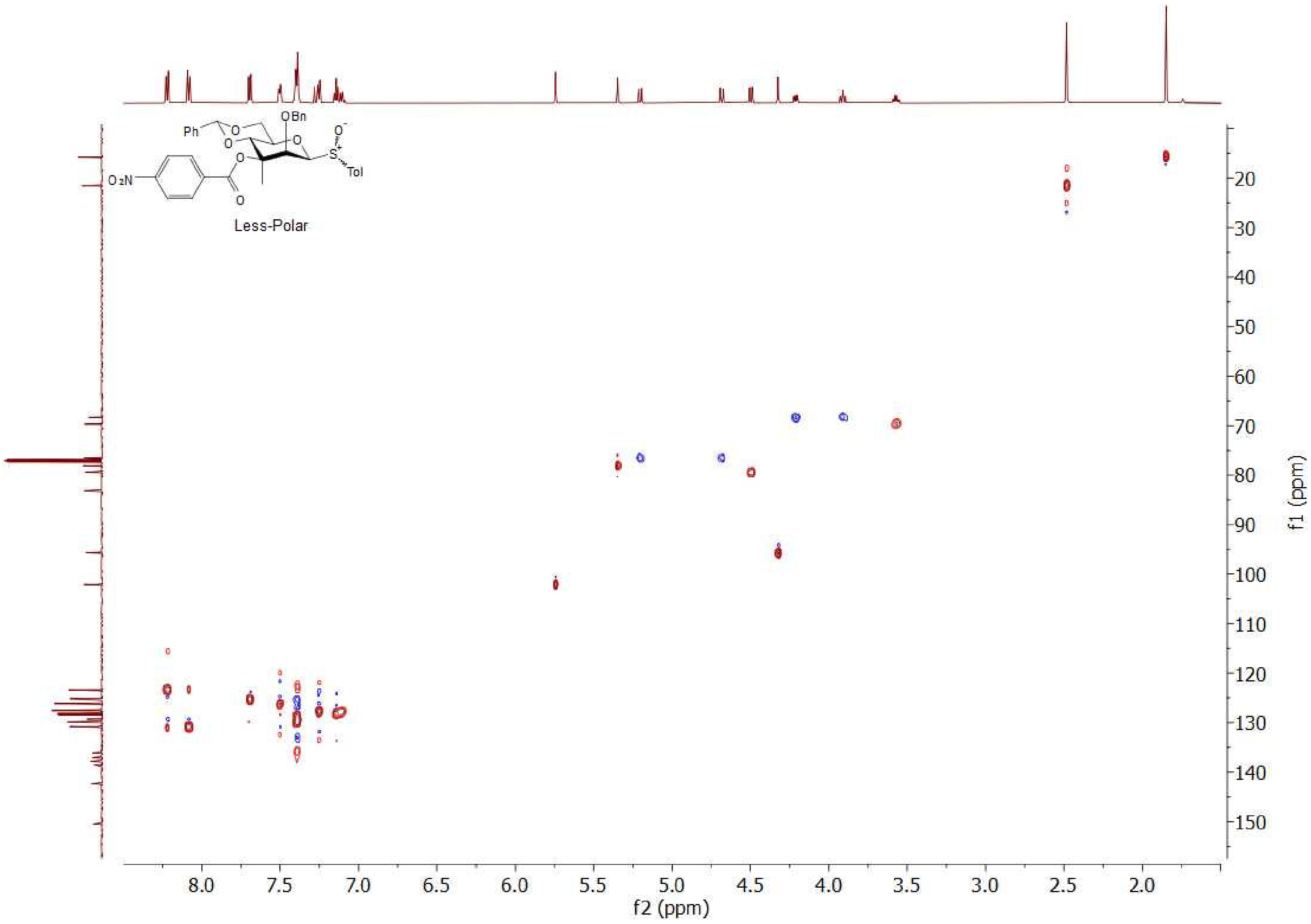

Figure S86. HMBC NMR (600 MHz, $CDCl_3$) spectrum of *p*-methylphenyl 2-*O*-benzyl-4,6-*O*-benzylidene-3-*O*-*p*-nitrobenzoyl-3-*C*-methyl-thio-β-D-mannopyranoside *S*-oxide **41** (Less-polar diastereoisomer):

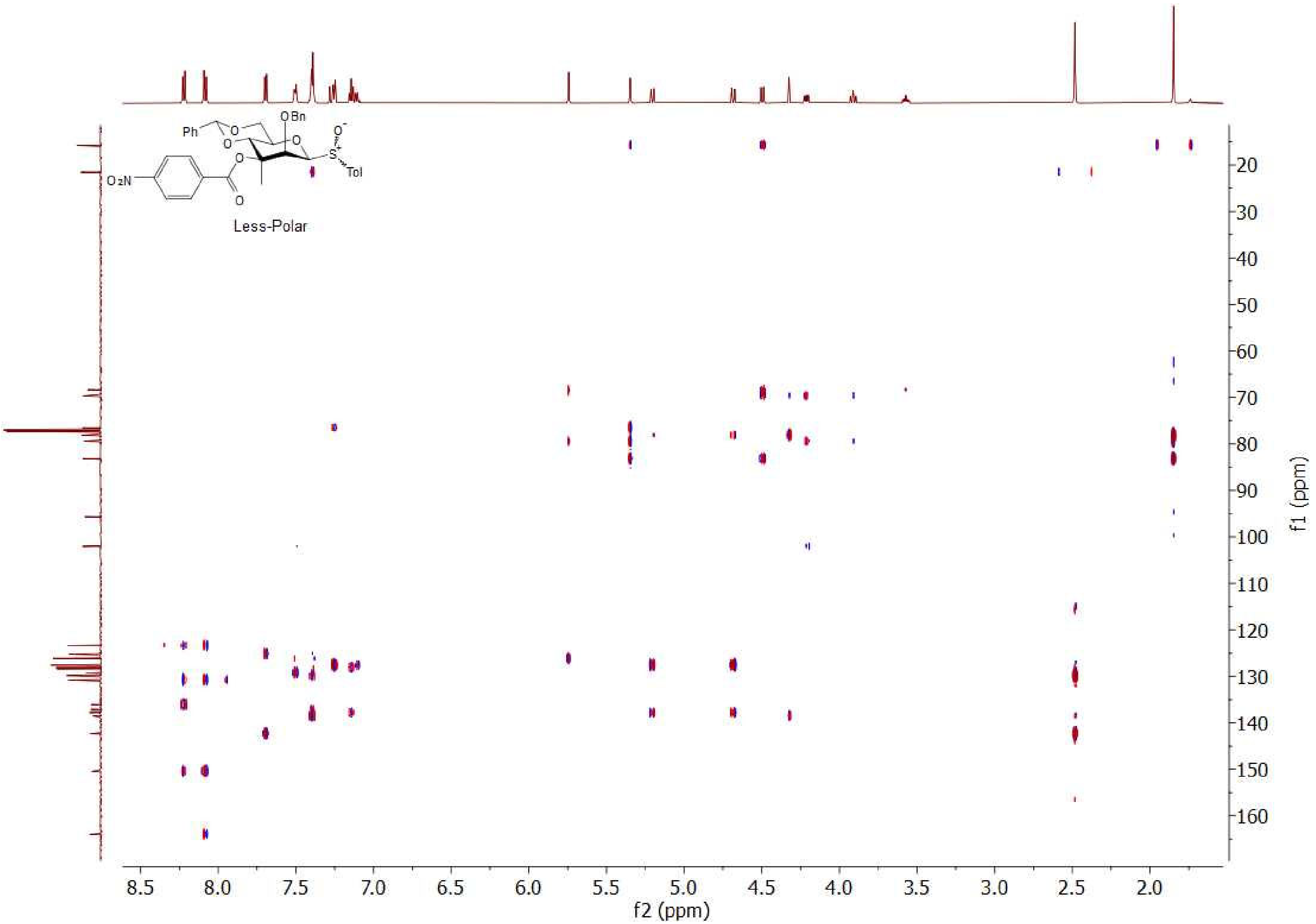

Figure S87. ^{1}H NMR (600 MHz, C_6D_6) spectrum of *p*-methylphenyl 2-*O*-benzyl-4,6-*O*-benzylidene-3-*O*-*p*-nitrobenzoyl-3-*C*-methyl-thio-β-D-mannopyranoside *S*-oxide **41** (Polar diastereoisomer):

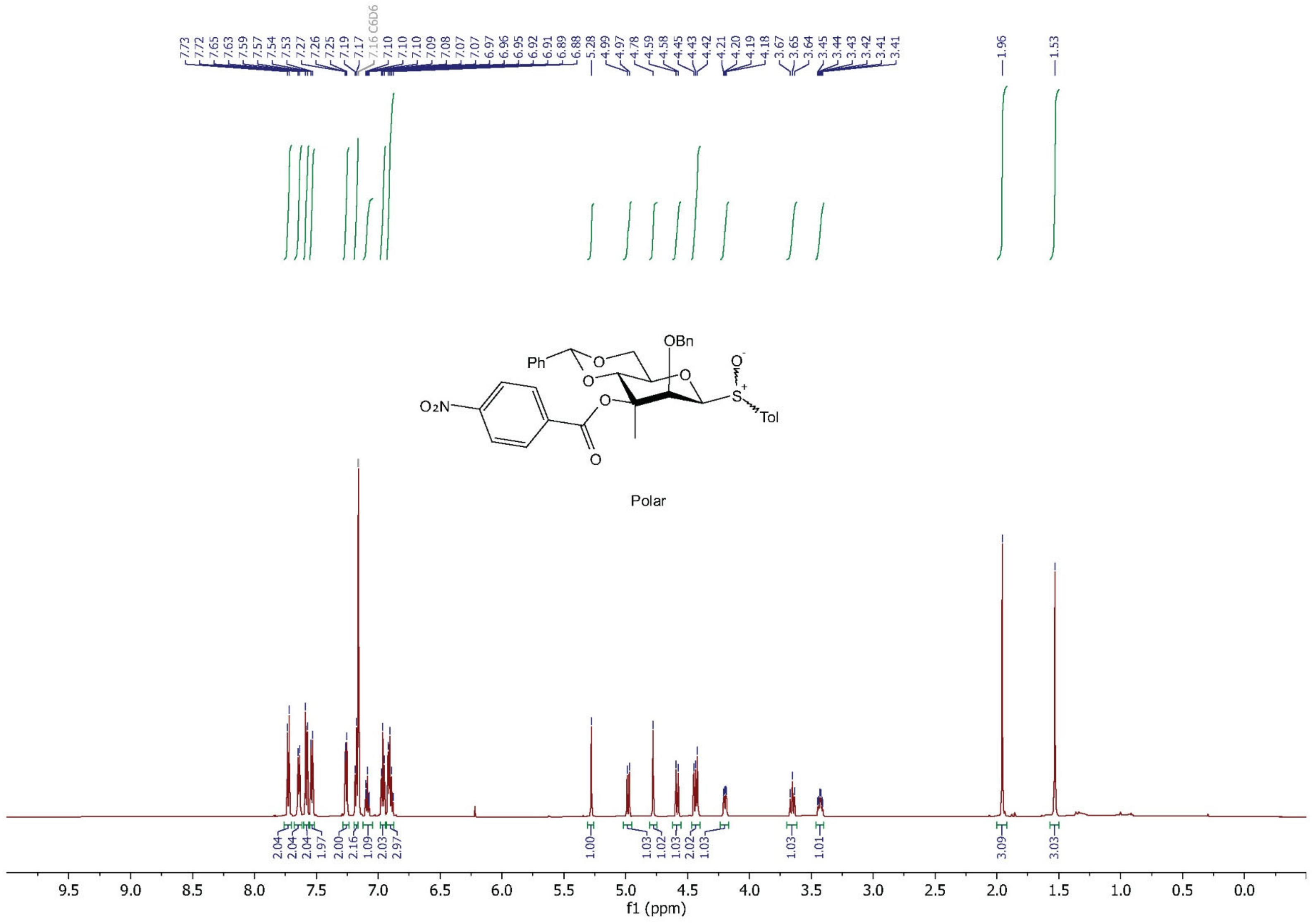

Figure S88. COSY NMR (600 MHz, C_6D_6) spectrum of *p*-methylphenyl 2-*O*-benzyl-4,6-*O*-benzylidene-3-*O*-*p*-nitrobenzoyl-3-*C*-methyl-thio-β-D-mannopyranoside *S*-oxide **41** (Polar diastereoisomer):

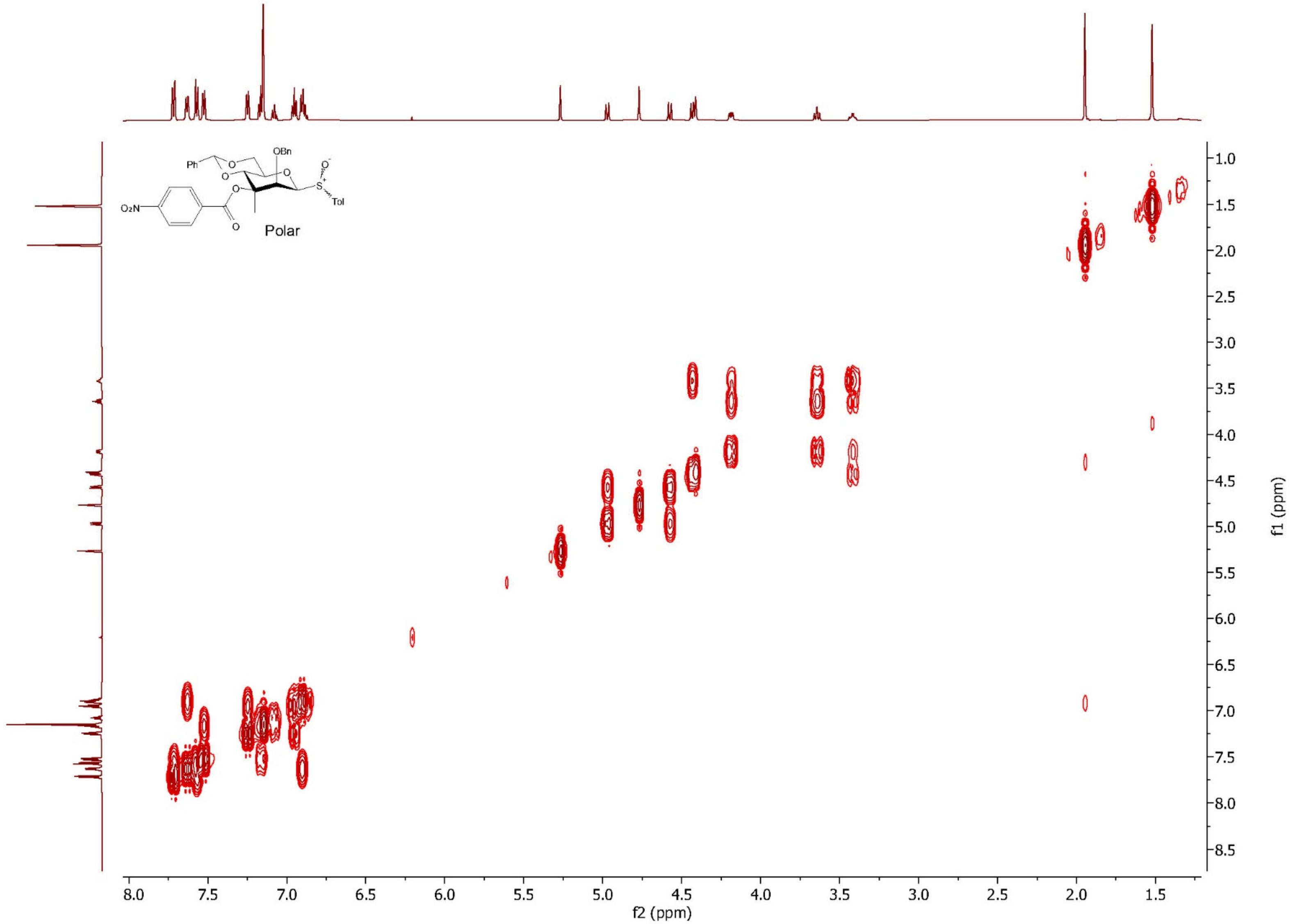

Figure S89. $^{13}C\{^{1}H\}$ NMR (151 MHz, C_6D_6) spectrum of *p*-methylphenyl 2-*O*-benzyl-4,6-*O*-benzylidene-3-*O*-*p*-nitrobenzoyl-3-*C*-methyl-thio-β-D-mannopyranoside *S*-oxide **41** (Polar diastereoisomer):

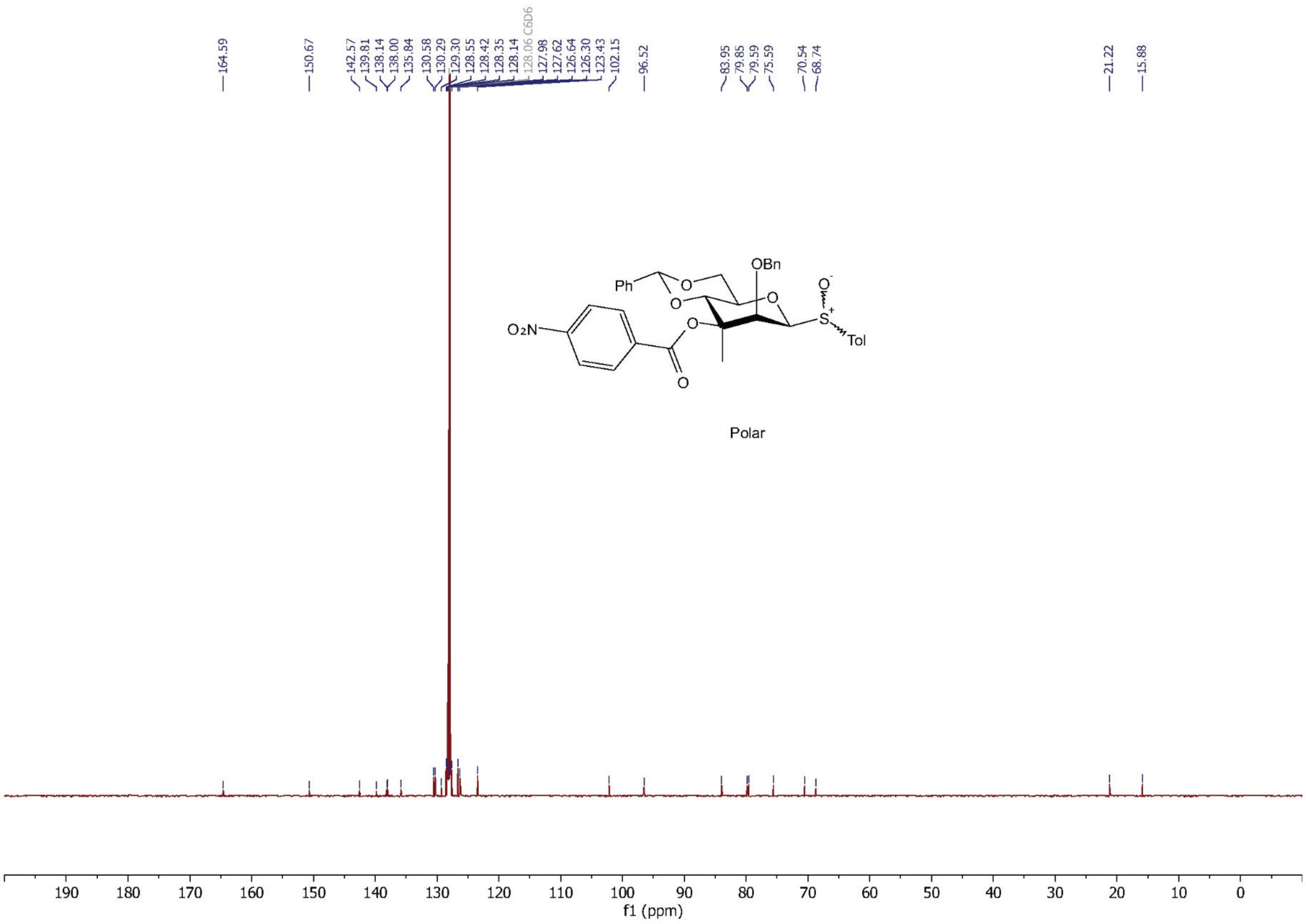

Figure S90. $^{13}C\{^{1}H\}$ DEPT NMR (151 MHz, C_6D_6) spectrum of *p*-methylphenyl 2-*O*-benzyl-4,6-*O*-benzylidene-3-*O*-*p*-nitrobenzoyl-3-*C*-methyl-thio-β-D-mannopyranoside *S*-oxide **41** (Polar diastereoisomer):

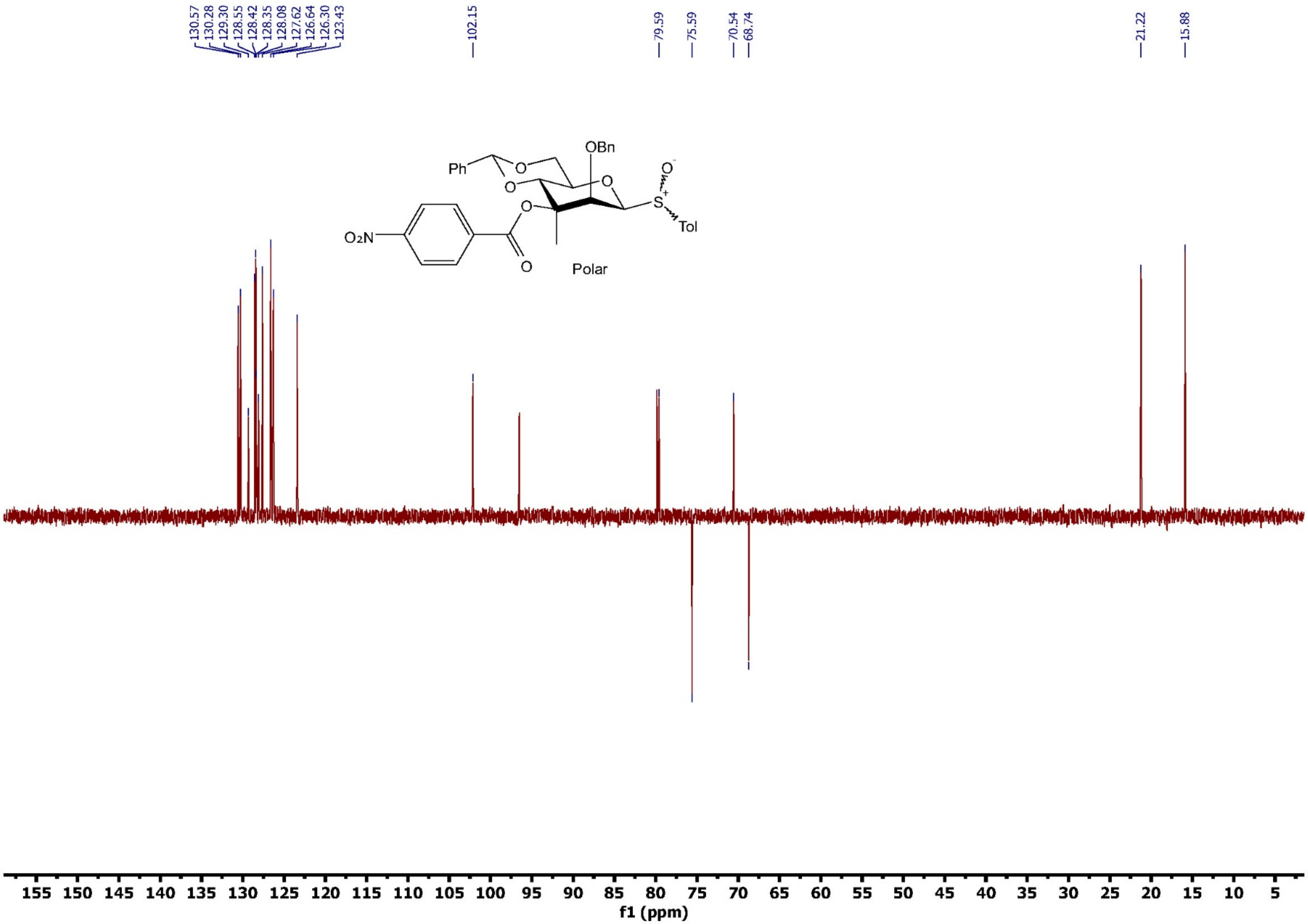

Figure S91. HSQC NMR (600 MHz, C_6D_6) spectrum of *p*-methylphenyl 2-*O*-benzyl-4,6-*O*-benzylidene-3-*O*-*p*-nitrobenzoyl-3-*C*-methyl-thio-β-D-mannopyranoside *S*-oxide **41** (Polar diastereoisomer):

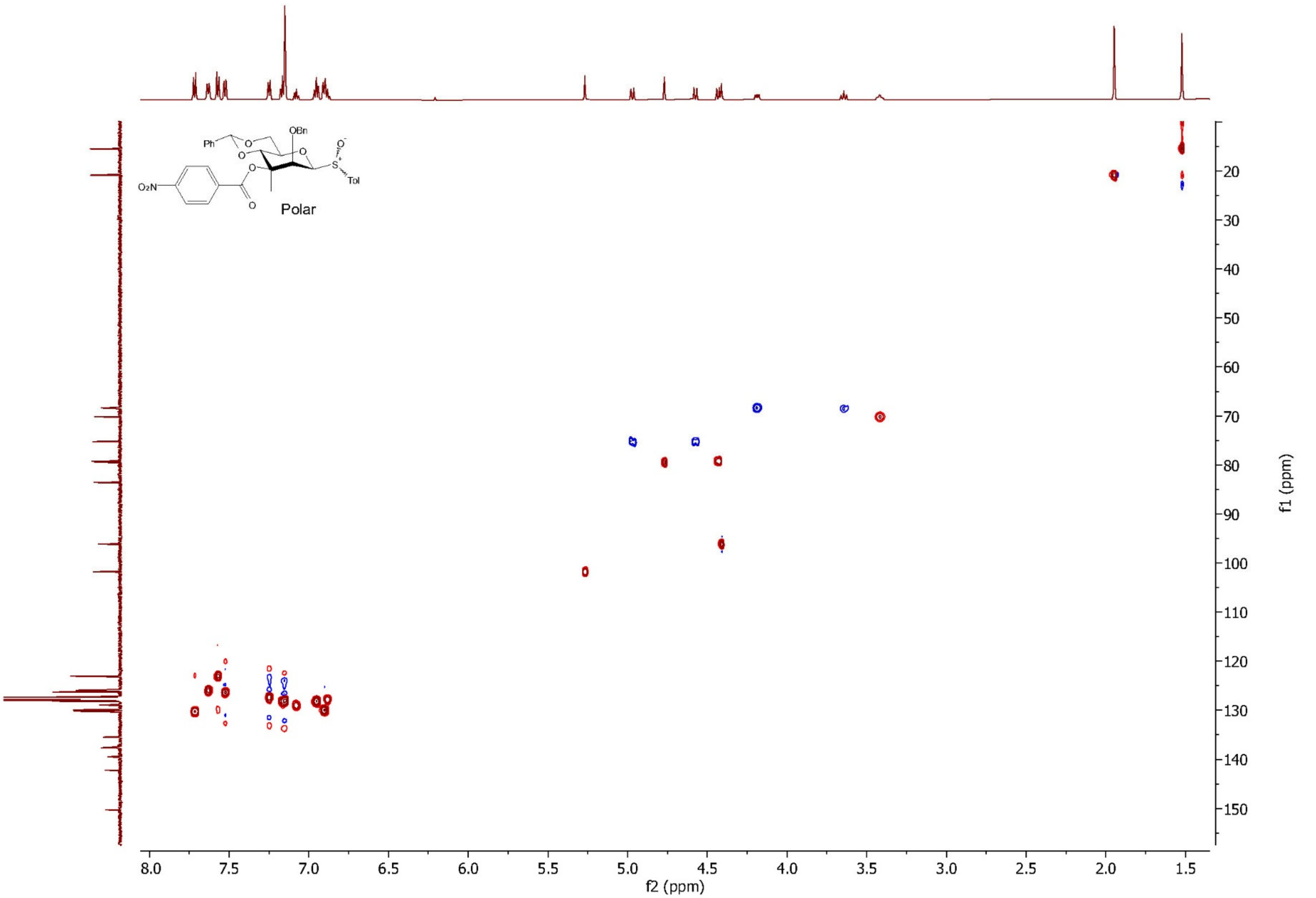

Figure S92. HMBC NMR (600 MHz, C_6D_6) spectrum of *p*-methylphenyl 2-*O*-benzyl-4,6-*O*-benzylidene-3-*O*-*p*-nitrobenzoyl-3-*C*-methyl-thio-β-D-mannopyranoside *S*-oxide **41** (Polar diastereoisomer):

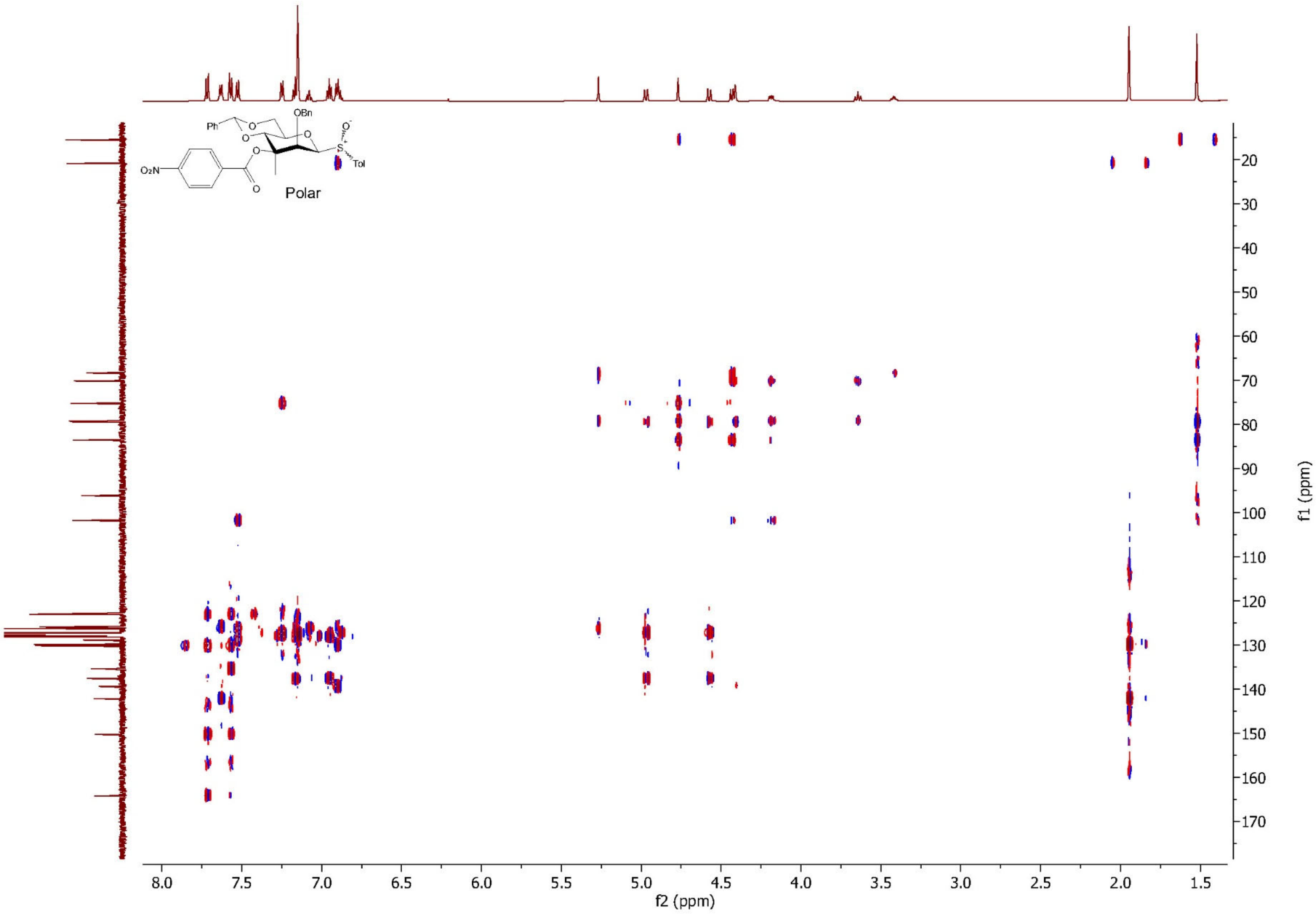

Figure S93. ^{1}H NMR (600 MHz, CD_3OD) spectrum of 4,6-*O*-benzylidene-3-*C*-methyl-D-mannopyranose **42**:

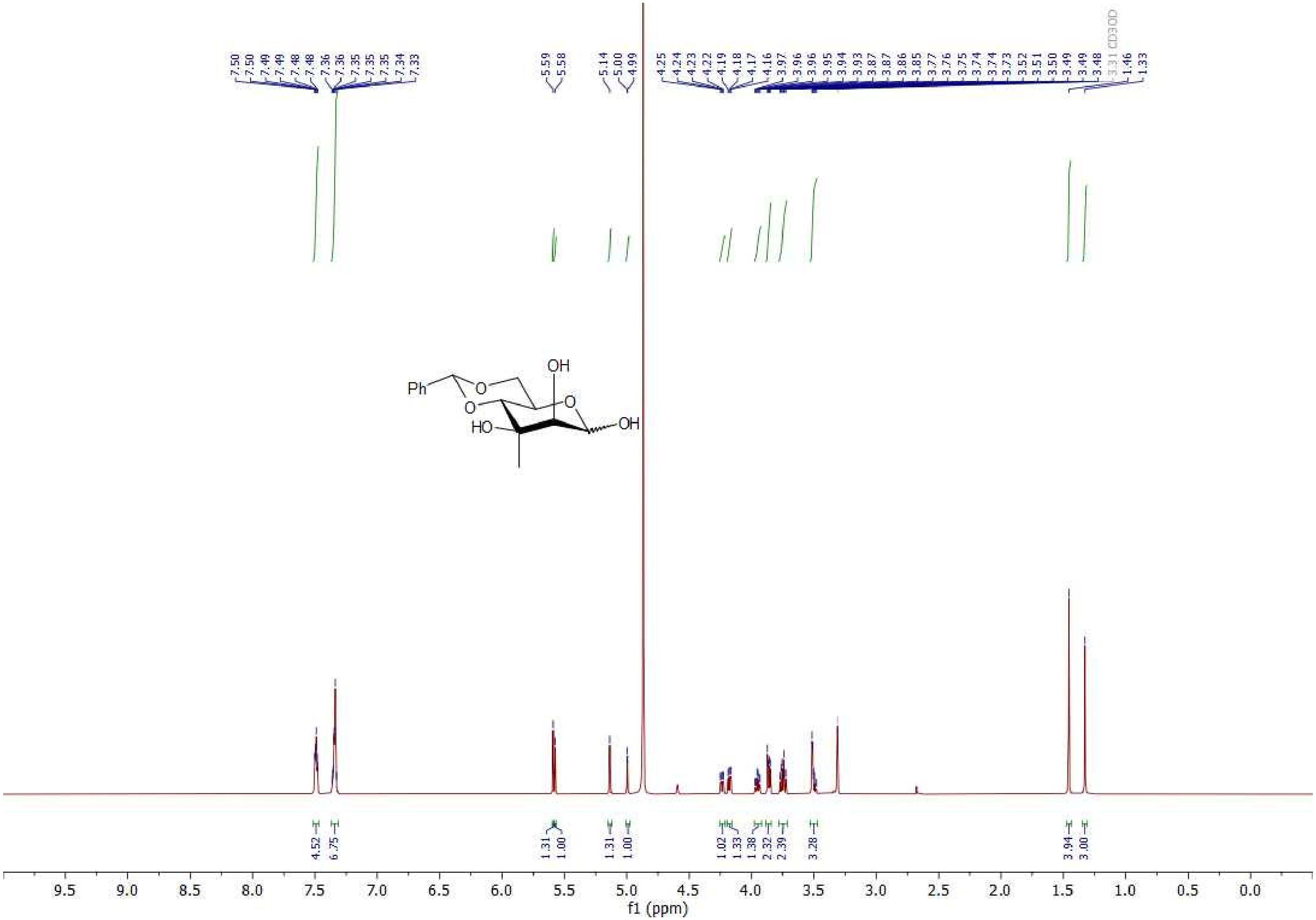

Figure S94. COSY NMR (600 MHz, CD_3OD) spectrum of 4,6-*O*-benzylidene-3-*C*-methyl-D-mannopyranose **42**:

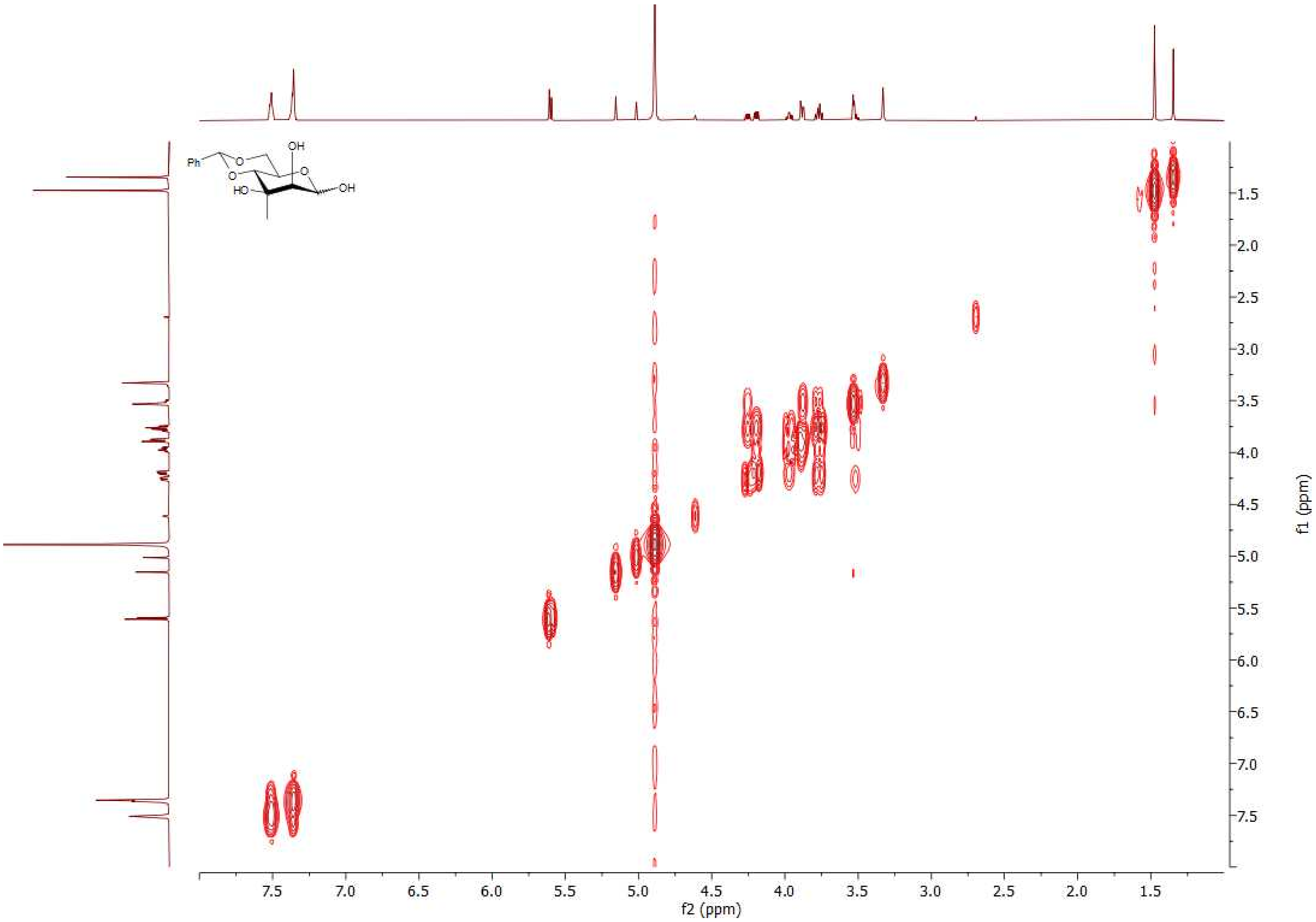

Figure S95. $^{13}C\{^{1}H\}$ NMR (151 MHz, CD_3OD) spectrum of 4,6-*O*-benzylidene-3-*C*-methyl-D-mannopyranose **42**:

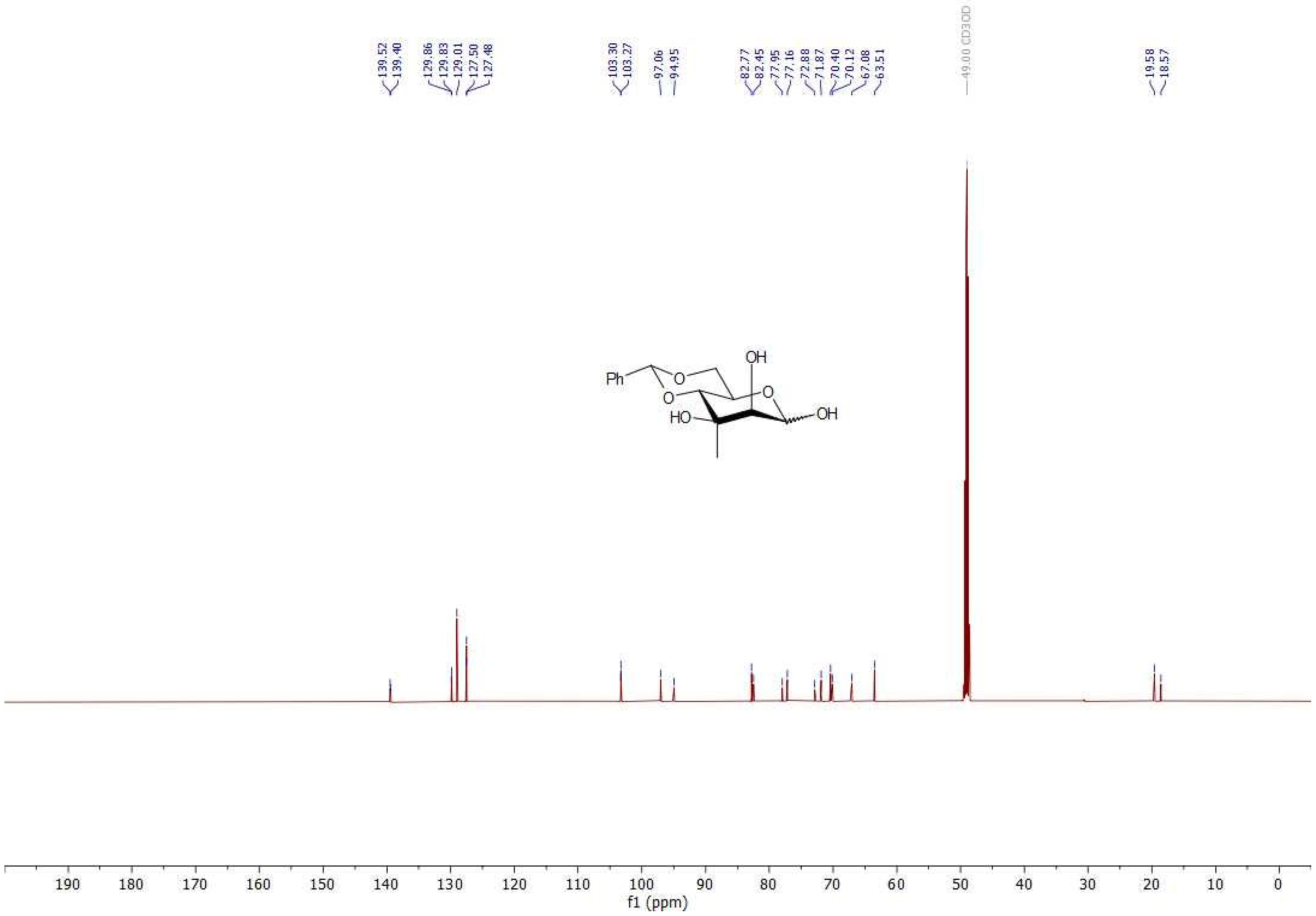

Figure S96. $^{13}C\{^1H\}$ DEPT NMR (151 MHz, CD_3OD) spectrum of 4,6-*O*-benzylidene-3-*C*-methyl-D-mannopyranose **42**:

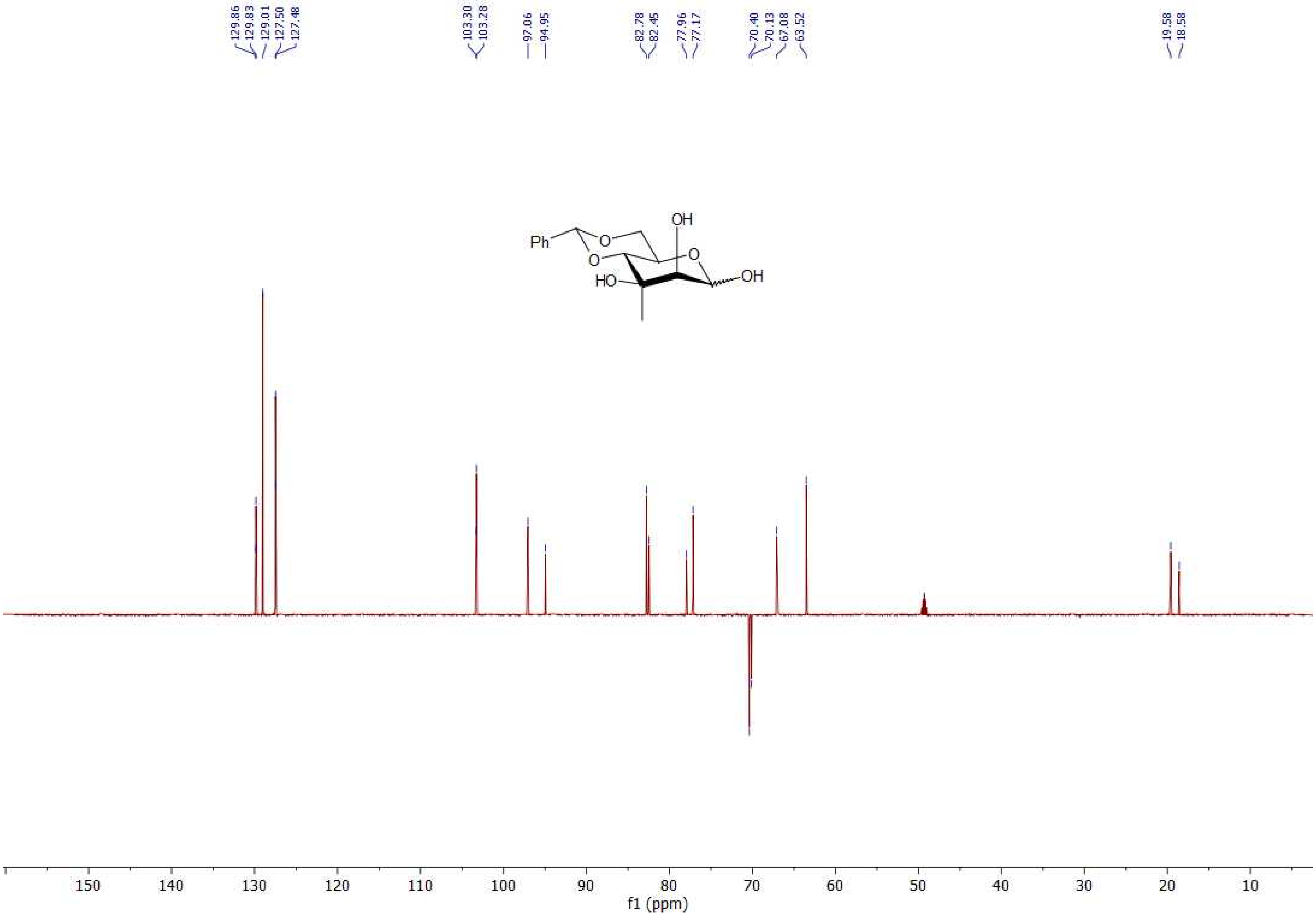

Figure S97. HSQC NMR (600 MHz, CD_3OD) spectrum of 4,6-*O*-benzylidene-3-*C*-methyl-D-mannopyranose **42**:

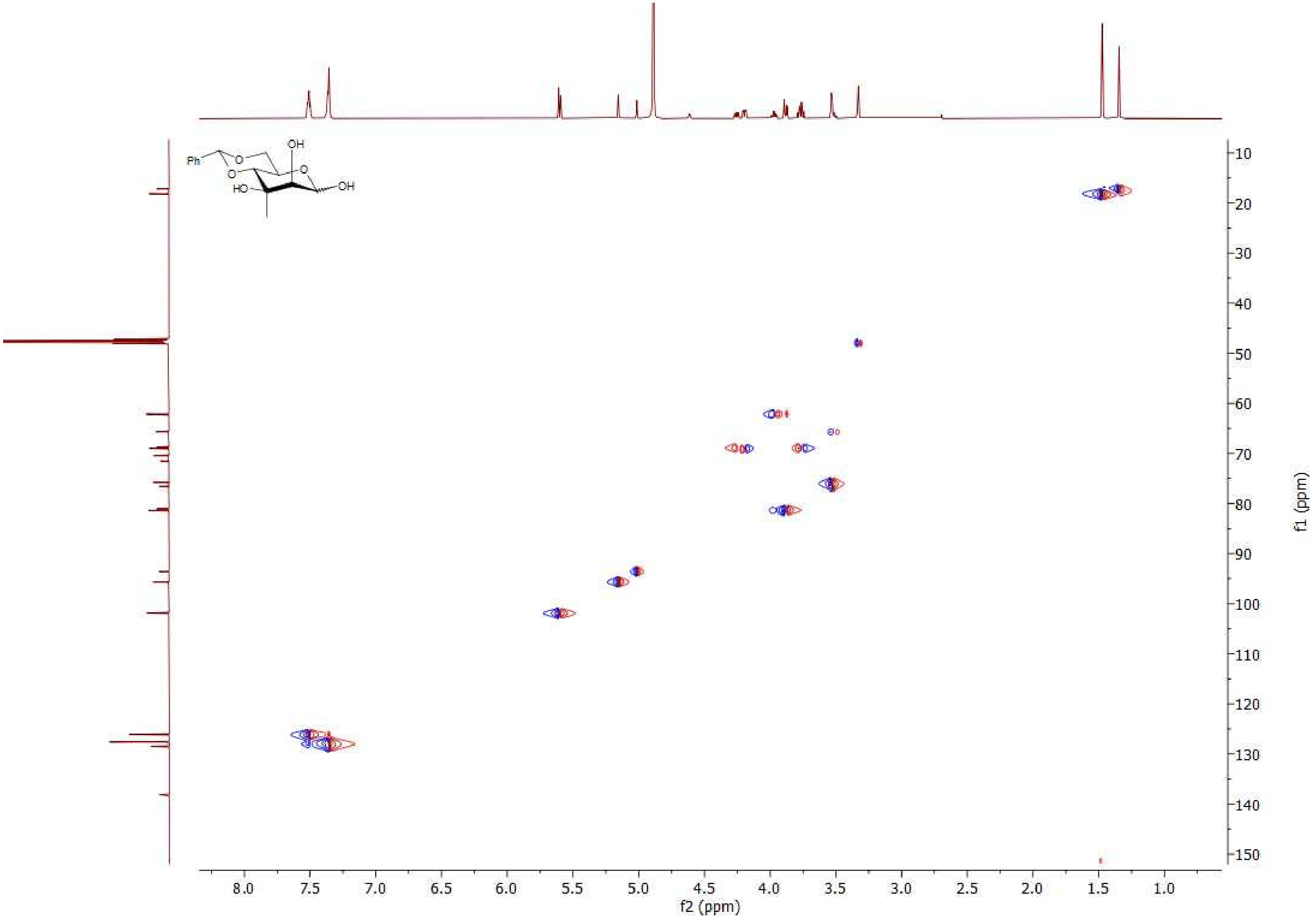

Figure S98. HMBC NMR (600 MHz, CD_3OD) spectrum of 4,6-*O*-benzylidene-3-*C*-methyl-D-mannopyranose **42**:

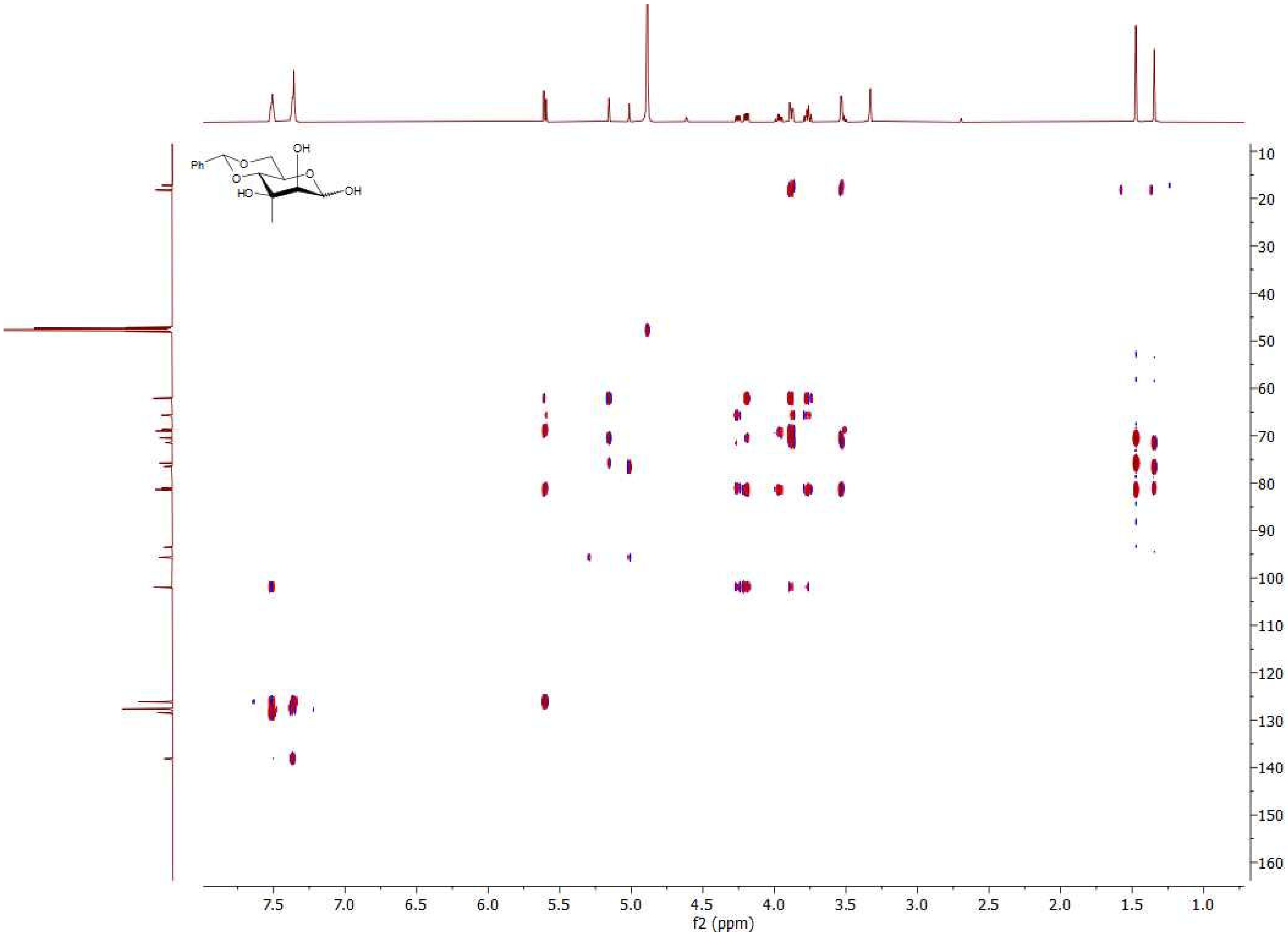

Figure S99. ^{1}H NMR (600 MHz, $CDCl_3$) spectrum of *p*-methylphenyl 4,6-*O*-benzylidene-3-*C*-methyl-thio-α-D-mannopyranoside **43**:

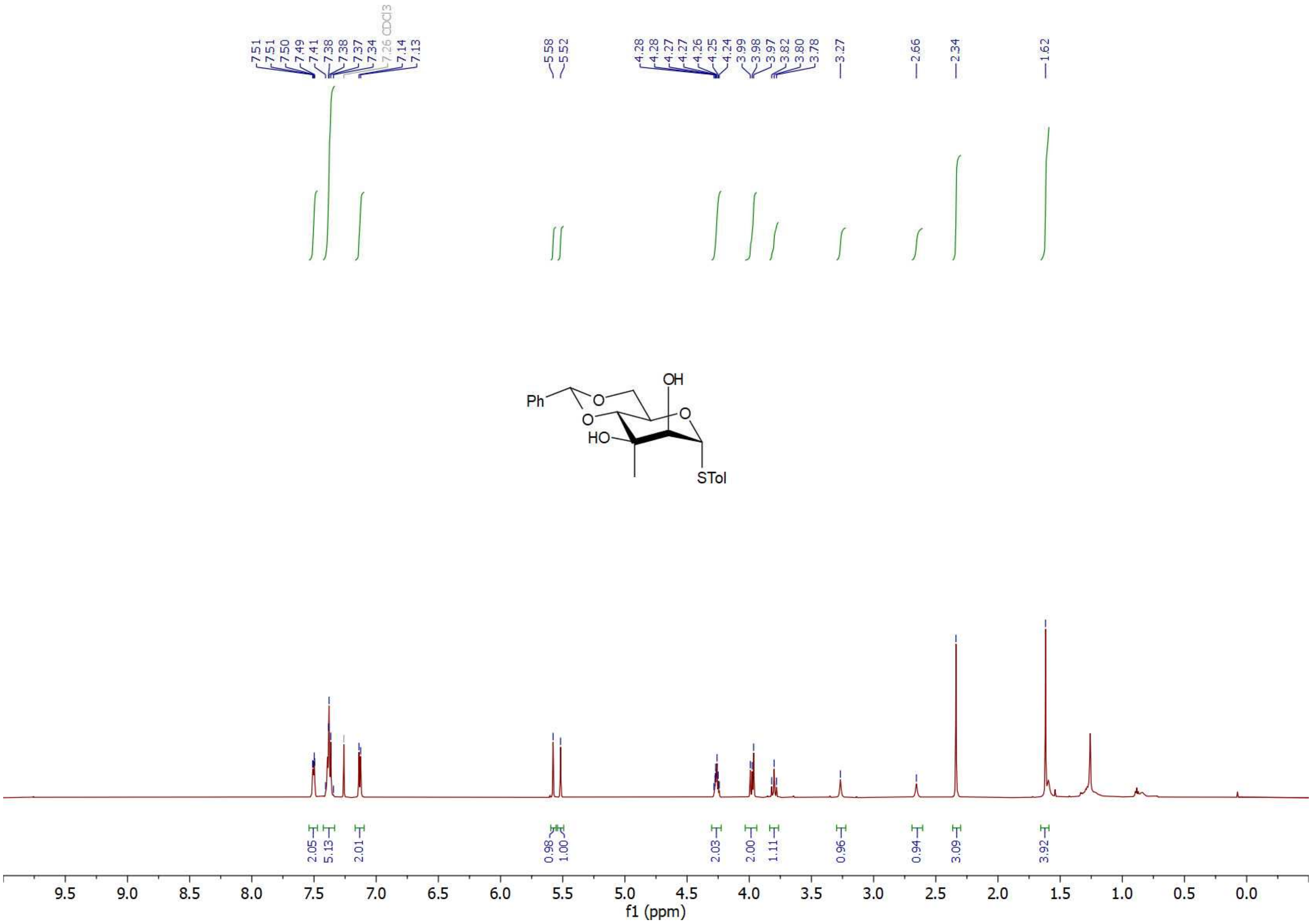

Figure S100. COSY NMR (600 MHz, CDCl$_3$) spectrum of *p*-methylphenyl 4,6-*O*-benzylidene-3-*C*-methyl-thio-α-D-mannopyranoside **43**:

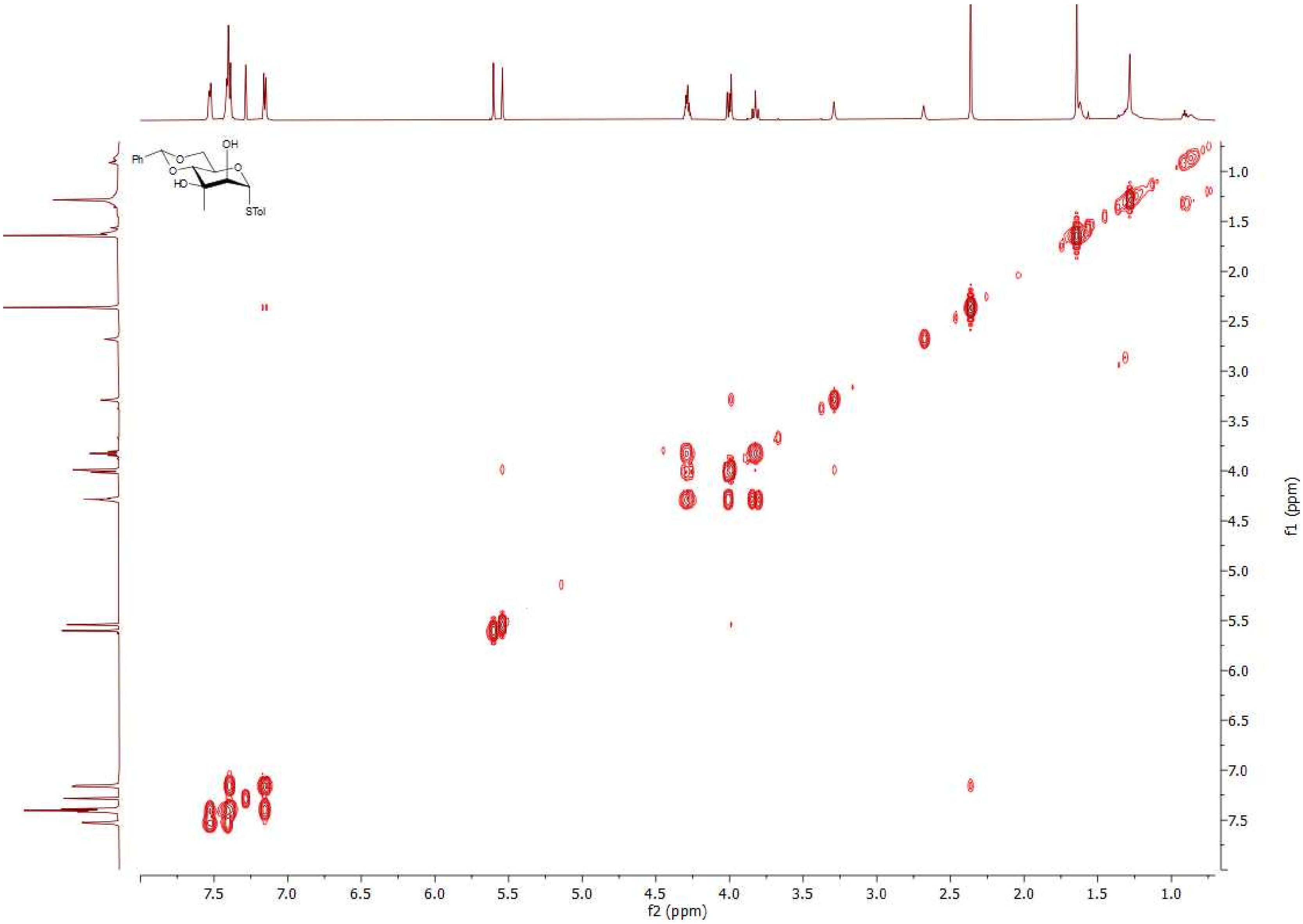

Figure S101. $^{13}C\{^1H\}$ NMR (151 MHz, $CDCl_3$) spectrum of *p*-methylphenyl 4,6-*O*-benzylidene-3-*C*-methyl-thio-α-D-mannopyranoside **43**:

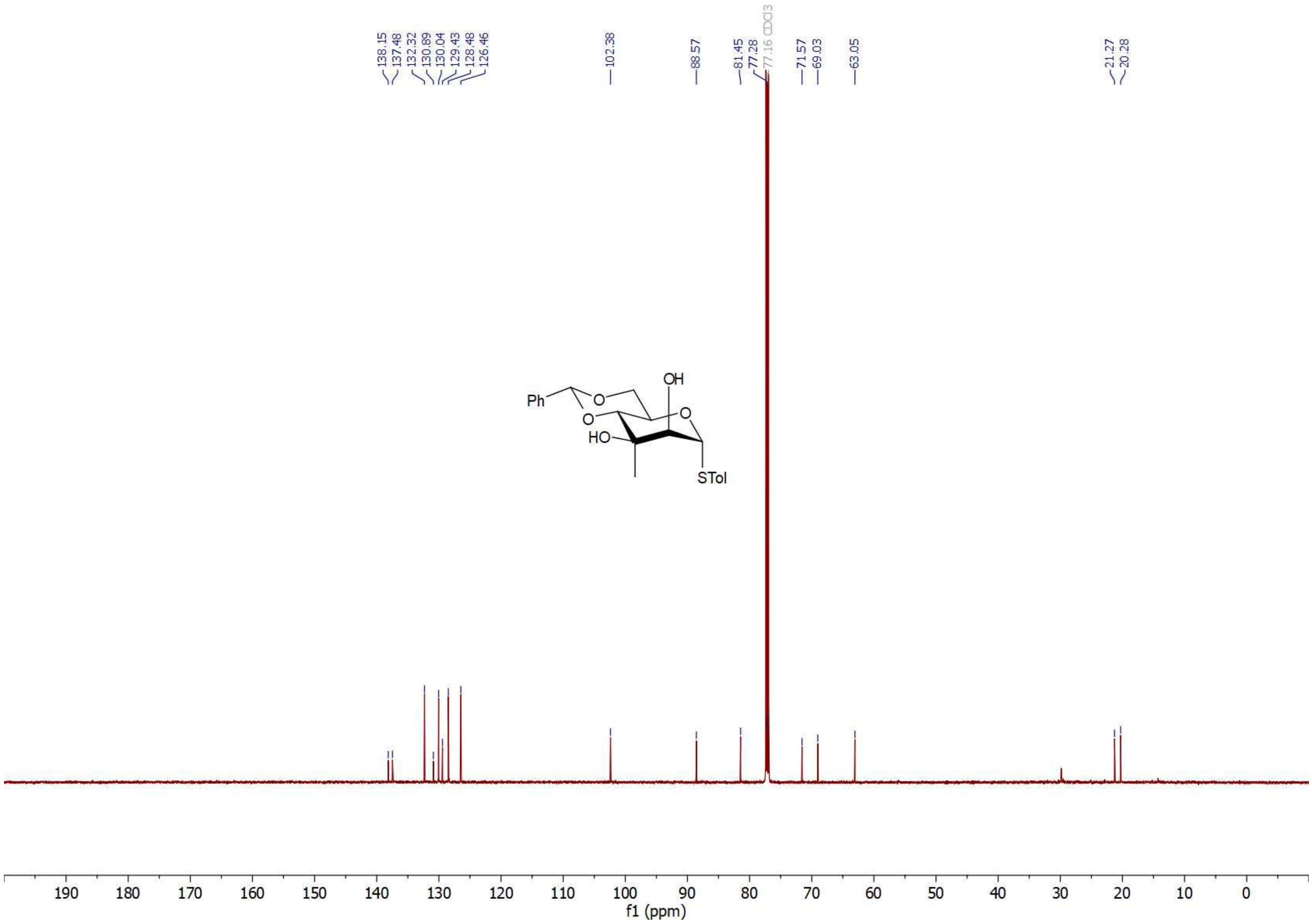

Figure S102. $^{13}C\{^{1}H\}$ DEPT NMR (151 MHz, $CDCl_3$) spectrum of *p*-methylphenyl 4,6-*O*-benzylidene-3-*C*-methyl-thio-α-D-mannopyranoside **43**:

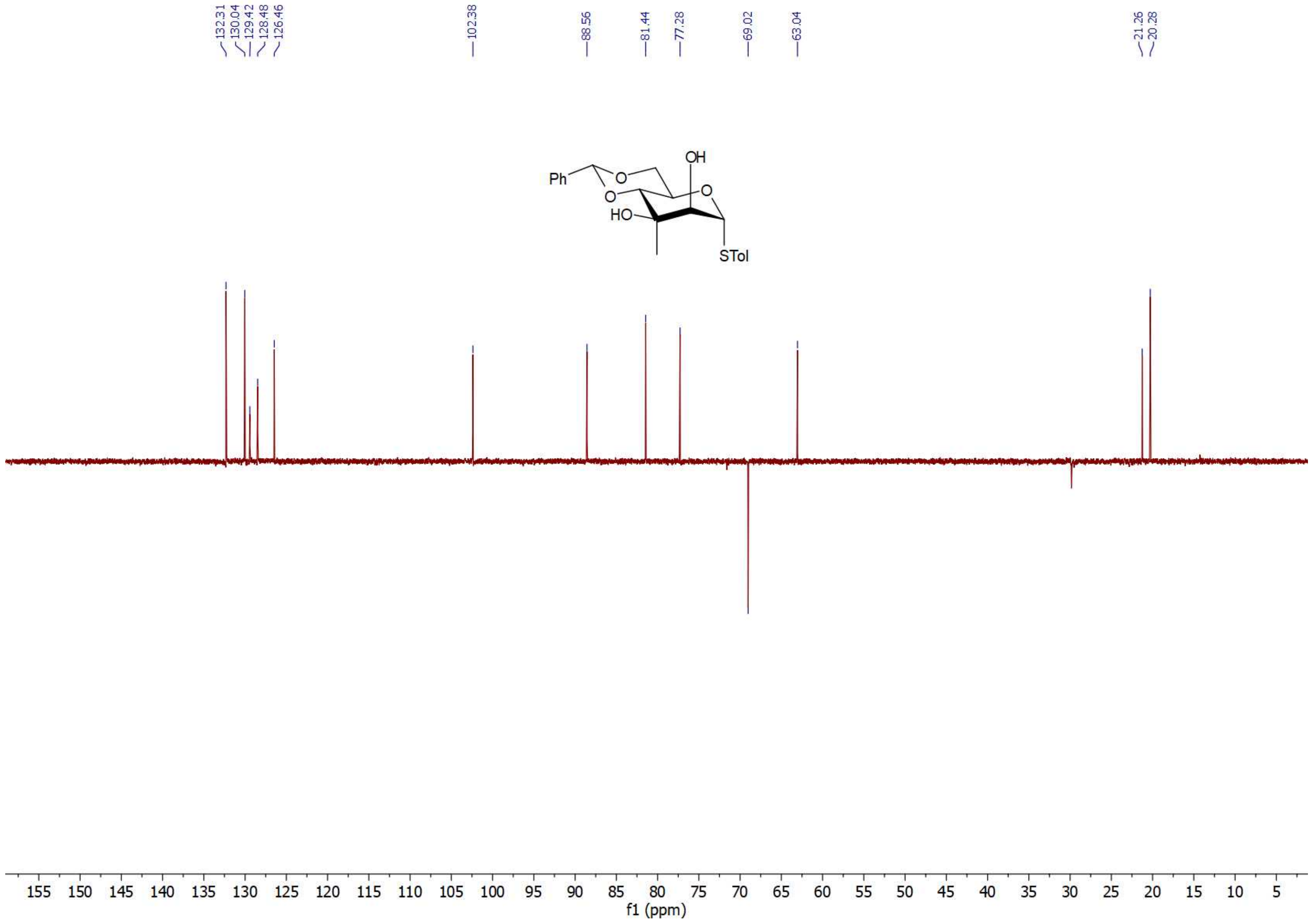

Figure S103. ^{13}C (C-H coupled) NMR (151 MHz, $CDCl_3$) spectrum of *p*-methylphenyl 4,6-*O*-benzylidene-3-*C*-methyl-thio-α-D-mannopyranoside **43**:

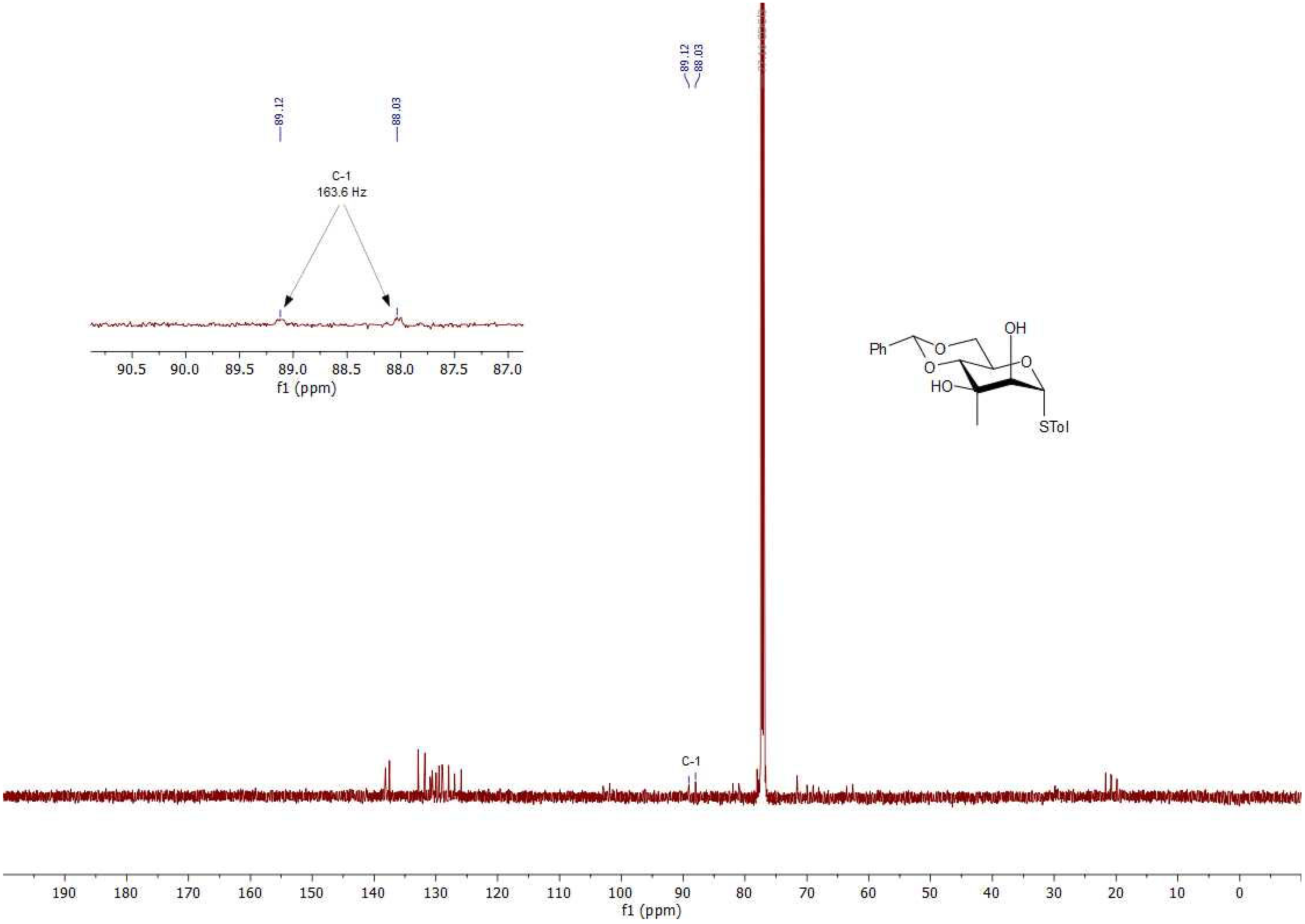

Figure S104. HSQC NMR (600 MHz, $CDCl_3$) spectrum of *p*-methylphenyl 4,6-*O*-benzylidene-3-*C*-methyl-thio-α-D-mannopyranoside **43**:

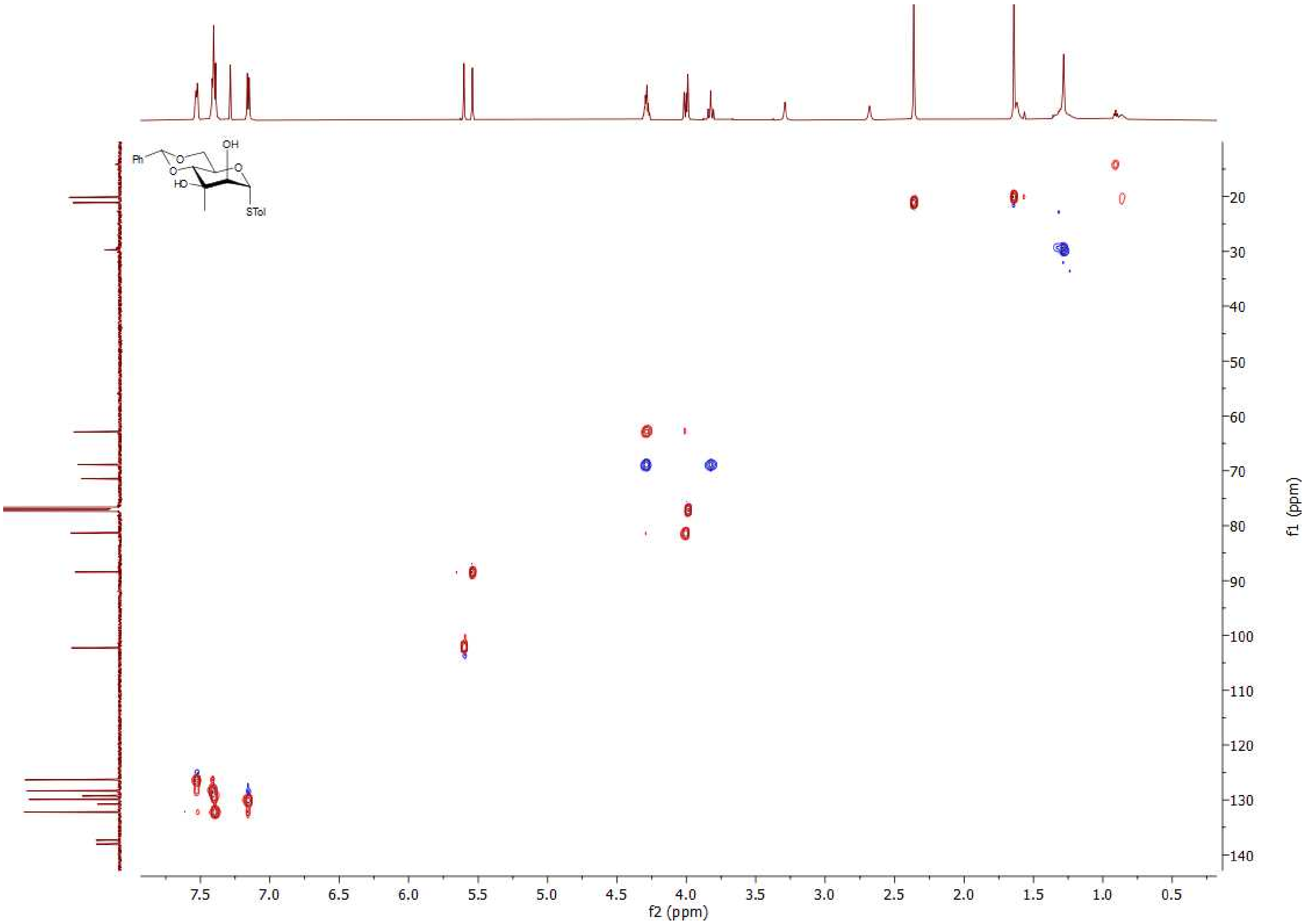

Figure S105. HMBC NMR (600 MHz, $CDCl_3$) spectrum of *p*-methylphenyl 4,6-*O*-benzylidene-3-*C*-methyl-thio-α-D-mannopyranoside **43**:

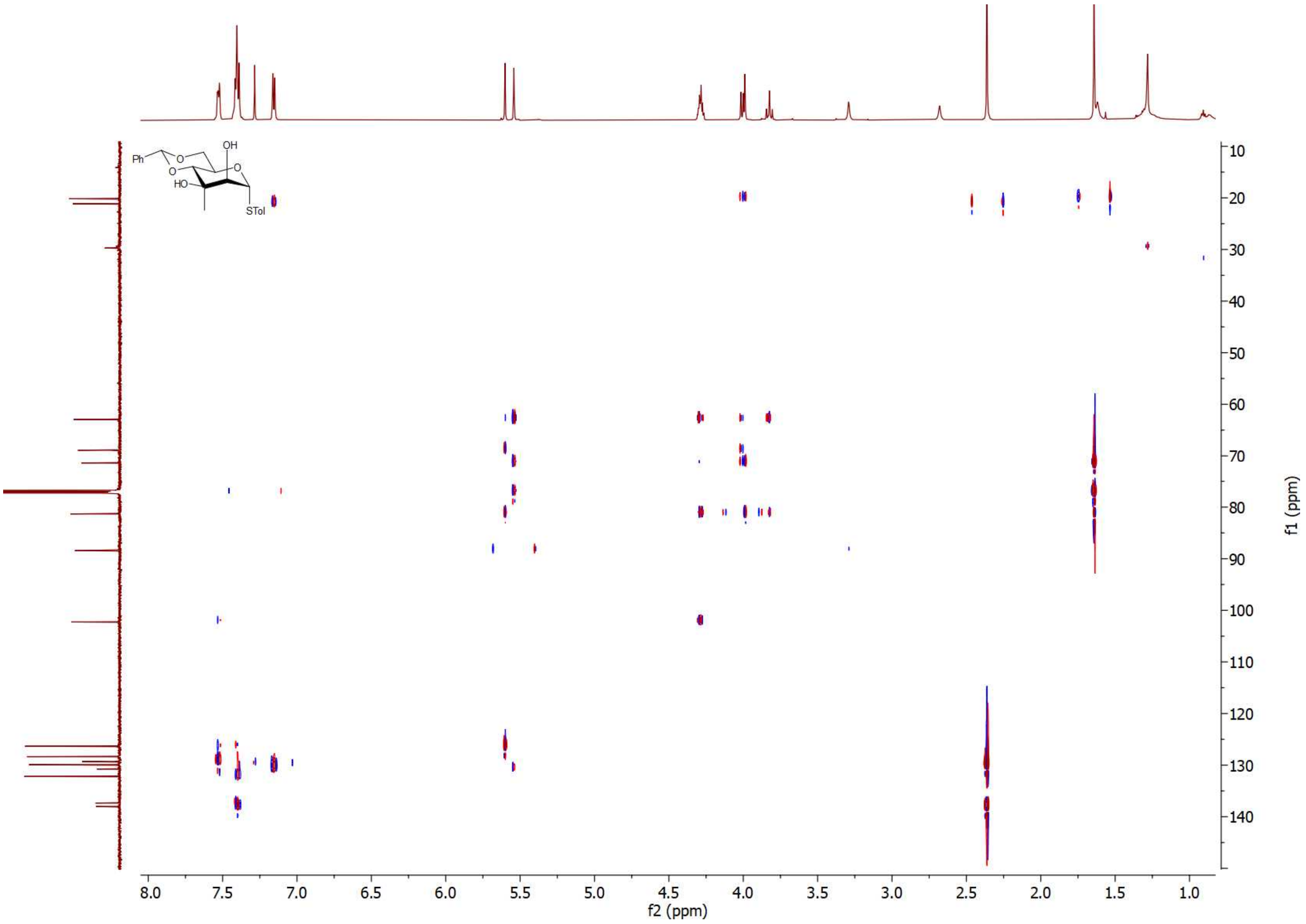

Figure S106. ^{1}H NMR (600 MHz, $CDCl_3$) spectrum of *p*-methylphenyl 2-*O*-benzyl-4,6-*O*-benzylidene-3-*C*-methyl-thio-α-D-mannopyranoside **44**:

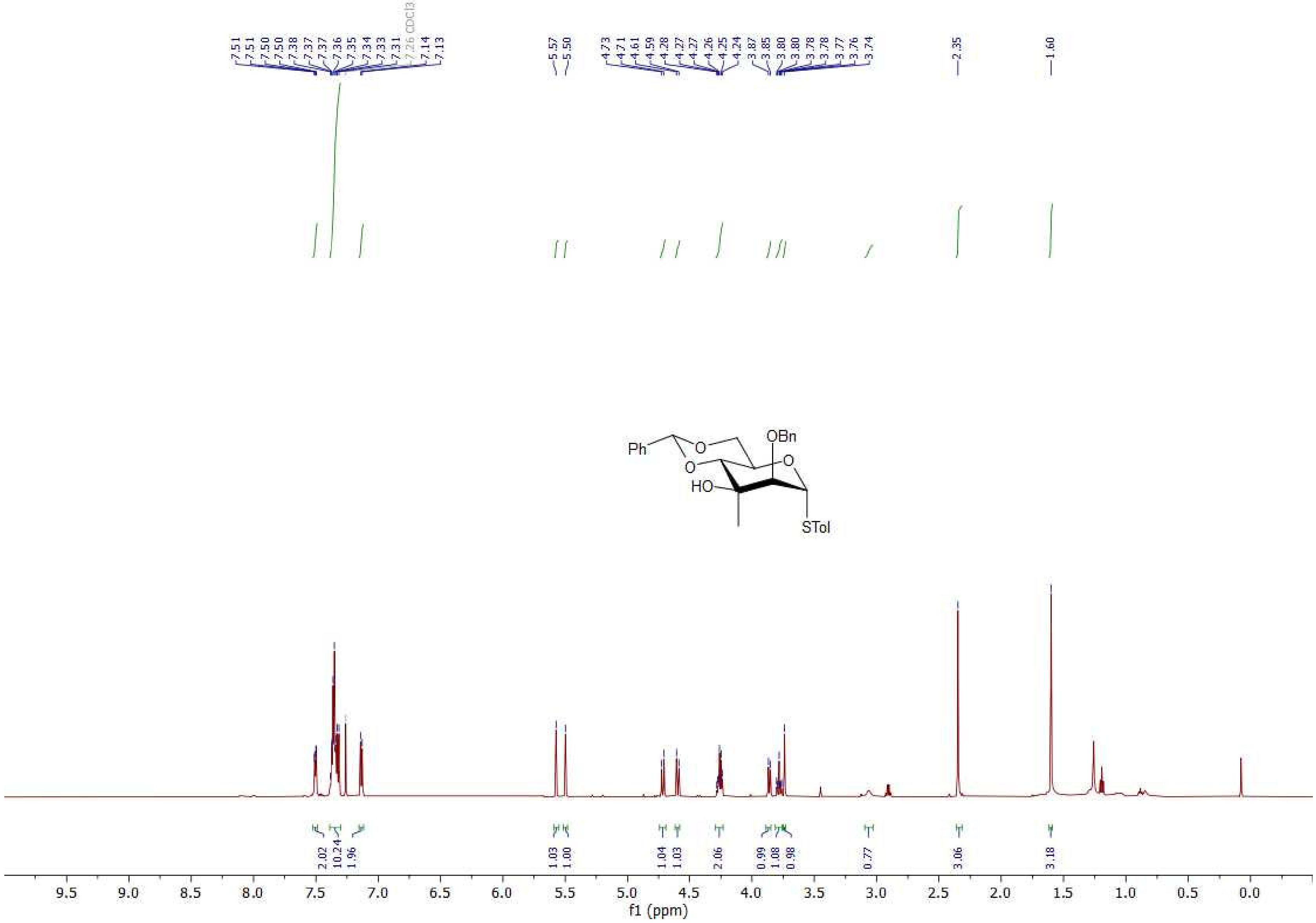

Figure S107. COSY NMR (600 MHz, $CDCl_3$) spectrum of *p*-methylphenyl 2-*O*-benzyl-4,6-*O*-benzylidene-3-*C*-methyl-thio-α-D-mannopyranoside **44**:

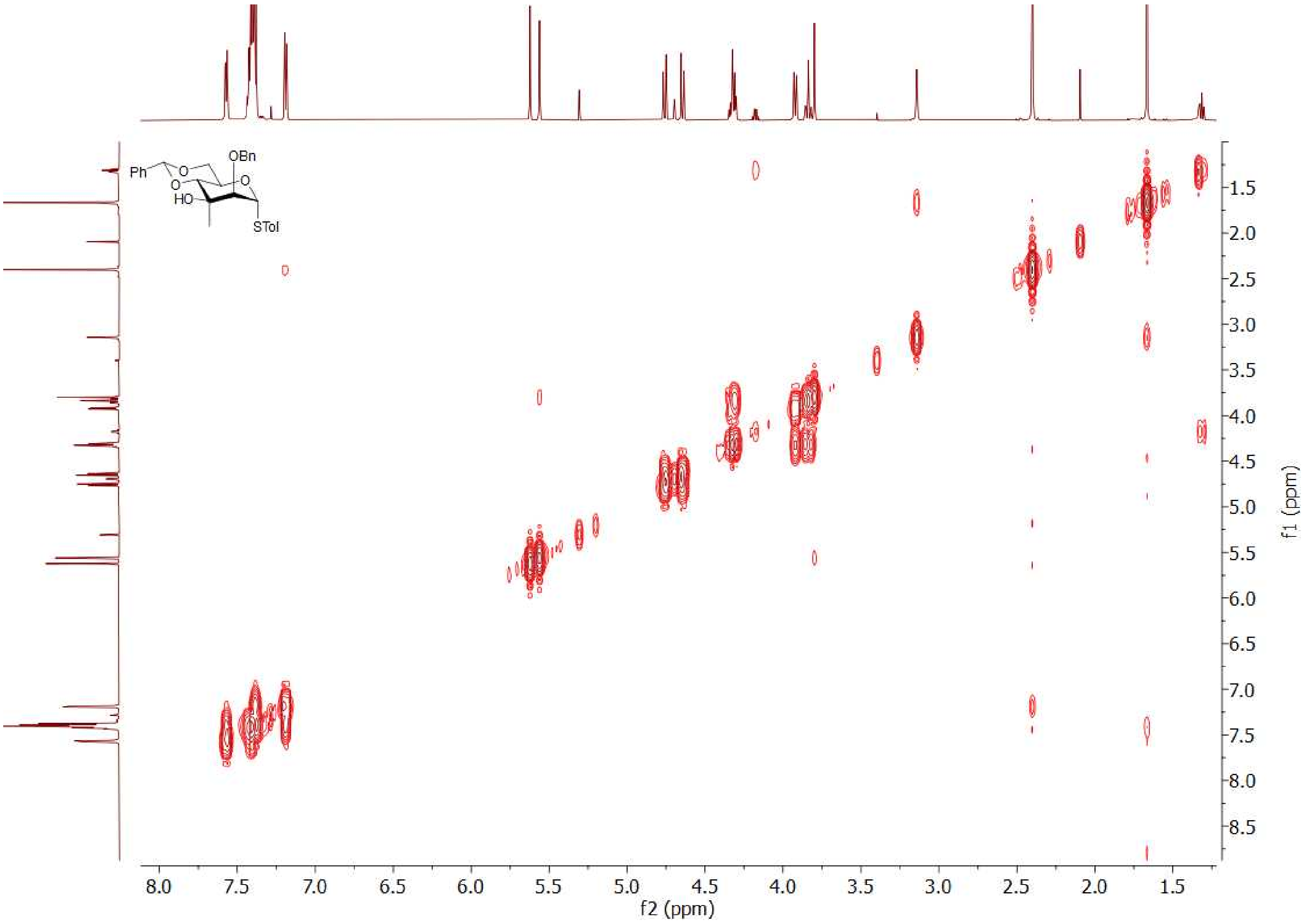

Figure S108. $^{13}C\{^{1}H\}$ NMR (151 MHz, $CDCl_3$) spectrum of *p*-methylphenyl 2-*O*-benzyl-4,6-*O*-benzylidene-3-*C*-methyl-thio-α-D-mannopyranoside **44**:

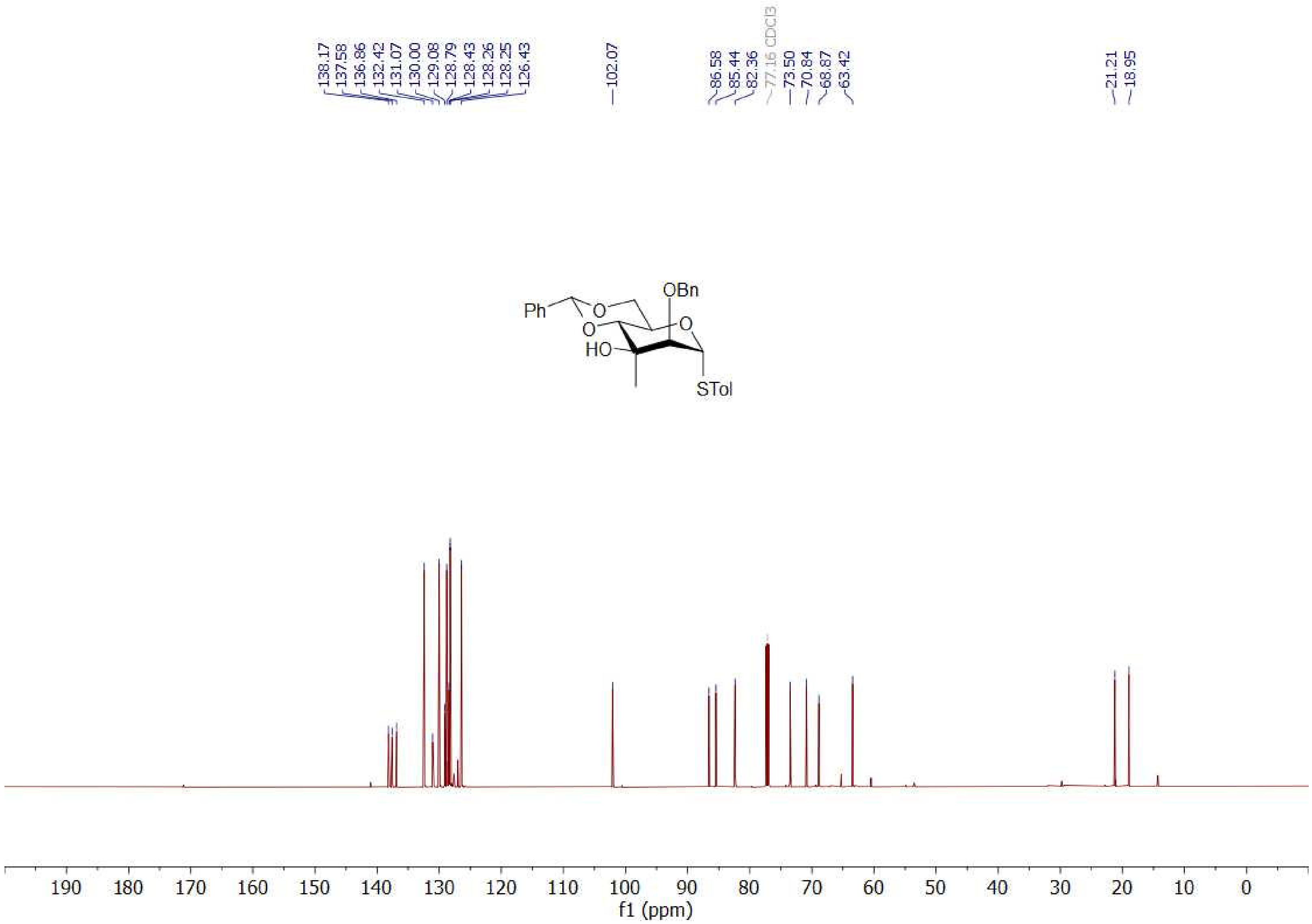

Figure S109. $^{13}C\{^{1}H\}$ DEPT NMR (151 MHz, $CDCl_3$) spectrum of *p*-methylphenyl 2-*O*-benzyl-4,6-*O*-benzylidene-3-*C*-methyl-thio-α-D-mannopyranoside **44**:

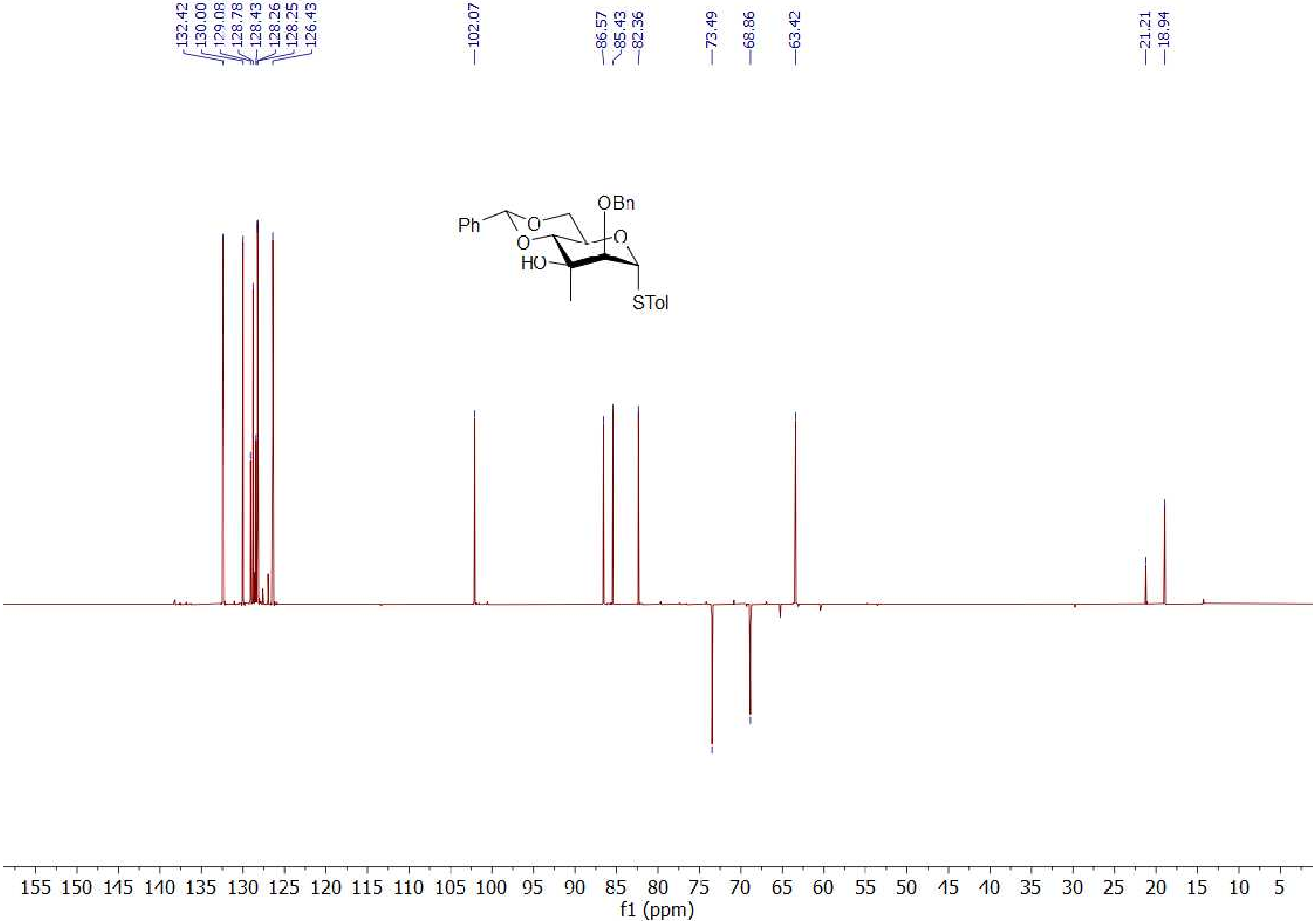

Figure S110. ^{13}C (C-H coupled) NMR (151 MHz, $CDCl_3$) spectrum of *p*-methylphenyl 2-*O*-benzyl-4,6-*O*-benzylidene-3-*C*-methyl-thio-α-D-mannopyranoside **44**:

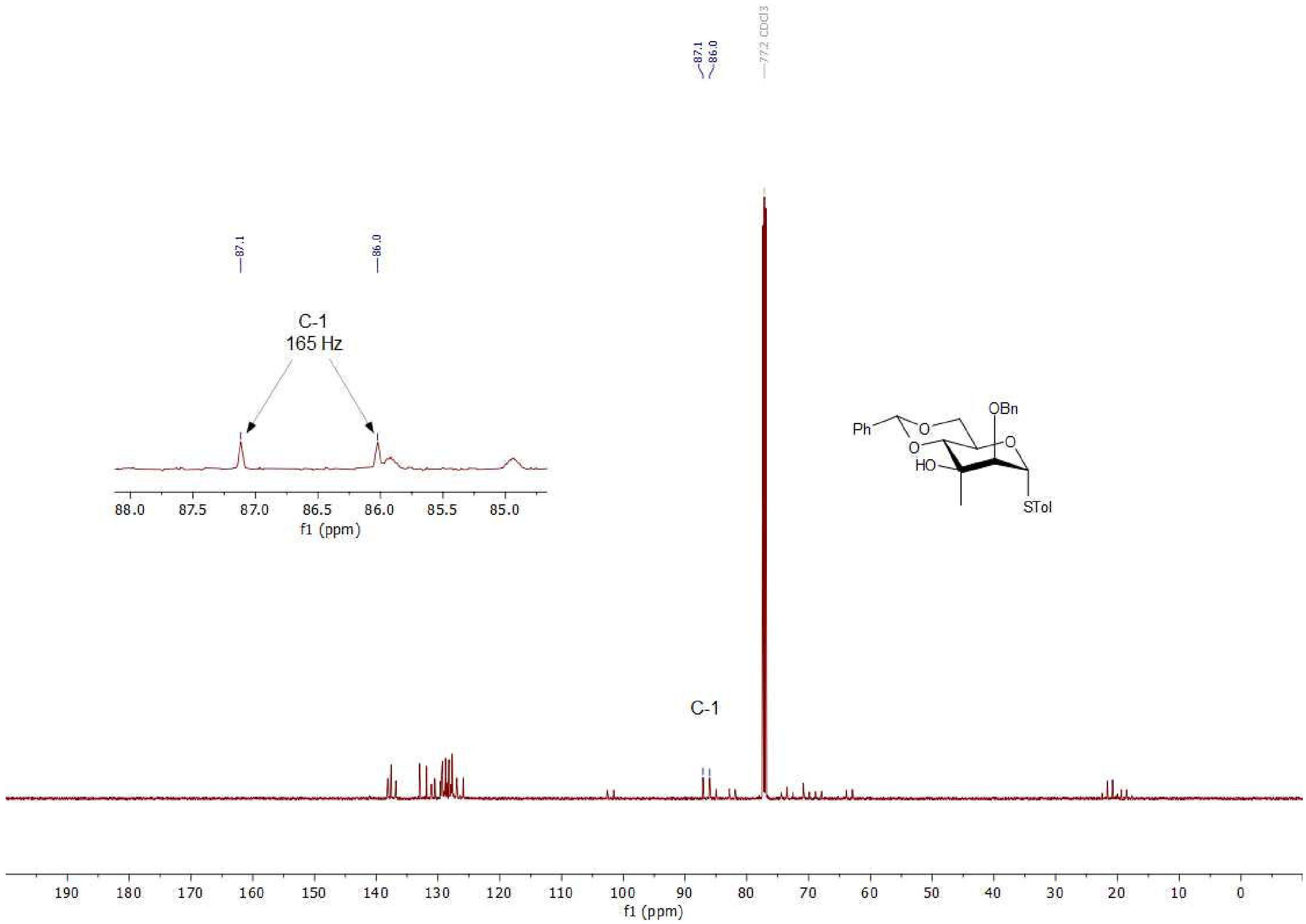

Figure S111. HSQC NMR (600 MHz, $CDCl_3$) spectrum of *p*-methylphenyl 2-*O*-benzyl-4,6-*O*-benzylidene-3-*C*-methyl-thio-α-D-mannopyranoside **44**:

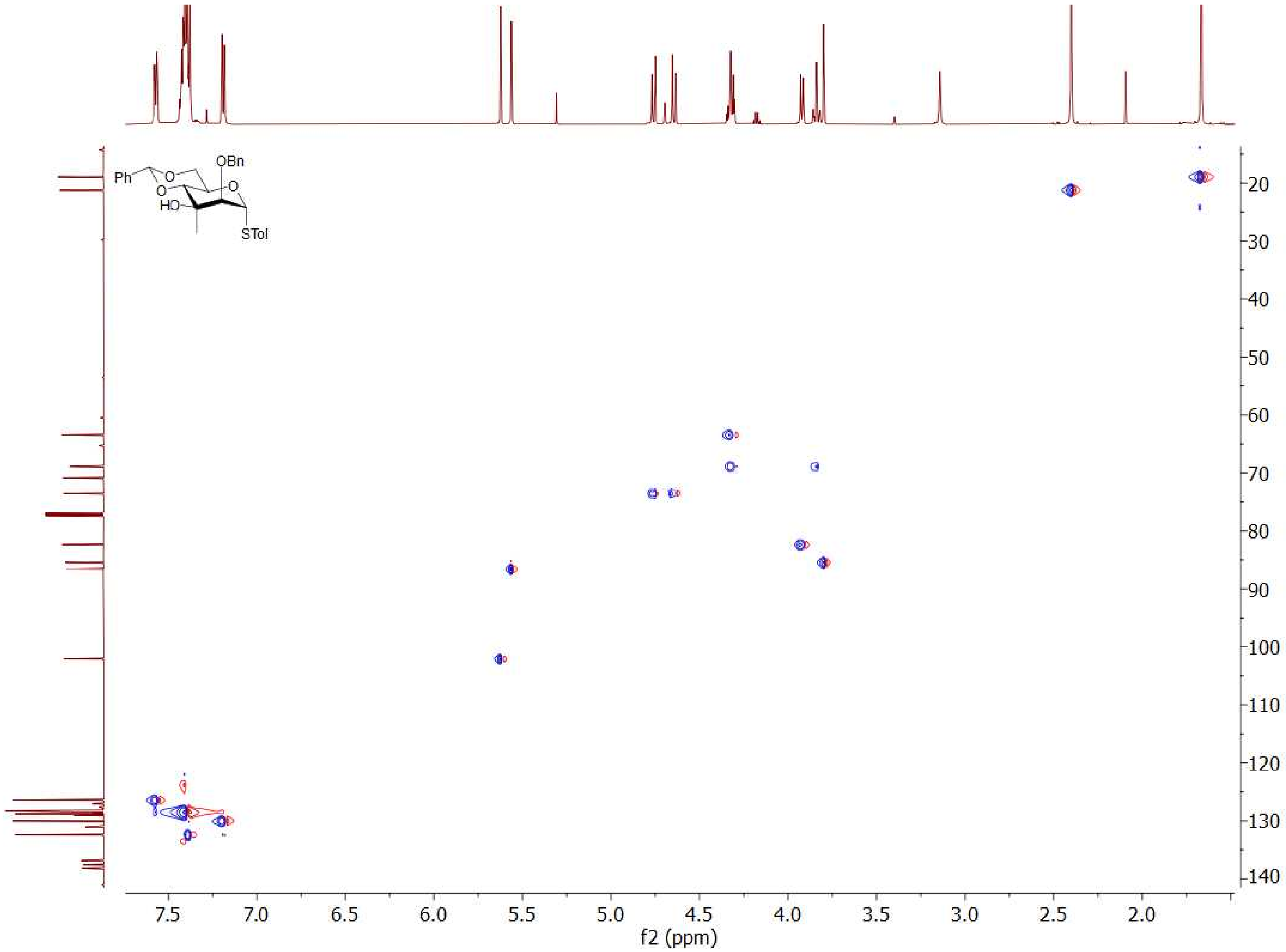

Figure S112. HMBC NMR (600 MHz, $CDCl_3$) spectrum of *p*-methylphenyl 2-*O*-benzyl-4,6-*O*-benzylidene-3-*C*-methyl-thio-α-D-mannopyranoside **44**:

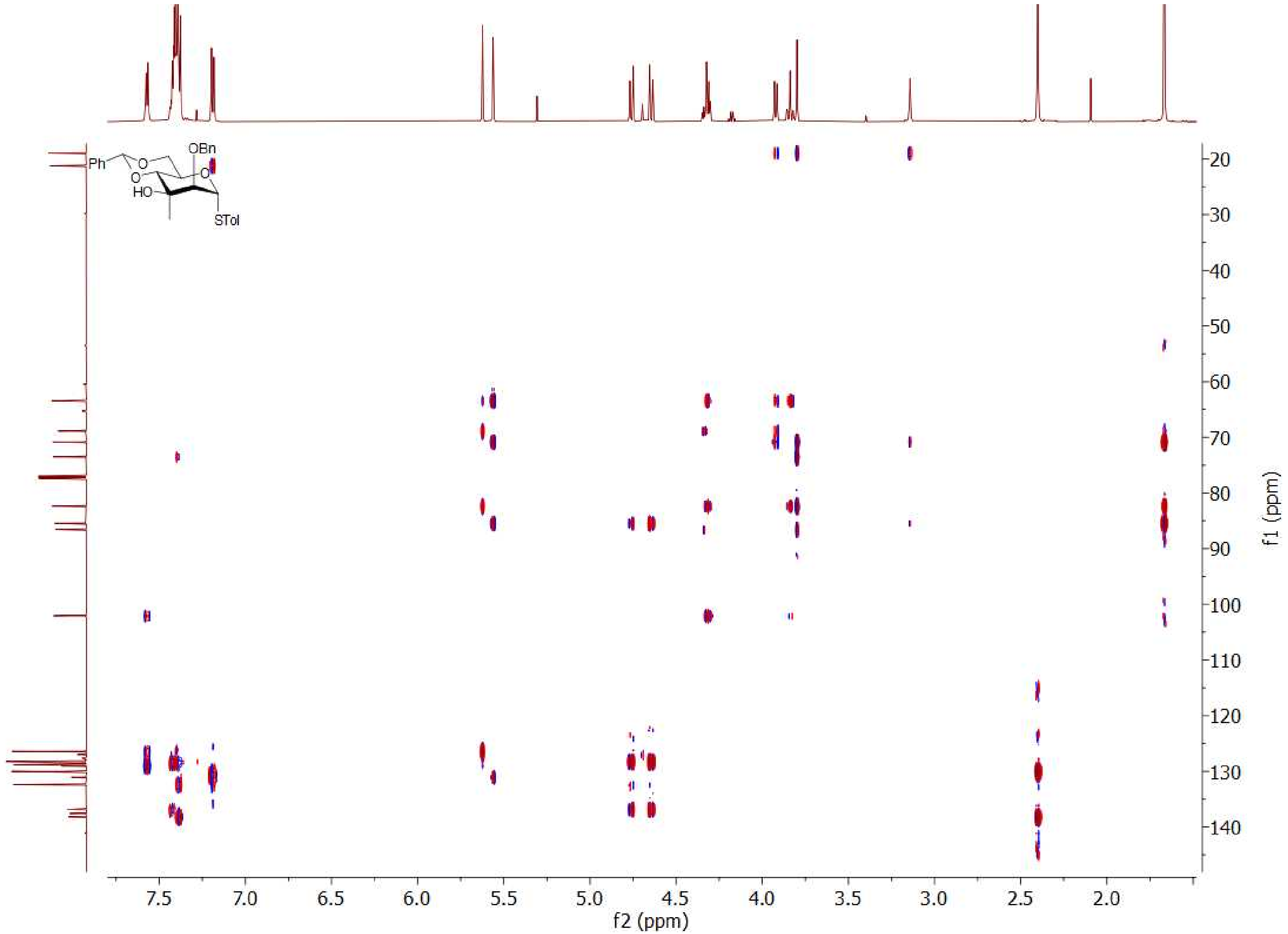

Figure S113. ^{1}H NMR (600 MHz, $CDCl_3$) comparative spectra of *p*-methylphenyl 2-*O*-benzyl-4,6-*O*-benzylidene-3-*C*-methyl-thio-β-D-mannopyranoside **37** and *p*-methylphenyl 2-*O*-benzyl-4,6-*O*-benzylidene-3-*C*-methyl-thio-α-D-mannopyranoside **44**:

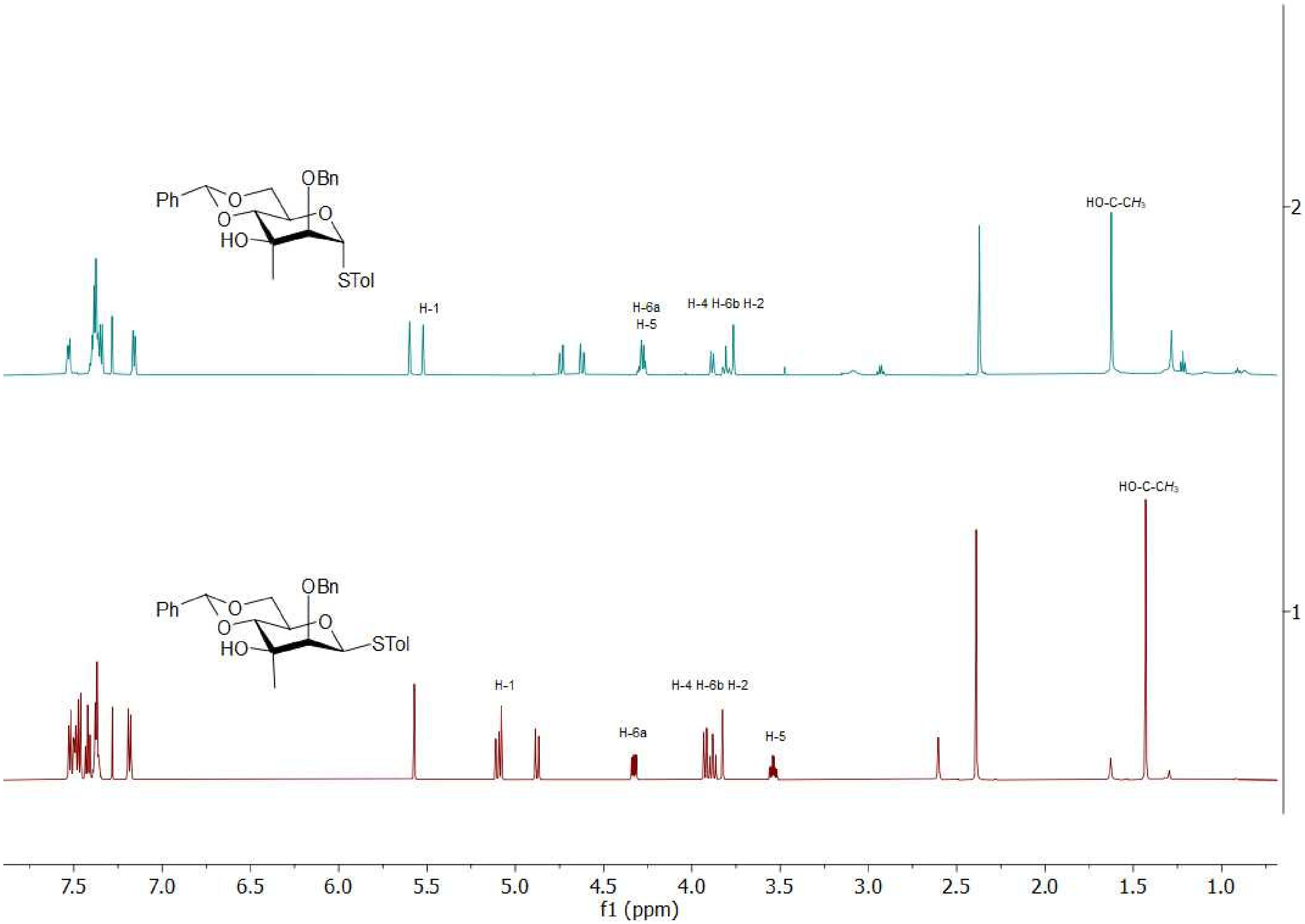

Figure S114. $^{13}C\{^1H\}$ NMR (151 MHz, $CDCl_3$) comparative spectra of *p*-methylphenyl 2-*O*-benzyl-4,6-*O*-benzylidene-3-*C*-methyl-thio-β-D-mannopyranoside **37** and *p*-methylphenyl 2-*O*-benzyl-4,6-*O*-benzylidene-3-*C*-methyl-thio-α-D-mannopyranoside **44**:

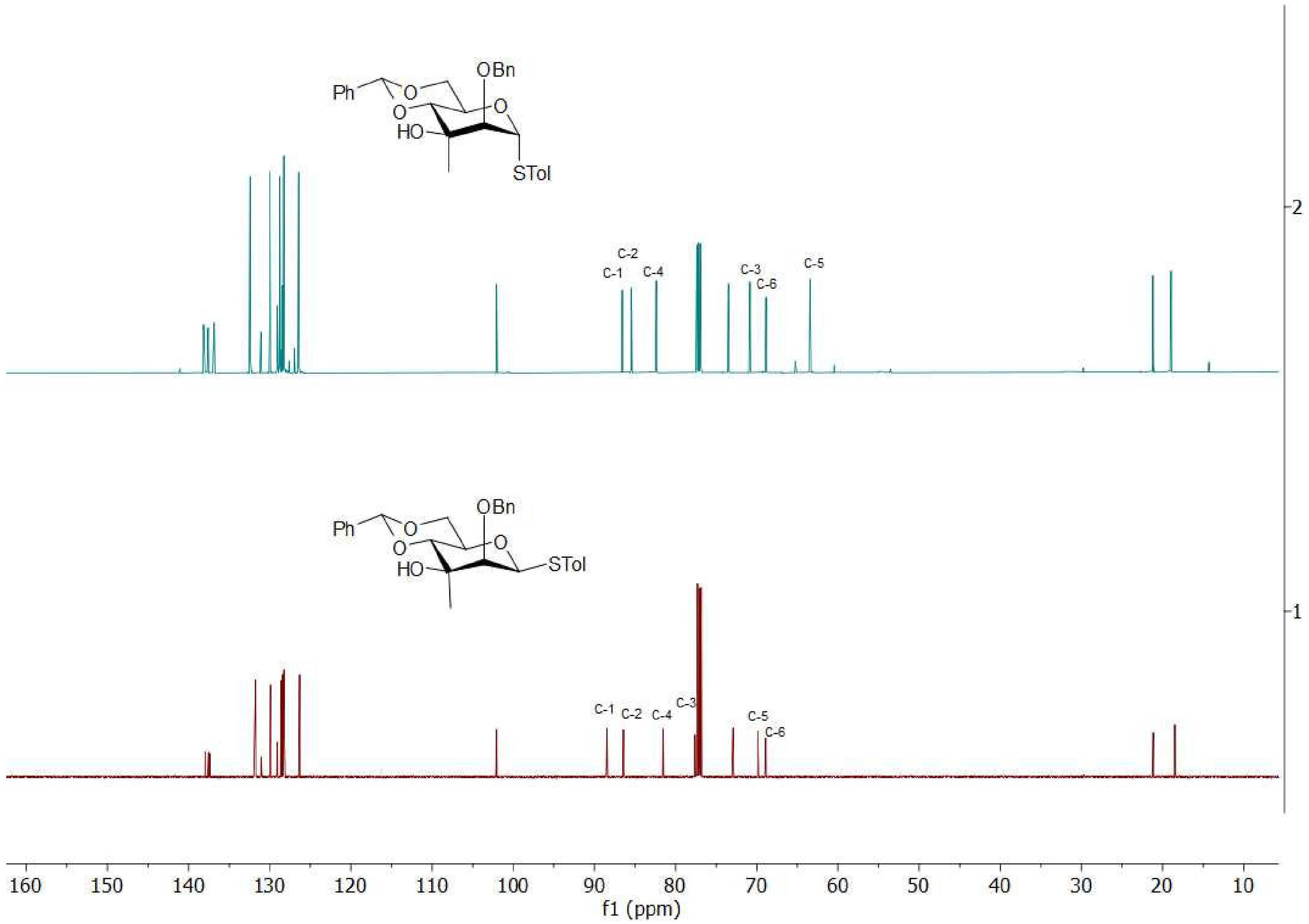

Figure S115. ^{1}H NMR (600 MHz, $CDCl_3$) spectrum of *p*-methylphenyl 3-*O*-(benzoyl-α-^{13}C)-2-*O*-benzyl-4,6-*O*-benzylidene-3-*C*-methyl-thio-α-D-mannopyranoside **^{13}C-45**:

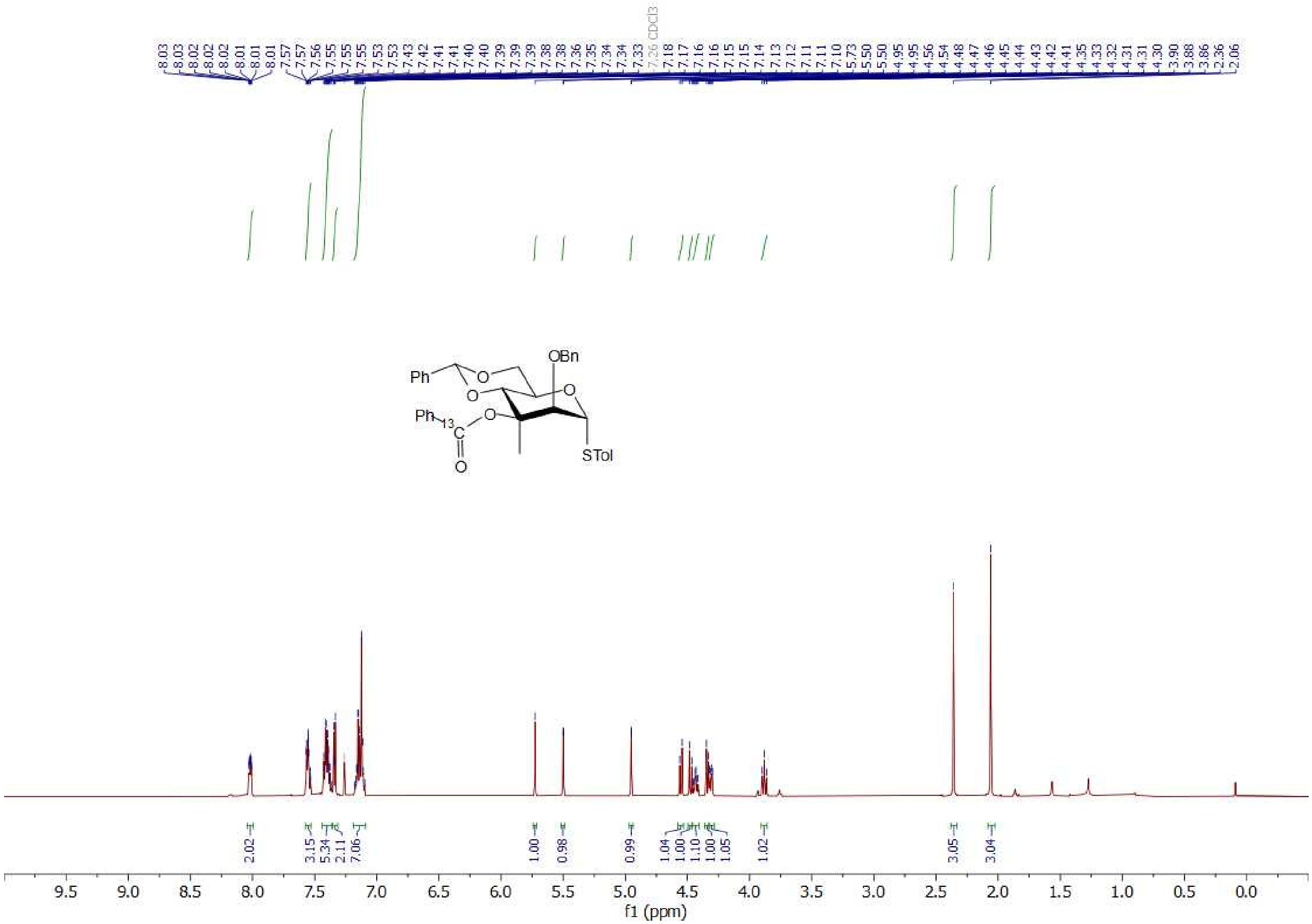

Figure S116. COSY NMR (600 MHz, $CDCl_3$) spectrum of *p*-methylphenyl 3-*O*-(benzoyl-α-^{13}C)-2-*O*-benzyl-4,6-*O*-benzylidene-3-*C*-methyl-thio-α-D-mannopyranoside **^{13}C-45**:

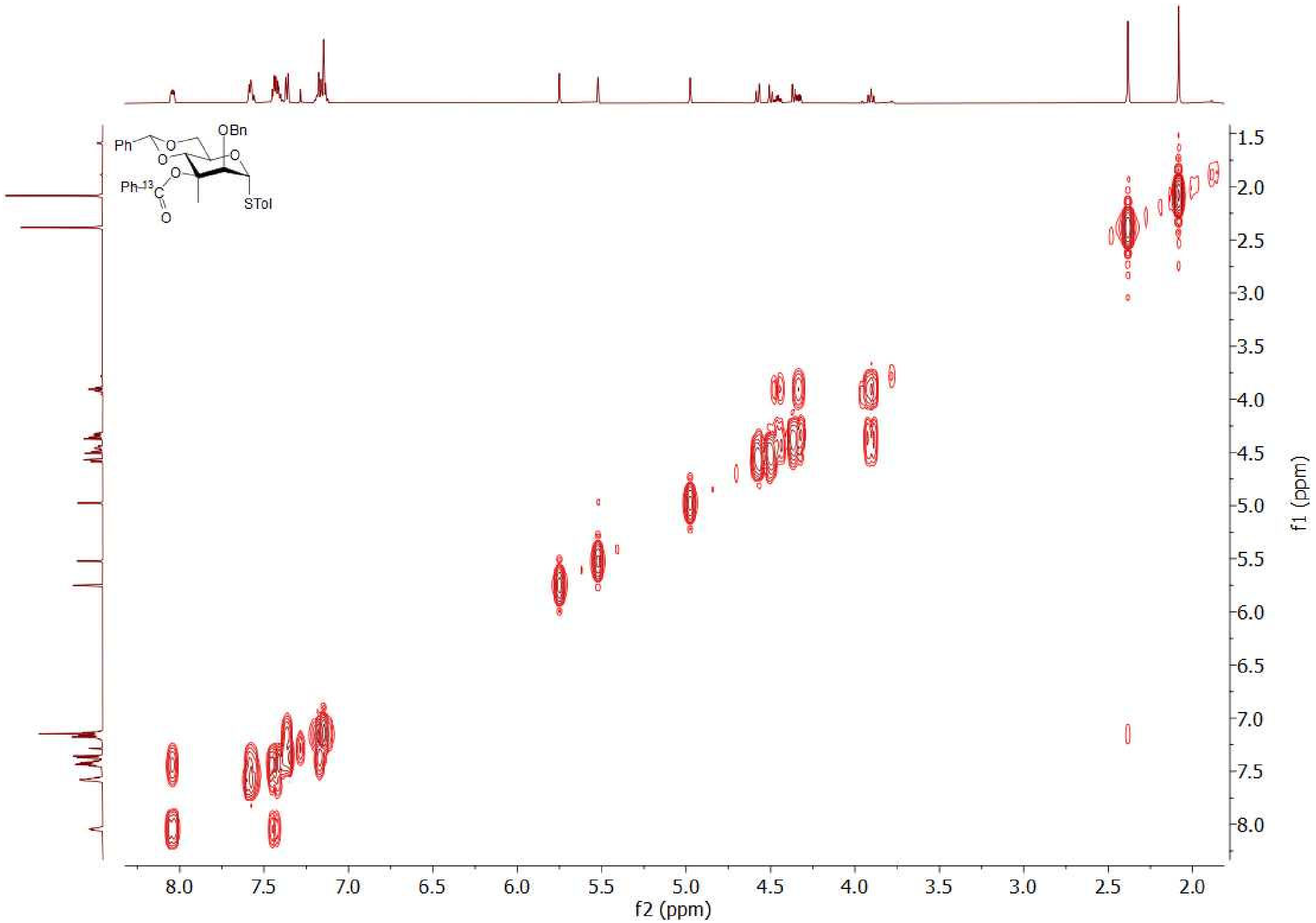

Figure S117. $^{13}C\{^{1}H\}$ NMR (151 MHz, $CDCl_3$) spectrum of *p*-methylphenyl 3-*O*-(benzoyl-α-^{13}C)-2-*O*-benzyl-4,6-*O*-benzylidene-3-*C*-methyl-thio-α-D-mannopyranoside **^{13}C-45**:

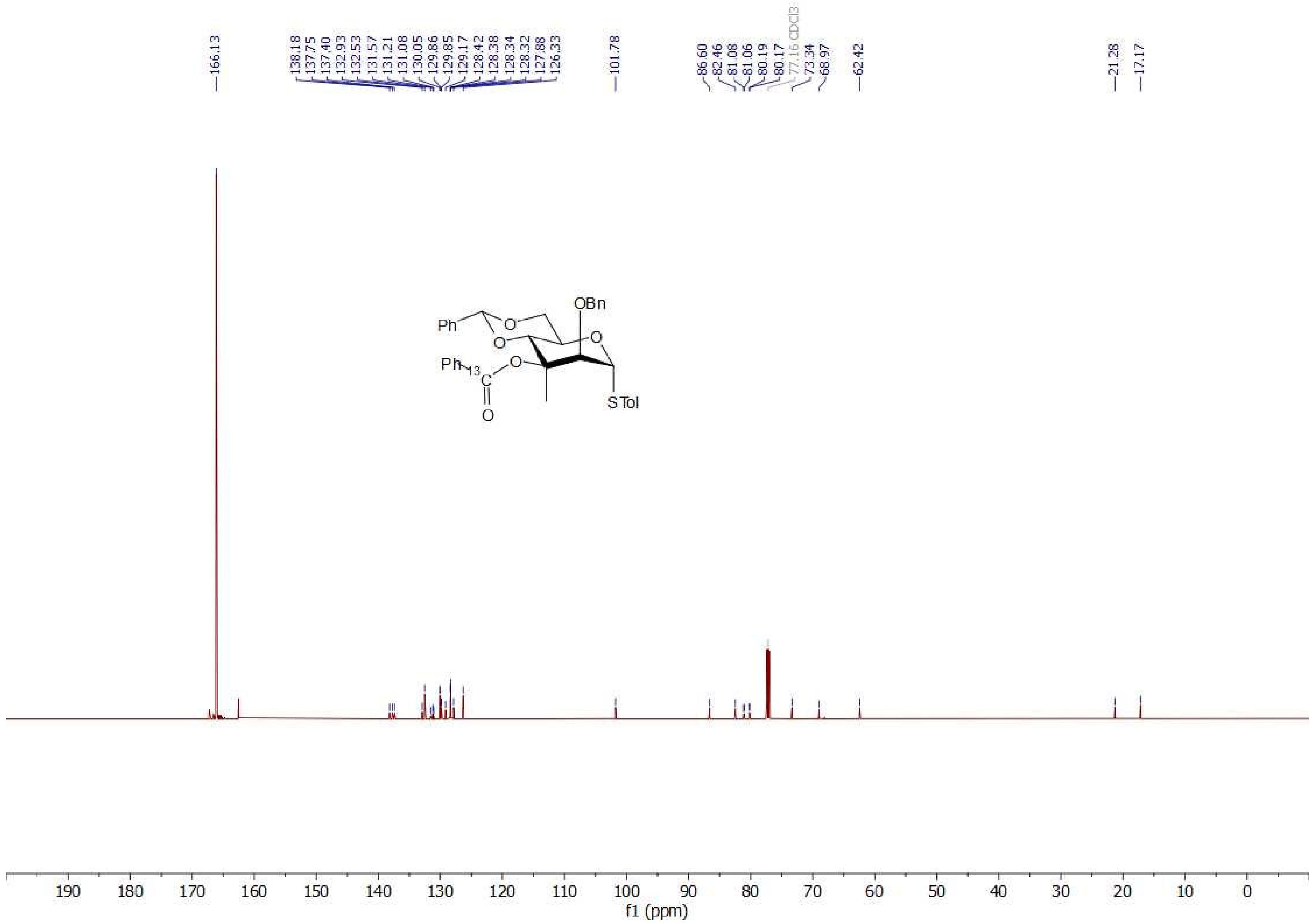

Figure S118. $^{13}C\{^1H\}$ DEPT NMR (151 MHz, $CDCl_3$) spectrum of *p*-methylphenyl 3-*O*-(benzoyl-α-^{13}C)-2-*O*-benzyl-4,6-*O*-benzylidene-3-*C*-methyl-thio-α-D-mannopyranoside **^{13}C-45**:

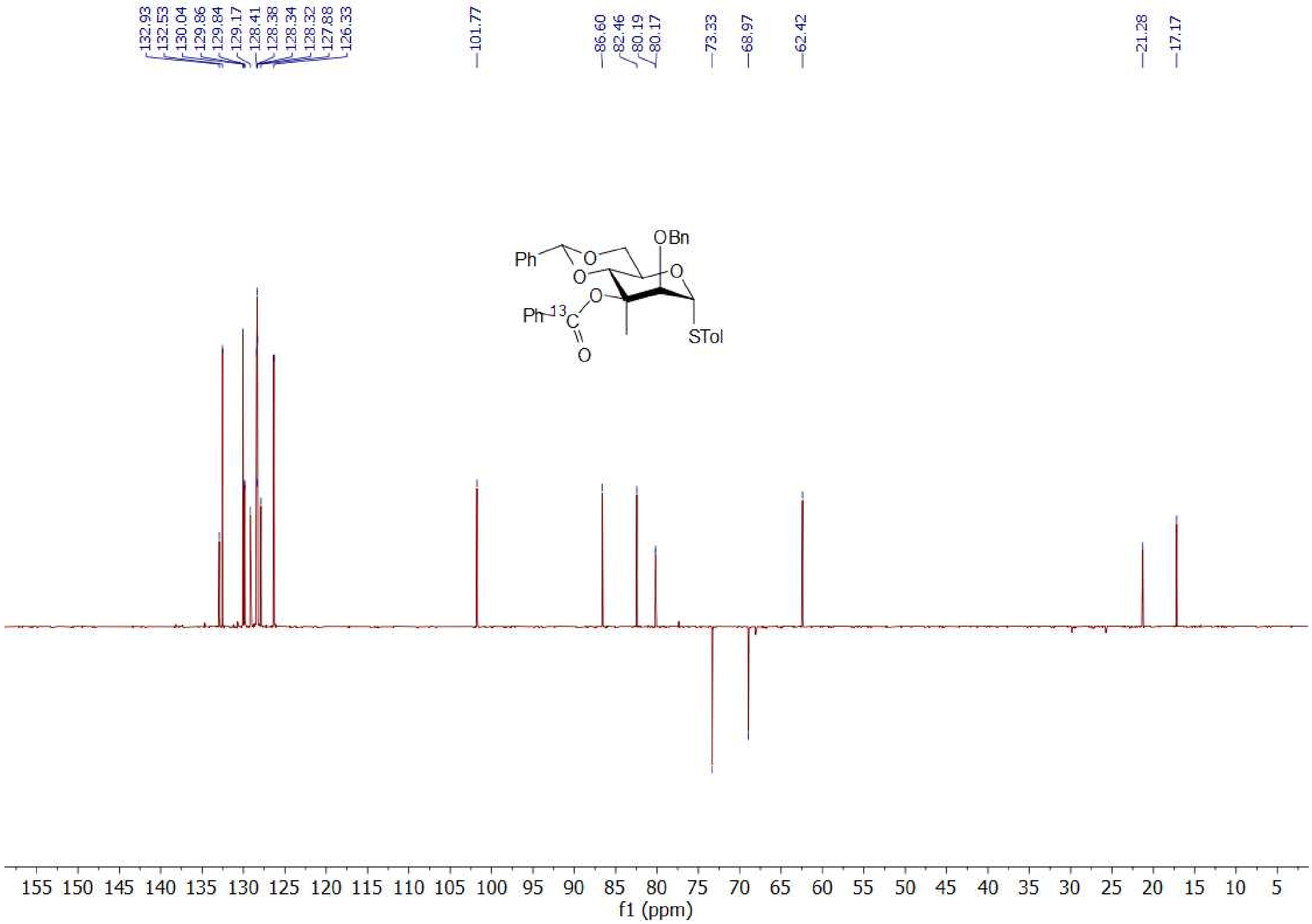

Figure S119. HSQC NMR (600 MHz, $CDCl_3$) spectrum of *p*-methylphenyl 3-*O*-(benzoyl-α-^{13}C)-2-*O*-benzyl-4,6-*O*-benzylidene-3-*C*-methyl-thio-α-D-mannopyranoside **^{13}C-45**:

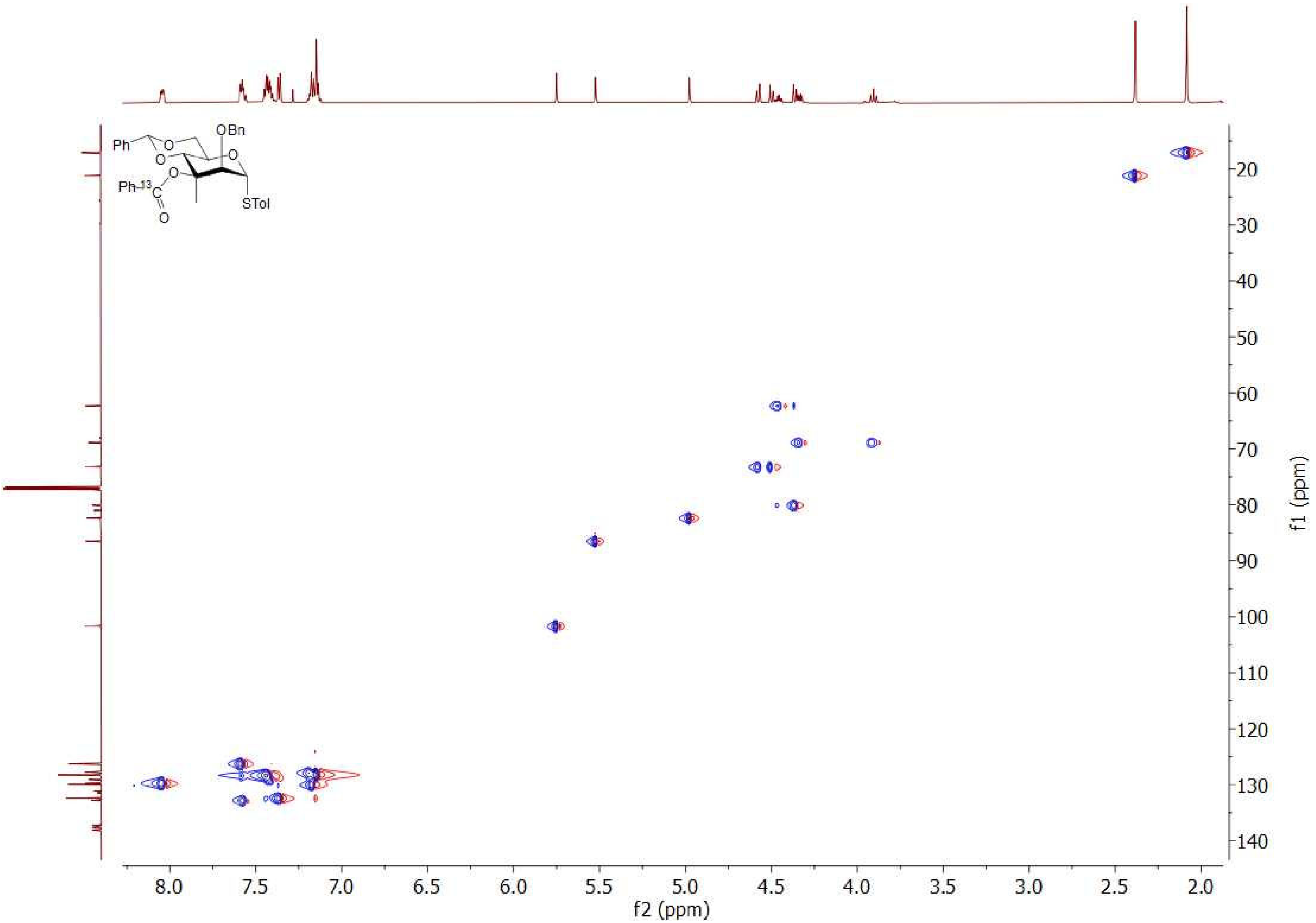

Figure S120. HMBC NMR (600 MHz, $CDCl_3$) spectrum of *p*-methylphenyl 3-*O*-(benzoyl-α-^{13}C)-2-*O*-benzyl-4,6-*O*-benzylidene-3-*C*-methyl-thio-α-D-mannopyranoside **^{13}C-45**:

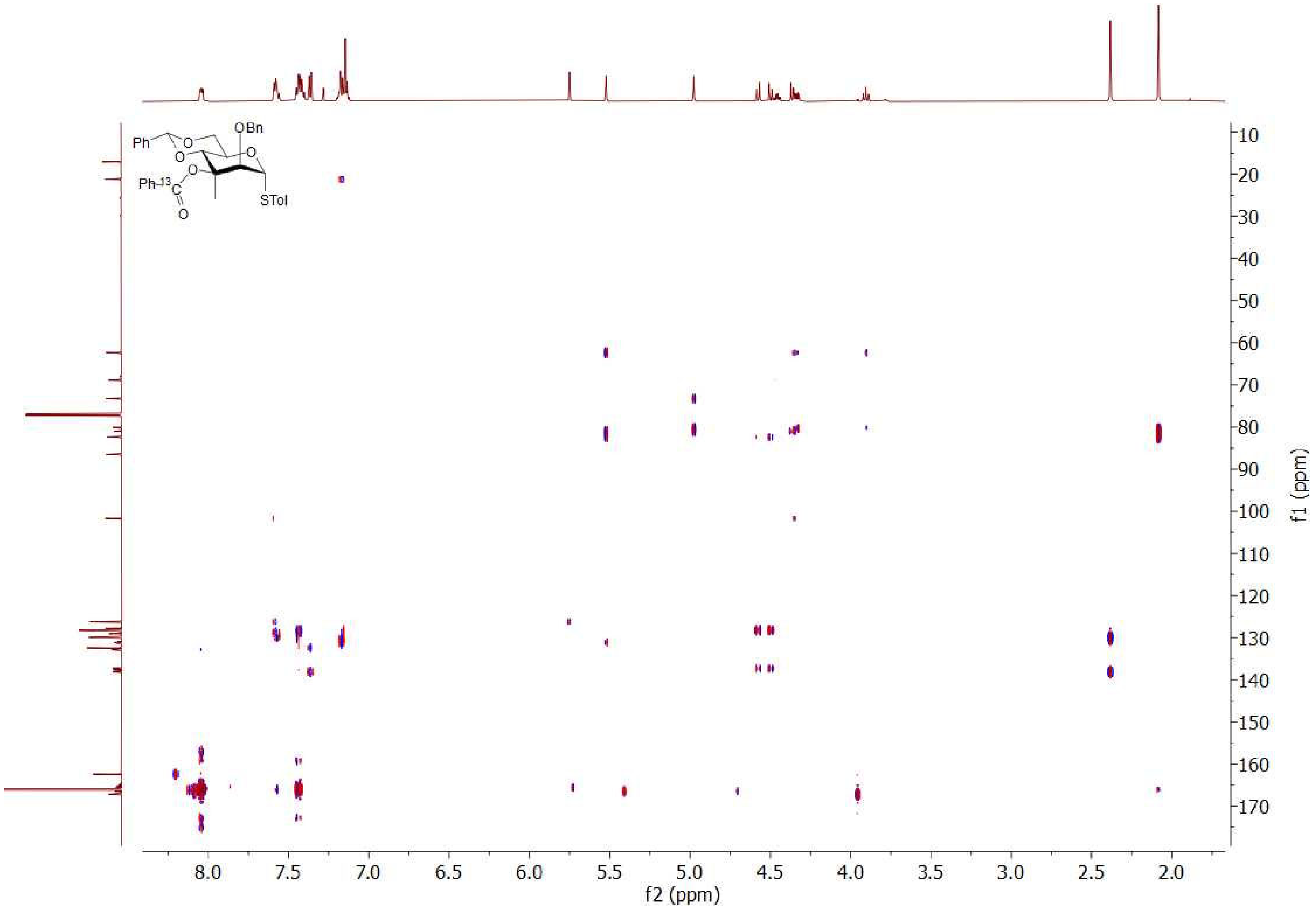

Figure S121. ^{1}H NMR (600 MHz, CDCl$_3$) spectrum of *p*-methylphenyl 3-*O*-(benzoyl-α-^{13}C)-2-*O*-benzyl-4,6-*O*-benzylidene-3-*C*-methyl-thio-α-D-mannopyranoside *S*-oxide **^{13}C-46**:

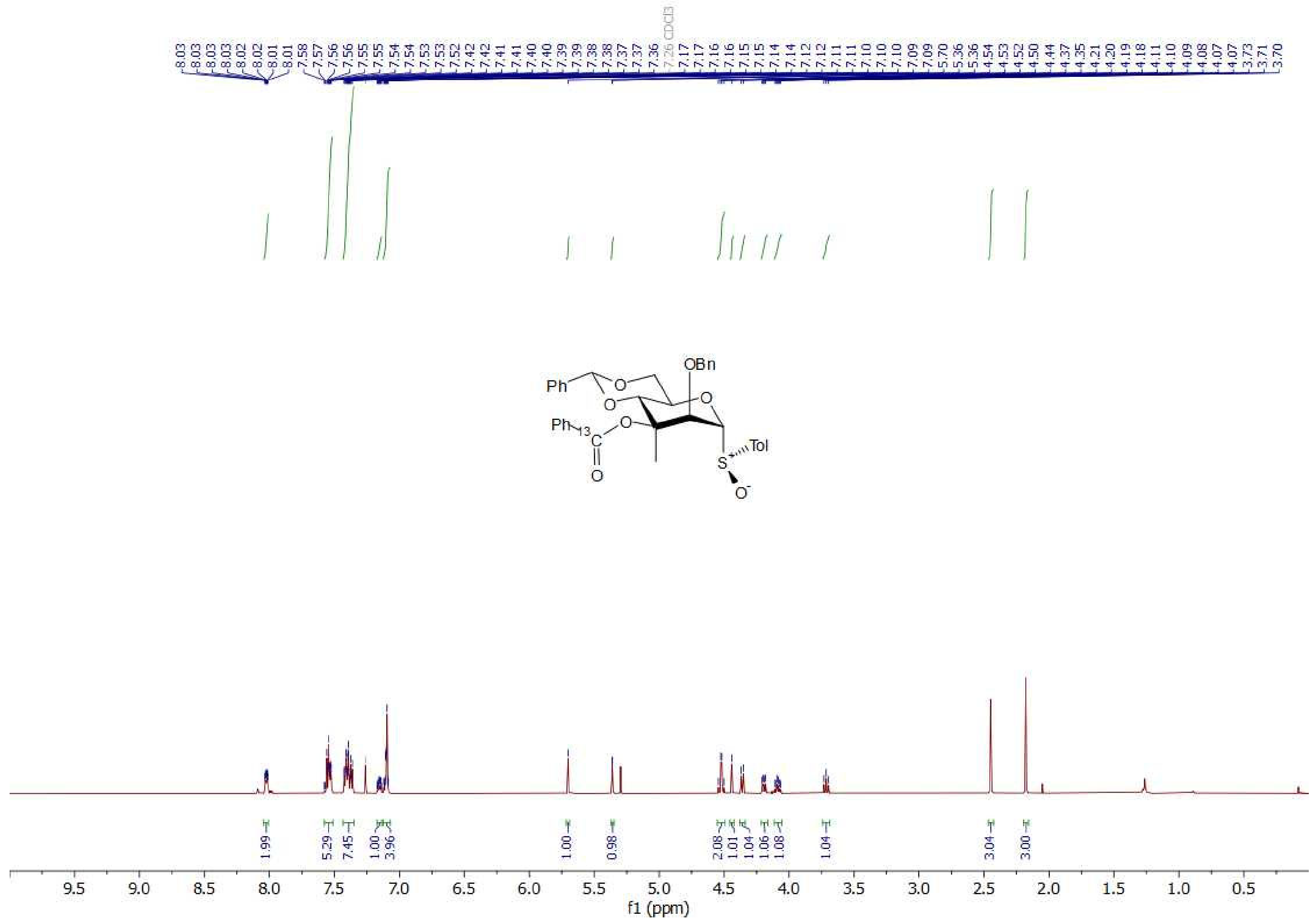

Figure S122. COSY NMR (600 MHz, $CDCl_3$) spectrum of *p*-methylphenyl 3-*O*-(benzoyl-α-^{13}C)-2-*O*-benzyl-4,6-*O*-benzylidene-3-*C*-methyl-thio-α-D-mannopyranoside *S*-oxide **^{13}C-46:**

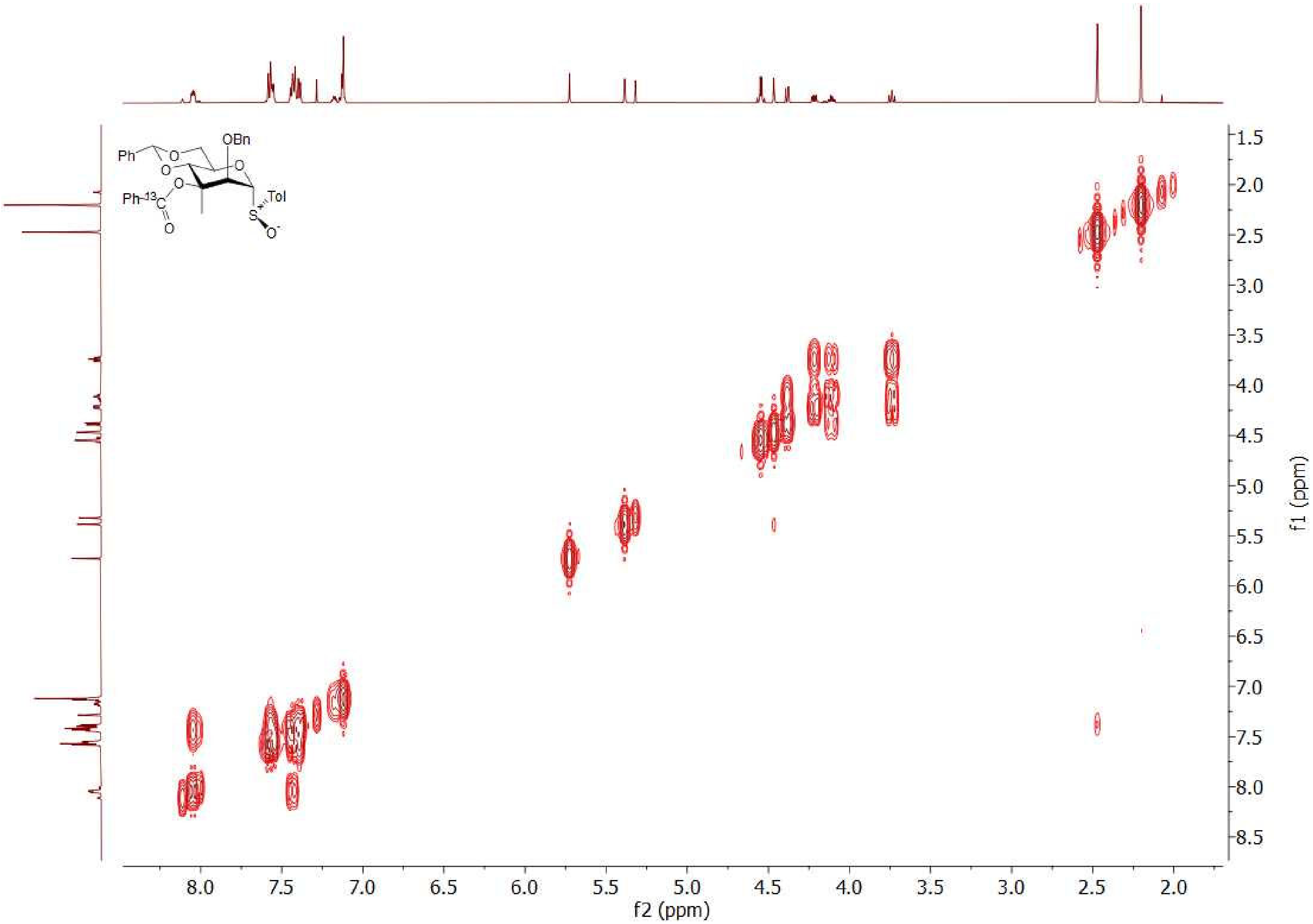

Figure S123. $^{13}C\{^1H\}$ NMR (151 MHz, $CDCl_3$) spectrum of *p*-methylphenyl 3-*O*-(benzoyl-α-^{13}C)-2-*O*-benzyl-4,6-*O*-benzylidene-3-*C*-methyl-thio-α-D-mannopyranoside *S*-oxide **^{13}C-46**:

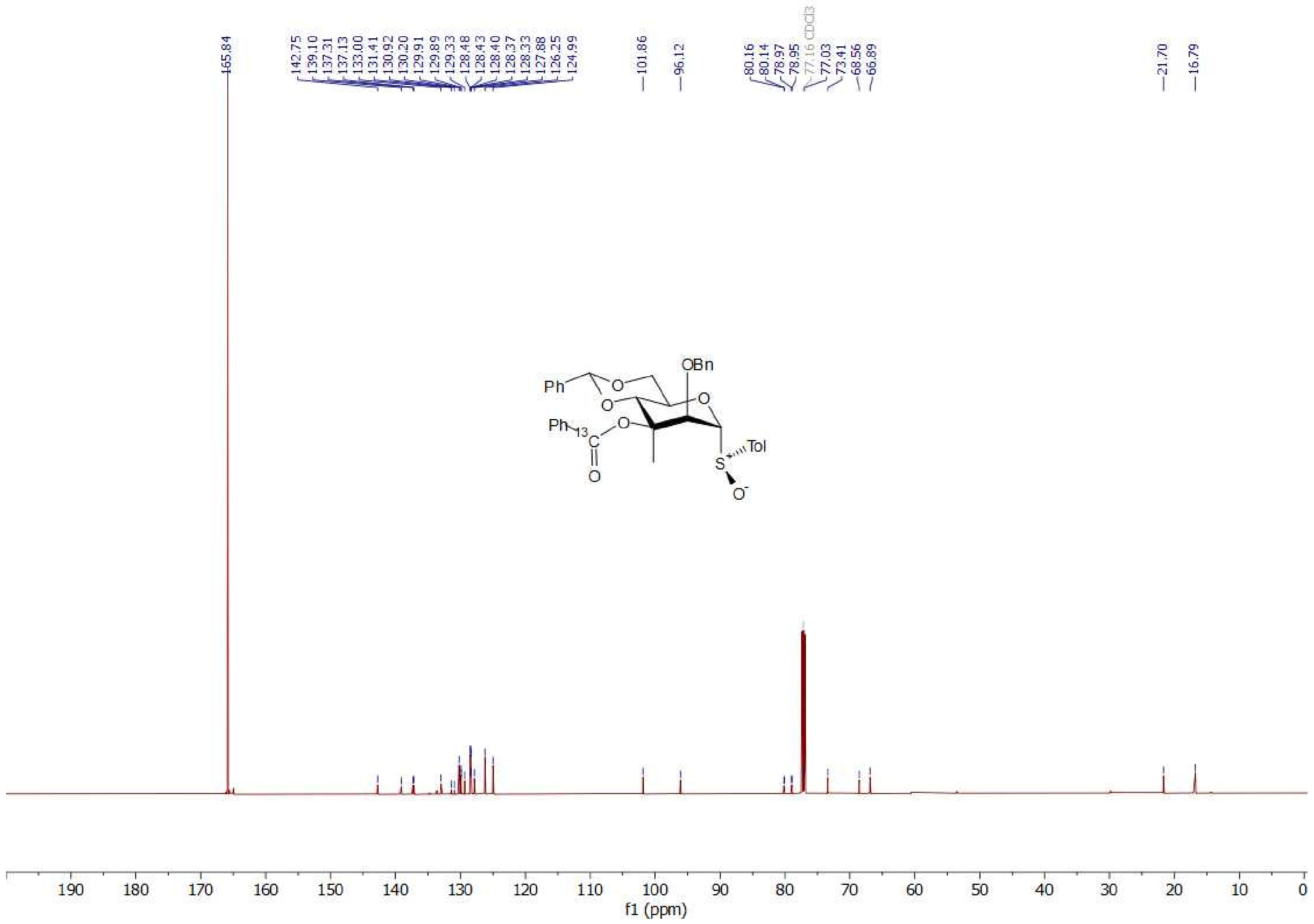

Figure S124. $^{13}C\{^{1}H\}$ DEPT NMR (151 MHz, $CDCl_3$) spectrum of *p*-methylphenyl 3-*O*-(benzoyl-α-^{13}C)-2-*O*-benzyl-4,6-*O*-benzylidene-3-*C*-methyl-thio-α-D-mannopyranoside *S*-oxide **^{13}C-46**:

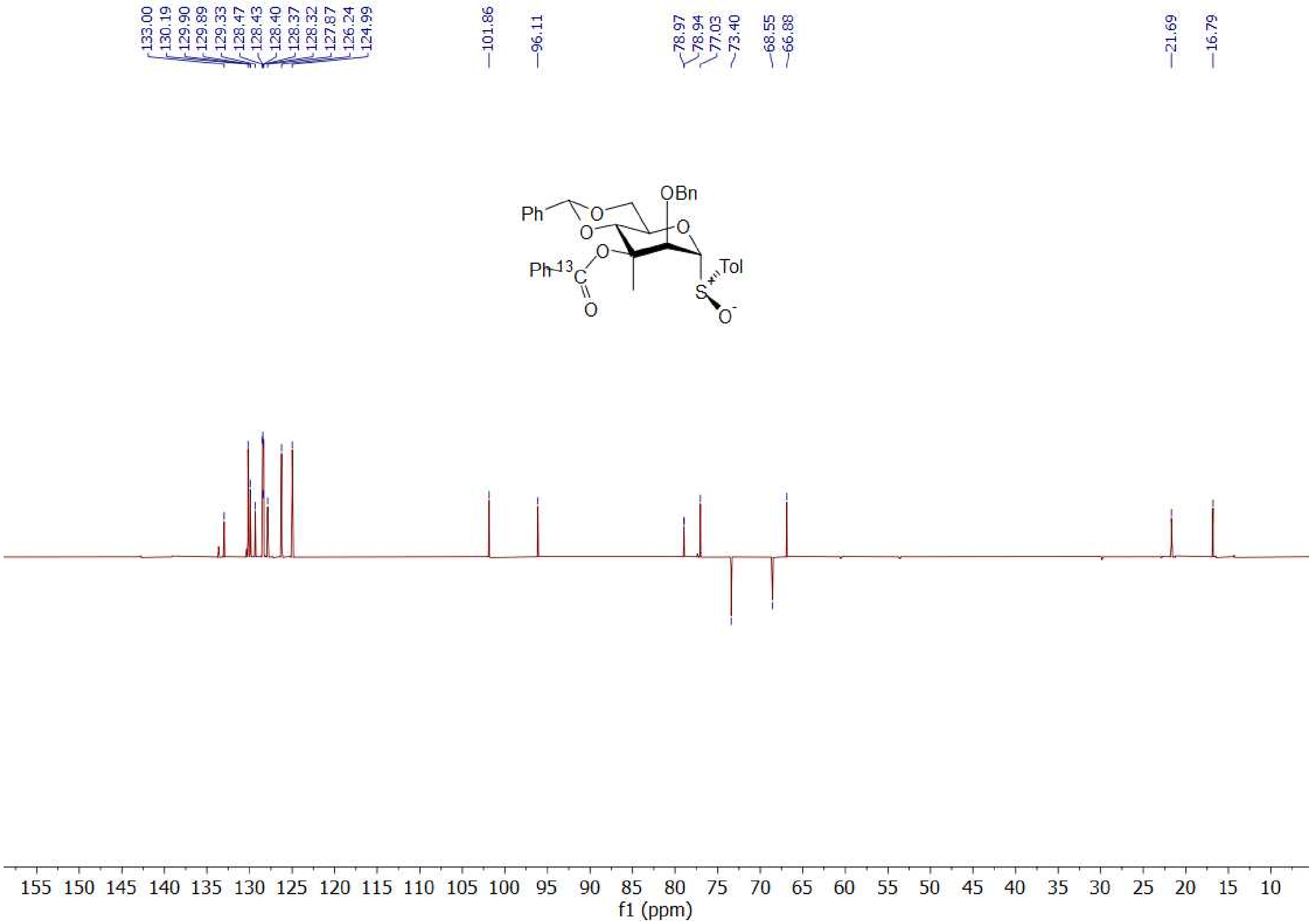

Figure S125. HSQC NMR (600 MHz, $CDCl_3$) spectrum of *p*-methylphenyl 3-*O*-(benzoyl-α-^{13}C)-2-*O*-benzyl-4,6-*O*-benzylidene-3-*C*-methyl-thio-α-D-mannopyranoside *S*-oxide **^{13}C-46**:

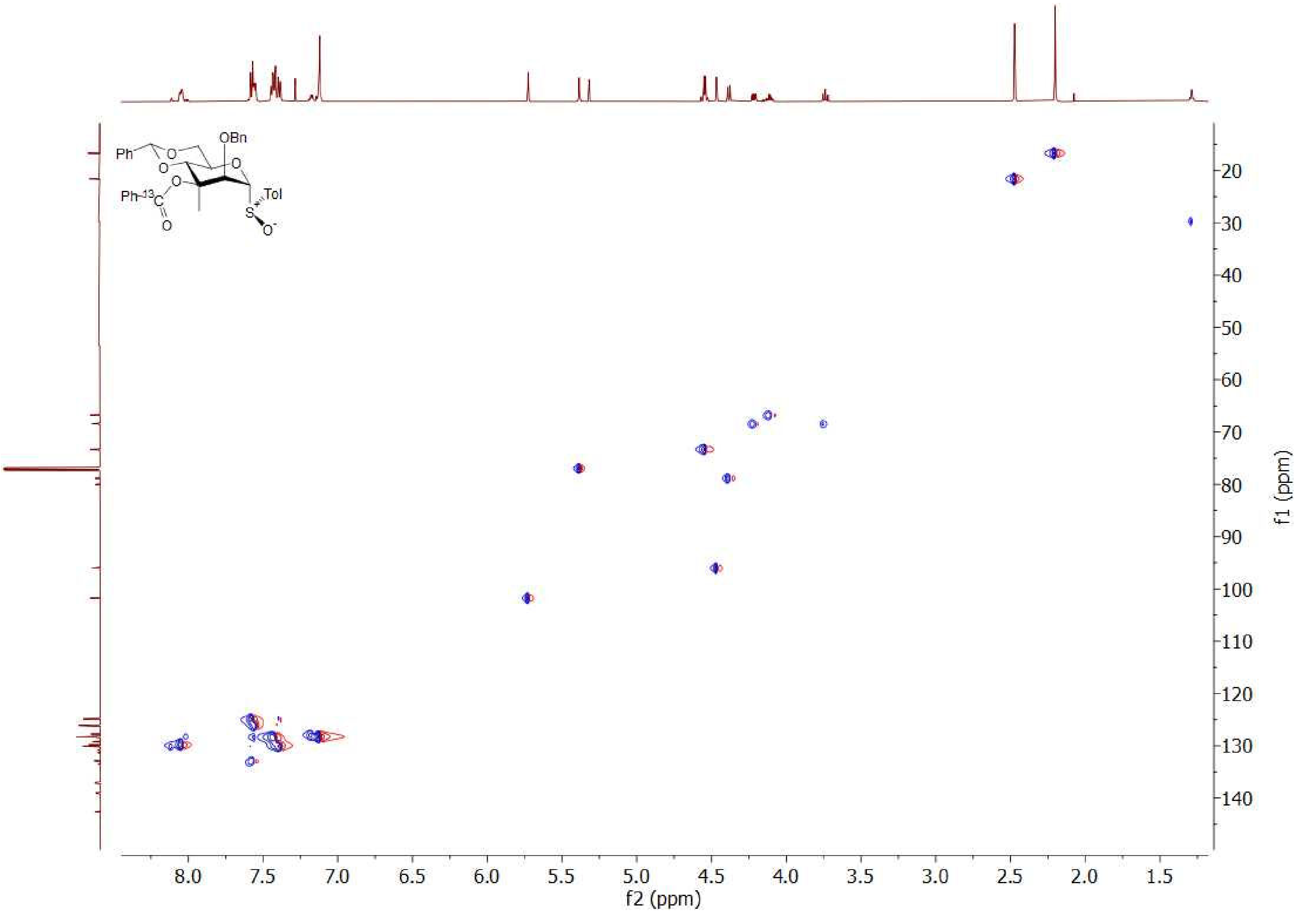

Figure S126. HMBC NMR (600 MHz, CDCl₃) spectrum of *p*-methylphenyl 3-*O*-(benzoyl-α-^{13}C)-2-*O*-benzyl-4,6-*O*-benzylidene-3-*C*-methyl-thio-α-D-mannopyranoside *S*-oxide **^{13}C-46**:

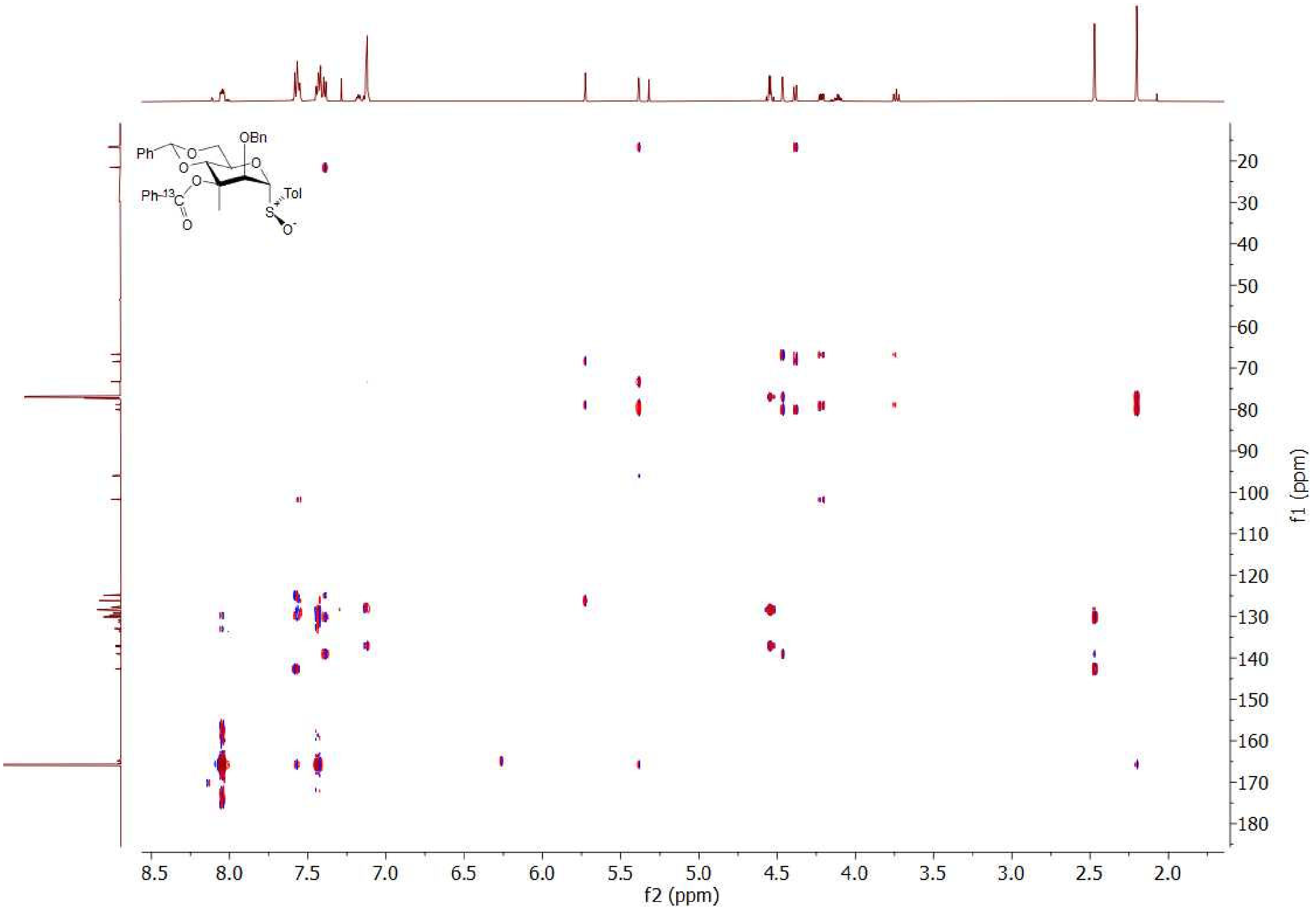

Figure S127. ^{1}H NMR (600 MHz, CDCl$_3$) spectrum of *p*-methylphenyl 3-*O*-benzoyl-2-*O*-benzyl-4,6-*O*-benzylidene-thio-α-D-mannopyranoside **48**:

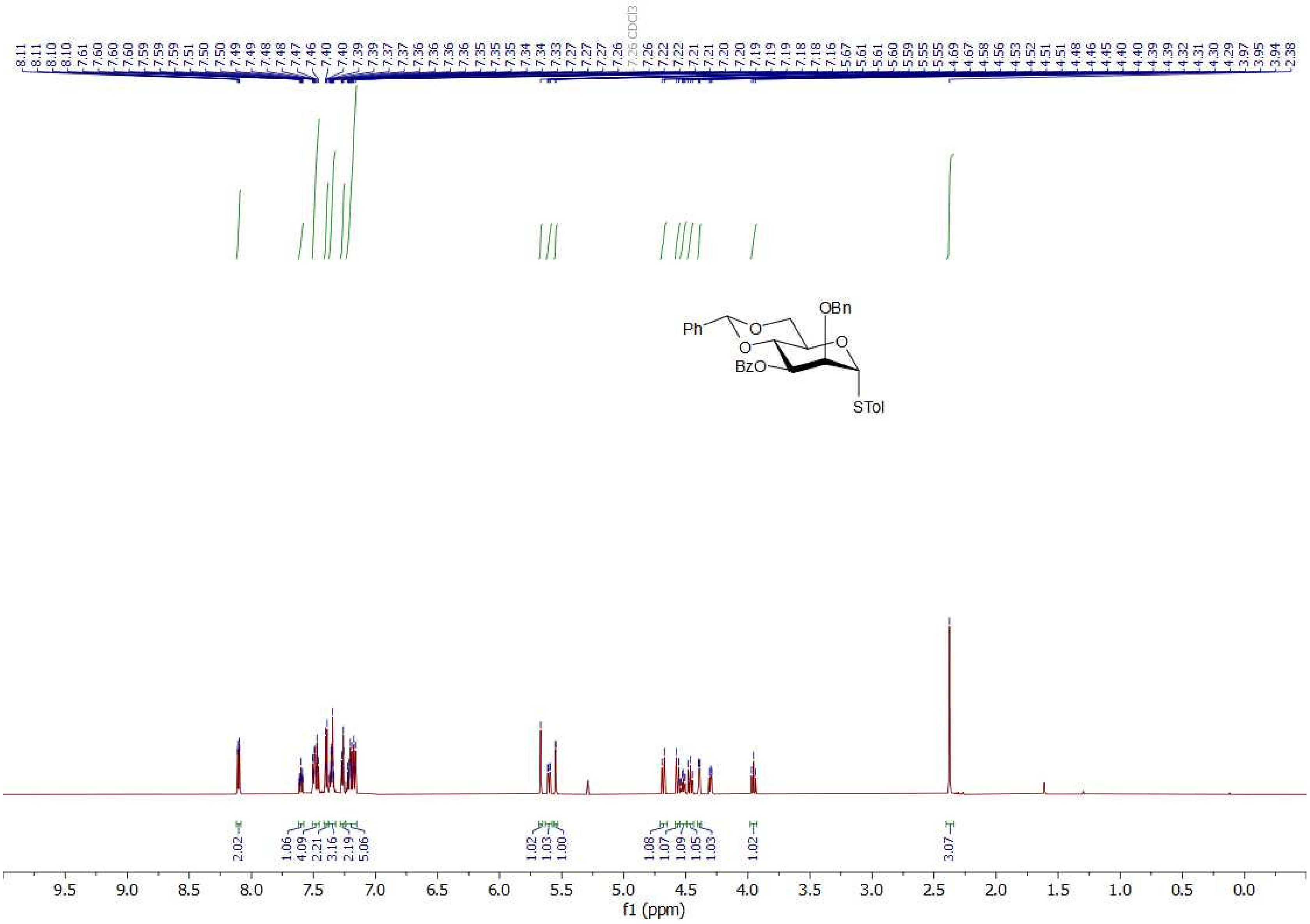

Figure S128. COSY NMR (600 MHz, $CDCl_3$) spectrum of *p*-methylphenyl 3-*O*-benzoyl-2-*O*-benzyl-4,6-*O*-benzylidene-thio-α-D-mannopyranoside **48**:

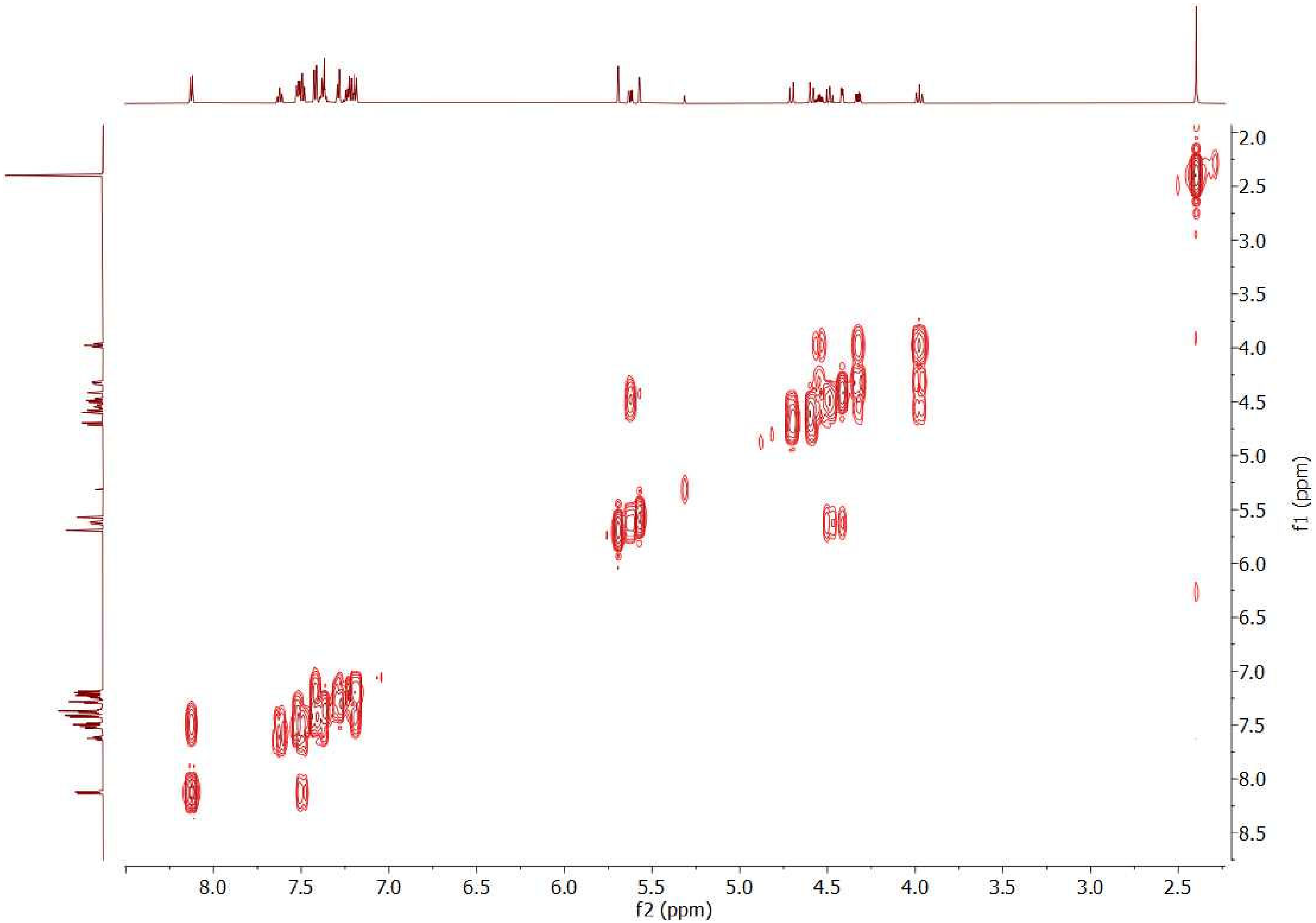

Figure S129. $^{13}C\{^1H\}$ NMR (151 MHz, $CDCl_3$) spectrum of *p*-methylphenyl 3-*O*-benzoyl-2-*O*-benzyl-4,6-*O*-benzylidene-thio-α-D-mannopyranoside **48**:

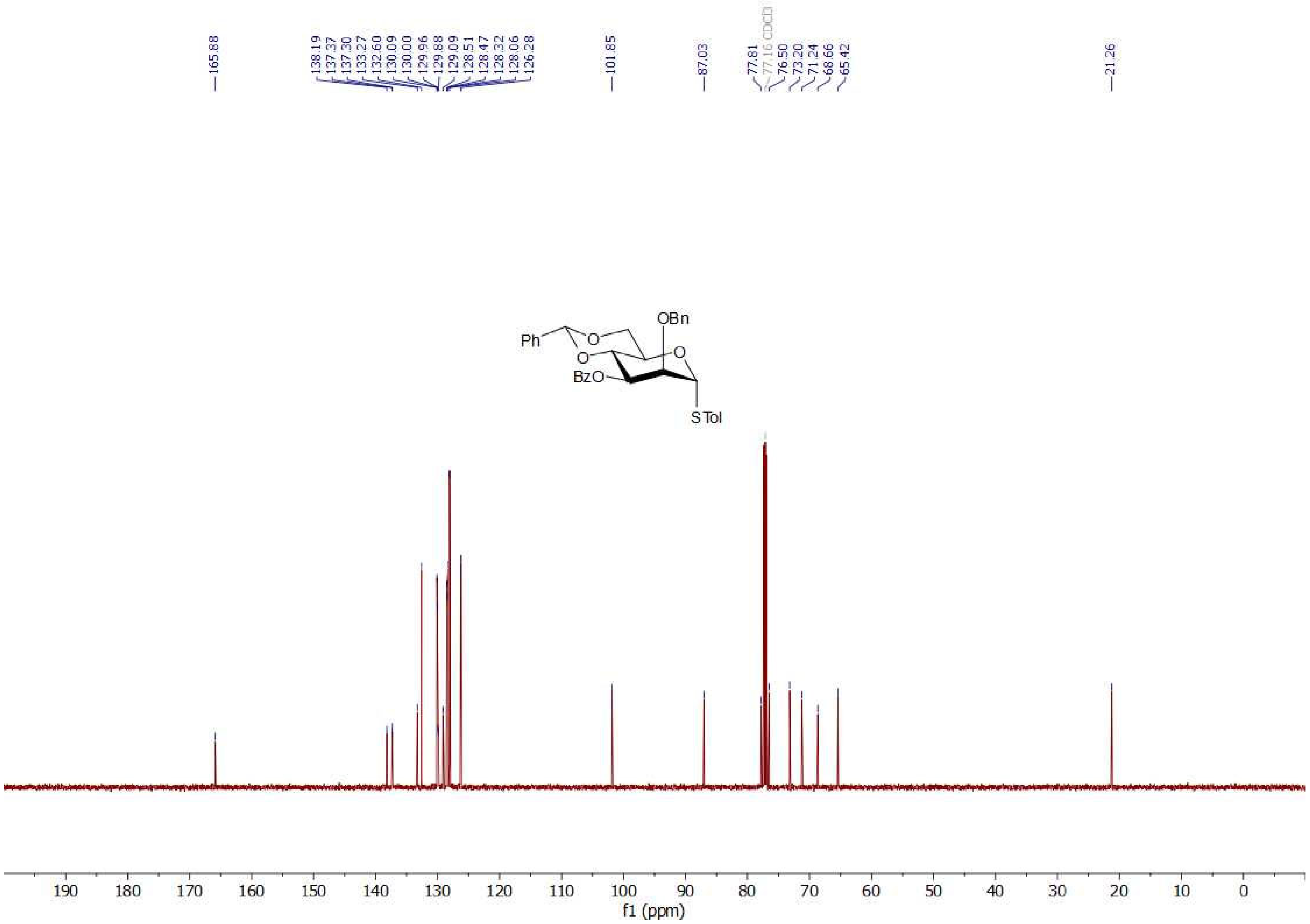

Figure S130. $^{13}C\{^1H\}$ DEPT NMR (151 MHz, $CDCl_3$) spectrum of *p*-methylphenyl 3-*O*-benzoyl-2-*O*-benzyl-4,6-*O*-benzylidene-thio-α-D-mannopyranoside **48**:

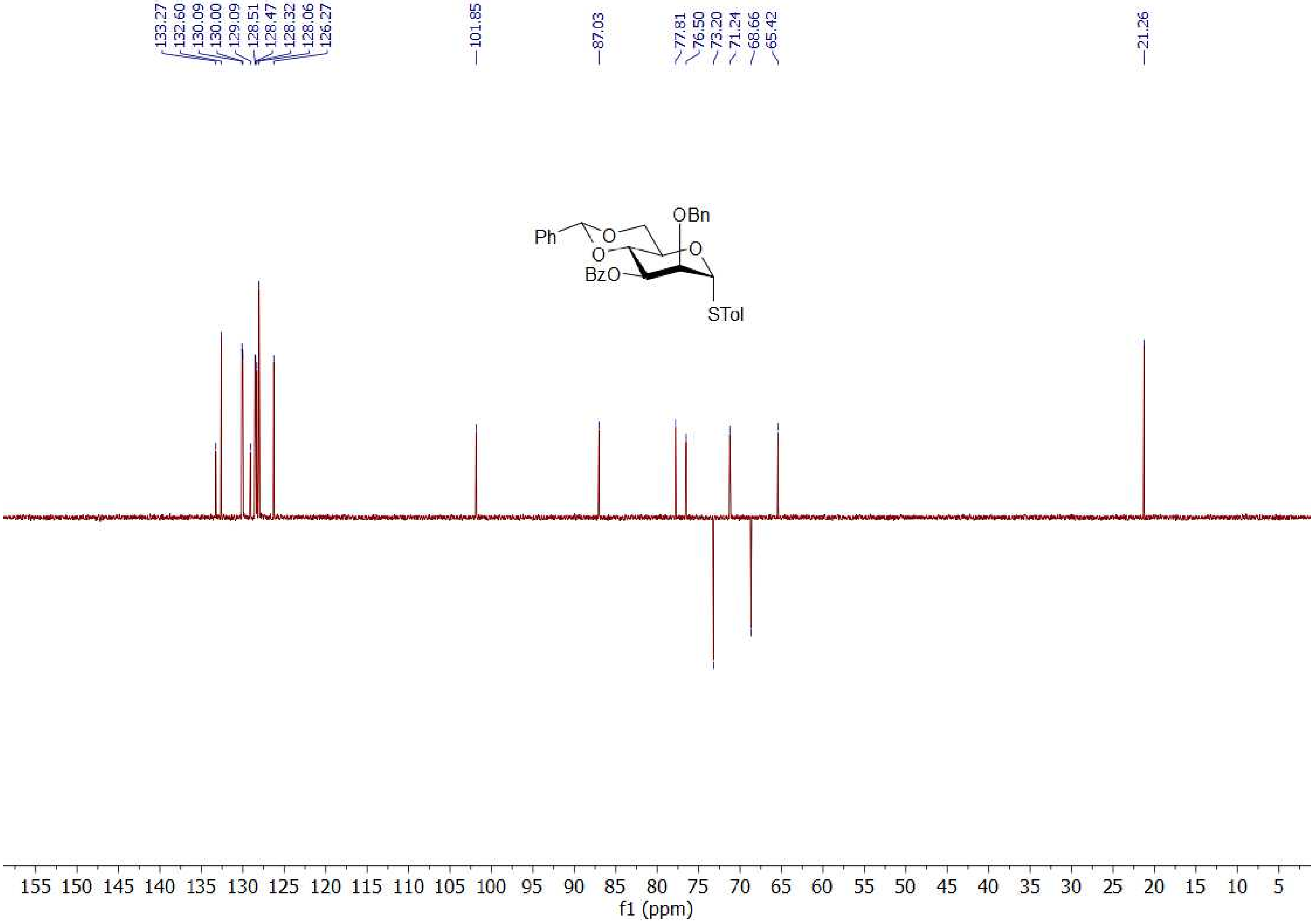

Figure S131. HSQC NMR (600 MHz, $CDCl_3$) spectrum of *p*-methylphenyl 3-*O*-benzoyl-2-*O*-benzyl-4,6-*O*-benzylidene-thio-α-D-mannopyranoside **48**:

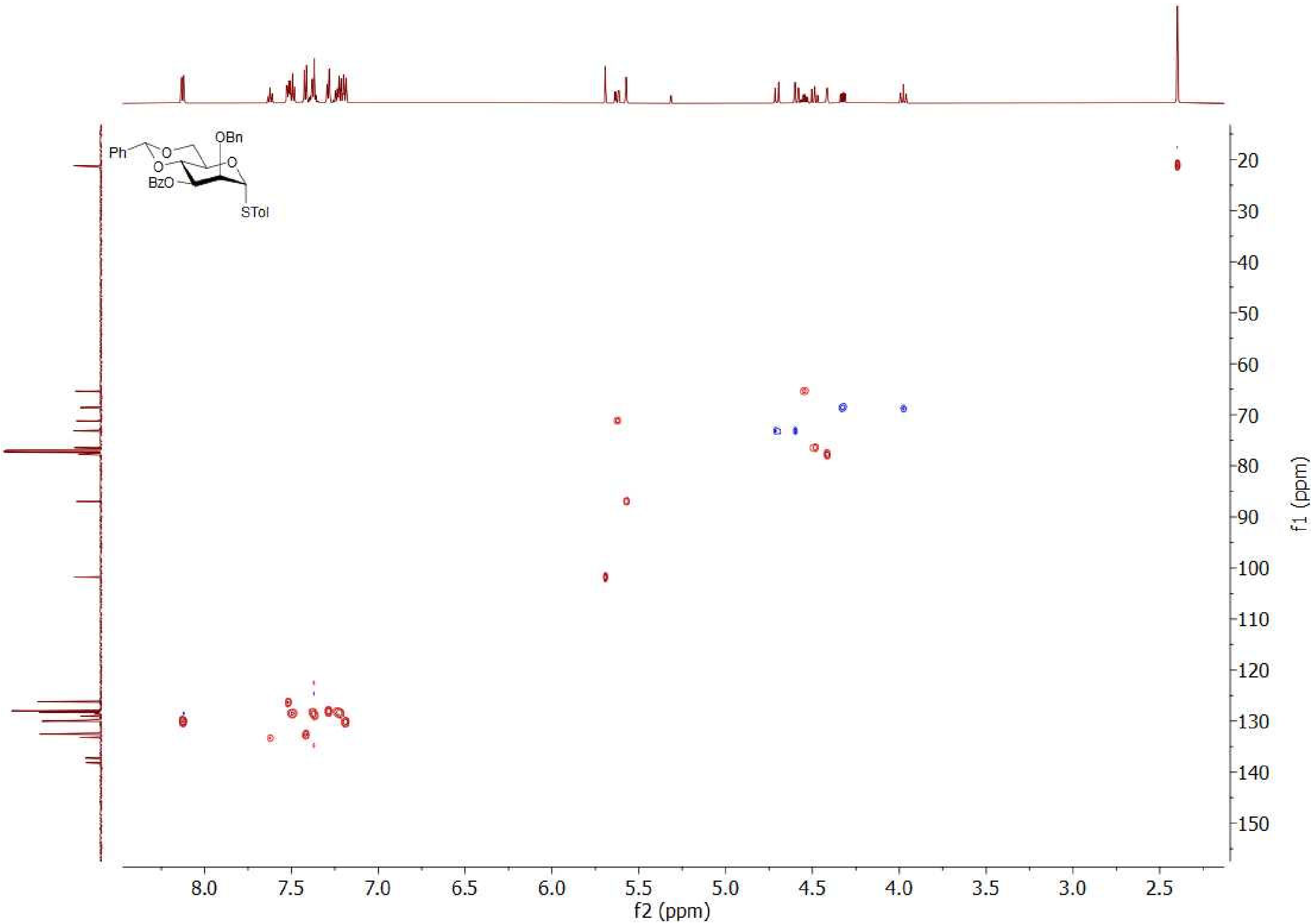

Figure S132. HMBC NMR (600 MHz, $CDCl_3$) spectrum of *p*-methylphenyl 3-*O*-benzoyl-2-*O*-benzyl-4,6-*O*-benzylidene-thio-α-D-mannopyranoside **48**:

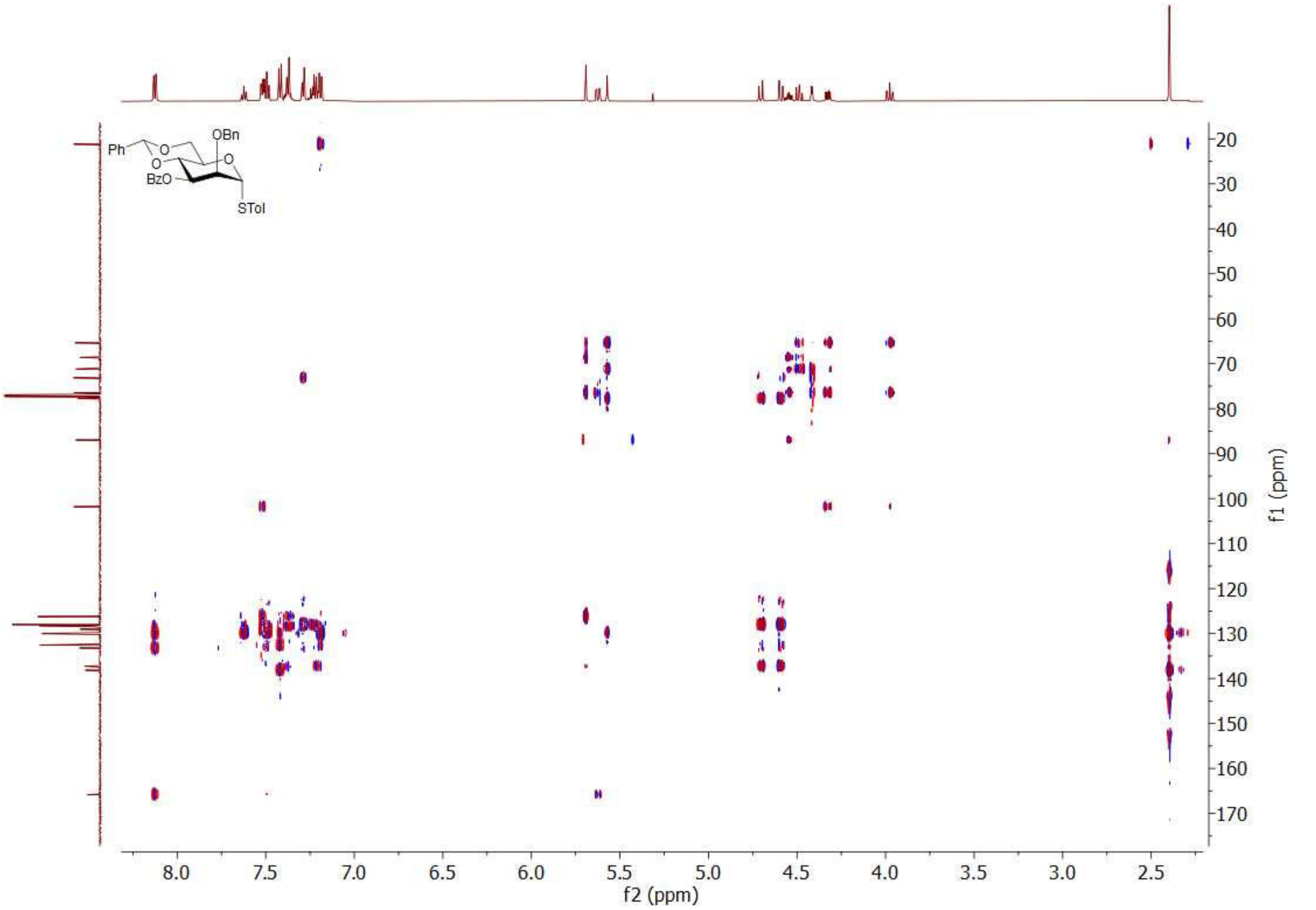

Figure S133. ^{1}H NMR (600 MHz, CDCl$_3$) spectrum of *p*-methylphenyl 3-*O*-(benzoyl-α-^{13}C)-2-*O*-benzyl-4,6-*O*-benzylidene-thio-α-D-mannopyranoside **^{13}C-48**:

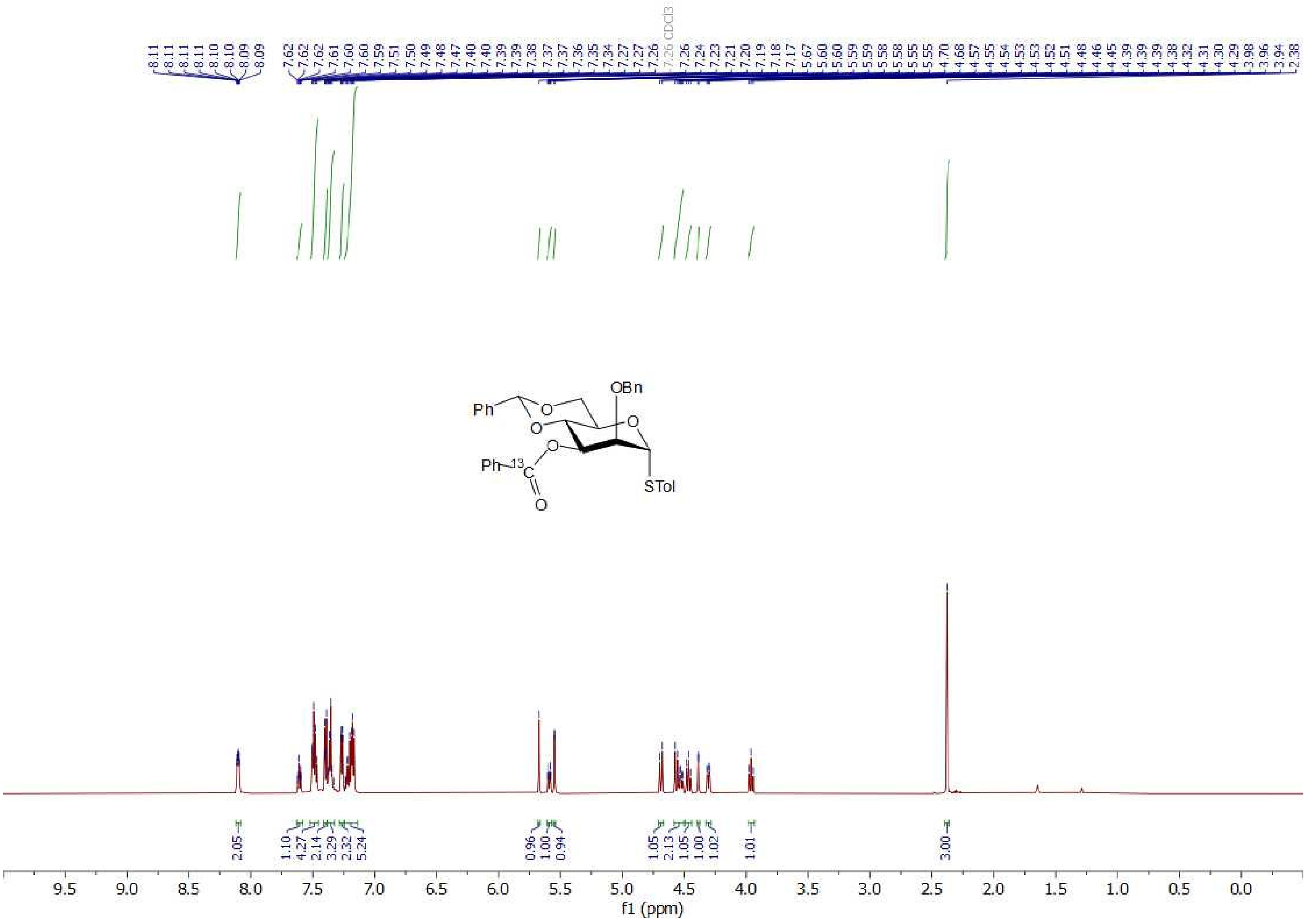

Figure S134. COSY NMR (600 MHz, $CDCl_3$) spectrum of *p*-methylphenyl 3-*O*-(benzoyl-α-^{13}C)-2-*O*-benzyl-4,6-*O*-benzylidene-thio-α-D-mannopyranoside **^{13}C-48**:

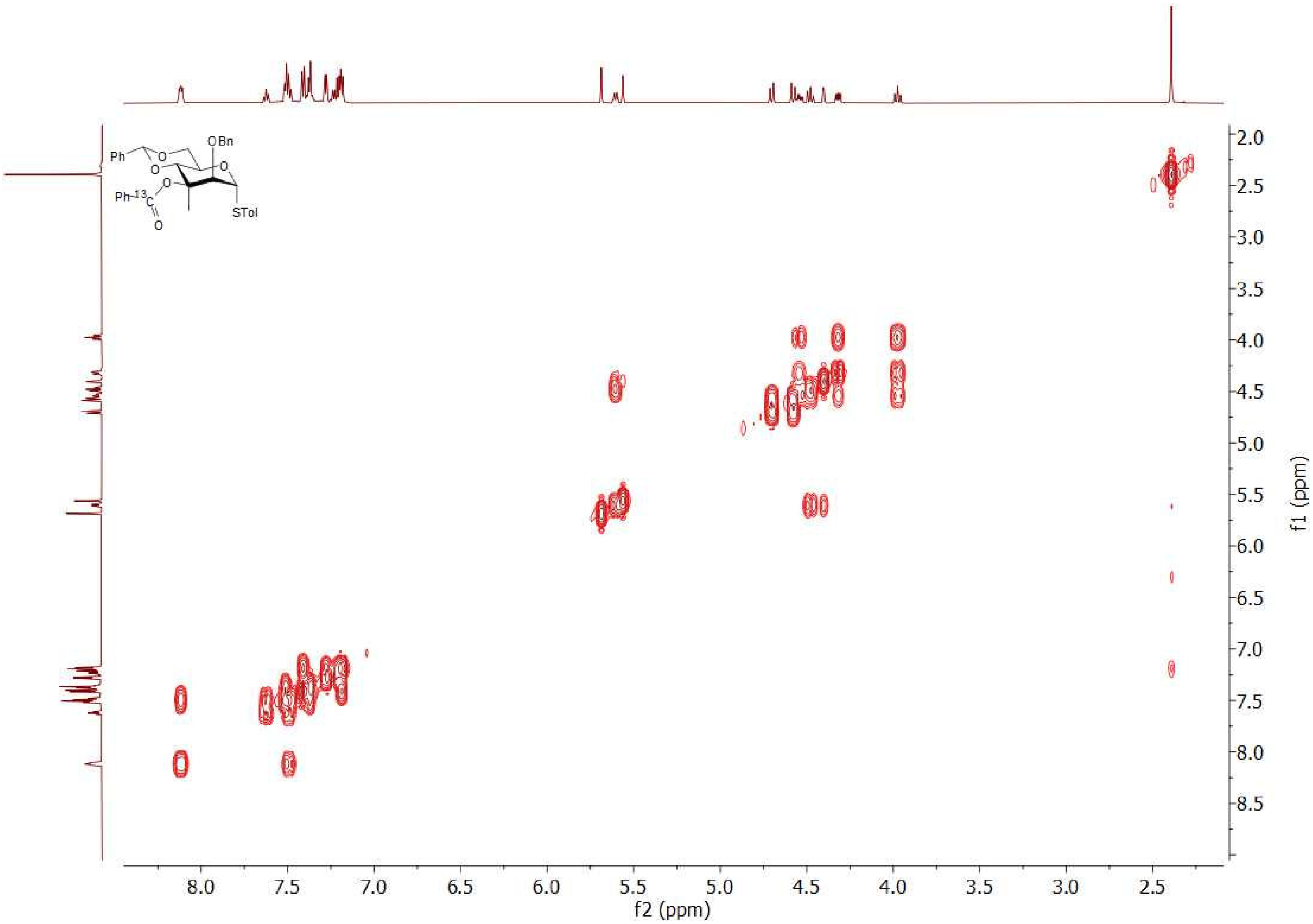

Figure S135. $^{13}C\{^{1}H\}$ NMR (151 MHz, $CDCl_3$) spectrum of *p*-methylphenyl 3-*O*-(benzoyl-α-^{13}C)-2-*O*-benzyl-4,6-*O*-benzylidene-thio-α-D-mannopyranoside **^{13}C-48**:

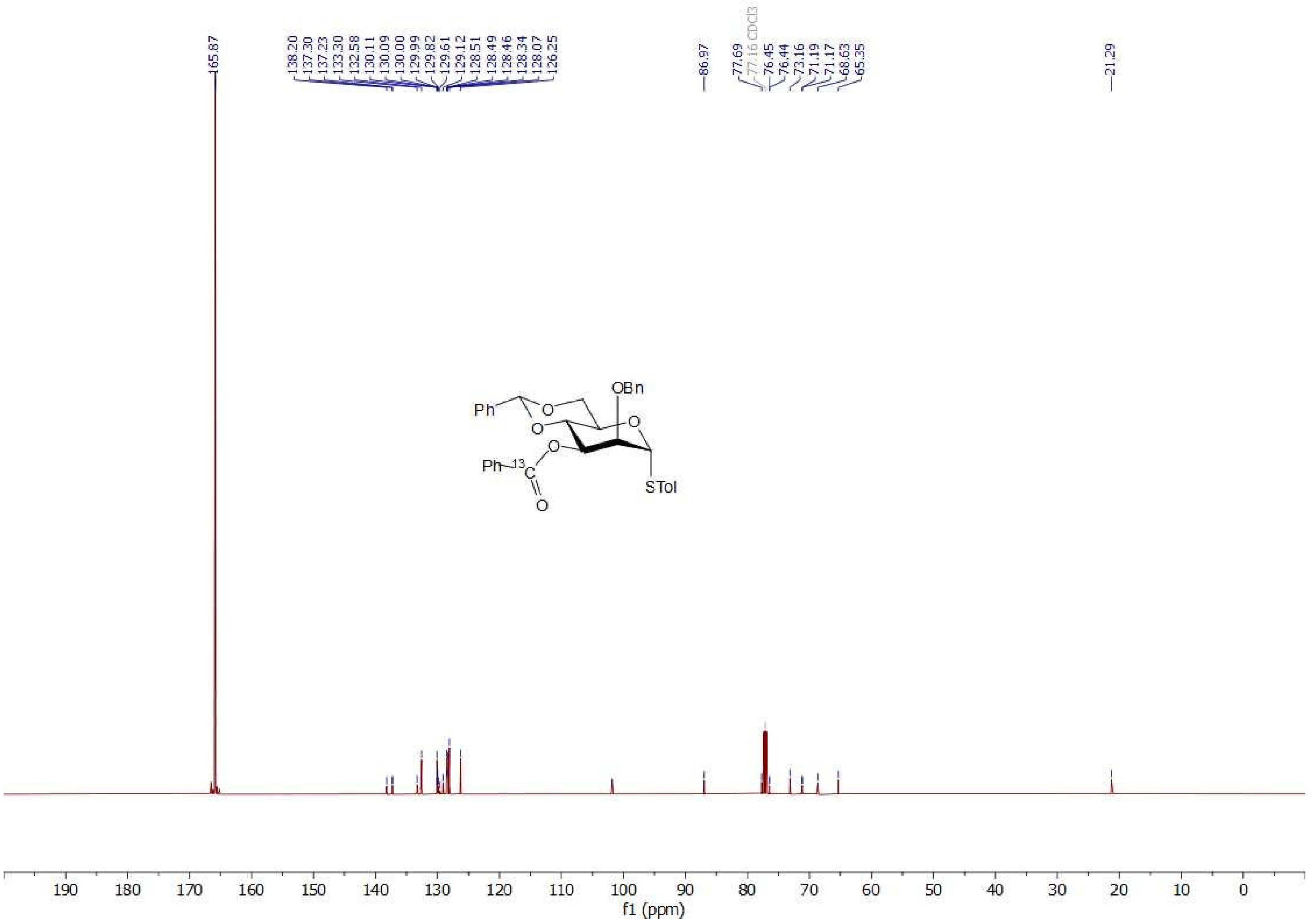

Figure S136. $^{13}C\{^{1}H\}$ DEPT NMR (151 MHz, $CDCl_3$) spectrum of *p*-methylphenyl 3-*O*-(benzoyl-α-^{13}C)-2-*O*-benzyl-4,6-*O*-benzylidene-thio-α-D-mannopyranoside **^{13}C-48**:

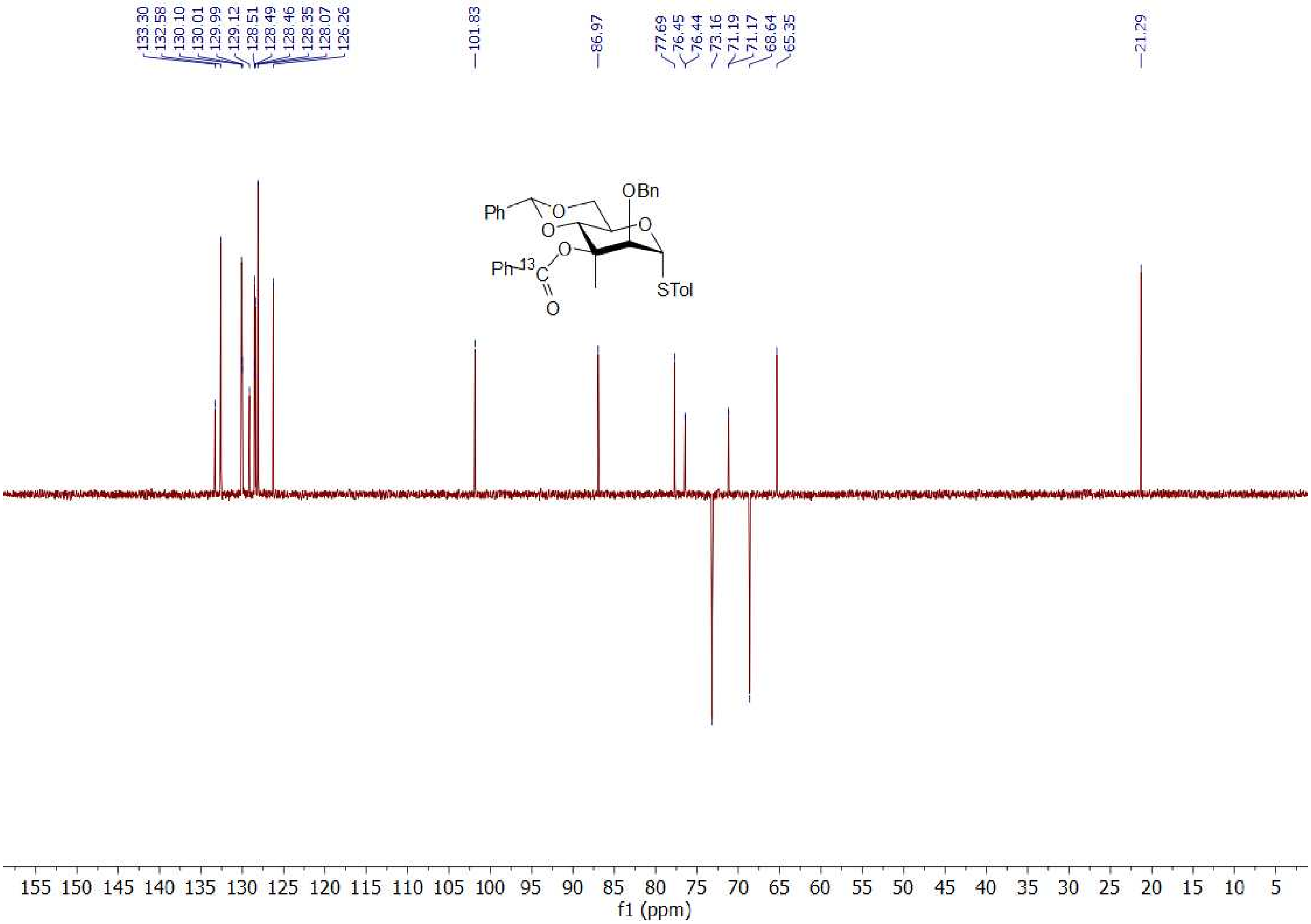

Figure S137. HSQC NMR (600 MHz, $CDCl_3$) spectrum of *p*-methylphenyl 3-*O*-(benzoyl-α-^{13}C)-2-*O*-benzyl-4,6-*O*-benzylidene-thio-α-D-mannopyranoside **^{13}C-48**:

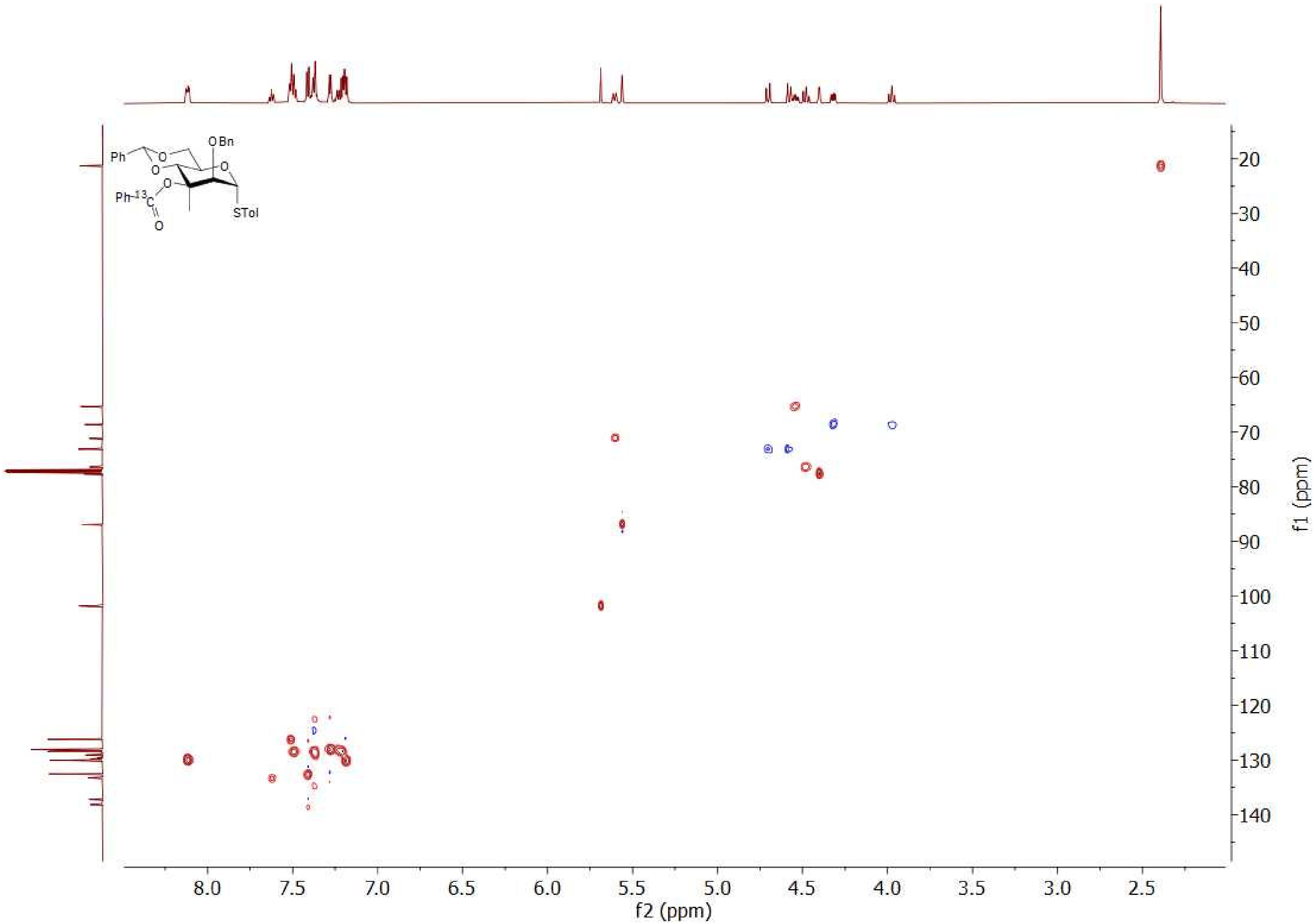

Figure S138. HMBC NMR (600 MHz, $CDCl_3$) spectrum of *p*-methylphenyl 3-*O*-(benzoyl-α-^{13}C)-2-*O*-benzyl-4,6-*O*-benzylidene-thio-α-D-mannopyranoside **^{13}C-48**:

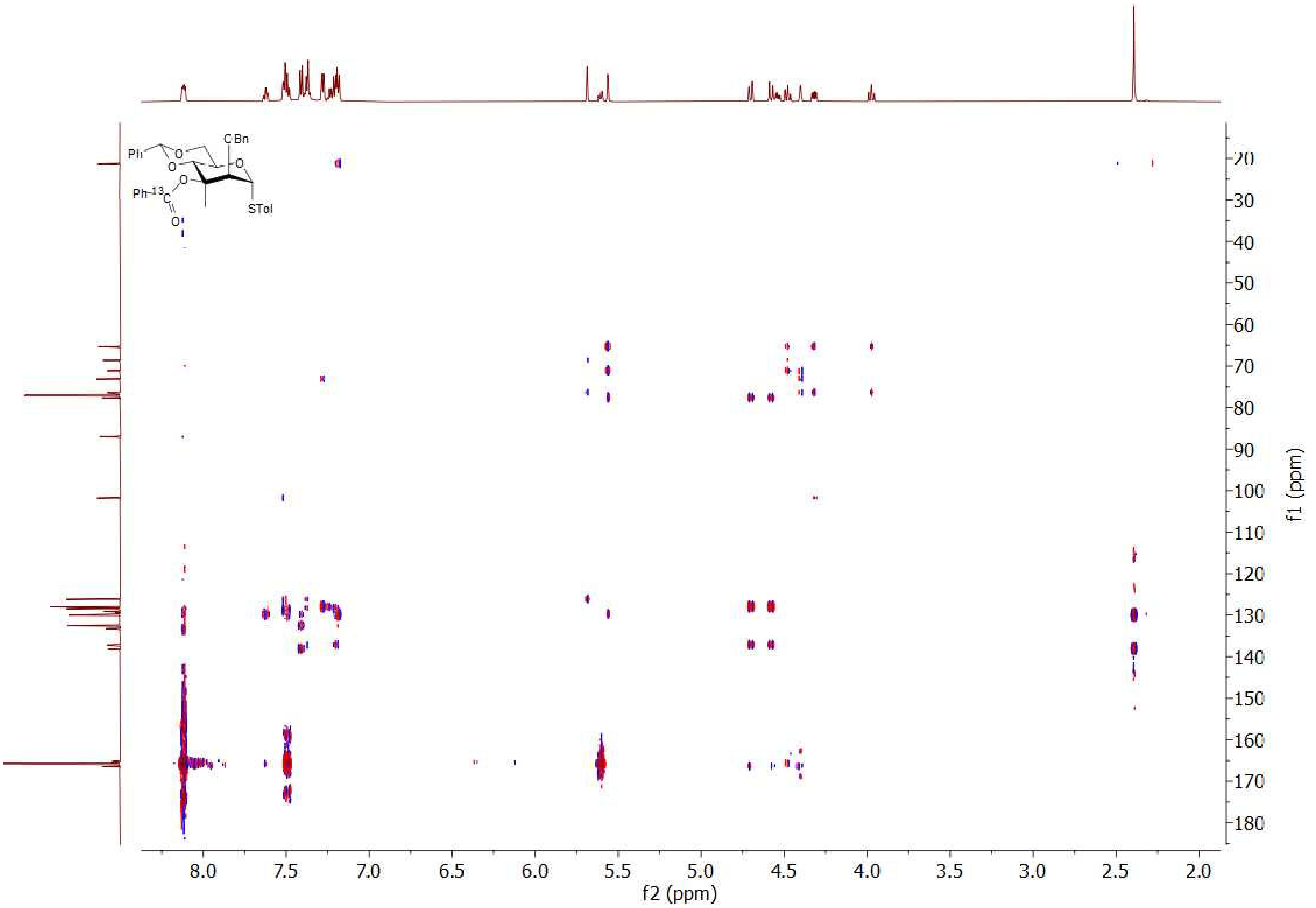

Figure S139. ^{1}H NMR (600 MHz, $CDCl_3$) spectrum of *p*-methylphenyl 3-*O*-benzoyl-2-*O*-benzyl-4,6-*O*-benzylidene-thio-α-D-mannopyranoside *S*-oxide **49**:

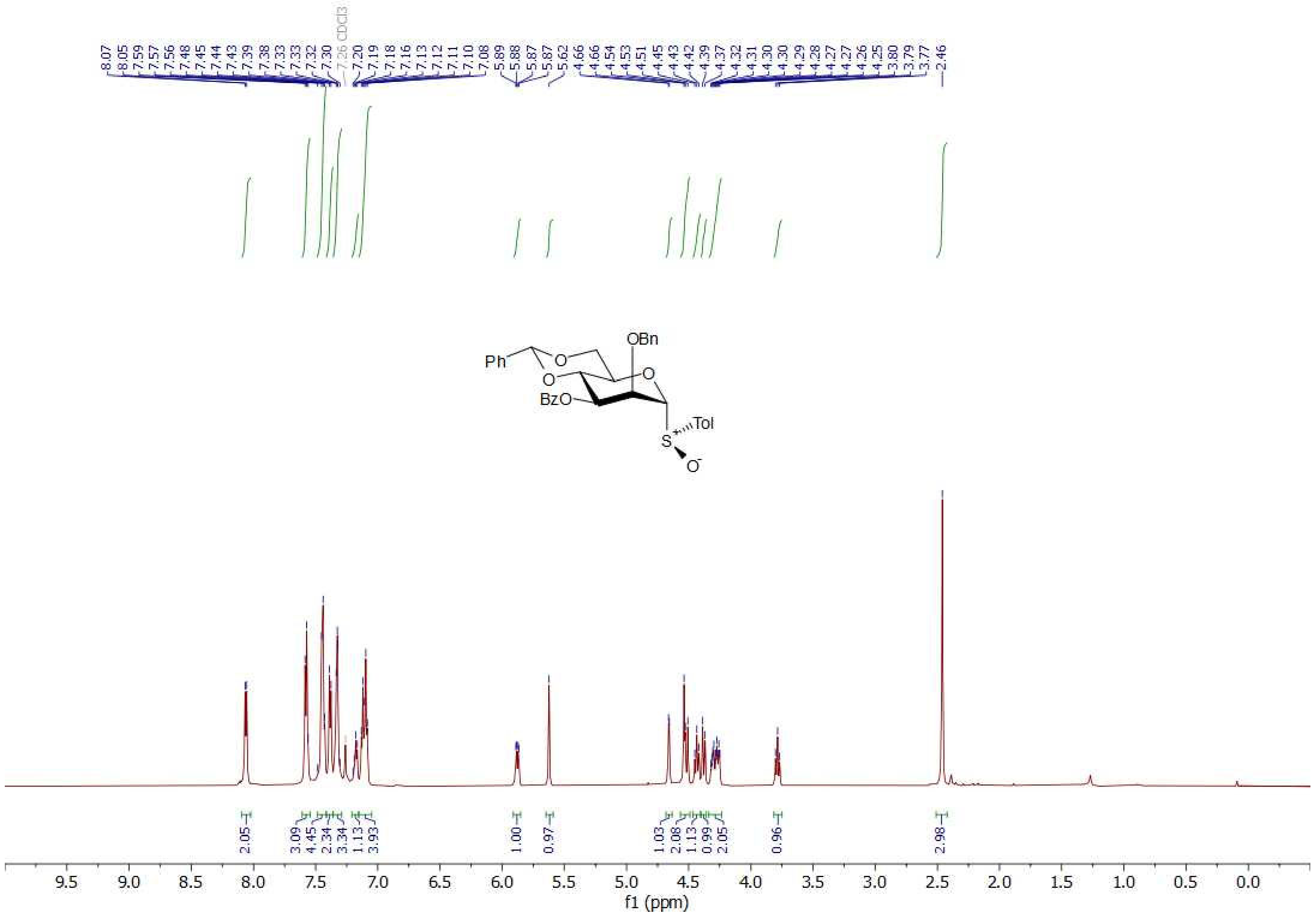

Figure S140. COSY NMR (600 MHz, $CDCl_3$) spectrum of *p*-methylphenyl 3-*O*-benzoyl-2-*O*-benzyl-4,6-*O*-benzylidene-thio-α-D-mannopyranoside *S*-oxide **49**:

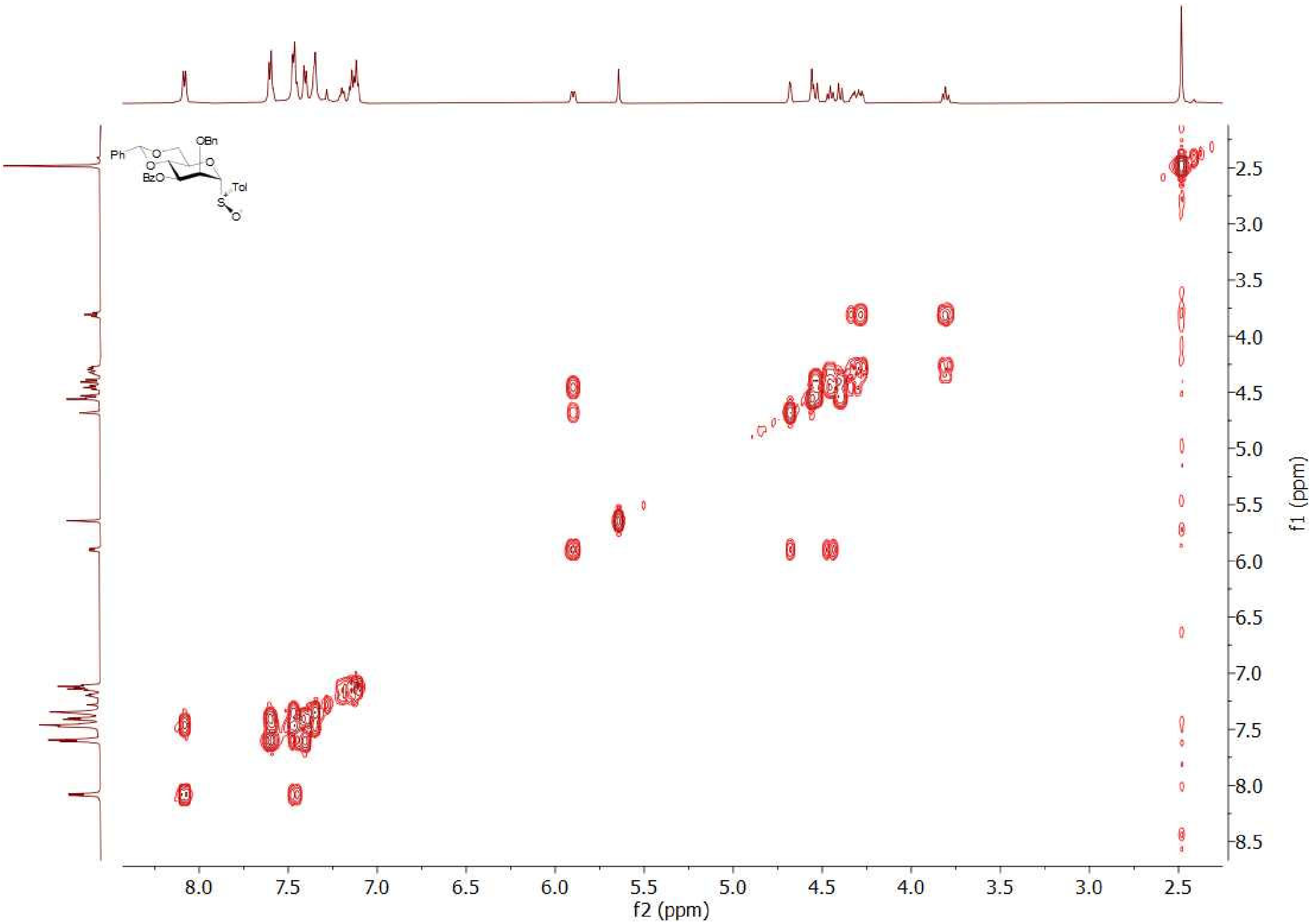

Figure S141. $^{13}C\{^{1}H\}$ NMR (151 MHz, $CDCl_3$) spectrum of *p*-methylphenyl 3-*O*-benzoyl-2-*O*-benzyl-4,6-*O*-benzylidene-thio-α-D-mannopyranoside *S*-oxide **49**:

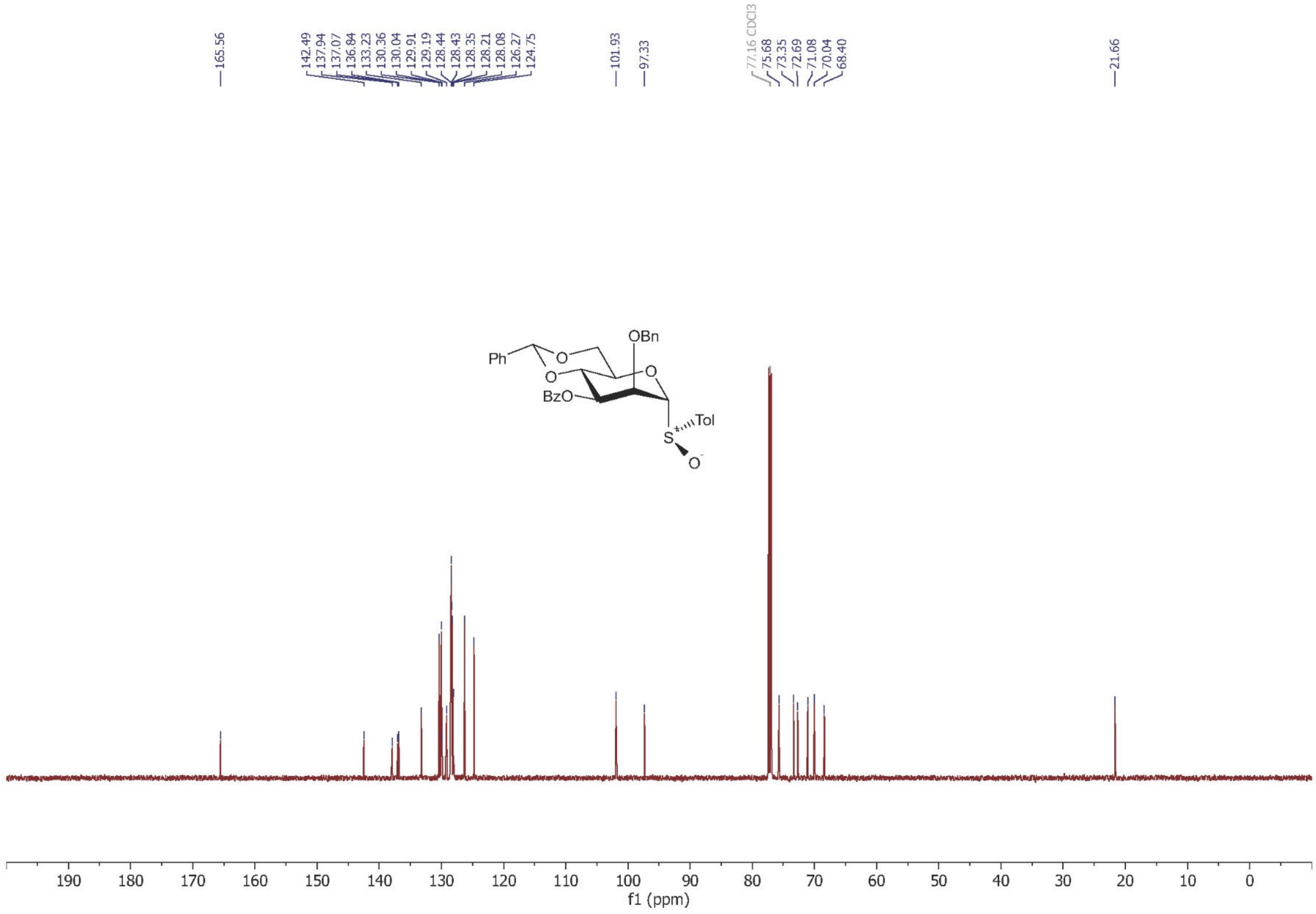

Figure S142. $^{13}C\{^1H\}$ DEPT NMR (151 MHz, $CDCl_3$) spectrum of *p*-methylphenyl 3-*O*-benzoyl-2-*O*-benzyl-4,6-*O*-benzylidene-thio-α-D-mannopyranoside *S*-oxide **49**:

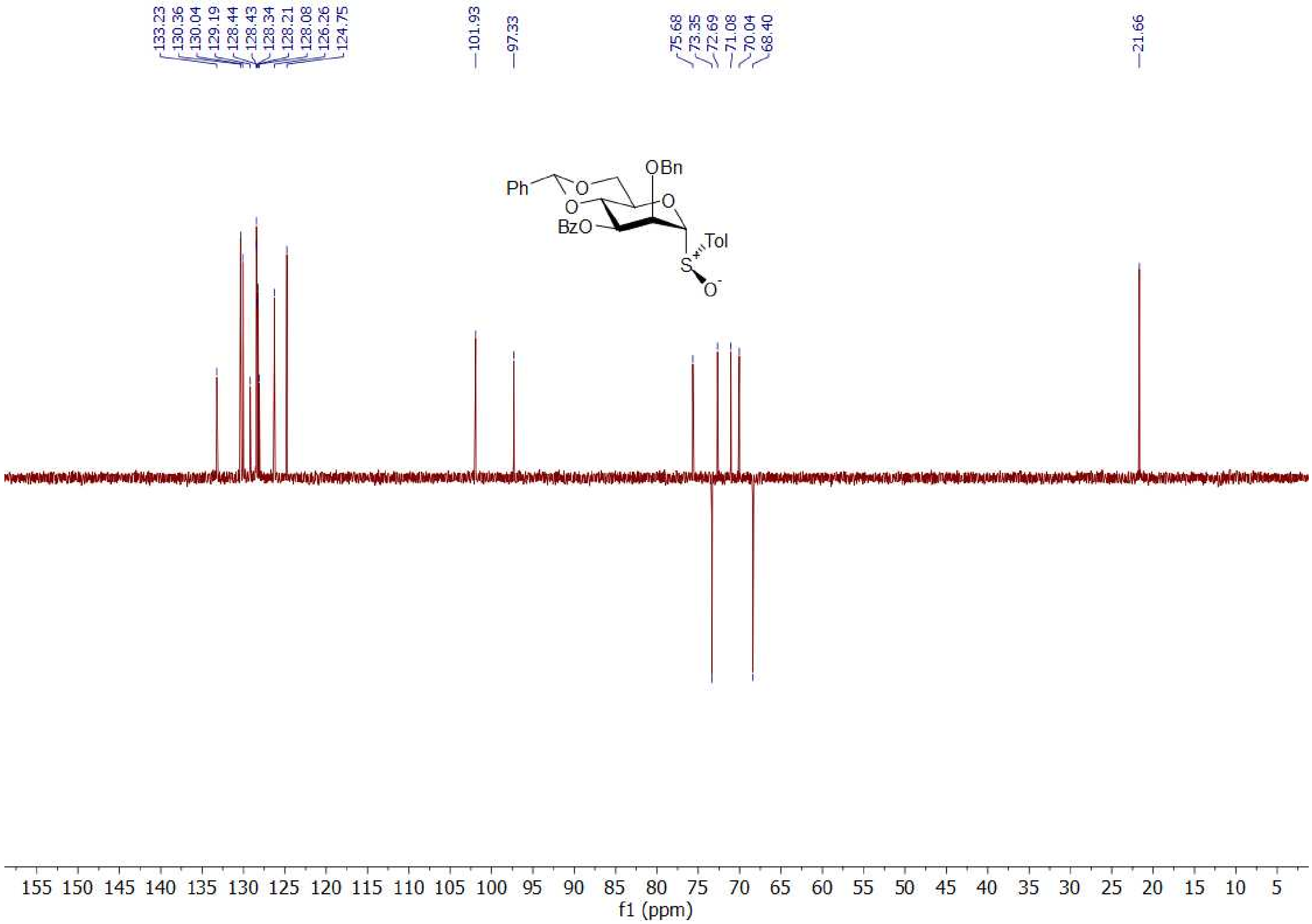

Figure S143. HSQC NMR (600 MHz, $CDCl_3$) spectrum of *p*-methylphenyl 3-*O*-benzoyl-2-*O*-benzyl-4,6-*O*-benzylidene-thio-α-D-mannopyranoside *S*-oxide **49**:

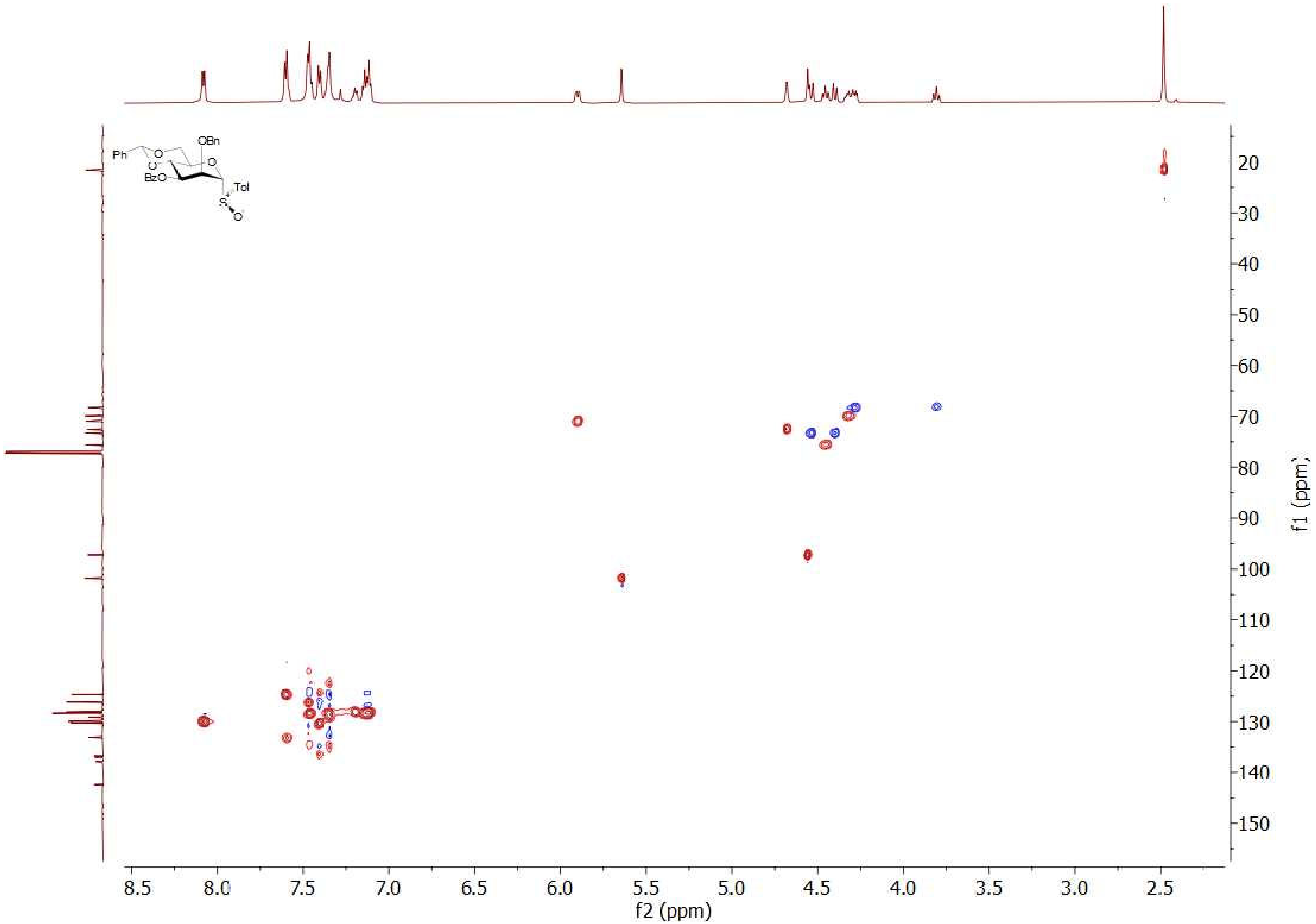

Figure S144. HMBC NMR (600 MHz, $CDCl_3$) spectrum of *p*-methylphenyl 3-*O*-benzoyl-2-*O*-benzyl-4,6-*O*-benzylidene-thio-α-D-mannopyranoside *S*-oxide **49**:

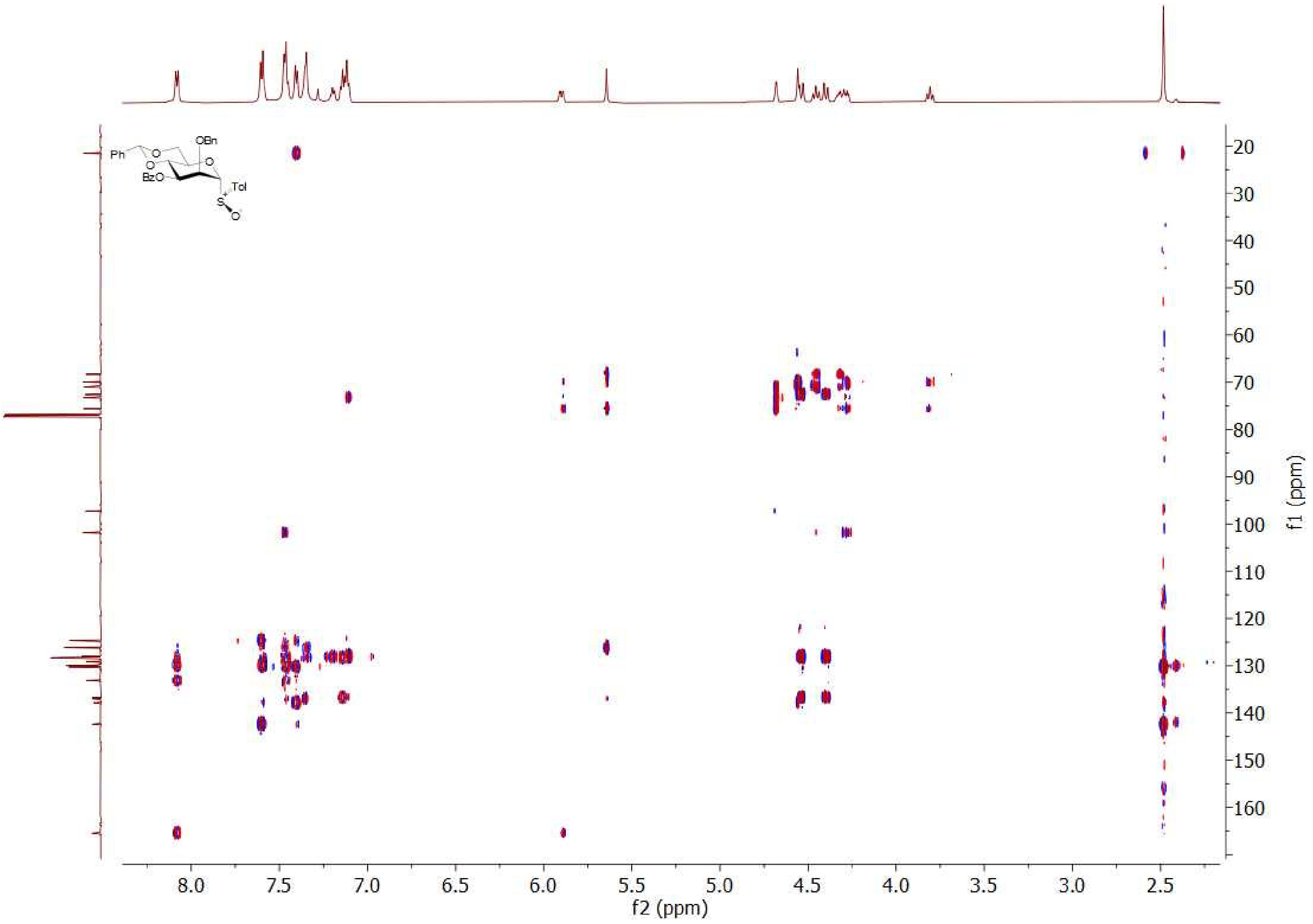

Figure S145. ^{1}H NMR (600 MHz, $CDCl_3$) spectrum of *p*-methylphenyl 3-*O*-(benzoyl-α-^{13}C)-2-*O*-benzyl-4,6-*O*-benzylidene-thio-α-D-mannopyranoside *S*-oxide **^{13}C-49**:

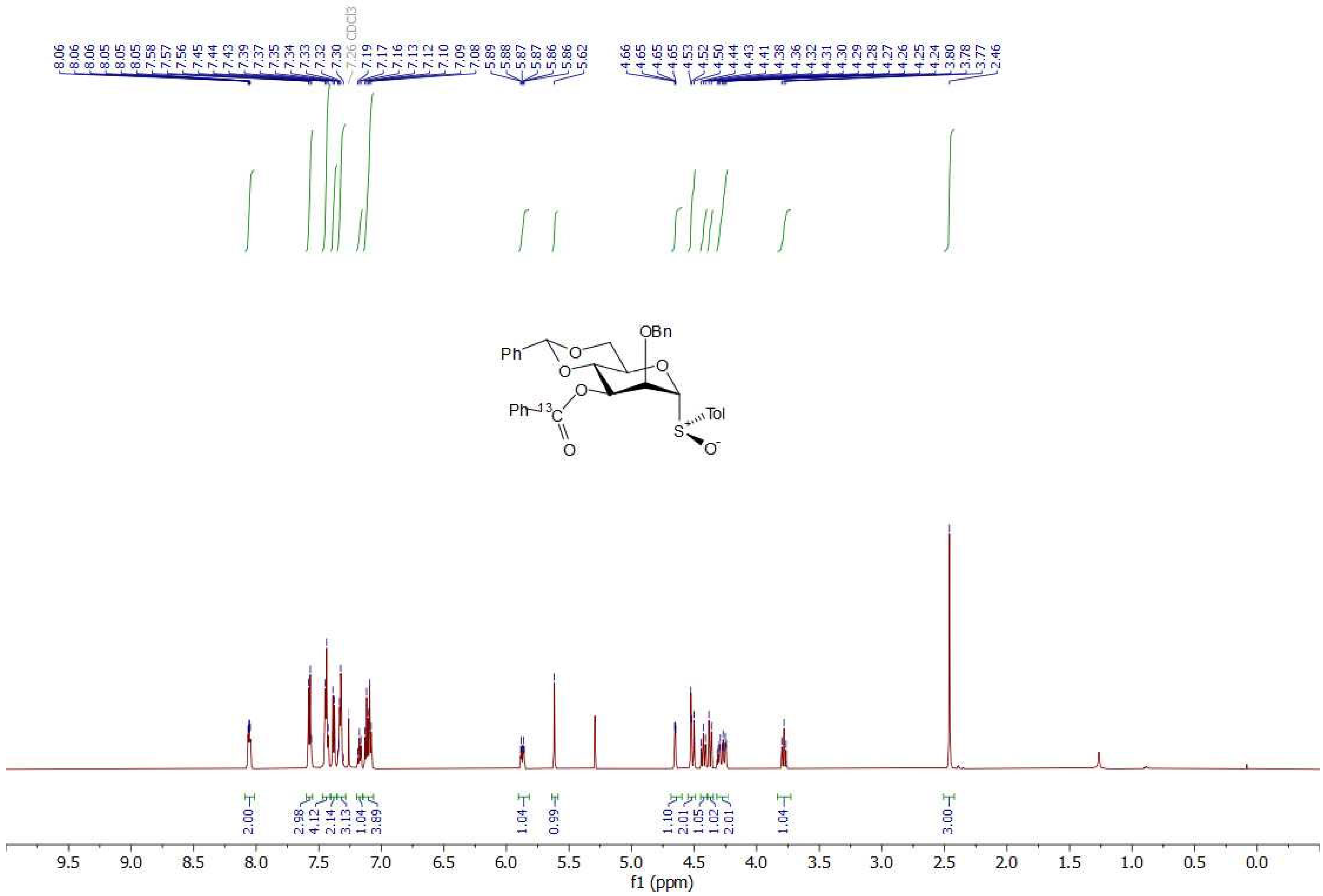

Figure S146. COSY NMR (600 MHz, $CDCl_3$) spectrum of *p*-methylphenyl 3-*O*-(benzoyl-α-^{13}C)-2-*O*-benzyl-4,6-*O*-benzylidene-thio-α-D-mannopyranoside *S*-oxide **^{13}C-49**:

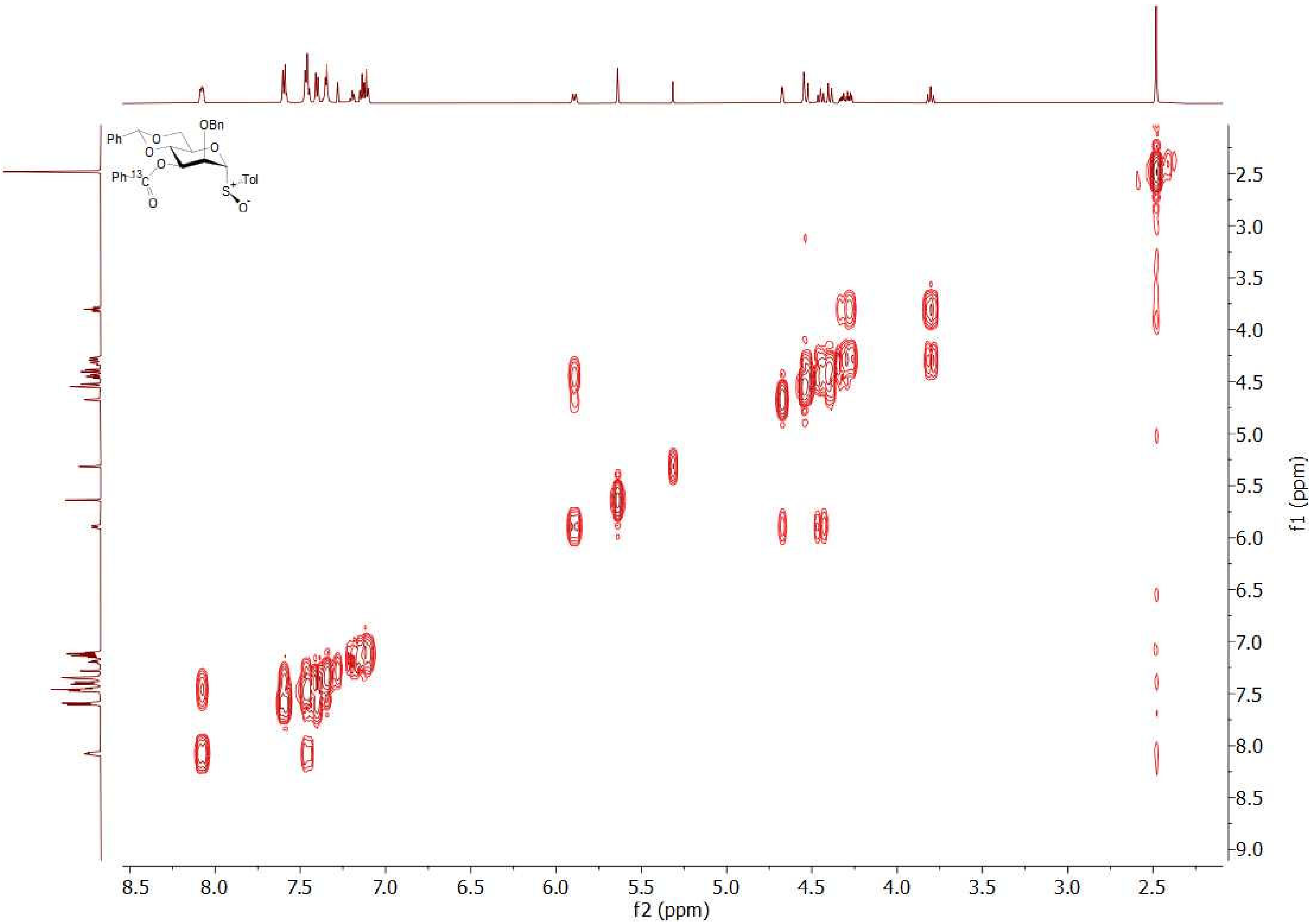

Figure S147. $^{13}C\{^1H\}$ NMR (151 MHz, $CDCl_3$) spectrum of *p*-methylphenyl 3-*O*-(benzoyl-α-^{13}C)-2-*O*-benzyl-4,6-*O*-benzylidene-thio-α-D-mannopyranoside *S*-oxide **^{13}C-49**:

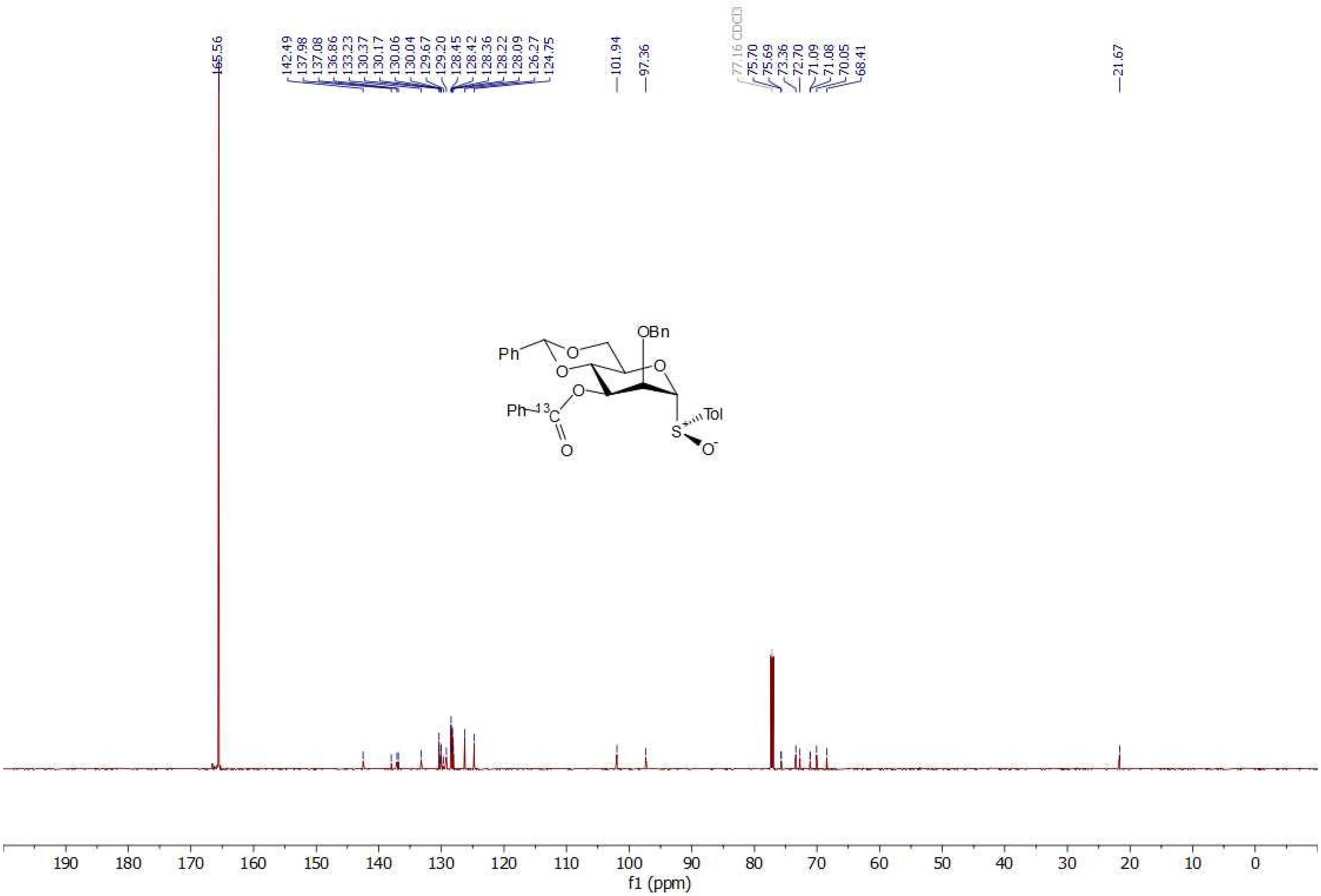

Figure S148. $^{13}C\{^1H\}$ DEPT NMR (151 MHz, $CDCl_3$) spectrum of *p*-methylphenyl 3-*O*-(benzoyl-α-^{13}C)-2-*O*-benzyl-4,6-*O*-benzylidene-thio-α-D-mannopyranoside *S*-oxide **^{13}C-49**:

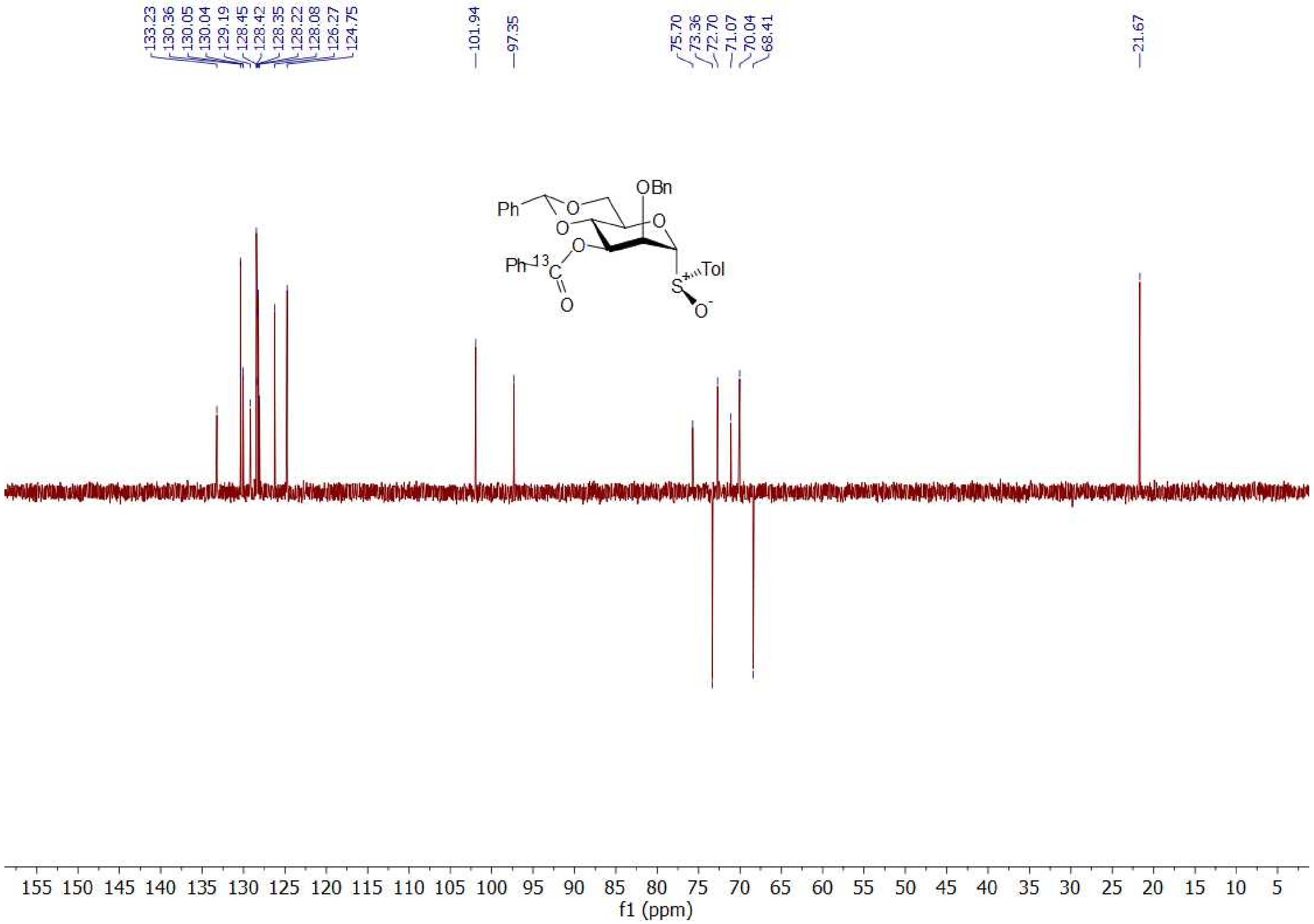

Figure S149. HSQC NMR (600 MHz, $CDCl_3$) spectrum of *p*-methylphenyl 3-*O*-(benzoyl-α-^{13}C)-2-*O*-benzyl-4,6-*O*-benzylidene-thio-α-D-mannopyranoside *S*-oxide **^{13}C-49**:

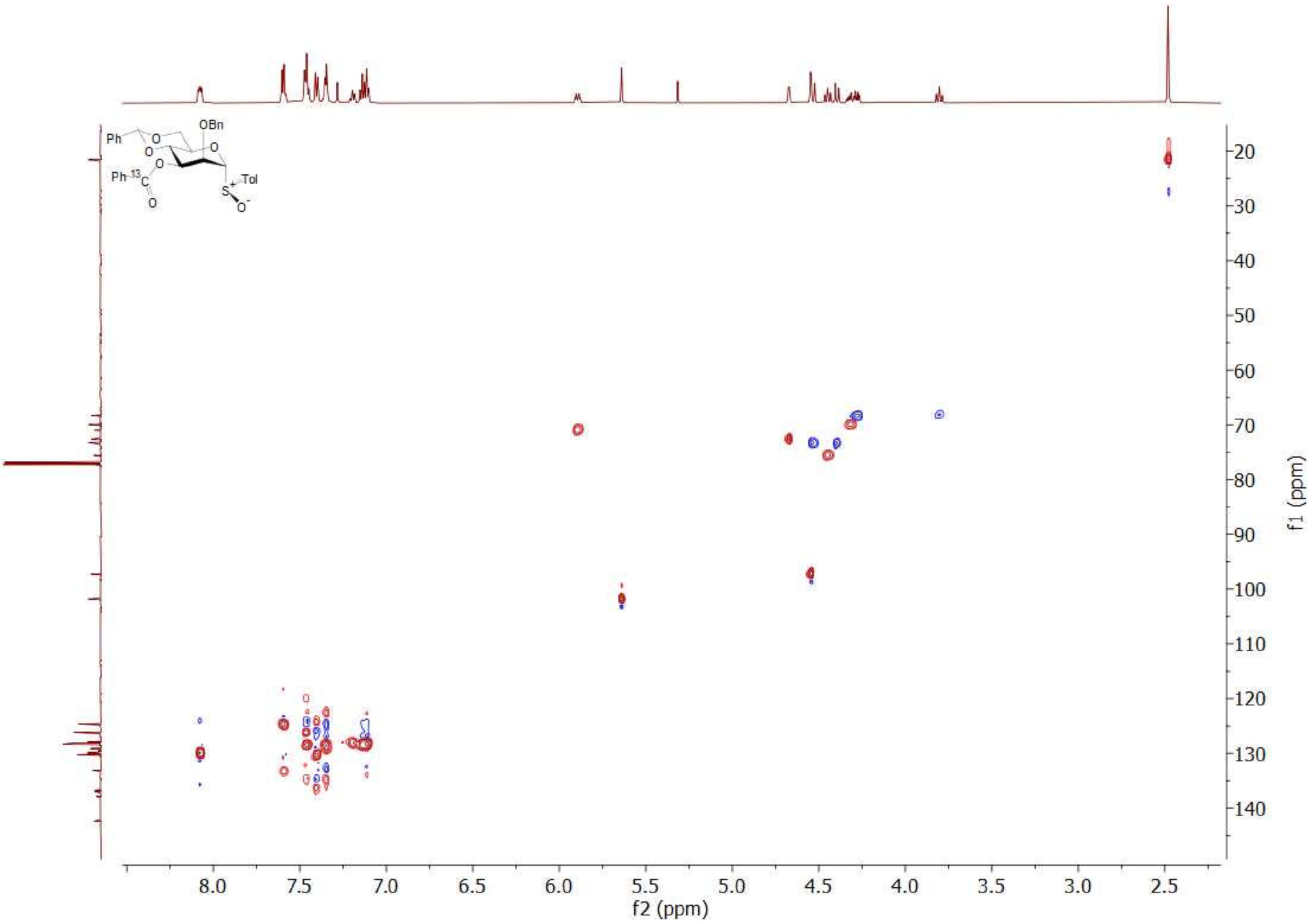

Figure S150. HMBC NMR (600 MHz, $CDCl_3$) spectrum of *p*-methylphenyl 3-*O*-(benzoyl-α-^{13}C)-2-*O*-benzyl-4,6-*O*-benzylidene-thio-α-D-mannopyranoside *S*-oxide **^{13}C-49**:

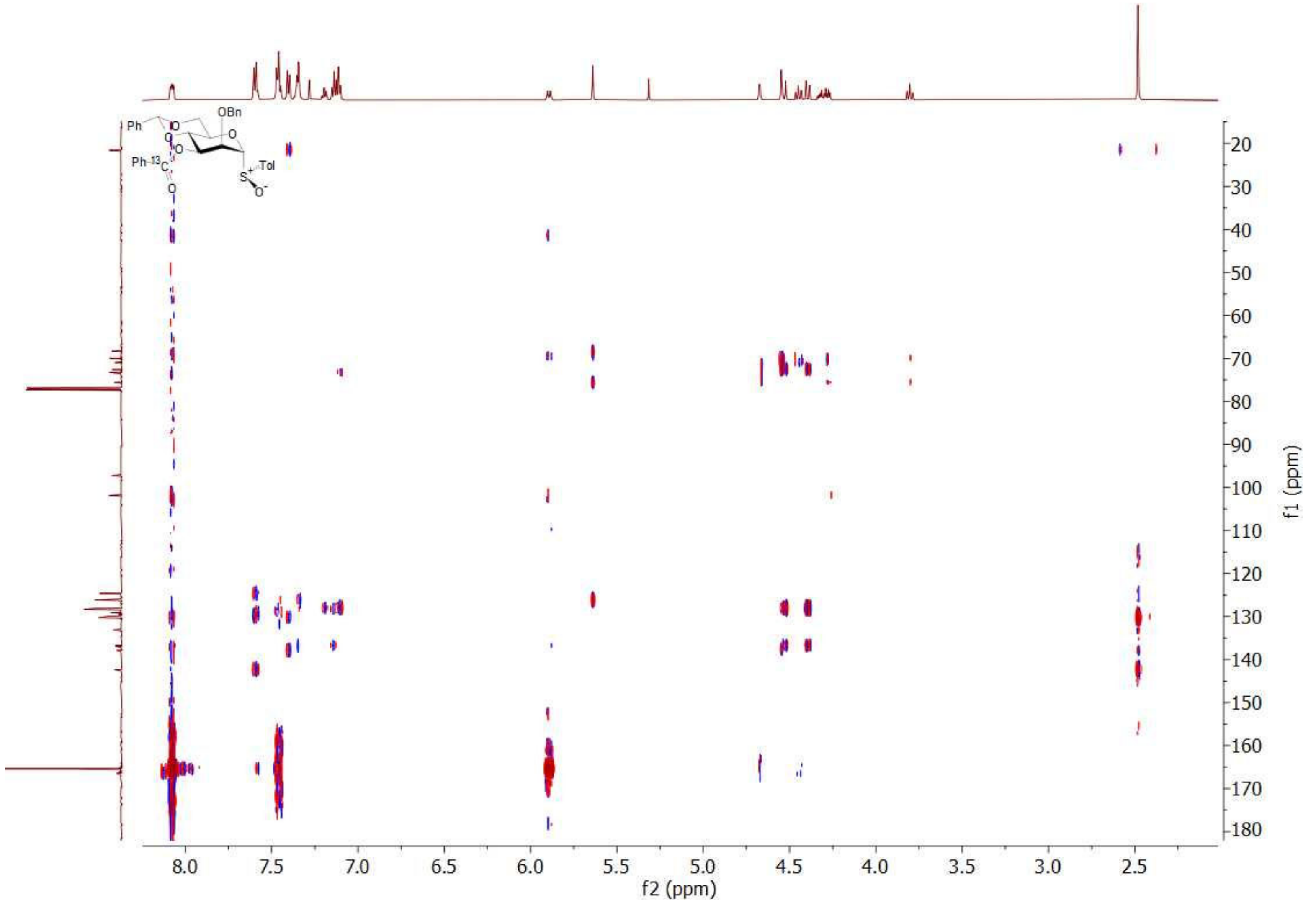

Figure S151. ^{1}H NMR (600 MHz, $CDCl_3$) spectrum of 6-*O*-(3-*O*-benzoyl-2-*O*-benzyl-4,6-*O*-benzylidene-α-D-mannopyranosyl)-1,2:3,4-di-*O*-isopropylidene-α-D-galactopyranose **52**:

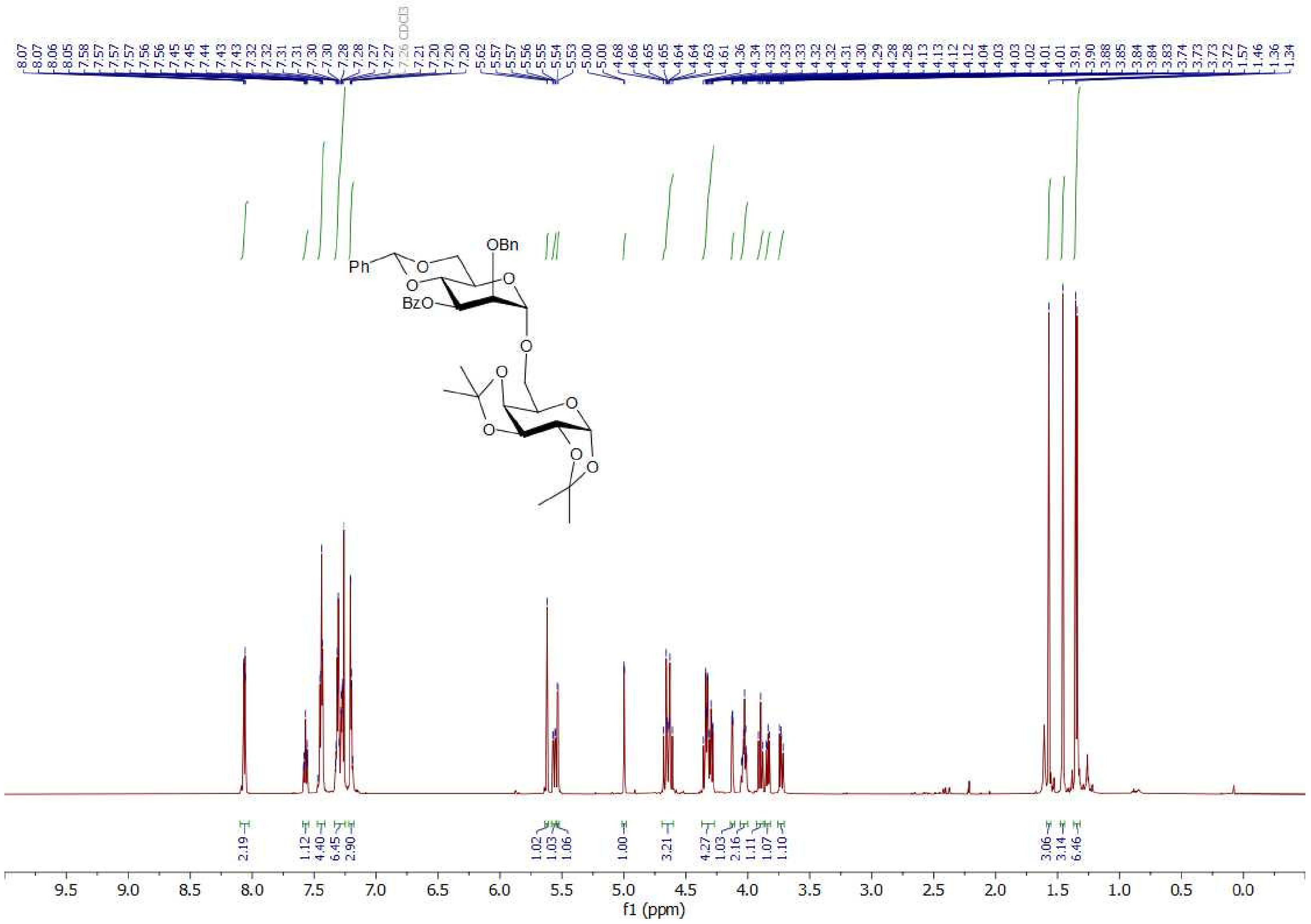

Figure S152. $^{13}C\{^{1}H\}$ NMR (151 MHz, $CDCl_3$) spectrum of 6-*O*-(3-*O*-benzoyl-2-*O*-benzyl-4,6-*O*-benzylidene-α-D-mannopyranosyl)-1,2:3,4-di-*O*-isopropylidene-α-D-galactopyranose **52**:

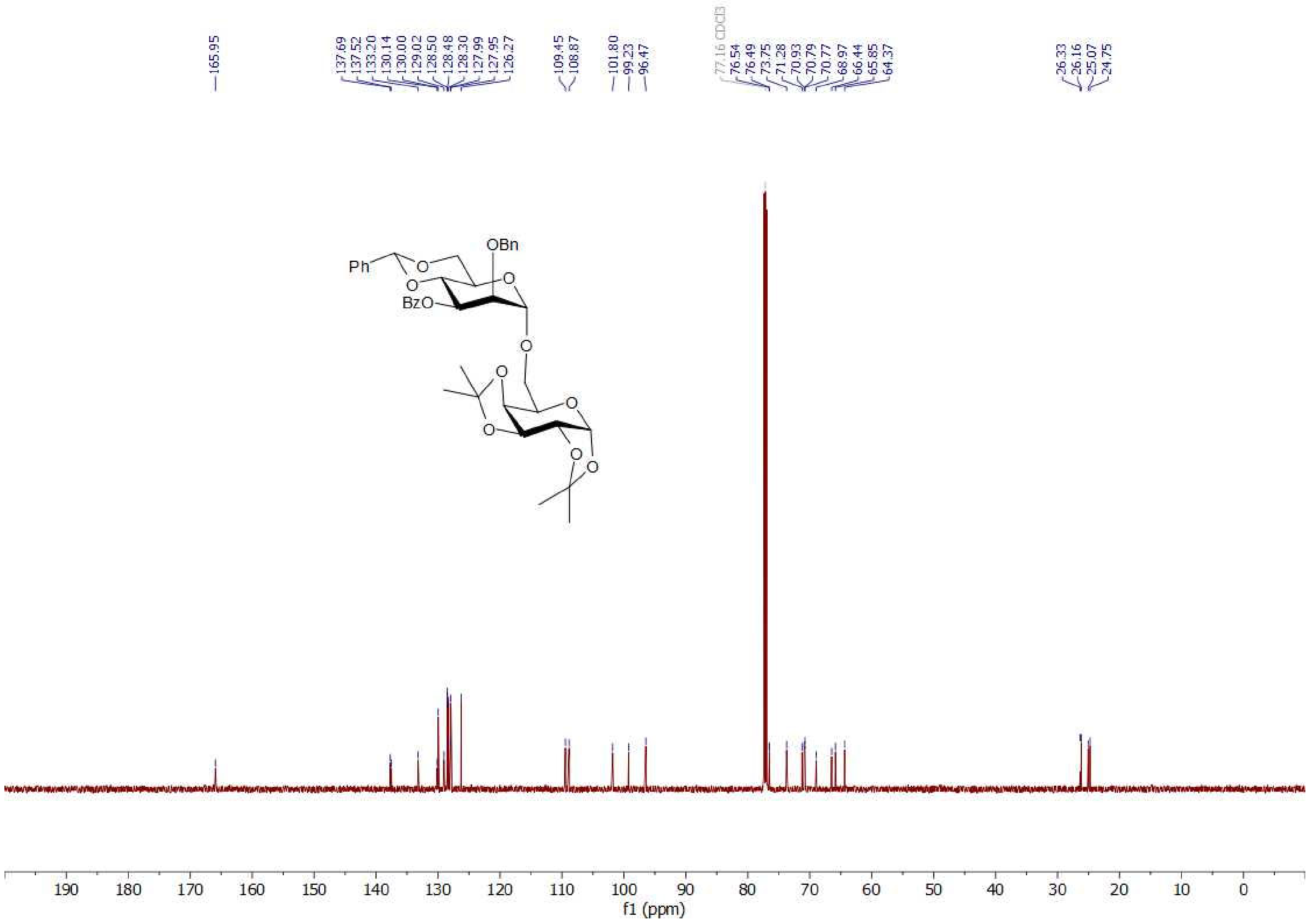

Figure S153. COSY NMR (600 MHz, $CDCl_3$) spectrum of 6-*O*-(3-*O*-benzoyl-2-*O*-benzyl-4,6-*O*-benzylidene-α-D-mannopyranosyl)-1,2:3,4-di-*O*-isopropylidene-α-D-galactopyranose **52**:

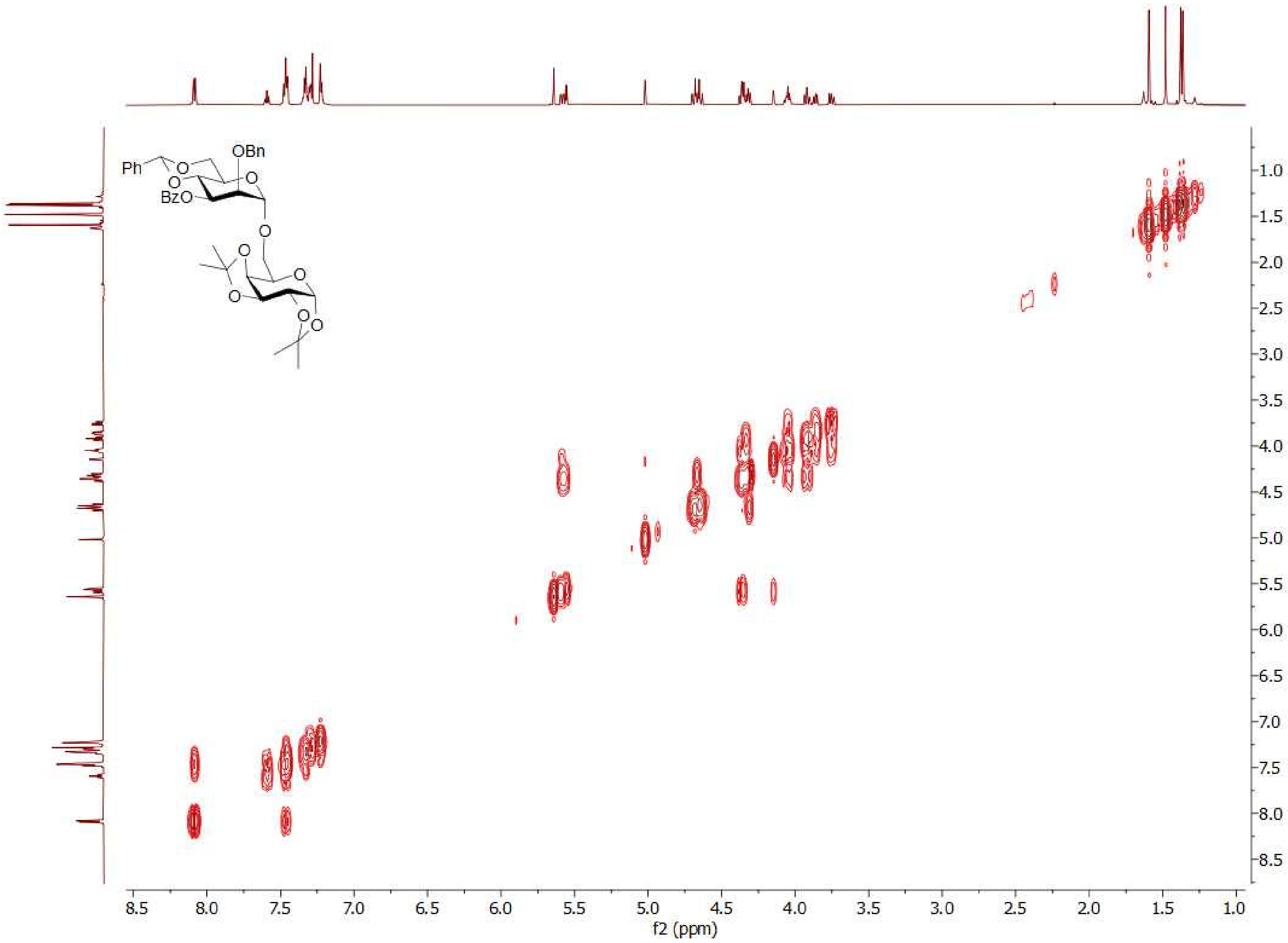

Figure S154. $^{13}C\{^1H\}$ DEPT NMR (151 MHz, $CDCl_3$) spectrum of 6-*O*-(3-*O*-benzoyl-2-*O*-benzyl-4,6-*O*-benzylidene-α-D-mannopyranosyl)-1,2:3,4-di-*O*-isopropylidene-α-D-galactopyranose **52**:

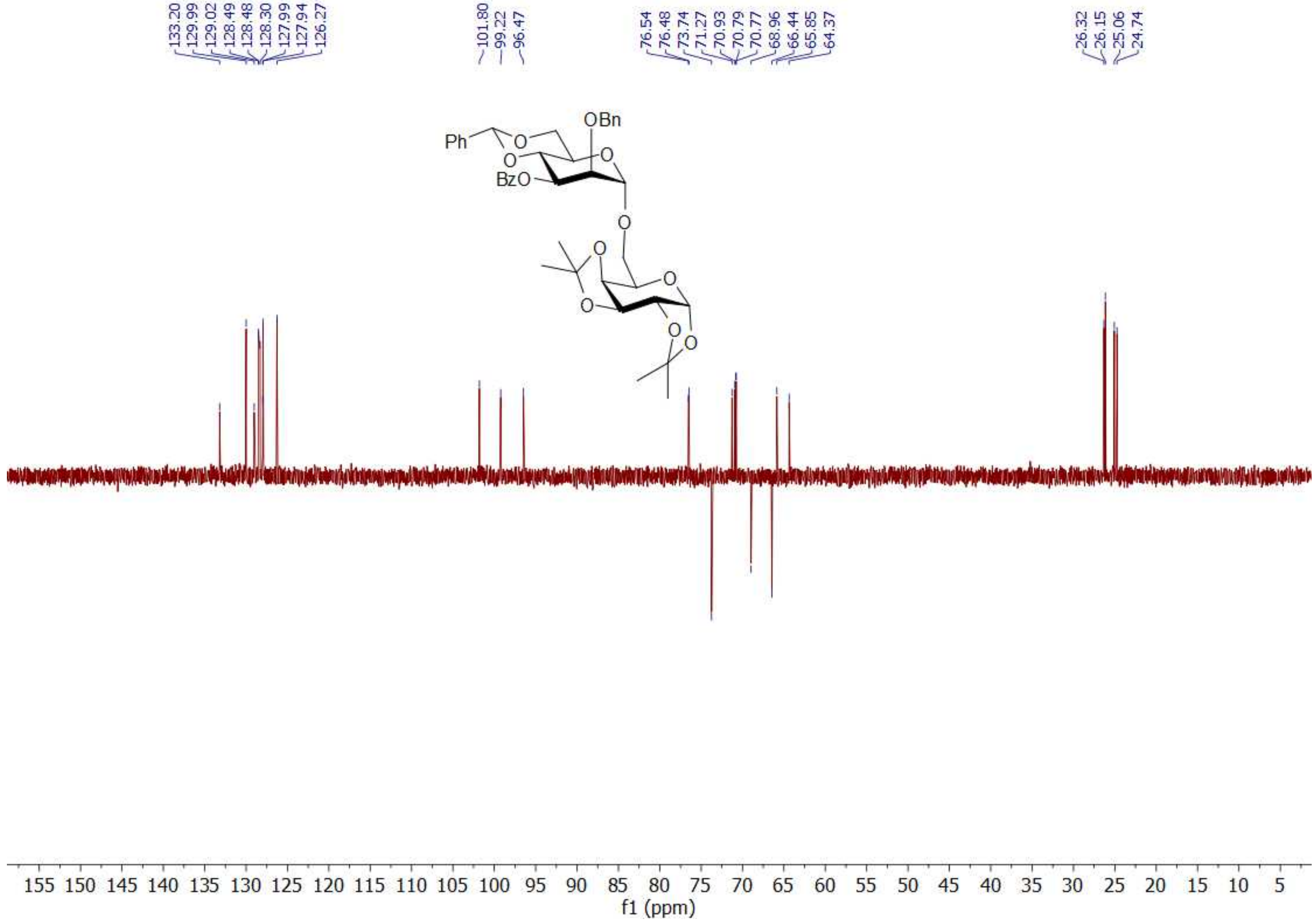

Figure S155. HSQC NMR (600 MHz, $CDCl_3$) spectrum of 6-*O*-(3-*O*-benzoyl-2-*O*-benzyl-4,6-*O*-benzylidene-α-D-mannopyranosyl)-1,2:3,4-di-*O*-isopropylidene-α-D-galactopyranose **52**:

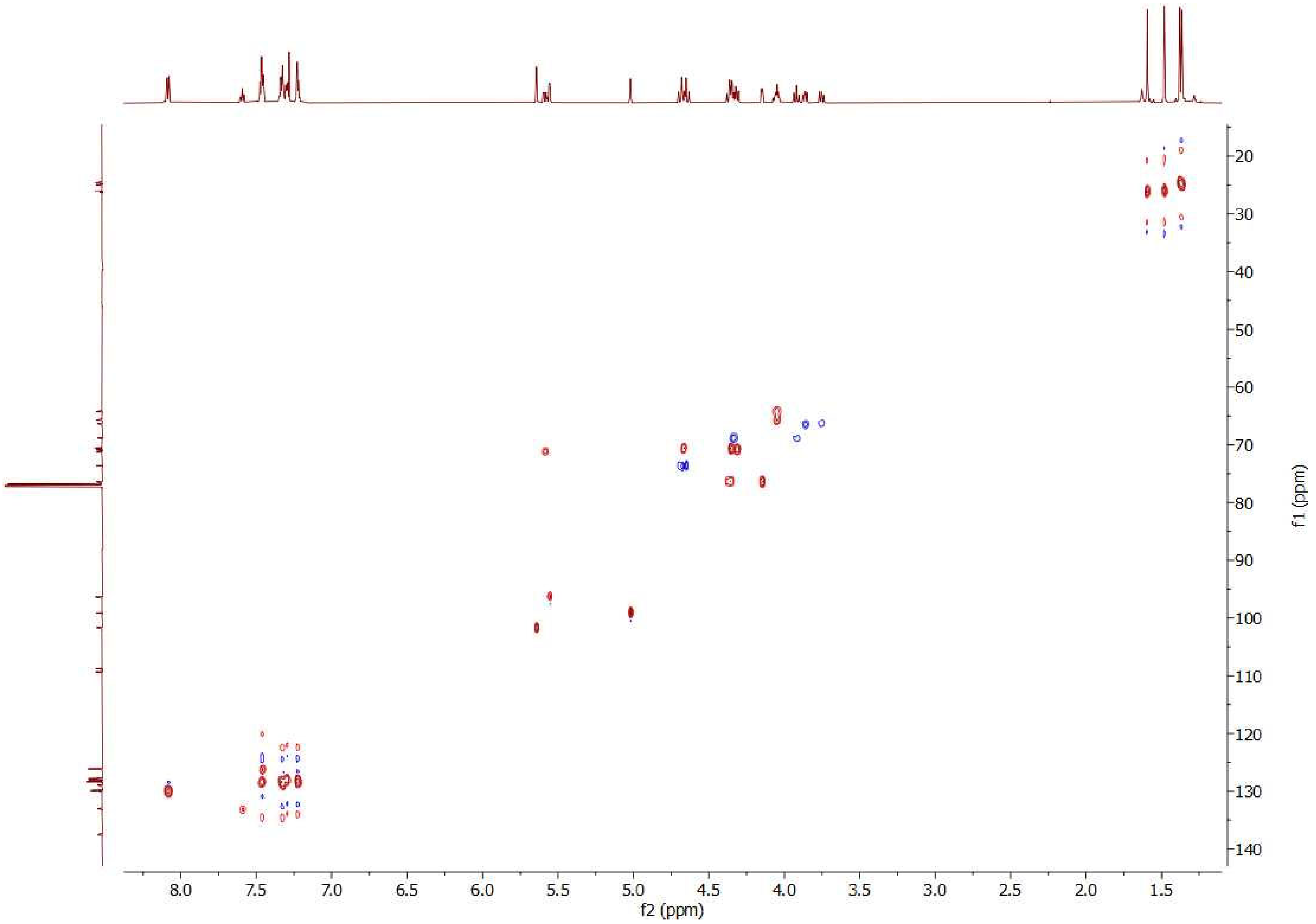

Figure S156. HMBC NMR (600 MHz, $CDCl_3$) spectrum of 6-*O*-(3-*O*-benzoyl-2-*O*-benzyl-4,6-*O*-benzylidene-α-D-mannopyranosyl)-1,2:3,4-di-*O*-isopropylidene-α-D-galactopyranose **52**:

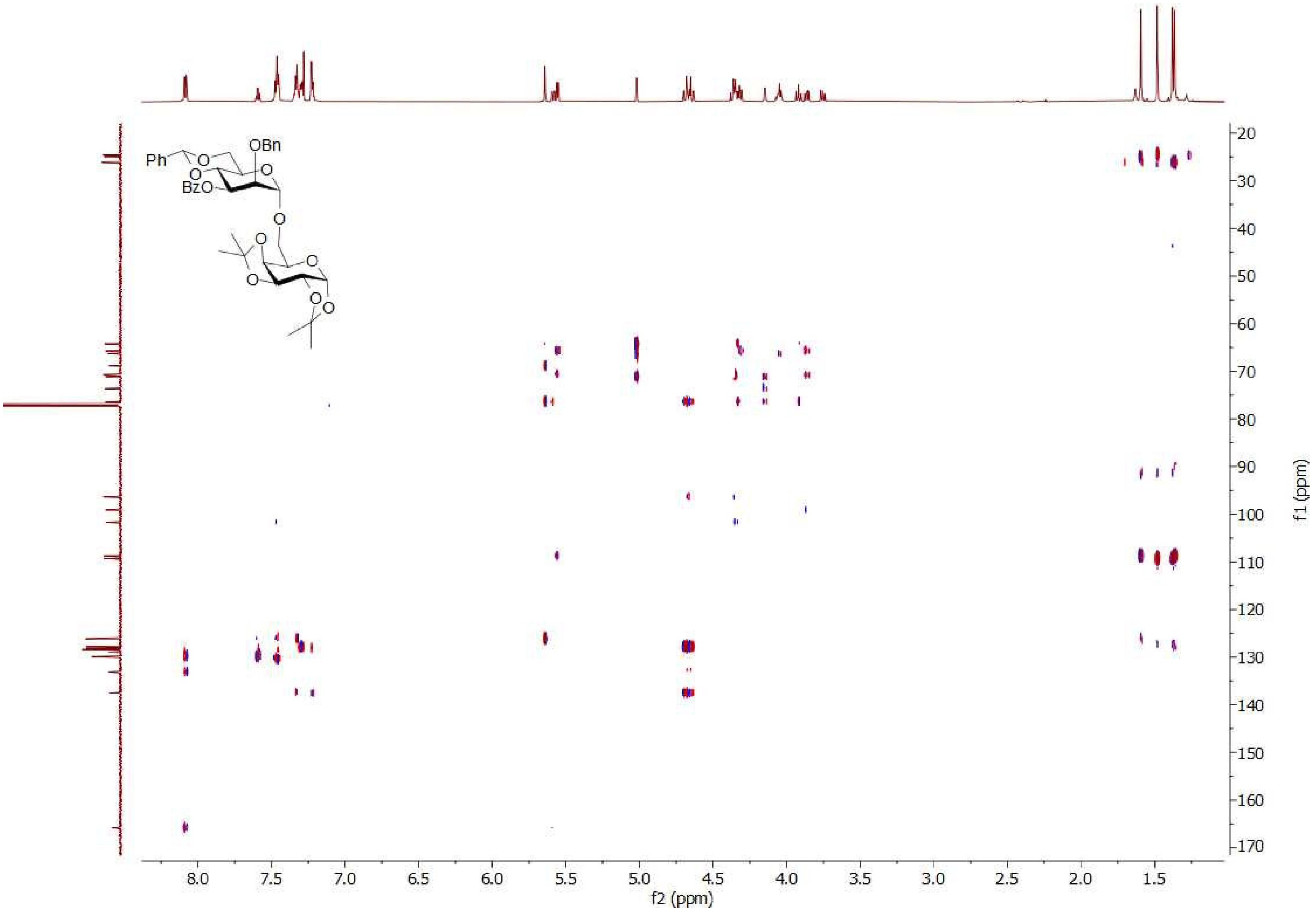

Figure S157. ^{1}H NMR (600 MHz, $CDCl_3$) spectrum of 6-*O*-(3-*O*-benzoyl-2-*O*-benzyl-4,6-*O*-benzylidene-3-*C*-methyl-α-D-mannopyranosyl)-1,2:3,4-di-*O*-isopropylidene-α-D-galactopyranose **53**:

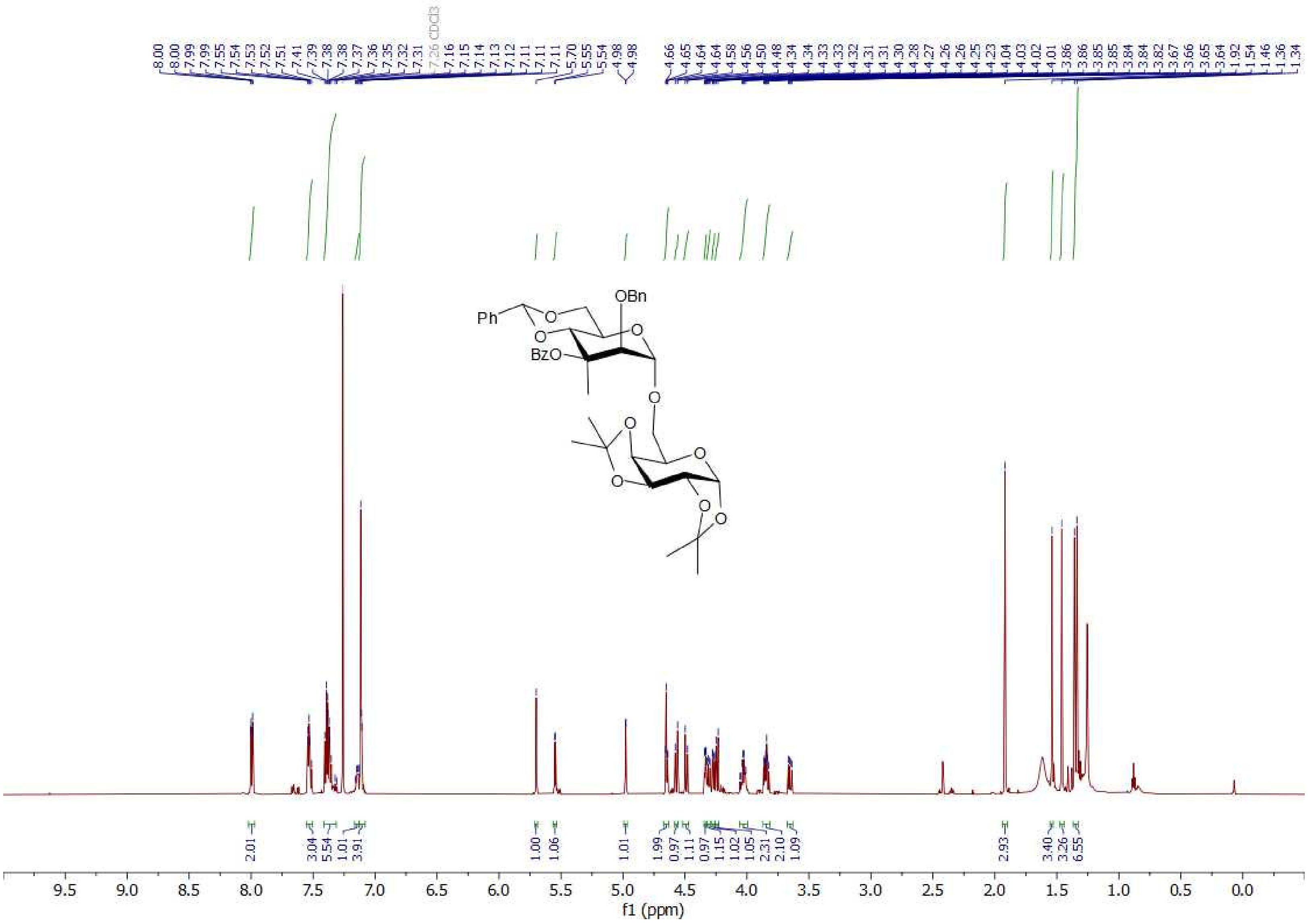

Figure S158. COSY NMR (600 MHz, $CDCl_3$) spectrum of 6-*O*-(3-*O*-benzoyl-2-*O*-benzyl-4,6-*O*-benzylidene-3-*C*-methyl-α-D-mannopyranosyl)-1,2:3,4-di-*O*-isopropylidene-α-D-galactopyranose **53**:

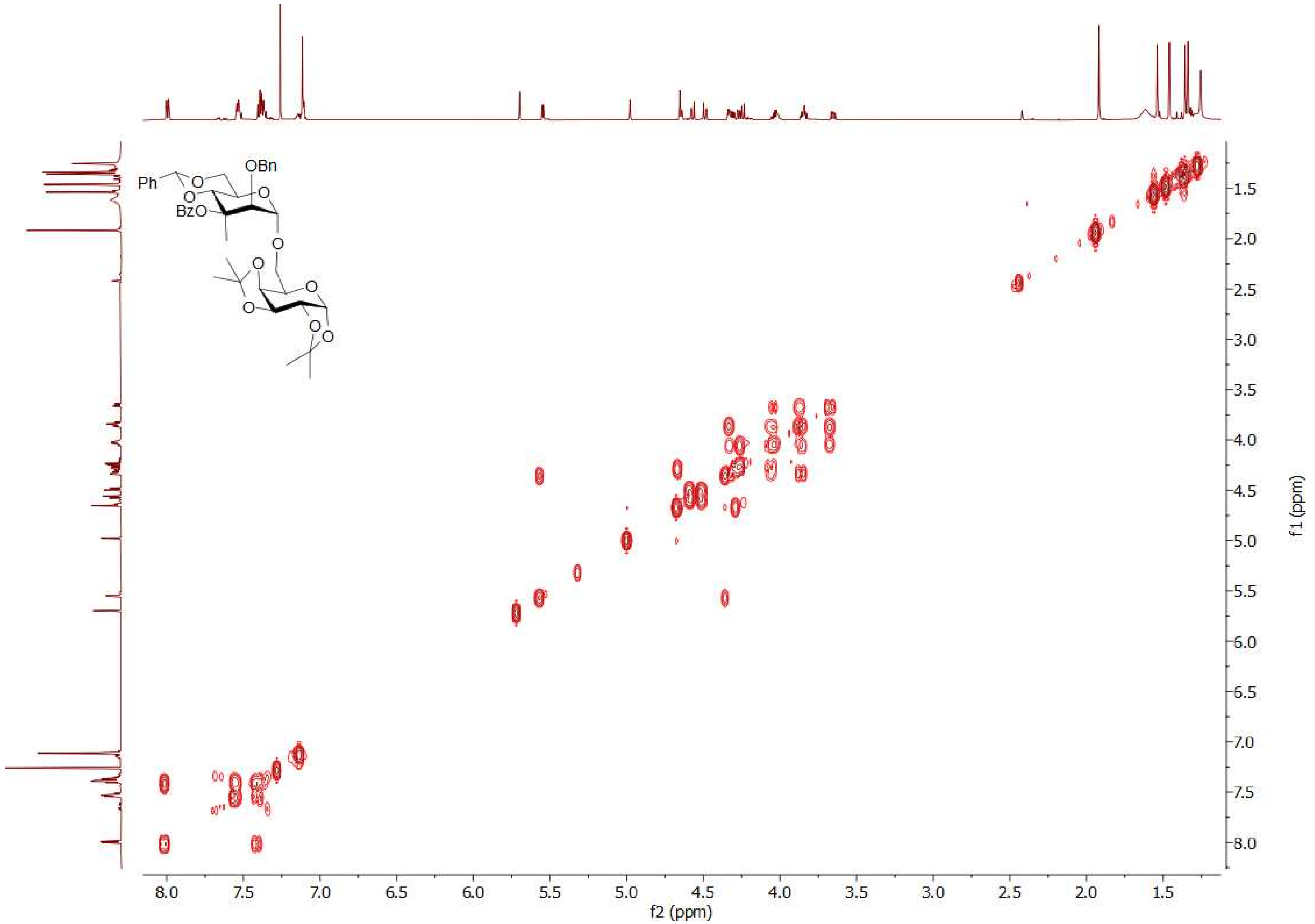

Figure S159. $^{13}C\{^1H\}$ NMR (151 MHz, $CDCl_3$) spectrum of 6-*O*-(3-*O*-benzoyl-2-*O*-benzyl-4,6-*O*-benzylidene-3-*C*-methyl-α-D-mannopyranosyl)-1,2:3,4-di-*O*-isopropylidene-α-D-galactopyranose **53**:

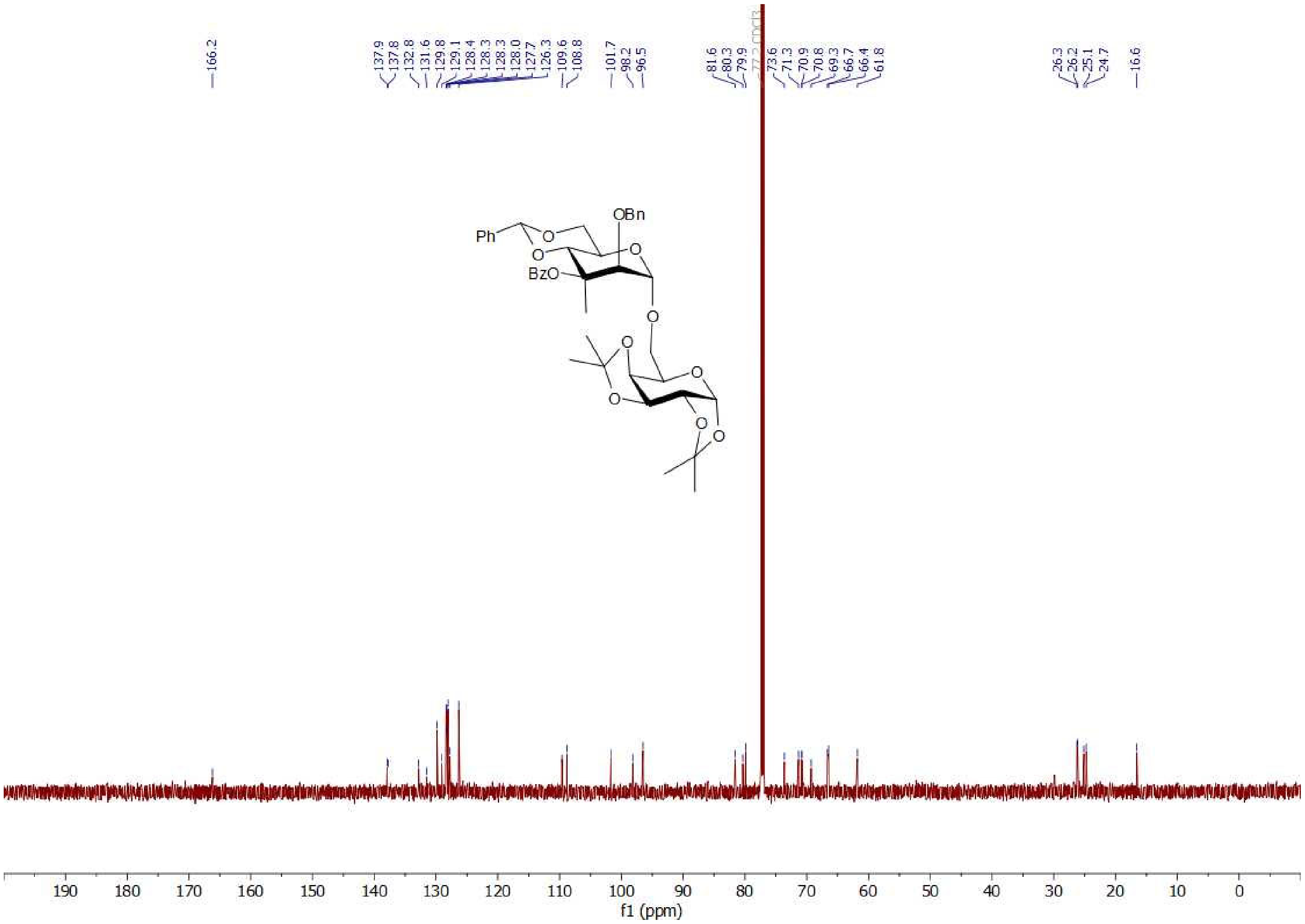

Figure S160. HSQC NMR (600 MHz, $CDCl_3$) spectrum of 6-*O*-(3-*O*-benzoyl-2-*O*-benzyl-4,6-*O*-benzylidene-3-*C*-methyl-α-D-mannopyranosyl)-1,2:3,4-di-*O*-isopropylidene-α-D-galactopyranose **53**:

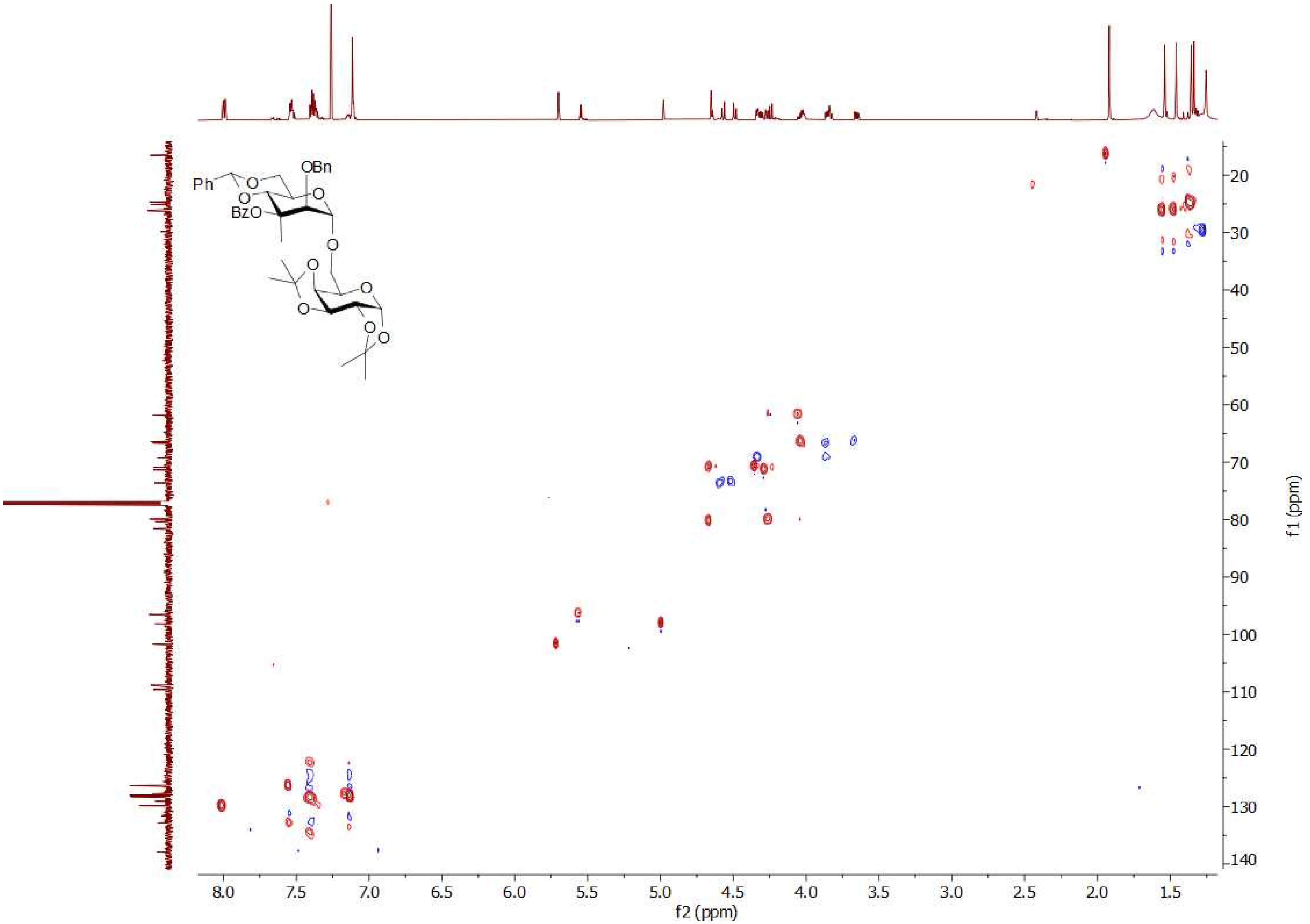

Figure S161. HMBC NMR (600 MHz, $CDCl_3$) spectrum of 6-*O*-(3-*O*-benzoyl-2-*O*-benzyl-4,6-*O*-benzylidene-3-*C*-methyl-α-D-mannopyranosyl)-1,2:3,4-di-*O*-isopropylidene-α-D-galactopyranose **53**:

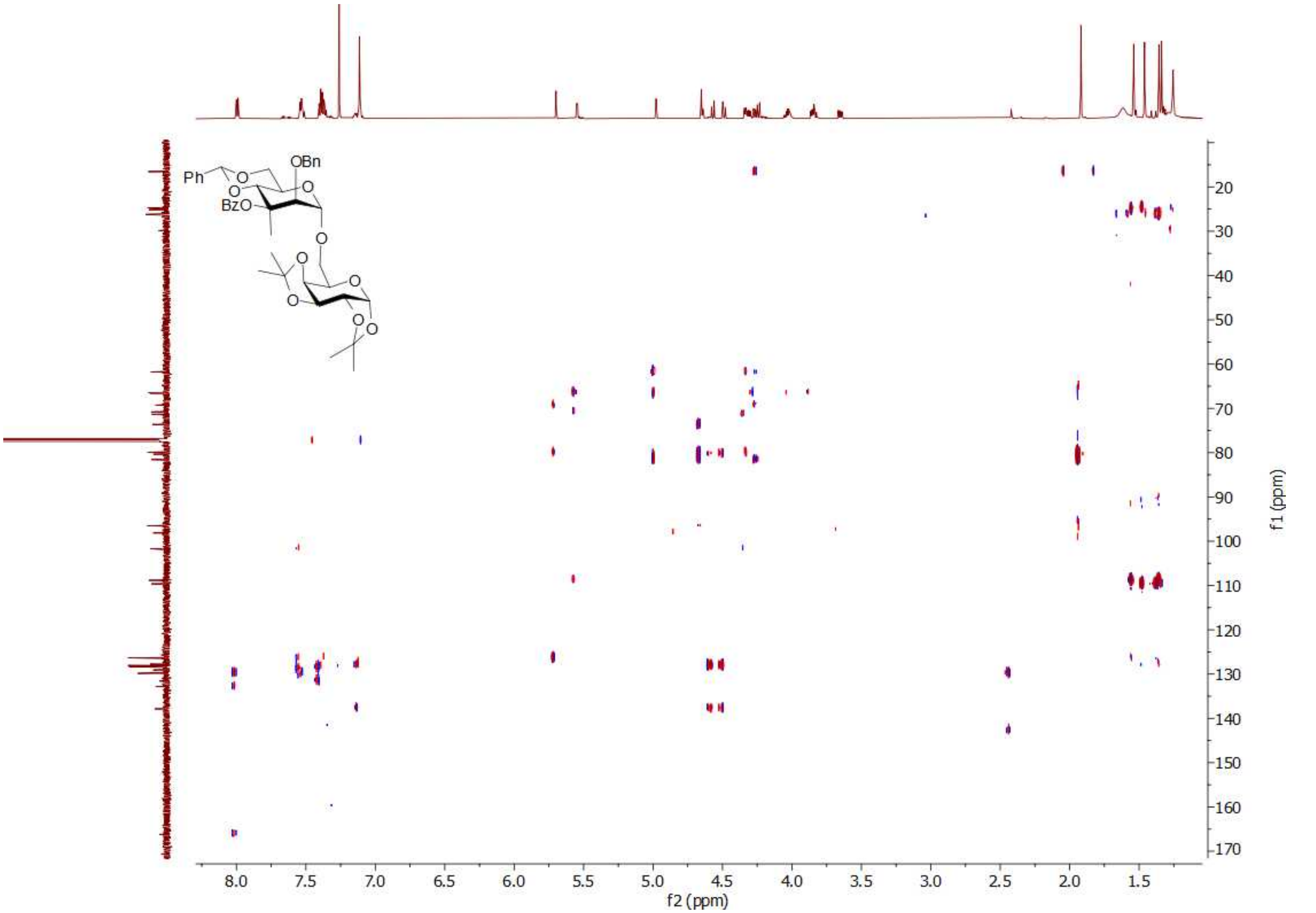

Figure S162. ^{1}H NMR (600 MHz, $CDCl_3$) spectrum of 6-*O*-(2-*O*-benzyl-4,6-*O*-benzylidene-3-*O*-*p*-nitrobenzoyl-3-*C*-methyl-α-D-mannopyranosyl)-1,2:3,4-di-*O*-isopropylidene-α-D-galactopyranose **54**:

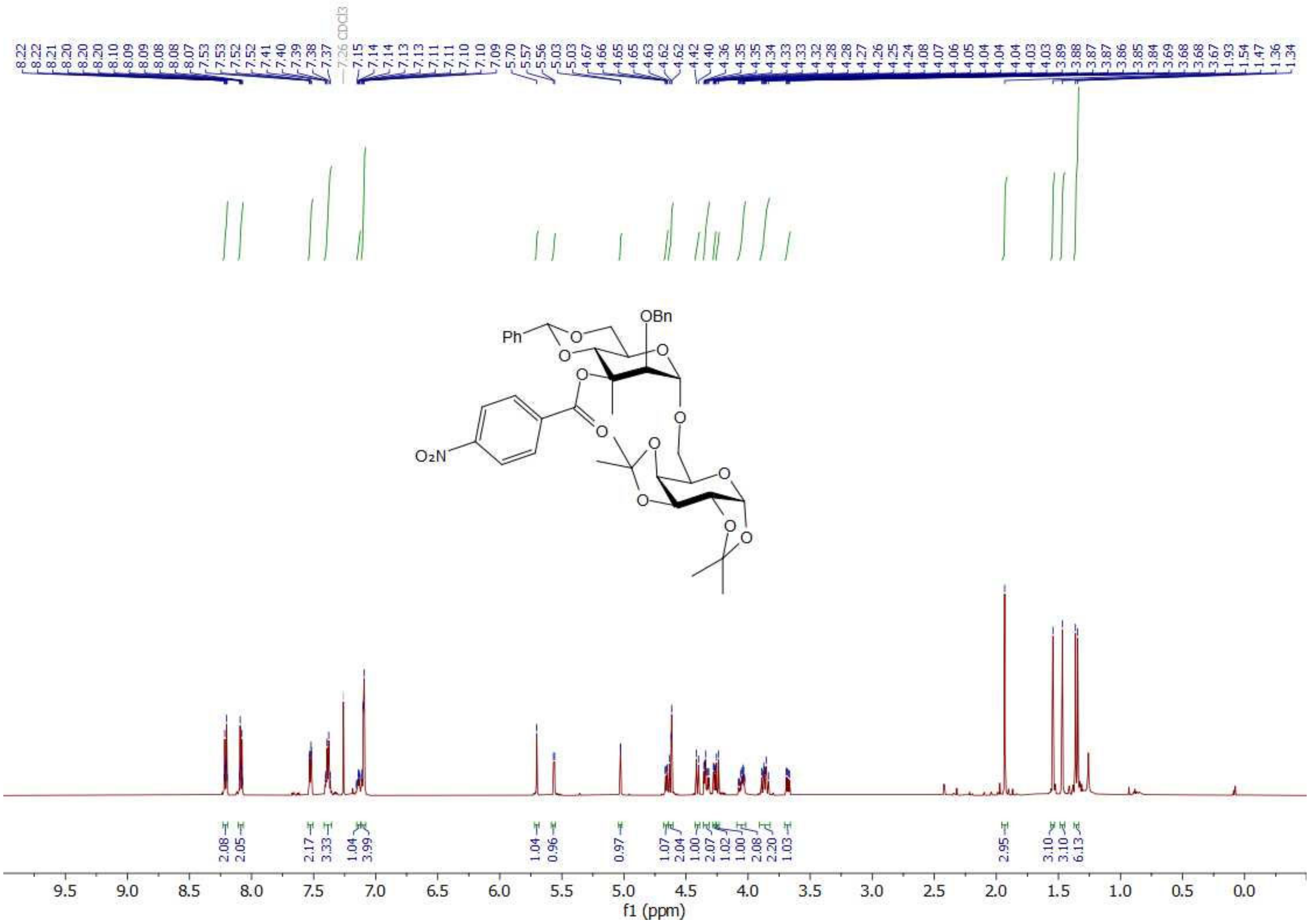

Figure S163. COSY (600 MHz, $CDCl_3$) spectrum of 6-*O*-(2-*O*-benzyl-4,6-*O*-benzylidene-3-*O*-*p*-nitrobenzoyl-3-*C*-methyl-α-D-mannopyranosyl)-1,2:3,4-di-*O*-isopropylidene-α-D-galactopyranose **54**:

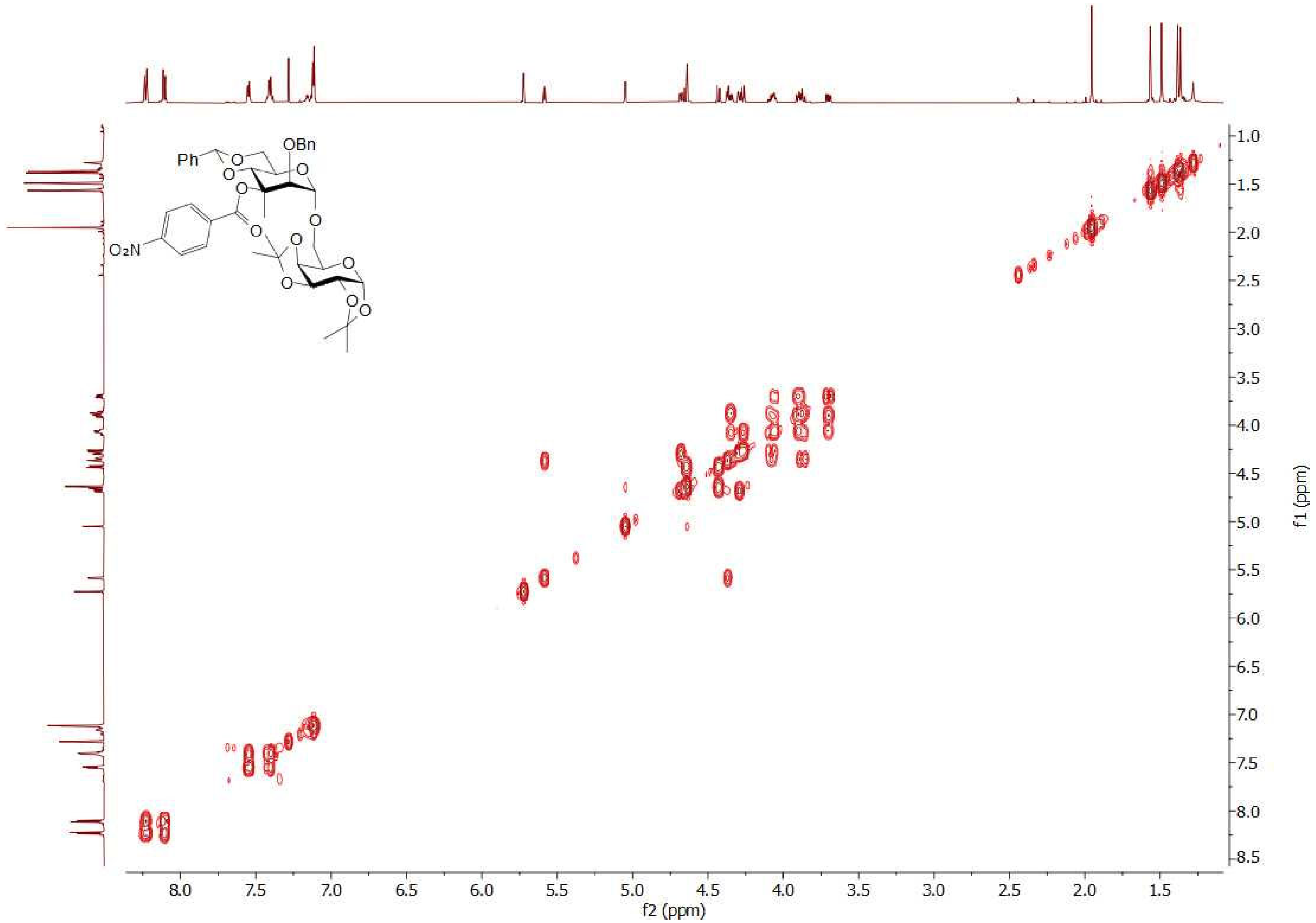

Figure S164. $^{13}C\{^{1}H\}$ NMR (151 MHz, $CDCl_3$) spectrum of 6-*O*-(2-*O*-benzyl-4,6-*O*-benzylidene-3-*O*-*p*-nitrobenzoyl-3-*C*-methyl-α-D-mannopyranosyl)-1,2:3,4-di-*O*-isopropylidene-α-D-galactopyranose **54**:

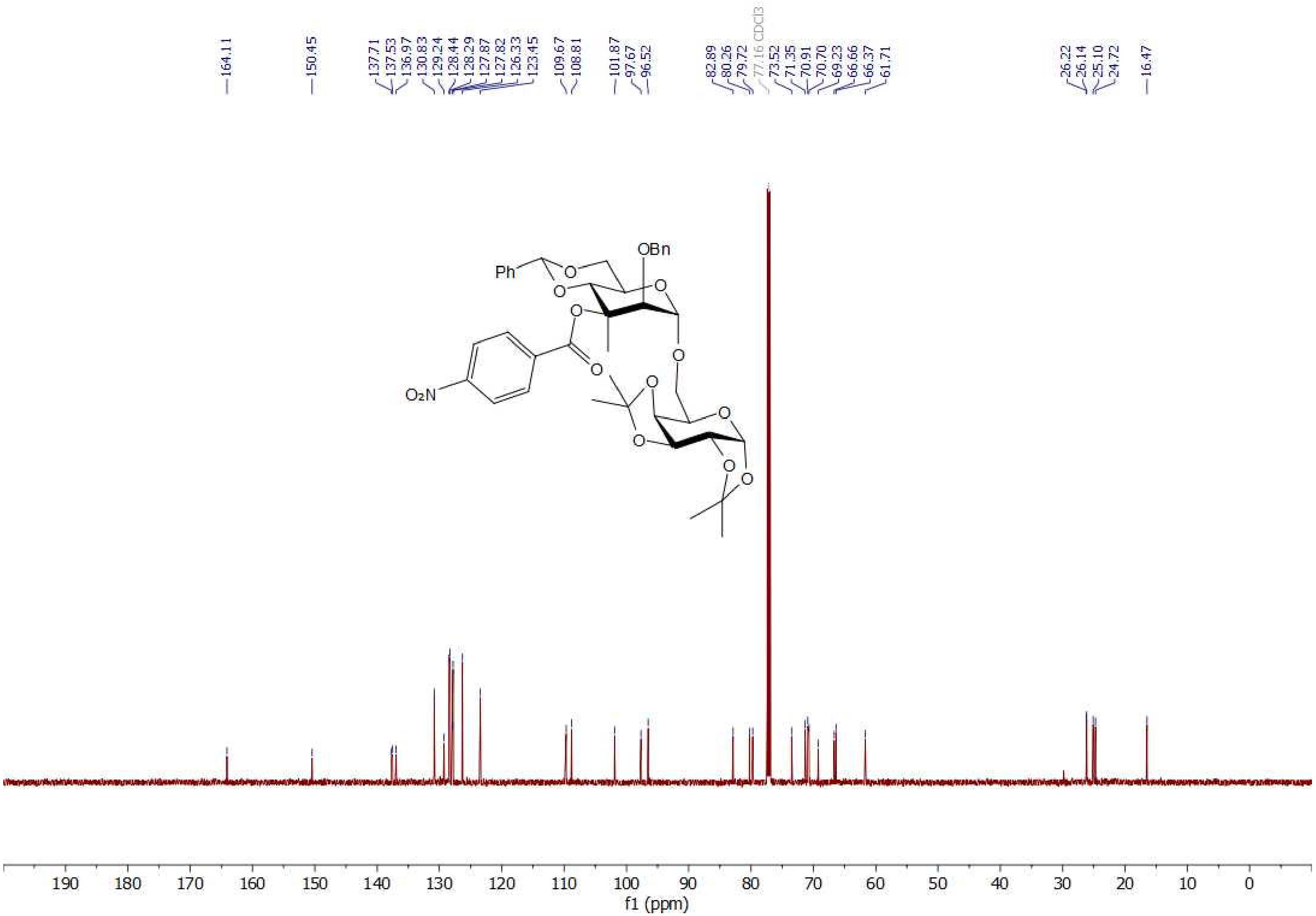

Figure S165. $^{13}C\{^1H\}$ DEPT NMR (151 MHz, $CDCl_3$) spectrum of 6-*O*-(2-*O*-benzyl-4,6-*O*-benzylidene-3-*O*-*p*-nitrobenzoyl-3-*C*-methyl-α-D-mannopyranosyl)-1,2:3,4-di-*O*-isopropylidene-α-D-galactopyranose **54**:

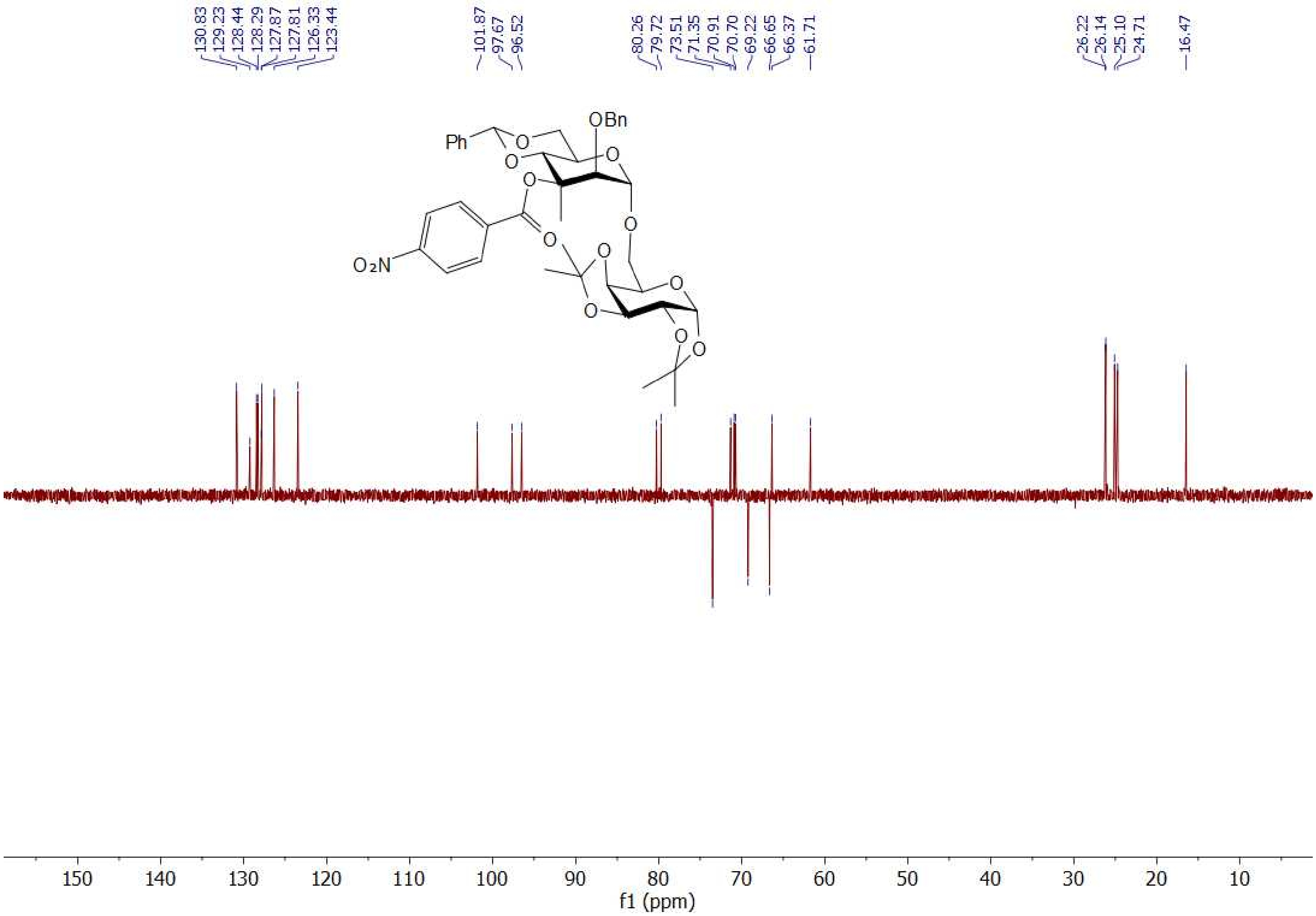

Figure S166. HSQC NMR (600 MHz, $CDCl_3$) spectrum of 6-*O*-(2-*O*-benzyl-4,6-*O*-benzylidene-3-*O*-*p*-nitrobenzoyl-3-*C*-methyl-α-D-mannopyranosyl)-1,2:3,4-di-*O*-isopropylidene-α-D-galactopyranose **54**:

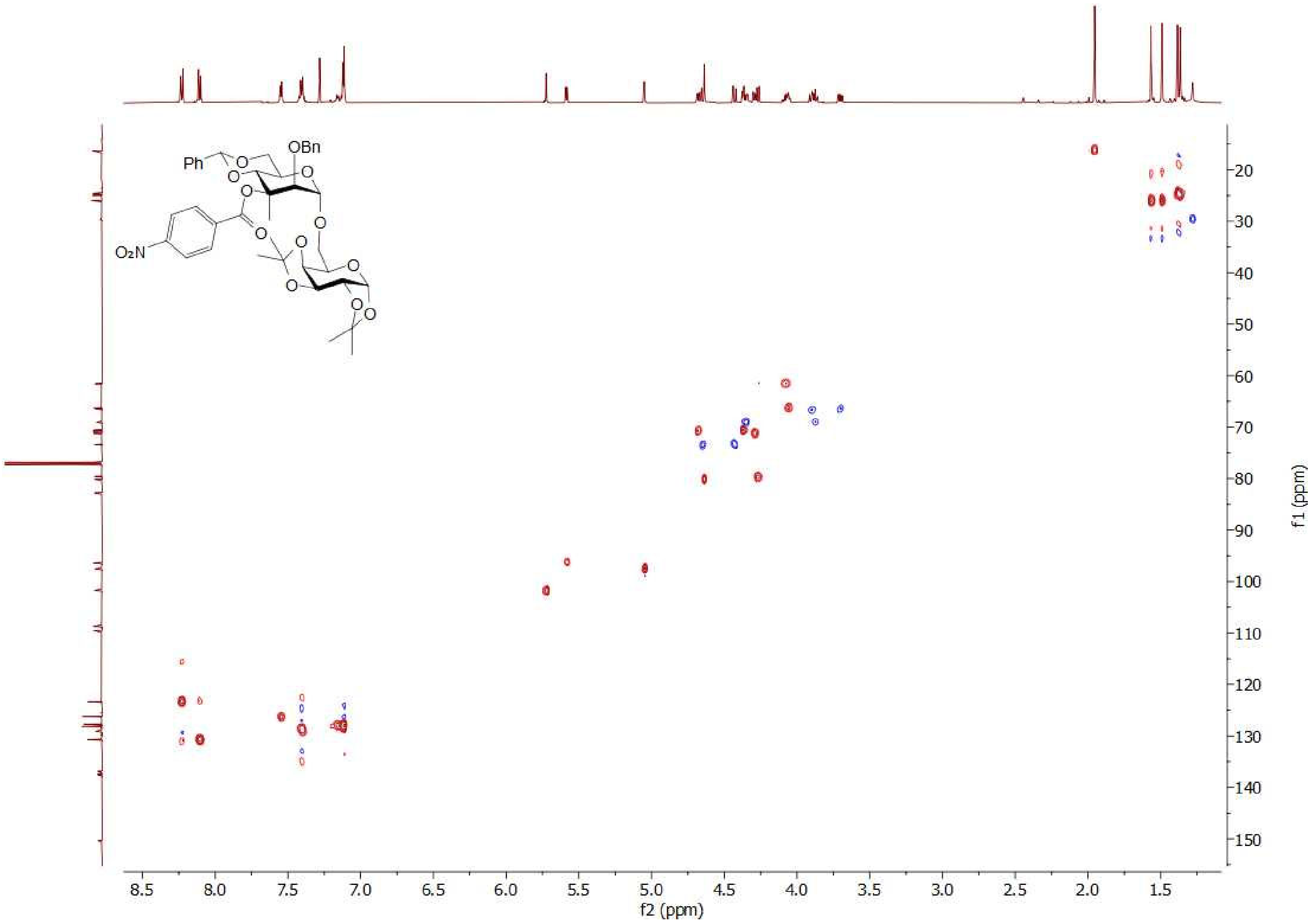

Figure S167. HMBC NMR (600 MHz, $CDCl_3$) spectrum of 6-*O*-(2-*O*-benzyl-4,6-*O*-benzylidene-3-*O*-*p*-nitrobenzoyl-3-*C*-methyl-α-D-mannopyranosyl)-1,2:3,4-di-*O*-isopropylidene-α-D-galactopyranose **54**:

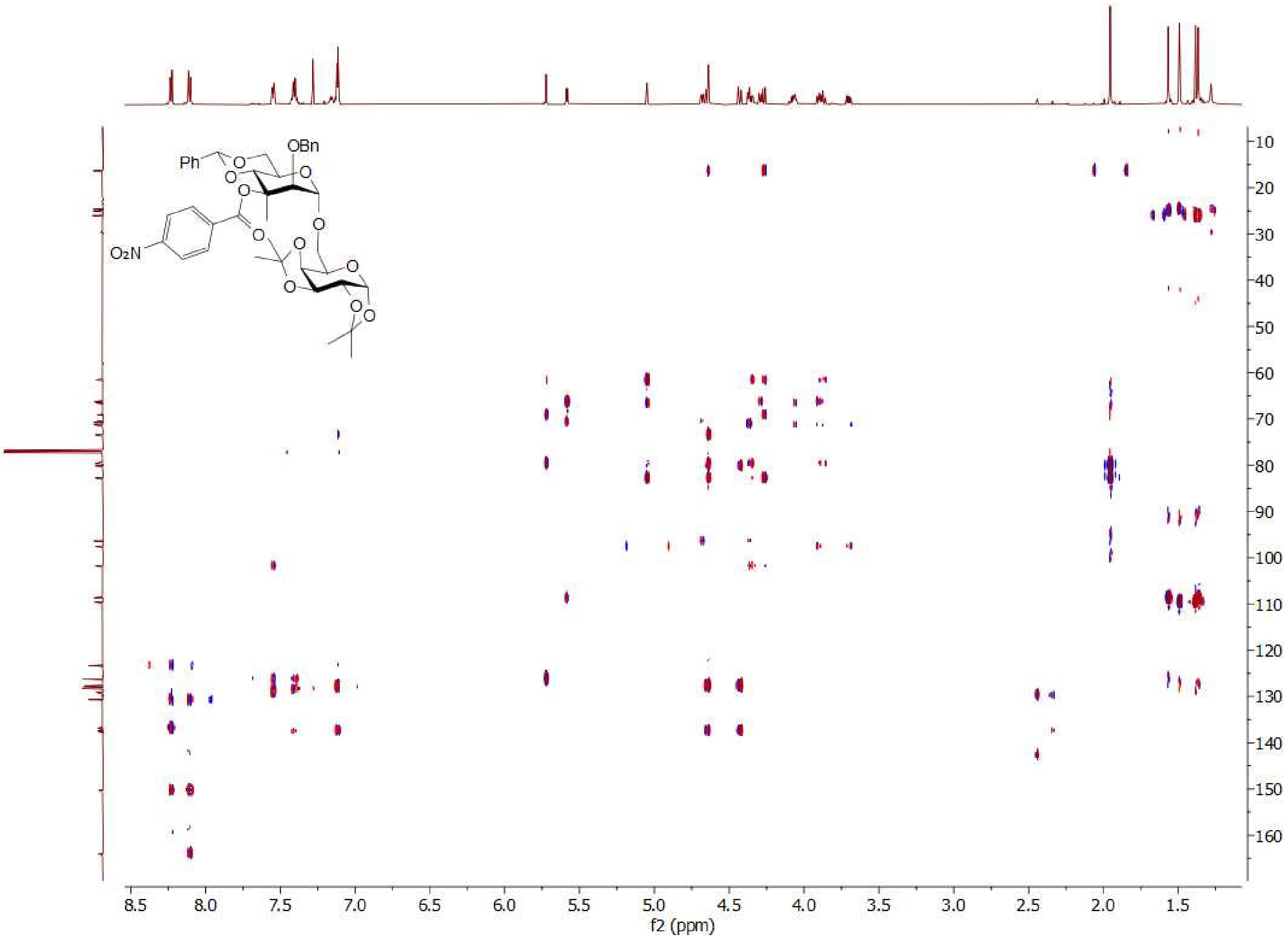

Figure S168. ^{1}H NMR (600 MHz, $CDCl_3$) spectrum of 3-*O*-(3-*O*-benzoyl-2-*O*-benzyl-4,6-*O*-benzylidene-α-D-mannopyranosyl)-1,2:5,6-di-*O*-isopropylidene-α-D-glucofuranose **55**:

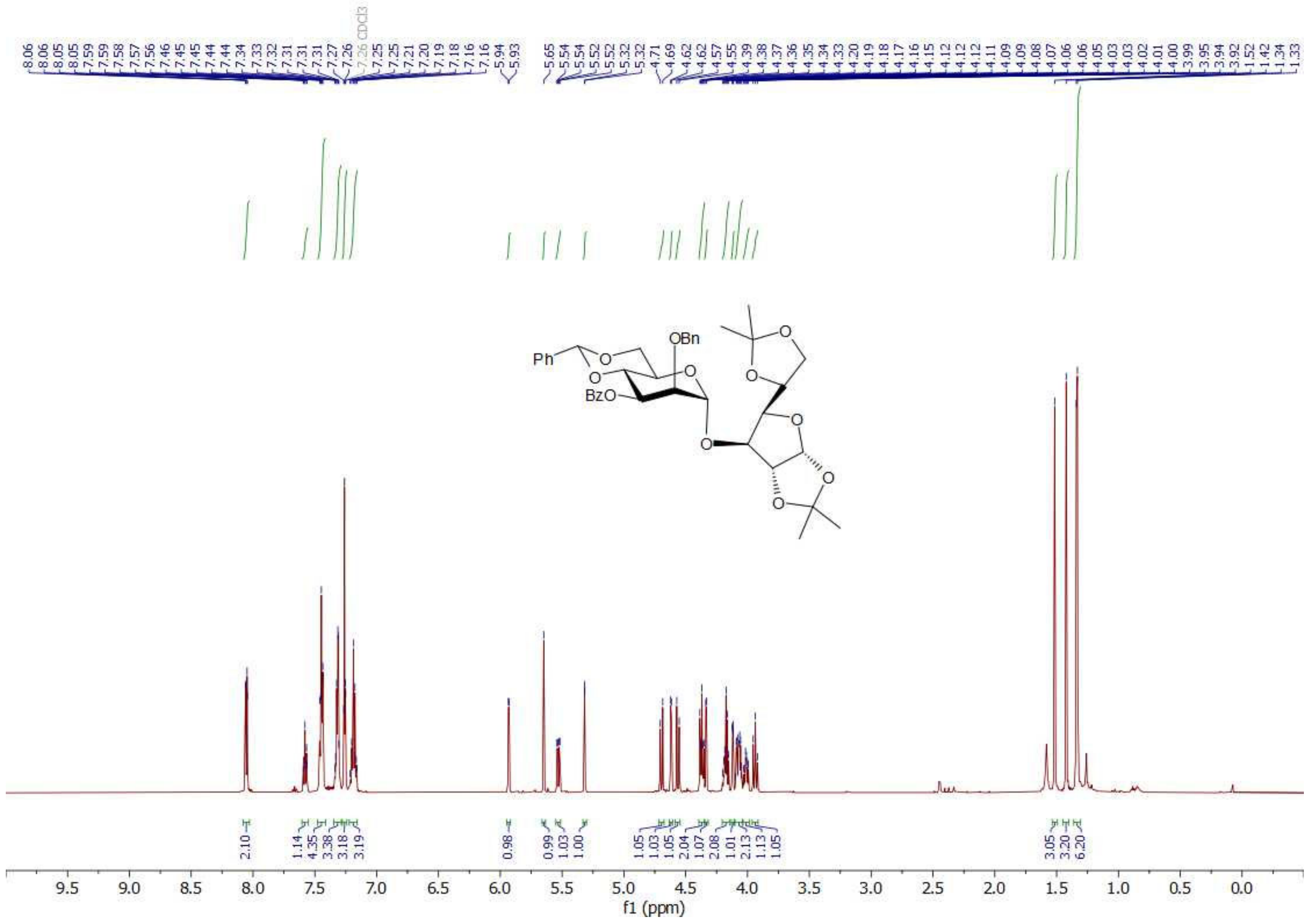

Figure S169. COSY NMR (600 MHz, $CDCl_3$) spectrum of 3-*O*-(3-*O*-benzoyl-2-*O*-benzyl-4,6-*O*-benzylidene-α-D-mannopyranosyl)-1,2:5,6-di-*O*-isopropylidene-α-D-glucofuranose **55**:

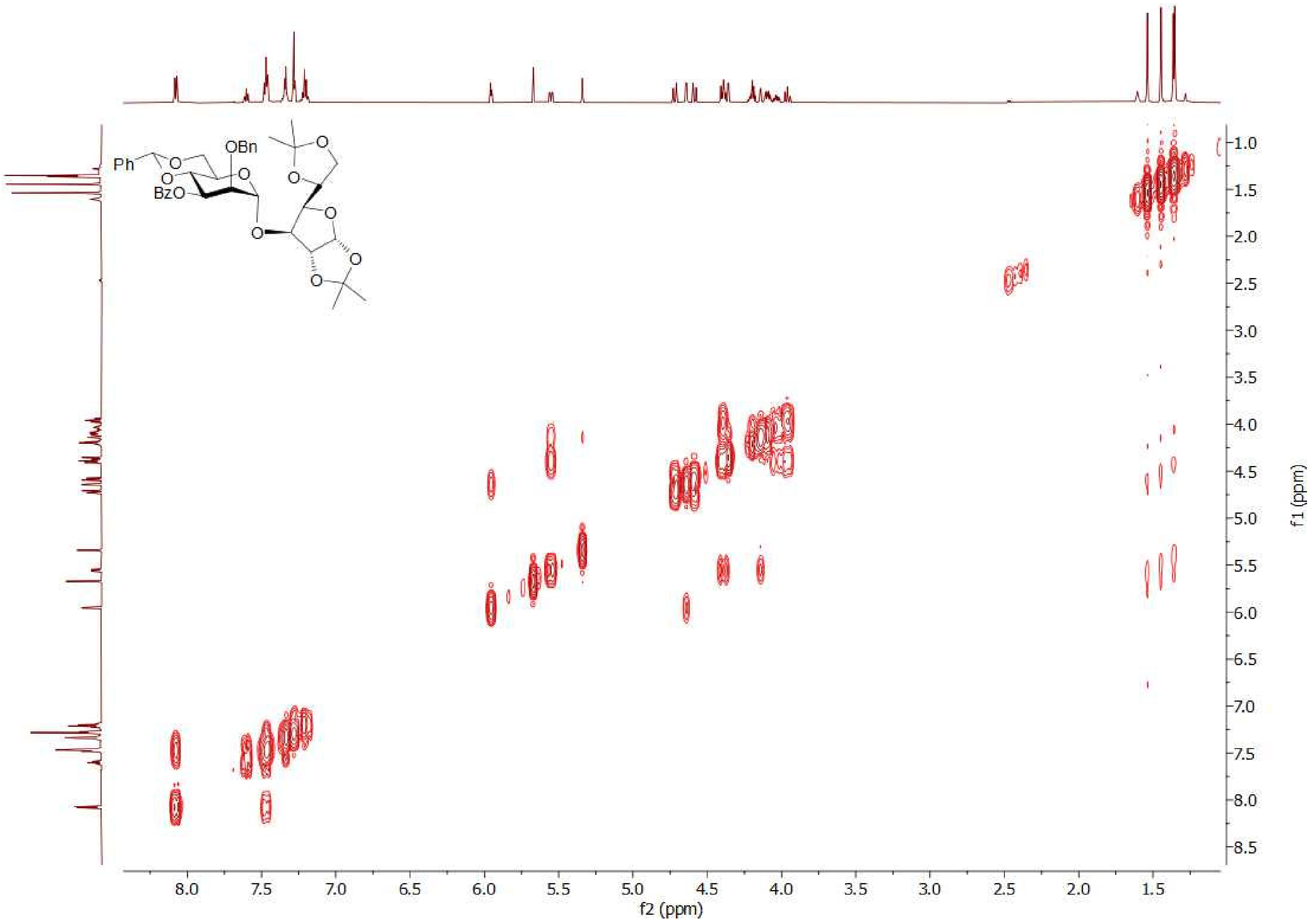

Figure S170. $^{13}C\{^{1}H\}$ NMR (151 MHz, $CDCl_3$) spectrum of 3-*O*-(3-*O*-benzoyl-2-*O*-benzyl-4,6-*O*-benzylidene-α-D-mannopyranosyl)-1,2:5,6-di-*O*-isopropylidene-α-D-glucofuranose **55**:

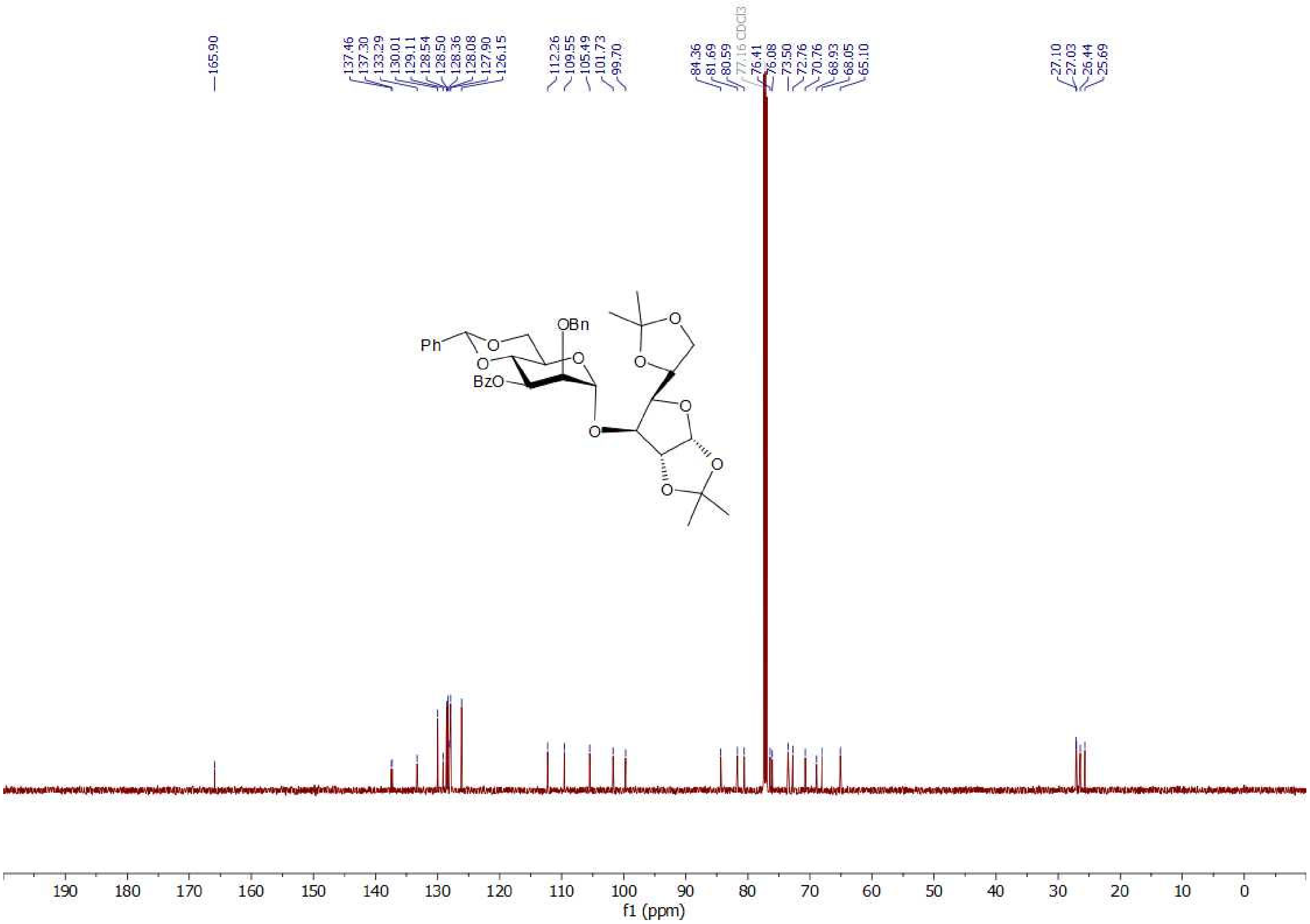

Figure S171. $^{13}C\{^{1}H\}$ DEPT NMR (151 MHz, $CDCl_3$) spectrum of 3-*O*-(3-*O*-benzoyl-2-*O*-benzyl-4,6-*O*-benzylidene-α-D-mannopyranosyl)-1,2:5,6-di-*O*-isopropylidene-α-D-glucofuranose **55**:

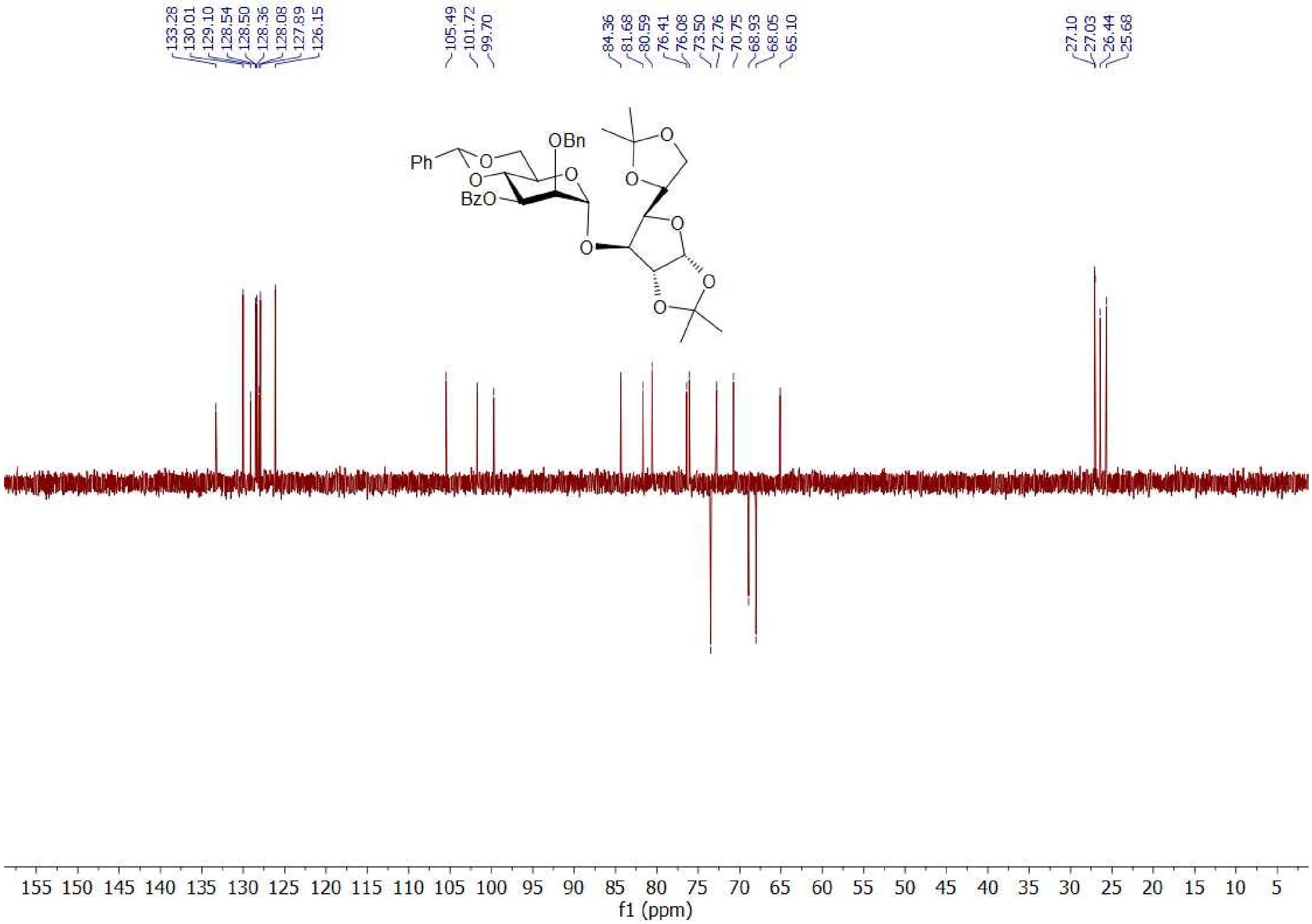

Figure S172. HSQC NMR (600 MHz, $CDCl_3$) spectrum of 3-*O*-(3-*O*-benzoyl-2-*O*-benzyl-4,6-*O*-benzylidene-α-D-mannopyranosyl)-1,2:5,6-di-*O*-isopropylidene-α-D-glucofuranose **55**:

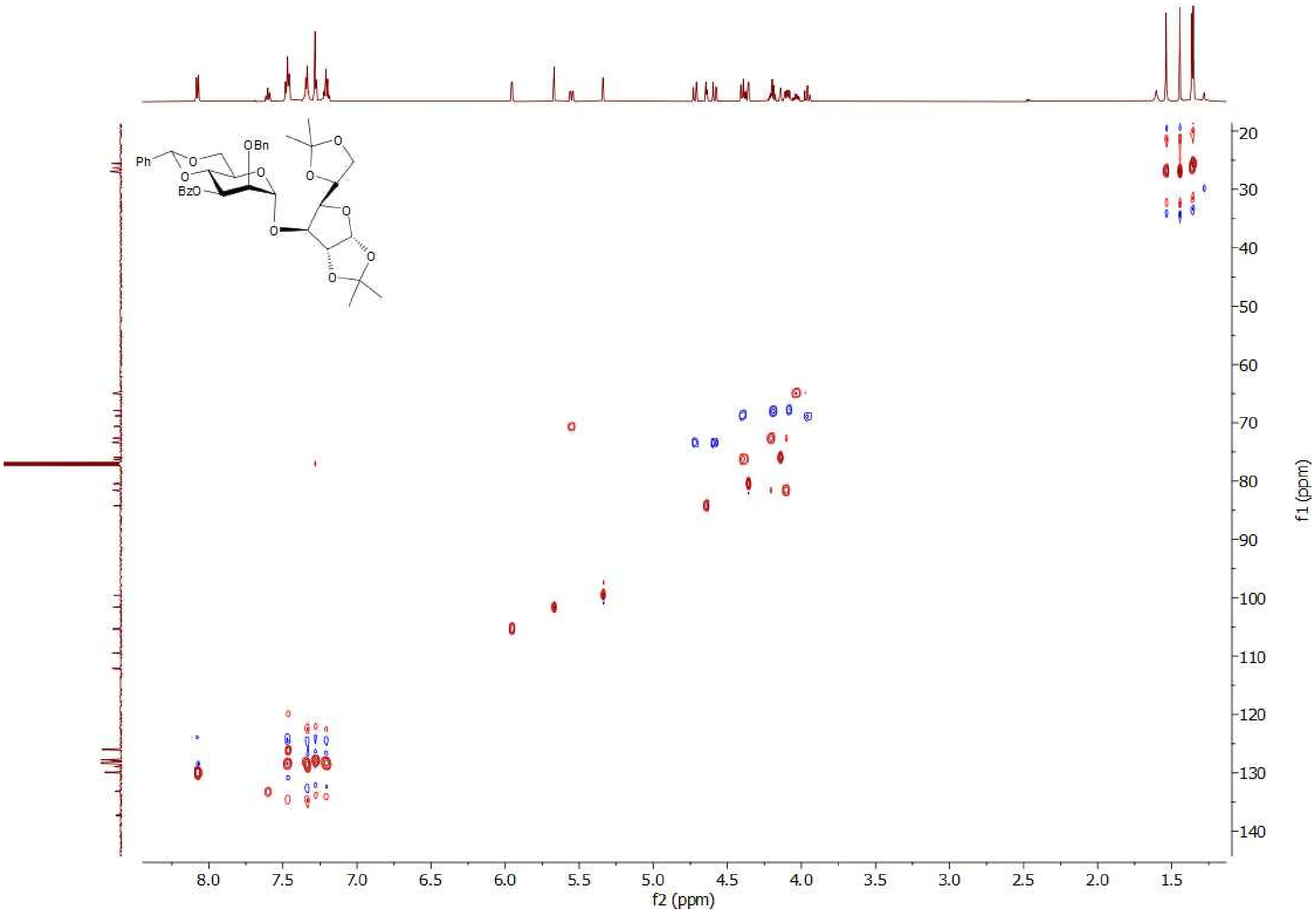

Figure S173. HMBC NMR (600 MHz, $CDCl_3$) spectrum of 3-*O*-(3-*O*-benzoyl-2-*O*-benzyl-4,6-*O*-benzylidene-α-D-mannopyranosyl)-1,2:5,6-di-*O*-isopropylidene-α-D-glucofuranose **55**:

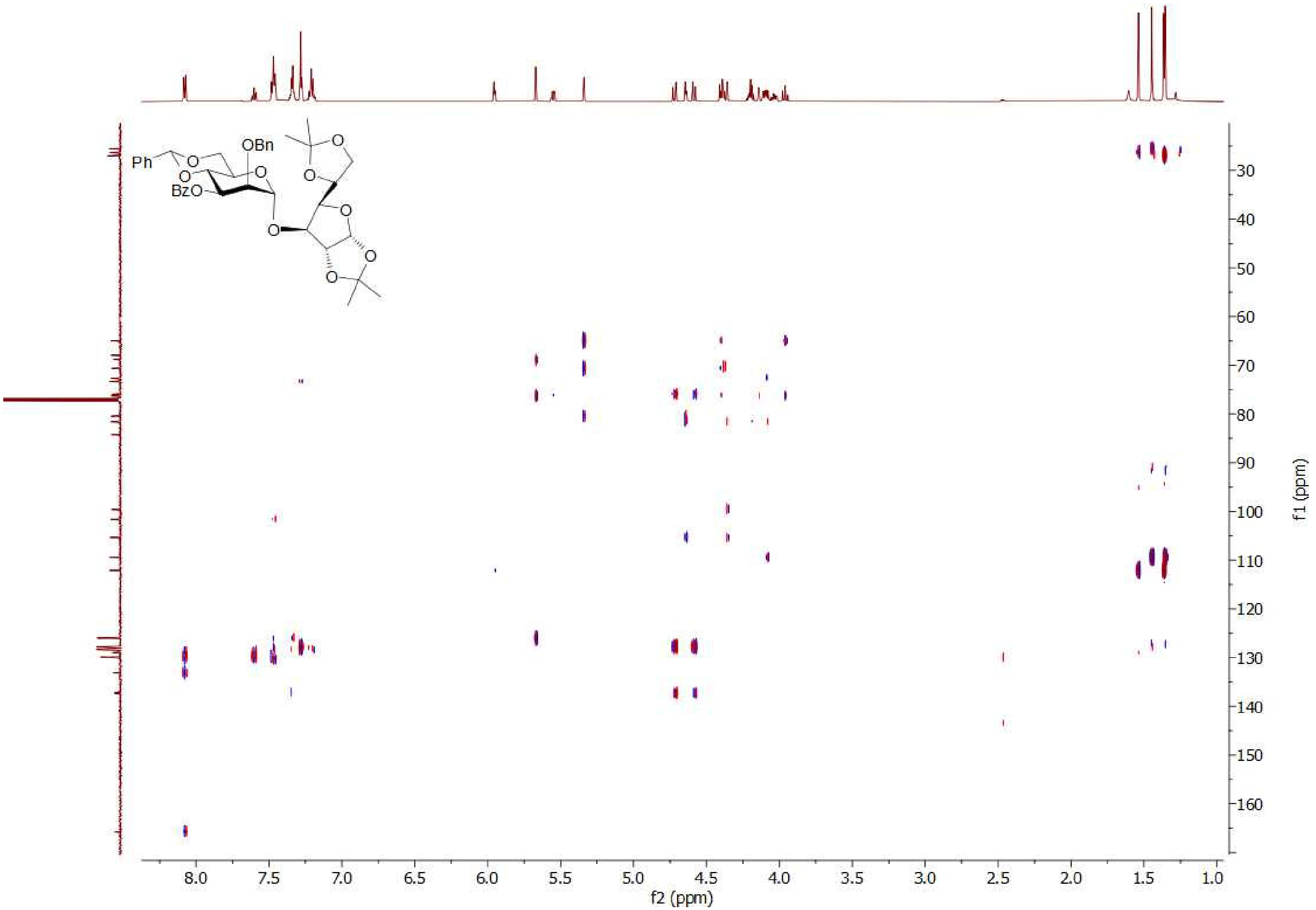

Figure S174. ^{1}H NMR (600 MHz, $CDCl_3$) spectrum of 3-*O*-(3-*O*-benzoyl-2-*O*-benzyl-4,6-*O*-benzylidene-3-*C*-methyl-α-D-mannopyranosyl)-1,2:5,6-di-*O*-isopropylidene-α-D-glucofuranose **56**:

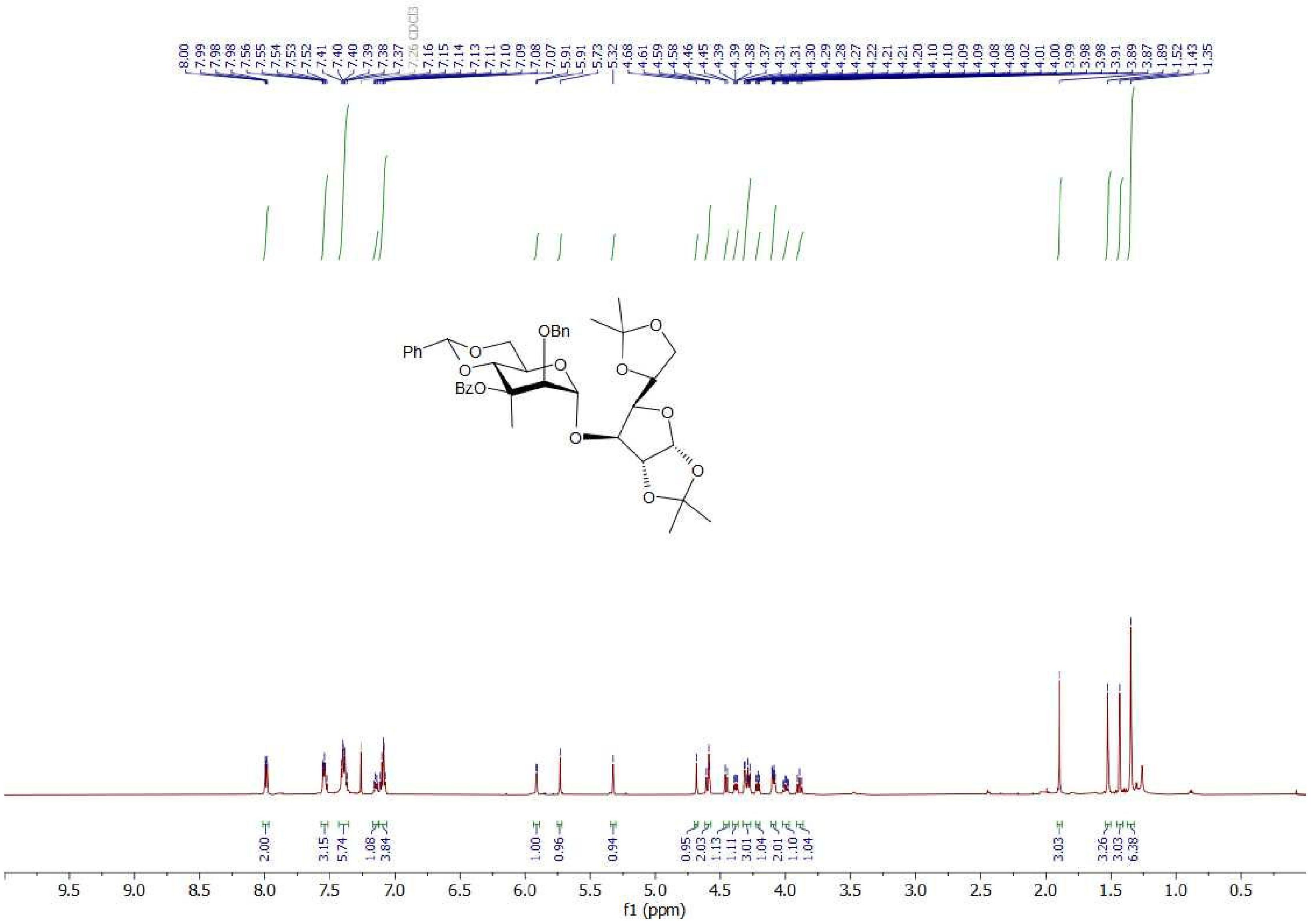

Figure S175. COSY NMR (600 MHz, $CDCl_3$) spectrum of 3-*O*-(3-*O*-benzoyl-2-*O*-benzyl-4,6-*O*-benzylidene-3-*C*-methyl-α-D-mannopyranosyl)-1,2:5,6-di-*O*-isopropylidene-α-D-glucofuranose **56**:

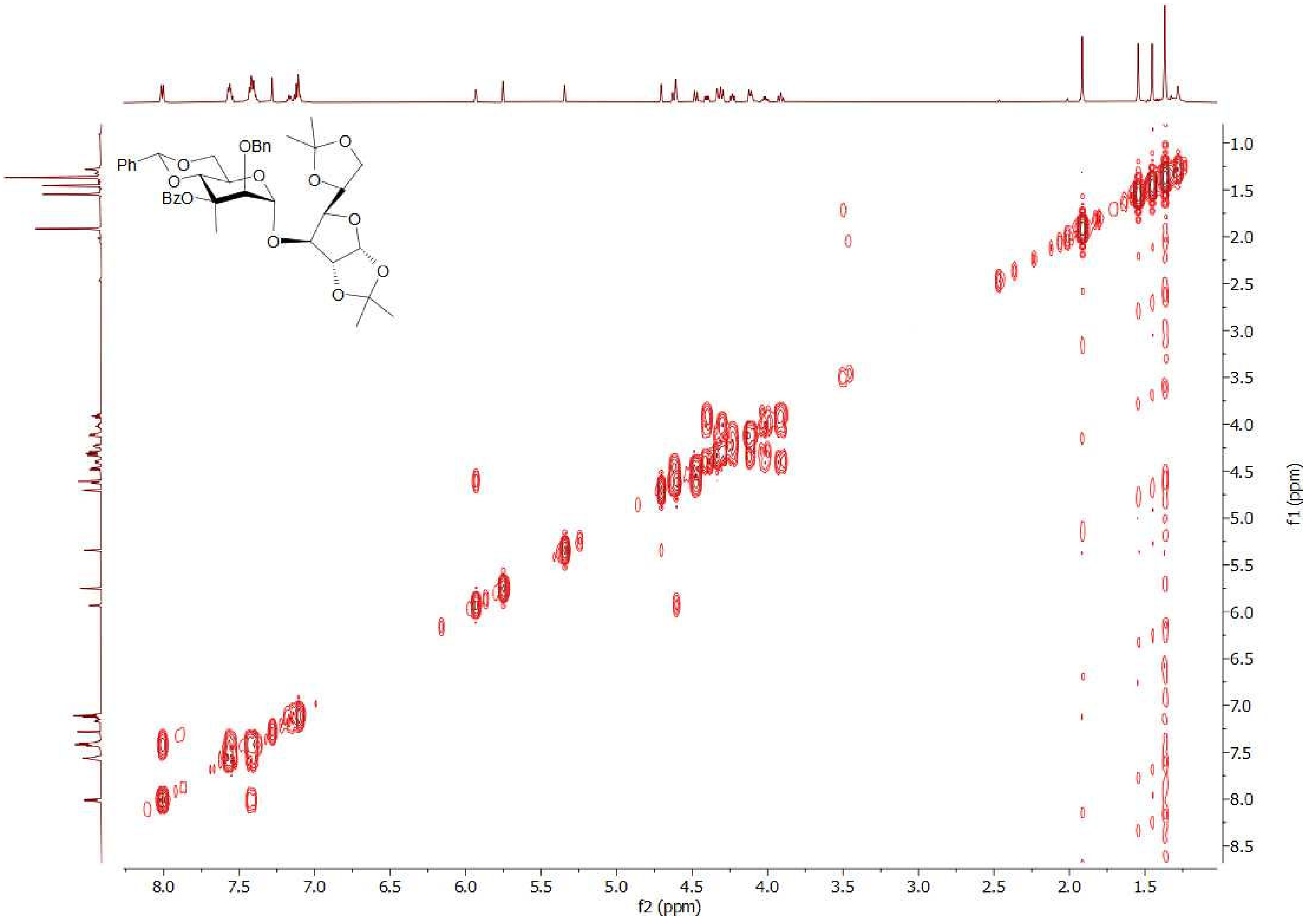

Figure S176. $^{13}C\{^{1}H\}$ NMR (151 MHz, $CDCl_3$) spectrum of 3-*O*-(3-*O*-benzoyl-2-*O*-benzyl-4,6-*O*-benzylidene-3-*C*-methyl-α-D-mannopyranosyl)-1,2:5,6-di-*O*-isopropylidene-α-D-glucofuranose **56**:

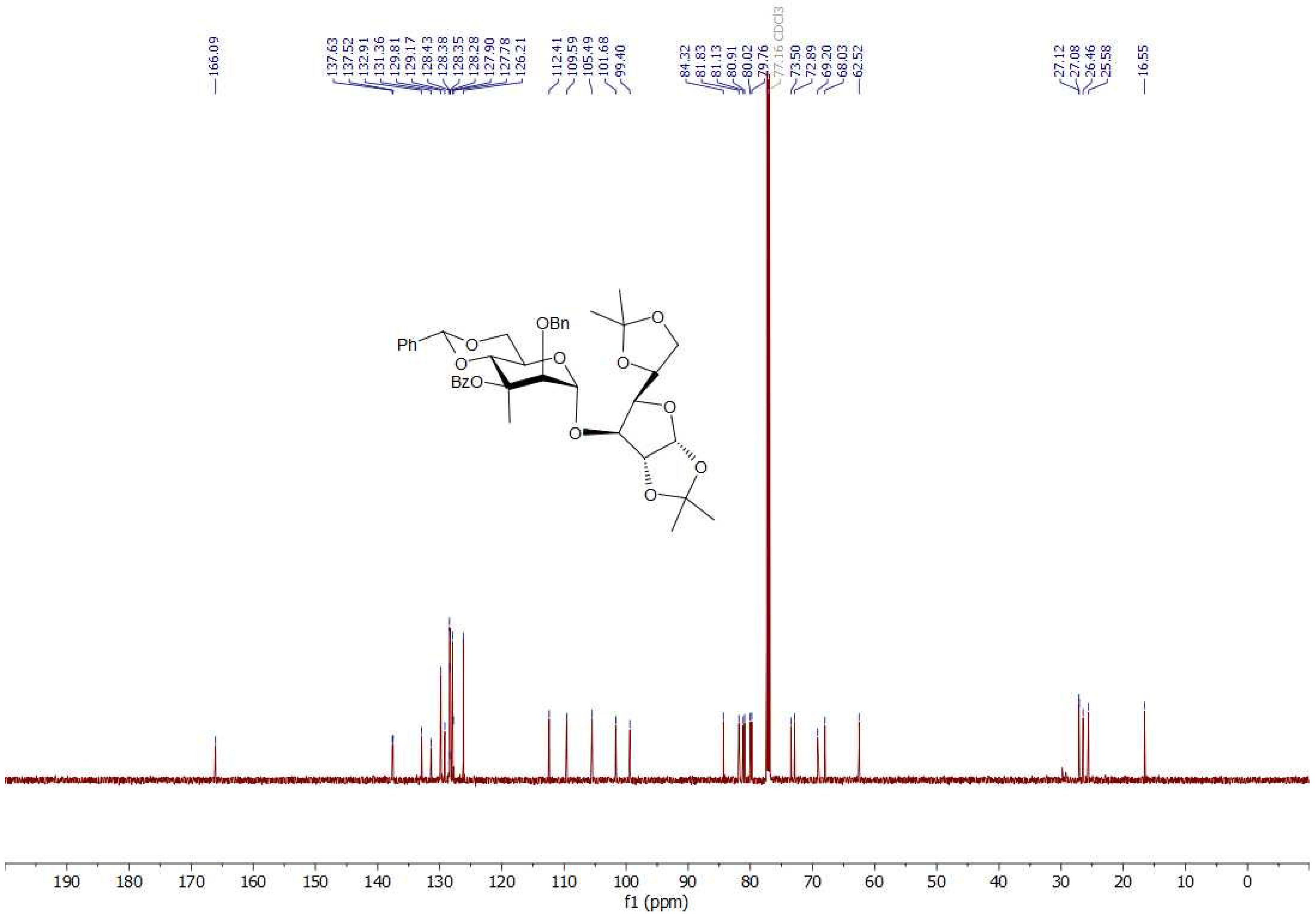

Figure S177. $^{13}C\{^{1}H\}$ DEPT NMR (151 MHz, $CDCl_3$) spectrum of 3-*O*-(3-*O*-benzoyl-2-*O*-benzyl-4,6-*O*-benzylidene-3-*C*-methyl-α-D-mannopyranosyl)-1,2:5,6-di-*O*-isopropylidene-α-D-glucofuranose **56**:

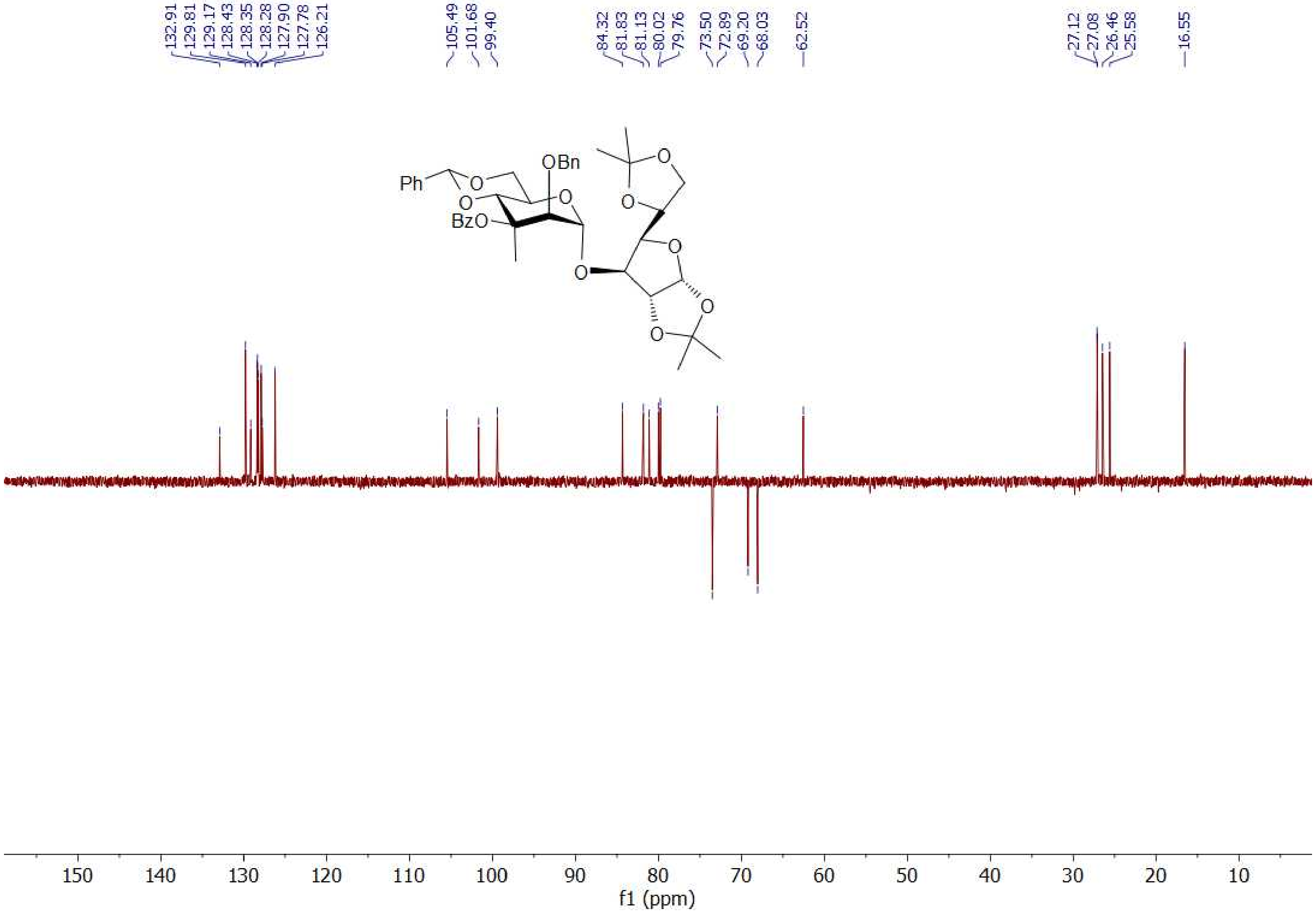

Figure S178. HSQC NMR (600 MHz, $CDCl_3$) spectrum of 3-*O*-(3-*O*-benzoyl-2-*O*-benzyl-4,6-*O*-benzylidene-3-*C*-methyl-α-D-mannopyranosyl)-1,2:5,6-di-*O*-isopropylidene-α-D-glucofuranose **56**:

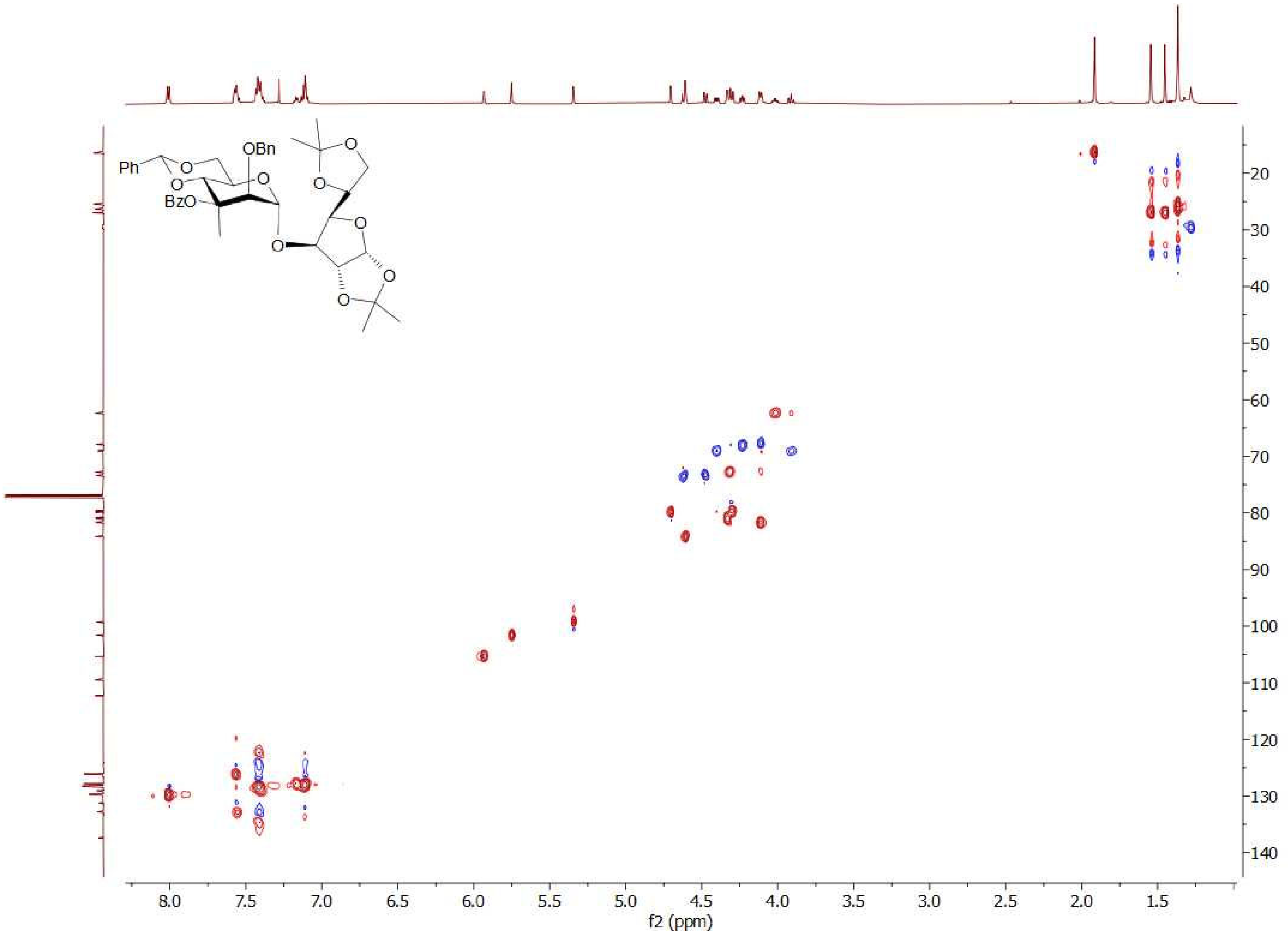

Figure S179. HMBC NMR (600 MHz, $CDCl_3$) spectrum of 3-*O*-(3-*O*-benzoyl-2-*O*-benzyl-4,6-*O*-benzylidene-3-*C*-methyl-α-D-mannopyranosyl)-1,2:5,6-di-*O*-isopropylidene-α-D-glucofuranose **56**:

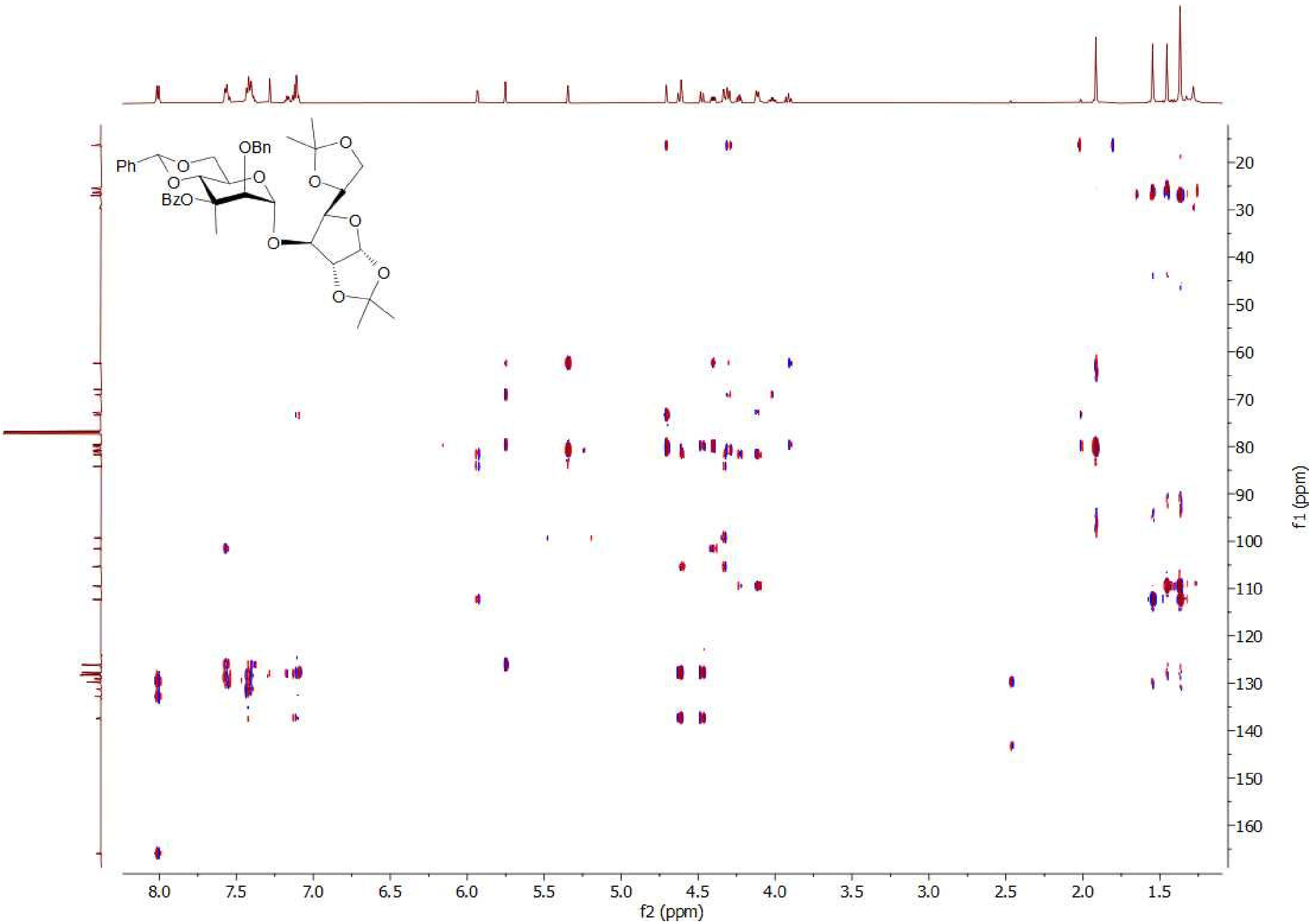

Figure S180. ^{1}H NMR (600 MHz, $CDCl_3$) spectrum of 3-*O*-(2-*O*-benzyl-4,6-*O*-benzylidene-3-*O*-*p*-nitrobenzoyl-3-*C*-methyl-α-D-mannopyranosyl)-1,2:5,6-di-*O*-isopropylidene-α-D-glucofuranose **57**:

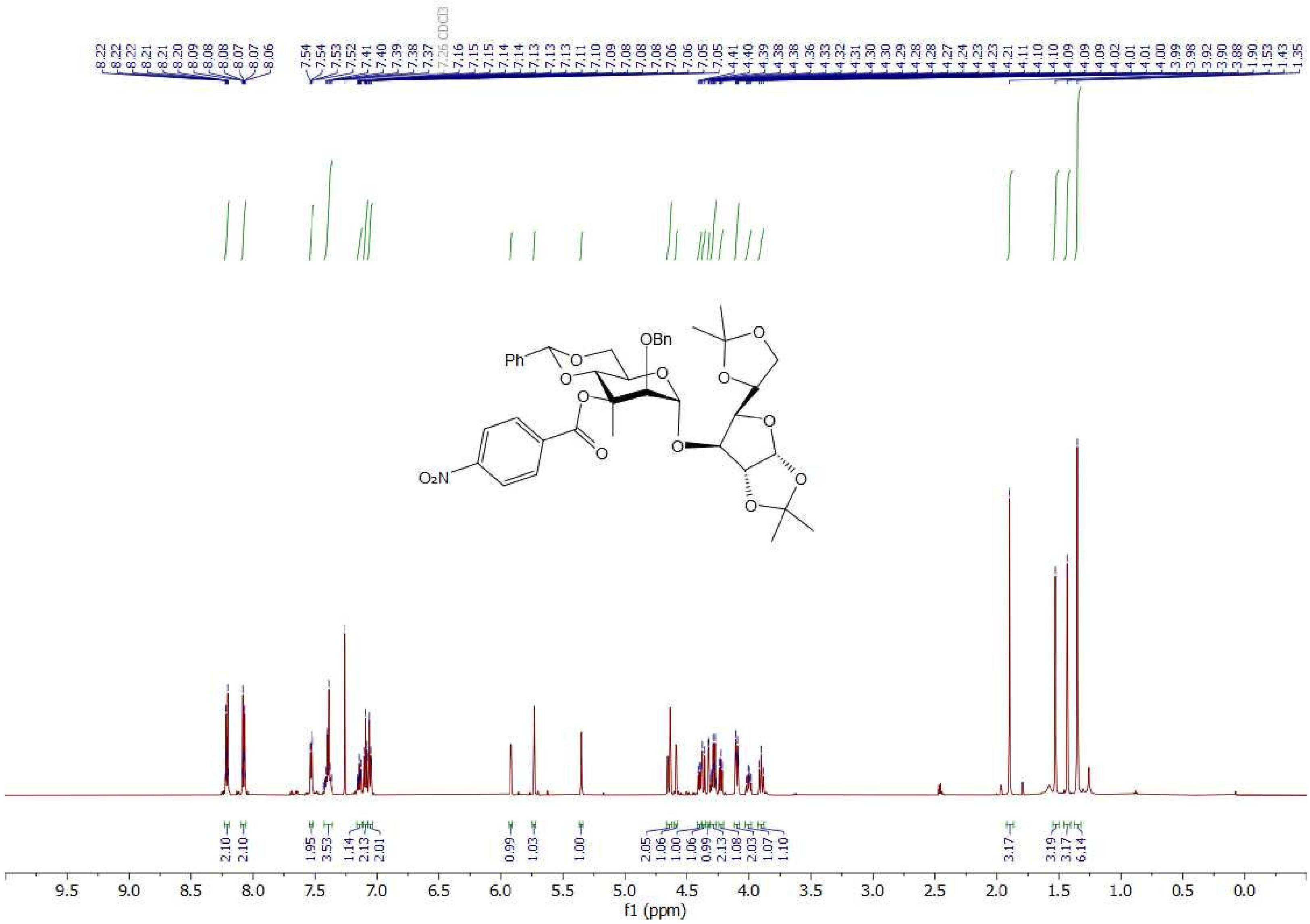

Figure S181. COSY NMR (600 MHz, $CDCl_3$) spectrum of 3-*O*-(2-*O*-benzyl-4,6-*O*-benzylidene-3-*O*-*p*-nitrobenzoyl-3-*C*-methyl-α-D-mannopyranosyl)-1,2:5,6-di-*O*-isopropylidene-α-D-glucofuranose **57**:

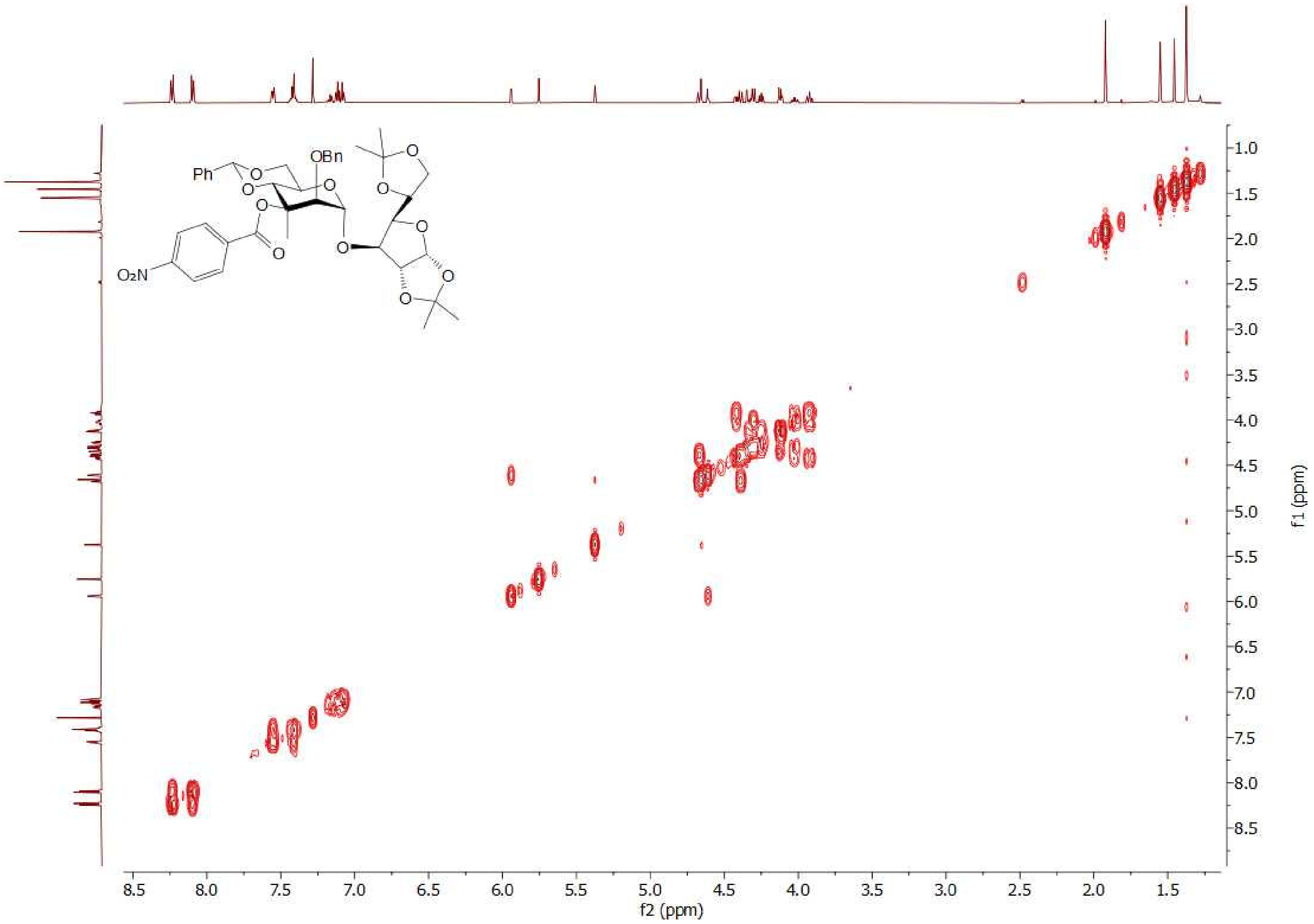

Figure S182. $^{13}C\{^{1}H\}$ NMR (151 MHz, $CDCl_3$) spectrum of 3-*O*-(2-*O*-benzyl-4,6-*O*-benzylidene-3-*O*-*p*-nitrobenzoyl-3-*C*-methyl-α-D-mannopyranosyl)-1,2:5,6-di-*O*-isopropylidene-α-D-glucofuranose **57**:

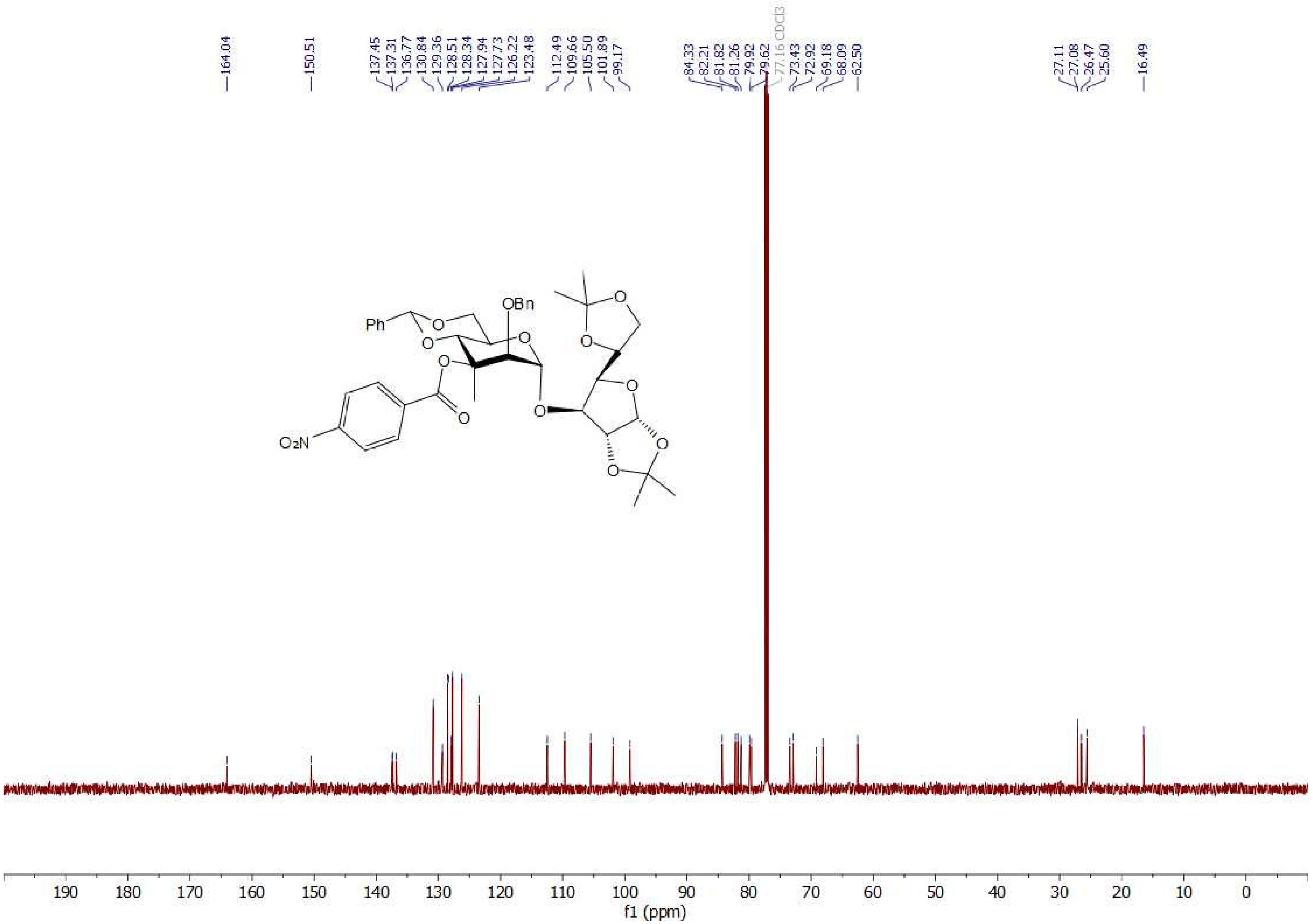

Figure S183. $^{13}C\{^1H\}$ DEPT NMR (151 MHz, $CDCl_3$) spectrum of 3-*O*-(2-*O*-benzyl-4,6-*O*-benzylidene-3-*O*-*p*-nitrobenzoyl-3-*C*-methyl-α-D-mannopyranosyl)-1,2:5,6-di-*O*-isopropylidene-α-D-glucofuranose **57**:

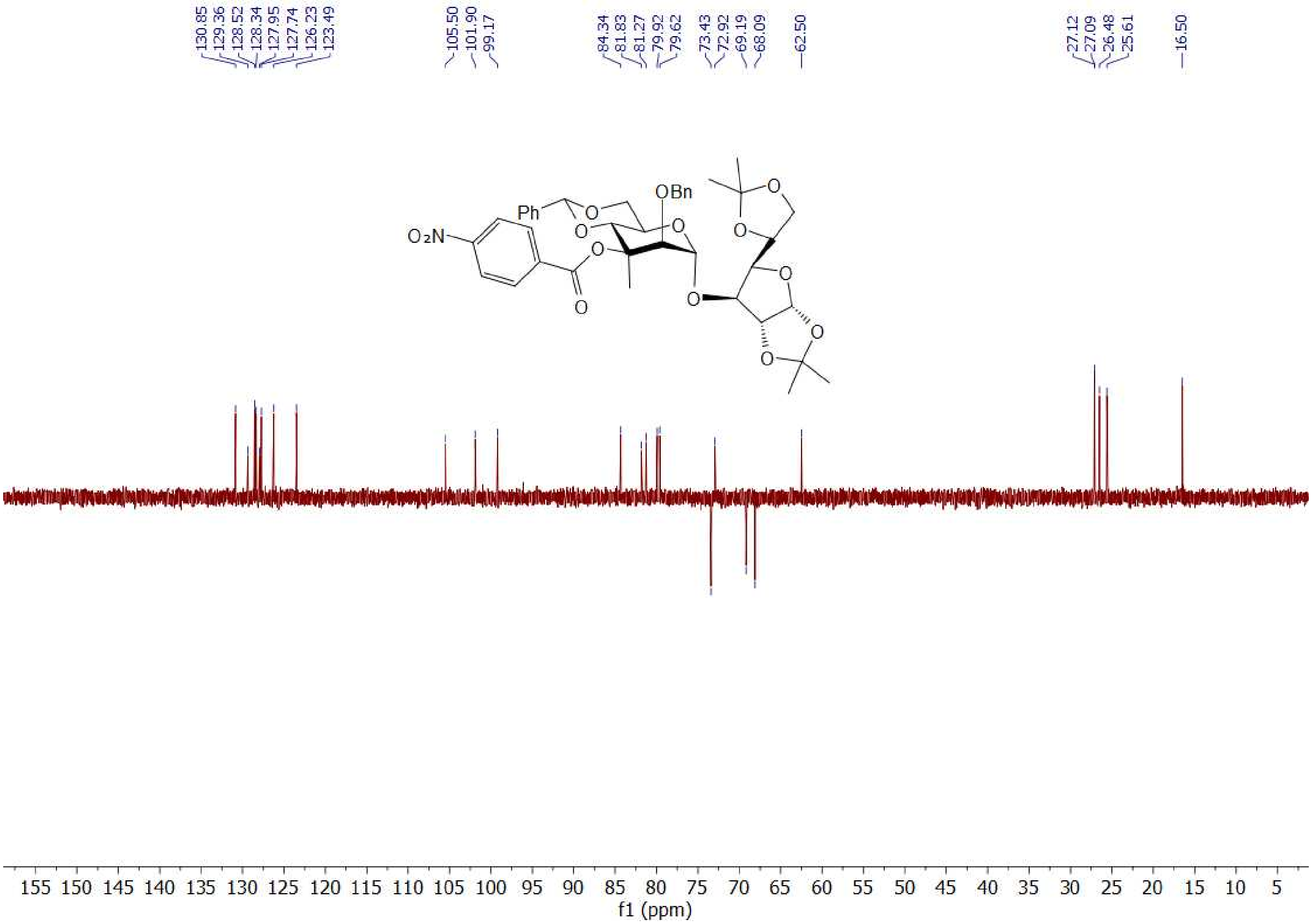

Figure S184. HSQC NMR (600 MHz, $CDCl_3$) spectrum of 3-*O*-(2-*O*-benzyl-4,6-*O*-benzylidene-3-*O*-*p*-nitrobenzoyl-3-*C*-methyl-α-D-mannopyranosyl)-1,2:5,6-di-*O*-isopropylidene-α-D-glucofuranose **57**:

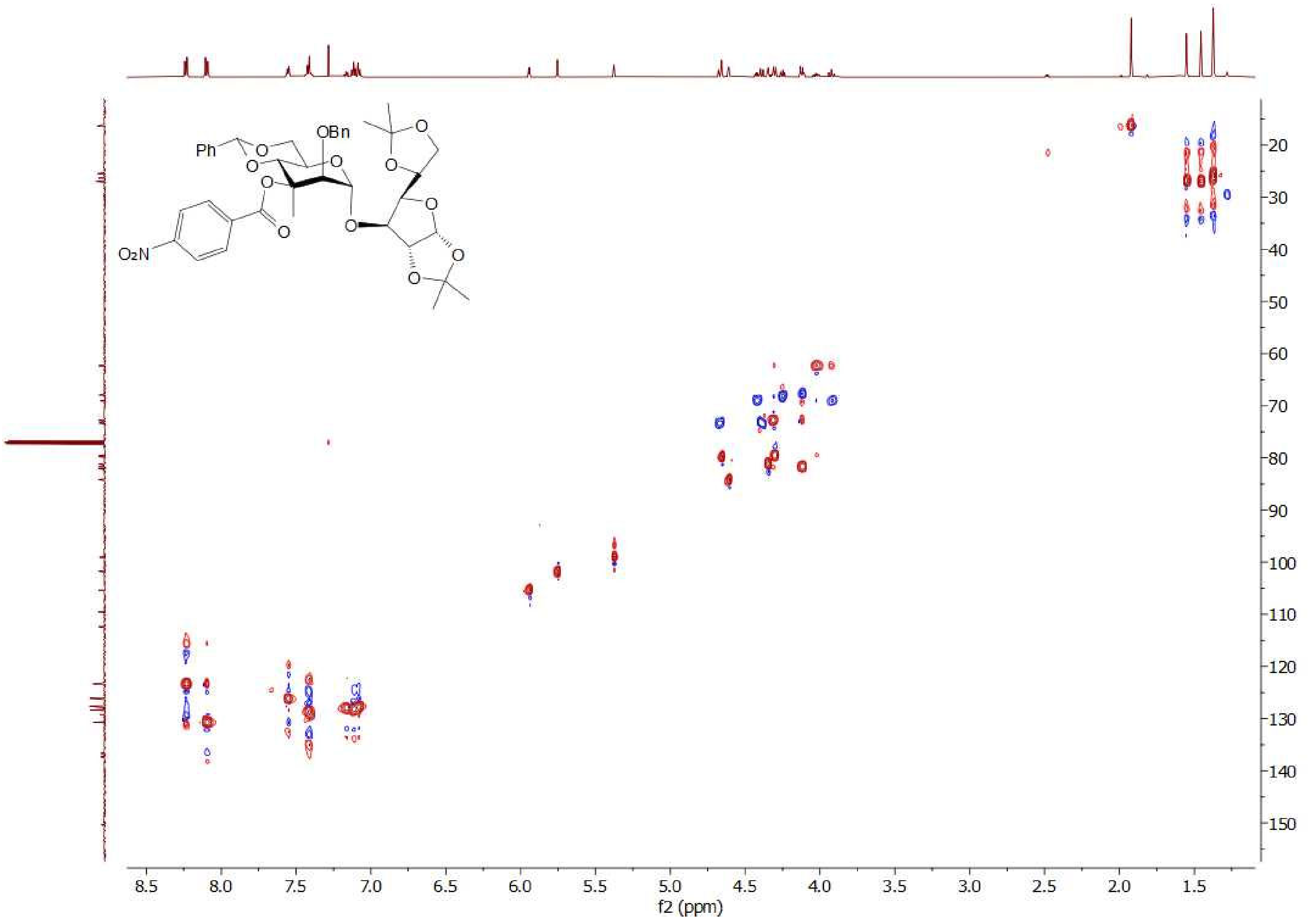

Figure S185. HMBC NMR (600 MHz, $CDCl_3$) spectrum of 3-*O*-(2-*O*-benzyl-4,6-*O*-benzylidene-3-*O*-*p*-nitrobenzoyl-3-*C*-methyl-α-D-mannopyranosyl)-1,2:5,6-di-*O*-isopropylidene-α-D-glucofuranose **57**:

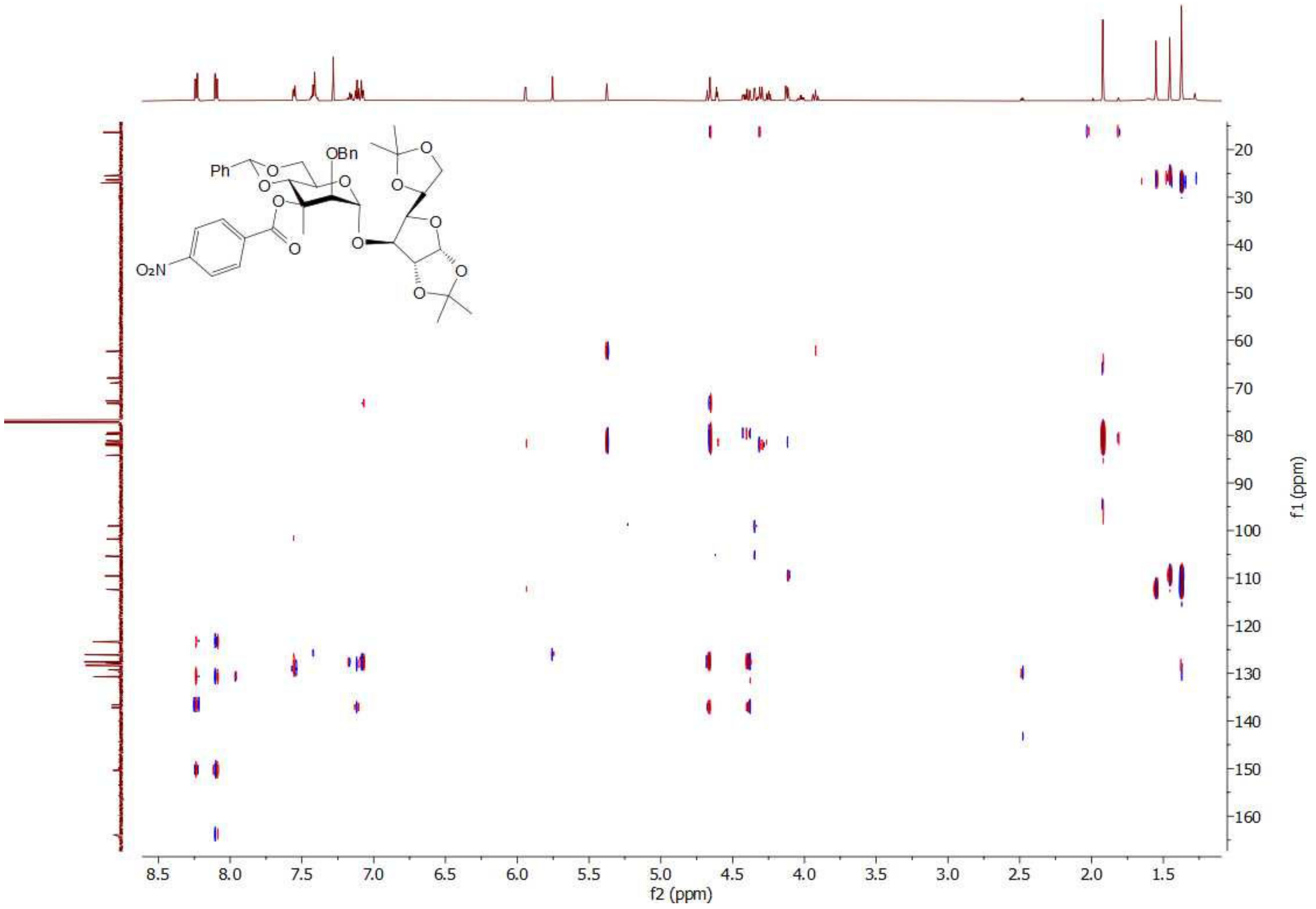

Variable Temperature (VT) NMR Study

Figure S186. VT ^{1}H NMR (500 MHz, CD_2Cl_2) study of *p*-methylphenyl 3-*O*-(benzoyl-α-^{13}C)-2-*O*-benzyl-4,6-*O*-benzylidene-thio-α-D-mannopyranoside *S*-oxide **^{13}C-49**:

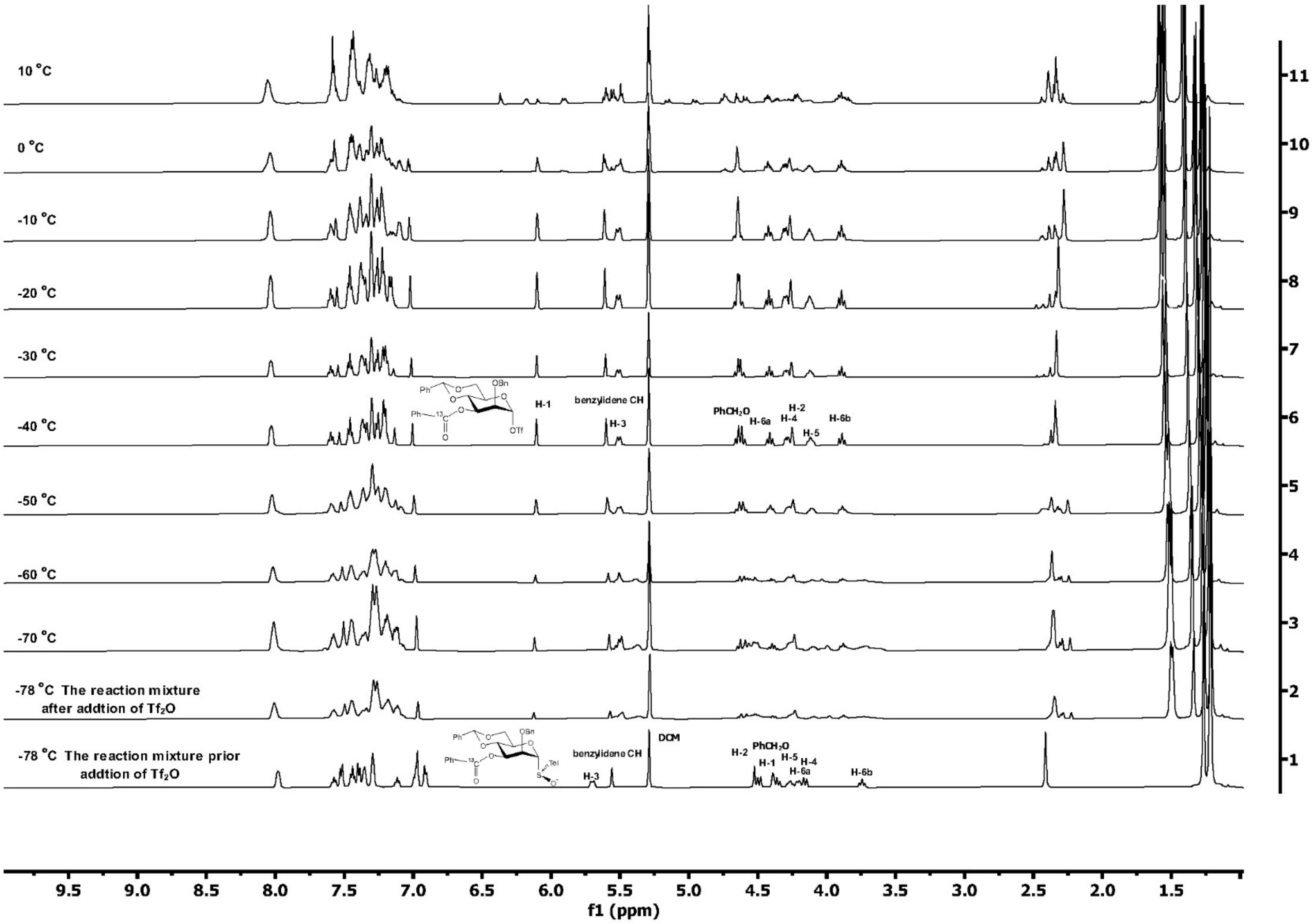

Figure S187. VT ^{1}H NMR (500 MHz, CD_2Cl_2) study of *p*-methylphenyl 3-*O*-(benzoyl-α-^{13}C)-2-*O*-benzyl-4,6-*O*-benzylidene-thio-α-D-mannopyranoside *S*-oxide **^{13}C-49** (COSY at -40 °C):

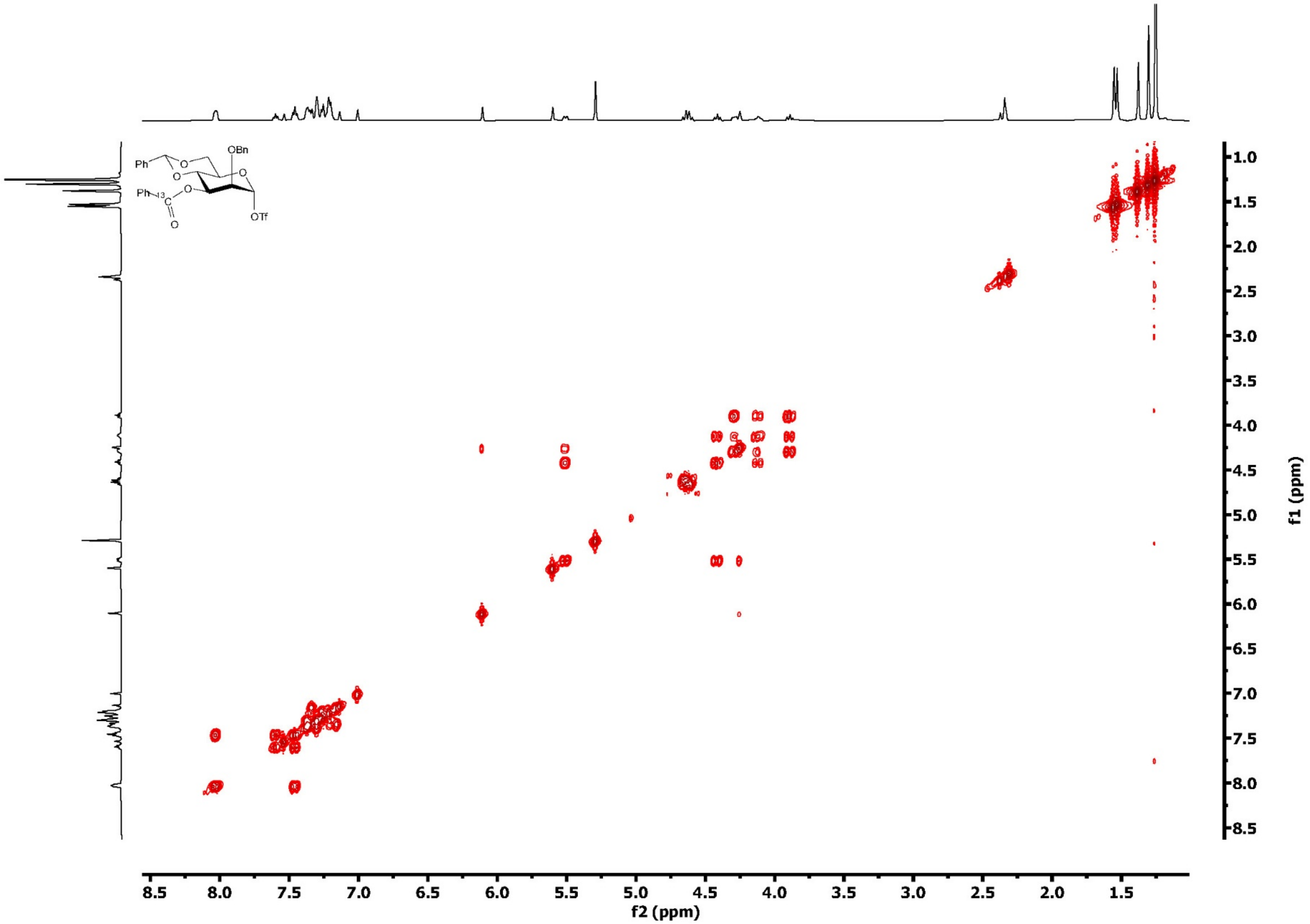

Figure S188. VT $^{13}C\{^1H\}$ NMR (126 MHz, CD_2Cl_2) study of *p*-methylphenyl 3-*O*-(benzoyl-α-^{13}C)-2-*O*-benzyl-4,6-*O*-benzylidene-thio-α-D-mannopyranoside *S*-oxide **^{13}C-49**:

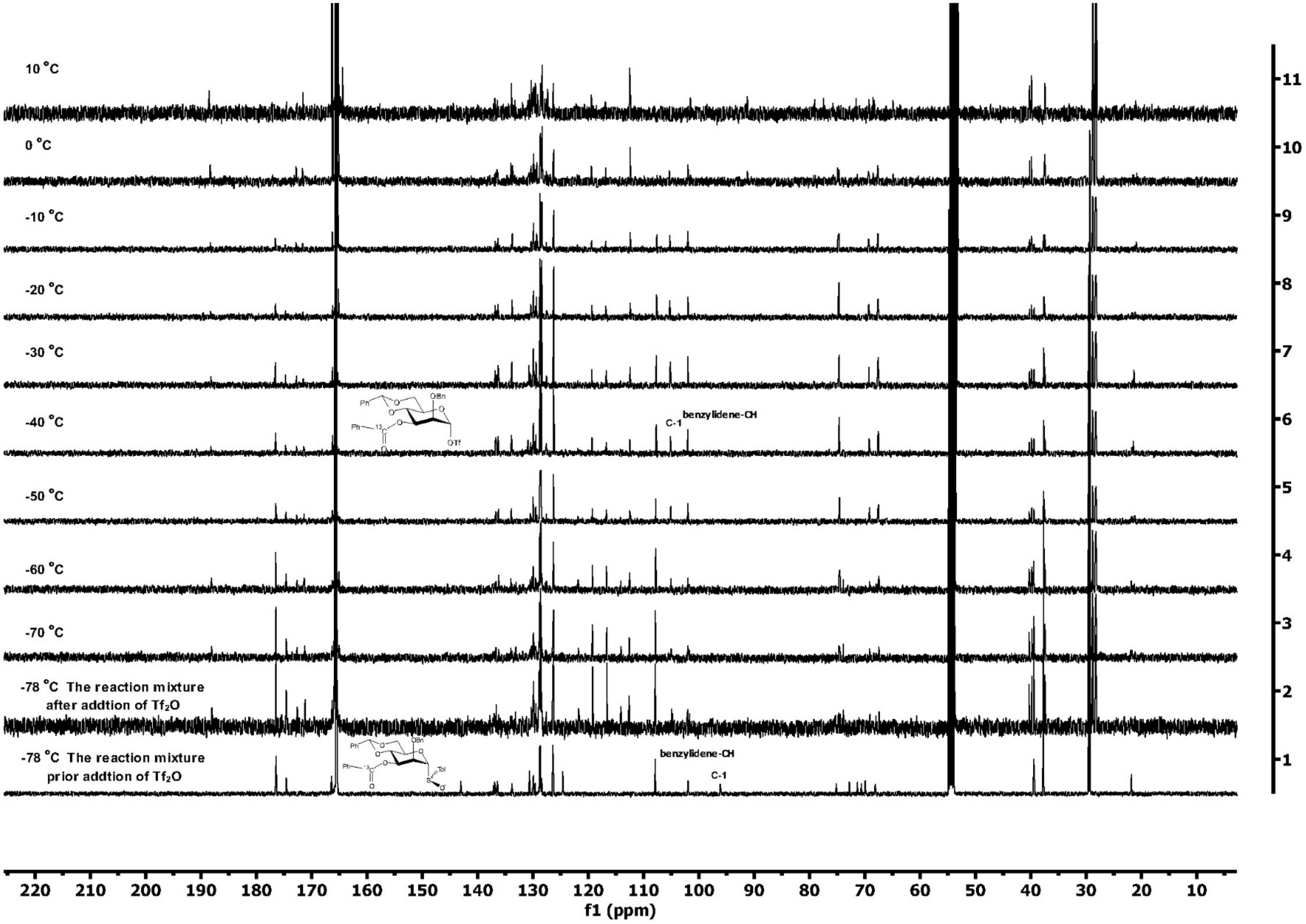

Figure S189. VT ^{19}F NMR (471 MHz, CD_2Cl_2) study of *p*-methylphenyl 3-*O*-(benzoyl-α-^{13}C)-2-*O*-benzyl-4,6-*O*-benzylidene-thio-α-D-mannopyranoside *S*-oxide **^{13}C-49**:

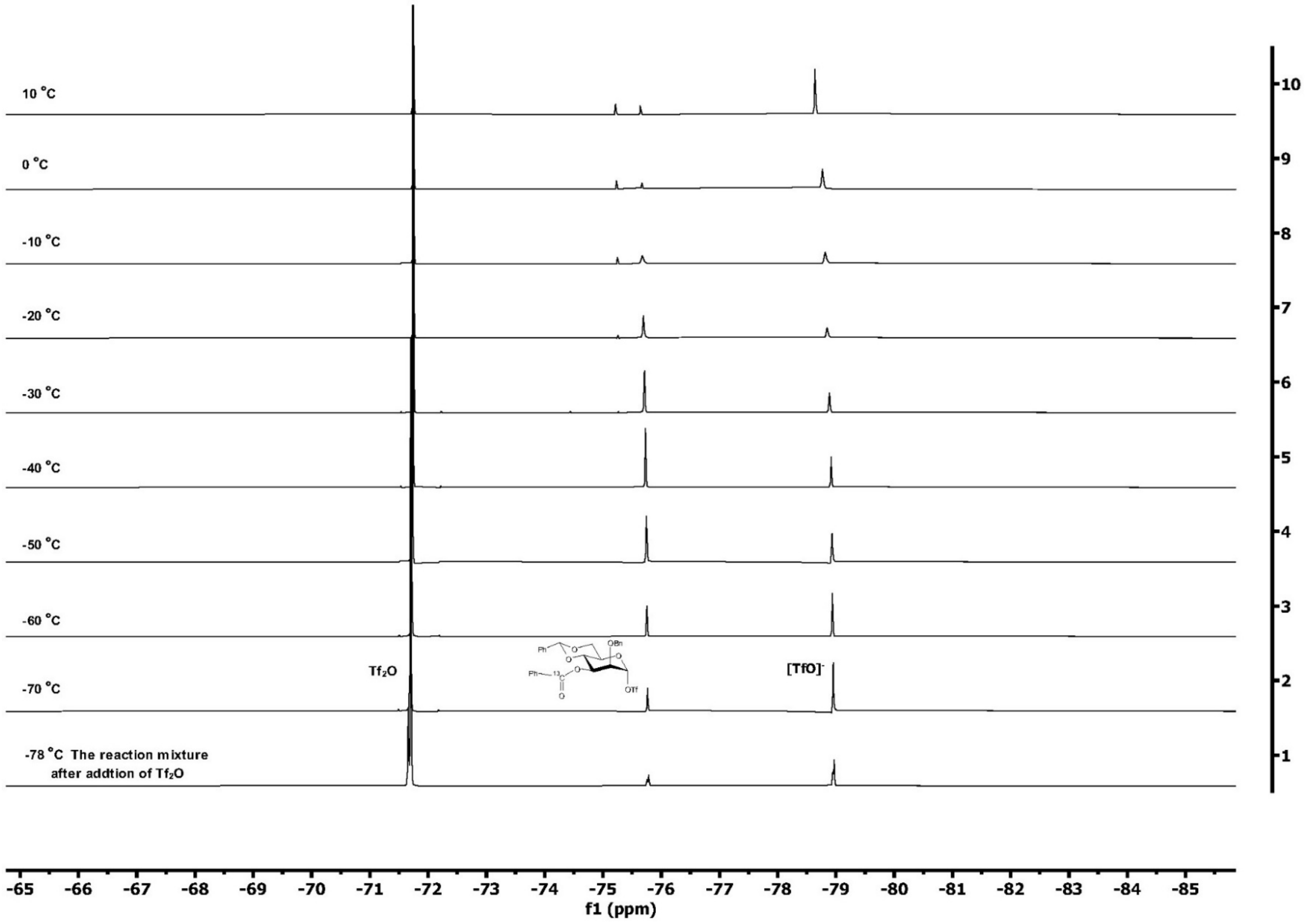

Figure S190. VT ^{1}H NMR (500 MHz, CD_2Cl_2) study of *p*-methylphenyl 3-*O*-(benzoyl-α-^{13}C)-2-*O*-benzyl-4,6-*O*-benzylidene-thio-α-D-mannopyranoside *S*-oxide **^{13}C-49** (HSQC at -40 °C):

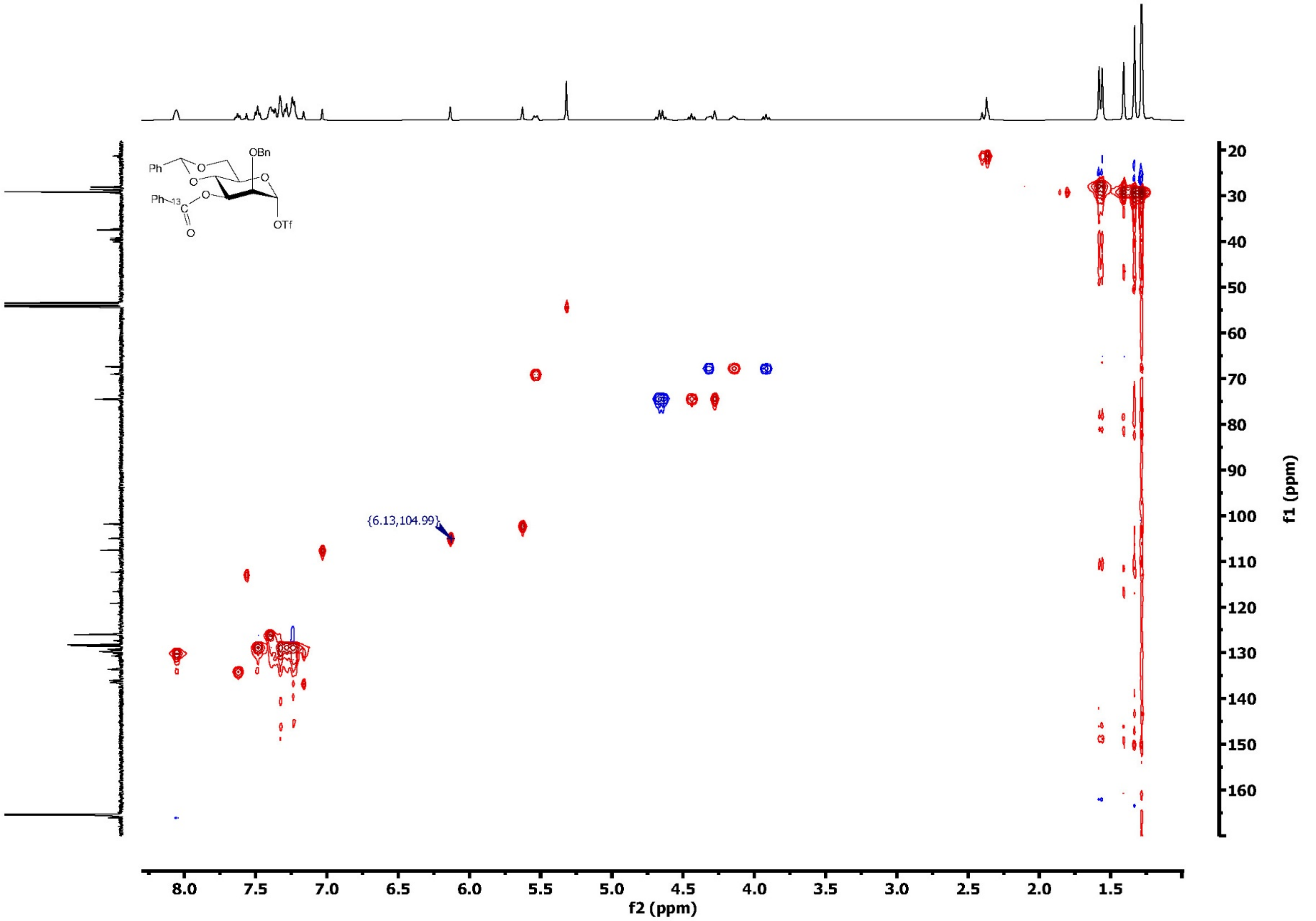

Figure S191. VT $^{13}C\{^{1}H\}$ NMR (126 MHz, CD_2Cl_2) study of *p*-methylphenyl 3-*O*-(benzoyl-α-^{13}C)-2-*O*-benzyl-4,6-*O*-benzylidene-thio-α-D-mannopyranoside *S*-oxide **^{13}C-49** (C-H coupled and decoupled at -40 °C):

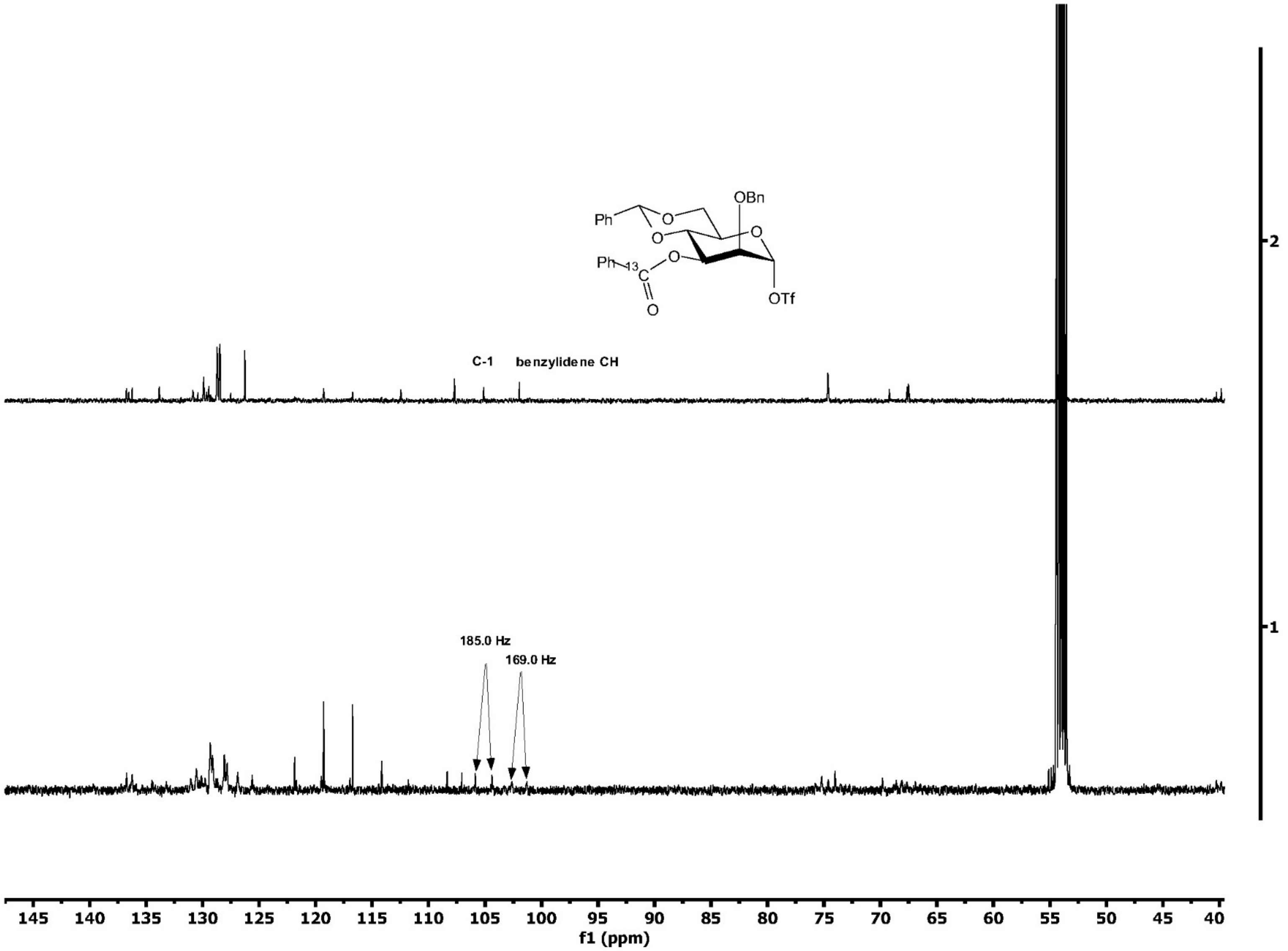

Figure S192. VT ^{1}H NMR (500 MHz, CD_2Cl_2) study of *p*-methylphenyl 3-*O*-(benzoyl-α-^{13}C)-2-*O*-benzyl-4,6-*O*-benzylidene-3-*C*-methyl-thio-β-D-mannopyranoside *S*-oxide **^{13}C-40** (Less-polar diastereoisomer):

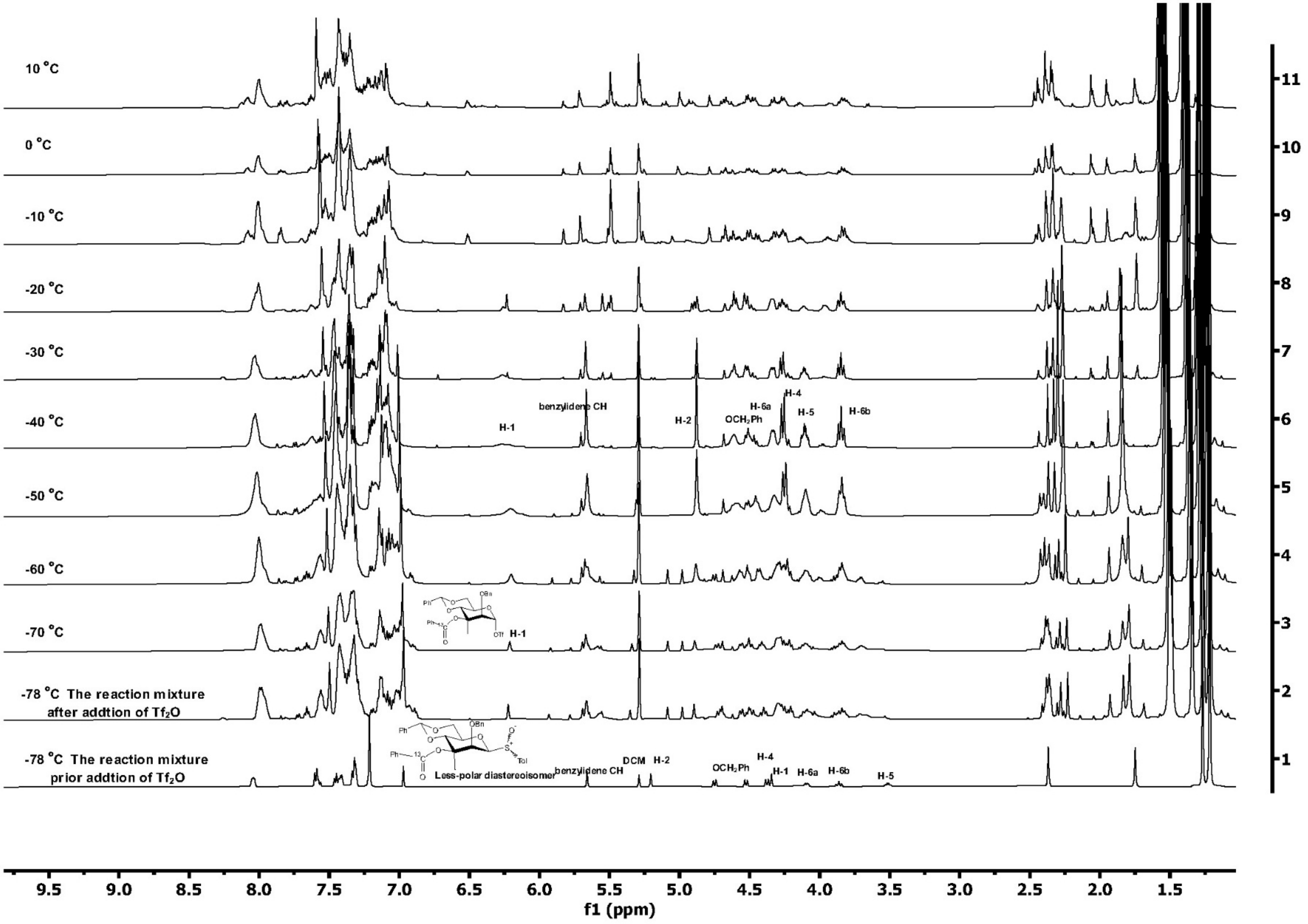

Figure S193. VT ^{1}H NMR (500 MHz, CD_2Cl_2) study of *p*-methylphenyl 3-*O*-(benzoyl-α-^{13}C)-2-*O*-benzyl-4,6-*O*-benzylidene-3-*C*-methyl-thio-β-D-mannopyranoside *S*-oxide **^{13}C-40** (Less-polar diastereoisomer; COSY at -40 °C):

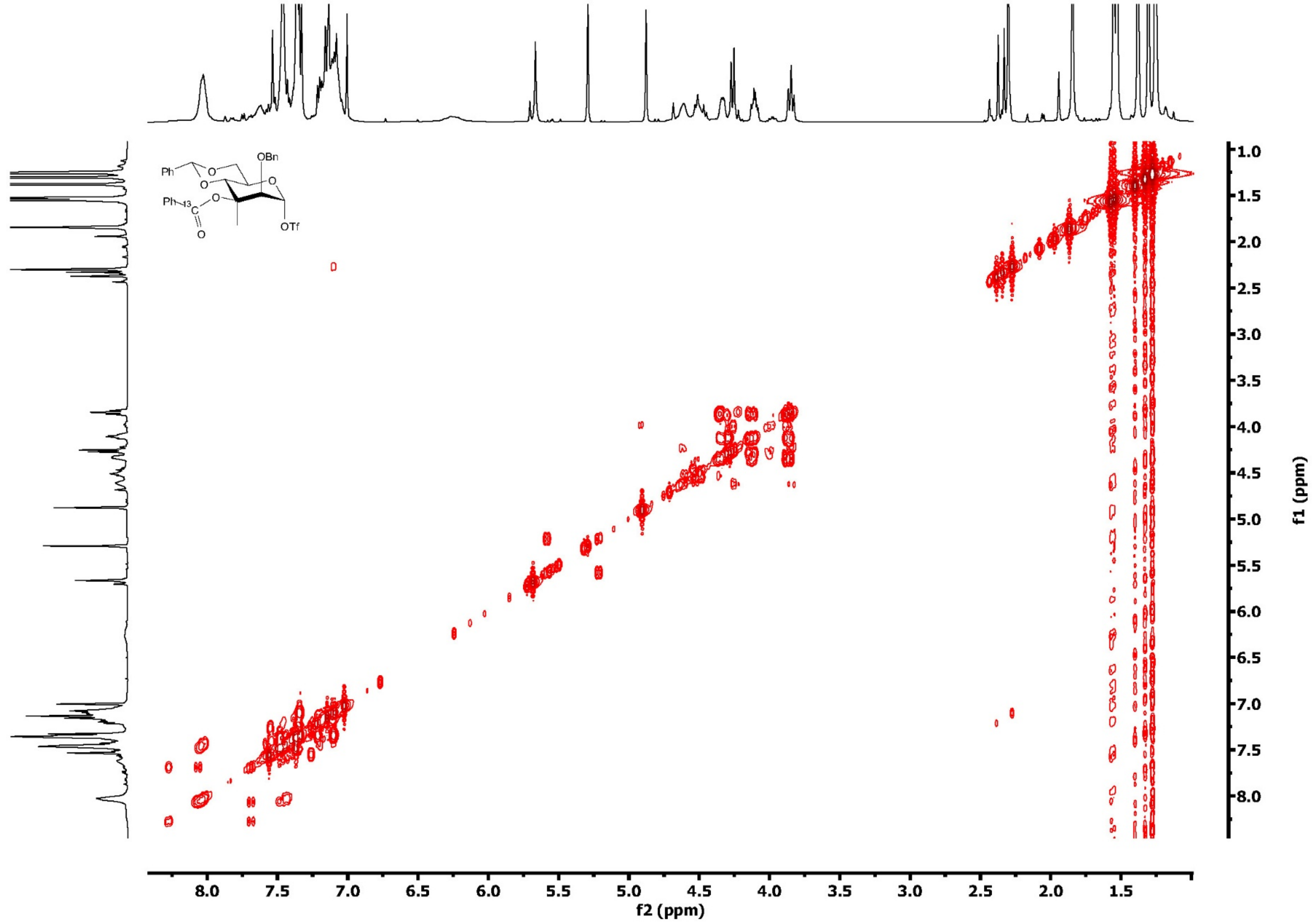

Figure S194. VT $^{13}C\{^{1}H\}$ NMR (126 MHz, CD_2Cl_2) study of *p*-methylphenyl 3-*O*-(benzoyl-α-^{13}C)-2-*O*-benzyl-4,6-*O*-benzylidene-3-*C*-methyl-thio-β-D-mannopyranoside *S*-oxide **^{13}C-40** (Less-polar diastereoisomer):

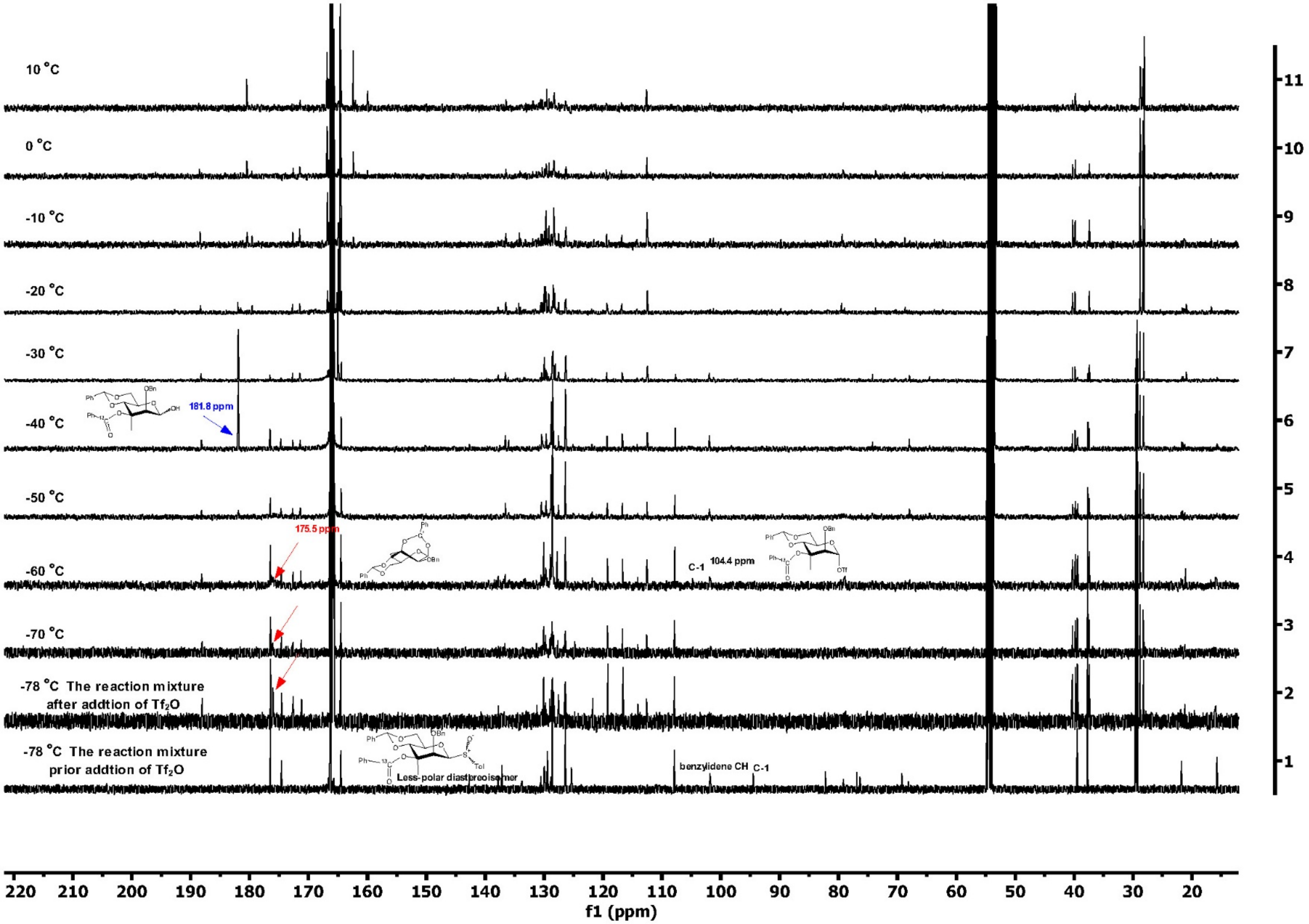

Figure S195. VT ^{19}F NMR (471 MHz, CD_2Cl_2) study of *p*-methylphenyl 3-*O*-(benzoyl-α-^{13}C)-2-*O*-benzyl-4,6-*O*-benzylidene-3-*C*-methyl-thio-β-D-mannopyranoside *S*-oxide **^{13}C-40** (Less-polar diastereoisomer):

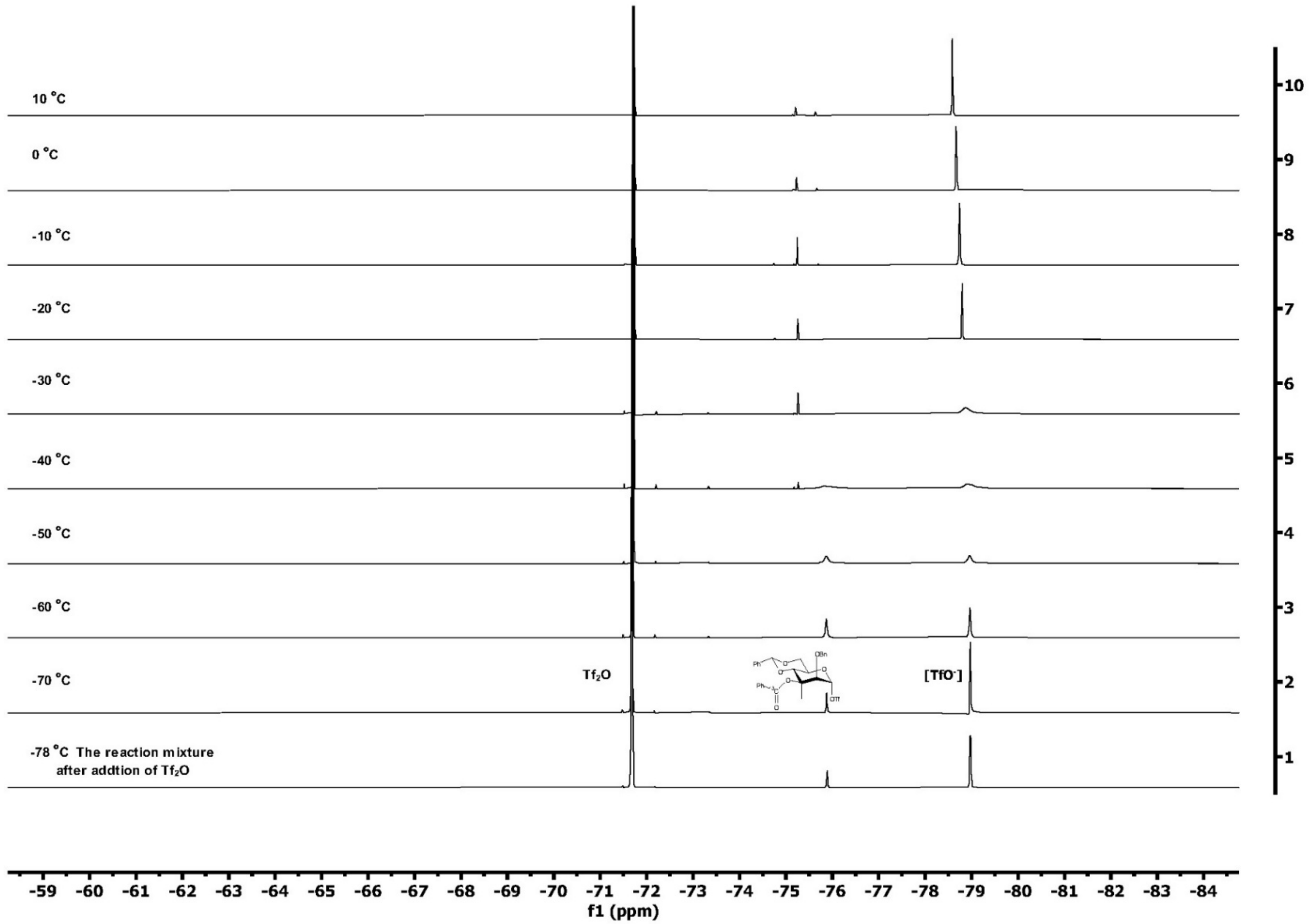

Figure S196. VT ^{1}H NMR (500 MHz, CD_2Cl_2) study of *p*-methylphenyl 3-*O*-(benzoyl-α-^{13}C)-2-*O*-benzyl-4,6-*O*-benzylidene-3-*C*-methyl-thio-β-D-mannopyranoside *S*-oxide **^{13}C-40** (HSQC at -70 °C) (Less-polar diastereoisomer):

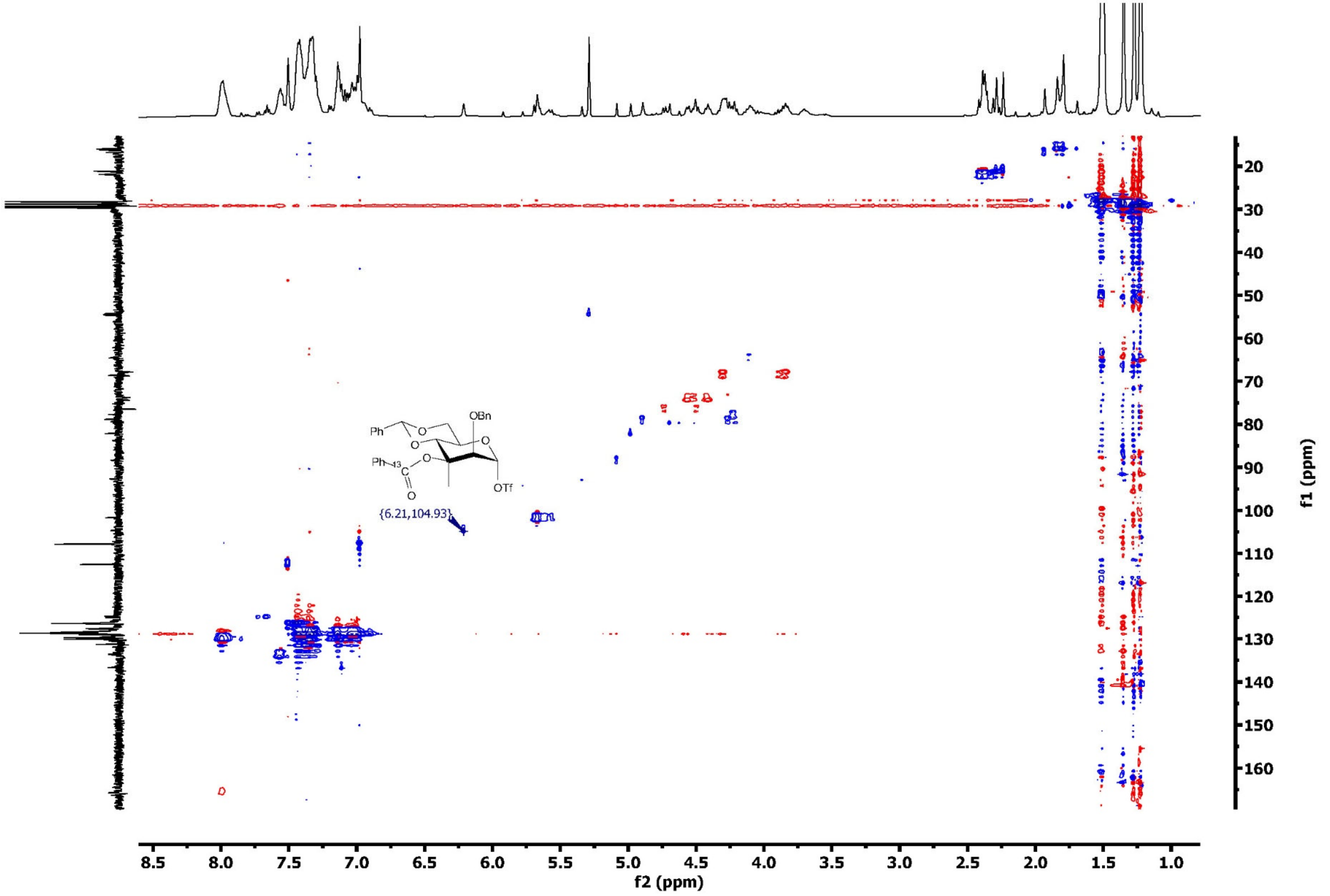

Figure S197. VT ^{1}H NMR (500 MHz, CD_2Cl_2) study of *p*-methylphenyl 3-*O*-(benzoyl-α-^{13}C)-2-*O*-benzyl-4,6-*O*-benzylidene-3-*C*-methyl-thio-β-D-mannopyranoside *S*-oxide **^{13}C-40** (Less-polar diastereoisomer):

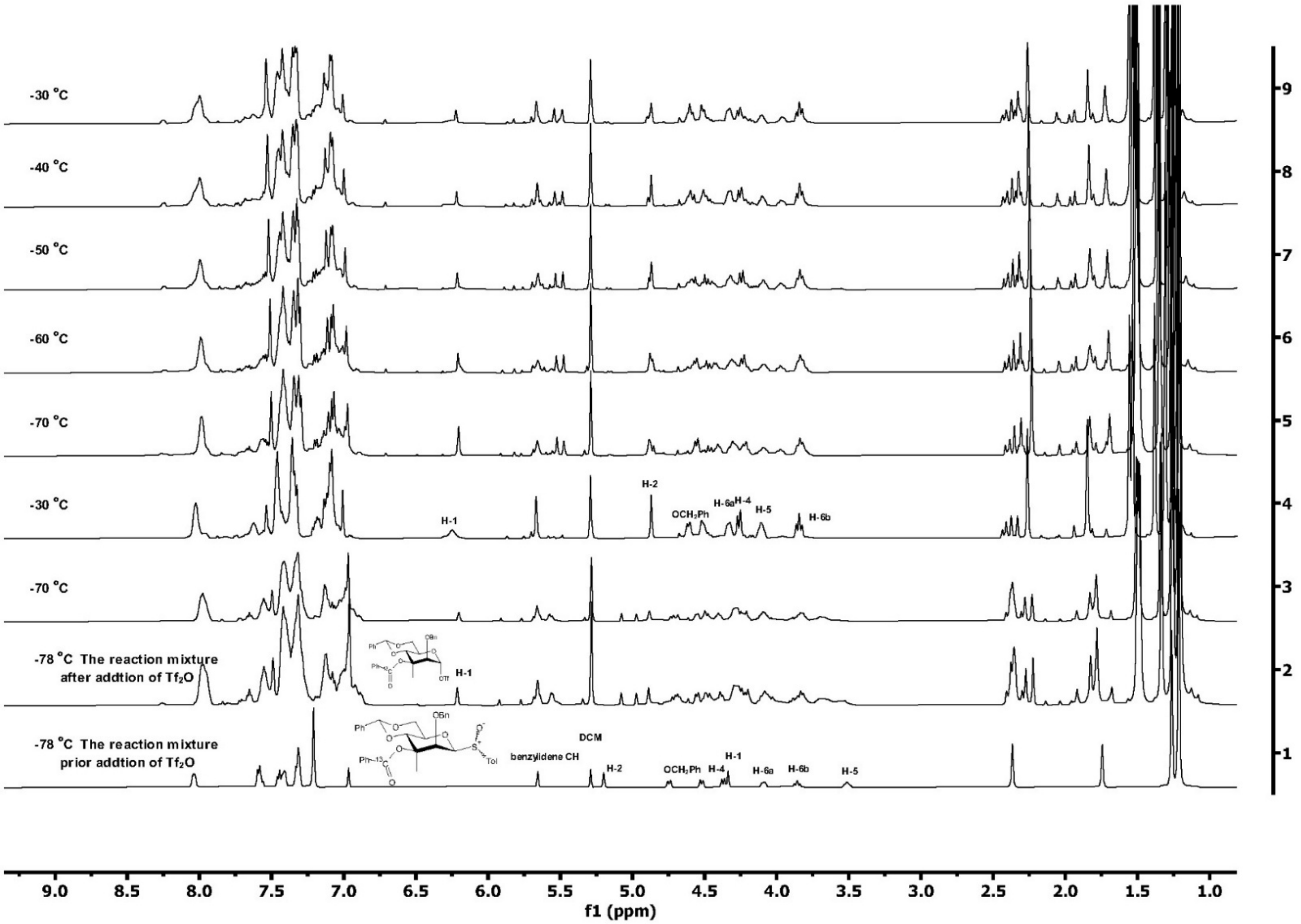

Note: The probe temperature was quickly increased from -70 °C to -30 °C, once ^{1}H, ^{13}C and ^{19}F were recorded, then decreased again to -70 °C. The 10 °C increments continued up to -30 °C.

Figure S198. VT $^{13}C\{^{1}H\}$ NMR (126 MHz, CD_2Cl_2) study of *p*-methylphenyl 3-*O*-(benzoyl-α-^{13}C)-2-*O*-benzyl-4,6-*O*-benzylidene-3-*C*-methyl-thio-β-D-mannopyranoside *S*-oxide **^{13}C-40** (Less-polar diastereoisomer):

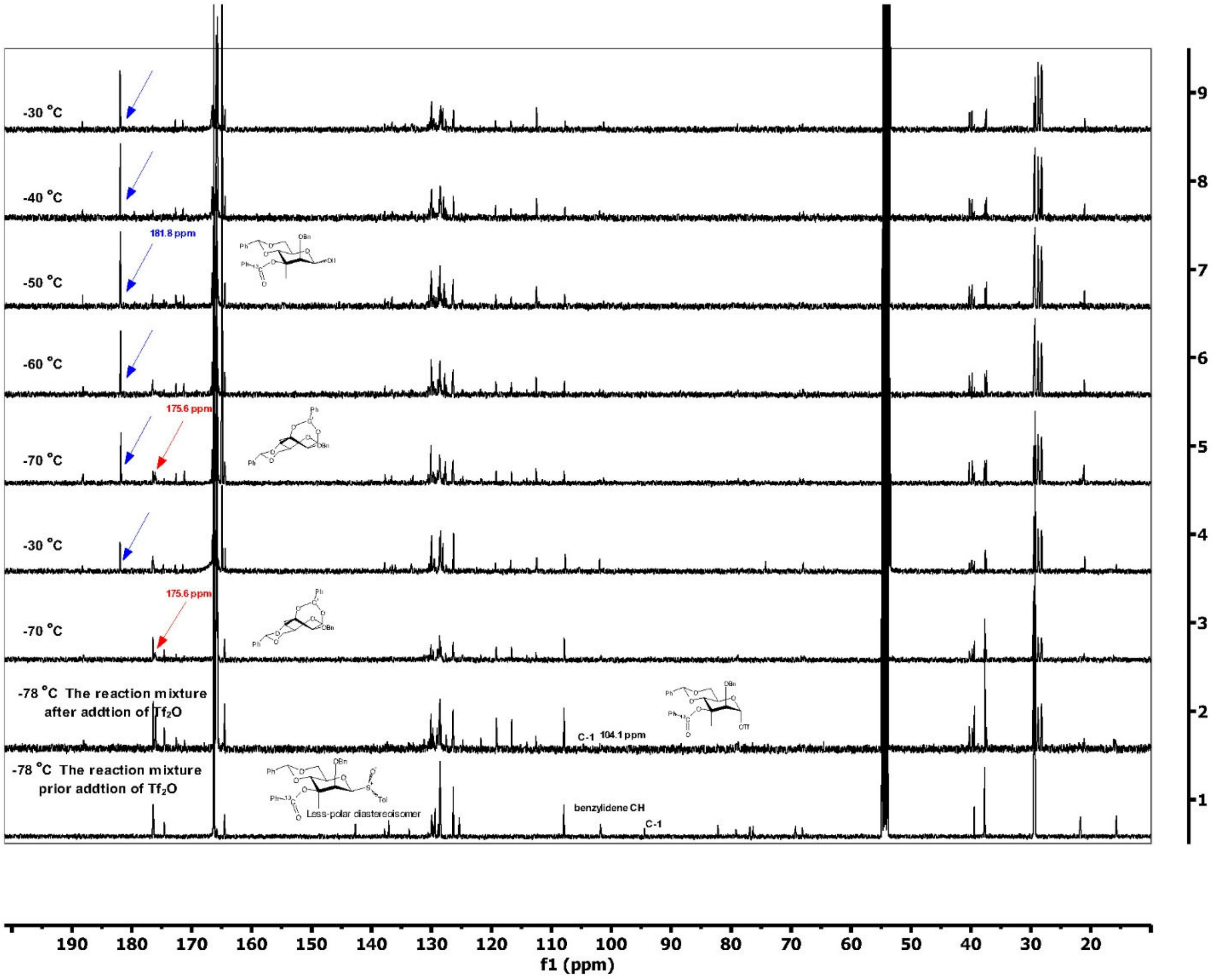

Note: The probe temperature was quickly increased from -70 °C to -30 °C, once ^{1}H, ^{13}C and ^{19}F were recorded, then decreased again to -70 °C. The 10 °C increments continued up to -30 °C.

Figure S199. VT ^{19}F NMR (471 MHz, CD_2Cl_2) study of *p*-methylphenyl 3-*O*-(benzoyl-α-^{13}C)-2-*O*-benzyl-4,6-*O*-benzylidene-3-*C*-methyl-thio-β-D-mannopyranoside *S*-oxide **^{13}C-40** (Less-polar diastereoisomer):

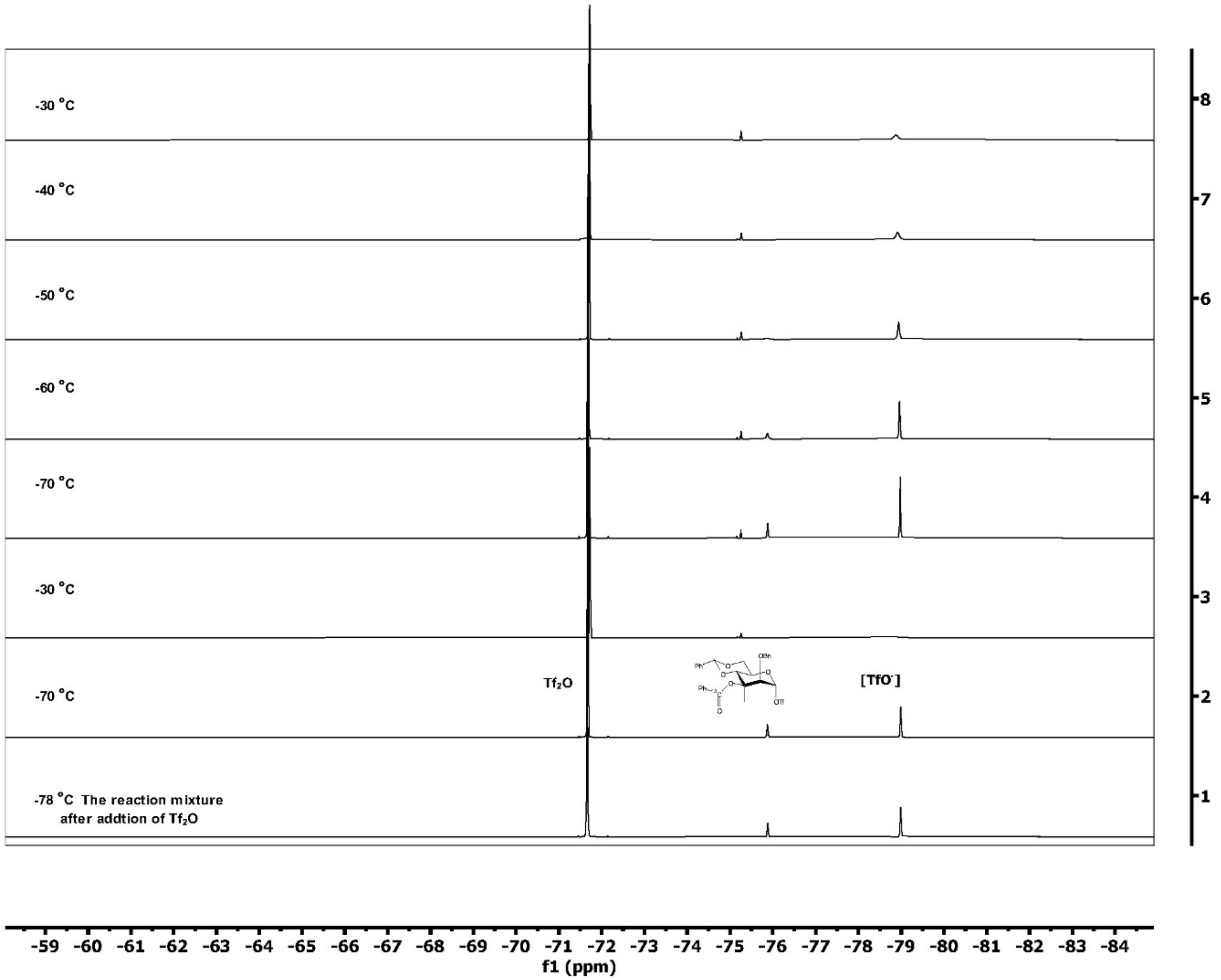

Note: The probe temperature was quickly increased from -70 °C to -30 °C, once ^{1}H, ^{13}C and ^{19}F were recorded, then decreased again to -70 °C. The 10 °C increments continued up to -30 °C.

Figure S200. VT ^{1}H NMR (500 MHz, CD_2Cl_2) study of *p*-methylphenyl 3-*O*-(benzoyl-α-^{13}C)-2-*O*-benzyl-4,6-*O*-benzylidene-3-*C*-methyl-thio-α-D-mannopyranoside *S*-oxide ^{13}**C-46:**

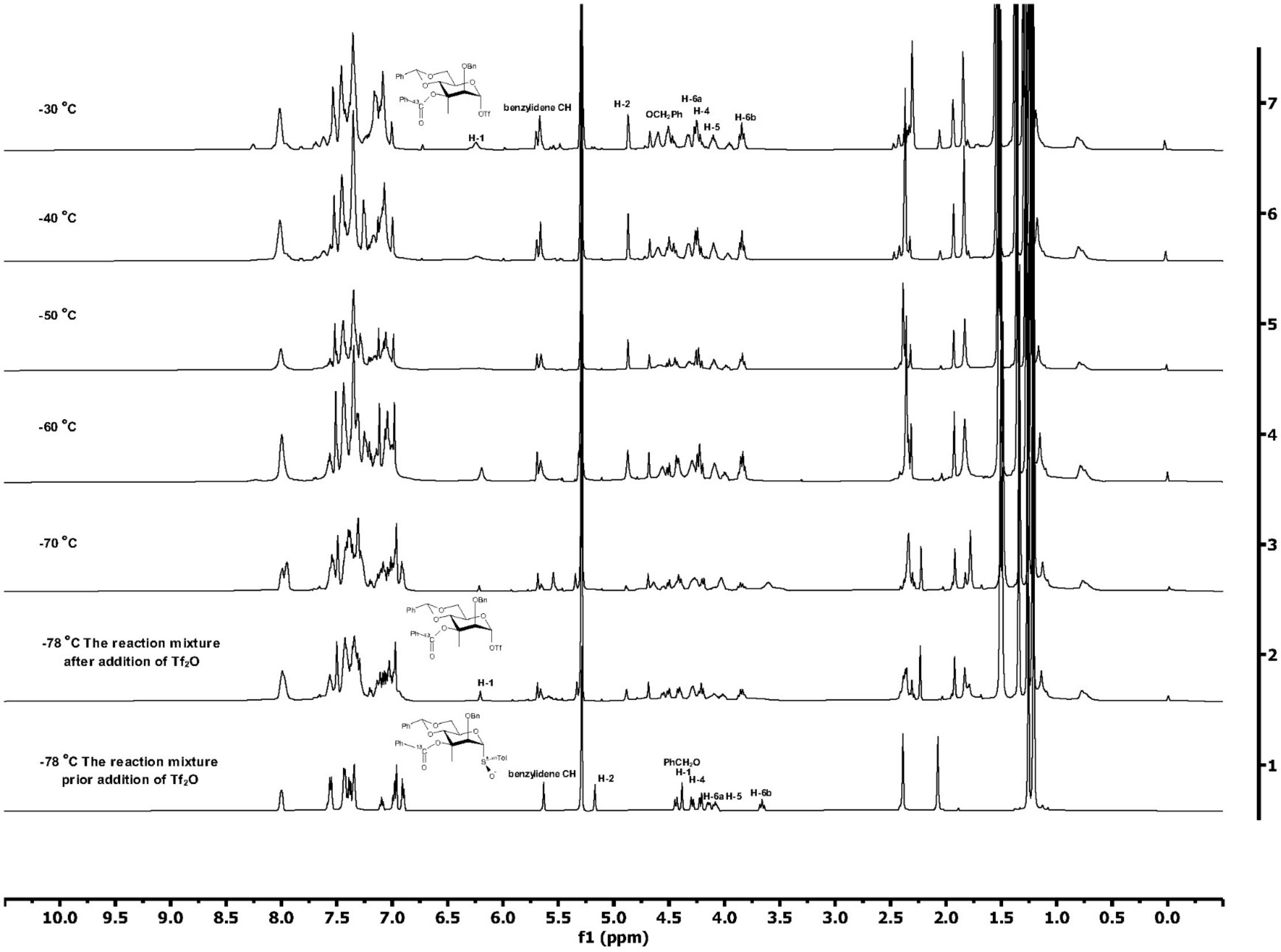

Figure S201. VT $^{13}C\{^1H\}$ NMR (126 MHz, CD_2Cl_2) study of *p*-methylphenyl 3-*O*-(benzoyl-α-^{13}C)-2-*O*-benzyl-4,6-*O*-benzylidene-3-*C*-methyl-thio-α-D-mannopyranoside *S*-oxide **^{13}C-46**:

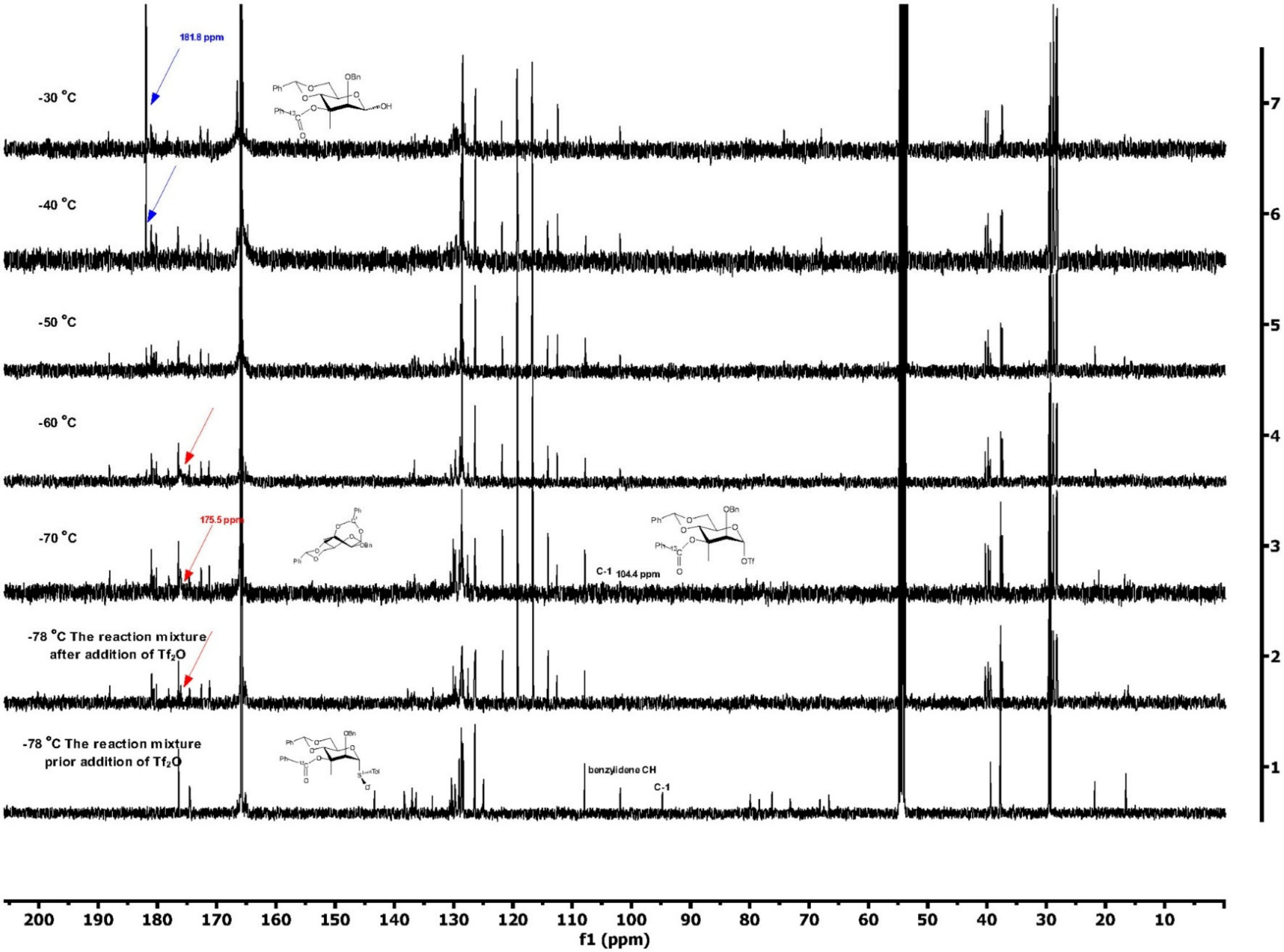

Figure S202. VT ^{19}F NMR (471 MHz, CD_2Cl_2) study of *p*-methylphenyl 3-*O*-(benzoyl-α-^{13}C)-2-*O*-benzyl-4,6-*O*-benzylidene-3-*C*-methyl-thio-α-D-mannopyranoside *S*-oxide **^{13}C-46**:

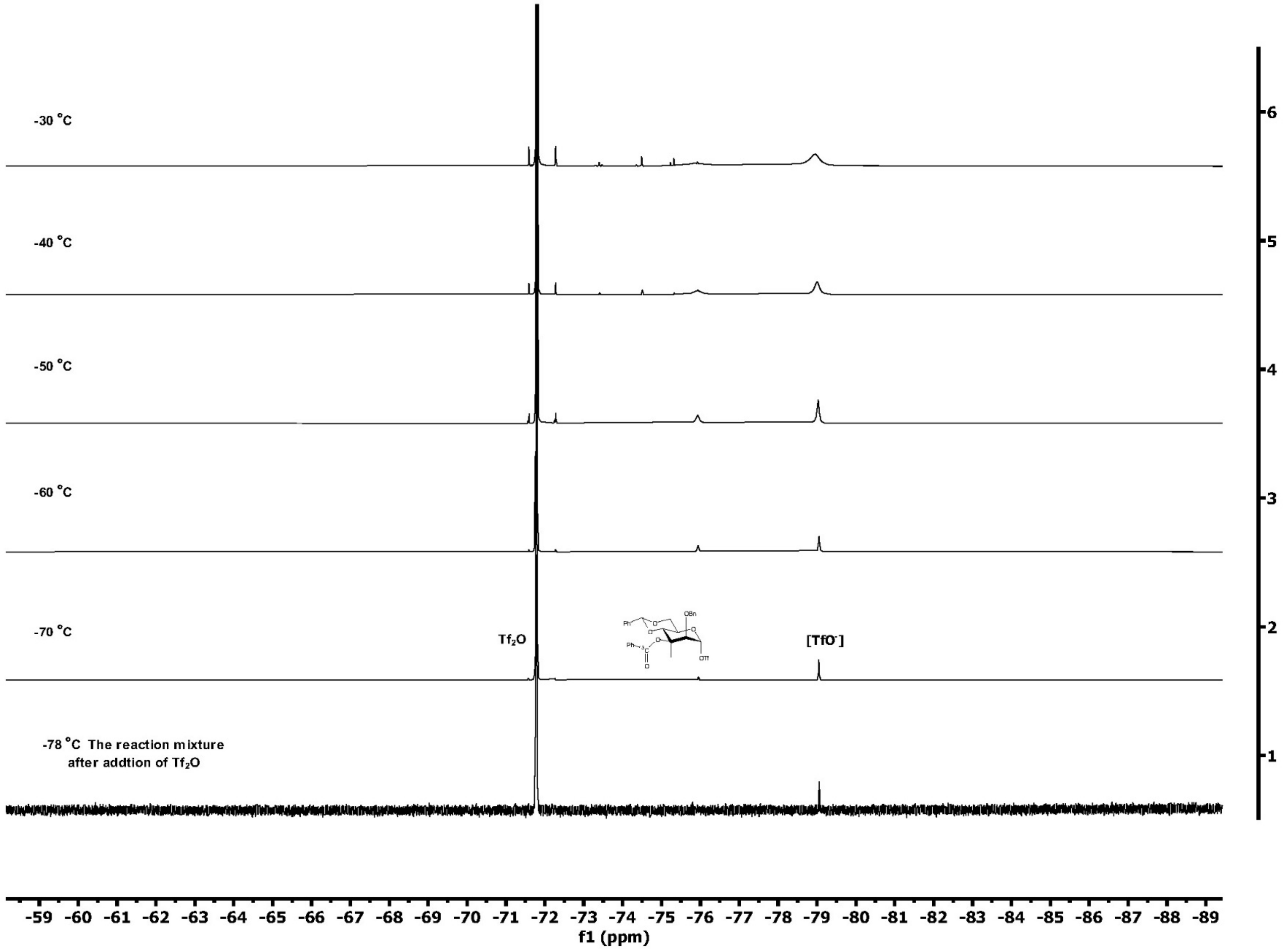

Figure S203. VT ^{1}H NMR (500 MHz, CD_2Cl_2) study of *p*-methylphenyl 3-*O*-(benzoyl-α-^{13}C)-2-*O*-benzyl-4,6-*O*-benzylidene-3-*C*-methyl-thio-β-D-mannopyranoside *S*-oxide **^{13}C-40** (Less-polar diastereoisomer) and *p*-methylphenyl 3-*O*-(benzoyl-α-^{13}C)-2-*O*-benzyl-4,6-*O*-benzylidene-3-*C*-methyl-thio-α-D-mannopyranoside *S*-oxide **^{13}C-46** (protons stacked at -30 °C):

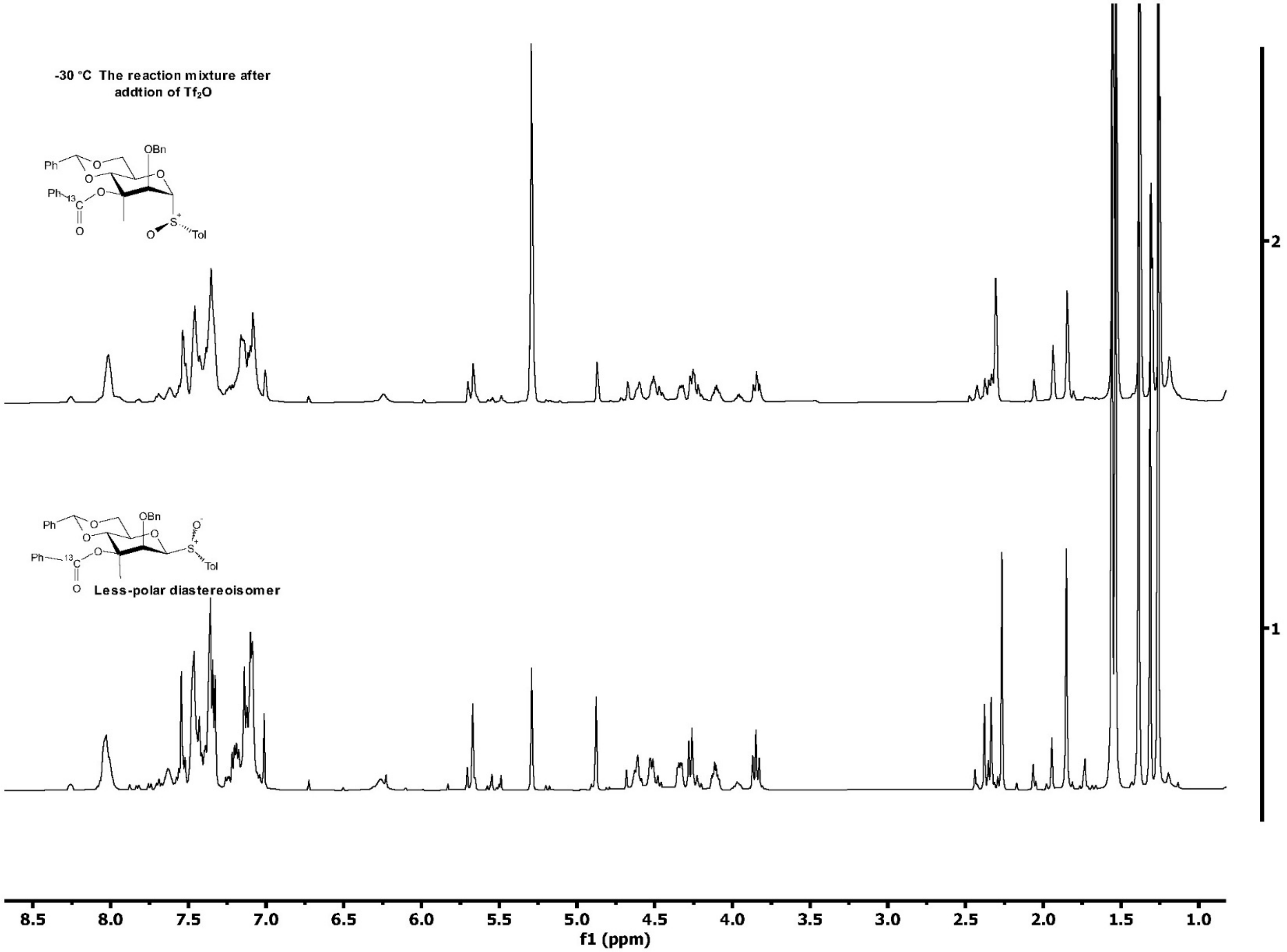

Figure S204. VT $^{13}C\{^1H\}$ NMR (126 MHz, CD_2Cl_2) study of *p*-methylphenyl 3-*O*-(benzoyl-α-^{13}C)-2-*O*-benzyl-4,6-*O*-benzylidene-3-*C*-methyl-thio-β-D-mannopyranoside *S*-oxide **^{13}C-40** (Less-polar diastereoisomer) and *p*-methylphenyl 3-*O*-(benzoyl-α-^{13}C)-2-*O*-benzyl-4,6-*O*-benzylidene-3-*C*-methyl-thio-α-D-mannopyranoside *S*-oxide **^{13}C-46** (carbons stacked at -30 °C):

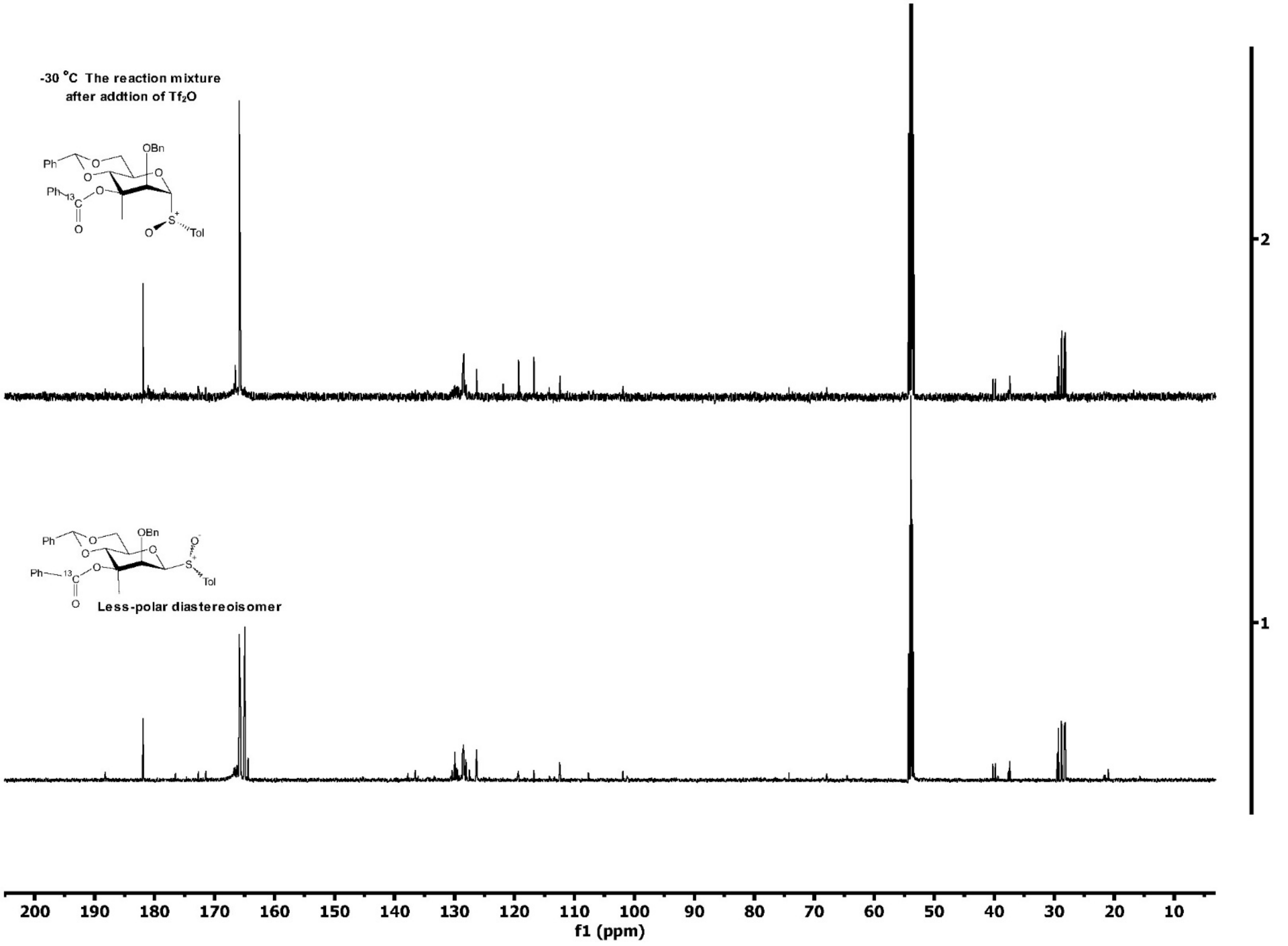

Figure S205. VT ^{1}H NMR (500 MHz, CD_2Cl_2) study of *p*-methylphenyl 3-*O*-(benzoyl-α-^{13}C)-2-*O*-benzyl-4,6-*O*-benzylidene-3-*C*-methyl-thio-β-D-mannopyranoside *S*-oxide **^{13}C-40** (Less-polar diastereoisomer):

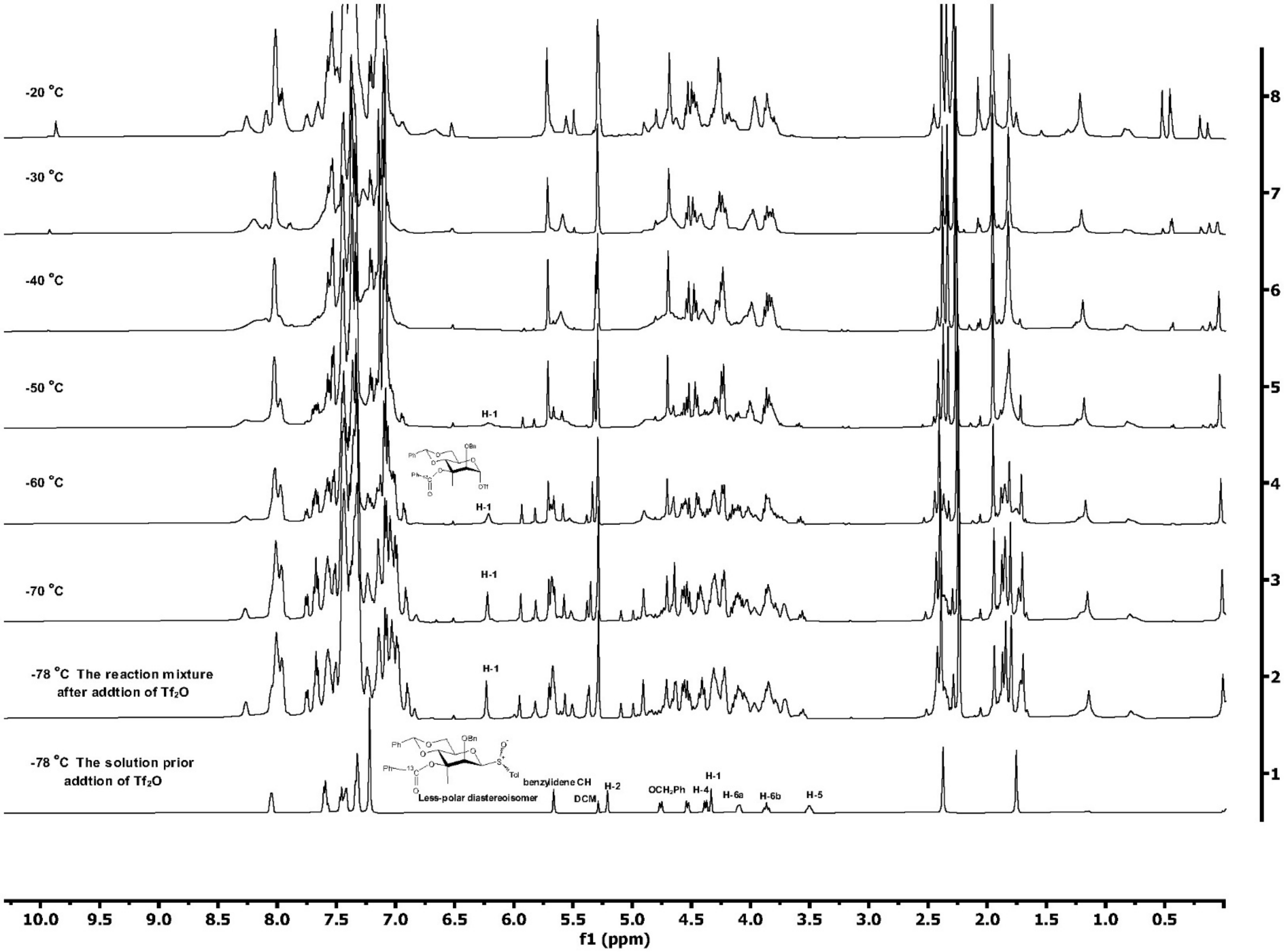

Note: This VT NMR study was conducted without TTBP (from -78 °C to 0 °C) according to Table 1. Entry 2.

Figure S206. VT $^{13}C\{^{1}H\}$ NMR (126 MHz, CD_2Cl_2) study of *p*-methylphenyl 3-*O*-(benzoyl-α-^{13}C)-2-*O*-benzyl-4,6-*O*-benzylidene-3-*C*-methyl-thio-β-D-mannopyranoside *S*-oxide **^{13}C-40** (Less-polar diastereoisomer):

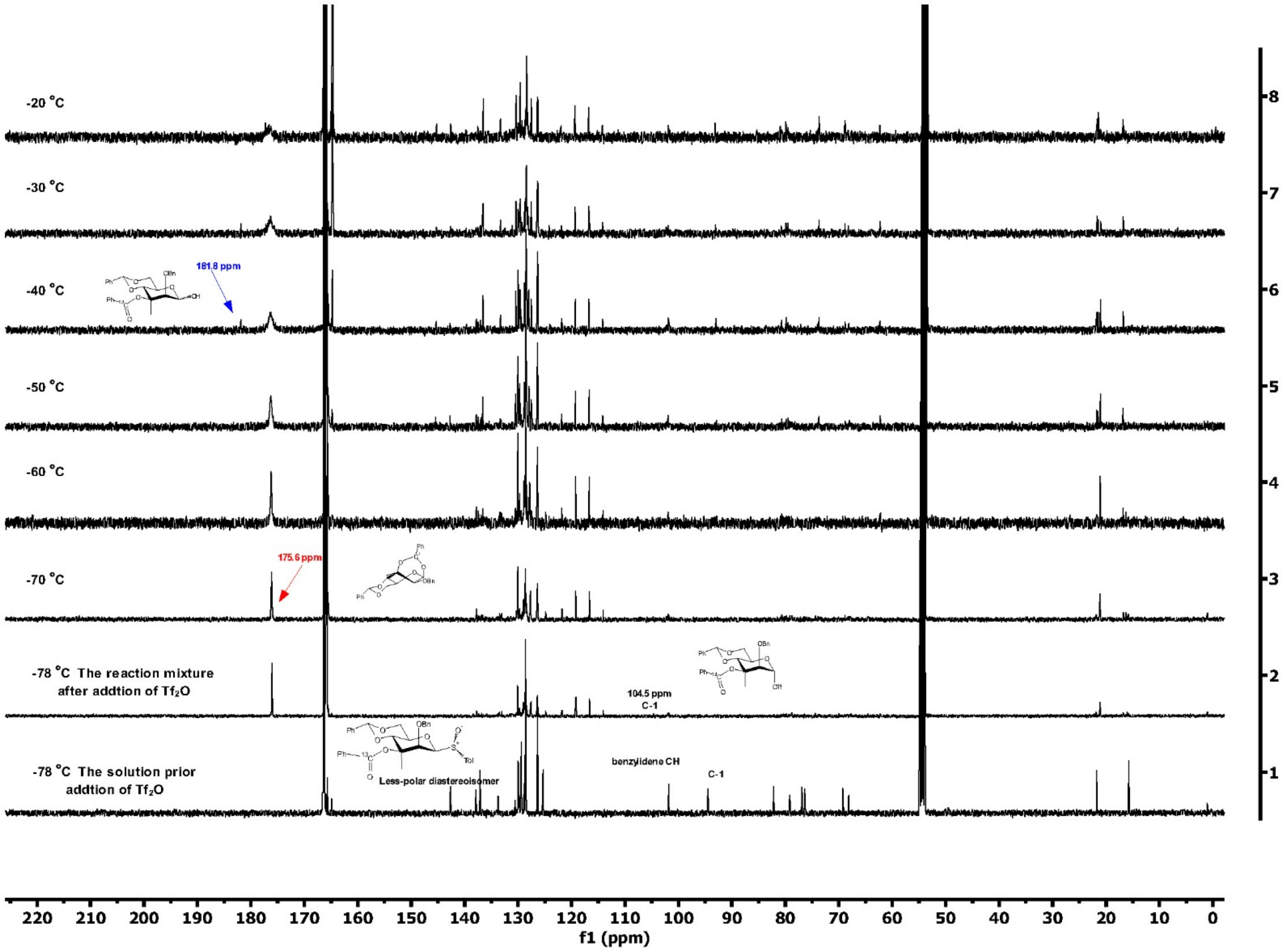

Note: This VT NMR study was conducted without TTBP (from -78 °C to 0 °C) according to Table 1. Entry 2.

Figure S207. VT ^{19}F NMR (471 MHz, CD_2Cl_2) study of *p*-methylphenyl 3-*O*-(benzoyl-α-^{13}C)-2-*O*-benzyl-4,6-*O*-benzylidene-3-*C*-methyl-thio-β-D-mannopyranoside *S*-oxide **^{13}C-40** (Less-polar diastereoisomer):

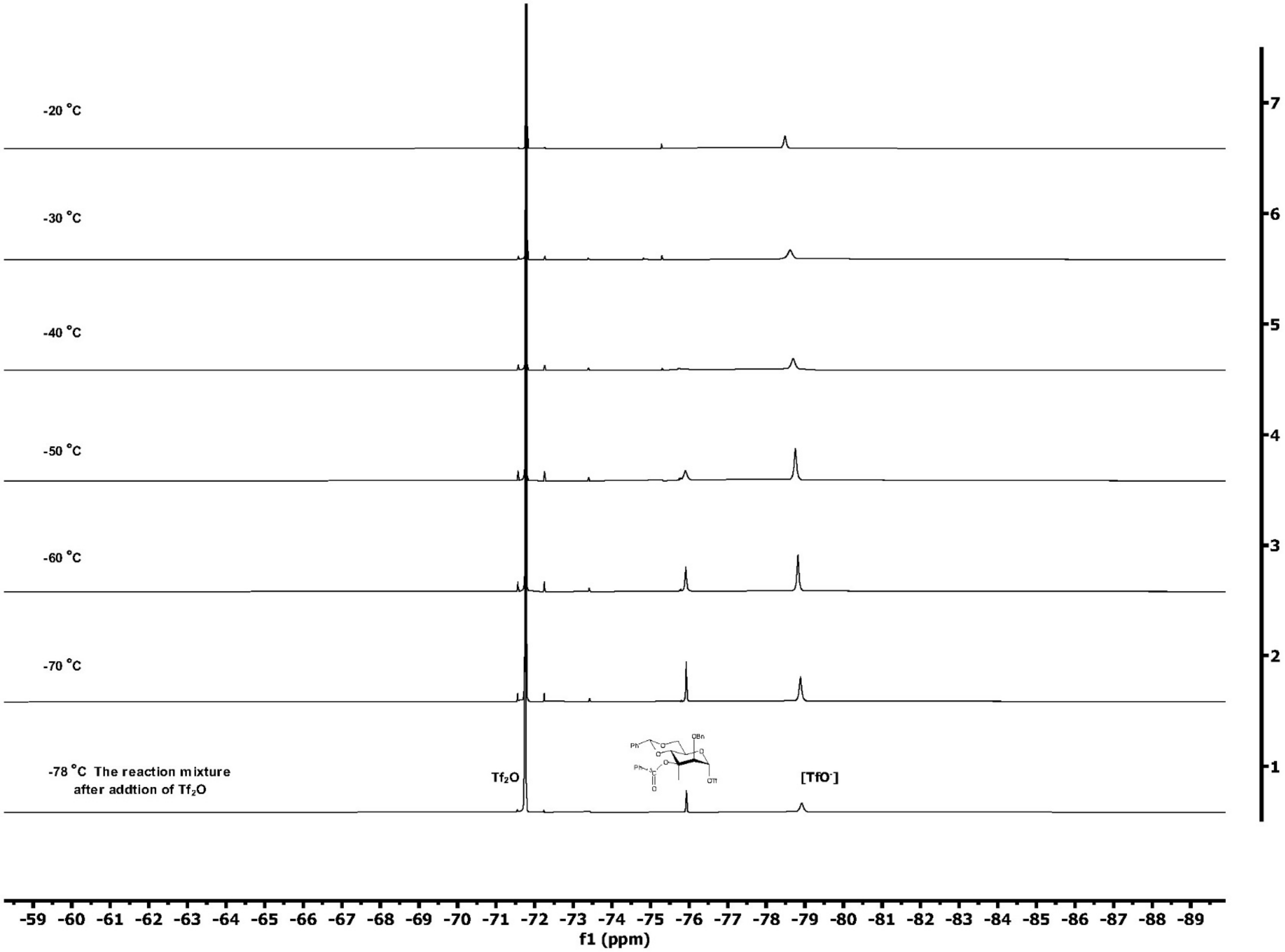

Note: This VT NMR study was conducted without TTBP (from -78 °C to 0 °C) according to Table 1. Entry 2.

Figure S208. VT ^{1}H NMR (500 MHz, CD_2Cl_2) study of *p*-methylphenyl 3-*O*-(benzoyl-α-^{13}C)-2-*O*-benzyl-4,6-*O*-benzylidene-3-*C*-methyl-thio-β-D-mannopyranoside *S*-oxide **^{13}C-40** (Less-polar diastereoisomer; HMBC at -60 °C):

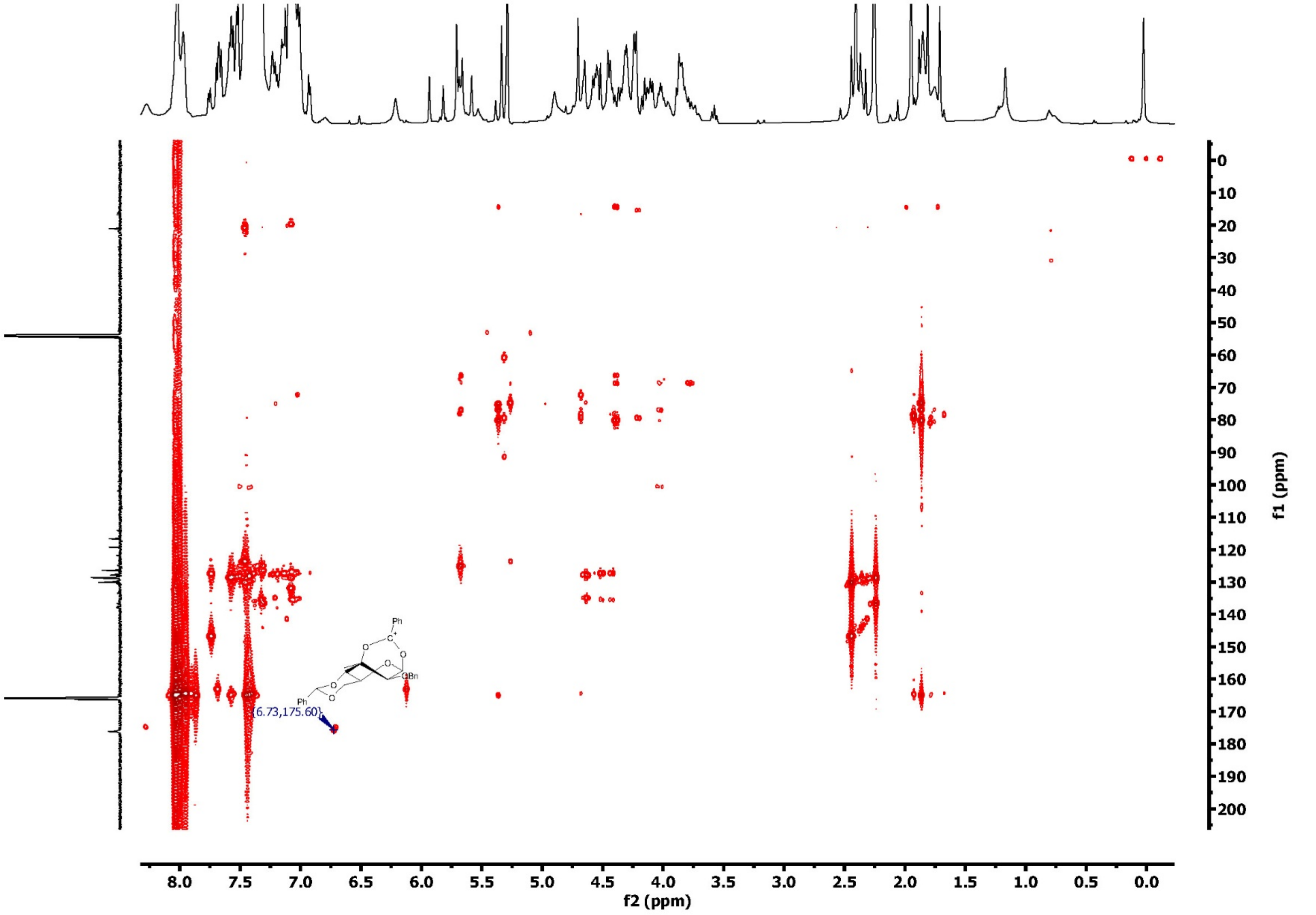

Note: This VT NMR study was conducted without TTBP (from -78 °C to 0 °C) according to Table 1. Entry 2.

Figure S209. VT ^{1}H NMR (500 MHz, CD_2Cl_2) study of *p*-methylphenyl 3-*O*-(benzoyl-α-^{13}C)-2-*O*-benzyl-4,6-*O*-benzylidene-3-*C*-methyl-thio-β-D-mannopyranoside *S*-oxide **^{13}C-40** (Polar diastereoisomer):

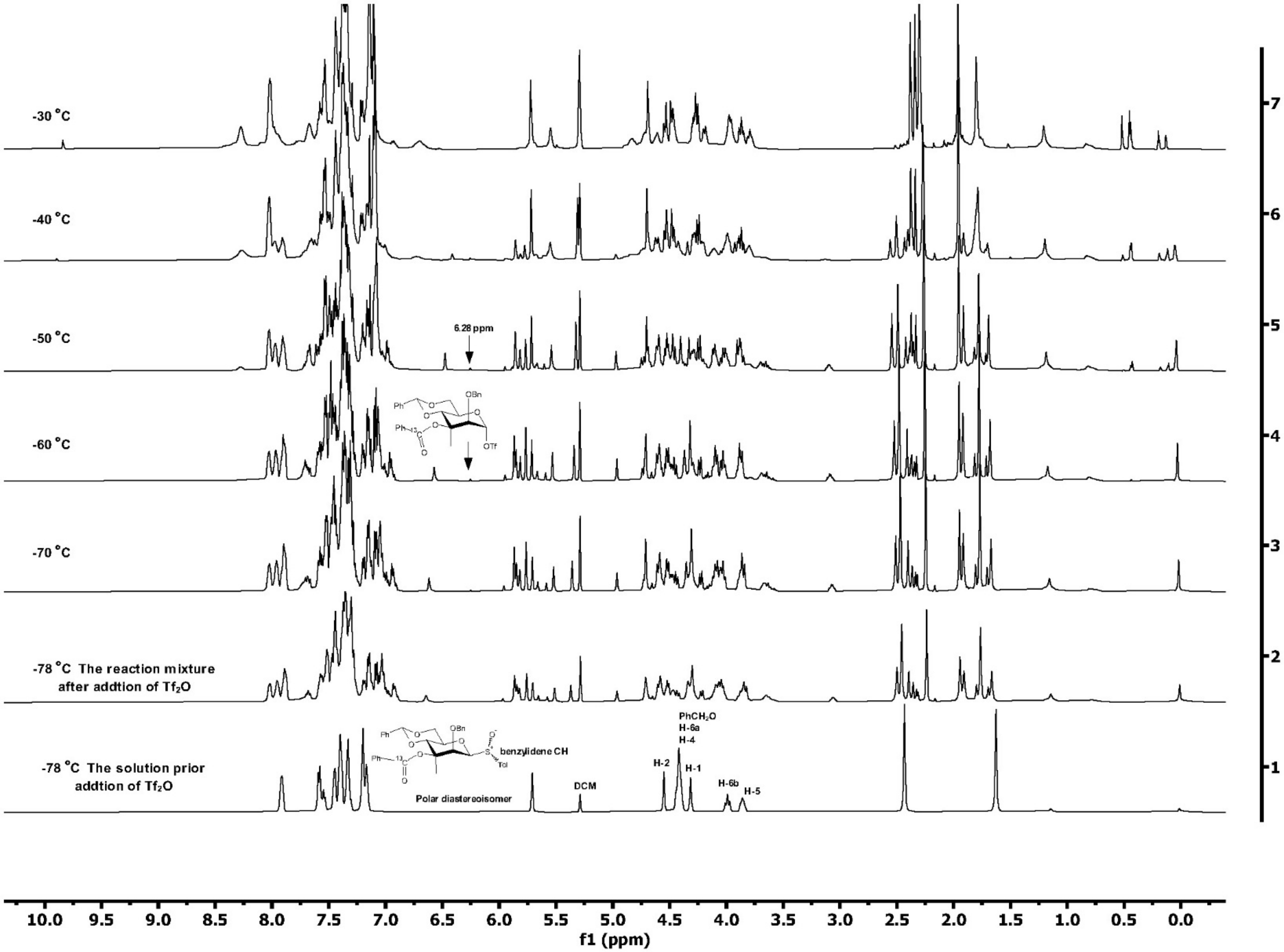

Note: This VT NMR study was conducted without TTBP (from -78 °C to -30 °C) according to Table 1. Entry 2.

Figure S210. VT $^{13}C\{^1H\}$ NMR (126 MHz, CD_2Cl_2) study of *p*-methylphenyl 3-*O*-(benzoyl-α-^{13}C)-2-*O*-benzyl-4,6-*O*-benzylidene-3-*C*-methyl-thio-β-D-mannopyranoside *S*-oxide **^{13}C-40** (Polar-diastereoisomer):

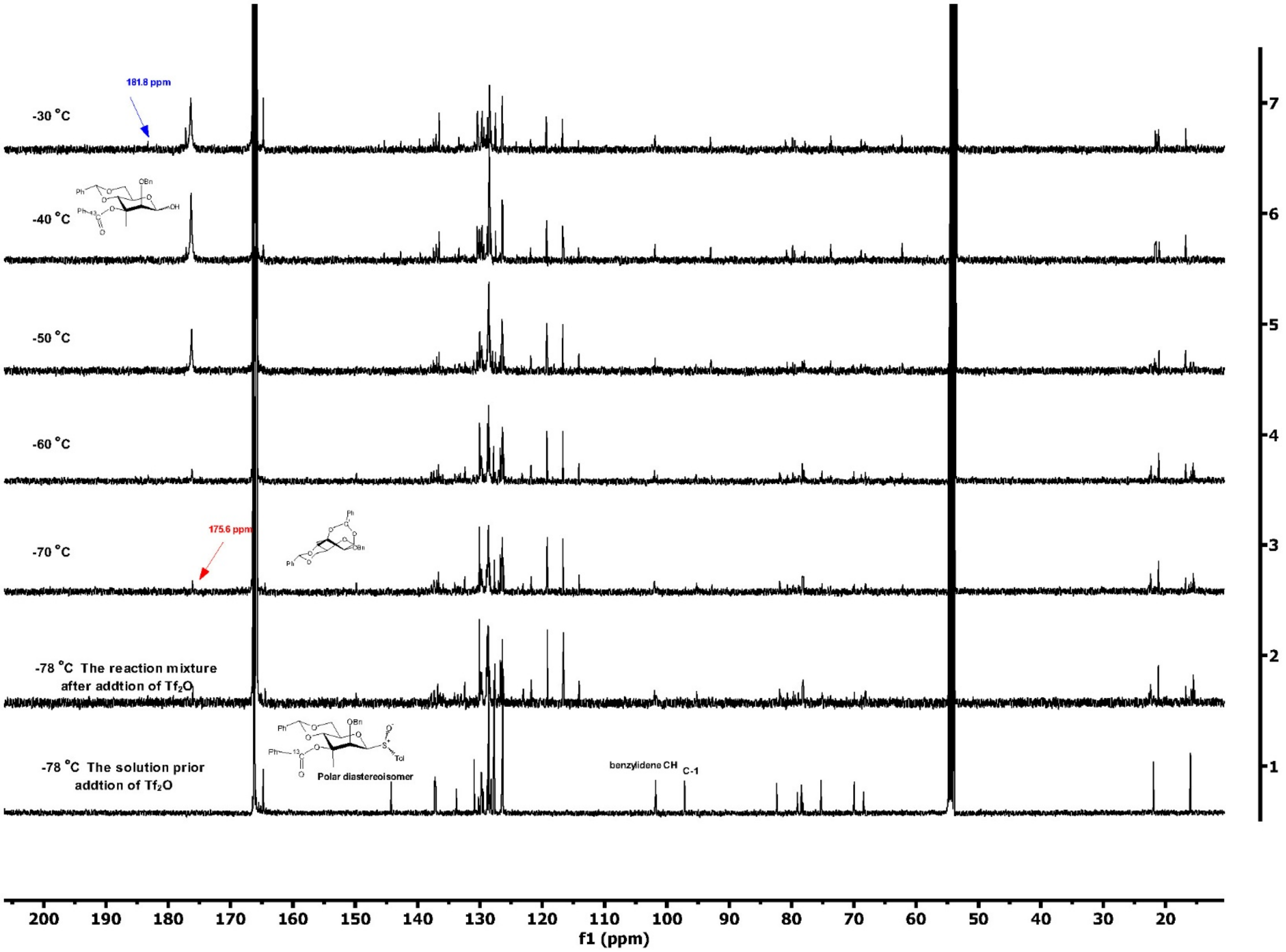

Note: This VT NMR study was conducted without TTBP (from -78 °C to -30 °C) according to Table 1. Entry 2.

Figure S211. VT ^{19}F NMR (471 MHz, CD_2Cl_2) study of *p*-methylphenyl 3-*O*-(benzoyl-α-^{13}C)-2-*O*-benzyl-4,6-*O*-benzylidene-3-*C*-methyl-thio-β-D-mannopyranoside *S*-oxide **^{13}C-40** (Polar diastereoisomer):

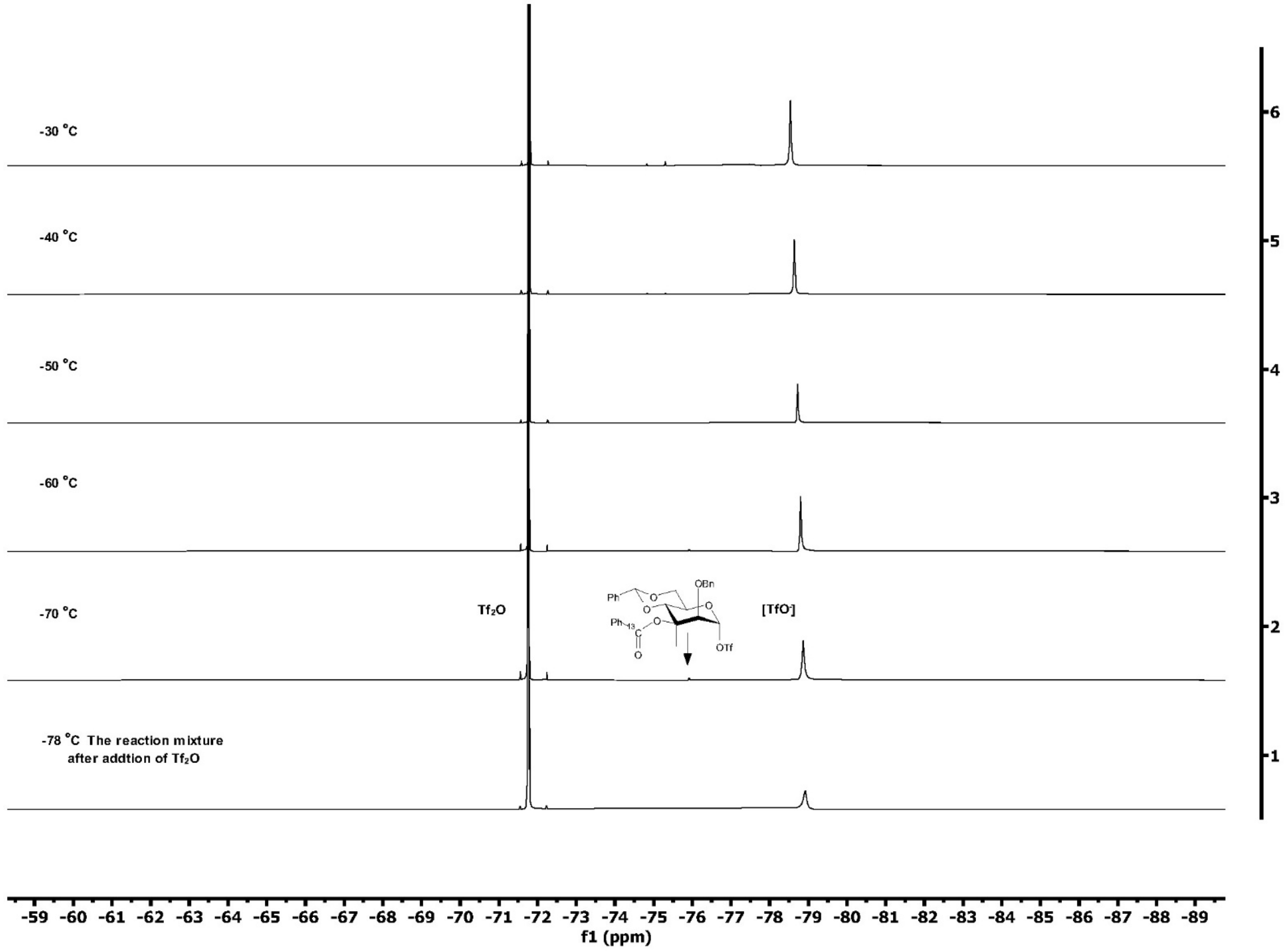

Note: This VT NMR study was conducted without TTBP (from -78 °C to -30 °C) according to Table 1. Entry 2.

Figure S212. VT ^{1}H NMR (500 MHz, CD_2Cl_2) study of *p*-methylphenyl 3-*O*-(benzoyl-α-^{13}C)-2-*O*-benzyl-4,6-*O*-benzylidene-3-*C*-methyl-thio-β-D-mannopyranoside *S*-oxide **^{13}C-40** (Polar diastereoisomer; HMBC at -30 °C):

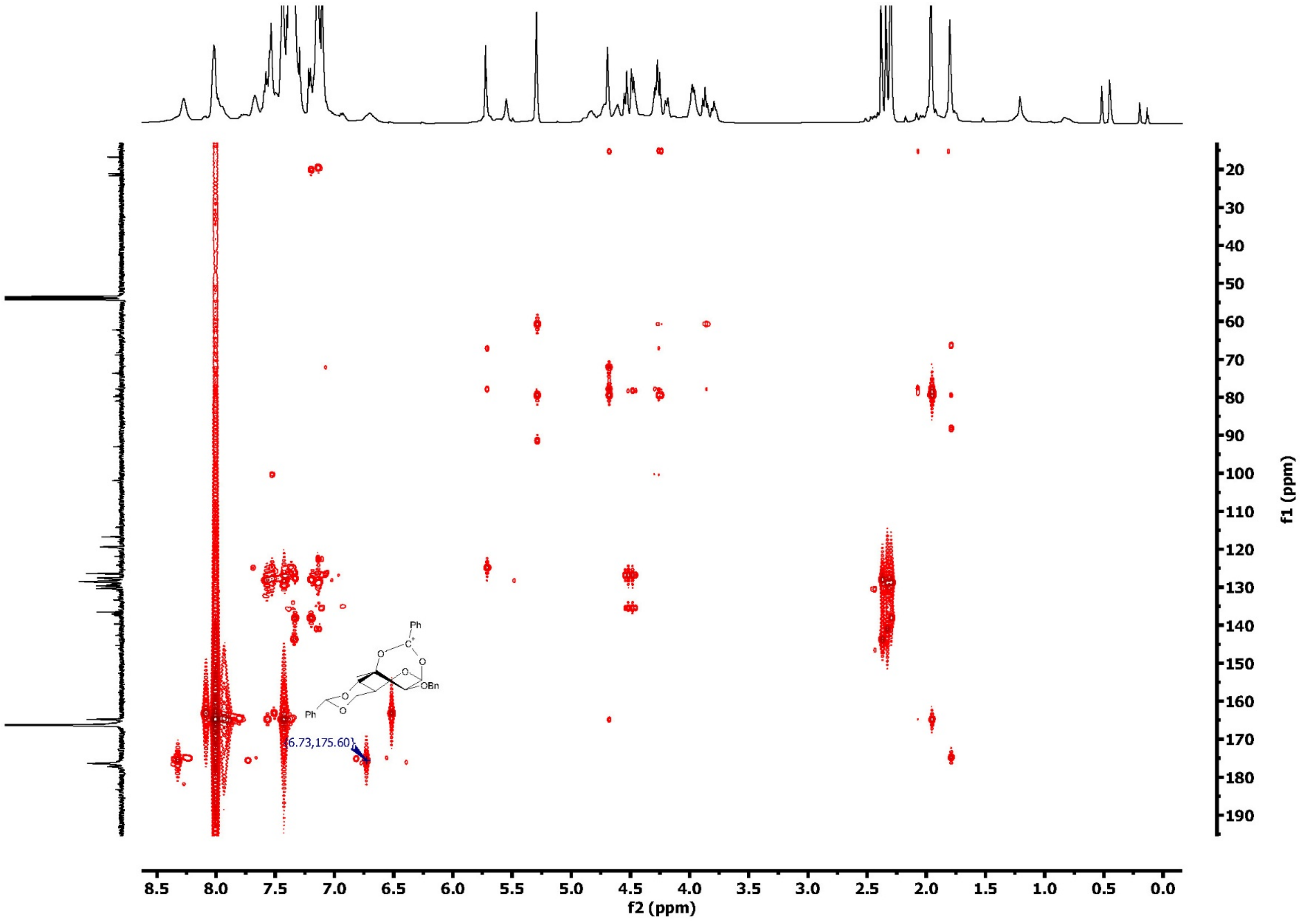

Note: This VT NMR study was conducted without TTBP (from -78 °C to -30 °C) according to Table 1. Entry 2.

Figure S213. VT ^{1}H NMR (500 MHz, CD_2Cl_2) study of *p*-methylphenyl 3-*O*-(benzoyl-α-^{13}C)-2-*O*-benzyl-4,6-*O*-benzylidene-3-*C*-methyl-thio-α-D-mannopyranoside *S*-oxide **^{13}C-46**:

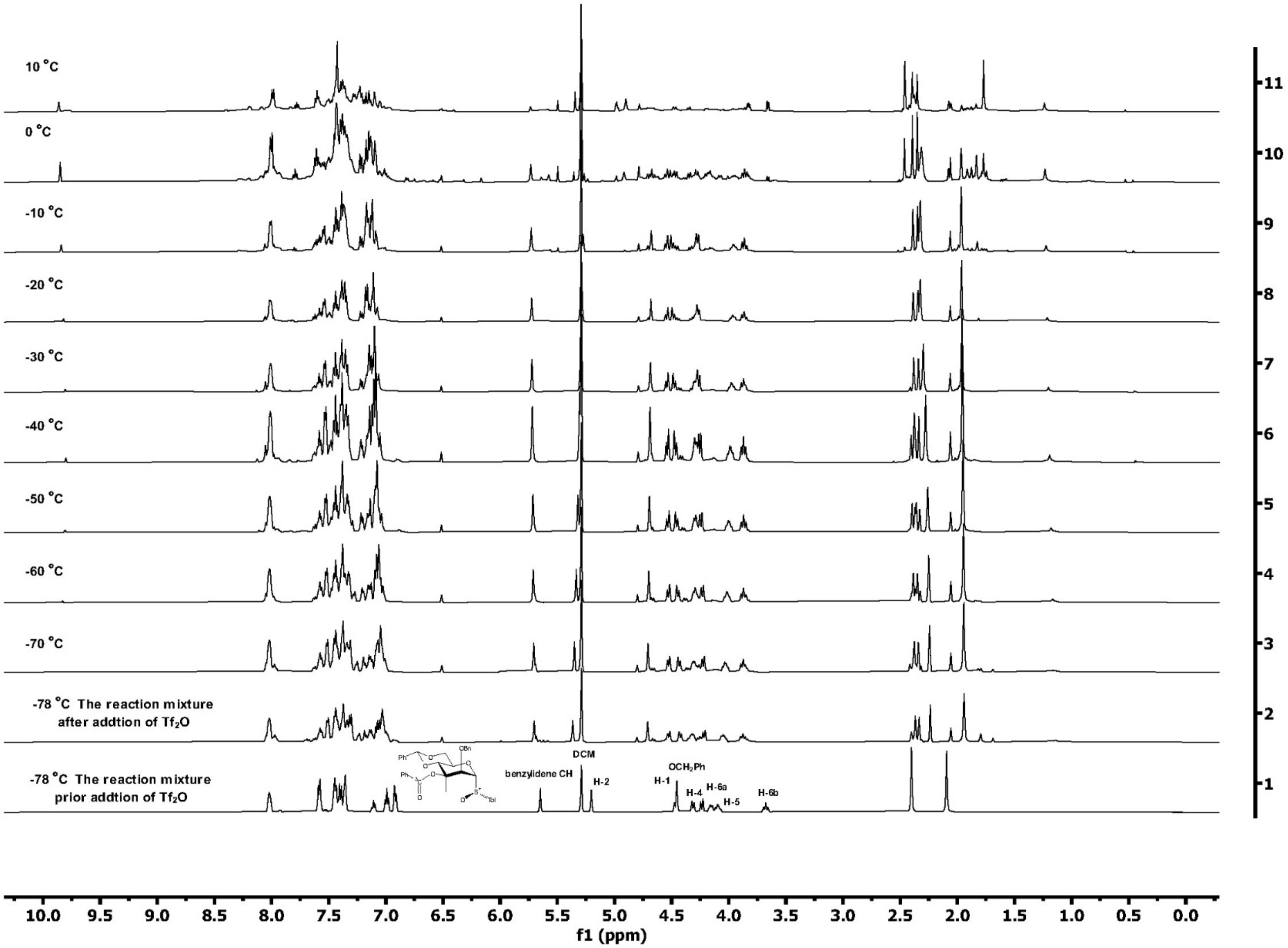

Note: This VT NMR study was conducted without TTBP (from -78 °C to 10 °C) according to Table 1. Entry 2.

Figure S214. VT $^{13}C\{^1H\}$ NMR (126 MHz, CD_2Cl_2) study of *p*-methylphenyl 3-*O*-(benzoyl-α-^{13}C)-2-*O*-benzyl-4,6-*O*-benzylidene-3-*C*-methyl-thio-α-D-mannopyranoside *S*-oxide **^{13}C-46**:

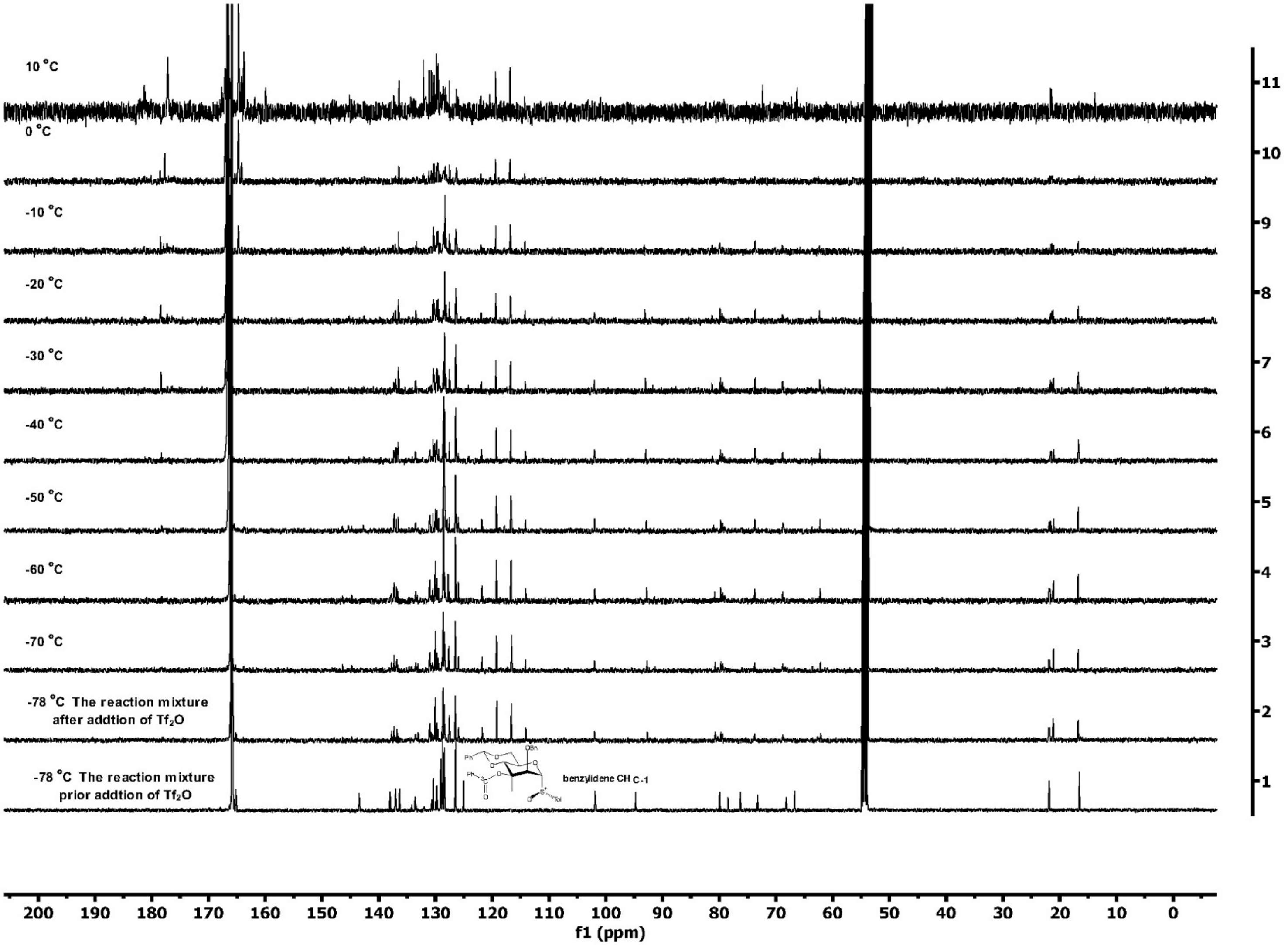

Note: This VT NMR study was conducted without TTBP (from -78 °C to 10 °C) according to Table 1. Entry 2.

Figure S215. VT ^{19}F NMR (471 MHz, CD_2Cl_2) study of *p*-methylphenyl 3-*O*-(benzoyl-α-^{13}C)-2-*O*-benzyl-4,6-*O*-benzylidene-3-*C*-methyl-thio-α-D-mannopyranoside *S*-oxide **^{13}C-46**:

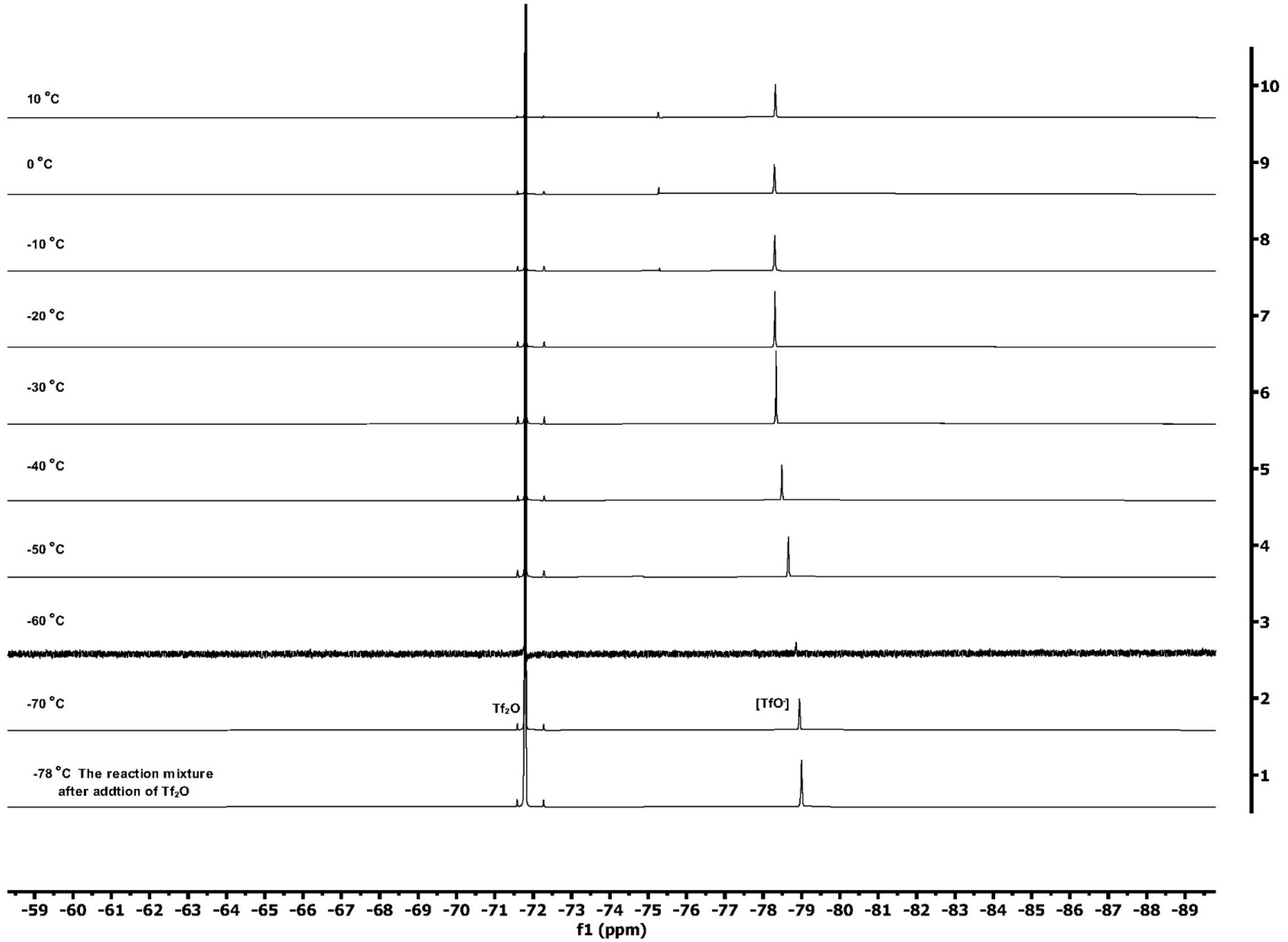

Note: This VT NMR study was conducted without TTBP (from -78 °C to 10 °C) according to Table 1. Entry 2.

Figure S216. VT ^{1}H NMR (500 MHz, CD_2Cl_2) study of *p*-methylphenyl 3-*O*-(benzoyl-α-^{13}C)-2-*O*-benzyl-4,6-*O*-benzylidene-3-*C*-methyl-thio-α-D-mannopyranoside *S*-oxide **^{13}C-46** (HMBC at -30 °C):

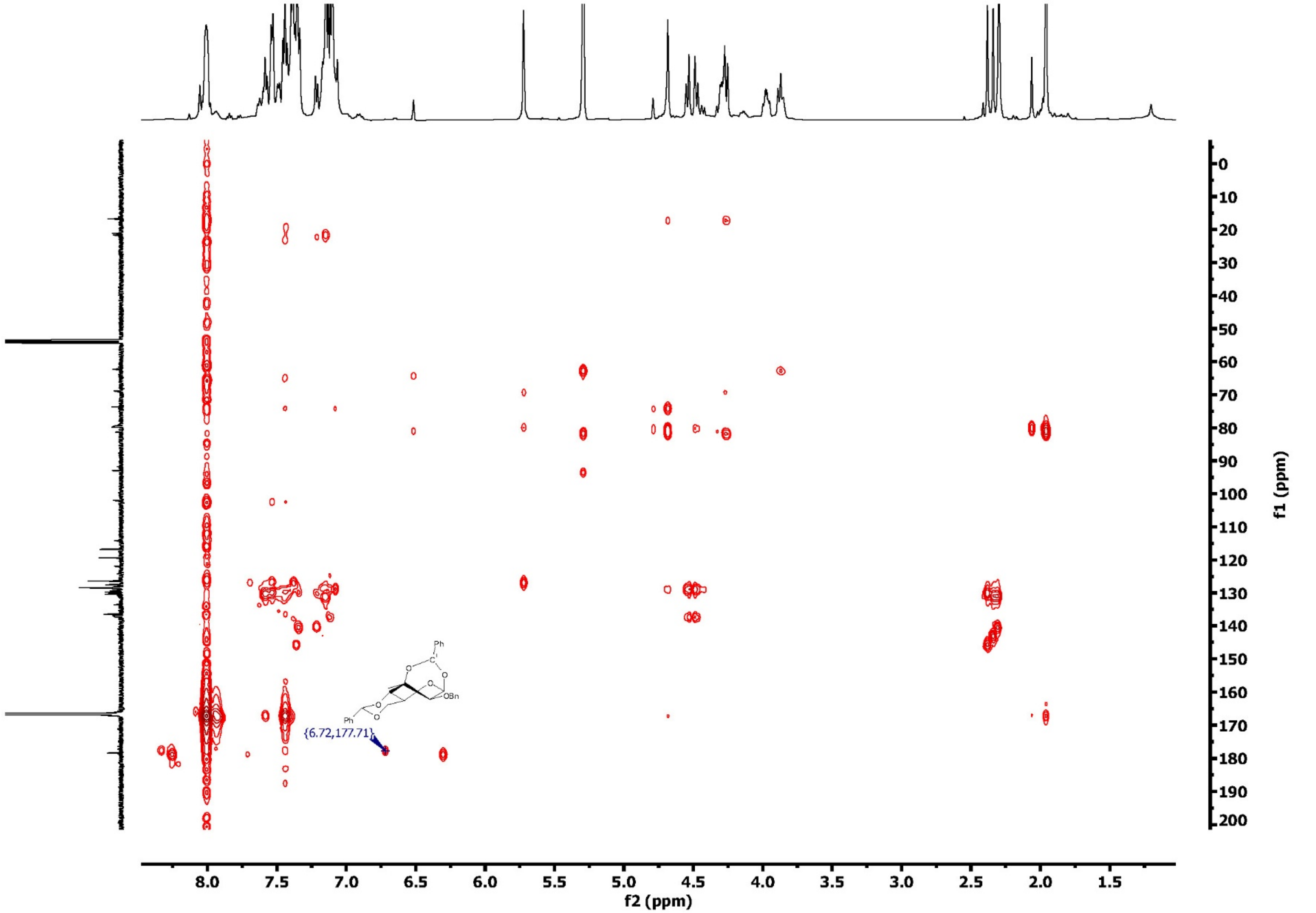

Note: This VT NMR study was conducted without TTBP (from -78 °C to 10 °C) according to Table 1. Entry 2.

Figure S217. VT ^{1}H NMR (500 MHz, CD_2Cl_2) study of *p*-methylphenyl 3-*O*-(benzoyl-α-^{13}C)-2-*O*-benzyl-4,6-*O*-benzylidene-3-*C*-methyl-thio-β-D-mannopyranoside *S*-oxides **^{13}C-40** (Less-polar and polar diastereoisomers) and *p*-methylphenyl 3-*O*-(benzoyl-α-^{13}C)-2-*O*-benzyl-4,6-*O*-benzylidene-3-*C*-methyl-thio-α-D-mannopyranoside *S*-oxide **^{13}C-46** (protons stacked at -30 °C):

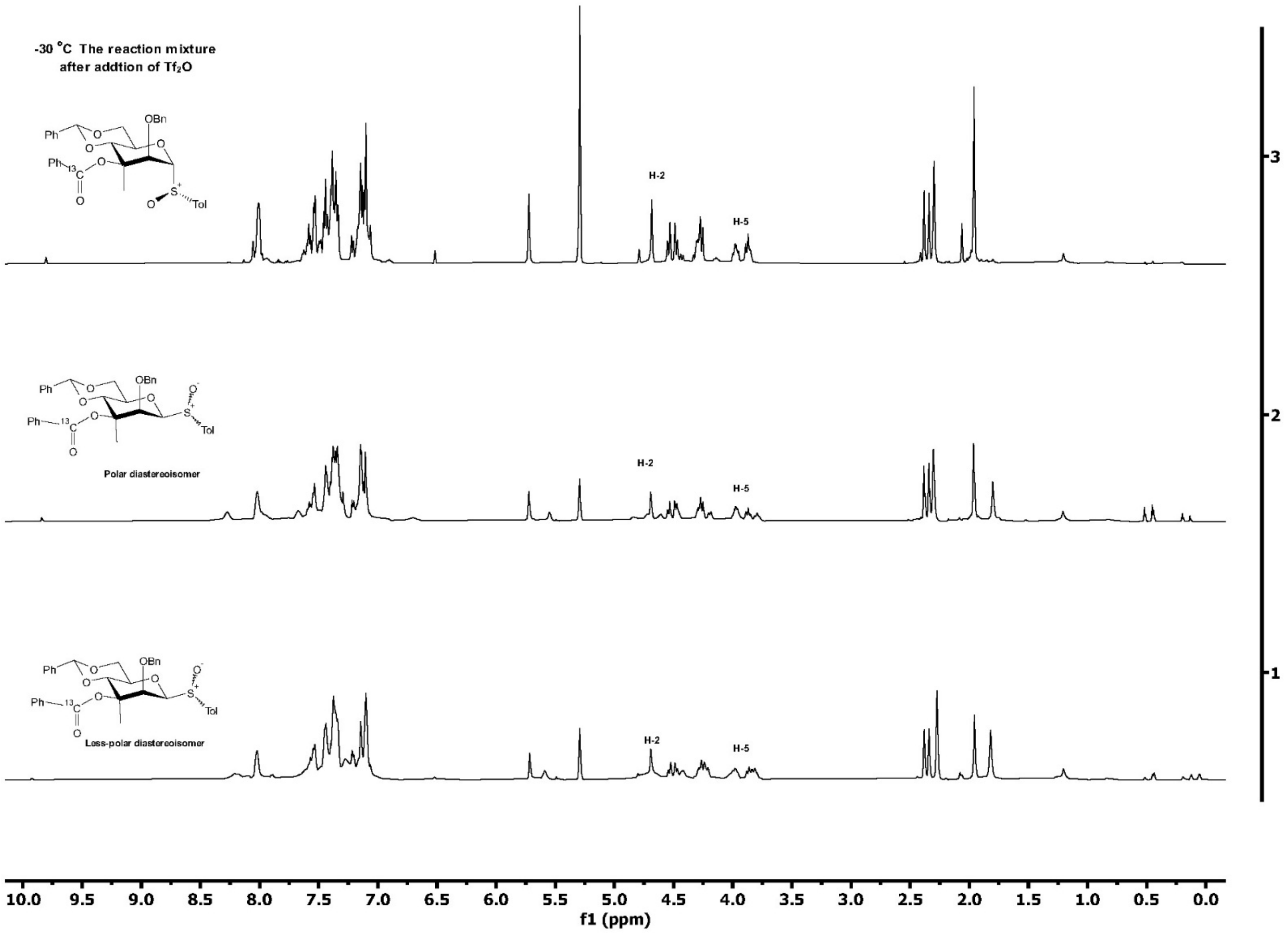

Note: This VT NMR study was conducted without TTBP (from -78 °C to 10 °C) according to Table 1. Entry 2.

Figure S218. VT $^{13}C\{^{1}H\}$ NMR (126 MHz, CD_2Cl_2) study of *p*-methylphenyl 3-*O*-(benzoyl-α-^{13}C)-2-*O*-benzyl-4,6-*O*-benzylidene-3-*C*-methyl-thio-β-D-mannopyranoside *S*-oxides **^{13}C-40** (Less-polar and polar diastereoisomers) and *p*-methylphenyl 3-*O*-(benzoyl-α-^{13}C)-2-*O*-benzyl-4,6-*O*-benzylidene-3-*C*-methyl-thio-α-D-mannopyranoside *S*-oxide **^{13}C-46** (carbons stacked at -30 °C):

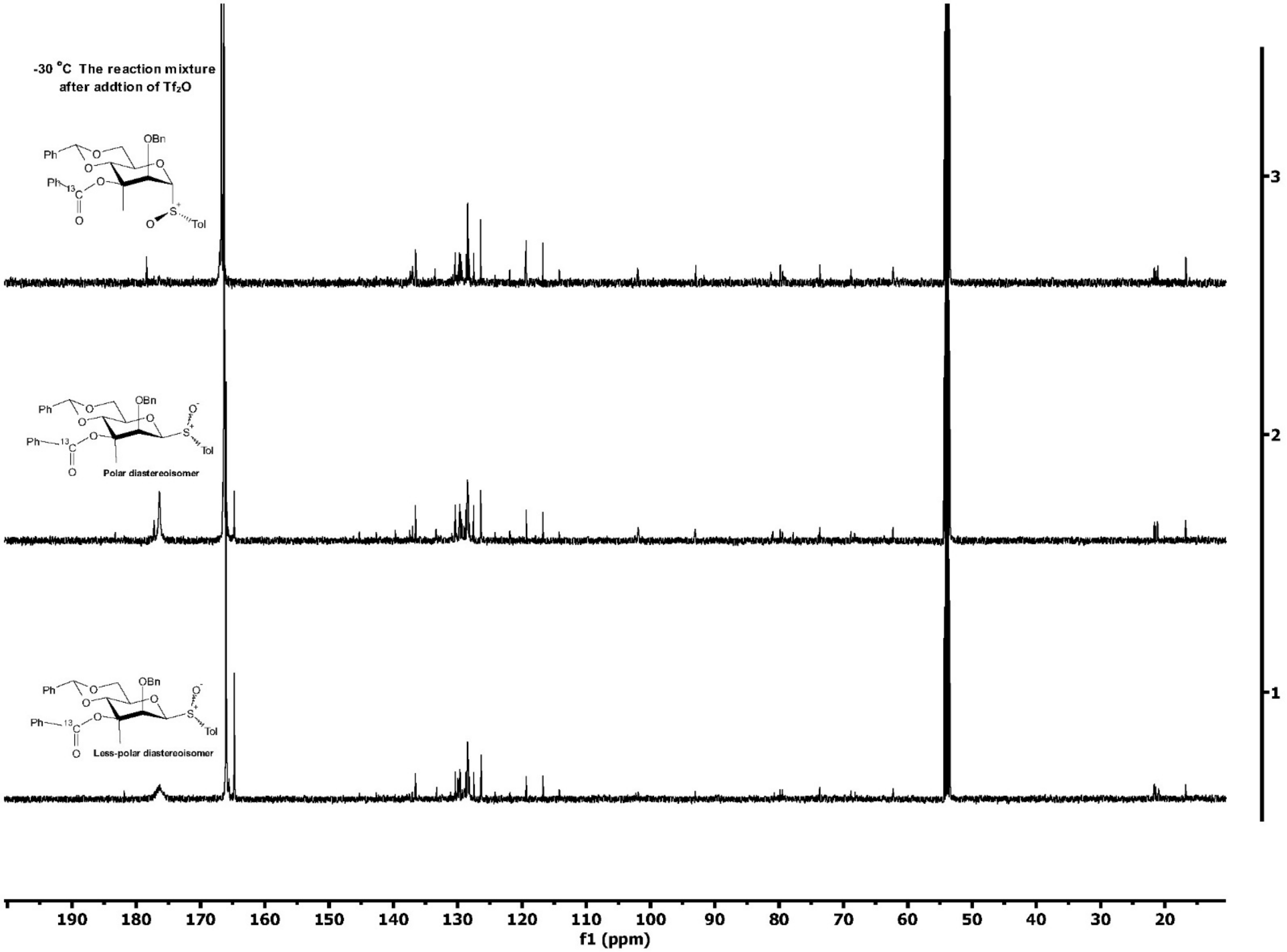

Note: This VT NMR study was conducted without TTBP (from -78 °C to 10 °C) according to Table 1. Entry 2.

Figure S219. VT $^{13}C\{^{1}H\}$ NMR (126 MHz, CD_2Cl_2) study of *p*-methylphenyl 3-*O*-benzoyl-2-*O*-benzyl-4,6-*O*-benzylidene-3-*C*-methyl-thio-β-D-mannopyranoside *S*-oxide **40** (Less-polar diastereoisomer):

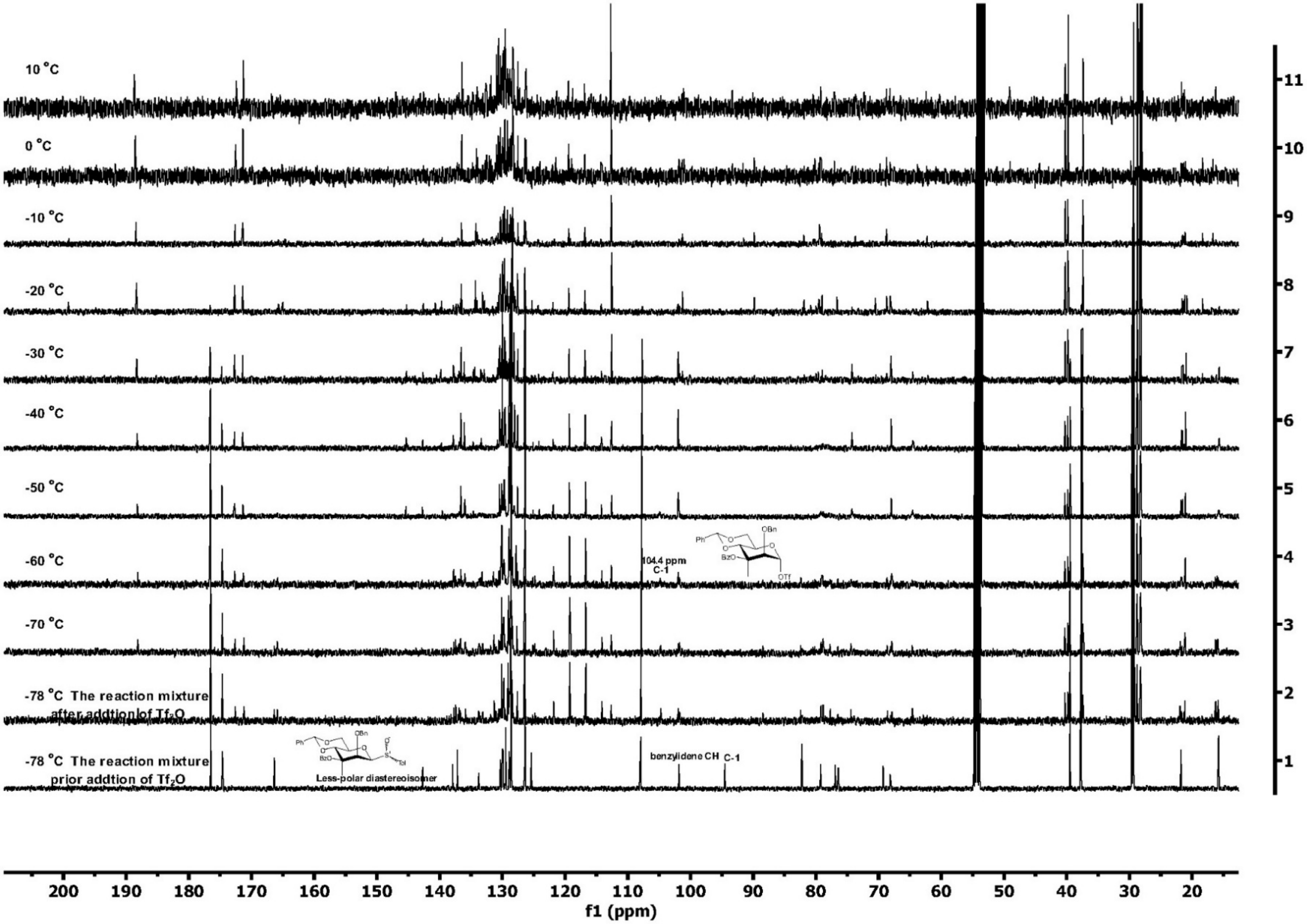

Figure S220. VT ^{1}H NMR (500 MHz, CD_2Cl_2) study of *p*-methylphenyl 3-*O*-(benzoyl-α-^{13}C)-2-*O*-benzyl-4,6-*O*-benzylidene-thio-α-D-mannopyranoside **^{13}C-48**:

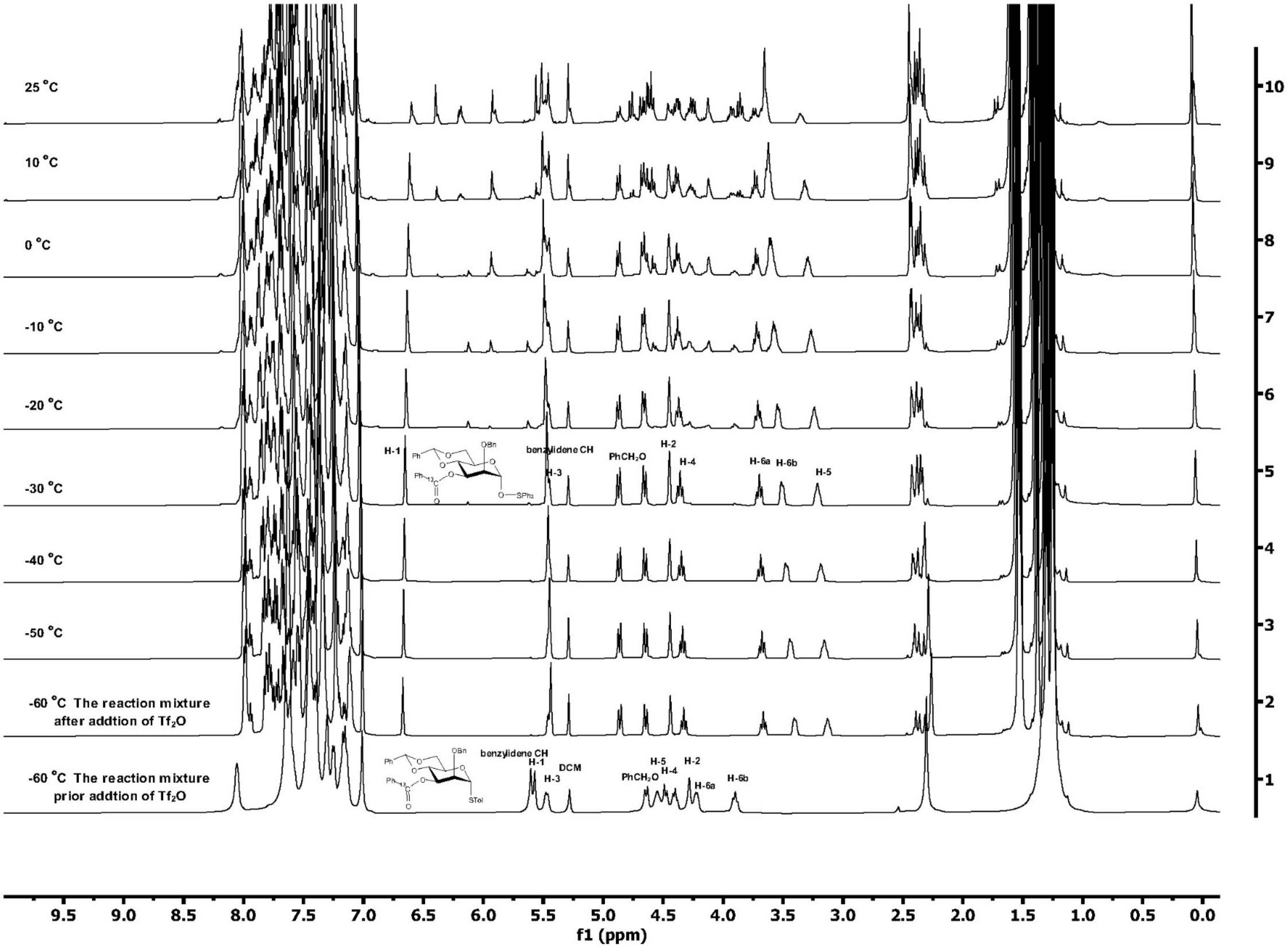

Figure S221. VT $^{13}C\{^1H\}$ NMR (126 MHz, CD_2Cl_2) study of *p*-methylphenyl 3-*O*-(benzoyl-α-^{13}C)-2-*O*-benzyl-4,6-*O*-benzylidene-thio-α-D-mannopyranoside **^{13}C-48**:

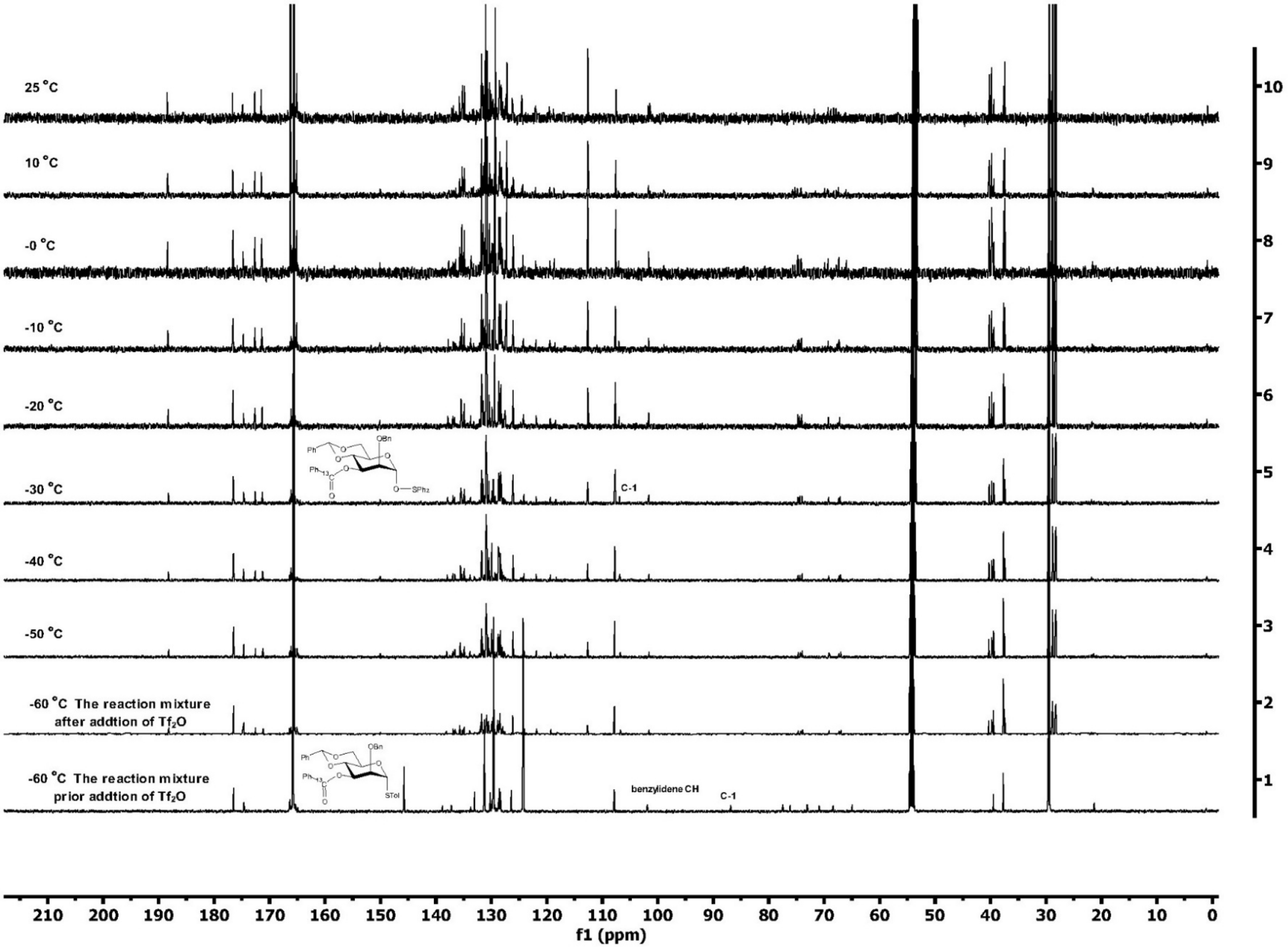

Figure S222. VT ^{19}F NMR (471 MHz, CD_2Cl_2) study of *p*-methylphenyl 3-*O*-(benzoyl-α-^{13}C)-2-*O*-benzyl-4,6-*O*-benzylidene-thio-α-D-mannopyranoside **^{13}C-48**:

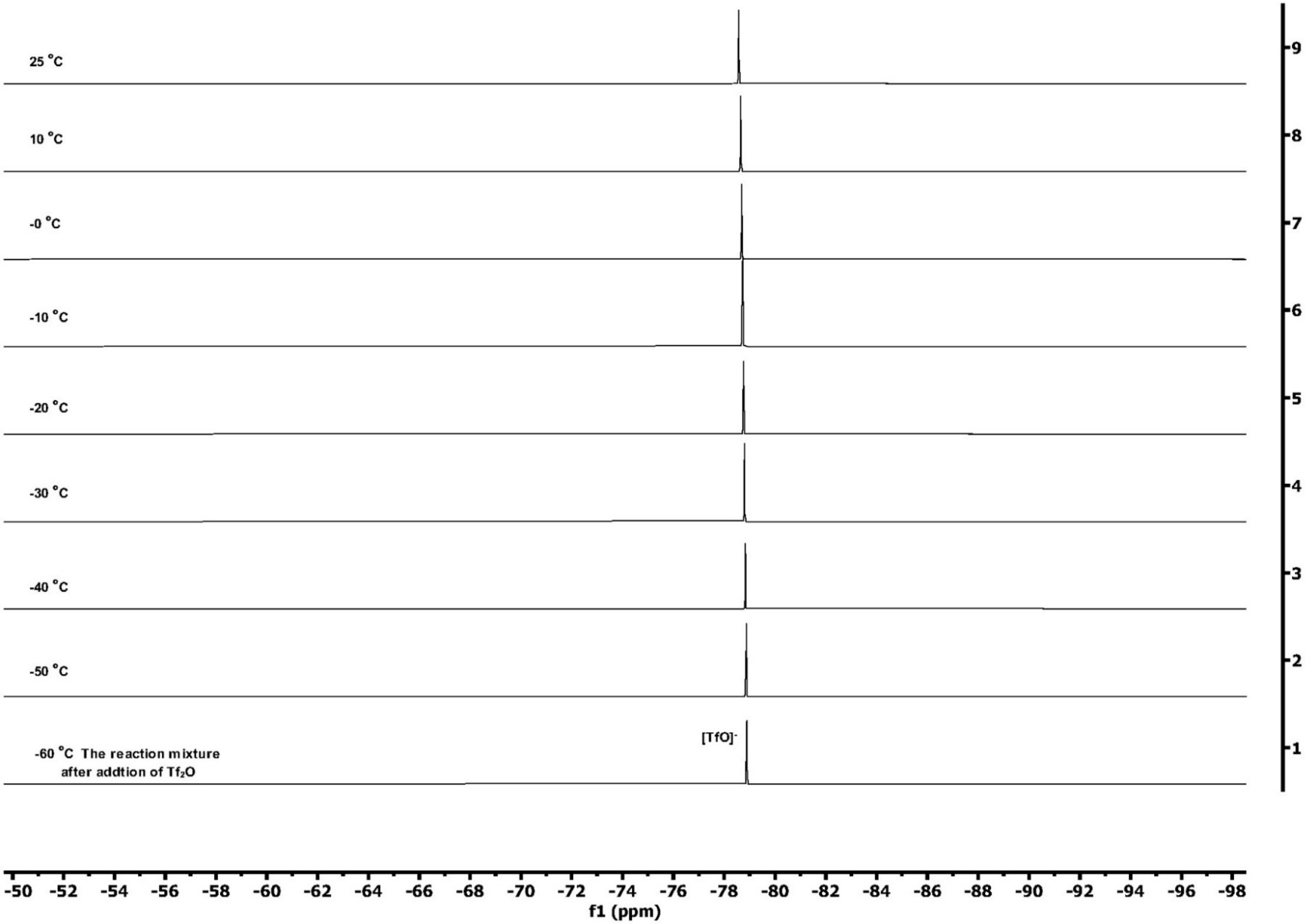

Figure S223. VT ^{1}H NMR (500 MHz, CD_2Cl_2) study of *p*-methylphenyl 3-*O*-(benzoyl-α-^{13}C)-2-*O*-benzyl-4,6-*O*-benzylidene-thio-α-D-mannopyranoside **^{13}C-48** (COSY at -60 °C):

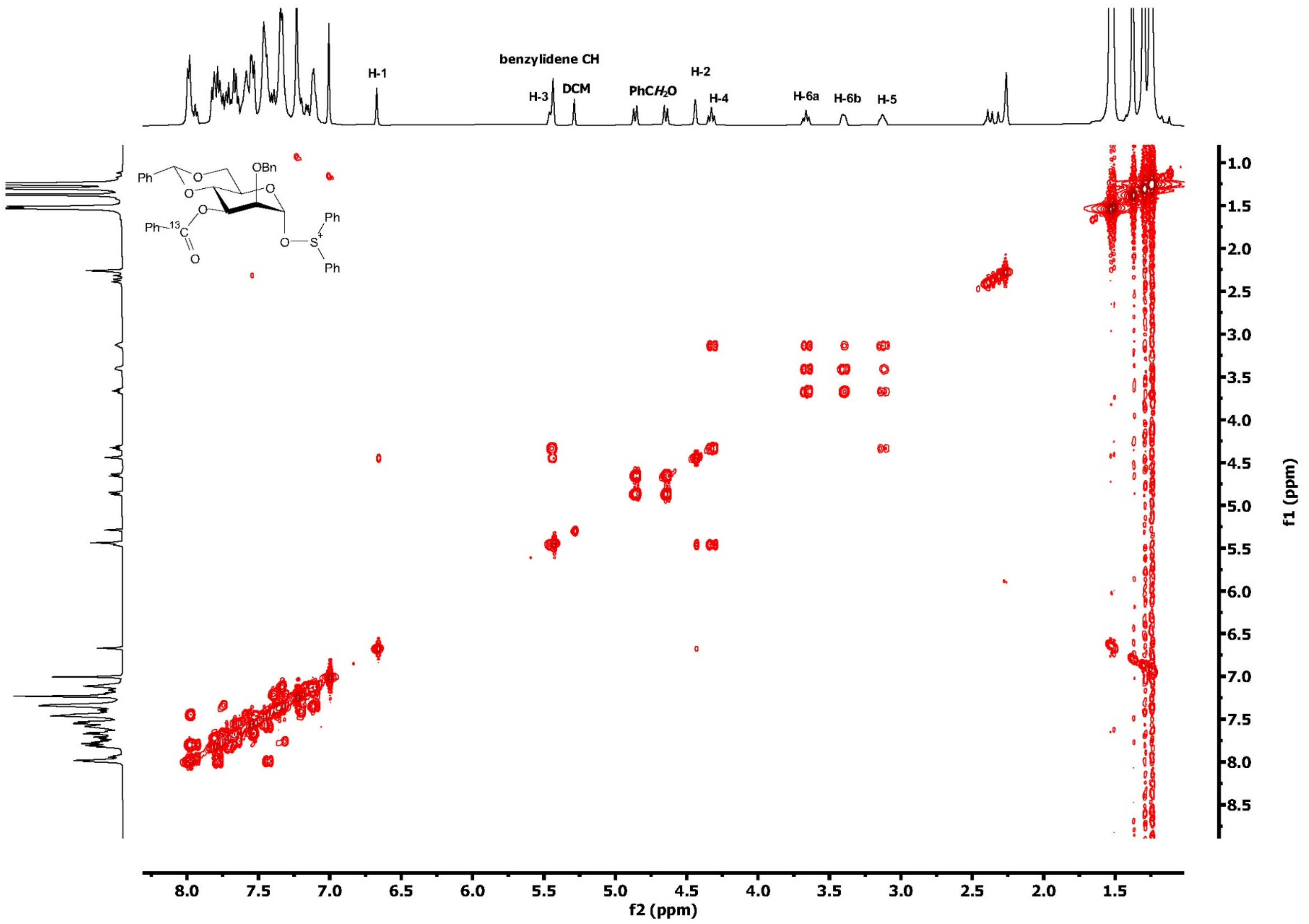

Figure S224. VT ^{1}H NMR (500 MHz, CD_2Cl_2) study of *p*-methylphenyl 3-*O*-(benzoyl-α-^{13}C)-2-*O*-benzyl-4,6-*O*-benzylidene-thio-α-D-mannopyranoside **^{13}C-48** (HSQC at -60 °C):

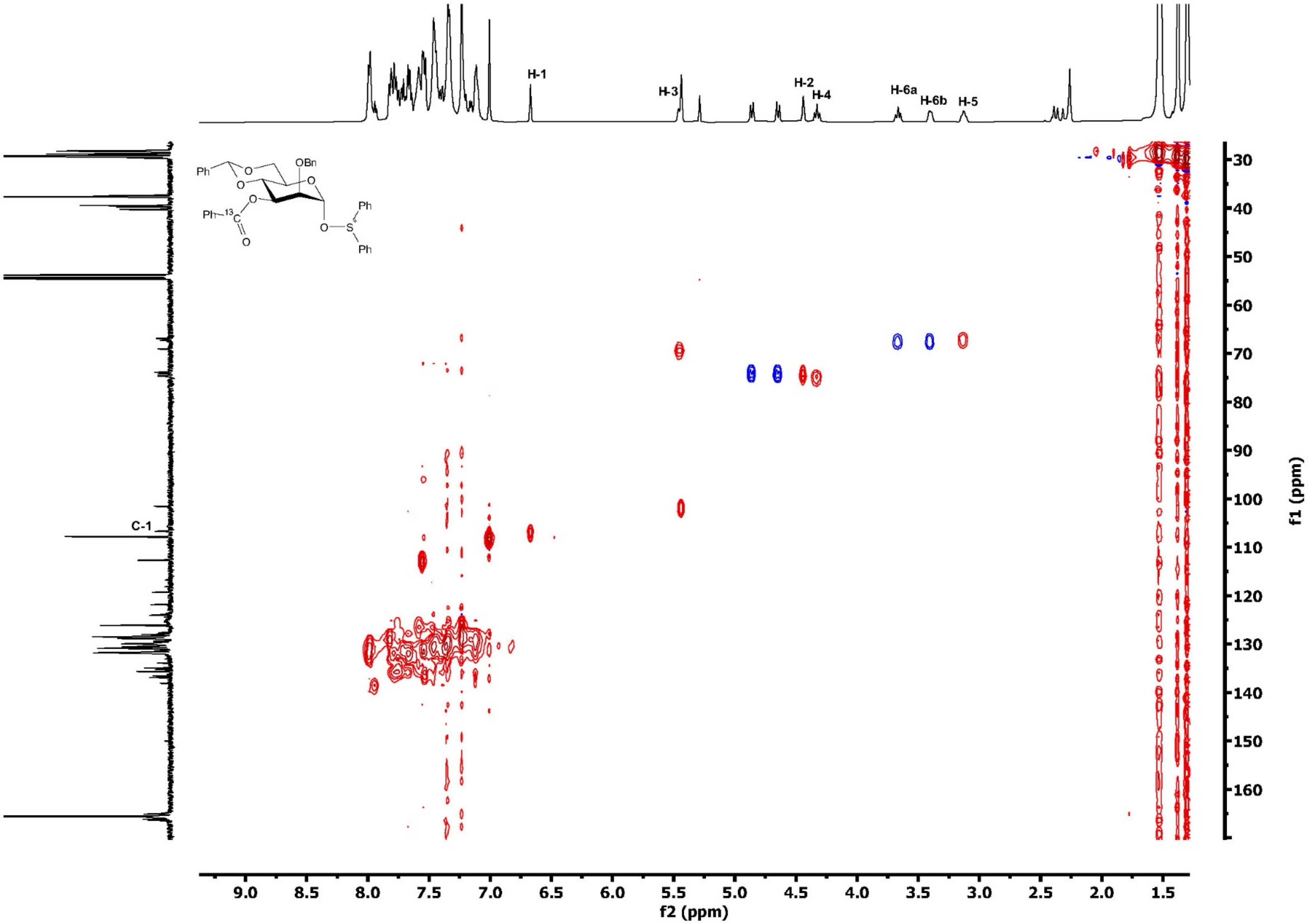

Figure S225. VT $^{13}C\{^1H\}$ NMR (126 MHz, CD_2Cl_2) study of *p*-methylphenyl 3-*O*-(benzoyl-α-^{13}C)-2-*O*-benzyl-4,6-*O*-benzylidene-thio-α-D-mannopyranoside **^{13}C-48** ($^{13}C\{^1H\}$ DEPT at -60 °C):

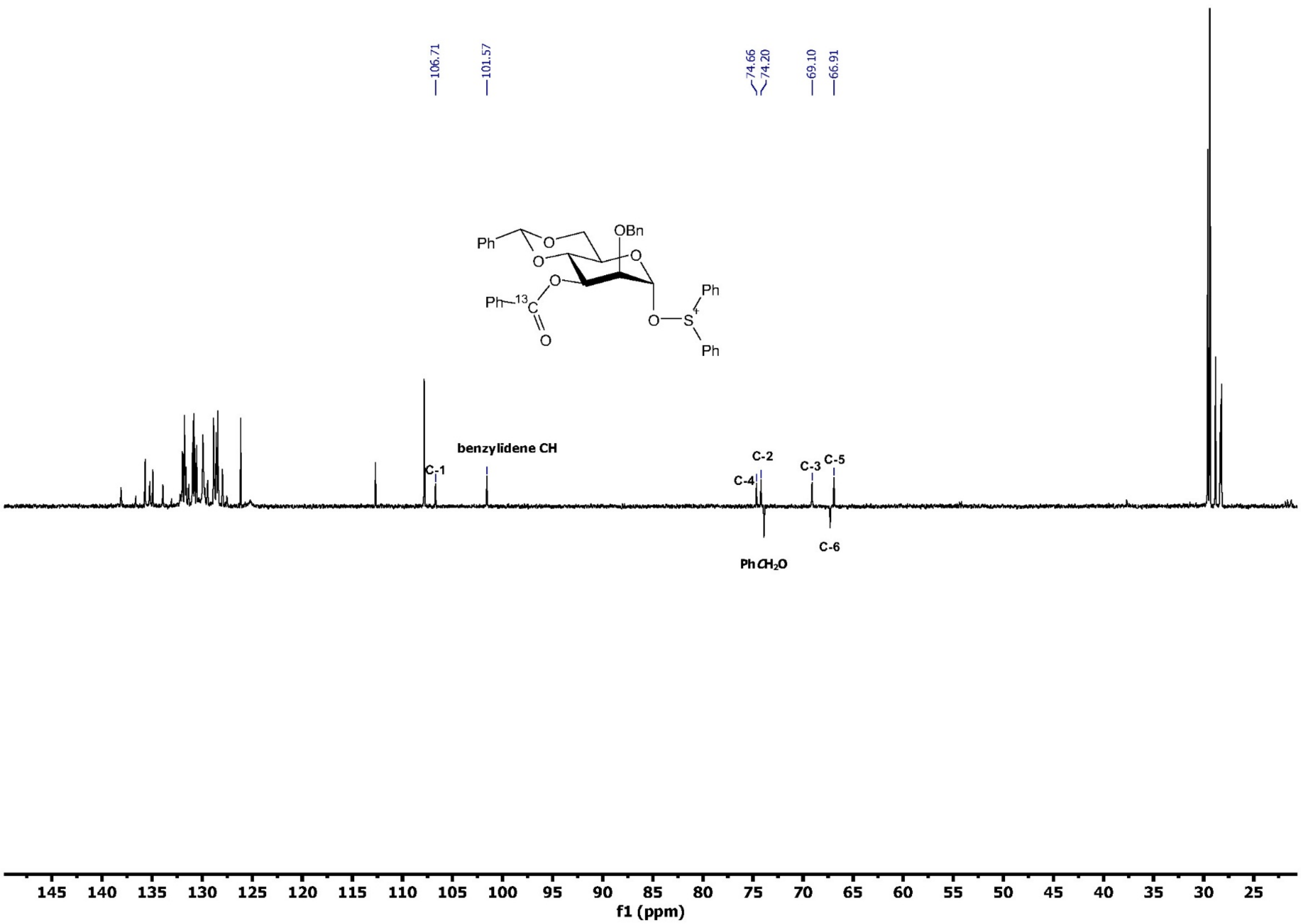

Figure S226. VT $^{13}C\{H\}$ NMR (126 MHz, CD_2Cl_2) study of *p*-methylphenyl 3-*O*-(benzoyl-α-^{13}C)-2-*O*-benzyl-4,6-*O*-benzylidene-thio-α-D-mannopyranoside **^{13}C-48** (C-H coupled and decoupled at -60 °C):

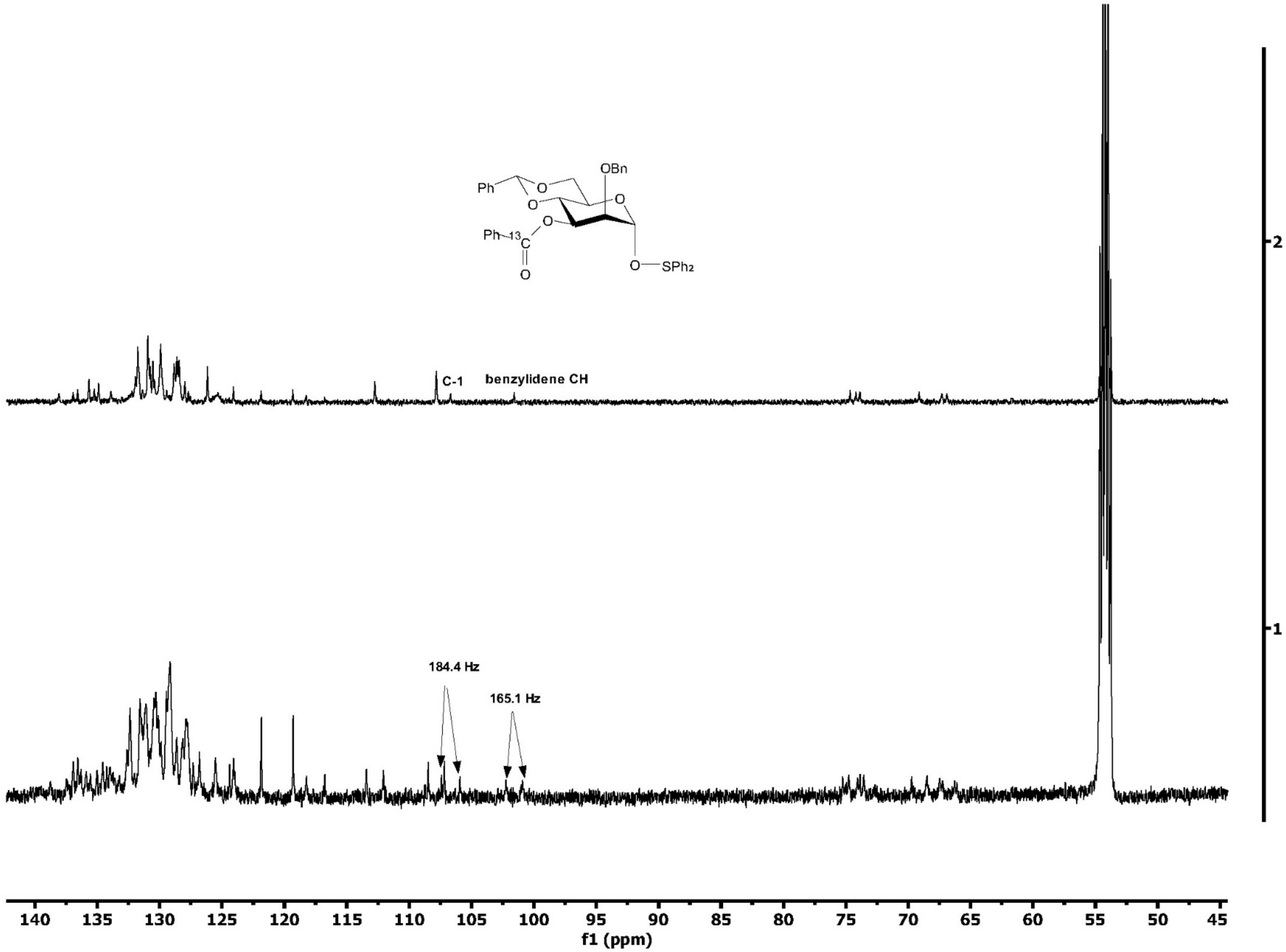

Figure S227. VT ^{1}H NMR (500 MHz, CD_2Cl_2) study of *p*-methylphenyl 3-*O*-(benzoyl-α-^{13}C)-2-*O*-benzyl-4,6-*O*-benzylidene-3-*C*-methyl-thio-β-D-mannopyranoside **^{13}C-38**:

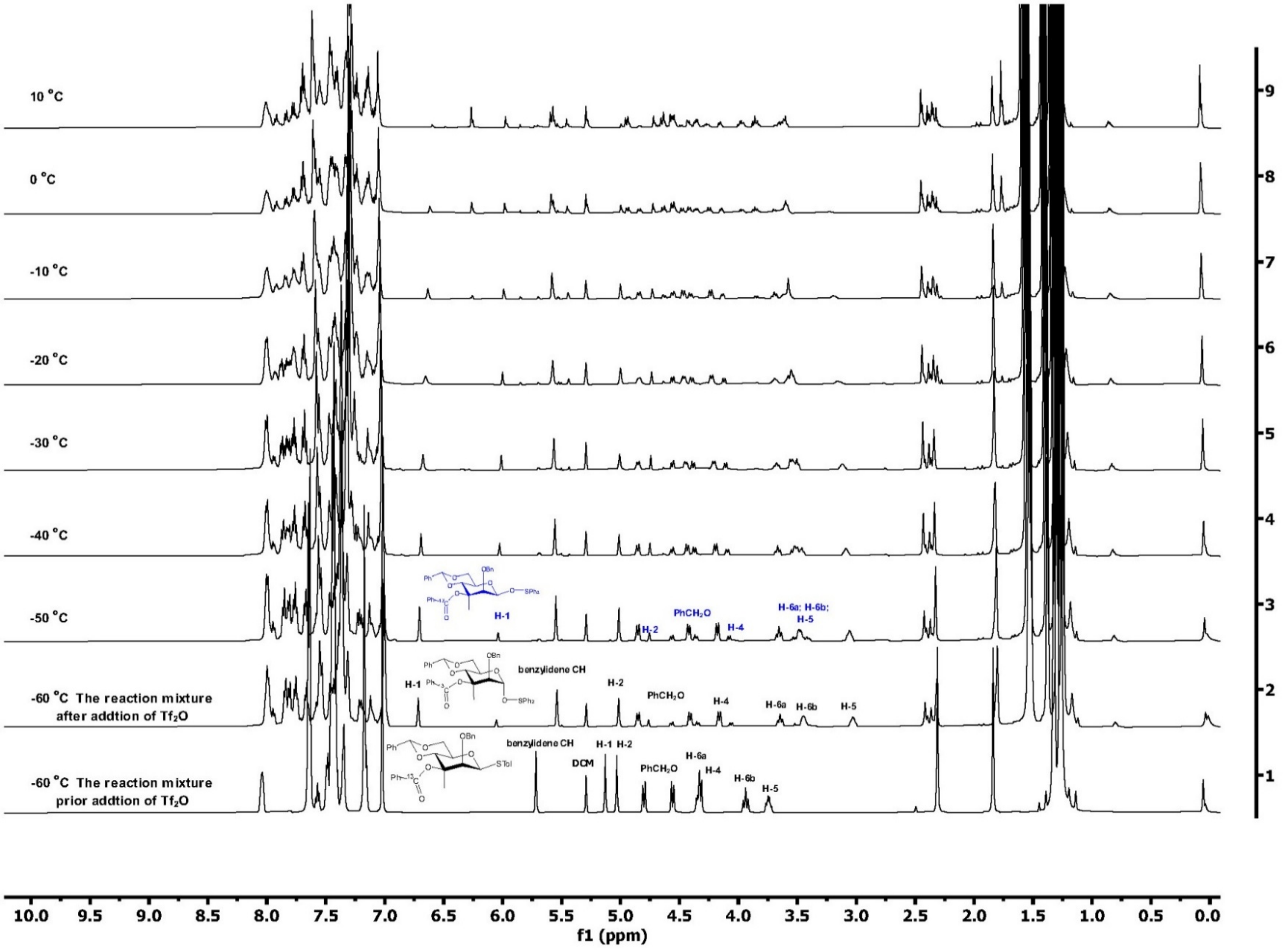

Figure S228. VT ^{1}H NMR (500 MHz, CD_2Cl_2) study of *p*-methylphenyl 3-*O*-(benzoyl-α-^{13}C)-2-*O*-benzyl-4,6-*O*-benzylidene-3-*C*-methyl-thio-β-D-mannopyranoside **^{13}C-38** (COSY at -30 °C):

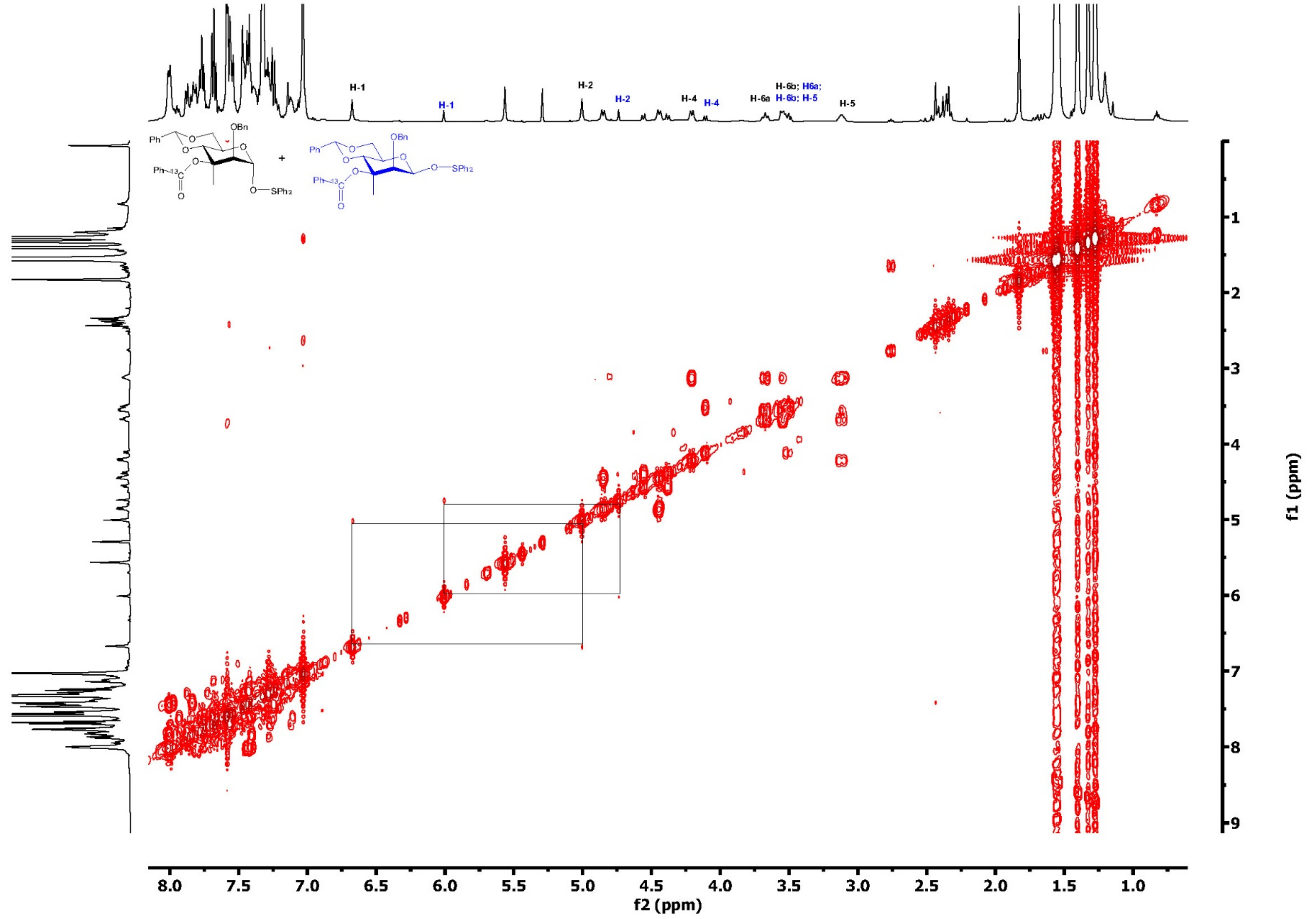

Figure S229. VT $^{13}C\{^1H\}$ NMR (126 MHz, CD_2Cl_2) study of *p*-methylphenyl 3-*O*-(benzoyl-α-^{13}C)-2-*O*-benzyl-4,6-*O*-benzylidene-3-*C*-methyl-thio-β-D-mannopyranoside **^{13}C-38**:

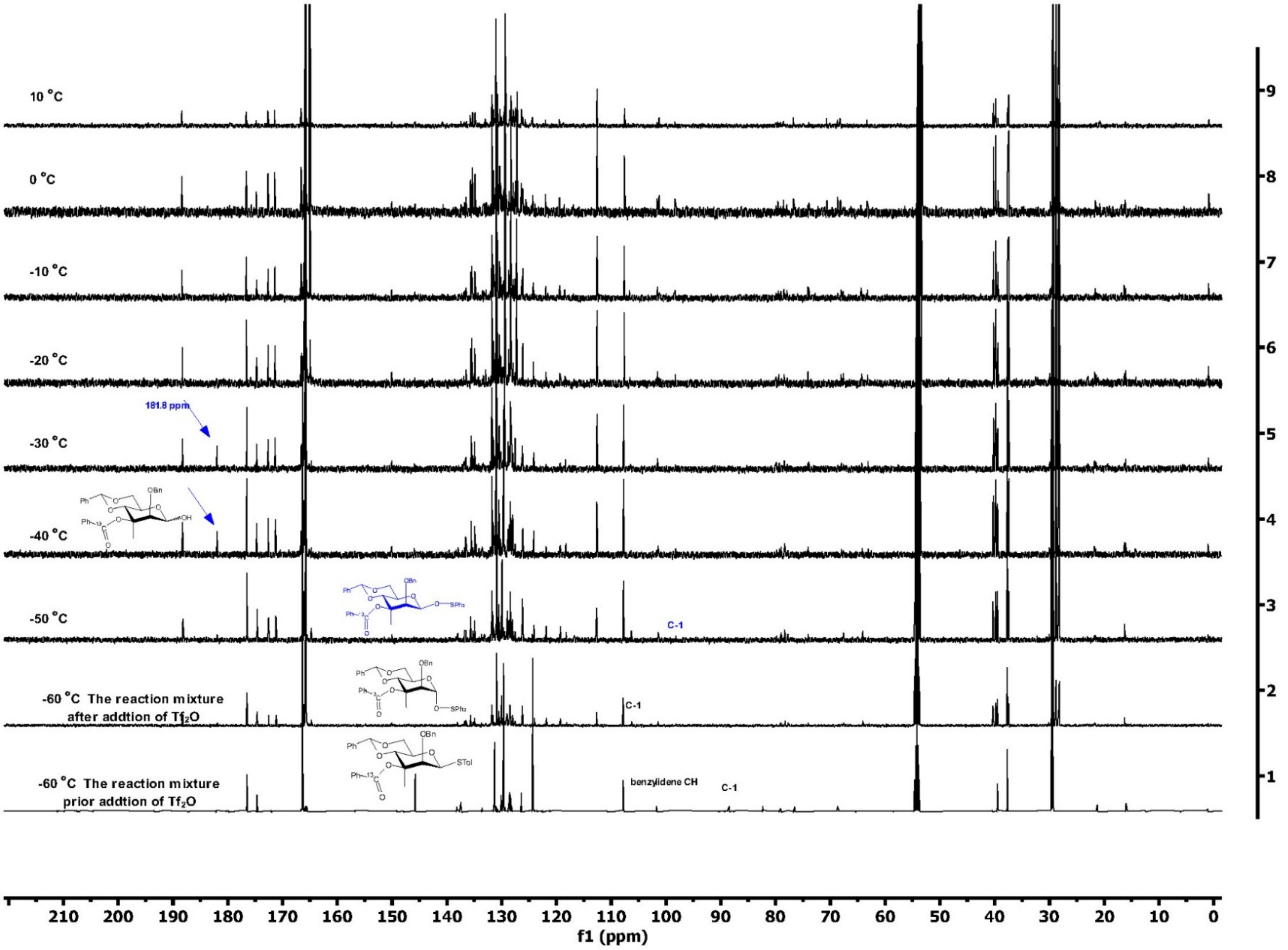

Figure S230. VT ^{19}F NMR (471 MHz, CD_2Cl_2) study of *p*-methylphenyl 3-*O*-(benzoyl-α-^{13}C)-2-*O*-benzyl-4,6-*O*-benzylidene-3-*C*-methyl-thio-β-D-mannopyranoside **^{13}C-38**:

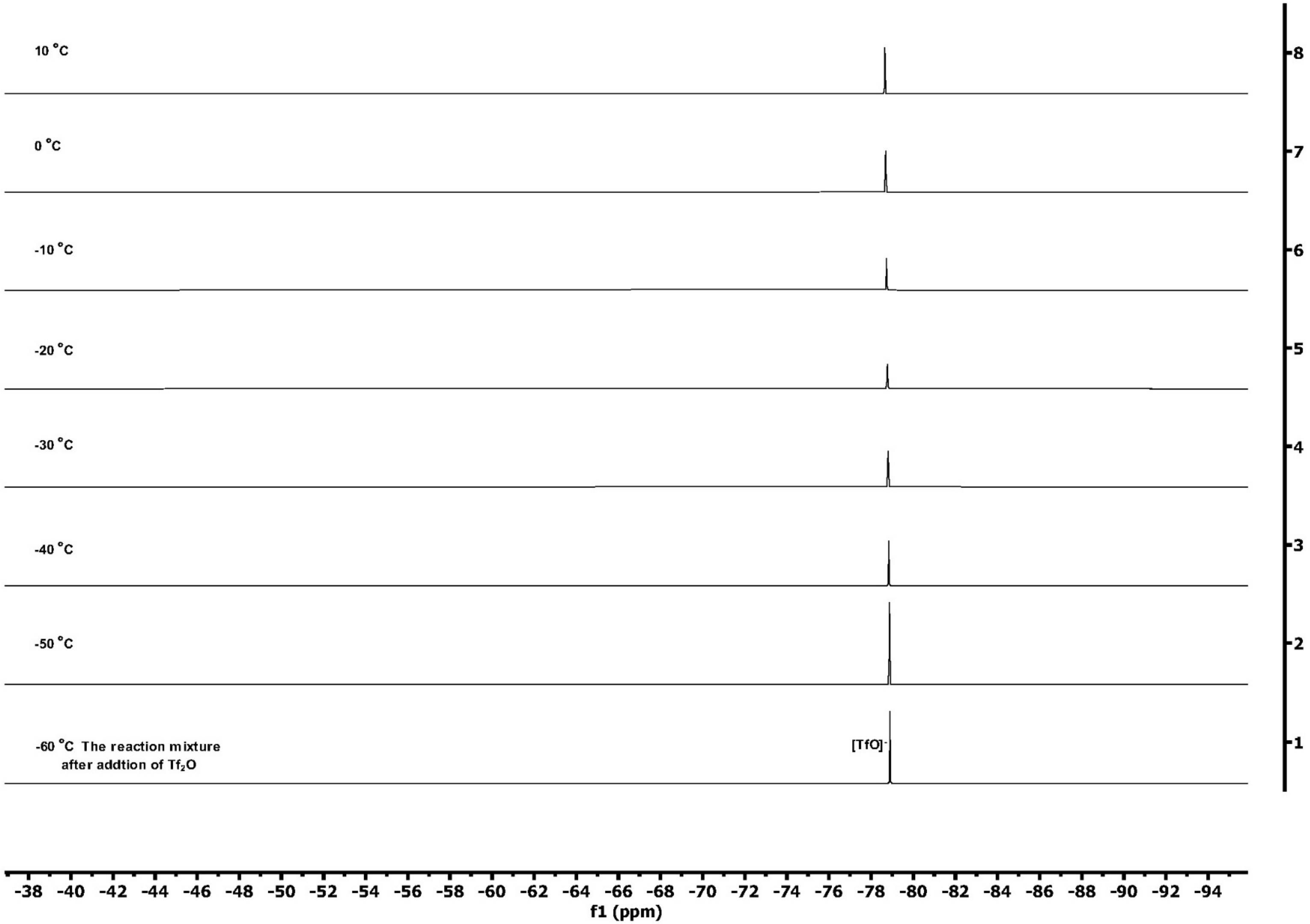

Figure S231. VT ^{1}H NMR (500 MHz, CD_2Cl_2) study of *p*-methylphenyl 3-*O*-(benzoyl-α-^{13}C)-2-*O*-benzyl-4,6-*O*-benzylidene-3-*C*-methyl-thio-β-D-mannopyranoside **^{13}C-38** (HSQC at -60 °C):

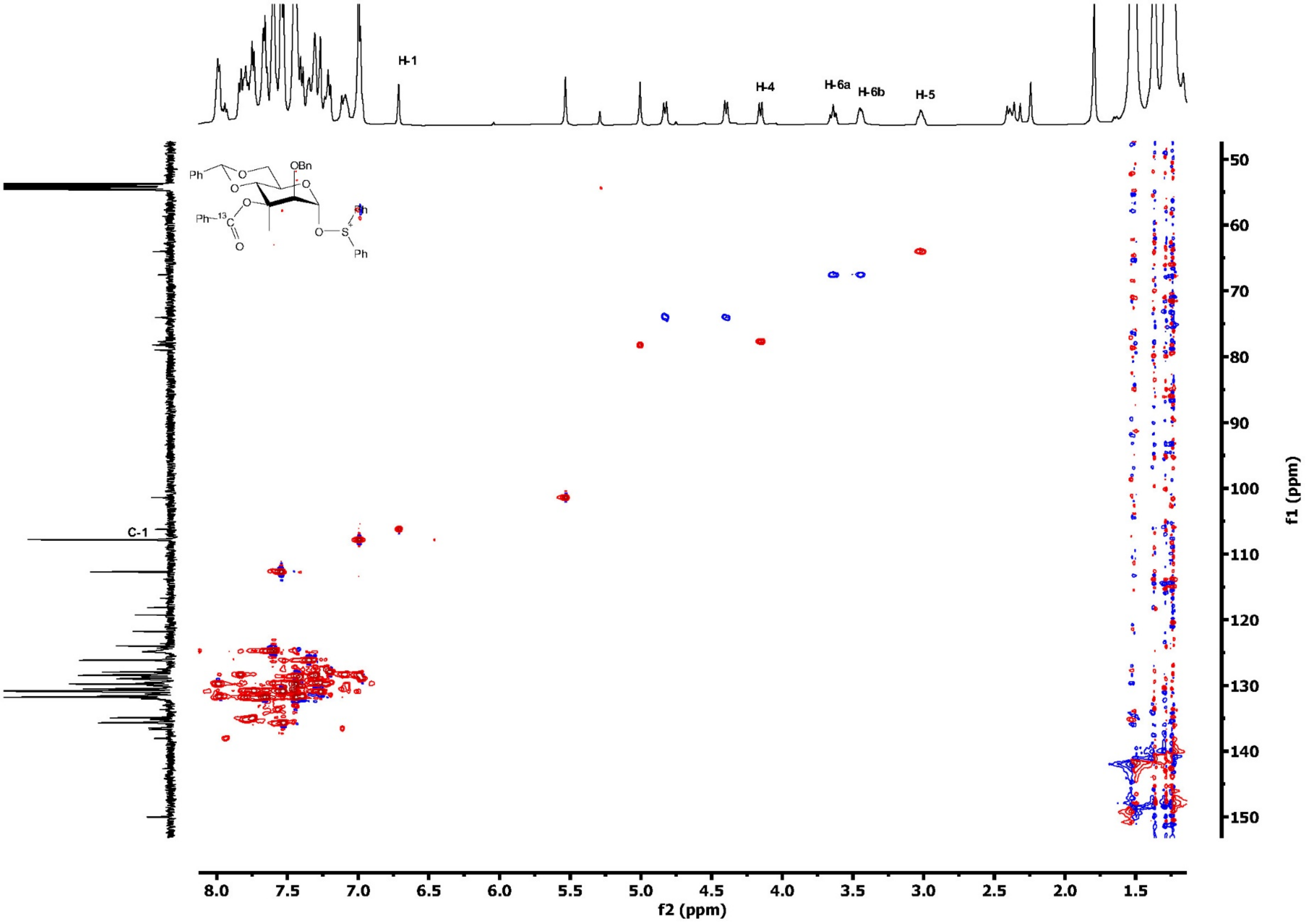

Figure S232. VT $^{13}C\{H\}$ NMR (126 MHz, CD_2Cl_2) study of *p*-methylphenyl 3-*O*-(benzoyl-α-^{13}C)-2-*O*-benzyl-4,6-*O*-benzylidene-3-*C*-methyl-thio-β-D-mannopyranoside **^{13}C-38** ($^{13}C\{H\}$ DEPT at -60 °C):

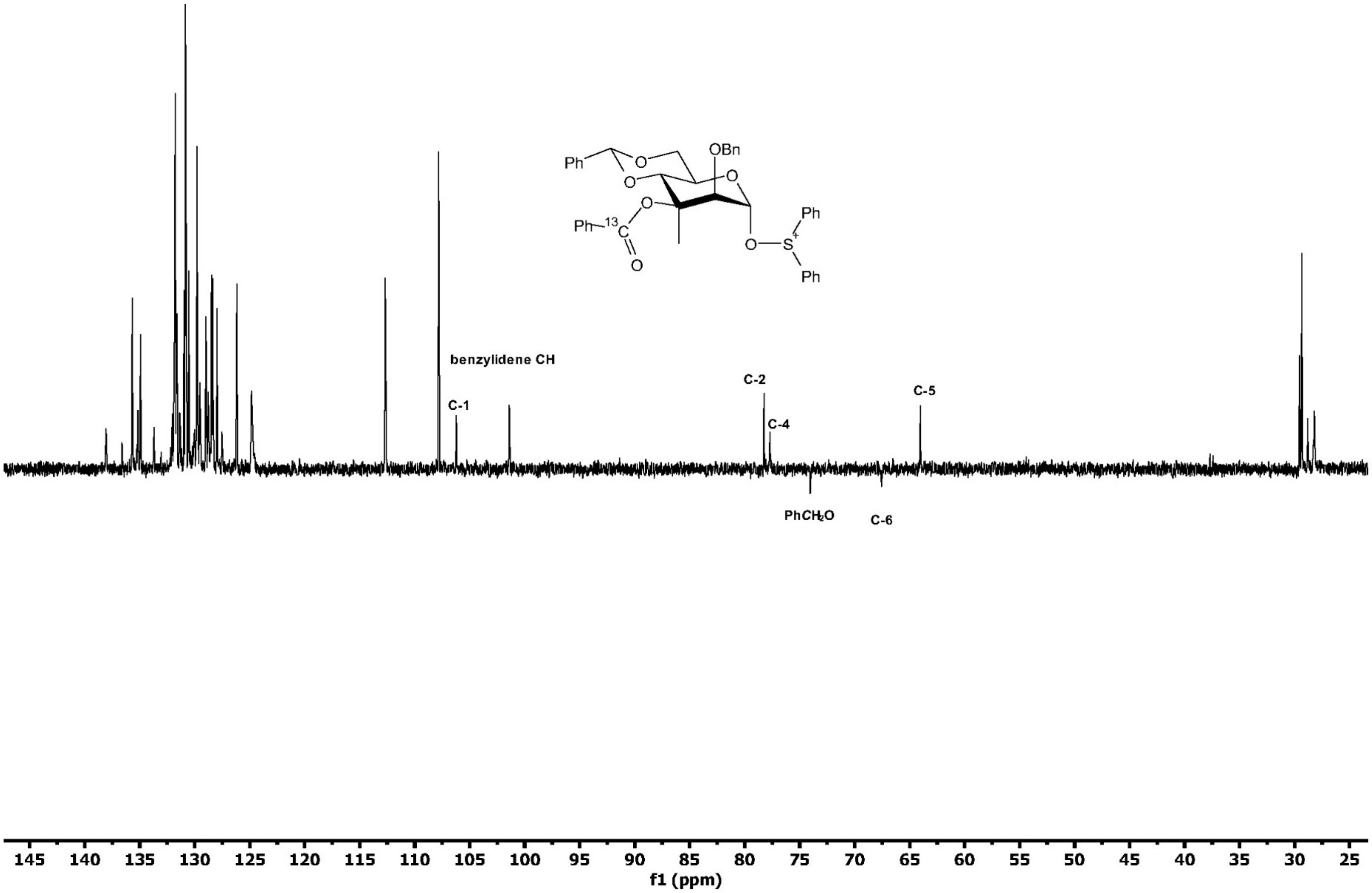

Figure S233. VT $^{13}C\{H\}$ NMR (126 MHz, CD_2Cl_2) study of *p*-methylphenyl 3-*O*-(benzoyl-α-^{13}C)-2-*O*-benzyl-4,6-*O*-benzylidene-3-*C*-methyl-thio-β-D-mannopyranoside **^{13}C-38** (C-H coupled and decoupled at -60 °C):

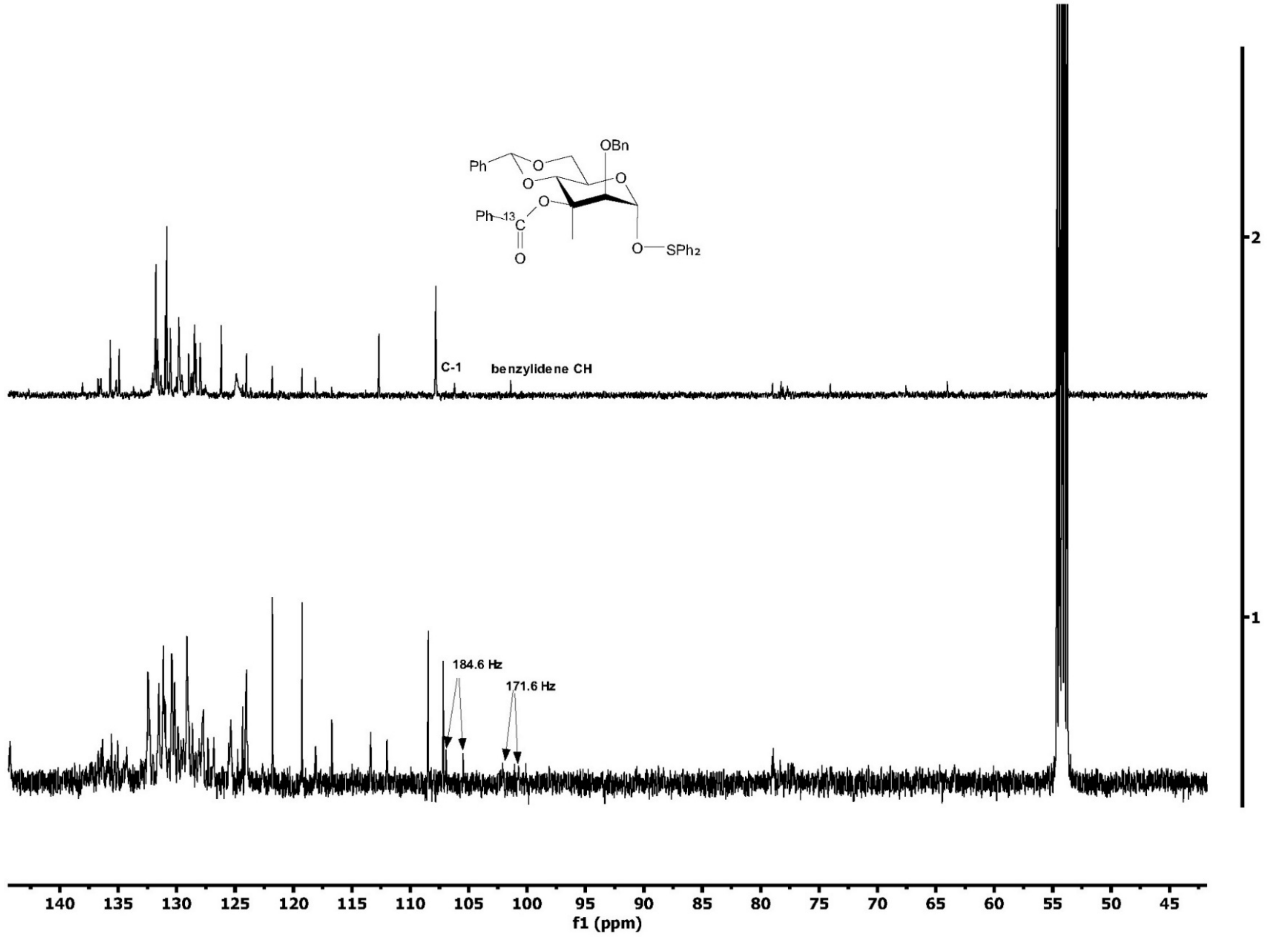

Figure S234. VT ^{1}H NMR (500 MHz, CD_2Cl_2) study of *p*-methylphenyl 3-*O*-(benzoyl-α-^{13}C)-2-*O*-benzyl-4,6-*O*-benzylidene-3-*C*-methyl-thio-α-D-mannopyranoside **^{13}C-45**:

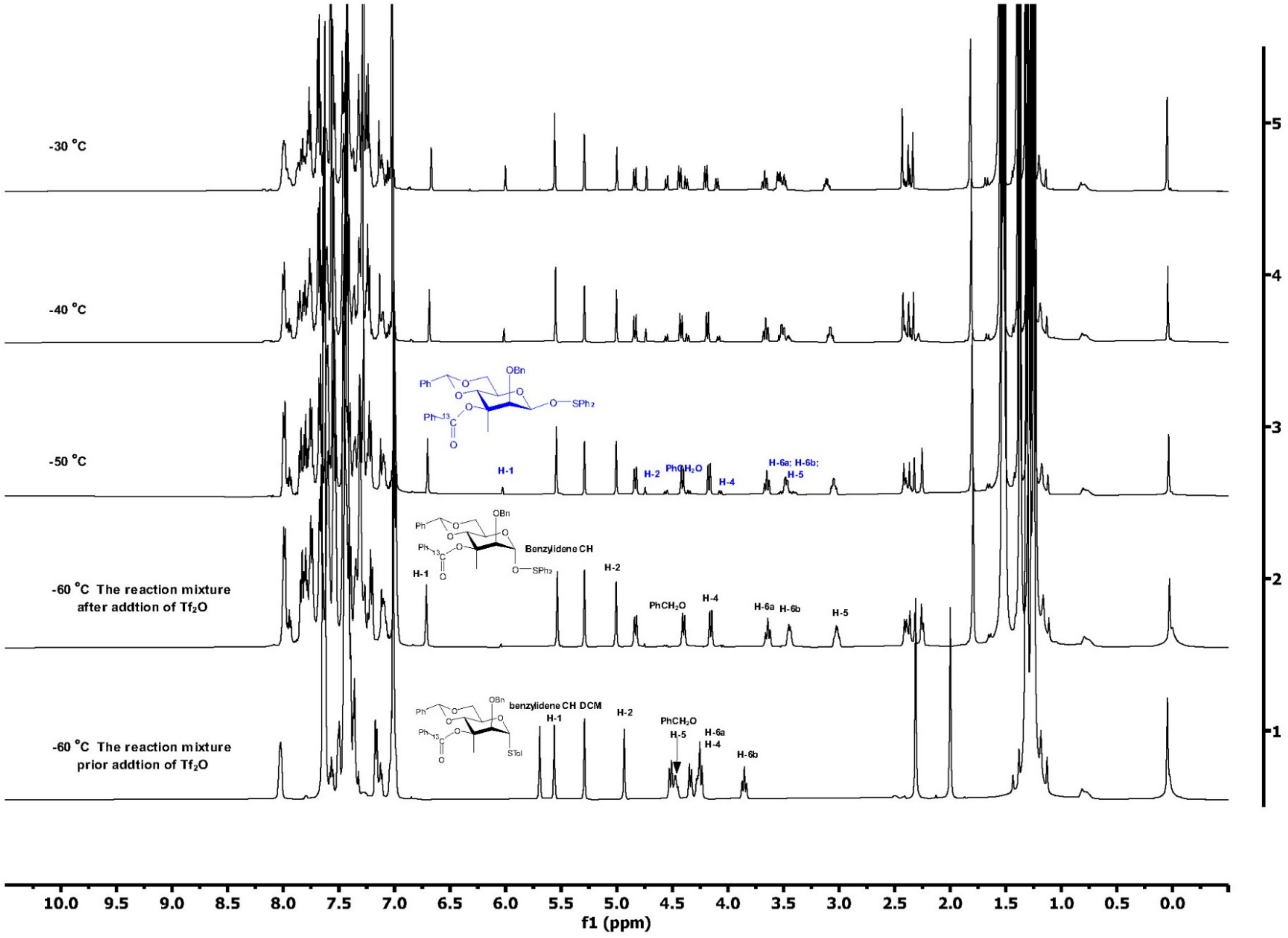

Figure S235. VT $^{13}C\{H\}$ NMR (126 MHz, CD_2Cl_2) study of *p*-methylphenyl 3-*O*-(benzoyl-α-^{13}C)-2-*O*-benzyl-4,6-*O*-benzylidene-3-*C*-methyl-thio-α-D-mannopyranoside **^{13}C-45**:

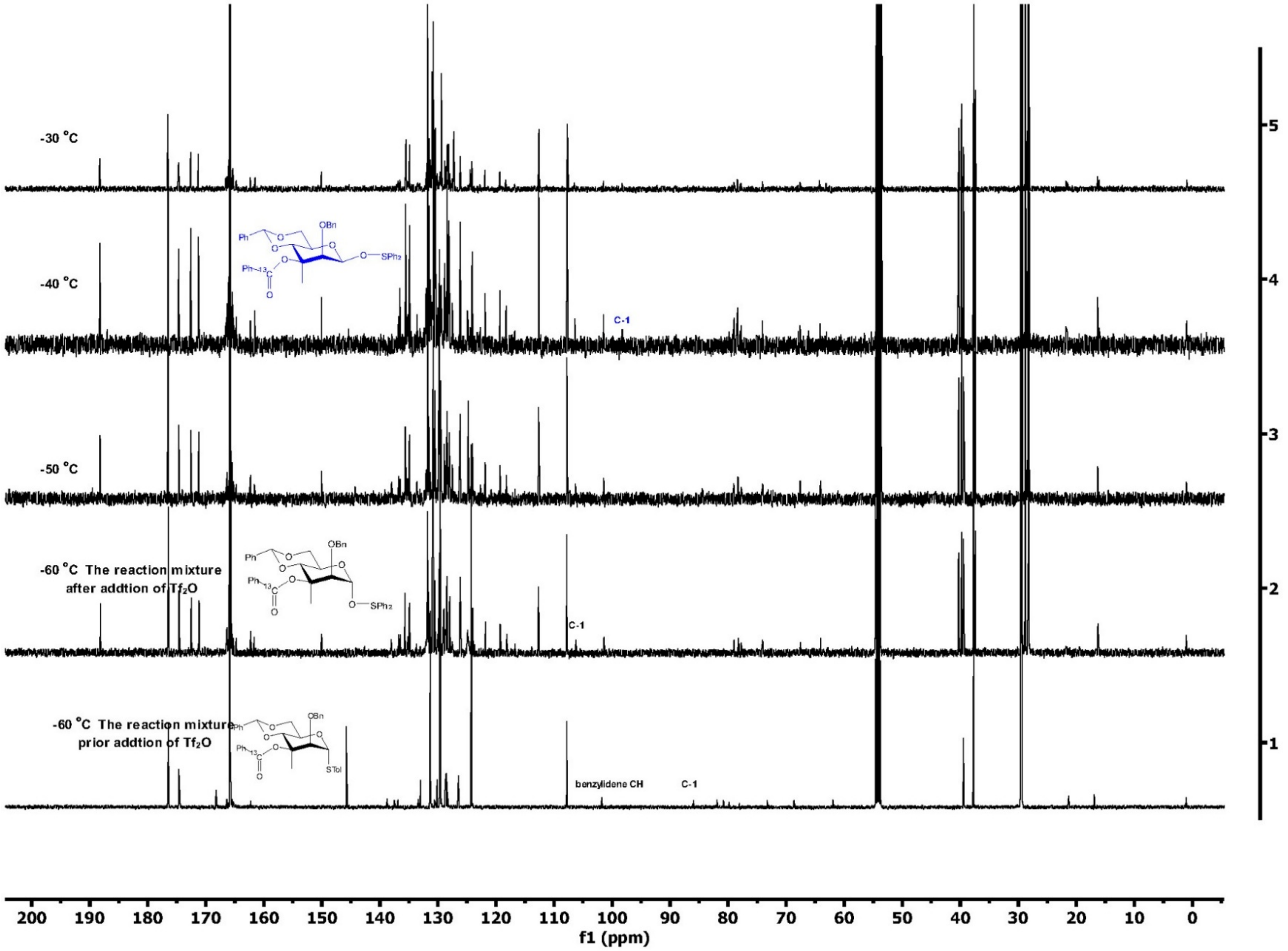

Supplement: Supplementary file 1 [file jo5c01978_si_001.pdf]
